# National, regional, and global trends in insufficient physical activity among adults from 2000 to 2022: a pooled analysis of 507 population-based surveys with 5·7 million participants

**DOI:** 10.1016/S2214-109X(24)00150-5

**Published:** 2024-06-25

**Authors:** Tessa Strain, Seth Flaxman, Regina Guthold, Elizaveta Semenova, Melanie Cowan, Leanne M Riley, Fiona C Bull, Gretchen A Stevens, Country Data Author Group

**Affiliations:** a Physical Activity for Health Research Centre, University of Edinburgh, Edinburgh, UK; b Department of Computer Science, University of Oxford, Oxford, UK; c Department of Maternal, Newborn, Child and Adolescent Health, and Ageing, Imperial College London, London, UK; d Department of Epidemiology and Biostatistics, Imperial College London, London, UK; e Department of Noncommunicable Diseases, Rehabilitation & Disability, World Health Organization, Geneva, Switzerland; f Department of Health Promotion, World Health Organization, Geneva, Switzerland; g Department of Sport Science Exercise and Health, School of Human Sciences, University of Western Australia, Perth, Australia

## Abstract

**Background:**

Insufficient physical activity increases the risk of non-communicable diseases, poor physical and cognitive function, weight gain, and mental ill-health. Global prevalence of adult insufficient physical activity was last published for 2016, with limited trend data. We aimed to estimate the prevalence of insufficient physical activity for 197 countries and territories, from 2000 to 2022.

**Methods:**

We collated physical activity reported by adults (aged ≥18 years) in population-based surveys. Insufficient physical activity was defined as not doing 150 minutes of moderate-intensity activity, 75 minutes of vigorous-intensity activity, or an equivalent combination per week. We used a Bayesian hierarchical model to compute estimates of insufficient physical activity by country or territory, year, age, and sex. We assessed whether countries or territories, regions, and the world would meet the global target of a 15% relative reduction of the prevalence of insufficient physical activity by 2030 if 2010–22 trends continue.

**Findings:**

We included 507 surveys across 163 countries and territories. The global age-standardised prevalence of insufficient physical activity was 31·3% (95% uncertainty interval 28·6–34·0) in 2022, an increase from 23·4% (21·1–26·0) in 2000 and 26·4% (24·8–27·9) in 2010. Prevalence was increasing in 103 (52%) of 197 countries and territories and six (67%) of nine regions, and was declining in the remainder. Prevalence was 5 percentage points higher among female (33·8% [29·9–37·7]) than male (28·7% [25·0–32·6]) individuals. Insufficient physical activity increased in people aged 60 years and older in all regions and both sexes, but age patterns differed for those younger than 60 years. If 2010–22 trends continue, the global target of a 15% relative reduction between 2010 and 2030 will not be met (posterior probability <0·01); however, two regions, Oceania and sub-Saharan Africa, were on track with considerable uncertainty (posterior probabilities 0·70–0·74).

**Interpretation:**

Concerted multi-sectoral efforts to reduce insufficient physical activity levels are needed to meet the 2030 target. Physical activity promotion should not exacerbate sex, age, or geographical inequalities.

**Funding:**

Ministry of Public Health, Qatar, and World Health Organization.

**Translations:**

For the Spanish and Portuguese translations of the abstract see Supplementary Materials section.

## Introduction

Regular physical activity reduces the risk of non-communicable diseases, poor physical and cognitive function, and mental ill-health,^1^, ^2^ and has benefits for mental wellbeing and weight maintenance.^1^, ^2^ The 2020 WHO physical activity guidelines recommend adults do at least 150 mins of moderate-intensity activity per week, 75 mins of vigorous-intensity activity, or an equivalent combination to confer many of these benefits.^2^ Individuals not meeting this aerobic activity recommendation are considered to be insufficiently physically active—this applies to all adults (aged 18 years and older), including those living with chronic conditions or disabilities, and pregnant or postpartum people. The World Health Assembly (WHA) set a target of a 15% relative reduction in insufficient physical activity between 2010 and 2030.^3^, ^4^ There is inconsistent and insufficient monitoring of other behaviours included in the physical activity guidelines (muscle strengthening, balance activities [for older adults aged ≥65 years], and sedentary time^2^) to produce global estimates and targets.^5^, ^6^

Previous studies have presented comparable estimates of adult insufficient physical activity for countries or regions worldwide,^7^, ^8^, ^9^ the most recent of which covered 168 countries and territories.^10^ The study by Guthold and colleagues^10^ was the only one to estimate time trends, which were based on data from 65 countries that had at least two comparable surveys. The results suggested global prevalence was stable between 2001 and 2016, but the trajectories varied considerably between countries and regions. Many countries have collected additional data since 2016.

**Research in context**

**Evidence before this study**

WHO has previously presented internationally comparable estimates of insufficient physical activity among adults, the most recent of which (Guthold and colleagues, 2018) estimated the global prevalence to be 27·5% in 2016 using data from 358 population-based surveys. This study was the first analysis to estimate trends in insufficient physical activity, and it found no change in global prevalence since 2001. However, global and regional trends in insufficient physical activity were estimated using data from only 65 countries and territories, with considerable variation in trajectories. To find other previous presentations of adult prevelance of insufficient physical activity for countries worldwide, we used various search strategies, including a systematic search of PubMed using terms including “global insufficient physical activity”, “global physical activity”, and “global physical inactivity”, with no language restriction, covering literature from Jan 1, 2000 to Oct 24, 2023. Our search confirmed that the work of Guthold and colleagues, 2018, was the most recent and most comprehensive in terms of data coverage and the only one to estimate time trends.

**Added value of this study**

This study provides estimates of global, regional, and country levels of insufficient physical activity for 2000–22. For the first time, we estimated insufficient physical activity prevalence for 197 countries and territories, regionally and globally; made estimates for seven age groups; and tracked progress towards the global target of reducing the prevalence of insufficient physical activity by a relative 15% by 2030.

**Implications of all the available evidence**

Nearly a third of the global population did not meet the recommended levels of physical activity in 2022, with notable inequalities by sex, age, region, and country. Although most countries, territories, and regions are not on track to meet the global target, lessons can be learned from countries which are on track. Promotion efforts, such as the multi-sectoral, whole systems approach recommended in the Global Physical Activity Action Plan 2018–2030, are needed to promote physical activity while reducing inequalities.

This study aimed to update the global estimates of insufficient physical activity (including time trends from 2000 to 2022) for 197 countries and territories by sex and age group, using a revised modelling framework to facilitate the estimation of trends and progress towards the global target.

## Methods

### Study design

Our study involved three steps: collating population-based data on insufficient physical activity prevalence among adults (aged ≥18 years); estimating insufficient physical activity prevalence between 2000 and 2022 by age group (aged 18–29, 30–39, 40–49, 50–59, 60–69, 70–79, and ≥80 years) and sex for 197 countries and territories, organised into nine analytical groups based on geography and developmental level, hereafter called countries and regions (appendix 3 p 2); and assessing progress toward the 2030 target of reducing insufficient physical activity prevalence by a relative 15%, using 2010 as the baseline.^11^ We defined insufficient physical activity as not meeting the WHO recommendations for moderate-to-vigorous aerobic activity: at least 150 minutes of moderate-intensity activity per week, 75 minutes of vigorous-intensity activity, or an equivalent combination.^2^ These estimates have been documented following the Guidelines for Accurate and Transparent Health Estimates Reporting (appendix 3 p 3).^12^

### Data sources

We collated data on self-reported physical activity that were nationally representative or covered at least three areas within the country. We included individual-level anonymised data when available. We included summary statistics by age and sex if insufficient physical activity prevalence was reported according to the current WHO recommendations^2^ or the International Physical Activity Questionnaire (IPAQ) low category.^13^ We included data if questionnaires assessed total weekly duration of moderate-intensity and vigorous-intensity activity across all domains (including work and household, travel, and leisure), if data were collected in or after the year 2000, and if the minimum survey sample size was 200 (appendix 3 p 4).

Data sources were identified through the following search strategies and assessed for inclusion: data from Guthold and colleagues;^10^ subsequent waves of data collection from those surveys; data shared with the WHO (eg, from the WHO STEPwise approach to surveillance); all surveys named in the 2021 WHO Country Capacity Survey in response to a query about the latest nationally representative physical activity data source;^14^ surveys identified by academic contacts; a targeted Google search; conducting a systematic review of the literature targeting the 35 most populous countries; and conducting a formal consultation with WHO member states (appendix 3 p 5). Our dataset closed on Feb 5, 2024.

Individual-level data collected using the Global Physical Activity Questionnaire (GPAQ) were processed according to the WHO protocol,^15^ whereas those collected using the IPAQ were processed using an adapted protocol harmonised to the GPAQ protocol (appendix 3 p 6). Other questionnaires were processed according to survey-specific protocols and harmonised as closely as possible with the GPAQ protocol. The prevalence of insufficient physical activity was calculated by sex and age group, applying survey weights and accounting for complex survey design when appropriate and when variables were available. We created a single dataset comprising these prevalence estimates and those available as summary statistics (appendix 3 pp 6–7).

We adjusted prevalences from surveys only representing urban areas and those reporting prevalence in the IPAQ low scoring category using regression equations developed by Guthold and colleagues (appendix 3 p 8).^10^

### Statistical analysis

We used hierarchical Bayesian probit regression models to estimate the prevalence of insufficient physical activity during 2000–22 by country and age. Separate models were fit for males and females (appendix 3 pp 9–11). Briefly, the model used all available data to make estimates for each country–year–age unit. Estimates were informed by data not only from that unit itself, but data from other ages and years in that country, and from other countries, particularly those in the same region with data in similar time periods. The hierarchical model shares information to a greater degree when data are non-existent or weakly informative (eg, data were inconsistent, subnational, or had a small sample size). Age patterns were modelled using natural cubic splines with 2 knots at age 30 years and 60 years and were allowed to vary by region. Trends over time were modelled linearly. As self-reported physical activity prevalence can vary by questionnaire,^16^ indicator variables were included for three survey types, with GPAQ as the reference: IPAQ-short, Eurobarometer 2013–22, and other (comprising various instruments). GPAQ was the reference category as it is recommended by the WHO for physical activity surveillance. Age-standardised adult prevalence of obesity was included as a country-specific, time-varying covariate.^17^ An additional variance term accounted for unobserved design factors (eg, sample design and season) that would lead to variance beyond that expected due to sample size. This term also accounted for greater variation in subnational data compared with nationally representative data. We estimated the prevalence of insufficient physical activity for all 197 countries, regardless of data availability.

We fit the models using the brms package in R.^18^ We obtained 4000 samples from the parameters' posterior, which were used to compute 4000 posterior samples of the prevalence of insufficient physical activity for each country, year, age, and sex. With each of the 4000 sampled prevalence values, we calculated crude and age-standardised prevalence for adults aged 18 years and older by sex and for both sexes, by country, by analytical region, by 2022 World Bank income group, and for the globe (appendix 3 p 11). Population data were from World Population Prospects 2022.^19^ All reported uncertainty intervals (UIs) are 95% Bayesian credible intervals, computed as the 2·5th and 97·5th percentile of the 4000 sampled prevalence values.

We assessed progress towards the target of a 15% relative reduction in insufficient physical activity prevalence between 2010 and 2030. To do so, we assumed that 2010–22 trends continue to project the prevalence of insufficient physical activity to 2030 globally, by country, by region, by sex, and for both sexes (appendix 3 p 13). We compared this projection with the estimate for 2010 to assess whether the target would be met if estimated 2010–22 trends continue to 2030. We also computed the posterior probability of meeting the target if 2010–22 trends continue, a measure of how certain we are that the estimated trends would be sufficient to meet the target if they continue. We combined our central estimate of whether populations were on track with our measure of certainty to categorise countries, regions, and the world as follows: on track with higher certainty (posterior probability of meeting the target ≥0·80), on track with lower certainty (posterior probability <0·80), off track with higher certainty (posterior probability ≤0·20), and off track with lower certainty (posterior probability >0·20). We also computed the posterior probability that 2010–22 trends were true increases or decreases.

### Role of the funding source

The funders of the study had no role in study design, data collection, data analysis, data interpretation, or writing of the report.

## Results

**Figure 1:**
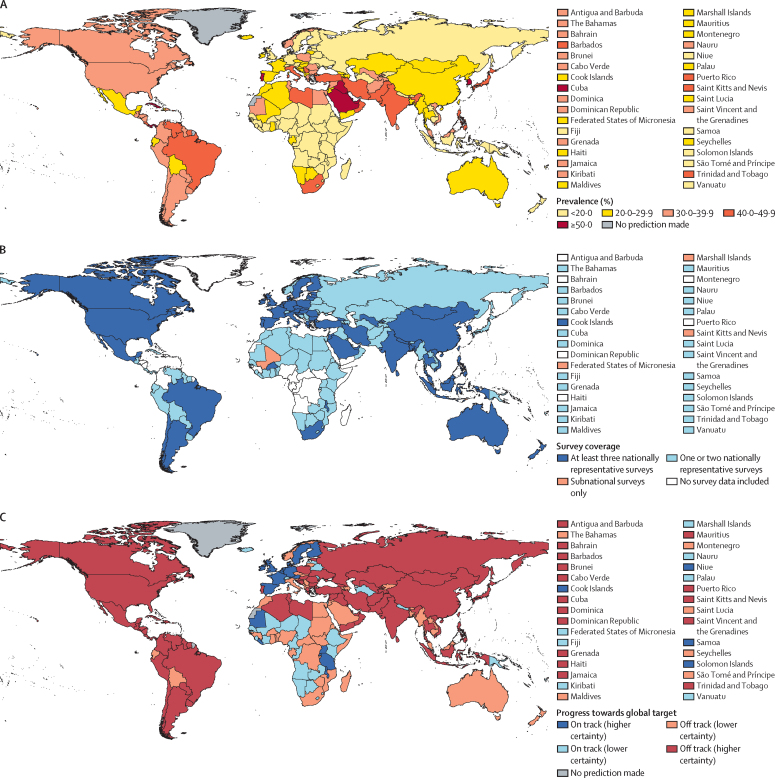
Map of (A) age-standardised prevalence of insufficient physical activity among adults aged 18 years and over in 2022, (B) data coverage and representativeness, and (C) country progress during 2010–22 toward the global target of a 15% relative reduction in insufficient physical activity prevalence among adults aged 18 years and over between 2010 and 2030 For visibility, small-area countries are listed next to a box indicating their corresponding values. Country progress towards the global target is assessed based on whether the estimated trend in insufficient physical activity during 2010–22 would be sufficient to meet the global target if trends were to continue to 2030. Higher and lower certainty indicate certainty about whether the estimated rate of change would be sufficient to meet the global target, if continued to 2030 (appendix 3 p 13).

We included 507 surveys from 163 of 197 countries, representing 93·0% of the global population (table 1, figure 1, appendix 3 pp 14–31). Most surveys were nationally representative (452 surveys from 158 countries). All countries in the high-income Asia Pacific, Oceania, and south Asia regions had at least one survey included in the analysis. Only 34 of the 49 sub-Saharan African countries had one eligible survey, representing 61·5% of the regional population. Just under half the surveys (228 [45%]) used the GPAQ, 137 used the IPAQ, and 142 used another questionnaire. 167 surveys were from before 2010, 268 from 2010–19, and 72 from 2020 or later. 34 countries had no identified eligible survey data.

**Table 1:** Distribution of included surveys by region, questionnaire, and year of data collection, and data coverage by region, questionnaire, and year

	**Countries in category (n)**	**Included surveys (n)**	**Included surveys that are nationally representative (n)**	**Countries with included surveys (n)**	**Countries with included surveys that are nationally representative (n)**	**Population (by category) covered by at least one survey**	**Population (by category) covered by at least one nationally representative survey**
**Region**							
Global	197	507	452	163	158	93·0%	92·8%
Central Asia and north Africa–Middle East	28	53	51	25	25	92·5%	92·5%
Central and eastern Europe	20	61	59	18	18	98·9%	98·9%
East and southeast Asia	14	44	40	12	11	97·6%	97·5%
High-income Asia Pacific	3	21	21	3	3	100%	100·0%
High-income western countries	28	168	149	24	24	99·0%	99·0%
Latin America and Caribbean	34	54	42	26	25	85·8%	85·8%
Oceania	14	25	18	14	12	100%	98·7%
South Asia	7	23	17	7	7	100%	100·0%
Sub-Saharan Africa	49	58	55	34	33	61·5%	59·8%
**World Bank income groups for 2022**							
Low income	26	26	24	16	15	62·1%	59·2%
Lower middle income	54	100	85	45	43	91·7%	91·7%
Upper middle income	53	112	97	46	45	97·6%	97·6%
High income	61	264	241	53	52	96·9%	96·9%
**Questionnaire type**							
GPAQ	197	228	200	119	110	81·7%	77·2%
IPAQ-short	197	137	128	83	81	77·1%	76·0%
Other	197	142	124	34	34	15·5%	15·5%
**Year of survey**							
2000–04	197	76	71	72	68	72·6%	72·6%
2005–09	197	91	76	74	63	68·9%	66·3%
2010–14	197	126	112	90	85	74·4%	54·7%
2015–19	197	142	127	100	97	81·0%	80·9%
2020–23	197	72	66	57	55	19·5%	18·0%

Globally, nearly a third of adults were insufficiently physically active (age-standardised prevalence of 31·3% [95% uncertainty interval 28·6–34·0]) in 2022 (table 2). The prevalence was highest in the high-income Asia Pacific region, closely followed by south Asia. Oceania had the lowest prevalence, followed by sub-Saharan Africa. These regional patterns were also evident in the sex-specific estimates. Prevalence was lowest among low-income countries and highest in lower-middle-income countries, although country-specific prevalence varied considerably within these categories. Country-specific prevalence ranged from 2·7% (95% UI 1·1–5·3) in Malawi to 66·1% (54·6–76·8) in the United Arab Emirates (figure 1, appendix 3 pp 32–36). The prevalence of insufficient physical activity was over 40% in 32 countries (figure 1), and exceeded 50% in the United Arab Emirates and nine other countries (ie, Kuwait, Cuba, Lebanon, South Korea, Panama, Qatar, Iraq, Portugal, and Saudi Arabia). Prevalence was under 10% in 15 countries in sub-Saharan Africa, high-income western countries, Oceania, and south Asia.

**Table 2:** Age-standardised prevalence of insufficient physical activity among adults aged 18 years and older in 2000, 2010, and 2022, projected prevalence in 2030 assuming 2010–22 trends continue, and progress during 2010–22 toward the global target of reducing the prevalence of insufficient physical activity by 15% between 2010 and 2030, by world region and by sex

			**Prevalence in 2000 (95% UI)**	**Prevalence in 2010 (95% UI)**	**Prevalence in 2022 (95% UI)**	**Prevalence in 2030 if 2010–22 trends continue (95% UI)**	**Progress towards global target during 2010–22**
**Both sexes**							
Region							
	Global		23·4% (21·1–26·0)	26·4% (24·8–27·9)	31·3% (28·6–34·0)	34·7% (30·4–39·1)	Off track, higher certainty
		Central Asia and north Africa–Middle East	29·7% (25·6–33·9)	33·6% (31·4–35·9)	38·5% (34·5–42·5)	42·0% (35·4–48·7)	Off track, higher certainty
		Central and eastern Europe	15·4% (12·4–18·8)	18·0% (15·5–20·8)	22·7% (17·2–30·3)	25·8% (17·3–38·1)	Off track, higher certainty
		East and southeast Asia	19·0% (14·7–24·0)	20·8% (18·5–23·2)	24·6% (20·2–29·6)	27·3% (19·8–35·9)	Off track, higher certainty
		High-income Asia Pacific	28·9% (16·6–44·1)	36·5% (28·4–45·5)	48·1% (40·5–55·6)	55·9% (44·0–67·3)	Off track, higher certainty
		High-income western countries	31·6% (27·8–35·6)	30·1% (27·9–32·3)	27·7% (24·3–31·4)	26·1% (21·0–31·9)	Off track, lower certainty
		Latin America and Caribbean	28·6% (23·5–34·0)	31·8% (28·6–35·1)	36·6% (32·2–41·3)	40·0% (32·8–47·4)	Off track, higher certainty
		Oceania	22·5% (15·5–30·7)	16·9% (12·7–21·9)	13·6% (6·4–24·6)	11·9% (3·4–27·9)	On track, lower certainty
		South Asia	22·4% (16·8–28·9)	32·1% (27·7–36·5)	45·4% (36·7–54·4)	54·7% (41·3–68·1)	Off track, higher certainty
		Sub-Saharan Africa	21·7% (17·8–26·4)	19·2% (16·5–22·4)	16·8% (13·2–21·2)	15·2% (10·5–21·1)	On track, lower certainty
World Bank income groups for 2022							
	Low income		18·9% (15·3–23·2)	17·7% (15·1–20·5)	17·0% (14·0–20·6)	16·4% (12·3–21·4)	Off track, lower certainty
	Lower middle income		21·4% (17·6–25·7)	28·4% (25·6–31·3)	38·2% (32·6–44·0)	45·3% (36·4–54·5)	Off track, higher certainty
	Upper middle income		21·5% (18·1–25·4)	23·4% (21·4–25·4)	26·9% (23·3–30·9)	29·3% (23·4–35·9)	Off track, higher certainty
	High income		30·4% (26·7–34·2)	31·6% (29·3–33·8)	32·7% (29·9–35·6)	33·4% (29·1–37·9)	Off track, higher certainty
**Male**							
Region							
	Global		21·0% (17·9–24·5)	24·1% (22·1–26·3)	28·7% (25·0–32·6)	32·0% (25·9–38·4)	Off track, higher certainty
		Central Asia and north Africa–Middle East	24·8% (19·9–30·5)	28·5% (25·7–31·4)	32·8% (27·9–37·9)	35·9% (28·0–44·6)	Off track, higher certainty
		Central and eastern Europe	14·0% (10·3–18·3)	17·3% (14·2–20·9)	22·5% (16·1–32·2)	26·2% (16·2–41·5)	Off track, higher certainty
		East and southeast Asia	19·0% (13·1–26·2)	21·9% (18·5–25·6)	26·9% (20·1–34·3)	30·6% (19·1–43·4)	Off track, higher certainty
		High-income Asia Pacific	25·0% (10·8–45·3)	32·5% (22·5–44·9)	44·4% (35·1–54·2)	52·6% (37·1–68·2)	Off track, higher certainty
		High-income western countries	27·5% (22·7–32·7)	26·6% (23·8–29·4)	24·2% (19·9–28·9)	22·6% (16·2–29·8)	Off track, lower certainty
		Latin America and Caribbean	26·4% (20·1–33·6)	28·6% (24·7–32·7)	31·9% (25·8–38·2)	34·2% (24·8–44·0)	Off track, higher certainty
		Oceania	19·6% (11·4–30·5)	13·0% (8·7–18·8)	8·9% (3·2–21·0)	7·2% (1·2–24·0)	On track, higher certainty
		South Asia	18·5% (11·6–26·6)	26·7% (21·2–32·7)	38·4% (26·4–50·7)	46·8% (27·7–65·8)	Off track, higher certainty
		Sub-Saharan Africa	18·4% (13·8–24·3)	16·5% (13·4–20·8)	14·3% (10·2–19·4)	12·8% (7·7–19·8)	On track, lower certainty
World Bank income groups for 2022							
	Low income		15·7% (11·7–20·7)	14·6% (11·8–17·9)	13·9% (10·6–18·2)	13·3% (8·7–19·4)	Off track, lower certainty
	Lower middle income		17·8% (13·1–23·4)	23·9% (20·1–27·9)	32·4% (24·7–40·4)	38·8% (26·3–51·5)	Off track, higher certainty
	Upper middle income		20·8% (16·0–26·6)	23·6% (20·7–26·7)	27·8% (22·5–34·0)	30·9% (21·9–41·0)	Off track, higher certainty
	High income		26·6% (22·1–31·5)	28·3% (25·6–31·3)	29·6% (26·2–33·3)	30·5% (25·1–36·5)	Off track, higher certainty
**Female**							
Region							
	Global		25·6% (21·9–29·6)	28·6% (26·4–30·9)	33·8% (29·9–37·7)	37·5% (31·2–43·9)	Off track, higher certainty
		Central Asia and north Africa–Middle East	34·4% (27·8–41·1)	38·7% (35·2–42·3)	44·2% (38·4–50·5)	48·1% (38·6–58·1)	Off track, higher certainty
		Central and eastern Europe	16·6% (12·1–21·8)	18·7% (15·1–23·0)	22·9% (15·1–34·8)	25·6% (14·1–45·0)	Off track, higher certainty
		East and southeast Asia	19·0% (13·1–26·3)	19·7% (16·7–22·9)	22·2% (16·7–28·7)	24·1% (14·7–35·2)	Off track, higher certainty
		High-income Asia Pacific	32·7% (14·1–56·5)	40·5% (27·8–54·5)	51·9% (40·6–62·9)	59·3% (41·5–75·8)	Off track, higher certainty
		High-income western countries	35·6% (29·9–41·8)	33·5% (30·2–37·0)	31·2% (25·9–37·2)	29·7% (21·6–39·2)	Off track, lower certainty
		Latin America and Caribbean	30·6% (22·9–38·6)	34·9% (29·9–40·0)	41·2% (34·3–48·5)	45·6% (35·1–56·7)	Off track, higher certainty
		Oceania	25·5% (15·5–38·4)	20·9% (14·3–29·7)	18·4% (6·7–39·3)	17·4% (3·2–49·0)	On track, lower certainty
		South Asia	26·4% (17·6–37·3)	37·6% (30·8–44·2)	52·6% (39·2–65·8)	62·3% (42·6–80·1)	Off track, higher certainty
		Sub-Saharan Africa	24·7% (18·7–32·0)	21·6% (17·6–26·7)	19·1% (13·6–26·5)	17·5% (10·3–27·5)	On track, lower certainty
World Bank income groups for 2022							
	Low income		21·9% (16·3–28·5)	20·5% (16·5–25·0)	19·9% (14·9–25·7)	19·4% (12·7–27·8)	Off track, lower certainty
	Lower middle income		24·9% (18·9–32·0)	32·9% (28·6–37·2)	44·0% (35·6–52·2)	51·9% (38·5–64·4)	Off track, higher certainty
	Upper middle income		22·1% (17·3–27·9)	23·2% (20·6–26·1)	26·0% (21·3–31·4)	27·9% (20·4–36·9)	Off track, higher certainty
	High income		34·1% (28·3–40·1)	34·8% (31·4–38·2)	35·8% (31·5–40·4)	36·5% (29·7–44·0)	Off track, higher certainty

For progress toward the global target, regions are assigned categories based on whether the estimated trend in insufficient physical activity during 2010–22 would be sufficient to meet the global target if trends were to continue to 2030. Higher and lower certainty indicate certainty about whether the estimated rate of change would be sufficient to meet the global target, if continued to 2030 (appendix 3 p 13). UI=uncertainty interval.

The global prevalence of insufficient physical activity in 2022 was 5 percentage points higher in female (33·8% [95% UI 29·9–37·7]) than male (28·7% [25·0–32·6]) individuals (table 2). South Asia was the region with the largest absolute sex difference—female prevalence (52·6% [39·2–65·8]) was 14 percentage points higher than male prevalence (38·4% [26·4–50·7]). Female prevalence was more than 10 percentage points higher than male prevalence in approximately a third of countries (61 of 197 countries). In Afghanistan, Pakistan, Cuba, Guyana, Iran, and The Bahamas, the female prevalence of insufficient activity was at least 20 percentage points higher than the male prevalence.

**Figure 2:**
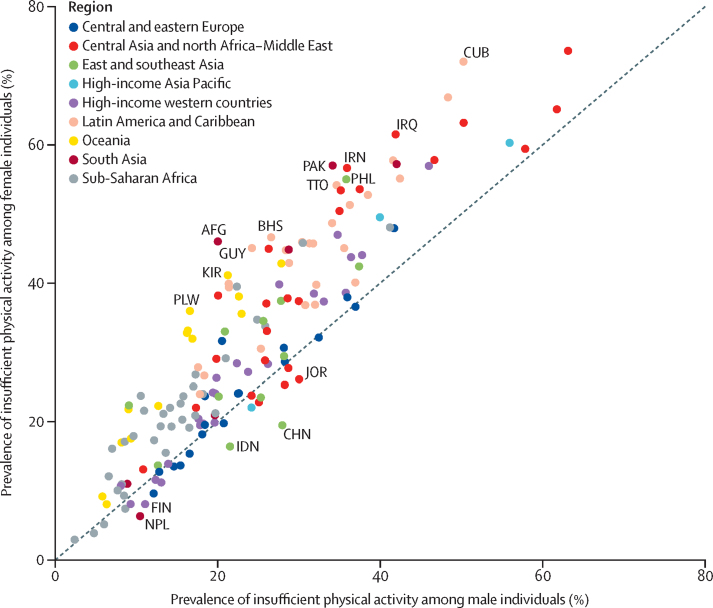
Male versus female age-standardised prevalence of insufficient physical activity in 2022, for adults aged 18 years and older, by country and region Countries with included survey data are plotted. The Pearson correlation coefficient for male and female values is 0·91. The median country difference between female and male prevalence of insufficient physical activity in 2022 was 6 percentage points (IQR 2–12). Countries with the largest difference in prevalence in each direction are labelled. AFG=Afghanistan. BHS=Bahamas. CHN=China. CUB=Cuba. FIN=Finalnd. GUY=Guyana. IDN=Indonesia. IRN=Iran. IRQ=Iraq. JOR=Jordan. KIR=Kiribati. NPL=Nepal. PAK=Pakistan. PHL=Philippines. PLW=Palau. TTO=Trinidad and Tobago.

This pattern of sex inequality was not evident across all regions or countries (figure 2, appendix 3 p 38). For example, in east and southeast Asia, male prevalence was 26·9% (20·1–34·3) and female prevalence was 22·2% (16·7–28·7%), whereas male and female prevalence was similar in central and eastern Europe (table 2). The prevalence of insufficient activity was higher in male than female individuals in 13% (26 of 197) of countries, although the uncertainty intervals overlapped; this included China, where there was an 8 percentage point difference (28·0% [18·7–38·6] for male individuals, 19·5% [11·6–28·8] for female individuals).

**Figure 3:**
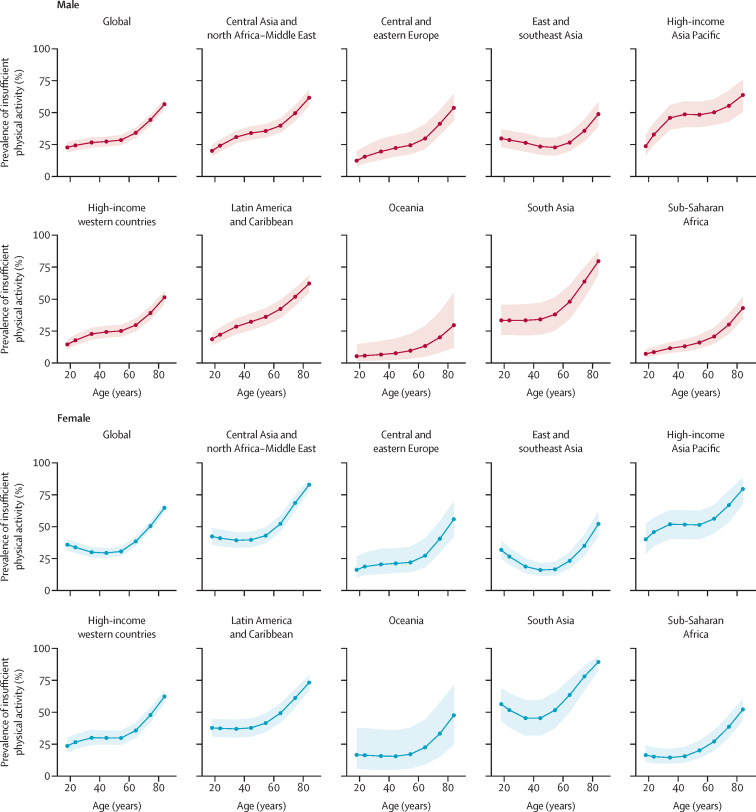
Age pattern of insufficient physical activity prevalence in 2022 by region and sex Shaded areas show 95% uncertainty intervals.

The prevalence of insufficient physical activity was highest in the oldest age groups in all regions (figure 3, appendix 3 p 39). For male individuals, the most common age pattern was a gentle increase in insufficient physical activity prevalence until approximately age 60 years, followed by a steeper increase. For female individuals, the most common pattern was J-shaped, with insufficient physical activity either stable or slightly decreasing up to approximately age 60 years. The region that differed most from these patterns was east and southeast Asia, where male individuals appeared to have stable—potentially even decreasing—insufficient physical activity levels up to age 60 years, and female individuals showed a clear decrease in insufficient physical activity up to this age.

**Figure 4:**
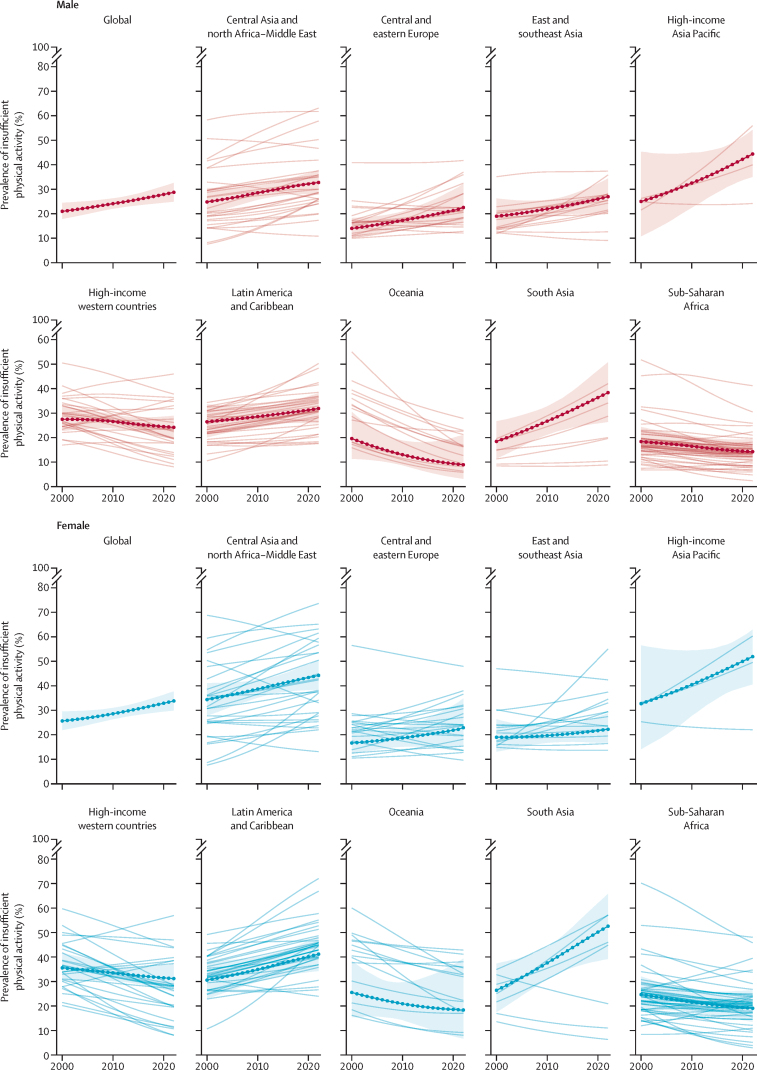
Trends in age-standardised insufficient physical activity prevalence 2000–22 for adults aged 18 years and older, by region and sex Country trends are shown as faint lines, regional average shown as a bold line with shaded uncertainty interval.

Our trend analysis shows the global age-standardised prevalence of insufficient physical activity has increased from 28·6% in 2010 to 31·3% in 2022 (table 1); the posterior probability of this being a true increase is more than 0·99. The steepest increases in insufficient physical activity were in the high-income Asia Pacific region (posterior probability 0·99) and south Asia region (posterior probability >0·99; figure 4). Conversely, decreasing trends were evident in high-income western countries (posterior probability 0·90), sub-Saharan Africa (posterior probability 0·94), and Oceania (posterior probability 0·83). In 103 (52%) of 197 countries, prevalence was increasing.

If 2010–22 trends continue, the global prevalence of insufficient physical activity in 2030 would be 34·7% (30·4–39·1%; table 2, appendix 3 pp 40–60), more than 8 percentage points higher than 2010. We have a high degree of certainty that the target of a 15% relative reduction between 2010 and 2030 would not be met if the current trajectory continues (posterior probability of meeting the target <0·01).

In the six regions with increasing trends in insufficient physical activity, we have high certainty that they will not meet the 2030 target if current trends continue (posterior probabilities of meeting the target ≤0·01). In the high-income western countries, there was a decreasing trend in insufficient physical activity, but the rate of decrease was just under what is necessary to meet the 2030 target. Although the region is classified as off track, there is lower certainty in this prediction (posterior probability 0·45). Sub-Saharan Africa and Oceania were classified as on track to meet the target with lower certainty (posterior probabilities 0·70–0·74).

At the country level, 136 countries are not on track to meet the 2030 target, whereas 61 countries are considered on track. Of these 61 countries, 22 countries are classified as on track with a higher certainty, of which 12 are European high-income western countries, four are in Oceania, and six are in sub-Saharan Africa (appendix 3 pp 40–60).

## Discussion

We estimate that nearly a third of adults globally (31·3%; 1·8 billion) were insufficiently physically active in 2022, an increase from 23·4% (900 million) in 2000. Approximately half of all countries and two-thirds of the regions had increasing trends in insufficient physical activity prevalence, the opposite trajectory to that needed to meet the 2030 global target of a 15% relative reduction in prevalence from a 2010 baseline. Female individuals were more likely to be insufficiently physically active than male individuals, a disparity that showed marked regional and national variability. Prevalence increased steeply among adults aged 60 years and older, but with geographical and sex differences in the age patterns for adults younger than 60 years.

This analysis included more surveys than the previous WHO estimates for 2016 (507 *vs* 358) and more countries' data-informed trends (108 *vs* 65).^10^ Because more data were included, we were able to revise modelling methods to estimate trends in insufficient physical activity in all 197 countries. We show a global increase in insufficient physical activity prevalence, whereas previous estimates had not detected a significant trend, probably due to divergent trajectories in the limited data previously available. Nevertheless, our global prevalence estimate for 2016 (28·7% [95% UI 26·9–30·4]) is similar to the previous analysis (27·5% [25·0–32·2]).^10^ Our model predictions might differ from national estimates due to adjustments for factors such as differences in questionnaires and age-standardisation. Our analysis also includes all domains; some countries highlight prevalence estimates based on leisure-time activity only.

Globally, progress toward the 2030 target is disappointing, with rising levels of insufficient physical activity. However, regional and country level analysis shows areas of optimism. As a region, high-income western countries are off track but close to the required rate of reduction. 12 of these countries, all in western Europe, are classified as on track with a high degree of certainty. Other analyses using different metrics of physical activity support this positive trend. Average weekly participation in non-sport and exercise physical activities rose across 27 EU member states between 2017 and 2022, from 44% to 53%.^20^ Similarly, the proportion achieving the recommendation through non-occupational activity rose from 29·9% in 2014 to 32·7% in 2019 across the same 27 countries.^21^ These positive trends could be linked to the physical activity promotion policies implemented in the region.^14^, ^22^ These policies tend to be multisectoral, as advocated for in the Global Action Plan for Physical Activity.^4^ Oceania, a region made up of 14 island nations, shows the greatest relative decreases in insufficient physical activity level. Eight of the 14 countries have more than two surveys separated in time, and three countries (Cook Islands, Samoa, and Solomon Islands) show steep decreasing trends. There have been increased policy efforts in the WHO Western Pacific region,^23^ potentially providing greater opportunities for physical activity and raising awareness. In Oceania and sub-Saharan Africa, the low baseline levels mean that an absolute decline of less than 3 percentage points is sufficient to meet the target; together with sparse data, this makes our predictions on progress towards the global target highly uncertain. As most countries globally are off track to meet the 2030 target, greater investment at the global and national level is needed to implement effective policies to reverse trends in physical activity.^22^ Countries already on track must continue their efforts to maintain the trajectory.

Epidemiological evidence shows the greatest health benefits can be gained by increasing activity among those with the lowest activity levels.^2^ Our results highlight important differences in insufficient activity levels by sex and age. Our findings support Mielke and colleagues'^24^ call for a greater focus on interventions that targeted women. We speculate that the regional variation in age patterns, most evident in those younger than 60 years, could be attributed to different cultural norms, average maternal age, and support for healthy ageing. In line with the 2030 Sustainable Development Goals' focus on leaving no one behind,^25^ and the Decade of Action on Health Aging,^26^ these results call for actions to increase female and older adult engagement in physical activity.

We were unable to detect differences in insufficient physical activity levels due to the COVID-19 pandemic and response. We included all data that fulfilled our inclusion criteria, but data collection slowed, especially during periods of stricter local responses. In general, in-person data collection ceased worldwide around the end of March 2020, with data collection resuming at different times according to national and local contexts. To our knowledge, none of our included data sources collected data from April to July, 2020. We identified and included only 30 surveys from 24 countries that collected any data from August, 2020 to December, 2021 (appendix 3 pp 16–31). We have modelled a linear trend in insufficient physical activity prevalence over time, but true prevalence during strict limitations on movement might have deviated from this. Some research suggests that lockdowns, on average, decreased activity levels;^27^ however, other evidence suggests differing individual trajectories.^28^ We conducted a sensitivity analysis to determine whether activity levels changed after March, 2020, when WHO announced COVID-19 as a pandemic, but found no evidence of a step change or non-linearity in trends in the included data collected during or after August 2020 (appendix 3 p 11). As the body of data collected post-pandemic increases, future updates will be better powered to detect any long-term effects of the pandemic on physical activity.

In their analysis, Guthold and colleagues^10^ stated that they expected a greater availability of device-based physical activity data in the next update. At the time of data compilation, we identified fewer than 15 countries with nationally representative data from devices, with considerable heterogeneity in protocols. WHO is addressing the need for standards and technical guidance in this area, which might lead to greater data availability for future updates.^29^ Measurement of compliance with the muscle strengthening, balance, and sedentary recommendations is another area where consensus is needed to encourage widespread data collection.

An important strength of this analysis is our extensive data search and rigorous inclusion of sources, including a systematic review, online search, consultation with WHO member states, and reanalysis of 390 individual-level datasets to ensure consistent data processing. Another key strength is our use of a Bayesian hierarchical model to use the totality of the data available to predict country trends in insufficient physical activity prevalence.

Our work is subject to various limitations. Most importantly, we do not have data from every country for every year, and we could not include any data from 34 countries. Countries with no, limited, or inconsistent data could differ systematically from countries with well developed surveillance systems. Self-report data are subject to recall and social desirability biases—there is potential for variation between countries or cultures and questions might be difficult to comprehend.^16^ Further, reported physical activity could vary by questionnaire. We have made a statistical adjustment for the most commonly used questionnaires to increase comparability; however, these adjustments are uncertain. These adjustments risk introducing a systematic bias dividing countries or time periods using specific questionnaires, particularly in countries and regions where the reference questionnaire, GPAQ, is used infrequently (eg, high-income western countries). Data quality and representativeness also might vary over time, even with consistent questionnaire usage due to advances in survey administration, sampling, and weighting. These advances are offset by declining survey response rates in many settings—the COVID-19 pandemic might have further depressed response rates or affected non-response bias.^30^ Our estimates disaggregate by age and sex, but not by other relevant characteristics such as wealth, geographical location, or disability.^25^ Finally, our uncertainty intervals do not reflect all sources of uncertainty (appendix 3 p 9).

In conclusion, nearly a third of adults globally do not meet the recommended levels of physical activity, and most countries are off track to meet the global target set for 2030. Given the evidence of persistent gaps in participation between sexes and across age groups, all countries are called upon to substantially increase implementation of policy and programmes to address these inequalities. Effective policies are known,^4^ but these data and recent progress reports^22^ show that globally implementation has been slow and uneven. Successful experiences from countries with a positive trend, and those on track to meet global target, should be shared widely. All counties are urged to align investments and strengthen multisector approaches to promote physical activity at both the national and local level, across both sexes, and across all age groups.

### Contributors

### Country Data Author Group

### Data sharing

Input data, statistical code, and estimates by country, age, and sex will be made available upon publication at https://www.github.com/MLGlobalHealth/PinA. Following data use agreements, individual-level data will not be made available. Country, regional, and global estimates will be made available upon publication on the WHO Global Health Observatory at https://www.who.int/data/gho.

## Declaration of interests

TS, SF, ES, and GAS report consulting contracts from WHO supporting this work. TS declares expenses paid to speak at an event organised by Biogredia AB in January, 2023.

## Supplementary Materials

Spanish translation of the abstract

Portuguese translation of the abstract
