# Correction to *Lancet Glob Health* 2024; 12: e1232–43

**DOI:** 10.1016/S2214-109X(24)00533-3

**Published:** 2024-12-06

*Strain T, Flaxman S, Guthold R, et al. National, regional, and global trends in insufficient physical activity among adults from 2000 to 2022: a pooled analysis of 507 population-based surveys with 5·7 million participants.* Lancet Glob Health *2024;* 12: *e1232–43*—For this Article, the Country Data Author Group was missing. This correction has been made as of Dec 6, 2024.

