# Supplementary material for: National, regional, and global trends in insufficient physical activity among adults from 2000 to 2022: a pooled analysis of 507 population-based surveys with 5·7 million participants
Source: Lancet Glob Health. 2024 Jun 25;12(8):e1232–43. doi: 10.1016/S2214-109X(24)00150-5 (PMC11254784; doi:10.1016/S2214-109X(24)00150-5)
Supplement: Spanish translation of the abstract [file mmc1.pdf]

THE LANCET
Global Health

Supplementary appendix 1

This translation in Spanish was submitted by the authors and we reproduce it as supplied. It has not been peer reviewed. *The Lancet*'s editorial processes have only been applied to the original in English, which should serve as reference for this manuscript.

Los autores nos proporcionaron esta traducción al español y la reproducimos tal como nos fue entregada. No la hemos revisado. Los procesos editoriales de *The Lancet* se han aplicado únicamente al original en inglés, que debe servir de referencia para este manuscrito.

Supplement to: Strain T, Flaxman S, Guthold R, et al. National, regional, and global trends in insufficient physical activity among adults from 2000 to 2022: a pooled analysis of 507 population-based surveys with 5·7 million participants. *Lancet Glob Health* 2024; published online June 25. https://doi.org/10.1016/S2214-109X(24)00150-5.

Resumen

Antecedentes

La actividad física insuficiente aumenta el riesgo de desarrollar enfermedades crónicas no transmisibles, baja función cognitiva y física, ganancia de peso y problemas de salud mental. La prevalencia global de actividad física insuficiente fue publicada por última vez en 2016, utilizando datos limitados de tendencias. El objetivo de este estudio fue estimar la prevalencia de actividad física insuficiente para 197 países y territorios, de 2000 a 2022.

Métodos

Recopilamos la actividad física reportada por adultos (≥18 años) de encuestas poblacionales. La actividad física insuficiente se definió como no realizar 150 minutos de actividad de intensidad moderada, o 75 minutos de actividad de intensidad vigorosa, o una combinación equivalente por semana. Utilizamos un modelo jerárquico Bayesiano para estimar la prevalencia de actividad física insuficiente por país o territorio, año, edad y sexo. Evaluamos si los países o territorios, regiones y el mundo cumplirían con el objetivo global de una reducción relativa de 15% en la prevalencia de actividad física insuficiente en 2030, si continuaran las tendencias observadas de 2010 a 2022.

Resultados

Incluimos 507 encuestas de 163 países/territorios. La prevalencia global ajustada por edad de actividad física insuficiente fue de 31.3% (Intervalo de incertidumbre al 95% 28.6-34.0%) en 2022, que representa un aumento en comparación con el 23.4% (21.1-26.0%) en 2000 y 26.4% (24.8-27.9%) en 2010. La prevalencia ajustada por edad tendió a aumentar en 103 de los 197 países y territorios, y 6 (57%) de las 9 regiones, y tendió a disminuir en el resto. La prevalencia fue 5 puntos porcentuales mayor en mujeres (33.8% (29.9-37.7%)) que en hombres (28.7% (25.0-32.6%)). La actividad física insuficiente aumentó en los mayores de 60 años de todas las regiones y para ambos sexos, mientras que los patrones por edad no fueron consistentes en los menores de 60 años. Si las tendencias de 2010-2022 continúan, el objetivo global de una reducción relativa de 15% entre 2010 y 2030 no se cumplirá (probabilidad posterior <0.01);

sin embargo, dos regiones, Oceanía y África Sub-Sahariana, están en camino a lograrlo con una considerable incertidumbre (probabilidades posteriores 0.70-0.74)

Interpretación

Se requieren esfuerzos coordinados y multisectoriales para reducir los niveles de actividad física insuficiente y alcanzar la meta del 2030. La promoción de actividad física no debe exacerbar las inequidades de sexo, edad o geográficas.

Financiamiento

Ministerio de Salud Pública, Catar; Organización Mundial de la Salud.
