# Supplementary material for: National, regional, and global trends in insufficient physical activity among adults from 2000 to 2022: a pooled analysis of 507 population-based surveys with 5·7 million participants
Source: Lancet Glob Health. 2024 Jun 25;12(8):e1232–43. doi: 10.1016/S2214-109X(24)00150-5 (PMC11254784; doi:10.1016/S2214-109X(24)00150-5)
Supplement: Portuguese translation of the abstract [file mmc2.pdf]

THE LANCET
Global Health

Supplementary appendix 2

This translation in Portuguese was submitted by the authors and we reproduce it as supplied. It has not been peer reviewed. *The Lancet*'s editorial processes have only been applied to the original in English, which should serve as reference for this manuscript.

Esta tradução em português foi submetida pelos autores e nós não fizemos quaisquer alterações. Esta versão não foi revista por pares. O processo editorial do The Lancet só foi aplicado à versão original em inglês, que deve servir como referência para este artigo.

Supplement to: Strain T, Flaxman S, Guthold R, et al. National, regional, and global trends in insufficient physical activity among adults from 2000 to 2022: a pooled analysis of 507 population-based surveys with 5·7 million participants. *Lancet Glob Health* 2024; published online June 25. https://doi.org/10.1016/S2214-109X(24)00150-5.

Resumo

Introdução

A atividade física insuficiente aumenta o risco para doenças crônicas não transmissíveis, reduzida funcionalidade física e cognitiva, ganho de peso e problemas relacionados a saúde mental. A prevalência global de adultos com atividade física insuficiente mais recente foi publicada em 2016, com dados limitados sobre tendencias temporais. Dessa forma, o objetivo do presente estudo foi estimar a prevalência de atividade física insuficiente em 197 países ou territórios, de 2000 a 2022.

Métodos

Dados de atividade física relatados por adultos (≥18 anos) em pesquisas de base populacional foram compilados. Atividade física insuficiente foi definida como não praticar 150 minutos de atividade física de intensidade moderada, ou 75 minutos de intensidade vigorosa, ou uma combinação equivalente por semana. O modelo hierárquico Bayesiano foi utilizado para computar as estimativas de atividade física insuficiente por pais ou território, ano, idade e sexo. Assim, foi avaliado se os países ou territórios, as regiões e o nível global atingiriam a meta global de uma redução relativa de 15% na prevalência de atividade física insuficiente até 2030, caso as tendências de 2010 a 2022 continuassem.

Achados

Foram incluídas 507 pesquisas em 163 países/territórios. A prevalência global, padronizada por idade, de atividade física insuficiente foi de 31,3% (intervalo de incerteza de 95%: 28,6-34,0%) em 2022, um aumento comparado a 23,4% (21,1-26,0%) em 2000 e 26,4% (24,8-27,9%) em 2010. A prevalência aumentou em 103 (52%) de 197 países e territórios, bem como seis (57%) das nove regiões, enquanto os demais reduziram. A prevalência foi cinco pontos percentuais superior em mulheres (33,8% (29,9-37,7%)) que homens (28,7% (25,0-32,6%)). A prática insuficiente de atividade física aumentou em pessoas com 60 anos ou mais, em todas as regiões e ambos os sexos, mas os padrões etários diferiram para aqueles com menos de 60 anos. Se as tendências entre 2010 e 2022 continuarem, a meta global de uma redução relativa de 15% entre 2010 e 2030 não será alcançada (probabilidade *a posteriori* < 0.01). No entanto, duas regiões (Oceania e África Subsaariana)

estiveram dentro da expectativa de cumprimento da meta, com considerável incerteza (probabilidades *a posteriori* de 0,70 a 0,74).

Interpretação

Esforços coordenados e multisetoriais são necessários para reduzir os níveis de atividade física insuficiente e atingir a meta de 2030. A promoção da atividade física não deve agravar as desigualdades relacionadas ao sexo, idade ou localização geográfica.

Financiamento

Ministério da Saúde Pública, Catar; Organização Mundial da Saúde.
