# Supplementary material for: National, regional, and global trends in insufficient physical activity among adults from 2000 to 2022: a pooled analysis of 507 population-based surveys with 5·7 million participants
Source: Lancet Glob Health. 2024 Jun 25;12(8):e1232–43. doi: 10.1016/S2214-109X(24)00150-5 (PMC11254784; doi:10.1016/S2214-109X(24)00150-5)
Supplement: Supplementary appendix 3 [file mmc3.pdf]

THE LANCET
Global Health

Supplementary appendix 3

This appendix formed part of the original submission and has been peer reviewed. We post it as supplied by the authors.

Supplement to: Strain T, Flaxman S, Guthold R, et al. National, regional, and global trends in insufficient physical activity among adults from 2000 to 2022: a pooled analysis of 507 population-based surveys with 5·7 million participants. *Lancet Glob Health* 2024; published online June 25. https://doi.org/10.1016/S2214-109X(24)00150-5.

Table of Contents

Appendix 1. Countries and territories by analysis region 2

Appendix 2. Location of GATHER reporting items. 3

Appendix 3. Data inclusion, identification and access 4

3.1 Data inclusion criteria 4

3.2 Data identification 5

3.2.1 Systematic review 5

3.3 Data access and processing 6

3.4 Accounting for complex survey design 7

3.5 Search for previous global comparable estimates of activity 7

Appendix 4. Methods for adjusting the prevalence of insufficient physical activity for urbanicity and definition 8

Appendix 5. Bayesian hierarchical model 9

5.1 Covariate selection 9

5.2 Model specification 10

5.3 Sensitivity analyses 11

5.3.1 Insufficient physical activity prevalence and COVID-19 11

5.3.2 Linear *vs.* flexible trends over time 11

5.3.3 Inclusion of indicator variables for questionnaire type 11

Appendix 6. Methods for assessing trends in insufficient physical activity and progress toward the 2030 physical activity target 13

Appendix 7. Flowchart of identification, inclusion and exclusion of data sources 14

Appendix 8. Map of number of surveys included by country 15

Appendix 9. Characteristics of included data sources 16

Appendix 10. Country prevalence of insufficient physical activity (95% uncertainty interval) in 2022 32

Appendix 11. Map of prevalence of insufficient physical activity in 2022, by country and sex 37

Appendix 12. Scatter plot of male and female age-standardised prevalence of insufficient physical activity in 2022, by income group 38

Appendix 13. Prevalence of insufficient physical activity in 2022, by age group 39

Appendix 14. Age-standardised prevalence of insufficient physical activity among adults aged 18 years and over in 2000, 2010 and 2022, projected prevalence in 2030 assuming trends 2010-2022 continue, and progress during 2010-2022 toward the global target of reducing the prevalence of insufficient physical activity by 15% between 2010 and 2030, by country for both sexes 40

Appendix 15. Age-standardised prevalence of insufficient physical activity among adults aged 18 years and over in 2000, 2010 and 2022, projected prevalence in 2030 assuming trends 2010-2022 continue, and progress during 2010-2022 toward the global target of reducing the prevalence of insufficient physical activity by 15% between 2010 and 2030, by country for men. 47

Appendix 16. Age-standardised prevalence of insufficient physical activity among adults aged 18 years and over in 2000, 2010 and 2022, projected prevalence in 2030 assuming trends 2010-2022 continue, and progress during 2010-2022 toward the global target of reducing the prevalence of insufficient physical activity by 15% between 2010 and 2030, by country for women. 54

Appendix 17. Country progress during 2010-2022 toward the global target of a 15% relative reduction in insufficient physical activity prevalence among adults aged 18 years and over between 2010 and 2030, by sex 61

Appendix 18. Model input data and estimated levels of insufficient physical activity, by country, sex, and age. 62

References 260

Appendix 1. Countries and territories by analysis region

Notes: Countries and territories are grouped into epidemiologically similar sets of countries based on geography and development level, and are referred to as regions. Estimates were made for all countries and territories, however, eligible survey data were not necessarily identified for all countries and territories.

**Region**	**Countries and territories**
Central Asia and North Africa-Middle East	Algeria, Armenia, Azerbaijan, Bahrain, Egypt, Georgia, Iran (Islamic Republic of), Iraq, Jordan, Kazakhstan, Kuwait, Kyrgyzstan, Lebanon, Libya, Mongolia, Morocco, Oman, Qatar, Saudi Arabia, Syrian Arab Republic, Tajikistan, Tunisia, Turkmenistan, Türkiye, United Arab Emirates, Uzbekistan, Yemen, occupied Palestinian territory including east Jerusalem
Central and Eastern Europe	Albania, Belarus, Bosnia and Herzegovina, Bulgaria, Croatia, Czechia, Estonia, Hungary, Latvia, Lithuania, Montenegro, Poland, Republic of Moldova, Republic of North Macedonia, Romania, Russian Federation, Serbia, Slovakia, Slovenia, Ukraine
East and South-East Asia	Brunei Darussalam, Cambodia, China, Democratic People's Republic of Korea, Indonesia, Lao People's Democratic Republic, Malaysia, Maldives, Myanmar, Philippines, Taiwan (China), Thailand, Timor-Leste, Viet Nam
High-income Asia Pacific	Japan, Republic of Korea, Singapore
High-income Western countries	Andorra, Australia, Austria, Belgium, Canada, Cyprus, Denmark, Finland, France, Germany, Greece, Iceland, Ireland, Israel, Italy, Luxembourg, Malta, Monaco, Netherlands, New Zealand, Norway, Portugal, San Marino, Spain, Sweden, Switzerland, United Kingdom, United States of America
Latin America and Caribbean	Antigua and Barbuda, Argentina, Bahamas, Barbados, Belize, Bolivia (Plurinational State of), Brazil, Chile, Colombia, Costa Rica, Cuba, Dominica, Dominican Republic, Ecuador, El Salvador, Grenada, Guatemala, Guyana, Haiti, Honduras, Jamaica, Mexico, Nicaragua, Panama, Paraguay, Peru, Puerto Rico, Saint Kitts and Nevis, Saint Lucia, Saint Vincent and the Grenadines, Suriname, Trinidad and Tobago, Uruguay, Venezuela (Bolivarian Republic of)
Oceania	Cook Islands, Fiji, Kiribati, Marshall Islands, Micronesia (Federated States of), Nauru, Niue, Palau, Papua New Guinea, Samoa, Solomon Islands, Tonga, Tuvalu, Vanuatu
South Asia	Afghanistan, Bangladesh, Bhutan, India, Nepal, Pakistan, Sri Lanka
Sub-Saharan Africa	Angola, Benin, Botswana, Burkina Faso, Burundi, Cabo Verde, Cameroon, Central African Republic, Chad, Comoros, Congo, Côte d'Ivoire, Democratic Republic of the Congo, Djibouti, Equatorial Guinea, Eritrea, Eswatini, Ethiopia, Gabon, Gambia, Ghana, Guinea, Guinea-Bissau, Kenya, Lesotho, Liberia, Madagascar, Malawi, Mali, Mauritania, Mauritius, Mozambique, Namibia, Niger, Nigeria, Rwanda, Sao Tome and Principe, Senegal, Seychelles, Sierra Leone, Somalia, South Africa, South Sudan, Sudan, Togo, Uganda, United Republic of Tanzania, Zambia, Zimbabwe

Appendix 2. Location of GATHER reporting items.

Item #	Checklist item	Location reported
**Objectives and funding**		
**1**	Define the indicator(s), populations (including age, sex, and geographic entities), and time period(s) for which estimates were made.	Paragraph 1 of methods
**2**	List the funding sources for the work.	Abstract
**Data Inputs**		
For all data inputs from multiple sources that are synthesized as part of the study:		
**3**	Describe how the data were identified and how the data were accessed.	Appendix 3
**4**	Specify the inclusion and exclusion criteria. Identify all ad-hoc exclusions.	Appendix 3
**5**	Provide information on all included data sources and their main characteristics. For each data source used, report reference information or contact name/institution, population represented, data collection method, year(s) of data collection, sex and age range, diagnostic criteria or measurement method, and sample size, as relevant.	Appendix 9, www.github.com/MLGlobalHealth/PinA
**6**	Identify and describe any categories of input data that have potentially important biases (e.g., based on characteristics listed in item 5).	Methods, Appendices 3-5
For data inputs that contribute to the analysis but were not synthesized as part of the study:		
**7**	Describe and give sources for any other data inputs.	Appendix 5
For all data inputs:		
**8**	Provide all data inputs in a file format from which data can be efficiently extracted (e.g., a spreadsheet rather than a PDF), including all relevant meta-data listed in item 5. For any data inputs that cannot be shared because of ethical or legal reasons, such as third-party ownership, provide a contact name or the name of the institution that retains the right to the data.	www.github.com/MLGlobalHealth/PinA
**Data analysis**		
**9**	Provide a conceptual overview of the data analysis method. A diagram may be helpful.	Methods
**10**	Provide a detailed description of all steps of the analysis, including mathematical formulae. This description should cover, as relevant, data cleaning, data pre-processing, data adjustments and weighting of data sources, and mathematical or statistical model(s).	Appendices 4-6
**11**	Describe how candidate models were evaluated and how the final model(s) were selected.	Appendix 5
**12**	Provide the results of an evaluation of model performance, if done, as well as the results of any relevant sensitivity analysis.	Appendix 5
**13**	Describe methods for calculating uncertainty of the estimates. State which sources of uncertainty were, and were not, accounted for in the uncertainty analysis.	Appendix 5
**14**	State how analytic or statistical source code used to generate estimates can be accessed.	www.github.com/MLGlobalHealth/PinA
**Results and Discussion**		
**15**	Provide published estimates in a file format from which data can be efficiently extracted.	www.github.com/MLGlobalHealth/PinA https://www.who.int/data/gho/data/themes/topics/noncommunicable-diseases-risk-factors
**16**	Report a quantitative measure of the uncertainty of the estimates (e.g. uncertainty intervals).	Table 2
**17**	Interpret results in light of existing evidence. If updating a previous set of estimates, describe the reasons for changes in estimates.	Paragraph 2 of discussion
**18**	Discuss limitations of the estimates. Include a discussion of any modelling assumptions or data limitations that affect interpretation of the estimates.	Paragraph 8 of discussion

Appendix 3. Data inclusion, identification and access

Our data inclusion, search and access strategy was designed to obtain as many sources as possible while ensuring that the sources collected comparable (self-reported, time-based) information on physical activity across all domains and were representative of the population at the national level or at a diverse subnational area.

3.1 Data inclusion criteria

We included data sources if:

- the prevalence of insufficient physical activity was reported according to the current WHO recommendations or the International Physical Activity Questionnaire (IPAQ) scoring categories, or one of these could be computed from the individual-level data. These categories are defined as follows:
    - Not meeting the current WHO physical activity recommendations for aerobic activity: at least 150 minutes/week of moderate intensity or 75 minutes/week of vigorous activity, or an equivalent combination of the two;
    - Not meeting the 'high' or 'moderate' level of activity criteria from the IPAQ scoring protocol:
        - At least 30 minutes of moderate-intensity activity or walking per day on at least 5 days per week; or
        - At least 20 minutes of vigorous-intensity activity per day on at least 3 days per week; or
        - Engaging in 5 or more days of any combination of walking, moderate- or vigorous-intensity activities achieving a minimum of at least 600 MET minutes per week;
- the survey questions assessed total weekly duration of moderate and vigorous physical activity across all domains of life, including work/household, transport and leisure time;
- prevalence was based on self-report, not device-based measurement of physical activity;
- data were collected from the general adult (aged 18 years and over) population through probabilistic sampling using a defined sampling frame, and were representative of a national or defined subnational population covering at least three regions within a country or a UK home nation or ≥75% of a country's population;
- statistics by sex and by defined age groups[1] were reported or could be calculated from individual record data;
- data were collected in or after 2000;
- data were from a WHO member state; Puerto Rico; Taiwan, China; or occupied Palestinian territory, including east Jerusalem; and
- total survey sample size was at least n=200.[2]

We excluded the following data sources:

- sources that excluded migrants when collected in countries where migrants comprised more than 40% of the population (Kuwait, Qatar, United Arab Emirates, and Singapore), or
- longitudinal studies where there was >60% loss to follow-up, or
- data obtained at the individual level with the following indicators of poor quality:
    - >20% of the sample missing data on age or sex;
    - >20% of the sample missing physical activity data.

Finally, we made three ad-hoc exclusions of data we considered to be implausible (unlikely prevalence values and/or strong age trends in the opposing direction to expected): Colombia ENSIN 2005; Estonia National Physical Activity Survey 2015; and Paraguay STEPS 2011.

Our second inclusion criteria, which required weekly duration of activity across all domains of life, resulted in excluding some national surveys which are commonly used for physical activity surveillance. For example, the European Health Interview Survey (EHIS), which has been conducted in all EU member states, does not collect <u>duration</u> of physical activity in the work/household domain, and therefore all EHIS surveys have been excluded. In Colombia, the ENSIN 2010, ELANS 2014/2015 and ENSIN 2015 administered a modified version of IPAQ long that excluded the work/household domain, and therefore we excluded all three surveys.

---

[1] Summary statistics covering age groups spanning more than 30 years of age and those covering open-ended age groups starting below 70 years of age were excluded.

[2] In addition, age-sex observations with a sample size below 10 were excluded.

3.2 Data identification

Data sources were identified through the following sources:

- data from Guthold et al. (2018) for inclusion (1);
- subsequent waves of data collection from surveys included in Guthold et al. (2018) (1);
- data shared with the WHO Department of Noncommunicable Diseases, such as those stemming from the WHO STEPwise approach to surveillance (2);
- surveys named in the 2021 WHO Country Capacity Survey in response to a query about the latest nationally representative physical activity data source (3);
- a targeted online search for all countries, using country name, terms of physical activity and national survey (translated as appropriate)[3] checking the first page of hits;
- a systematic review of the literature targeted at the 35 most populous countries (details below); and
- surveys identified by academic contacts or by focal points nominated by WHO Member States during a formal country consultation.

The initial searches were undertaken between November 2022 and March 2023, with the retrieval of identified data sources continuing until June 2023. In August 2023, preliminary estimates and data sources identified for each country were shared with nominated focal points in each country (typically in the Ministry of Health) as part of a WHO country consultation with a response deadline of October 6, 2023. Nominated focal points reviewed estimates and suggested additional data sources, which were included if they met our inclusion criteria. Our database was closed in February, 2024.

3.2.1 Systematic review
Our previous update of insufficient physical activity included a systematic review of the literature for data on adults 18 and older that extended through October 2015, as described in Guthold et al 2018 (1). Based on the experience of Guthold et al, the systematic review was expected to identify a maximum of 10% of the final data sources. Therefore, the size of the search will be proportionate rather than extensive and it was restricted to data sources published from October 2015 to February 2023.

The systematic review targeted the 35 most populous countries making up 80% of the global population (4): China, India, Indonesia, Pakistan, Brazil, Nigeria, Bangladesh, Russian Federation, Japan, Ethiopia, Philippines, Egypt, Viet Nam, Democratic Republic of Congo, Turkey, United Republic of Tanzania, Italy, South Africa, Myanmar, Kenya, Colombia, Argentina, Sudan, Uganda, Ukraine, and Algeria.

PubMed was searched on the 2nd February 2023 using terms that included the country names in English and alternative spellings, terms for physical activity, population surveillance, and specific physical activity questionnaires (see below). One reviewer (TS) screened 1254 title and abstracts and excluded 1093 based on the criteria listed in above. The same reviewer screened 161 full texts, 109 of which were excluded based on the criteria, 46 were excluded as the survey had already been identified. We obtained data from two of the five surveys newly identified; all others were subnational.

The search terms were:

("physical activity" OR "physical inactivity" OR "exercise" OR "exercise" OR “motor activity”) AND

("Physical Activity Questionnaire" OR "International Physical Activity Questionnaire" OR "IPAQ" OR "Global Physical Activity Questionnaire" OR "GPAQ") AND

("population surveillance" OR "population surveillance" OR population OR "Sentinel Surveillance" OR "comparative study" OR "comparative study" OR "incidence" OR "incidence" OR "prevalence" OR "prevalence" OR "Health Services Research" OR "statistics and numerical data" OR statistic* OR "epidemiology" OR epidemiology OR epidemiol* OR "Data Collection") AND

(China OR “PRC” OR India OR Indonesia OR Pakistan OR Brazil OR Brasil OR Nigeria OR Bangladesh OR “Russian Federation” OR Russia OR Japan OR Ethiopia OR Philippines OR “Pilipinas” OR Egypt OR “Viet Nam” OR “Vietnam” OR “Democratic Republic of Congo” OR “DRC” OR “République démocratique du Congo” OR “RDC” OR “DR Congo” OR Turkey OR “Türkiye Cumhuriyeti” OR “Turkiye” OR “Türkiye” OR “United Republic of Tanzania” OR Tanzania OR Italy OR Italie OR Italia OR South Africa OR Myanmar OR

[3] E.g. for Australia, search terms were “Australia” AND “physical activity” AND “national survey”.

Kenya OR Colombia OR Argentina OR "Argentine" OR Sudan OR Uganda OR Ukraine OR Algeria OR Algerie OR Algérie)

3.3 Data access and processing

Once a survey was identified for inclusion, we obtained either summary statistics or individual-level data. Our decision process for data access method is summarized in the following figure:

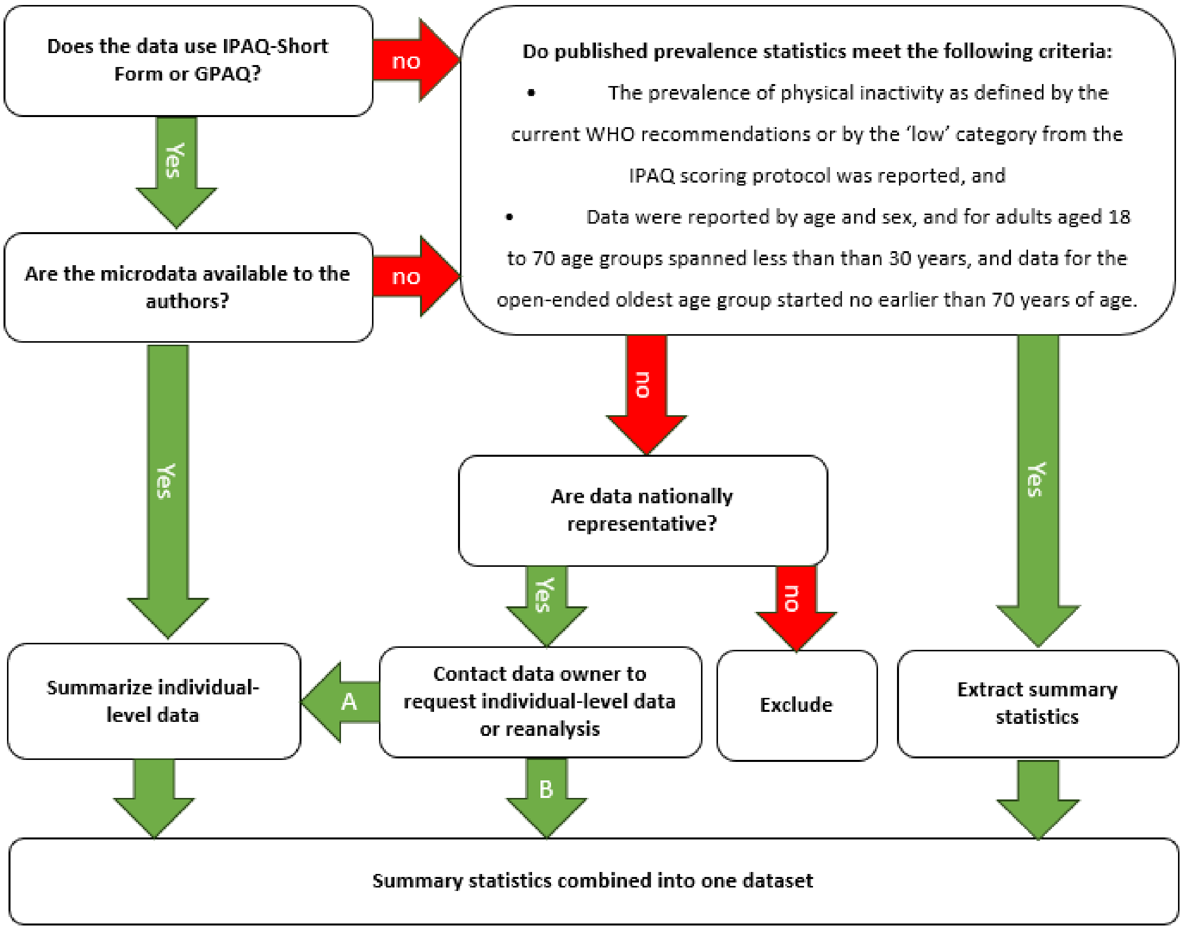

Notes: A. Data owner shared individual-level data. B. Data owner shared summary statistics.

For each data source accessed as individual-level data, we extracted the following individual-level variables: age, sex,[4] responses to all questions on physical activity, and survey sampling variables if available (survey sample weight, stratum, and primary sampling unit). We also recorded the questionnaire used, the survey administration method (i.e., face-to-face or telephone), the geographic area represented (country and region, if applicable), the years and months of survey fieldwork, and how we obtained the data. We processed physical activity questionnaire data using standard protocols according to the questionnaire used. Data collected using the Global Physical Activity Questionnaire (GPAQ) were processed according to the WHO protocol, which assigns 8 METs for vigorous intensity activity and 4 METs for moderate intensity activity (which includes all active travel) (5). Data collected using the IPAQ short form (6) were processed using an adapted protocol harmonized to the GPAQ protocol. This involved adopting the same principles relating to missing data as the GPAQ protocol, and assigning the same MET values. Other questionnaires were processed according to survey specific protocols. We then computed the prevalence of insufficient physical activity (<600 MET minutes per week) for standard age groups (18-29, 30-39, 40-49, 50-59, 60-69, 70-79, ≥80 years) by sex, taking into account complex survey design as described below. We excluded surveys that did not meet our additional criteria for individual-level data (Appendix 3.1): age or sex must be reported for more than 80% of observations, and physical activity data must be reported for more than 80% of observations.

[4] We have chosen to use the term sex when referring to survey data and estimates rather than gender as this is how the data were collected in the majority of surveys.

For each data source accessed as summary statistics, we checked processing protocols wherever possible for compatibility with our own for individual-level data. We extracted the following information: the questionnaire used, the survey administration method (i.e., face-to-face or telephone), the geographic area represented, the years and months of survey fieldwork, the definition of insufficient physical activity, and the source of data. For each age-sex observation, we extracted the prevalence of insufficient physical activity, sex, age range, and sample size. We excluded age-sex observations (but not the whole survey) with age range wider than 30 years, open-ended age groups starting before age 70, or observations with sample size < 10.

Finally, we combined data accessed as summary statistics and data accessed at the individual level into a single database. We manually identified and removed duplicated data accessed from more than one source.

3.4 Accounting for complex survey design

Most of the individual-level data in our analysis came from surveys that used complex survey designs. Specifically, in designing a representative survey, the target populations were usually divided into strata based on geographical regions within the country and possibly other factors such as whether place of residence was rural or urban; within each stratum, a number of clusters were randomly selected. Clusters may be villages, administrative units, or census units. Households or participants were then randomly sampled within each cluster. Because the total population may differ among strata and clusters, individuals or households in smaller units have a higher probability of being selected than those in larger units. To account for the differences in probability of being sampled, each observation is assigned a sample weight. Sample weights may also adjust for differential non-response, i.e., if response rates are higher among some demographic groups or geographic areas. These weights are calculated to make the survey data representative of the total population. If they were available, we took sample weights into account when computing the prevalence of insufficient physical activity. In some cases, we accessed data from a nationally representative survey using a complex survey design, but for which sample weights were not available. In these cases, we treated the survey as subnationally, rather than nationally representative.

An implication of the sampling method is that the so-called effective sample size of the survey (ESS) is different from its actual sample size. This occurs primarily because the sampled individuals are from clusters that are representative but do not cover the entire country, and hence contain less information than they would, had they been a true random sample of the population.

To reflect the true availability of information in each survey and in the individual level data that it provided to the statistical model, we estimated ESS using the Stata version 17.0 svy suite of commands (StataCorp, 2021).

Data sources providing only summary statistics were also predominantly from surveys that used complex survey designs, but sample sizes recorded for these data sources are actual sample sizes and not the effective sample sizes. In addition, we received some individual-level data from complex surveys, but the data did not include design variables. To ensure that the sample sizes used for these sources in the statistical modelling also reflect the complex survey design, we estimated ESS for each study as the actual sample size divided by an estimate of the design effect (DEFF). We used the median DEFF from all surveys with individual-level data to estimate ESS for these data sources.

3.5 Search for previous global comparable estimates of activity

We conducted a supplementary search for the Research in Context. We aimed to identify previously published comparable estimates of (insufficient) physical activity amongst adults for countries worldwide. We searched PubMed using the search terms listed below with no language restriction covering literature from 1 January 2000 to 24 October 2023. We cited the relevant papers in the introduction.

(("global insufficient physical activity"[Title/Abstract:~1]) OR ("global physical activity"[Title/Abstract:~1]) OR ("global physical inactivity"[Title/Abstract:~1]))

AND (((estimate*[Title/Abstract]) OR (level*[Title/Abstract])) OR (prevalence[Title/Abstract]))

AND "adult"

NOT "global physical activity questionnaire"

Appendix 4. Methods for adjusting the prevalence of insufficient physical activity for urbanicity and definition

Some data sources reported the prevalence of insufficient physical activity according the International Physical Activity Questionnaire (IPAQ) 'low' category (6,7). Further, some data sources covered only urban areas. We used regression equations developed by Guthold et al (1) to adjust these data sources to ensure that the data are comparable to data reporting the prevalence of insufficient physical activity using current recommendations and data that are representative of both urban and rural areas. We accounted for uncertainty of these steps. First, we calculated the regression prediction variance for each summary statistic using the "predict" command in Stata version 17.0 (StataCorp, 2021). We used this prediction variance to adjust the ESS for each adjusted observation using the following formula:

$$ESS_{adj} = \frac{p(1-p)}{\frac{p(1-p)}{ESS_{survey}} + var(p)}$$

where p is the adjusted prevalence of insufficient physical activity, ESS_{survey} is the effective sample size for the survey observation, and *var(p)* is the regression prediction variance.

Appendix 5. Bayesian hierarchical model

Our aim was to estimate the prevalence of insufficient physical activity for every country, year, age group (18 years of age and older), and for males and females. All analyses were done separately for males and females.

We used a Bayesian hierarchical model, which uses all available data to make estimates for each country-year-age unit. In the hierarchical model, estimates for each country-year-age were informed by data from that unit itself, if available, and by data from other ages and years in the same country and in other countries, especially those in the same region with data in similar time periods. The hierarchical model shares information to a greater degree where data are non-existent or weakly informative (i.e., have large uncertainty), and a lesser degree in data-rich countries and regions. Countries and territories were organized *a priori* into nine epidemiologically similar sets of countries, referred to as regions (Appendix 1).

Age patterns were modelled using a flexible age pattern that varied by region. Specifically, we fit natural splines with knots at ages 30 and 60 for each region. Natural splines enforce the constraint that the function is linear beyond the boundary knots, which provides for stable estimates at the youngest and oldest ages. The choice of knots was made based on two factors: (1) expectation of a smooth evolution of insufficient physical activity with age and (2) numerical stability of the resulting model. We considered automatic knot choices in which we defined the degrees of freedom (df=3) but this led to numerical instability in estimates at the youngest ages. The final choice of knots was the best balance between epidemiology and a stable model. While assessing model fit, we found that data from China have a unique age pattern of insufficient physical activity, with a U-shape in both sexes. While the same U shape was observed in other countries for women, it was not observed elsewhere for men. Therefore, we separated China into its own region for the purpose of modelling age patterns.

We modelled trends over time as a linear trend at the country and regional levels. We assessed this assumption via cross-validation (see Appendix 5.3.2 below). The estimates are also informed by a time-varying country covariate that helps predict insufficient physical activity levels: age-standardized prevalence of obesity (8) (details on covariate selection in Appendix 5.1). The model included a variance term that accounted for unobserved design factors (sample design, season, etc.) that lead to additional variability in the data beyond that expected due to sample size. Finally, the model accounted for the fact that subnational data may have larger variation than national data.

Self-report of physical activity may vary by questionnaire (9,10). We included indicator variables for three categories of questionnaire, with the GPAQ questionnaire as the reference standard: IPAQ-short form, the Eurobarometer instrument used for the 2013, 2017 and 2022 rounds, and other questionnaires (comprising a variety of national instruments). The final fitted fixed effects were as follows:

**Questionnaire**	**Male model**	**Female model**
IPAQ-short form	0.260 (0.169, 0.348)	0.316 (0.220, 0.415)
Eurobarometer 2013-2022	-0.148 (-0.264, -0.036)	-0.163 (-0.288, -0.039)
Other questionnaires	-0.096 (-0.260, 0.067)	-0.138 (-0.317, 0.044)

These indicator variables were set to zero when predicting prevalence of insufficient physical activity, thereby adjusting for systematic under- or over-reporting relative to GPAQ when using other questionnaires. This served the same purpose as the regression adjustment that was carried out by Guthold et al for surveys administering the IPAQ-short form questionnaire (1).

The uncertainties of our estimates incorporated sampling error in each data source; non-sampling error of national data, *e.g.*, because of issues in sample design and measurement; additional error associated with subnational data; uncertainty due to adjustments for definition or urbanicity; and uncertainty due to making estimates by country and year when data were missing altogether. However, they do not reflect any measurement or selection bias that is common across data sources, nor do they reflect model misspecification bias.

5.1 Covariate selection

We selected candidate time-varying covariates using the following criteria: first, the covariate should be related to the levels of physical activity (either influencing insufficient physical activity levels, *i.e.*, walkable cities, or outcomes that are influenced by insufficient physical activity levels). Second, because the goal of including country covariates is to improve model estimates when primary data are sparse, inconsistent or missing altogether, country covariates should be based on ample, reliable primary data.

We accessed and reviewed potential covariate data against the criteria above. We selected two covariates for testing within our model: percentage of population living in urban areas (11) and mean BMI (12). We tested four covariate sets. Set 0 excluded time-varying covariates. Sets 1-3 included all combinations of the preliminary covariates: (1) mean BMI, (2) percentage living in an urban area, and (3) both covariates. We carried out 10-fold cross-validation for each covariate set, in which data from 10% of countries are withheld in each iteration and model predictions are compared to withheld data. We computed mean absolute error (MAE), mean squared error (MSE) and root mean square error (RMSE). We also compared Bayesian information criterion (BIC) – a measure of model fit – across the three covariate sets. Finally, we compared the fitted estimates using each of the three covariate sets. For all three measures of error and for the male model, and for MAE and RMSE for the female model, the model including only the BMI covariate had the smallest error from the cross-validation exercise, but differences were small. For both the male and female models, the BIC was very similar across the covariate sets. Comparing fitted estimates using each of the three covariate sets, estimated prevalence of insufficient physical activity was similar at the global and regional levels and in countries with primary data available. Finally, we considered the estimates generated for countries with no, sparse or inconsistent data using mean BMI to be most plausible. Subsequent to carrying out these analyses, estimates of BMI by category, sex and country were updated for the period 1990-2022 (8), but mean BMI was not updated. Probit of the age-standardised prevalence of adult obesity is highly correlated with age-standardised mean adult BMI (Pearson correlation coefficient of 0.97). Therefore, we selected the probit of the age-standardised prevalence of obesity as the only country covariate for our final model.

5.2 Model specification

We used Bayesian hierarchical probit regression because it is able to predict the probability that an observation falls into one of two dichotomous categories (sufficient / insufficient physical activity) based on the values of predictor variables. Two separate hierarchical Bayesian hierarchical probit regression models were fit for males and females using R and the brms package (13). We used the default priors provided by the brms package. The brms formula, defining the structure of the model has the form

"m_m = brm(meet_recs|trials(meet_recs + failure_meet_recs_count) ~

(-1 + ns(midage, knots = c(30,60))|regionname_china) +

(year|regionname) +

(year|iso3) +

(0+national | survey)+

(0+other | survey)+

qcat +

malebmi_sc,

family=binomial(link = "probit"),

data=df[sex == 1],

control=list(max_treedepth = 12))"

for males. For females the model was the same with the difference of `femalebmi_sc` variable being used to describe probit of female obesity, and `data=df[sex == 2]` to select females from the data.

This model is designed to analyse the probability of "meeting physical activity recommendations" (abbreviated as "meet_recs") as a function of various predictors, including fixed and random effects. The breakdown of the key model components of the model is as follows:

- The proportion of individuals meeting requirements (`meet_recs`) among all individuals in the group (`meet_recs + failure_meet_recs_count`) is the response variable of interest.
- Formula: The formula specifies the relationship between the dependent variable and the predictor variables.
- Predictors:
    - (-1 + ns(midage, knots = c(30,60)) | regionname_china): This part of the formula specifies a non-linear spline relationship between the predictor variable `midage` (the median age of the age group) and the response, within the levels of the categorical predictor `regionname_china`

(analytical region as defined in Appendix 1 with China separate), with knots fixed at 30 and 60.
  - (year | regionname): This part accounts for random intercepts and random slopes for the variable `year` within the levels of the categorical predictor `regionname` (analytical region).
  - (year | iso3): Similar to the previous part, this component models random intercepts and random slopes for the variable `year` within different ISO3 codes representing different countries.
  - (0 + national | survey) + (0 + other | survey): This component models variation separately for survey at `national` (nationally-representative) and `other` levels. It allows for variability in survey results great than what is implied by effective sample sizes, and it allows the variability to be greater for non-national surveys than national surveys.
  - `qcat` and `malebmi_sc`: These are additional fixed effects included in the model capturing questionnaire category and the BMI covariate.
- Control Options: The control argument includes settings for controlling the MCMC sampling process. Specifically, it sets the maximum tree depth to 12.

We fit the models (one for males and one for females) using the brms package in R (14). We fitted the Bayesian model using Hamiltonian Monte Carlo. We assessed convergence of the models using the R-hat Gelman-Rubin statistic. We obtained 4,000 samples from the parameters' posterior, in turn used to obtain 4,000 posterior estimates of the prevalence of insufficient physical activity for each country-year-age-sex. With each of the 4,000 sampled prevalence values we calculated crude and age-standardized prevalence for adults aged 18 years and over for each country-year-sex. We age standardized to the WHO age standard (15). We also weighted country-year-age-sex-specific prevalences by population to obtain regional prevalences by age, year and sex for analytical regions and 2022 World Bank income groups (16), and repeated the process above to obtain crude and age-standardized prevalences for adults 18 and over for each region, year and sex. Population data were from World Population Prospects 2022 (4). All reported uncertainty intervals are Bayesian credible intervals, computed as the 2.5th-97.5th percentiles of these 4,000 draws.

5.3 Sensitivity analyses

5.3.1 Insufficient physical activity prevalence and COVID-19
We assessed whether the prevalence of insufficient physical activity changed due to the COVID-19 pandemic and response. First, we assessed our data coverage after March 2020, when the World Health Organization declared COVID-19 a global pandemic. We found that data collection was interrupted, and, to the best of our knowledge, we did not include any data collected between March and July 2020. Therefore, our estimates do not reflect the situation during initial COVID-19 closures of 2020. However, our database includes data collected starting in August 2020, when COVID-related limitations continued in many countries. In general, face-to-face data collection occurred when COVID-19 transmission was less severe and local restrictions were lifted, which varied by country, and frequency of data collection was reduced in late 2020 and 2021. We took two approaches to assess whether activity levels changed after data collection had resumed: first, we visually assessed the fit of post-March 2020 data against the model estimates. We found that the available data were generally in line with the model estimates. Second, we included an indicator variable for data collected after March 2020 in our model, to test whether these data significantly deviated from the pre-pandemic trend. We found that the coefficient for the indicator variable was not significantly different from zero and the model Bayesian Information Criterion (BIC) was not improved.

5.3.2 Linear *vs.* flexible trends over time
We modelled trends over time as a linear trend at the country and regional levels. We compared our linear model to a model with a natural cubic spline with one knot at 2010. This change was made for the regional time trends, because only a few countries had enough surveys over time to consider nonlinear country time trends. We found no statistically significant difference between the two models in terms of held-out prediction accuracy, giving support for the simpler model.

5.3.3 Inclusion of indicator variables for questionnaire type
We tested the influence of the inclusion of fixed effects for the 'Eurobarometer 2013-2022' and 'other questionnaires' survey categories. We fitted the model with an indicator variable for surveys using the IPAQ instrument, with all other surveys as reference, and compared (1) country estimates of the prevalence of physical inactivity, and (2) trends, with a focus on countries with Eurobarometer data. The fixed effect for the surveys administering IPAQ was larger in this sensitivity (0.32 (0.23, 0.40) for males and 0.38 (0.30, 0.46) for females) compared to our main model. Age-standardized estimates by sex were always within the uncertainty interval of

our main model estimate. Age-standardized country prevalence estimates by sex for 2022 ranged from 2.2 percentage points lower to 7.7 percentage points higher, with a median difference of 0.0 percentage points. Differences were most pronounced in the 27 countries administering Eurobarometer in 2022, where estimates were a median of 4.6 percentage points higher in the sensitivity analysis (range: 1.2 to 7.7 percentage points; interquartile range: 3.3 to 6.0 percentage points). Nevertheless, the number of Western European countries assessed as on track for the global target with higher certainty was the same as in our main model.

Appendix 6. Methods for assessing trends in insufficient physical activity and progress toward the 2030 physical activity target

We computed several indicators to assist in interpretation of trends in insufficient physical activity:

- The posterior probability of an increasing or decreasing trend in the prevalence of insufficient physical activity 2010-2022,
- The projected prevalence of insufficient physical activity in 2030, assuming trends 2010-2022 continue, and
- The posterior probability that the 2030 target will be met, assuming trends 2010-2022 continue.

2010 was used as the baseline for trends, as defined by the WHO Discussion Paper on the Development of an Implementation Roadmap 2023-2030 for the WHO Global Action Plan on Non-communicable Diseases 2013-2030 (17).

Posterior probabilities are a measure of certainty. They indicate – based on available data and assumptions – our estimated probability of a certain outcome being true (*e.g.*, increasing prevalence, meeting the target). To compute these indicators, we first computed the age-standardized prevalence of insufficient physical activity for each year, each sex and both sexes, and for each country, region and the globe, for every Bayesian model iteration. We then fit the following regression to the data for 2010-2022 separately for each geography-sex-iteration unit:

$$\text{probit}(prev) = \alpha + \beta * year \qquad (1)$$

We report the posterior probability that an estimated increase/decrease represents a truly increasing/decreasing trend as the percentage of draws for which β is greater than/less than 0. The projected prevalence in insufficient physical activity is computed for each geography-sex-iteration unit using the year 2030 and the fitted coefficients in equation 1. We also compute the target for every geography-sex-iteration unit as 85% of the estimated 2010 prevalence for that geography-sex-iteration. We compute 2030 target and the projected 2030 prevalence of insufficient physical activity for each geography-sex as the mean values of all iterations, and the 95% uncertainty interval as the 2.5^{th}-97.5^{th} percentile of the iterations. Finally, the posterior probability that the 2030 target will be met assuming trends 2010-2022 continue was computed as the percentage of iterations in which the projected 2030 prevalence was lower than the target prevalence. To facilitate communication of these two measures, we have created four categories that combine our certainty of whether the population is on track to meet the target with our best guess of whether the population is on track to meet the target:

**Category**	**Comparison of projection to target**	**Posterior probability (pp) of meeting the target**
On track (higher certainty)	2030 projection < 2030 target	pp ≥ 0.80
On track (lower certainty)	2030 projection < 2030 target	pp < 0.80
Off track (lower certainty)	2030 projection > 2030 target	pp > 0.20
Off track (higher certainty)	2030 projection > 2030 target	pp ≤ 0.20

Appendix 7. Flowchart of identification, inclusion and exclusion of data sources

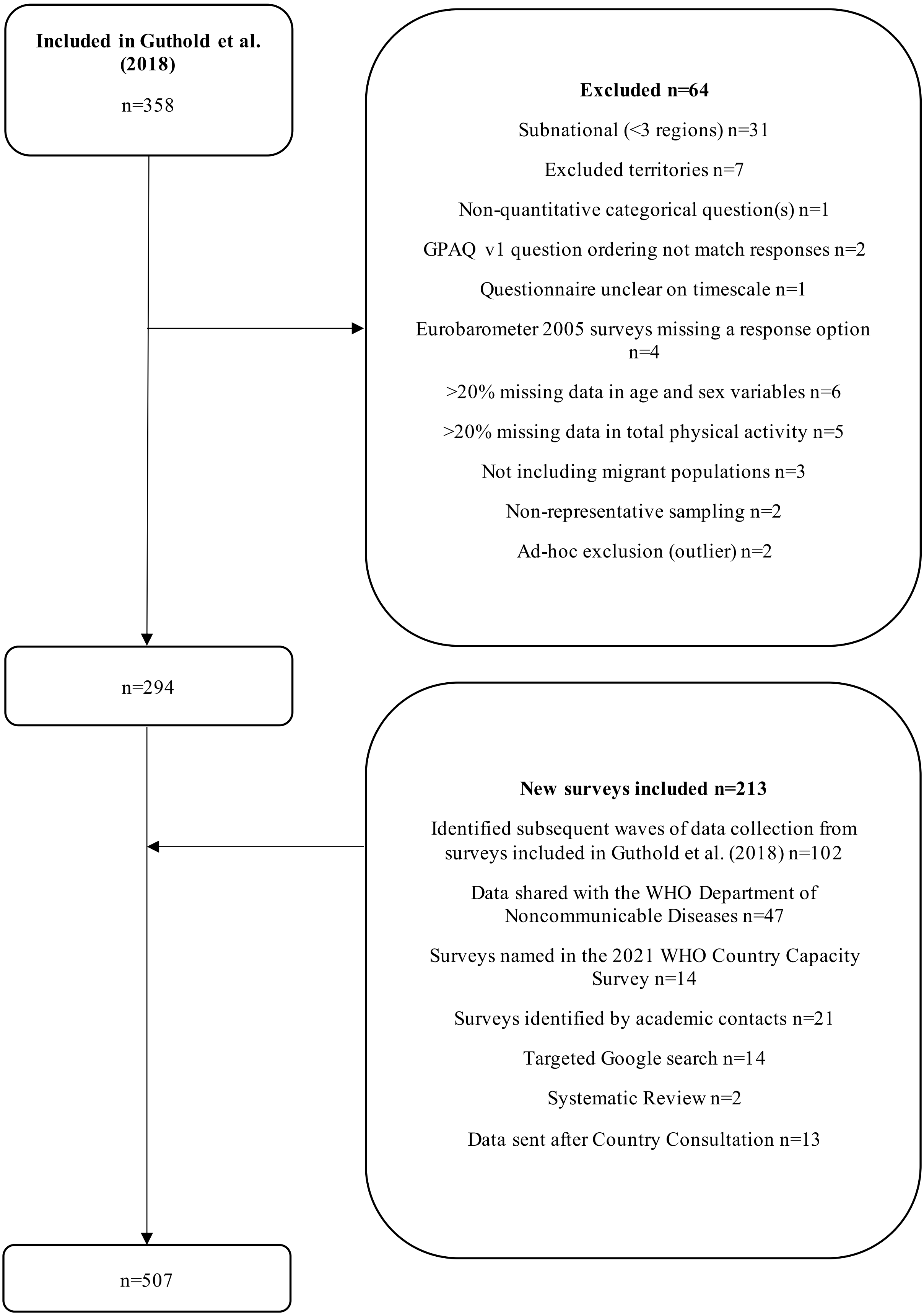

Appendix 8. Map of number of surveys included by country

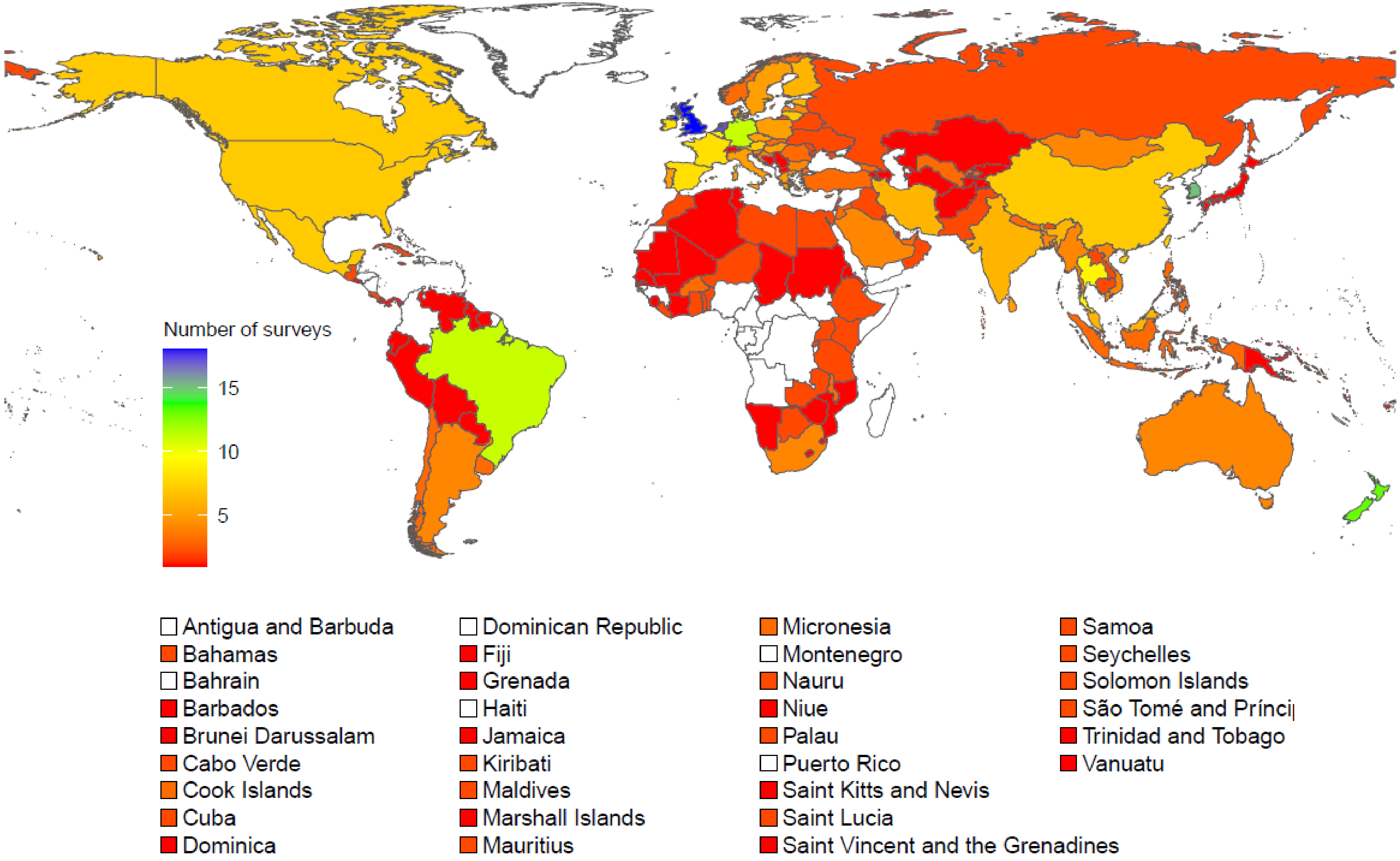

Appendix 9. Characteristics of included data sources

Country	Year	Administrative level	Questionnaire	Survey administration	Age range	Sample size	Individual-level data	Survey
Afghanistan	2018	national	GPAQ	face-to-face	18-69	3841	yes	Afghanistan STEPS 2018
Algeria	2016-2017	national	GPAQ	face-to-face	18-69	6659	yes	Algeria STEPS 2016-2017
Andorra	2004-2005	national	IPAQ	face-to-face	25-75	758	no	Andorra National Nutrition Survey 2004
Argentina	2009	national	ENFR IPAQ short 2009	face-to-face	18+	34731	yes	Argentina ENFR 2009
Argentina	2013	national	ENFR IPAQ short 2013/18	face-to-face	18+	32361	yes	Argentina ENFR 2013
Argentina	2018	national	ENFR IPAQ short 2013/18	face-to-face	18+	29222	yes	Argentina ENFR 2018
Argentina	2020-2021	subnational, urban	IPAQ	telephone	18+	4613	no	Argentina VIGITEL 2020-2021
Armenia	2016	national	GPAQ	face-to-face	18-69	2279	yes	Armenia STEPS 2016
Australia	2003	national	IPAQ	telephone	20-65	2578	yes	Australia IPS 2003
Australia	2017-2018	national	Australia NHS 2017-2018	face-to-face	18+	16370	no	Australia NHS 2017-2018
Australia	2020-2021	national	Australia NHS 2020+	web based	18+	9880	no	Australia NHS 2020-2021
Australia	2022	national	Australia NHS 2020+	web based	18+	13336	no	Australia NHS 2022
Austria	2002	national	IPAQ	face-to-face	18+	954	yes	Austria Eurobarometer 2002
Austria	2005	national	IPAQ	face-to-face	18+	977	yes	Austria Eurobarometer 2005
Austria	2013	national	Eurobarometer 2013-2022	face-to-face	18-87	944	yes	Austria Eurobarometer 2013
Austria	2017	national	GPAQ	face-to-face	18+	3955	yes	Austria BMKOES 2017
Austria	2017	national	Eurobarometer 2013-2022	face-to-face	18+	955	yes	Austria Eurobarometer 2017
Austria	2022	national	GPAQ	face-to-face and web-based	18+	3889	yes	Austria BMKOES 2022
Austria	2022	national	Eurobarometer 2013-2022	face-to-face	18-88	937	yes	Austria Eurobarometer 2022
Azerbaijan	2017	national	GPAQ	face-to-face	18-69	2771	yes	Azerbaijan STEPS 2017
Bahamas	2011-2012	national	GPAQ	face-to-face	25-64	1616	yes	Bahamas STEPS 2011-2012
Bahamas	2019	national	GPAQ	face-to-face	18-69	2248	yes	Bahamas STEPS 2019
Bangladesh	2003	national	IPAQ	face-to-face	18+	5525	yes	Bangladesh WHS 2003
Bangladesh	2009-2010	subnational	GPAQ	face-to-face	25+	9275	yes	Bangladesh STEPS 2009-2010
Bangladesh	2018	national	GPAQ	face-to-face	18-69	8118	yes	Bangladesh STEPS 2018
Bangladesh	2018-2019	national	GPAQ	face-to-face	60+	4816	no	Bangladesh Food Security and Nutrition Surveillance 2018-2019
Barbados	2007	national	GPAQ	face-to-face	25+	1188	yes	Barbados STEPS 2007
Belarus	2016	national	GPAQ	face-to-face	18-69	4985	yes	Belarus STEPS 2016
Belarus	2020	national	GPAQ	face-to-face	18-69	5315	yes	Belarus STEPS 2020
Belgium	2001	national	IPAQ	face-to-face	25+	7291	no	Belgium HIS 2001
Belgium	2002	national	IPAQ	face-to-face	18+	1054	yes	Belgium Eurobarometer 2002
Belgium	2004	national	IPAQ	face-to-face	25+	4595	no	Belgium HIS 2004
Belgium	2005	national	IPAQ	face-to-face	18+	963	yes	Belgium Eurobarometer 2005
Belgium	2008	national	IPAQ	face-to-face	25+	4720	no	Belgium HIS 2008

Country	Year	Administrative level	Questionnaire	Survey administration	Age range	Sample size	Individual-level data	Survey
Belgium	2013	national	IPAQ	face-to-face	25+	3777	no	Belgium HIS 2013
Belgium	2013	national	Eurobarometer 2013-2022	face-to-face	18+	1002	yes	Belgium Eurobarometer 2013
Belgium	2017	national	Eurobarometer 2013-2022	face-to-face	18+	950	yes	Belgium Eurobarometer 2017
Belgium	2022	national	Eurobarometer 2013-2022	face-to-face and web-based	18+	1013	yes	Belgium Eurobarometer 2022
Benin	2008	subnational	GPAQ	face-to-face	25-64	6709	yes	Benin STEPS 2008
Benin	2015	national	GPAQ	face-to-face	18-69	4960	yes	Benin STEPS 2015
Bhutan	2014	national	GPAQ	face-to-face	18-69	2712	yes	Bhutan STEPS 2014
Bhutan	2019	national	GPAQ	face-to-face	18-69	4993	yes	Bhutan STEPS 2019
Bolivia (Plurinational State of)	2019	national	GPAQ	face-to-face	18-69	4142	yes	Bolivia STEPS 2019
Bosnia and Herzegovina	2003	national	IPAQ	face-to-face	18-79	1013	yes	Bosnia and Herzegovina WHS 2003
Botswana	2007	national	GPAQ	face-to-face	25-64	3218	yes	Botswana STEPS 2007
Botswana	2014	national	GPAQ	face-to-face	18-69	3528	yes	Botswana STEPS 2014
Brazil	2003	national	IPAQ	face-to-face	18+	5000	yes	Brazil WHS 2003
Brazil	2013	subnational, urban	VIGITEL questionnaire	telephone	18-64	44355	no	Brazil VIGITEL 2013
Brazil	2013	national	PNS questionnaire	face-to-face	18+	60214	no	Brazil PNS 2013
Brazil	2013-2014	subnational, urban	GPAQ	face-to-face	18+	30085	yes	Brazil PNAUM 2013-2014
Brazil	2014	subnational, urban	VIGITEL questionnaire	telephone	18-64	35052	no	Brazil VIGITEL 2014
Brazil	2015	subnational, urban	VIGITEL questionnaire	telephone	18-64	44432	no	Brazil VIGITEL 2015
Brazil	2016	subnational, urban	VIGITEL questionnaire	telephone	18-64	35704	no	Brazil VIGITEL 2016
Brazil	2017	subnational, urban	VIGITEL questionnaire	telephone	18-64	40094	no	Brazil VIGITEL 2017
Brazil	2018	subnational, urban	VIGITEL questionnaire	telephone	18-64	39611	no	Brazil VIGITEL 2018
Brazil	2019	subnational, urban	VIGITEL questionnaire	telephone	18-64	37707	no	Brazil VIGITEL 2019
Brazil	2019	national	PNS questionnaire	face-to-face	18+	88531	yes	Brazil PNS 2019
Brunei Darussalam	2015	national	GPAQ	face-to-face	18-69	3530	yes	Brunei Darussalam STEPS 2015
Bulgaria	2005	national	IPAQ	face-to-face	18-79	933	yes	Bulgaria Eurobarometer 2005
Bulgaria	2013	national	Eurobarometer 2013-2022	face-to-face	18-89	924	yes	Bulgaria Eurobarometer 2013
Bulgaria	2017	national	Eurobarometer 2013-2022	face-to-face	18-86	884	yes	Bulgaria Eurobarometer 2017
Bulgaria	2022	national	Eurobarometer 2013-2022	face-to-face	18-88	952	yes	Bulgaria Eurobarometer 2022

Country	Year	Administrative level	Questionnaire	Survey administration	Age range	Sample size	Individual-level data	Survey
Burkina Faso	2003	national	IPAQ	face-to-face	18+	4812	yes	Burkina Faso WHS 2003
Burkina Faso	2013	national	GPAQ	face-to-face	25-64	3782	yes	Burkina Faso STEPS 2013
Burkina Faso	2021	national	GPAQ	face-to-face	18-69	3345	yes	Burkina Faso STEPS 2021
Cabo Verde	2007	national	GPAQ	face-to-face	25-64	1719	yes	Cabo Verde STEPS 2007
Cabo Verde	2020	national	GPAQ	face-to-face	18-69	4285	yes	Cabo Verde STEPS 2020
Cambodia	2010	national	GPAQ	face-to-face	25-64	5430	yes	Cambodia STEPS 2010
Cambodia	2023	national	GPAQ	face-to-face	18-69	4222	yes	Cambodia STEPS 2023
Canada	2003	national	IPAQ	telephone	18-69	2750	yes	Canada IPS 2003
Canada	2015	national	CCHS Questionnaire	face-to-face and telephone	18+	45900	no	Canada CCHS 2015
Canada	2016	national	CCHS Questionnaire	face-to-face and telephone	18+	49850	no	Canada CCHS 2016
Canada	2017	national	CCHS Questionnaire	face-to-face and telephone	18+	51000	no	Canada CCHS 2017
Canada	2018	national	CCHS Questionnaire	face-to-face and telephone	18+	48800	no	Canada CCHS 2018
Canada	2020	national	CCHS Questionnaire	face-to-face and telephone	18+	38500	no	Canada CCHS 2020
Canada	2021	national	CCHS Questionnaire	face-to-face and telephone	18+	44600	no	Canada CCHS 2021
Chad	2003	national	IPAQ	face-to-face	18+	4254	yes	Chad WHS 2003
Chile	2009-2010	national	GPAQ	face-to-face	18+	4988	yes	Chile ENS 2009-2010
Chile	2015-2016	national	IPAQ	face-to-face	18+	6669	yes	Chile ENCAVI 2015-2016
Chile	2016-2017	national	GPAQ	face-to-face	18+	5790	yes	Chile ENS 2016-2017
China	2003	national	IPAQ	face-to-face	18+	3993	yes	China WHS 2003
China	2007	national	GPAQ	face-to-face	18-69	47554	no	China STEPS 2007
China	2008	national	GPAQ	face-to-face	50+	12651	yes	China SAGE 2008
China	2010-2011	national	GPAQ	face-to-face	18+	97837	no	China CCDRFS 2010-2011
China	2013-2014	national	GPAQ	face-to-face	18+	175485	no	China CCDRFS 2013-2014
China	2015-2016	national	GPAQ	face-to-face	18+	188768	no	China CCDRFS 2015-2016
China	2018-2019	national	GPAQ	face-to-face	18+	183813	no	China CCDRFS 2018-2019
Comoros	2003	national	IPAQ	face-to-face	18+	1765	yes	Comoros WHS 2003
Comoros	2011	national	GPAQ	face-to-face	25-64	4643	yes	Comoros STEPS 2011
Cook Islands	2003	national	GPAQ	face-to-face	25-64	1819	yes	Cook Islands STEPS 2003
Cook Islands	2013-2015	national	GPAQ	face-to-face	18-64	1202	yes	Cook Islands STEPS 2013-2015
Cook Islands	2022	national	GPAQ	face-to-face	18-69	1383	yes	Cook Islands STEPS 2022
Costa Rica	2010	national	GPAQ	face-to-face	20-64	3023	no	Costa Rica Surveillance of CVD risk factors 2010
Costa Rica	2014	national	GPAQ	face-to-face	20-64	3023	no	Costa Rica Surveillance of CVD risk factors 2014
Croatia	2003	national	IPAQ	face-to-face	18+	987	yes	Croatia WHS 2003
Croatia	2013	national	Eurobarometer 2013-2022	face-to-face	18-87	919	yes	Croatia Eurobarometer 2013

Country	Year	Administrative level	Questionnaire	Survey administration	Age range	Sample size	Individual-level data	Survey
Croatia	2017	national	Eurobarometer 2013-2022	face-to-face	18+	978	yes	Croatia Eurobarometer 2017
Croatia	2022	national	Eurobarometer 2013-2022	face-to-face	18-79	970	yes	Croatia Eurobarometer 2022
Cuba	2010	national	IPAQ	face-to-face	18+	7595	no	Cuba Third Risk Factor Survey 2010
Cuba	2018-2020	national	IPAQ	face-to-face	18+	13832	no	Cuba ENS 2018-2020
Cyprus	2005	national	IPAQ	face-to-face	18-79	463	yes	Cyprus Eurobarometer 2005
Cyprus	2013	national	Eurobarometer 2013-2022	face-to-face	18+	468	yes	Cyprus Eurobarometer 2013
Cyprus	2017	national	Eurobarometer 2013-2022	face-to-face	18-86	464	yes	Cyprus Eurobarometer 2017
Cyprus	2022	national	Eurobarometer 2013-2022	face-to-face	19+	486	yes	Cyprus Eurobarometer 2022
Czechia	2002	national	IPAQ	face-to-face	20-69	1782	no	Czechia IPAQ study 2002
Czechia	2011	national	GPAQ	face-to-face	20-69	1359	no	Czechia GPAQ study 2011
Czechia	2013	national	Eurobarometer 2013-2022	face-to-face	18-79	965	yes	Czechia Eurobarometer 2013
Czechia	2017	national	Eurobarometer 2013-2022	face-to-face	18-86	966	yes	Czechia Eurobarometer 2017
Czechia	2022	national	Eurobarometer 2013-2022	face-to-face and web-based	18-88	1020	yes	Czechia Eurobarometer 2022
Côte d'Ivoire	2003	national	IPAQ	face-to-face	18+	3054	yes	Côte d'Ivoire WHS 2003
Denmark	2002	national	IPAQ	face-to-face	18+	984	yes	Denmark Eurobarometer 2002
Denmark	2005	national	IPAQ	face-to-face	18+	1007	yes	Denmark Eurobarometer 2005
Denmark	2013	national	Eurobarometer 2013-2022	face-to-face	18+	970	yes	Denmark Eurobarometer 2013
Denmark	2017	national	Eurobarometer 2013-2022	face-to-face	18+	962	yes	Denmark Eurobarometer 2017
Denmark	2022	national	Eurobarometer 2013-2022	face-to-face and web-based	18+	970	yes	Denmark Eurobarometer 2022
Dominica	2007	national	GPAQ	face-to-face	18-64	950	yes	Dominica STEPS 2007
Ecuador	2018	national	GPAQ	face-to-face	18-69	4630	yes	Ecuador STEPS 2018
Egypt	2011	national	GPAQ	face-to-face	18-65	4790	yes	Egypt STEPS 2011
Egypt	2017	national	GPAQ	face-to-face	18-69	6123	yes	Egypt STEPS 2017
El Salvador	2015	national	IPAQ	face-to-face	20+	4812	yes	El Salvador ENECA 2015
Eritrea	2010	national	GPAQ	face-to-face	25-74	6114	yes	Eritrea STEPS 2010
Estonia	2003	national	IPAQ	face-to-face	18+	1010	yes	Estonia WHS 2003
Estonia	2005	national	IPAQ	face-to-face	18+	947	yes	Estonia Eurobarometer 2005
Estonia	2013	national	Eurobarometer 2013-2022	face-to-face	18-88	954	yes	Estonia Eurobarometer 2013
Estonia	2017	national	Eurobarometer 2013-2022	face-to-face	18+	932	yes	Estonia Eurobarometer 2017
Estonia	2021-2022	national	IPAQ	unclear	25-65	596	yes	Estonia Salt consumption survey 2021-2022
Estonia	2022	national	Eurobarometer 2013-2022	face-to-face and web-based	18+	953	yes	Estonia Eurobarometer 2022
Eswatini	2014	national	GPAQ	face-to-face	18-69	2786	yes	Eswatini STEPS 2014
Ethiopia	2003	national	IPAQ	face-to-face	18+	4677	yes	Ethiopia WHS 2003
Ethiopia	2015	national	GPAQ	face-to-face	30-69	5841	no	Ethiopia STEPS 2015
Fiji	2011	national	GPAQ	face-to-face	25-64	2325	yes	Fiji STEPS 2011

Country	Year	Administrative level	Questionnaire	Survey administration	Age range	Sample size	Individual-level data	Survey
Finland	2002	national	IPAQ	face-to-face	18+	966	yes	Finland Eurobarometer 2002
Finland	2005	national	IPAQ	face-to-face	18+	978	yes	Finland Eurobarometer 2005
Finland	2011	subnational	GPAQ	face-to-face	18+	1869	last round	Finland COURAGE 2011
Finland	2013	national	Eurobarometer 2013-2022	face-to-face	18+	914	yes	Finland Eurobarometer 2013
Finland	2017	national	Eurobarometer 2013-2022	face-to-face	18+	965	yes	Finland Eurobarometer 2017
Finland	2022	national	Eurobarometer 2013-2022	face-to-face and web-based	18+	979	yes	Finland Eurobarometer 2022
France	2002	national	IPAQ	face-to-face	18-89	997	yes	France Eurobarometer 2002
France	2005	subnational	IPAQ	telephone	18-69	4815	no	France Barometre Sante 2005
France	2005	national	IPAQ	face-to-face	18+	985	yes	France Eurobarometer 2005
France	2006	national	IPAQ	face-to-face	18-74	3114	no	ENNS 2006
France	2008	national	GPAQ	telephone	18-75	3128	yes	France BNS 2008
France	2013	national	Eurobarometer 2013-2022	face-to-face	18+	978	yes	France Eurobarometer 2013
France	2017	national	Eurobarometer 2013-2022	face-to-face	18+	964	yes	France Eurobarometer 2017
France	2022	national	Eurobarometer 2013-2022	face-to-face	18+	958	yes	France Eurobarometer 2022
Gambia	2010	national	GPAQ	face-to-face	25-64	3888	yes	Gambia STEPS 2010
Georgia	2003	national	IPAQ	face-to-face	18+	2729	yes	Georgia WHS 2003
Georgia	2010	national	GPAQ	face-to-face	18-64	6423	yes	Georgia STEPS 2010
Georgia	2016	national	GPAQ	face-to-face	18-69	4135	yes	Georgia STEPS 2016
Germany	2002	national	IPAQ	face-to-face	18+	1950	yes	Germany Eurobarometer 2002
Germany	2005	national	IPAQ	face-to-face	18+	1477	yes	Germany Eurobarometer 2005
Germany	2010	national	GPAQ	telephone	18+	2450	yes	Germany DKV 2010
Germany	2012	national	GPAQ	telephone	18+	2959	yes	Germany DKV 2012
Germany	2013	national	Eurobarometer 2013-2022	face-to-face	18+	1471	yes	Germany Eurobarometer 2013
Germany	2014	national	GPAQ	telephone	18+	3027	yes	Germany DKV 2014
Germany	2016	national	GPAQ	telephone	18+	2769	yes	Germany DKV 2016
Germany	2017	national	Eurobarometer 2013-2022	face-to-face	18+	1468	yes	Germany Eurobarometer 2017
Germany	2018	national	GPAQ	telephone	18+	2871	yes	Germany DKV 2018
Germany	2021	national	GPAQ	telephone	18+	2799	yes	Germany DKV 2021
Germany	2022	national	Eurobarometer 2013-2022	face-to-face	18+	1466	yes	Germany Eurobarometer 2022
Ghana	2008	national	GPAQ	face-to-face	50+	4163	yes	Ghana SAGE 2008
Ghana	2014-2015	national	GPAQ	face-to-face	50+	3398	yes	Ghana SAGE 2014-2015
Greece	2002	national	IPAQ	face-to-face	18-88	954	yes	Greece Eurobarometer 2002
Greece	2005	national	IPAQ	face-to-face	18+	979	yes	Greece Eurobarometer 2005
Greece	2013	national	Eurobarometer 2013-2022	face-to-face	18-89	947	yes	Greece Eurobarometer 2013
Greece	2017	national	Eurobarometer 2013-2022	face-to-face	18+	965	yes	Greece Eurobarometer 2017
Greece	2022	national	Eurobarometer 2013-2022	face-to-face	18+	983	yes	Greece Eurobarometer 2022

Country	Year	Administrative level	Questionnaire	Survey administration	Age range	Sample size	Individual-level data	Survey
Grenada	2011	national	GPAQ	face-to-face	25-64	1030	yes	Grenada STEPS 2011
Guatemala	2003	national	IPAQ	face-to-face	18+	4738	yes	Guatemala WHS 2003
Guatemala	2015	national	GPAQ	face-to-face	18+	1996	yes	Guatemala STEPS 2015
Guyana	2016	national	GPAQ	face-to-face	18-69	2616	yes	Guyana STEPS 2016
Hungary	2003	national	IPAQ	face-to-face	18+	1419	yes	Hungary WHS 2003
Hungary	2005	national	IPAQ	face-to-face	18-89	980	yes	Hungary Eurobarometer 2005
Hungary	2013	national	Eurobarometer 2013-2022	face-to-face	18-88	967	yes	Hungary Eurobarometer 2013
Hungary	2017	national	Eurobarometer 2013-2022	face-to-face	18+	1001	yes	Hungary Eurobarometer 2017
Hungary	2022	national	Eurobarometer 2013-2022	face-to-face	18+	992	yes	Hungary Eurobarometer 2022
India	2003	national	IPAQ	face-to-face	18+	9357	yes	India WHS 2003
India	2007	subnational	GPAQ	face-to-face	25-64	30448	no	India STEPS 2007
India	2008	national	GPAQ	face-to-face	50+	6489	yes	India SAGE 2008
India	2009	subnational	GPAQ	face-to-face	20+	14227	no	India ICMR-INDIAB 2009
India	2011	subnational	GPAQ	face-to-face	20-69	323	no	India Validity Study 2011
India	2017-2018	national	GPAQ	face-to-face	18-69	10560	no	India NNMS 2017-2018
Indonesia	2007-2008	national	Riskedas questionnaire	face-to-face	18+	610647	yes	Indonesia Riskedas 2007-2008
Indonesia	2013	national	Riskedas questionnaire	face-to-face	18+	665920	yes	Indonesia Riskedas 2013
Indonesia	2018	national	Riskedas questionnaire	face-to-face	18+	658201	yes	Indonesia Riskedas 2018
Iran (Islamic Republic of)	2007	national	GPAQ	face-to-face	25-64	23941	no	Iran (Islamic Republic of) STEPS 2007
Iran (Islamic Republic of)	2008	national	GPAQ	face-to-face	25-64	23736	no	Iran (Islamic Republic of) STEPS 2008
Iran (Islamic Republic of)	2009	national	GPAQ	face-to-face	25-64	23828	no	Iran (Islamic Republic of) STEPS 2009
Iran (Islamic Republic of)	2011	national	GPAQ	face-to-face	25-64	24000	no	Iran (Islamic Republic of) STEPS 2011
Iran (Islamic Republic of)	2016	national	GPAQ	face-to-face	18+	25460	yes	Iran (Islamic Republic of) STEPS 2016
Iran (Islamic Republic of)	2020-2021	national	GPAQ	face-to-face	18+	27491	yes	Iran (Islamic Republic of) STEPS 2020-2021
Iraq	2006	subnational	GPAQ	face-to-face	25-65	4503	no	Iraq STEPS 2006
Iraq	2015	national	GPAQ	face-to-face	18+	3977	yes	Iraq STEPS 2015
Ireland	2002	national	IPAQ	face-to-face	18+	944	yes	Ireland Eurobarometer 2002
Ireland	2005	national	IPAQ	face-to-face	18+	958	yes	Ireland Eurobarometer 2005
Ireland	2010	national	IPAQ	face-to-face	50+	8096	no	Ireland TILDA 2010
Ireland	2013	national	Eurobarometer 2013-2022	face-to-face	18+	947	yes	Ireland Eurobarometer 2013
Ireland	2014-2015	national	IPAQ	face-to-face	25+	6462	no	Ireland Healthy Ireland Survey 2014-2015

Country	Year	Administrative level	Questionnaire	Survey administration	Age range	Sample size	Individual-level data	Survey
Ireland	2017	national	Eurobarometer 2013-2022	face-to-face	18+	966	yes	Ireland Eurobarometer 2017
Ireland	2018-2019	national	IPAQ	face-to-face	25+	6354	no	Ireland Healthy Ireland Survey 2018-2019
Ireland	2022	national	Eurobarometer 2013-2022	face-to-face	18+	979	yes	Ireland Eurobarometer 2022
Italy	2002	national	IPAQ	face-to-face	18+	986	yes	Italy Eurobarometer 2002
Italy	2005	national	IPAQ	face-to-face	18-79	957	yes	Italy Eurobarometer 2005
Italy	2013	national	Eurobarometer 2013-2022	face-to-face	18-79	941	yes	Italy Eurobarometer 2013
Italy	2017	national	Eurobarometer 2013-2022	face-to-face	18-88	958	yes	Italy Eurobarometer 2017
Italy	2022	national	Eurobarometer 2013-2022	face-to-face	18+	941	yes	Italy Eurobarometer 2022
Jamaica	2007	national	IPAQ	face-to-face	25-74	2037	no	Jamaica Health and Lifestyle Survey II 2007
Japan	2020	national	GPAQ	self-complete paper	18+	2967	no	Japan Survey on Sports Life 2020
Jordan	2004	national	GPAQ	face-to-face	18+	3127	yes	Jordan STEPS 2004
Jordan	2007	national	GPAQ	face-to-face	18+	3612	yes	Jordan STEPS 2007
Jordan	2019	national	GPAQ	face-to-face	18-69	5538	yes	Jordan STEPS 2019
Kazakhstan	2003	national	IPAQ	face-to-face	18-89	4494	yes	Kazakhstan WHS 2003
Kenya	2003	national	IPAQ	face-to-face	18+	4320	yes	Kenya WHS 2003
Kenya	2015	national	GPAQ	face-to-face	18-69	4372	yes	Kenya STEPS 2015
Kiribati	2004	subnational	GPAQ	face-to-face	18-64	1546	yes	Kiribati STEPS 2004
Kiribati	2015-2016	national	GPAQ	face-to-face	18-69	2066	yes	Kiribati STEPS 2015-2016
Kuwait	2008-2010	national	GPAQ	face-to-face	18-79	3529	yes	Kuwait WHS+GCC 2008-2010
Kyrgyzstan	2013	national	GPAQ	face-to-face	25-64	2620	yes	Kyrgyzstan STEPS 2013
Lao People's Democratic Republic	2003	national	IPAQ	face-to-face	18+	4882	yes	Lao People's Democratic Republic WHS 2003
Lao People's Democratic Republic	2013	national	GPAQ	face-to-face	18-64	2457	yes	Lao People's Democratic Republic STEPS 2013
Latvia	2005	national	IPAQ	face-to-face	18-74	914	yes	Latvia Eurobarometer 2005
Latvia	2013	national	Eurobarometer 2013-2022	face-to-face	18-74	943	yes	Latvia Eurobarometer 2013
Latvia	2017	national	Eurobarometer 2013-2022	face-to-face	18-74	939	yes	Latvia Eurobarometer 2017
Latvia	2022	national	Eurobarometer 2013-2022	face-to-face and web-based	18-79	971	yes	Latvia Eurobarometer 2022
Lebanon	2008	national	GPAQ	face-to-face	25-64	1982	no	Lebanon STEPS 2008
Lebanon	2017	national	GPAQ	face-to-face	18-69	1641	yes	Lebanon STEPS 2017
Lesotho	2012	national	GPAQ	face-to-face	25-64	1778	yes	Lesotho STEPS 2012
Liberia	2011	national	GPAQ	face-to-face	25-64	2289	yes	Liberia STEPS 2011
Liberia	2022	national	GPAQ	face-to-face	18-69	3505	yes	Liberia STEPS 2022
Libya	2009	national	GPAQ	face-to-face	25-64	3458	yes	Libya STEPS 2009
Libya	2022-2023	national	GPAQ	face-to-face	18-69	4894	yes	Libya STEPS 2022-2023
Lithuania	2003	national	IPAQ	face-to-face	18-88	2356	yes	Lithuania IPS 2003

Country	Year	Administrative level	Questionnaire	Survey administration	Age range	Sample size	Individual-level data	Survey
Lithuania	2005	national	IPAQ	face-to-face	18+	910	yes	Lithuania Eurobarometer 2005
Lithuania	2010	national	GPAQ	telephone	45-74	445	no	Lithuania National Survey 2010
Lithuania	2013	national	Eurobarometer 2013-2022	face-to-face	18+	937	yes	Lithuania Eurobarometer 2013
Lithuania	2017	national	Eurobarometer 2013-2022	face-to-face	18+	969	yes	Lithuania Eurobarometer 2017
Lithuania	2019	subnational	GPAQ	face-to-face	18-75	2910	no	Lithuania National Survey of Diet and Physical Activity 2019
Lithuania	2022	national	Eurobarometer 2013-2022	face-to-face	18+	951	yes	Lithuania Eurobarometer 2022
Luxembourg	2002	national	IPAQ	face-to-face	18-79	554	yes	Luxembourg Eurobarometer 2002
Luxembourg	2005	national	IPAQ	face-to-face	18-85	467	yes	Luxembourg Eurobarometer 2005
Luxembourg	2008	national	IPAQ	self-completed	18-69	1379	no	Luxembourg ORISCAV-LUX 2008
Luxembourg	2013	national	Eurobarometer 2013-2022	face-to-face	18+	451	yes	Luxembourg Eurobarometer 2013
Luxembourg	2016-2018	national	IPAQ	web based	25-79	864	no	Luxembourg ORISCAV-LUX 2 2016-2018
Luxembourg	2017	national	Eurobarometer 2013-2022	face-to-face	18+	453	yes	Luxembourg Eurobarometer 2017
Luxembourg	2022	national	Eurobarometer 2013-2022	face-to-face	18+	464	yes	Luxembourg Eurobarometer 2022
Malawi	2003	national	IPAQ	face-to-face	18+	5301	yes	Malawi WHS 2003
Malawi	2009	national	GPAQ	face-to-face	25-64	4057	yes	Malawi STEPS 2009
Malawi	2017	national	GPAQ	face-to-face	18-69	3369	yes	Malawi STEPS 2017
Malaysia	2003	national	IPAQ	face-to-face	18+	6004	yes	Malaysia WHS 2003
Malaysia	2005	national	GPAQ	face-to-face	25-64	2572	no	Malaysia STEPS 2005
Malaysia	2006	national	GPAQ	face-to-face	18-64	28290	no	Malaysia NHMS 2006
Malaysia	2011	national	IPAQ	face-to-face	25-64	13461	no	Malaysia NHMS 2011
Malaysia	2015	national	IPAQ	face-to-face	18+	19700	no	Malaysia NHMS 2015
Malaysia	2019	national	IPAQ	face-to-face	18+	10356	no	Malaysia National Health and Morbidity Survey 2019
Maldives	2011	subnational, urban	GPAQ	face-to-face	18-64	1555	yes	Maldives STEPS 2011
Maldives	2020-2021	national	GPAQ	face-to-face	18-69	2617	yes	Maldives STEPS 2020-2021
Mali	2007	subnational	GPAQ	face-to-face	18-64	1579	yes	Mali STEPS 2007
Malta	2005	national	IPAQ	face-to-face	18+	483	yes	Malta Eurobarometer 2005
Malta	2013	national	Eurobarometer 2013-2022	face-to-face	18-89	460	yes	Malta Eurobarometer 2013
Malta	2017	national	Eurobarometer 2013-2022	face-to-face	18+	481	yes	Malta Eurobarometer 2017
Malta	2022	national, urban	Eurobarometer 2013-2022	face-to-face and web-based	18+	479	yes	Malta Eurobarometer 2022
Marshall Islands	2002	subnational	GPAQ	face-to-face	18-64	2592	yes	Marshall Islands STEPS 2002
Mauritania	2003	national	IPAQ	face-to-face	18+	3419	yes	Mauritania WHS 2003
Mauritius	2003	national	IPAQ	face-to-face	18+	3891	yes	Mauritius WHS 2003
Mauritius	2021	national	GPAQ	face-to-face	25-74	3408	no	Mauritius NCD Survey 2021
Mexico	2003	national	IPAQ	face-to-face	18+	38610	yes	Mexico WHS 2003

Country	Year	Administrative level	Questionnaire	Survey administration	Age range	Sample size	Individual-level data	Survey
Mexico	2006	national	IPAQ	face-to-face	20+	45071	yes	Mexico ENSANUT 2006
Mexico	2008	national	GPAQ	face-to-face	50+	2186	yes	Mexico SAGE 2008
Mexico	2012	national	IPAQ	face-to-face	18-69	11656	yes	Mexico ENSANUT 2012
Mexico	2016	national	IPAQ	face-to-face	20-69	7453	yes	Mexico ENSANUT 2016
Mexico	2018	national	IPAQ	face-to-face	18-69	42302	yes	Mexico ENSANUT 2018-2019
Mexico	2022	national	IPAQ	face-to-face	18-69	7590	yes	Mexico ENSANUT 2022
Micronesia (Federated States of)	2002	subnational	GPAQ	face-to-face	25-64	1447	yes	Micronesia (Federated States of) STEPS 2002
Micronesia (Federated States of)	2006-2009	subnational	GPAQ	face-to-face	18-64	6143	yes	Micronesia (Federated States of) STEPS 2006-2009
Micronesia (Federated States of)	2016	subnational	GPAQ	face-to-face	18-64	1803	yes	Micronesia (Federated States of) STEPS 2016
Mongolia	2005	national	GPAQ	face-to-face	18-64	2502	yes	Mongolia STEPS 2005
Mongolia	2009	national	GPAQ	face-to-face	18-64	5017	yes	Mongolia STEPS 2009
Mongolia	2013	national	GPAQ	face-to-face	18-64	5190	yes	Mongolia STEPS 2013
Mongolia	2019	national	GPAQ	face-to-face	18-69	6324	yes	Mongolia STEPS 2019
Morocco	2008	subnational	IPAQ	face-to-face	18-47	1751	no	Morocco IPAQ Survey 2008
Morocco	2017	national	GPAQ	face-to-face	18+	10788	yes	Morocco STEPS 2017
Mozambique	2005	national	GPAQ	face-to-face	24-64	2998	yes	Mozambique STEPS 2005
Myanmar	2003	subnational	GPAQ	face-to-face	25-74	4448	no	Myanmar STEPS 2003
Myanmar	2003	national	IPAQ	face-to-face	18+	5886	yes	Myanmar WHS 2003
Myanmar	2009	subnational	GPAQ	face-to-face	25-64	6414	no	Myanmar STEPS 2009
Myanmar	2014	national	GPAQ	face-to-face	25-64	8143	yes	Myanmar STEPS 2014
Namibia	2003	national	IPAQ	face-to-face	18+	3975	yes	Namibia WHS 2003
Nauru	2004	national	GPAQ	face-to-face	18-64	1942	yes	Nauru STEPS 2004
Nauru	2015	national	GPAQ	face-to-face	18-69	1342	yes	Nauru STEPS 2015
Nepal	2003	national	IPAQ	face-to-face	18+	8670	yes	Nepal WHS 2003
Nepal	2012-2013	national	GPAQ	face-to-face	18-69	4006	yes	Nepal STEPS 2012-2013
Nepal	2019	national	GPAQ	face-to-face	18-69	5276	yes	Nepal STEPS 2019
Netherlands	2002	national	IPAQ	face-to-face	18-87	977	yes	Netherlands Eurobarometer 2002
Netherlands	2005	national	IPAQ	face-to-face	18+	993	yes	Netherlands Eurobarometer 2005
Netherlands	2010	national	NL Lifestyle Monitor	web based and face-to-face	18+	13676	no	Netherlands Lifestyle Monitor 2010
Netherlands	2011	national	NL Lifestyle Monitor	web based and face-to-face	18+	5936	no	Netherlands Lifestyle Monitor 2011
Netherlands	2012	national	NL Lifestyle Monitor	web based and face-to-face	18+	6058	no	Netherlands Lifestyle Monitor 2012
Netherlands	2013	national	Eurobarometer 2013-2022	face-to-face	18+	968	yes	Netherlands Eurobarometer 2013

Country	Year	Administrative level	Questionnaire	Survey administration	Age range	Sample size	Individual-level data	Survey
Netherlands	2013	national	NL Lifestyle Monitor	web based and face-to-face	18+	6102	no	Netherlands Lifestyle Monitor 2013
Netherlands	2014	national	NL Lifestyle Monitor	web based and face-to-face	18+	7304	no	Netherlands Lifestyle Monitor 2014
Netherlands	2015	national	NL Lifestyle Monitor	web based and face-to-face	18+	7238	no	Netherlands Lifestyle Monitor 2015
Netherlands	2016	national	NL Lifestyle Monitor	web based and face-to-face	18+	7099	no	Netherlands Lifestyle Monitor 2016
Netherlands	2017	national	Eurobarometer 2013-2022	face-to-face	18-78	976	yes	Netherlands Eurobarometer 2017
Netherlands	2017	national	NL Lifestyle Monitor	web based and face-to-face	18+	7635	no	Netherlands Lifestyle Monitor 2017
Netherlands	2018	national	NL Lifestyle Monitor	web based and face-to-face	18+	8028	no	Netherlands Lifestyle Monitor 2018
Netherlands	2019	national	NL Lifestyle Monitor	web based and face-to-face	18+	7821	no	Netherlands Lifestyle Monitor 2019
Netherlands	2020	national	NL Lifestyle Monitor	web based and face-to-face	18+	7028	no	Netherlands Lifestyle Monitor 2020
Netherlands	2021	national	NL Lifestyle Monitor	web based and face-to-face	18+	6862	no	Netherlands Lifestyle Monitor 2021
Netherlands	2022	national	Eurobarometer 2013-2022	face-to-face and web-based	18-78	996	yes	Netherlands Eurobarometer 2022
New Zealand	2003	national	IPAQ	telephone	18-65	1494	yes	New Zealand IPS 2003
New Zealand	2006-2007	national	NZPAQ	face-to-face	18+	11923	yes	New Zealand Health Survey 2006-2007
New Zealand	2011-2012	national	NZPAQ	face-to-face	18+	12201	yes	New Zealand Health Survey 2011-2012
New Zealand	2012-2013	national	NZPAQ	face-to-face	18+	12606	yes	New Zealand Health Survey 2012-2013
New Zealand	2013-2014	national	NZPAQ	face-to-face	18+	12890	yes	New Zealand Health Survey 2013-2014
New Zealand	2014-2015	national	NZPAQ	face-to-face	18+	13021	yes	New Zealand Health Survey 2014-2015
New Zealand	2015-2016	national	NZPAQ	face-to-face	18+	13297	yes	New Zealand Health Survey 2015-2016
New Zealand	2016-2017	national	NZPAQ	face-to-face	18+	13151	yes	New Zealand Health Survey 2016-2017
New Zealand	2017-2018	national	NZPAQ	face-to-face	18+	13449	yes	New Zealand Health Survey 2017-2018
New Zealand	2018-2019	national	NZPAQ	face-to-face	18+	13152	yes	New Zealand Health Survey 2018-2019
New Zealand	2019-2020	national	IPAQ long	web based and self-complete paper	18+	13756	no	New Zealand Active NZ 2019-2020
New Zealand	2019-2020	national	NZPAQ	face-to-face	18+	9418	yes	New Zealand Health Survey 2019-2020
New Zealand	2020-2021	national	NZPAQ	face-to-face	18+	9438	yes	New Zealand Health Survey 2020-2021
Niger	2007	subnational	GPAQ	face-to-face	18-64	2417	yes	Niger STEPS 2007
Niger	2021	national	GPAQ	face-to-face	18-69	5551	yes	Niger STEPS 2021
Niue	2011-2012	national	GPAQ	face-to-face	18-79	831	yes	Niue STEPS 2011-2012
Norway	2003	national	IPAQ	self administered	18-65	1630	yes	Norway IPS 2003
Norway	2008-2009	subnational	IPAQ	self-administered	19-84	3332	yes	Norway KAN1 2008-2009

Country	Year	Administrative level	Questionnaire	Survey administration	Age range	Sample size	Individual-level data	Survey
Norway	2014-2015	subnational	IPAQ	self-administered	19-85	3131	yes	Norway KAN2 2014-2015
Occupied Palestinian Territory	2010	national	GPAQ	face-to-face	25-64	5105	yes	Occupied Palestinian Territory STEPS 2010
Occupied Palestinian Territory	2022	national	GPAQ	face-to-face	18-69	5469	yes	Occupied Palestinian Territory STEPS 2022
Oman	2007-2008	national	GPAQ	face-to-face	18+	4337	yes	Oman WHS+GCC 2007-2008
Oman	2017	national	GPAQ	face-to-face	18+	6438	yes	Oman STEPS 2017
Pakistan	2003	national	IPAQ	face-to-face	18+	6103	yes	Pakistan WHS 2003
Pakistan	2014	national	GPAQ	face-to-face	18-69	7052	yes	Pakistan STEPS 2014
Palau	2011-2013	national	GPAQ	face-to-face	25-64	1977	yes	Palau STEPS 2011-2013
Palau	2016	subnational	GPAQ	face-to-face	18+	1748	yes	Palau STEPS 2016
Panama	2019	national	GPAQ	face-to-face	18+	17303	yes	Panama National Health Survey 2019
Papua New Guinea	2007	national	GPAQ	face-to-face	18-64	2395	yes	Papua New Guinea STEPS 2007
Paraguay	2003	national	IPAQ	face-to-face	18+	5119	yes	Paraguay WHS 2003
Peru	2017-2018	national	IPAQ	face-to-face	18-59	1082	yes	Peru VIANEV 2017-2018
Philippines	2003	national	IPAQ	face-to-face	18+	10079	yes	Philippines WHS 2003
Philippines	2013	national	GPAQ	face-to-face	18+	19560	no	Philippines National Nutrition Survey 2013
Philippines	2015	national	GPAQ	face-to-face	18+	21635	no	Philippines National Nutrition Survey 2015
Poland	2005	national	IPAQ	face-to-face	18+	944	yes	Poland Eurobarometer 2005
Poland	2011	subnational	GPAQ	face-to-face	18+	3855	last round	Poland COURAGE 2011
Poland	2013	national	Eurobarometer 2013-2022	face-to-face	18+	888	yes	Poland Eurobarometer 2013
Poland	2017	national	Eurobarometer 2013-2022	face-to-face	18+	890	yes	Poland Eurobarometer 2017
Poland	2022	national	Eurobarometer 2013-2022	face-to-face	18+	961	yes	Poland Eurobarometer 2022
Portugal	2002	national	IPAQ	face-to-face	18-88	946	yes	Portugal Eurobarometer 2002
Portugal	2005	national	IPAQ	face-to-face	18+	958	yes	Portugal Eurobarometer 2005
Portugal	2013	national	Eurobarometer 2013-2022	face-to-face	18+	1001	yes	Portugal Eurobarometer 2013
Portugal	2017	national	Eurobarometer 2013-2022	face-to-face	18+	1025	yes	Portugal Eurobarometer 2017
Portugal	2022	national	Eurobarometer 2013-2022	face-to-face	18+	957	yes	Portugal Eurobarometer 2022
Qatar	2008-2009	national	GPAQ	face-to-face	18-79	4451	yes	Qatar WHS+GCC 2008-2009
Republic of Korea	2007	national	IPAQ	face-to-face	19+	2875	no	Republic of Korea KNHANES 2007
Republic of Korea	2008	national	IPAQ	face-to-face	19+	6763	no	Republic of Korea KNHANES 2008
Republic of Korea	2009	national	IPAQ	face-to-face	19+	7471	no	Republic of Korea KNHANES 2009
Republic of Korea	2010	national	IPAQ	face-to-face	19+	6242	no	Republic of Korea KNHANES 2010
Republic of Korea	2011	national	IPAQ	face-to-face	19+	6003	no	Republic of Korea KNHANES 2011
Republic of Korea	2012	national	IPAQ	face-to-face	19+	5573	no	Republic of Korea KNHANES 2012

Country	Year	Administrative level	Questionnaire	Survey administration	Age range	Sample size	Individual-level data	Survey
Republic of Korea	2013	national	IPAQ	face-to-face	19+	5327	no	Republic of Korea KNHANES 2013
Republic of Korea	2014	national	GPAQ	face-to-face	19+	4981	yes	Republic of Korea KNHANES 2014
Republic of Korea	2015	national	GPAQ	face-to-face	19+	5075	yes	Republic of Korea KNHANES 2015
Republic of Korea	2016	national	GPAQ	face-to-face	19+	5807	yes	Republic of Korea KNHANES 2016
Republic of Korea	2017	national	GPAQ	face-to-face	19+	5816	yes	Republic of Korea KNHANES 2017
Republic of Korea	2018	national	GPAQ	face-to-face	19+	5963	yes	Republic of Korea KNHANES 2018
Republic of Korea	2019	national	GPAQ	face-to-face	19+	5919	yes	Republic of Korea KNHANES 2019
Republic of Korea	2020	national	GPAQ	face-to-face	19+	5399	yes	Republic of Korea KNHANES 2020
Republic of Korea	2021	national	GPAQ	face-to-face	19+	5320	yes	Republic of Korea KNHANES 2021
Republic of Moldova	2013	national	GPAQ	face-to-face	18-69	4608	yes	Republic of Moldova STEPS 2013
Republic of Moldova	2021	national	GPAQ	face-to-face	18-69	4032	yes	Republic of Moldova STEPS 2021
Republic of North Macedonia	2015	national, urban	IPAQ	telephone	18-78	485	yes	North Macedonia Nutrition Survey 2015
Romania	2013	national	Eurobarometer 2013-2022	face-to-face	18+	869	yes	Romania Eurobarometer 2013
Romania	2017	national	Eurobarometer 2013-2022	face-to-face	18+	919	yes	Romania Eurobarometer 2017
Romania	2022	national	Eurobarometer 2013-2022	face-to-face	18-79	940	yes	Romania Eurobarometer 2022
Russian Federation	2003	national	IPAQ	face-to-face	18+	4406	yes	Russian Federation WHS 2003
Russian Federation	2008	national	GPAQ	face-to-face	50+	3846	yes	Russian Federation SAGE 2008
Rwanda	2012	national	GPAQ	face-to-face	18-64	6537	yes	Rwanda STEPS 2012
Rwanda	2021-2022	national	GPAQ	face-to-face	18-69	5519	yes	Rwanda STEPS 2021-2022
Saint Kitts and Nevis	2007	subnational	GPAQ	face-to-face	25-64	1404	yes	Saint Kitts and Nevis STEPS 2007
Saint Lucia	2012	subnational	GPAQ	face-to-face	25-64	1674	yes	Saint Lucia STEPS 2012
Saint Lucia	2019-2020	national	GPAQ	face-to-face	18-69	2865	yes	Saint Lucia STEPS 2019-2020
Saint Vincent and the Grenadines	2013-2014	national	GPAQ	face-to-face	18-69	3467	yes	Saint Vincent and the Grenadines STEPS 2013-2014
Samoa	2002	national	GPAQ	face-to-face	25-64	2692	yes	Samoa STEPS 2002
Samoa	2013	national	GPAQ	face-to-face	25-64	1412	yes	Samoa STEPS 2013
Sao Tome and Principe	2009	national	GPAQ	face-to-face	25-64	2261	yes	Sao Tome and Principe STEPS 2009
Sao Tome and Principe	2019	national	GPAQ	face-to-face	18-69	2320	yes	Sao Tome and Principe STEPS 2019
Saudi Arabia	2005	national	GPAQ	face-to-face	25-64	3546	no	Saudi Arabia STEPS 2005
Saudi Arabia	2008-2009	national	GPAQ	face-to-face	18+	8085	yes	Saudi Arabia WHS+GCC 2008-2009
Saudi Arabia	2013	national	GPAQ	face-to-face	25-64	7478	no	Saudi Arabia Health Interview Survey 2013
Saudi Arabia	2019	national	IPAQ	face-to-face	18+	8361	yes	Saudi Arabia World Health Survey 2019
Senegal	2015	national	GPAQ	face-to-face	18-69	5052	no	Senegal STEPS 2015
Serbia	2006	national	IPAQ	face-to-face	25+	7700	no	Serbia National Health Survey 2006
Seychelles	2004	national	GPAQ	face-to-face	25-64	1253	yes	Seychelles Heart Survey III 2004

Country	Year	Administrative level	Questionnaire	Survey administration	Age range	Sample size	Individual-level data	Survey
Seychelles	2013	national	GPAQ	face-to-face	25-64	1239	yes	Seychelles Heart Survey IV 2013
Sierra Leone	2009	national	GPAQ	face-to-face	25-64	2413	yes	Sierra Leone STEPS 2009
Singapore	2007	national	GPAQ	face-to-face	18-69	6929	no	Singapore National Health Survey 2007
Singapore	2010	national	GPAQ	face-to-face	18-69	4044	no	Singapore National Health Survey 2010
Singapore	2013	national	GPAQ	face-to-face	18+	9337	no	Singapore National Health Survey 2013
Singapore	2018-2019	national	GPAQ	face-to-face	18-74	6248	no	Singapore National Population Health Survey 2018-2019
Singapore	2019-2020	national	GPAQ	face-to-face	18-74	6250	no	Singapore National Population Health Survey 2019-2020
Slovakia	2013	national	Eurobarometer 2013-2022	face-to-face	18-79	917	yes	Slovakia Eurobarometer 2013
Slovakia	2017	national	Eurobarometer 2013-2022	face-to-face	18+	965	yes	Slovakia Eurobarometer 2017
Slovakia	2022	national	Eurobarometer 2013-2022	face-to-face	18-88	941	yes	Slovakia Eurobarometer 2022
Slovenia	2005	national	IPAQ	face-to-face	18-85	973	yes	Slovenia Eurobarometer 2005
Slovenia	2013	national	Eurobarometer 2013-2022	face-to-face	18+	1052	yes	Slovenia Eurobarometer 2013
Slovenia	2017	national	Eurobarometer 2013-2022	face-to-face	18+	980	yes	Slovenia Eurobarometer 2017
Slovenia	2022	national	Eurobarometer 2013-2022	face-to-face and web-based	18+	973	yes	Slovenia Eurobarometer 2022
Solomon Islands	2005-2006	national	GPAQ	face-to-face	18-64	2658	yes	Solomon Islands STEPS 2005-2006
Solomon Islands	2015	national	GPAQ	face-to-face	18-69	2361	yes	Solomon Islands STEPS 2015
South Africa	2003	national	IPAQ	face-to-face	18-79	2460	yes	South Africa WHS 2003
South Africa	2003	national	GPAQ	face-to-face	25-64	4342	no	DHS 2003
South Africa	2008	national	GPAQ	face-to-face	50+	3538	yes	South Africa SAGE 2008
South Africa	2014-2015	national	GPAQ	face-to-face	50+	1977	yes	South Africa SAGE 2014-2015
Spain	2002	national	IPAQ	face-to-face	18-89	913	yes	Spain Eurobarometer 2002
Spain	2005	national	IPAQ	face-to-face	18+	949	yes	Spain Eurobarometer 2005
Spain	2011	subnational	GPAQ	face-to-face	18+	4530	last round	Spain COURAGE 2011
Spain	2011	national	IPAQ	face-to-face	18-69	15741	no	Spain National Health Survey 2011
Spain	2013	national	Eurobarometer 2013-2022	face-to-face	18+	977	yes	Spain Eurobarometer 2013
Spain	2016-2017	national	IPAQ	face-to-face	25-69	15362	no	Spain ENSE 2016-2017
Spain	2017	national	Eurobarometer 2013-2022	face-to-face	18-89	988	yes	Spain Eurobarometer 2017
Spain	2022	national	Eurobarometer 2013-2022	face-to-face	18+	964	yes	Spain Eurobarometer 2022
Sri Lanka	2003	national	IPAQ	face-to-face	18+	6557	yes	Sri Lanka WHS 2003
Sri Lanka	2005	subnational	IPAQ	face-to-face	18+	4492	yes	Sri Lanka DCS 2005
Sri Lanka	2006	subnational	GPAQ	face-to-face	18-64	11282	yes	Sri Lanka STEPS 2006
Sri Lanka	2014	national	GPAQ	face-to-face	18-69	5100	yes	Sri Lanka STEPS 2014
Sri Lanka	2021	national	GPAQ	face-to-face	18-69	6239	yes	Sri Lanka STEPS 2021
Sudan	2016	national	GPAQ	face-to-face	18-69	7563	yes	Sudan STEPS 2016
Suriname	2013	national	GPAQ	face-to-face	18-64	4939	no	Suriname STEPS 2013

Country	Year	Administrative level	Questionnaire	Survey administration	Age range	Sample size	Individual-level data	Survey
Sweden	2002	national	IPAQ	face-to-face	18+	982	yes	Sweden Eurobarometer 2002
Sweden	2005	national	IPAQ	face-to-face	18+	1016	yes	Sweden Eurobarometer 2005
Sweden	2013	national	Eurobarometer 2013-2022	face-to-face	18+	973	yes	Sweden Eurobarometer 2013
Sweden	2017	national	Eurobarometer 2013-2022	face-to-face	18+	1012	yes	Sweden Eurobarometer 2017
Sweden	2022	national	Eurobarometer 2013-2022	face-to-face and web-based	18-89	1014	yes	Sweden Eurobarometer 2022
Switzerland	2014-2015	national	IPAQ	self-administered (paper)	18-76	2031	yes	Switzerland menuCH 2014-2015
Tajikistan	2016	national	GPAQ	face-to-face	18-69	2549	yes	Tajikistan STEPS 2016
Thailand	2008	national	GPAQ	face-to-face	30+	17590	no	Thailand National Health Examination Survey IV 2008
Thailand	2012	national	GPAQ	face-to-face	18-79	5648	no	Thailand Surveillance on Physical Activity 2012
Thailand	2013	national	GPAQ	face-to-face	18-79	5751	no	Thailand Surveillance on Physical Activity 2013
Thailand	2014	national	GPAQ	face-to-face	30+	16824	no	Thailand National Health Examination Survey V 2014
Thailand	2014	national	GPAQ	face-to-face	18-79	5840	no	Thailand Surveillance on Physical Activity 2014
Thailand	2015	national	GPAQ	face-to-face	18-79	5954	no	Thailand Surveillance on Physical Activity 2015
Thailand	2016	national	GPAQ	face-to-face	18+	6074	no	Thailand Surveillance on Physical Activity 2016
Thailand	2019-2020	national	GPAQ	face-to-face	30+	18897	no	Thailand National Health Examination Survey VI 2019-2020
Thailand	2022	national	GPAQ	face-to-face	18+	6513	no	Thailand Surveillance on Physical Activity 2022
Timor-Leste	2014	subnational	GPAQ	face-to-face	18-69	2300	yes	Timor-Leste STEPS 2014
Togo	2011	national	GPAQ	face-to-face	18-64	3629	yes	Togo STEPS 2011
Togo	2021-2022	national	GPAQ	face-to-face	18-69	3851	yes	Togo STEPS 2021-2022
Tonga	2004	subnational	GPAQ	face-to-face	18-64	901	yes	Tonga STEPS 2004
Tonga	2011	national	GPAQ	face-to-face	25-64	2422	yes	Tonga STEPS 2011
Tonga	2017	national	GPAQ	face-to-face	18-69	3784	yes	Tonga STEPS 2017
Trinidad and Tobago	2011	national	GPAQ	face-to-face	18-64	2500	yes	Trinidad and Tobago STEPS 2011
Tunisia	2003	national	IPAQ	face-to-face	18+	5037	yes	Tunisia WHS 2003
Türkiye	2003	national	IPAQ	face-to-face	18+	9996	yes	Turkey WHS 2003
Türkiye	2005	national	IPAQ	face-to-face	18-79	945	yes	Turkey Eurobarometer 2005
Türkiye	2017	national	GPAQ	face-to-face	18+	5695	yes	Turkey STEPS 2017
Turkmenistan	2018	national	GPAQ	face-to-face	18-69	4011	yes	Turkmenistan STEPS 2018
Tuvalu	2015	national	GPAQ	face-to-face	18-69	1144	yes	Tuvalu STEPS 2015
Uganda	2014	national	GPAQ	face-to-face	18-69	3846	yes	Uganda STEPS 2014
Uganda	2023	national	GPAQ	face-to-face	18-69	3464	yes	Uganda STEPS 2023
Ukraine	2003	national	IPAQ	face-to-face	18+	2408	yes	Ukraine WHS 2003
Ukraine	2019	national	GPAQ	face-to-face	18-69	3988	yes	Ukraine STEPS 2019
United Arab Emirates	2003	national	IPAQ	face-to-face	18-68	1150	yes	United Arab Emirates WHS 2003

Country	Year	Administrative level	Questionnaire	Survey administration	Age range	Sample size	Individual-level data	Survey
United Arab Emirates	2008-2009	national	GPAQ	face-to-face	18-79	2451	yes	United Arab Emirates WHS+GCC 2008-2009
United Arab Emirates	2017-2018	national	GPAQ	face-to-face	18+	7717	yes	United Arab Emirates STEPS 2017-2018
United Kingdom	2002	national	IPAQ	face-to-face	18+	1266	yes	UK Eurobarometer 2002
United Kingdom	2005	national	IPAQ	face-to-face	18+	1272	yes	UK Eurobarometer 2005
United Kingdom	2012	subnational	England/NI Long PA module questionnaire	face-to-face	25+	7042	no	United Kingdom HSE 2012
United Kingdom	2013	national	Eurobarometer 2013-2022	face-to-face	18+	1263	yes	UK Eurobarometer 2013
United Kingdom	2013	subnational	IPAQ	face-to-face	18+	7685	yes	United Kingdom HSE 2013
United Kingdom	2014	subnational	HSE IPAQ short version 2014	face-to-face	18+	6968	yes	United Kingdom HSE 2014
United Kingdom	2015	subnational	UK surveys IPAQ short version	face-to-face	25+	6599	yes	United Kingdom HSE 2015
United Kingdom	2016	subnational	England/NI Long PA module questionnaire	face-to-face	25+	6853	no	United Kingdom HSE 2016
United Kingdom	2017	subnational	IPAQ	face-to-face	25+	6719	yes	United Kingdom HSE 2017
United Kingdom	2017	national	Eurobarometer 2013-2022	face-to-face	18+	1273	yes	UK Eurobarometer 2017
United Kingdom	2017-2018	subnational	UK surveys IPAQ short version	face-to-face	18+	5569	yes	United Kingdom NSW 2017-2018
United Kingdom	2018	subnational	IPAQ	face-to-face	25+	6687	yes	United Kingdom HSE 2018
United Kingdom	2018-2019	subnational	UK surveys IPAQ short version	face-to-face	18+	5851	yes	United Kingdom NSW 2018-2019
United Kingdom	2019-2020	subnational	UK surveys IPAQ short version	face-to-face	18+	6213	yes	United Kingdom NSW 2019-2020
United Kingdom	2020	subnational	UK surveys IPAQ short version	telephone	18+	1906	yes	United Kingdom SHeS 2020
United Kingdom	2020-2021	subnational	UK surveys IPAQ short version	telephone	18+	7193	yes	United Kingdom NSW 2020-2021
United Kingdom	2021	subnational	England/NI Long PA module questionnaire	face-to-face	25+	5069	no	United Kingdom HSE 2021
United Kingdom	2021-2022	subnational	UK surveys IPAQ short version	telephone and face-to-face	18+	6367	yes	United Kingdom NSW 2021-2022
United Republic of Tanzania	2012	national	GPAQ	face-to-face	25-64	5480	yes	United Republic of Tanzania STEPS 2012
United Republic of Tanzania	2023	national	GPAQ	face-to-face	18-69	3481	yes	United Republic of Tanzania STEPS 2023
United States of America	2003	national	IPAQ	telephone	18+	5507	yes	United States of America IPS 2003
United States of America	2007-2008	national	GPAQ	face-to-face	18-80	6198	yes	United States NHANES 2007-2008
United States of America	2009-2010	national	GPAQ	face-to-face	18-80	6511	yes	United States NHANES 2009-2010
United States of America	2011-2012	national	GPAQ	face-to-face	18-80	5844	yes	United States NHANES 2011-2012

Country	Year	Administrative level	Questionnaire	Survey administration	Age range	Sample size	Individual-level data	Survey
United States of America	2013-2014	national	GPAQ	face-to-face	18-80	6094	yes	United States NHANES 2013-2014
United States of America	2015-2016	national	GPAQ	face-to-face	18-80	5934	yes	United States NHANES 2015-2016
United States of America	2017-2020	national	GPAQ	face-to-face	18-80	9615	yes	United States NHANES 2017-2020
Uruguay	2003	national	IPAQ	face-to-face	18+	2983	yes	Uruguay WHS 2003
Uruguay	2006	subnational, urban	GPAQ	face-to-face	25-64	1966	yes	Uruguay STEPS 2006
Uruguay	2013-2014	national, urban	GPAQ	face-to-face	18-64	2340	yes	Uruguay STEPS 2013-2014
Uzbekistan	2002	national	IPAQ	face-to-face	18-59	6824	yes	Uzbekistan DHS 2002
Uzbekistan	2014	national	GPAQ	face-to-face	18-64	3643	yes	Uzbekistan STEPS 2014
Uzbekistan	2019	national	GPAQ	face-to-face	18-69	3544	yes	Uzbekistan STEPS 2019
Vanuatu	2011	national	GPAQ	face-to-face	25-64	4457	yes	Vanuatu STEPS 2011
Venezuela (Bolivarian Republic of)	2014-2017	national	IPAQ	face-to-face	20+	3114	no	Venezuela EVESCAM 2014-2017
Viet Nam	2003	national	IPAQ	face-to-face	18+	4146	yes	Viet Nam WHS 2003
Viet Nam	2009	national	GPAQ	face-to-face	25-64	14220	yes	Viet Nam STEPS 2009
Viet Nam	2015	national	GPAQ	face-to-face	18-69	3653	yes	Viet Nam STEPS 2015
Viet Nam	2021	national	GPAQ	face-to-face	18+	4489	yes	Viet Nam STEPS 2021
Zambia	2003	national	IPAQ	face-to-face	18+	4049	yes	Zambia WHS 2003
Zambia	2017	national	GPAQ	face-to-face	18-69	3584	yes	Zambia STEPS 2017
Zimbabwe	2003	national	IPAQ	face-to-face	18+	3970	yes	Zimbabwe WHS 2003

Appendix 10. Country prevalence of insufficient physical activity (95% uncertainty interval) in 2022

Notes: Countries/territories marked with an asterisk indicate no eligible survey data identified.

	Both sexes		Men		Women	
Country	Crude	Age-standardised	Crude	Age-standardised	Crude	Age-standardised
Afghanistan	31.0% (20.7%-41.8%)	33.4% (23.0%-44.2%)	17.6% (8.1%-30.1%)	20.0% (9.9%-33.1%)	44.2% (26.6%-62.3%)	46.0% (28.5%-63.8%)
Albania*	26.5% (10.5%-48.3%)	24.3% (9.0%-45.9%)	23.6% (6.6%-51.2%)	21.4% (5.6%-48.5%)	29.5% (6.2%-64.3%)	27.1% (5.1%-61.9%)
Algeria	28.5% (18.5%-39.3%)	29.0% (19.0%-39.9%)	19.6% (9.8%-32.7%)	20.1% (10.1%-33.2%)	37.6% (20.8%-56.6%)	38.2% (21.4%-57.2%)
Andorra	29.2% (13.7%-49.0%)	27.2% (12.3%-46.6%)	28.0% (9.7%-53.0%)	26.2% (8.7%-50.7%)	30.4% (8.0%-63.0%)	28.3% (6.9%-60.8%)
Angola*	15.4% (4.5%-33.8%)	17.3% (5.5%-36.4%)	11.8% (2.2%-32.4%)	13.9% (3.0%-36.0%)	18.7% (2.5%-50.1%)	20.4% (3.1%-52.4%)
Antigua and Barbuda*	39.0% (18.5%-62.0%)	38.1% (17.8%-61.0%)	31.4% (10.5%-61.3%)	30.8% (10.2%-60.6%)	45.8% (13.4%-80.5%)	44.8% (12.7%-79.8%)
Argentina	39.8% (29.7%-50.3%)	38.5% (28.5%-48.9%)	37.5% (24.8%-50.8%)	36.9% (24.3%-50.3%)	42.0% (26.8%-58.0%)	40.1% (24.9%-56.2%)
Armenia	28.4% (17.8%-40.6%)	26.6% (16.3%-38.5%)	28.9% (15.5%-45.1%)	28.2% (15.0%-44.3%)	28.1% (13.6%-46.3%)	25.3% (11.5%-43.3%)
Australia	28.0% (21.7%-34.9%)	25.5% (19.4%-32.2%)	25.9% (17.8%-35.5%)	23.7% (16.0%-33.1%)	30.0% (20.2%-41.1%)	27.2% (17.8%-38.1%)
Austria	22.9% (18.3%-27.8%)	19.8% (15.5%-24.4%)	22.1% (16.2%-29.1%)	19.6% (14.1%-26.2%)	23.6% (17.1%-31.2%)	19.9% (13.9%-27.0%)
Azerbaijan	23.6% (15.0%-34.2%)	23.9% (15.3%-34.5%)	24.6% (13.2%-38.9%)	25.1% (13.6%-39.4%)	22.7% (10.6%-38.4%)	22.8% (10.7%-38.5%)
Bahamas	37.4% (27.1%-47.9%)	37.2% (26.9%-47.6%)	26.5% (15.4%-39.8%)	26.6% (15.4%-39.8%)	47.1% (31.0%-63.7%)	46.6% (30.6%-63.3%)
Bahrain*	36.9% (17.9%-59.4%)	38.6% (19.4%-60.7%)	34.6% (11.6%-64.3%)	35.7% (12.5%-65.1%)	41.1% (10.2%-76.6%)	42.8% (11.5%-77.8%)
Bangladesh	19.3% (13.4%-26.1%)	20.3% (14.3%-27.2%)	18.5% (11.2%-27.6%)	19.7% (12.1%-28.8%)	20.0% (11.6%-30.8%)	20.9% (12.3%-31.9%)
Barbados	46.7% (27.4%-65.8%)	44.0% (25.1%-63.4%)	38.7% (16.5%-64.2%)	36.3% (14.8%-61.7%)	53.8% (23.3%-82.5%)	51.3% (21.0%-80.9%)
Belarus	16.3% (11.1%-22.6%)	14.0% (9.3%-19.8%)	15.9% (9.3%-24.1%)	14.6% (8.5%-22.4%)	16.6% (9.1%-26.0%)	13.5% (7.0%-22.1%)
Belgium	28.8% (22.3%-36.0%)	25.4% (19.2%-32.3%)	25.0% (16.8%-34.5%)	22.4% (14.7%-31.5%)	32.5% (22.6%-43.9%)	28.4% (18.9%-39.6%)
Belize*	40.2% (19.7%-62.5%)	41.5% (20.8%-63.7%)	33.5% (11.6%-63.1%)	35.2% (12.7%-64.8%)	46.8% (13.9%-81.5%)	47.9% (14.7%-82.1%)
Benin	16.1% (8.4%-26.2%)	18.0% (9.8%-28.7%)	13.4% (5.2%-25.7%)	15.6% (6.5%-28.8%)	18.7% (7.3%-35.7%)	20.3% (8.3%-37.8%)
Bhutan	9.1% (5.3%-14.0%)	9.9% (5.9%-15.0%)	8.0% (3.7%-14.2%)	8.9% (4.2%-15.4%)	10.4% (4.6%-18.1%)	11.0% (5.0%-19.0%)
Bolivia (Plurinational State of)	21.3% (13.6%-30.4%)	22.6% (14.7%-31.9%)	16.8% (8.5%-27.4%)	18.3% (9.6%-29.4%)	25.8% (13.5%-40.9%)	26.6% (14.2%-41.8%)
Bosnia and Herzegovina	23.8% (9.3%-43.7%)	21.1% (7.7%-40.1%)	20.6% (5.5%-43.7%)	18.4% (4.6%-40.6%)	26.8% (5.7%-60.8%)	23.7% (4.4%-57.1%)
Botswana	20.2% (10.8%-31.7%)	22.3% (12.4%-34.1%)	15.1% (6.0%-28.2%)	17.3% (7.3%-31.1%)	25.0% (10.2%-45.1%)	26.8% (11.5%-47.0%)
Brazil	40.9% (31.9%-50.3%)	40.4% (31.4%-49.8%)	35.7% (24.6%-48.0%)	35.6% (24.4%-47.8%)	45.8% (32.4%-59.9%)	45.1% (31.6%-59.2%)
Brunei Darussalam	31.0% (19.4%-44.2%)	32.5% (20.8%-45.7%)	26.2% (12.7%-43.2%)	27.8% (13.9%-44.8%)	36.1% (18.1%-56.6%)	37.4% (19.3%-57.8%)
Bulgaria	37.0% (28.9%-45.5%)	32.3% (24.6%-40.5%)	36.3% (25.5%-48.0%)	32.4% (22.1%-43.9%)	37.7% (25.9%-50.1%)	32.1% (21.0%-44.3%)
Burkina Faso	14.7% (9.5%-20.6%)	16.9% (11.4%-23.3%)	11.8% (6.4%-18.8%)	14.3% (8.1%-22.1%)	17.4% (9.4%-27.7%)	19.3% (10.8%-30.0%)
Burundi*	12.4% (3.2%-27.8%)	14.3% (4.1%-30.7%)	11.7% (2.1%-31.9%)	14.0% (2.9%-35.5%)	13.0% (1.2%-38.7%)	14.5% (1.6%-41.3%)
Cabo Verde	29.8% (20.9%-39.5%)	31.2% (22.2%-41.1%)	20.3% (11.1%-32.3%)	22.4% (12.6%-34.7%)	39.0% (24.5%-55.2%)	39.5% (24.9%-55.7%)
Cambodia	12.4% (8.2%-17.7%)	13.3% (8.9%-18.7%)	11.6% (6.3%-18.5%)	12.6% (7.0%-19.8%)	13.1% (7.1%-21.4%)	13.7% (7.6%-22.0%)
Cameroon*	16.7% (5.1%-34.8%)	18.8% (6.2%-37.5%)	13.8% (2.8%-35.9%)	16.1% (3.7%-39.5%)	19.5% (2.6%-50.9%)	21.4% (3.2%-53.2%)
Canada	40.4% (33.1%-48.0%)	37.2% (30.0%-44.8%)	38.5% (28.6%-48.8%)	35.8% (26.1%-45.9%)	42.2% (31.1%-53.1%)	38.6% (27.7%-49.7%)
Central African Republic*	14.5% (4.2%-31.7%)	16.5% (5.2%-34.5%)	11.5% (2.2%-31.9%)	13.8% (3.1%-36.0%)	17.5% (2.1%-47.0%)	19.1% (2.6%-49.1%)
Chad	15.5% (5.3%-31.8%)	17.8% (6.6%-34.8%)	13.8% (3.0%-33.9%)	16.5% (4.1%-37.9%)	17.2% (2.5%-45.3%)	19.1% (3.2%-48.0%)
Chile	39.8% (30.0%-50.2%)	38.2% (28.5%-48.6%)	31.7% (20.2%-44.7%)	30.4% (19.1%-43.3%)	47.7% (32.0%-63.8%)	45.9% (30.3%-62.3%)
China	23.3% (17.2%-30.1%)	23.8% (17.7%-30.8%)	27.4% (18.3%-37.9%)	28.0% (18.7%-38.6%)	19.1% (11.4%-28.2%)	19.5% (11.6%-28.8%)
Colombia*	34.5% (14.9%-57.2%)	34.4% (14.8%-57.1%)	27.9% (8.0%-57.4%)	28.1% (8.2%-57.6%)	40.9% (10.2%-76.6%)	40.5% (10.0%-76.3%)
Comoros	15.9% (6.8%-29.4%)	17.2% (7.6%-31.0%)	9.3% (2.6%-22.1%)	10.5% (3.1%-24.2%)	22.5% (6.9%-46.6%)	23.7% (7.7%-48.0%)
Congo*	14.2% (4.0%-31.1%)	16.0% (4.9%-33.4%)	10.5% (1.8%-28.8%)	12.3% (2.4%-31.8%)	17.8% (2.1%-48.5%)	19.5% (2.7%-50.7%)

	Both sexes		Men		Women	
Country	Crude	Age-standardised	Crude	Age-standardised	Crude	Age-standardised
Cook Islands	26.6% (19.2%-35.0%)	25.4% (18.2%-33.7%)	17.6% (10.2%-27.1%)	16.3% (9.3%-25.4%)	34.3% (22.1%-47.8%)	33.1% (21.1%-46.6%)
Costa Rica	50.5% (35.8%-64.9%)	49.7% (34.9%-64.1%)	42.3% (25.3%-61.2%)	41.6% (24.7%-60.5%)	58.6% (35.9%-78.8%)	57.7% (34.9%-78.1%)
Croatia	33.0% (25.5%-41.3%)	28.4% (21.3%-36.5%)	31.9% (21.8%-43.0%)	28.3% (18.7%-39.1%)	34.1% (23.4%-46.2%)	28.6% (18.6%-40.5%)
Cuba	63.8% (53.5%-73.4%)	61.1% (50.5%-70.9%)	53.1% (38.5%-67.8%)	50.2% (35.7%-65.2%)	74.1% (59.4%-85.9%)	72.0% (56.7%-84.5%)
Cyprus	43.0% (34.2%-51.9%)	40.8% (32.1%-49.8%)	36.5% (25.3%-48.4%)	34.8% (23.8%-46.5%)	49.4% (35.7%-63.2%)	47.0% (33.3%-61.1%)
Czechia	27.1% (20.3%-34.4%)	23.4% (17.0%-30.3%)	25.6% (16.9%-35.8%)	22.6% (14.5%-32.3%)	28.6% (18.8%-39.6%)	24.1% (15.1%-34.6%)
Côte d'Ivoire	17.4% (5.6%-35.0%)	19.7% (6.9%-37.6%)	13.4% (2.8%-32.7%)	15.8% (3.8%-36.3%)	21.5% (3.4%-53.1%)	23.7% (4.3%-55.7%)
Democratic People's Republic of Korea*	28.1% (12.0%-49.0%)	27.0% (11.2%-47.8%)	24.4% (6.8%-52.8%)	24.4% (6.7%-52.6%)	31.7% (7.1%-65.6%)	29.4% (5.8%-63.6%)
Democratic Republic of the Congo*	13.2% (3.5%-28.6%)	14.9% (4.3%-31.0%)	10.3% (1.8%-28.4%)	12.1% (2.4%-31.7%)	16.1% (1.8%-43.5%)	17.5% (2.3%-45.6%)
Denmark	14.5% (10.1%-19.7%)	12.1% (8.2%-16.9%)	15.1% (8.9%-22.8%)	13.1% (7.5%-20.1%)	13.9% (7.9%-21.9%)	11.2% (6.0%-18.5%)
Djibouti*	15.6% (4.5%-33.2%)	17.0% (5.2%-34.9%)	12.1% (2.4%-32.6%)	13.6% (3.0%-34.9%)	19.0% (2.4%-49.8%)	20.2% (2.8%-51.3%)
Dominica	30.8% (14.6%-49.5%)	30.5% (14.4%-49.2%)	21.5% (7.0%-42.9%)	21.4% (7.0%-42.7%)	40.0% (13.1%-71.2%)	39.4% (12.7%-70.7%)
Dominican Republic*	36.8% (17.0%-59.6%)	37.0% (17.2%-59.9%)	30.5% (9.5%-61.2%)	31.1% (9.9%-61.9%)	42.9% (11.9%-79.7%)	42.9% (11.8%-79.7%)
Ecuador	22.5% (13.9%-32.5%)	22.8% (14.1%-32.8%)	17.0% (8.3%-28.4%)	17.6% (8.6%-29.1%)	27.9% (14.7%-44.5%)	27.8% (14.6%-44.4%)
Egypt	32.4% (22.3%-43.8%)	33.8% (23.6%-45.3%)	28.4% (16.1%-42.8%)	30.0% (17.4%-44.4%)	36.3% (20.4%-54.4%)	37.4% (21.4%-55.3%)
El Salvador	38.9% (25.6%-52.8%)	39.2% (25.8%-53.2%)	30.4% (15.6%-47.6%)	31.8% (16.6%-49.2%)	46.1% (25.3%-67.4%)	45.7% (24.9%-67.1%)
Equatorial Guinea*	17.6% (5.6%-36.1%)	19.5% (6.7%-38.6%)	13.3% (2.7%-34.9%)	15.3% (3.5%-37.8%)	22.6% (3.7%-56.0%)	24.3% (4.4%-58.0%)
Eritrea	10.7% (3.7%-22.3%)	11.8% (4.3%-23.9%)	5.9% (1.4%-14.9%)	7.0% (1.8%-17.0%)	15.2% (3.3%-36.6%)	16.1% (3.7%-38.0%)
Estonia	19.5% (14.7%-24.8%)	15.9% (11.7%-20.7%)	18.7% (12.3%-26.0%)	16.5% (10.6%-23.4%)	20.2% (13.5%-27.8%)	15.4% (9.7%-22.2%)
Eswatini	17.3% (8.7%-28.4%)	19.2% (10.1%-30.7%)	13.2% (4.8%-25.4%)	15.4% (6.0%-28.5%)	21.3% (7.7%-40.1%)	22.6% (8.5%-41.7%)
Ethiopia	8.0% (3.8%-14.3%)	9.4% (4.6%-16.2%)	5.3% (1.7%-11.2%)	6.6% (2.3%-13.4%)	10.8% (3.6%-22.1%)	12.1% (4.3%-24.0%)
Fiji	14.9% (5.9%-27.8%)	15.5% (6.3%-28.6%)	8.3% (2.1%-20.3%)	9.0% (2.4%-21.4%)	21.3% (5.9%-45.4%)	21.8% (6.2%-46.0%)
Finland	12.0% (8.1%-16.8%)	9.6% (6.2%-13.8%)	13.2% (7.6%-20.3%)	11.1% (6.2%-17.5%)	10.9% (5.8%-17.9%)	8.1% (4.0%-14.1%)
France	27.1% (20.2%-34.6%)	23.2% (16.7%-30.3%)	22.8% (14.5%-32.2%)	19.8% (12.2%-28.7%)	31.0% (20.6%-42.6%)	26.3% (16.5%-37.5%)
Gabon*	19.2% (6.2%-38.2%)	20.8% (7.1%-40.2%)	15.4% (3.2%-38.1%)	17.1% (3.9%-40.6%)	23.1% (3.6%-56.4%)	24.6% (4.2%-58.1%)
Gambia	18.8% (8.3%-32.7%)	21.2% (9.9%-35.6%)	14.5% (4.4%-30.8%)	17.0% (5.6%-34.4%)	23.0% (6.9%-47.6%)	25.1% (8.2%-50.0%)
Georgia	26.5% (17.3%-37.4%)	24.0% (15.3%-34.6%)	25.3% (13.4%-40.1%)	24.2% (12.6%-38.7%)	27.5% (14.8%-43.3%)	23.8% (11.9%-39.1%)
Germany	15.0% (11.8%-18.6%)	12.0% (9.2%-15.1%)	14.8% (10.6%-19.7%)	12.4% (8.7%-16.8%)	15.2% (10.7%-20.5%)	11.6% (7.8%-16.2%)
Ghana	19.2% (10.4%-30.5%)	21.2% (11.9%-32.8%)	16.0% (6.6%-30.0%)	18.2% (7.9%-32.8%)	22.3% (8.7%-41.1%)	24.0% (9.8%-43.0%)
Greece	39.8% (31.5%-48.3%)	35.2% (27.2%-43.7%)	37.2% (26.4%-48.4%)	33.1% (22.8%-44.1%)	42.2% (30.2%-54.9%)	37.3% (25.6%-50.2%)
Grenada	36.6% (21.4%-53.2%)	35.9% (20.7%-52.5%)	29.2% (12.7%-50.7%)	28.8% (12.5%-50.3%)	44.0% (19.4%-71.2%)	42.9% (18.5%-70.4%)
Guatemala	35.2% (22.4%-48.9%)	36.8% (23.9%-50.7%)	26.1% (12.6%-43.4%)	28.4% (14.3%-46.2%)	43.8% (23.6%-65.4%)	44.8% (24.5%-66.2%)
Guinea*	14.9% (4.2%-31.9%)	16.7% (5.0%-34.4%)	11.6% (2.0%-31.4%)	13.7% (2.7%-34.9%)	18.0% (2.3%-47.1%)	19.4% (2.7%-48.9%)
Guinea-Bissau*	15.5% (4.5%-33.1%)	17.6% (5.6%-36.1%)	12.7% (2.6%-33.8%)	15.2% (3.6%-37.7%)	18.2% (2.1%-49.0%)	19.8% (2.7%-51.2%)
Guyana	34.3% (22.6%-46.7%)	35.1% (23.2%-47.5%)	23.1% (11.3%-37.8%)	24.2% (12.1%-39.2%)	44.7% (25.8%-65.1%)	45.1% (26.1%-65.4%)
Haiti*	26.8% (9.8%-47.9%)	28.3% (10.8%-49.5%)	18.6% (4.6%-44.8%)	20.3% (5.4%-47.2%)	34.8% (7.5%-70.0%)	35.8% (8.1%-70.9%)
Honduras*	35.0% (14.7%-57.3%)	36.8% (16.1%-59.1%)	28.3% (8.4%-57.7%)	30.6% (9.7%-60.3%)	41.6% (10.7%-77.4%)	42.9% (11.6%-78.3%)
Hungary	33.1% (25.5%-41.2%)	29.4% (22.2%-37.3%)	30.6% (20.9%-41.5%)	28.1% (18.8%-38.7%)	35.3% (24.3%-47.1%)	30.6% (20.2%-42.3%)
Iceland*	27.7% (11.0%-48.7%)	25.9% (9.8%-46.7%)	25.4% (6.9%-53.0%)	23.9% (6.2%-51.1%)	30.1% (6.2%-65.6%)	28.0% (5.2%-63.5%)
India	48.7% (37.6%-60.3%)	49.4% (38.4%-61.0%)	41.0% (26.1%-56.8%)	42.0% (27.2%-57.8%)	56.8% (39.6%-73.4%)	57.2% (40.1%-73.7%)
Indonesia	18.3% (11.4%-26.3%)	19.0% (12.0%-27.1%)	20.5% (10.9%-32.5%)	21.5% (11.7%-33.7%)	16.1% (7.4%-27.9%)	16.4% (7.7%-28.3%)
Iran (Islamic Republic of)	45.9% (37.4%-54.1%)	46.3% (37.8%-54.4%)	35.8% (25.5%-47.4%)	35.9% (25.7%-47.5%)	56.2% (43.3%-68.4%)	56.6% (43.8%-68.8%)
Iraq	49.8% (36.2%-63.5%)	52.0% (38.5%-65.5%)	39.1% (21.7%-57.8%)	41.9% (24.1%-60.5%)	60.1% (39.4%-79.9%)	61.5% (41.1%-80.8%)

	Both sexes		Men		Women	
Country	Crude	Age-standardised	Crude	Age-standardised	Crude	Age-standardised
Ireland	23.9% (18.4%-29.7%)	21.9% (16.6%-27.6%)	21.4% (14.8%-29.0%)	19.7% (13.4%-27.0%)	26.3% (18.2%-35.2%)	24.1% (16.2%-32.7%)
Israel*	27.9% (11.4%-49.1%)	26.6% (10.4%-47.6%)	25.0% (6.7%-53.2%)	24.1% (6.3%-52.2%)	30.7% (6.7%-65.6%)	29.0% (5.8%-63.9%)
Italy	45.2% (36.7%-53.8%)	40.1% (31.8%-48.8%)	40.7% (29.5%-52.7%)	36.4% (25.6%-48.4%)	49.4% (37.1%-61.9%)	43.8% (31.5%-56.7%)
Jamaica	38.2% (20.0%-57.0%)	38.6% (20.4%-57.3%)	30.5% (11.0%-55.7%)	31.3% (11.5%-56.4%)	45.5% (16.5%-76.2%)	45.7% (16.7%-76.3%)
Japan	50.6% (39.5%-61.4%)	44.7% (33.7%-55.8%)	44.6% (30.3%-59.5%)	40.0% (26.1%-54.7%)	56.1% (39.9%-71.3%)	49.5% (33.0%-65.9%)
Jordan	26.4% (17.7%-36.3%)	28.2% (19.3%-38.2%)	28.2% (16.3%-41.7%)	30.0% (17.9%-43.7%)	24.5% (12.8%-39.0%)	26.1% (14.1%-40.7%)
Kazakhstan	28.4% (12.6%-49.1%)	28.2% (12.5%-48.7%)	28.3% (8.8%-54.5%)	28.7% (9.0%-54.8%)	28.5% (6.4%-61.2%)	27.7% (6.0%-60.4%)
Kenya	7.5% (3.5%-13.3%)	8.9% (4.4%-15.3%)	6.9% (2.3%-14.3%)	8.5% (3.1%-16.8%)	8.1% (2.4%-17.6%)	9.3% (3.0%-19.5%)
Kiribati	30.3% (18.7%-42.5%)	31.9% (20.1%-44.2%)	19.3% (8.3%-34.4%)	21.2% (9.7%-36.8%)	40.1% (21.7%-60.2%)	41.1% (22.7%-61.1%)
Kuwait	63.1% (44.7%-79.8%)	63.3% (45.3%-79.7%)	62.8% (38.1%-83.6%)	61.7% (37.3%-82.7%)	63.6% (33.9%-87.7%)	65.1% (35.9%-88.4%)
Kyrgyzstan	18.6% (9.4%-30.3%)	19.9% (10.3%-31.7%)	16.0% (5.8%-30.9%)	17.3% (6.5%-32.7%)	21.1% (7.4%-40.7%)	22.0% (8.0%-41.8%)
Lao People's Democratic Republic	14.6% (6.8%-25.1%)	15.8% (7.7%-26.5%)	8.2% (2.2%-17.9%)	9.1% (2.7%-19.2%)	21.0% (7.7%-40.2%)	22.4% (8.7%-41.8%)
Latvia	18.2% (12.6%-24.6%)	14.5% (9.7%-20.1%)	17.7% (10.3%-26.1%)	15.4% (8.7%-23.2%)	18.5% (10.9%-28.1%)	13.7% (7.4%-22.1%)
Lebanon	59.6% (47.0%-71.6%)	58.6% (46.0%-70.8%)	58.3% (41.3%-74.3%)	57.8% (40.8%-73.9%)	60.7% (42.1%-77.9%)	59.4% (40.6%-77.1%)
Lesotho	7.0% (2.7%-14.3%)	8.0% (3.2%-15.8%)	7.2% (1.9%-16.8%)	8.6% (2.5%-19.2%)	6.8% (1.2%-18.3%)	7.4% (1.4%-19.4%)
Liberia	12.9% (8.3%-18.6%)	14.6% (9.6%-20.7%)	11.6% (6.0%-19.2%)	13.6% (7.4%-21.9%)	14.1% (7.4%-23.3%)	15.5% (8.5%-25.1%)
Libya	44.3% (35.3%-53.3%)	45.6% (36.5%-54.5%)	36.2% (25.1%-48.7%)	37.5% (26.3%-49.9%)	52.5% (38.7%-65.8%)	53.6% (39.9%-66.8%)
Lithuania	24.3% (18.1%-30.8%)	20.2% (14.7%-26.2%)	23.3% (15.5%-32.3%)	20.7% (13.5%-29.4%)	25.1% (16.6%-35.1%)	19.8% (12.2%-29.1%)
Luxembourg	15.5% (10.9%-20.9%)	13.9% (9.7%-19.0%)	15.1% (9.2%-22.4%)	13.9% (8.3%-20.8%)	15.8% (9.5%-23.7%)	13.9% (8.0%-21.3%)
Madagascar*	12.1% (3.4%-27.5%)	13.6% (4.2%-29.8%)	10.8% (2.1%-30.7%)	12.5% (2.7%-33.8%)	13.3% (1.3%-40.2%)	14.6% (1.6%-42.2%)
Malawi	2.1% (0.8%-4.2%)	2.7% (1.1%-5.3%)	1.7% (0.5%-4.1%)	2.4% (0.7%-5.4%)	2.4% (0.6%-6.1%)	2.9% (0.8%-7.1%)
Malaysia	39.2% (29.9%-48.8%)	39.9% (30.7%-49.5%)	36.5% (24.7%-49.0%)	37.4% (25.6%-49.9%)	41.9% (28.3%-55.9%)	42.4% (28.8%-56.3%)
Maldives	22.6% (15.2%-31.4%)	24.6% (16.9%-33.6%)	23.2% (13.1%-35.4%)	25.3% (14.7%-37.7%)	21.7% (11.5%-34.3%)	23.5% (12.8%-36.4%)
Mali	27.1% (11.4%-47.8%)	29.8% (13.4%-50.8%)	22.7% (6.2%-47.0%)	25.9% (7.9%-50.8%)	31.5% (7.1%-65.7%)	33.8% (8.4%-67.7%)
Malta	43.7% (33.4%-54.2%)	40.7% (30.5%-51.3%)	40.0% (26.5%-54.5%)	37.8% (24.5%-52.2%)	47.7% (32.0%-64.4%)	44.1% (28.4%-61.2%)
Marshall Islands	23.3% (8.2%-43.4%)	24.4% (9.0%-44.7%)	15.9% (2.8%-38.9%)	16.9% (3.2%-40.0%)	30.7% (5.8%-66.1%)	32.0% (6.5%-67.2%)
Mauritania	36.2% (16.2%-58.8%)	38.5% (18.1%-61.0%)	27.8% (7.4%-55.0%)	30.5% (8.8%-58.0%)	43.9% (11.5%-80.2%)	45.8% (12.9%-81.3%)
Mauritius	31.5% (23.0%-40.8%)	29.9% (21.6%-39.0%)	26.0% (15.8%-38.7%)	24.9% (15.0%-37.3%)	36.8% (23.3%-51.3%)	34.7% (21.4%-49.1%)
Mexico	28.0% (22.1%-34.9%)	28.0% (22.1%-34.8%)	25.1% (17.5%-33.7%)	25.3% (17.7%-33.9%)	30.7% (21.6%-41.2%)	30.5% (21.4%-41.0%)
Micronesia (Federated States of)	28.5% (15.1%-44.6%)	29.3% (15.8%-45.4%)	21.6% (7.8%-41.4%)	22.9% (8.6%-43.0%)	35.2% (13.7%-60.9%)	35.6% (14.0%-61.3%)
Monaco*	33.4% (15.3%-55.2%)	25.5% (9.8%-46.6%)	30.3% (9.9%-59.2%)	23.3% (6.1%-51.0%)	36.4% (9.3%-70.3%)	27.8% (4.9%-62.1%)
Mongolia	26.3% (18.1%-35.8%)	27.5% (19.2%-37.0%)	24.6% (14.7%-36.9%)	25.8% (15.7%-38.2%)	27.8% (16.0%-42.4%)	28.8% (16.8%-43.4%)
Montenegro*	25.1% (9.6%-46.4%)	22.8% (8.1%-43.5%)	23.2% (6.1%-50.5%)	21.6% (5.3%-48.2%)	26.9% (5.0%-61.6%)	23.9% (3.8%-58.2%)
Morocco	24.2% (15.0%-34.9%)	24.5% (15.3%-35.2%)	19.5% (9.9%-32.2%)	19.8% (10.1%-32.5%)	28.9% (14.7%-45.6%)	29.1% (14.8%-45.7%)
Mozambique	8.2% (2.1%-19.5%)	9.7% (2.7%-22.1%)	6.5% (1.0%-19.0%)	8.2% (1.6%-22.3%)	9.7% (1.0%-28.9%)	11.0% (1.3%-31.1%)
Myanmar	21.1% (12.0%-32.6%)	22.0% (12.8%-33.5%)	19.1% (8.4%-33.9%)	20.1% (9.2%-35.1%)	23.1% (9.3%-41.3%)	23.6% (9.8%-41.9%)
Namibia	23.5% (8.7%-42.8%)	25.4% (10.0%-45.1%)	18.6% (4.3%-40.9%)	21.0% (5.5%-44.1%)	27.9% (5.8%-62.4%)	29.1% (6.5%-63.6%)
Nauru	33.2% (21.2%-46.4%)	35.5% (23.3%-48.7%)	25.1% (11.3%-42.5%)	27.8% (13.4%-45.6%)	41.2% (21.3%-62.9%)	42.8% (22.7%-64.3%)
Nepal	7.7% (4.4%-12.0%)	8.2% (4.9%-12.7%)	9.7% (4.7%-16.8%)	10.4% (5.1%-17.7%)	5.9% (2.4%-11.5%)	6.3% (2.7%-12.1%)
Netherlands (Kingdom of)	11.4% (8.6%-14.7%)	9.4% (6.9%-12.4%)	9.6% (6.2%-13.8%)	8.1% (5.1%-11.9%)	13.2% (8.6%-18.8%)	10.7% (6.7%-15.7%)
New Zealand	20.7% (16.0%-25.9%)	18.7% (14.2%-23.6%)	19.6% (13.6%-26.6%)	17.8% (12.2%-24.6%)	21.9% (15.0%-30.2%)	19.5% (13.0%-27.5%)
Nicaragua*	37.4% (17.0%-60.8%)	38.7% (18.0%-62.1%)	31.0% (9.8%-60.6%)	32.9% (10.9%-62.5%)	43.5% (11.5%-79.0%)	44.3% (12.1%-79.5%)
Niger	14.1% (8.7%-20.8%)	16.2% (10.4%-23.3%)	10.7% (5.2%-18.5%)	13.0% (6.7%-21.6%)	17.5% (9.1%-29.3%)	19.3% (10.4%-31.6%)
Nigeria*	16.2% (4.9%-34.5%)	18.1% (6.0%-36.9%)	13.5% (2.5%-34.1%)	15.5% (3.3%-37.4%)	18.9% (2.3%-51.6%)	20.6% (2.9%-53.7%)

	Both sexes		Men		Women	
Country	Crude	Age-standardised	Crude	Age-standardised	Crude	Age-standardised
Niue	8.6% (3.1%-18.2%)	7.4% (2.4%-16.2%)	7.4% (1.6%-18.8%)	6.3% (1.3%-16.7%)	9.7% (1.9%-25.5%)	8.1% (1.4%-22.7%)
North Macedonia	28.0% (14.2%-44.2%)	26.1% (12.9%-42.0%)	21.8% (7.6%-41.6%)	20.5% (6.9%-39.7%)	34.0% (12.7%-61.5%)	31.6% (11.1%-59.1%)
Norway	38.1% (22.5%-55.5%)	35.1% (20.0%-52.5%)	34.4% (16.2%-56.6%)	31.8% (14.4%-53.9%)	41.8% (18.6%-68.2%)	38.5% (15.8%-65.3%)
occupied Palestinian territory, including east Jerusalem	27.6% (20.0%-36.6%)	29.7% (21.9%-38.8%)	23.7% (14.6%-34.7%)	26.1% (16.5%-37.3%)	31.4% (19.7%-45.1%)	33.1% (21.1%-46.8%)
Oman	38.0% (27.1%-50.2%)	40.9% (30.0%-52.9%)	32.6% (19.1%-48.4%)	35.0% (21.2%-50.8%)	48.1% (30.1%-67.5%)	50.4% (32.5%-69.4%)
Pakistan	44.2% (30.0%-58.8%)	45.7% (31.6%-60.0%)	32.3% (16.3%-50.9%)	34.1% (17.9%-52.7%)	56.0% (34.0%-77.3%)	57.0% (35.2%-77.9%)
Palau	26.4% (14.9%-39.8%)	26.0% (14.6%-39.2%)	16.8% (6.8%-32.3%)	16.6% (6.8%-31.9%)	36.8% (16.7%-59.6%)	36.0% (16.1%-58.8%)
Panama	58.0% (46.9%-68.3%)	57.6% (46.6%-68.0%)	48.4% (33.6%-63.5%)	48.3% (33.5%-63.4%)	67.3% (51.0%-81.3%)	66.9% (50.4%-81.0%)
Papua New Guinea	11.3% (3.2%-24.6%)	12.5% (3.8%-26.2%)	7.1% (1.2%-20.8%)	8.2% (1.5%-22.9%)	15.8% (2.5%-42.2%)	17.0% (2.9%-44.0%)
Paraguay	35.1% (16.8%-56.4%)	36.0% (17.4%-57.3%)	30.8% (10.6%-57.5%)	32.1% (11.4%-58.9%)	39.3% (11.1%-73.2%)	39.8% (11.4%-73.5%)
Peru	34.5% (23.0%-46.3%)	34.5% (23.0%-46.2%)	31.8% (17.8%-47.2%)	32.0% (18.0%-47.4%)	37.1% (19.9%-56.0%)	36.9% (19.7%-55.8%)
Philippines	44.4% (32.4%-56.1%)	45.5% (33.5%-57.0%)	34.6% (20.1%-51.4%)	35.8% (21.3%-52.5%)	54.4% (35.4%-72.8%)	55.0% (36.2%-73.2%)
Poland	40.4% (32.1%-48.9%)	37.0% (28.9%-45.4%)	38.5% (27.3%-50.4%)	36.0% (25.1%-47.8%)	42.1% (29.9%-54.6%)	38.0% (25.9%-50.5%)
Portugal	56.1% (47.2%-64.6%)	51.7% (42.6%-60.5%)	49.8% (37.8%-62.2%)	46.0% (34.1%-58.7%)	61.6% (49.1%-73.6%)	56.9% (43.9%-69.8%)
Puerto Rico*	45.9% (23.9%-68.2%)	41.6% (20.1%-64.3%)	40.9% (16.7%-69.9%)	36.6% (13.7%-66.0%)	50.3% (16.4%-83.4%)	46.0% (13.1%-80.8%)
Qatar	51.1% (32.1%-70.3%)	53.5% (34.8%-71.9%)	48.3% (24.8%-71.8%)	50.3% (26.9%-73.1%)	60.3% (30.2%-86.3%)	63.2% (33.7%-87.7%)
Republic of Korea	60.7% (55.0%-66.1%)	58.1% (52.4%-63.7%)	58.3% (50.5%-65.6%)	55.9% (48.2%-63.4%)	63.0% (55.0%-70.5%)	60.3% (52.0%-68.1%)
Republic of Moldova	11.6% (7.5%-16.8%)	10.8% (6.9%-15.7%)	12.4% (6.7%-20.0%)	12.1% (6.6%-19.5%)	10.9% (5.4%-18.7%)	9.6% (4.7%-16.8%)
Romania	40.4% (32.1%-49.3%)	36.8% (28.6%-45.6%)	39.8% (28.7%-52.0%)	37.0% (26.2%-49.1%)	41.0% (28.8%-54.1%)	36.6% (24.7%-49.7%)
Russian Federation	20.3% (8.6%-37.2%)	18.1% (7.3%-34.3%)	19.2% (6.2%-40.1%)	18.1% (5.7%-38.3%)	21.2% (5.2%-46.6%)	18.2% (3.9%-42.6%)
Rwanda	7.6% (4.6%-11.5%)	9.0% (5.6%-13.3%)	6.1% (3.0%-10.6%)	7.7% (4.0%-12.9%)	8.9% (4.4%-15.5%)	10.1% (5.2%-17.1%)
Saint Kitts and Nevis	42.4% (22.7%-62.9%)	41.7% (22.1%-62.2%)	34.8% (13.0%-61.1%)	34.1% (12.6%-60.3%)	49.3% (18.4%-80.7%)	48.7% (17.9%-80.3%)
Saint Lucia	21.1% (13.8%-29.3%)	20.9% (13.7%-29.1%)	17.7% (9.3%-27.5%)	17.8% (9.4%-27.6%)	24.4% (13.1%-38.0%)	24.0% (12.8%-37.5%)
Saint Vincent and the Grenadines	31.4% (19.2%-45.0%)	30.5% (18.4%-43.9%)	22.3% (10.0%-38.3%)	21.4% (9.4%-37.1%)	40.9% (20.7%-64.1%)	39.9% (19.9%-63.2%)
Samoa	12.9% (5.7%-22.9%)	13.5% (6.1%-23.6%)	8.7% (2.5%-19.1%)	9.3% (2.8%-20.1%)	17.2% (5.3%-34.9%)	17.6% (5.5%-35.4%)
San Marino*	29.0% (12.2%-49.5%)	25.7% (10.0%-45.5%)	25.8% (7.8%-53.5%)	22.9% (6.4%-49.8%)	31.9% (7.3%-65.1%)	28.3% (5.5%-61.5%)
Saudi Arabia	49.6% (38.9%-60.1%)	51.5% (40.9%-61.8%)	45.7% (31.3%-60.4%)	46.7% (32.3%-61.2%)	55.5% (39.4%-70.4%)	57.8% (41.9%-72.2%)
Senegal	14.7% (7.5%-24.5%)	16.6% (8.8%-26.8%)	9.0% (3.3%-18.3%)	10.9% (4.3%-21.2%)	19.9% (7.8%-37.0%)	21.6% (8.9%-39.0%)
Serbia	48.9% (27.1%-69.3%)	44.9% (23.6%-65.7%)	44.9% (18.8%-70.7%)	41.7% (16.4%-67.9%)	52.4% (20.4%-83.0%)	47.9% (16.6%-80.1%)
Seychelles	20.6% (10.8%-32.4%)	20.4% (10.7%-32.2%)	19.4% (8.1%-35.8%)	19.7% (8.4%-36.1%)	22.0% (8.0%-41.9%)	21.2% (7.6%-40.9%)
Sierra Leone	11.3% (4.0%-22.8%)	13.0% (4.9%-25.0%)	7.0% (1.6%-17.6%)	8.6% (2.1%-20.3%)	15.6% (3.2%-37.4%)	17.1% (3.9%-39.5%)
Singapore	24.6% (18.4%-31.5%)	23.2% (17.2%-29.9%)	25.6% (16.9%-35.7%)	24.2% (15.8%-34.0%)	23.5% (14.8%-33.5%)	22.0% (13.7%-31.8%)
Slovakia	25.8% (18.7%-33.3%)	23.3% (16.5%-30.5%)	24.3% (15.1%-34.9%)	22.5% (13.7%-32.8%)	27.3% (17.3%-38.7%)	24.1% (14.6%-35.1%)
Slovenia	22.7% (16.4%-29.6%)	19.0% (13.3%-25.4%)	21.3% (13.4%-31.0%)	18.4% (11.3%-27.4%)	24.1% (15.1%-34.9%)	19.5% (11.5%-29.7%)
Solomon Islands	16.2% (8.3%-26.4%)	17.5% (9.2%-27.9%)	11.3% (4.1%-22.5%)	12.7% (4.9%-24.7%)	21.1% (8.1%-38.6%)	22.3% (8.8%-39.8%)
Somalia*	16.0% (4.9%-33.6%)	18.1% (6.0%-36.3%)	11.3% (2.0%-31.1%)	13.5% (2.8%-34.8%)	20.7% (2.9%-52.4%)	22.4% (3.6%-54.5%)
South Africa	43.6% (30.6%-57.5%)	44.8% (31.8%-58.7%)	39.0% (22.4%-57.6%)	41.2% (24.4%-59.7%)	47.7% (28.7%-68.0%)	48.1% (29.0%-68.2%)
South Sudan*	14.6% (4.0%-31.9%)	16.3% (4.8%-34.2%)	11.5% (2.1%-32.9%)	13.3% (2.7%-36.0%)	17.6% (2.1%-48.1%)	18.9% (2.6%-49.9%)
Spain	25.3% (19.3%-31.5%)	21.8% (16.3%-27.7%)	22.1% (14.9%-30.3%)	19.4% (12.7%-27.2%)	28.4% (19.8%-38.1%)	24.2% (16.2%-33.6%)
Sri Lanka	38.5% (29.7%-47.2%)	37.2% (28.5%-45.8%)	29.2% (19.1%-40.8%)	28.7% (18.8%-40.2%)	46.9% (33.5%-60.2%)	44.8% (31.4%-58.3%)
Sudan	17.1% (9.6%-26.8%)	19.1% (11.1%-29.3%)	14.9% (6.5%-27.0%)	17.2% (8.0%-30.0%)	19.3% (8.2%-35.1%)	20.9% (9.3%-37.0%)

	Both sexes		Men		Women	
Country	Crude	Age-standardised	Crude	Age-standardised	Crude	Age-standardised
Suriname	48.7% (33.6%-64.7%)	48.9% (33.8%-64.8%)	41.8% (23.0%-62.3%)	42.4% (23.6%-62.9%)	55.4% (31.2%-78.4%)	55.1% (30.9%-78.2%)
Sweden	10.7% (7.2%-15.3%)	8.7% (5.6%-12.7%)	11.0% (6.2%-17.2%)	9.3% (5.1%-14.9%)	10.4% (5.5%-17.3%)	8.1% (4.0%-14.1%)
Switzerland	21.9% (12.7%-33.3%)	19.0% (10.6%-29.9%)	19.9% (9.0%-35.0%)	17.6% (7.6%-31.9%)	23.8% (10.6%-42.1%)	20.4% (8.3%-38.0%)
Syrian Arab Republic*	36.3% (16.2%-59.2%)	37.8% (17.5%-60.6%)	31.0% (9.4%-60.4%)	33.1% (10.6%-62.5%)	41.4% (10.7%-77.1%)	42.4% (11.4%-77.7%)
São Tomé and Príncipe	16.6% (10.1%-24.6%)	18.2% (11.3%-26.3%)	12.5% (5.9%-21.8%)	14.1% (6.9%-24.1%)	20.7% (10.5%-34.3%)	22.0% (11.4%-35.9%)
Taiwan, China*	29.4% (12.5%-50.8%)	27.4% (11.0%-48.7%)	28.5% (9.0%-57.5%)	27.2% (8.1%-56.1%)	30.3% (6.4%-63.7%)	27.4% (4.9%-61.1%)
Tajikistan	33.8% (22.2%-46.5%)	35.7% (24.0%-48.4%)	24.3% (12.3%-39.3%)	26.3% (13.8%-41.6%)	43.2% (24.1%-63.4%)	45.0% (25.9%-64.9%)
Thailand	31.2% (25.7%-37.0%)	28.9% (23.5%-34.7%)	29.5% (22.4%-37.5%)	28.2% (21.1%-36.1%)	32.7% (24.7%-41.4%)	29.4% (21.6%-38.1%)
Timor-Leste	26.4% (13.3%-42.8%)	26.9% (13.8%-43.2%)	20.4% (6.7%-40.4%)	20.9% (7.1%-40.9%)	32.6% (11.7%-59.7%)	33.0% (12.2%-59.9%)
Togo	13.0% (8.4%-18.4%)	14.8% (9.8%-20.5%)	10.3% (5.4%-17.2%)	12.2% (6.7%-19.6%)	15.6% (8.3%-25.1%)	17.3% (9.5%-27.1%)
Tonga	30.2% (19.8%-41.9%)	30.7% (20.2%-42.4%)	21.8% (11.3%-36.1%)	22.6% (11.8%-37.0%)	38.0% (21.1%-56.2%)	38.1% (21.2%-56.3%)
Trinidad and Tobago	45.7% (28.8%-63.2%)	44.5% (27.8%-62.0%)	35.6% (16.9%-57.4%)	34.6% (16.4%-56.3%)	55.3% (29.2%-80.8%)	54.2% (28.0%-80.0%)
Tunisia	33.8% (16.1%-55.1%)	33.4% (15.9%-54.6%)	29.0% (9.7%-53.6%)	28.6% (9.6%-53.1%)	38.3% (11.2%-73.0%)	37.8% (10.8%-72.6%)
Turkmenistan	11.2% (6.1%-18.0%)	12.1% (6.8%-19.1%)	9.9% (4.0%-18.3%)	10.8% (4.5%-19.5%)	12.4% (5.1%-23.5%)	13.1% (5.5%-24.4%)
Tuvalu	24.1% (13.6%-36.8%)	24.4% (13.9%-37.2%)	15.4% (6.3%-29.1%)	16.2% (6.8%-30.1%)	33.0% (15.2%-54.2%)	32.8% (15.1%-53.9%)
Türkiye	44.5% (32.4%-56.8%)	44.4% (32.4%-56.7%)	34.9% (20.5%-51.0%)	35.1% (20.8%-51.3%)	53.9% (35.5%-71.7%)	53.4% (34.9%-71.3%)
Uganda	4.2% (2.4%-6.5%)	5.5% (3.4%-8.3%)	4.3% (2.0%-7.6%)	6.0% (3.1%-10.1%)	4.1% (1.8%-7.6%)	5.2% (2.4%-9.3%)
Ukraine	15.4% (9.3%-22.9%)	12.8% (7.4%-19.5%)	14.3% (7.0%-24.4%)	12.8% (6.1%-22.2%)	16.3% (7.5%-27.5%)	12.8% (5.4%-22.8%)
United Arab Emirates	64.0% (52.0%-75.2%)	66.1% (54.6%-76.8%)	61.3% (45.5%-75.5%)	63.1% (47.7%-76.9%)	71.4% (55.8%-84.6%)	73.6% (58.8%-86.0%)
United Kingdom	21.9% (17.0%-27.7%)	19.0% (14.4%-24.5%)	20.6% (14.2%-27.9%)	18.2% (12.3%-25.0%)	23.1% (15.6%-31.9%)	19.7% (12.8%-28.0%)
United Republic of Tanzania	3.5% (1.9%-5.7%)	4.3% (2.5%-6.8%)	3.8% (1.7%-6.9%)	4.8% (2.2%-8.5%)	3.2% (1.4%-6.2%)	3.9% (1.7%-7.2%)
United States of America	36.4% (29.1%-44.5%)	33.7% (26.6%-41.8%)	29.7% (20.5%-40.3%)	27.6% (18.7%-37.9%)	42.9% (31.2%-55.9%)	39.8% (28.3%-52.9%)
Uruguay	36.4% (20.8%-54.0%)	33.8% (18.7%-51.3%)	32.3% (13.6%-53.8%)	30.8% (12.5%-52.2%)	40.1% (16.0%-67.6%)	36.8% (13.4%-64.8%)
Uzbekistan	30.4% (22.0%-39.4%)	31.7% (23.2%-40.7%)	24.6% (15.1%-36.6%)	26.0% (16.2%-38.1%)	36.1% (22.8%-50.5%)	37.1% (23.8%-51.4%)
Vanuatu	6.8% (2.3%-15.0%)	7.5% (2.6%-16.0%)	5.1% (1.0%-13.8%)	5.8% (1.3%-15.2%)	8.5% (1.3%-23.3%)	9.2% (1.5%-24.5%)
Venezuela (Bolivarian Republic of)	46.2% (33.0%-59.9%)	45.7% (32.6%-59.4%)	38.4% (22.4%-55.8%)	38.5% (22.5%-55.9%)	53.4% (33.0%-72.5%)	52.8% (32.3%-72.0%)
Viet Nam	30.2% (22.9%-38.0%)	30.3% (23.0%-38.1%)	24.8% (15.8%-35.2%)	25.6% (16.5%-36.1%)	35.2% (24.2%-47.2%)	34.5% (23.4%-46.6%)
Yemen*	26.7% (10.0%-48.2%)	29.2% (11.7%-50.9%)	21.4% (5.5%-48.5%)	24.0% (6.7%-51.9%)	32.1% (6.3%-68.0%)	34.1% (7.4%-69.7%)
Zambia	11.6% (6.1%-18.8%)	14.1% (7.9%-21.9%)	7.4% (2.8%-14.5%)	9.6% (4.0%-17.8%)	15.7% (6.5%-29.0%)	17.9% (8.0%-31.9%)
Zimbabwe	15.7% (4.9%-33.6%)	17.7% (6.0%-36.1%)	11.2% (2.3%-27.8%)	13.3% (3.1%-31.3%)	19.5% (3.0%-50.1%)	21.1% (3.6%-52.2%)

Appendix 11. Map of prevalence of insufficient physical activity in 2022, by country and sex

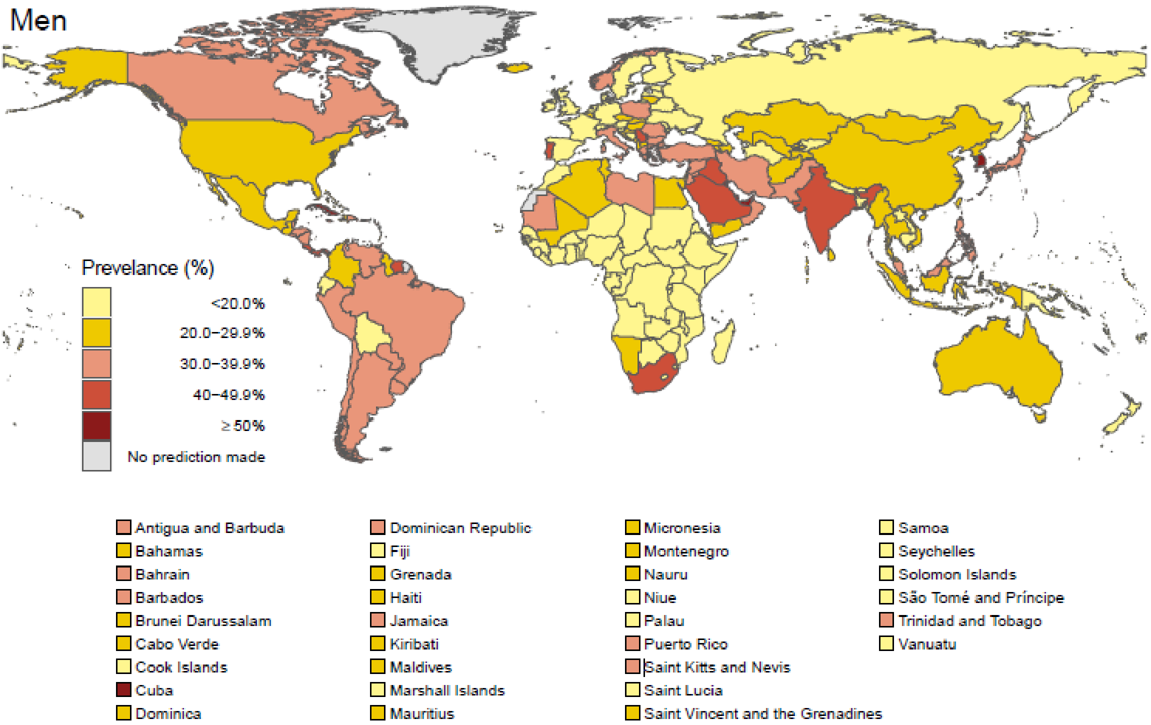

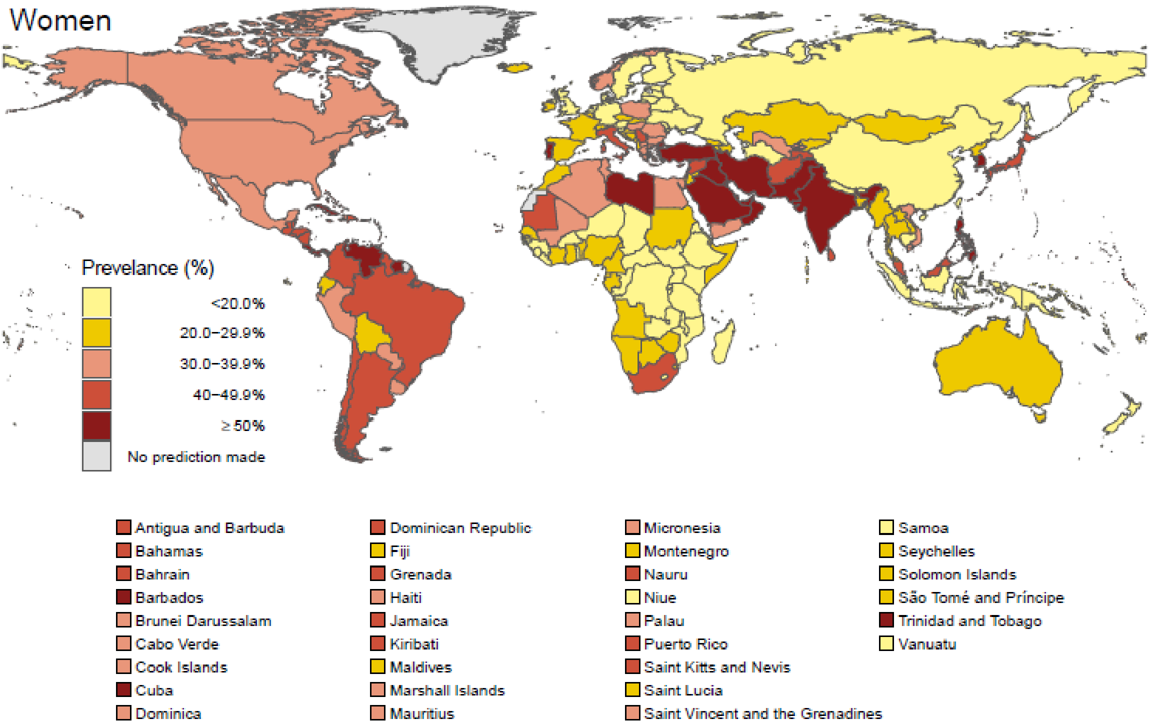

Appendix 12. Scatter plot of male and female age-standardised prevalence of insufficient physical activity in 2022, by income group.

Notes: Countries with survey data included are plotted. Selected countries are labelled as follows: AFG: Afghanistan; ARM: Armenia; AZE: Azerbaijan; CHN: China; CUB: Cuba; GUY: Guyana; IRN: Islamic Republic of Iran; IRQ: Iraq; JOR: Jordan; PAK: Pakistan; PAN: Panama; PHL: Philippines; PLW: Palau.

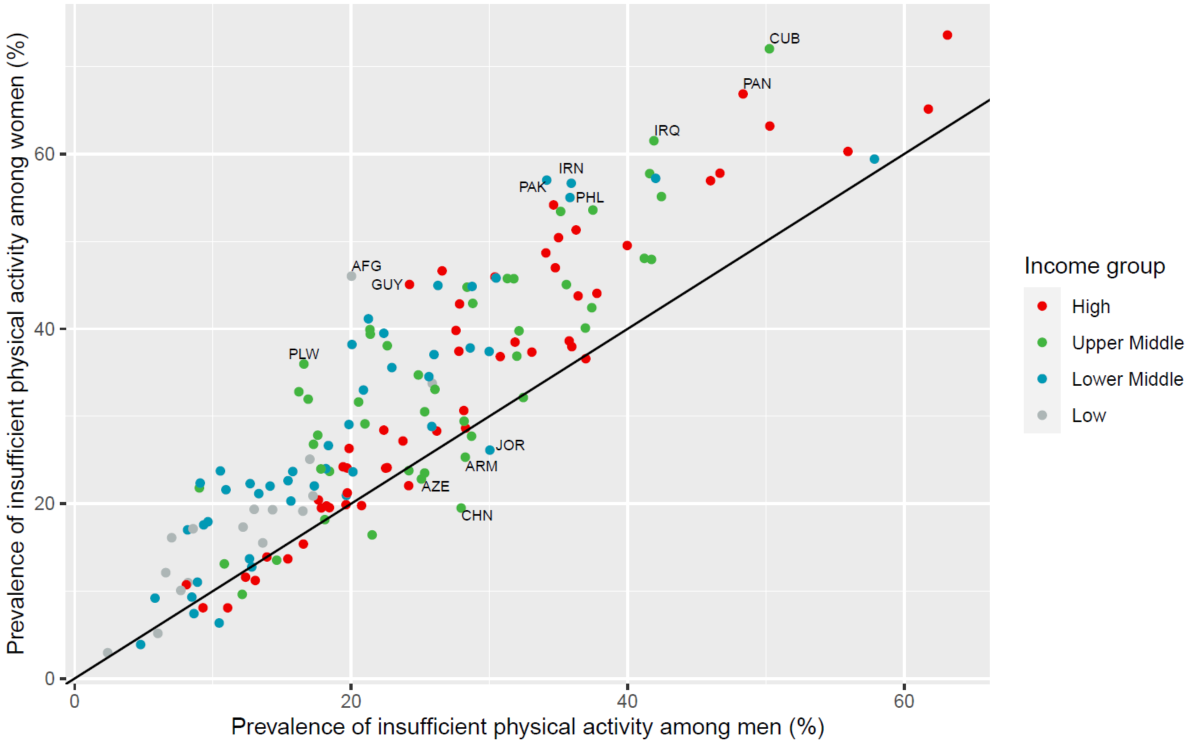

Appendix 13. Prevalence of insufficient physical activity in 2022, by age group

	Both sexes			Men			Women		
	18-39 years	40-59 years	≥60 years	18-39 years	40-59 years	≥60 years	18-39 years	40-59 years	≥60 years
Global	28.6% (25.8%-31.5%)	28.9% (26.3%-31.7%)	43.5% (41.0%-46.2%)	25.3% (21.5%-29.3%)	27.9% (24.1%-31.7%)	40.0% (36.0%-43.8%)	32.1% (27.9%-36.3%)	30.0% (26.3%-33.8%)	46.5% (43.0%-50.2%)
Region									
Central Asia and North Africa-Middle East	33.5% (29.6%-37.5%)	37.7% (33.7%-41.8%)	52.9% (48.7%-57.2%)	27.3% (22.6%-32.1%)	34.6% (29.6%-39.9%)	44.0% (38.4%-49.9%)	40.2% (34.3%-46.7%)	41.1% (35.1%-47.3%)	60.5% (54.5%-66.6%)
Central and Eastern Europe	18.7% (13.6%-26.1%)	22.4% (16.9%-30.1%)	36.6% (29.0%-46.0%)	17.7% (11.9%-26.8%)	23.3% (16.4%-33.3%)	35.9% (27.5%-46.9%)	19.7% (12.5%-31.4%)	21.6% (14.1%-33.6%)	37.0% (26.5%-50.8%)
East and South-East Asia	25.1% (20.6%-30.1%)	19.7% (15.6%-24.3%)	31.4% (26.2%-37.1%)	27.5% (20.7%-34.8%)	23.1% (16.6%-30.3%)	31.7% (24.2%-39.9%)	22.6% (17.0%-29.2%)	16.3% (11.6%-22.0%)	31.1% (24.1%-39.0%)
High-income Asia Pacific	43.7% (36.5%-51.0%)	50.0% (42.2%-57.7%)	61.5% (52.9%-69.5%)	39.1% (30.5%-48.5%)	48.5% (38.6%-58.7%)	55.0% (43.5%-66.1%)	48.7% (37.6%-59.8%)	51.5% (40.1%-62.7%)	66.9% (54.8%-77.4%)
High-income Western countries	24.1% (20.7%-27.8%)	27.3% (23.9%-30.9%)	41.5% (37.8%-45.3%)	20.1% (16.0%-24.7%)	24.7% (20.3%-29.5%)	36.6% (31.6%-41.8%)	28.2% (22.8%-34.4%)	29.8% (24.7%-35.7%)	45.6% (40.2%-51.2%)
Latin America and Caribbean	31.1% (26.8%-35.6%)	36.8% (32.1%-41.8%)	52.4% (47.5%-57.4%)	25.0% (19.5%-30.8%)	34.0% (27.6%-40.7%)	47.5% (40.4%-54.4%)	37.2% (30.5%-44.4%)	39.4% (32.5%-46.9%)	56.5% (49.3%-63.6%)
Oceania	11.0% (4.6%-21.6%)	12.3% (5.5%-23.1%)	21.5% (11.8%-34.4%)	6.1% (1.9%-16.2%)	8.5% (2.9%-20.7%)	16.0% (6.4%-33.1%)	16.1% (5.2%-37.1%)	16.2% (5.4%-36.6%)	27.2% (11.9%-49.1%)
South Asia	41.0% (32.3%-50.1%)	41.9% (32.7%-51.5%)	63.2% (54.8%-71.1%)	33.4% (21.9%-45.8%)	35.8% (23.6%-48.9%)	55.2% (42.2%-67.1%)	49.0% (35.4%-62.6%)	48.1% (34.2%-62.1%)	70.6% (58.6%-80.6%)
Sub-Saharan Africa	12.3% (9.3%-16.3%)	15.8% (12.3%-20.4%)	29.3% (24.1%-34.9%)	9.6% (6.5%-13.8%)	14.2% (10.0%-19.8%)	25.0% (18.8%-32.4%)	14.9% (10.0%-22.0%)	17.3% (12.0%-24.8%)	32.7% (25.1%-41.4%)
World Bank 2022 Income group									
Low income	12.8% (10.2%-15.9%)	15.6% (12.6%-19.2%)	30.0% (25.1%-35.2%)	9.6% (7.0%-13.0%)	13.6% (10.1%-18.0%)	24.8% (19.2%-31.2%)	16.1% (11.7%-21.5%)	17.6% (12.7%-23.4%)	34.1% (26.6%-42.1%)
Lower Middle income	33.6% (28.1%-39.4%)	35.4% (29.6%-41.6%)	55.4% (49.9%-60.7%)	27.4% (19.9%-35.3%)	30.6% (22.7%-38.9%)	48.2% (39.5%-56.3%)	40.0% (31.5%-48.6%)	40.3% (31.5%-49.2%)	61.7% (54.0%-68.3%)
Upper Middle income	26.3% (22.8%-30.3%)	23.3% (20.0%-27.1%)	34.8% (30.4%-39.3%)	26.7% (21.5%-32.6%)	25.8% (20.6%-31.9%)	34.4% (28.4%-41.3%)	25.9% (21.2%-31.3%)	21.0% (16.9%-25.9%)	35.0% (29.3%-41.6%)
High income	28.9% (26.2%-31.7%)	32.8% (30.0%-35.7%)	46.4% (43.3%-49.6%)	25.2% (22.0%-28.7%)	31.2% (27.7%-34.8%)	41.4% (37.2%-45.7%)	32.7% (28.4%-37.4%)	34.5% (30.3%-39.0%)	50.6% (46.0%-55.2%)

Appendix 14. Age-standardised prevalence of insufficient physical activity among adults aged 18 years and over in 2000, 2010 and 2022, projected prevalence in 2030 assuming trends 2010-2022 continue, and progress during 2010-2022 toward the global target of reducing the prevalence of insufficient physical activity by 15% between 2010 and 2030, by country for both sexes.

Notes: UI: Uncertainty interval. * indicates no surveys from the country were included; ^ indicates 1 or 2 surveys from the country were included.

	Prevalence in 2000 (95% UI)	Prevalence in 2010 (95% UI)	Prevalence in 2022 (95% UI)	Prevalence in 2030 if trends 2010-2022 continue (95% UI)	Progress towards global target during 2010-2022
Afghanistan^	22.2% (9.1%-39.1%)	26.4% (17.1%-37.0%)	33.4% (23.0%-44.2%)	38.6% (21.9%-57.4%)	Off track, higher certainty
Albania*	20.3% (7.5%-39.0%)	21.2% (9.7%-37.5%)	24.3% (9.0%-45.9%)	26.7% (6.8%-55.1%)	Off track, lower certainty
Algeria^	23.9% (10.9%-39.8%)	25.8% (17.4%-34.7%)	29.0% (19.0%-39.9%)	31.6% (16.0%-49.8%)	Off track, higher certainty
Andorra^	35.9% (24.5%-48.2%)	31.8% (22.3%-42.4%)	27.2% (12.3%-46.6%)	24.7% (7.0%-51.3%)	On track, lower certainty
Angola*	22.2% (9.0%-40.3%)	19.5% (9.1%-34.3%)	17.3% (5.5%-36.4%)	16.4% (3.0%-41.0%)	On track, lower certainty
Antigua and Barbuda*	29.9% (13.7%-50.6%)	33.5% (18.2%-51.3%)	38.1% (17.8%-61.0%)	41.4% (15.0%-70.4%)	Off track, higher certainty
Argentina	33.9% (21.6%-48.2%)	35.5% (28.3%-43.0%)	38.5% (28.5%-48.9%)	40.6% (24.7%-57.6%)	Off track, higher certainty
Armenia^	23.3% (10.9%-40.3%)	24.3% (16.4%-33.7%)	26.6% (16.3%-38.5%)	28.5% (12.7%-48.1%)	Off track, lower certainty
Australia	26.6% (18.1%-36.6%)	26.2% (20.6%-32.3%)	25.5% (19.4%-32.2%)	25.0% (16.4%-35.1%)	Off track, lower certainty
Austria	33.1% (25.3%-41.7%)	26.8% (22.7%-31.3%)	19.8% (15.5%-24.4%)	15.8% (10.3%-22.2%)	On track, higher certainty
Azerbaijan^	22.5% (10.1%-39.4%)	22.5% (14.8%-31.6%)	23.9% (15.3%-34.5%)	25.2% (11.9%-42.4%)	Off track, lower certainty
Bahamas^	37.4% (22.6%-53.8%)	37.6% (29.4%-46.4%)	37.2% (26.9%-47.6%)	37.1% (20.8%-54.3%)	Off track, lower certainty
Bahrain*	31.7% (15.4%-52.1%)	34.8% (20.0%-52.0%)	38.6% (19.4%-60.7%)	41.3% (16.2%-69.3%)	Off track, higher certainty
Bangladesh	21.7% (14.2%-30.2%)	20.8% (16.5%-25.5%)	20.3% (14.3%-27.2%)	20.1% (11.4%-31.2%)	Off track, lower certainty
Barbados^	34.7% (22.6%-48.1%)	39.1% (29.4%-48.8%)	44.0% (25.1%-63.4%)	47.4% (20.4%-74.0%)	Off track, higher certainty
Belarus^	18.8% (7.8%-34.7%)	16.3% (10.5%-23.7%)	14.0% (9.3%-19.8%)	12.9% (5.7%-22.7%)	On track, lower certainty
Belgium	47.1% (40.3%-53.8%)	37.0% (32.5%-41.3%)	25.4% (19.2%-32.3%)	19.0% (11.7%-28.1%)	On track, higher certainty
Belize*	32.5% (15.6%-53.4%)	36.7% (21.2%-54.2%)	41.5% (20.8%-63.7%)	44.8% (17.9%-72.9%)	Off track, higher certainty
Benin^	16.6% (7.7%-28.4%)	17.1% (11.6%-23.6%)	18.0% (9.8%-28.7%)	18.9% (6.9%-36.5%)	Off track, lower certainty
Bhutan^	12.5% (4.9%-24.3%)	10.7% (6.8%-15.6%)	9.9% (5.9%-15.0%)	9.7% (3.7%-18.6%)	Off track, lower certainty
Bolivia (Plurinational State of)^	22.3% (9.5%-40.0%)	21.9% (13.8%-31.8%)	22.6% (14.7%-31.9%)	23.4% (11.2%-38.6%)	Off track, lower certainty
Bosnia and Herzegovina^	15.3% (8.8%-23.3%)	17.1% (10.1%-25.8%)	21.1% (7.7%-40.1%)	24.1% (5.8%-52.1%)	Off track, higher certainty
Botswana^	28.8% (18.0%-41.0%)	25.6% (20.0%-32.0%)	22.3% (12.4%-34.1%)	20.5% (7.5%-38.5%)	On track, lower certainty
Brazil	31.9% (22.9%-42.3%)	35.0% (29.6%-40.7%)	40.4% (31.4%-49.8%)	44.1% (30.4%-58.7%)	Off track, higher certainty
Brunei Darussalam^	26.2% (13.0%-42.3%)	28.9% (20.4%-38.0%)	32.5% (20.8%-45.7%)	35.3% (16.7%-56.5%)	Off track, higher certainty

	Prevalence in 2000 (95% UI)	Prevalence in 2010 (95% UI)	Prevalence in 2022 (95% UI)	Prevalence in 2030 if trends 2010-2022 continue (95% UI)	Progress towards global target during 2010-2022
Bulgaria	20.1% (12.3%-29.4%)	25.1% (19.9%-30.7%)	32.3% (24.6%-40.5%)	37.5% (24.9%-51.1%)	Off track, higher certainty
Burkina Faso	16.0% (10.1%-23.2%)	16.5% (12.7%-21.0%)	16.9% (11.4%-23.3%)	17.3% (9.3%-27.2%)	Off track, lower certainty
Burundi*	17.8% (6.9%-34.8%)	15.4% (6.6%-28.3%)	14.3% (4.1%-30.7%)	13.9% (2.4%-35.1%)	Off track, lower certainty
Cabo Verde^	21.8% (12.3%-33.4%)	25.9% (19.6%-32.7%)	31.2% (22.2%-41.1%)	35.1% (20.9%-50.8%)	Off track, higher certainty
Cambodia^	14.0% (7.3%-23.0%)	13.3% (9.3%-18.2%)	13.3% (8.9%-18.7%)	13.4% (7.0%-22.2%)	Off track, lower certainty
Cameroon*	23.9% (10.2%-42.6%)	21.6% (10.3%-36.5%)	18.8% (6.2%-37.5%)	17.4% (3.5%-41.5%)	On track, lower certainty
Canada	25.6% (16.8%-35.6%)	31.1% (25.5%-37.2%)	37.2% (30.0%-44.8%)	41.4% (30.1%-53.6%)	Off track, higher certainty
Central African Republic*	19.8% (7.8%-36.3%)	17.7% (7.8%-31.0%)	16.5% (5.2%-34.5%)	16.1% (3.2%-40.4%)	Off track, lower certainty
Chad^	23.5% (15.7%-32.8%)	20.7% (13.4%-30.0%)	17.8% (6.6%-34.8%)	16.4% (3.5%-39.6%)	On track, lower certainty
Chile	23.3% (12.6%-36.4%)	29.1% (22.7%-35.8%)	38.2% (28.5%-48.6%)	44.7% (27.8%-62.9%)	Off track, higher certainty
China	19.8% (14.2%-26.4%)	21.1% (18.0%-24.3%)	23.8% (17.7%-30.8%)	25.8% (15.9%-37.3%)	Off track, higher certainty
Colombia*	26.2% (10.8%-46.7%)	29.7% (15.1%-47.2%)	34.4% (14.8%-57.1%)	37.9% (12.7%-66.8%)	Off track, higher certainty
Comoros^	9.7% (5.3%-15.6%)	12.3% (8.6%-16.7%)	17.2% (7.6%-31.0%)	21.2% (6.4%-44.7%)	Off track, higher certainty
Congo*	21.8% (8.7%-40.3%)	18.8% (8.5%-33.1%)	16.0% (4.9%-33.4%)	14.7% (2.6%-37.9%)	On track, lower certainty
Cook Islands	57.5% (46.3%-68.4%)	41.5% (35.2%-48.1%)	25.4% (18.2%-33.7%)	17.0% (8.9%-27.4%)	On track, higher certainty
Costa Rica^	41.8% (27.4%-57.5%)	45.5% (38.1%-53.2%)	49.7% (34.9%-64.1%)	52.4% (29.3%-73.8%)	Off track, higher certainty
Croatia	12.6% (7.4%-19.2%)	18.6% (14.3%-23.1%)	28.4% (21.3%-36.5%)	36.2% (24.2%-49.3%)	Off track, higher certainty
Cuba^	32.8% (19.5%-47.4%)	45.6% (37.3%-53.8%)	61.1% (50.5%-70.9%)	70.4% (54.7%-83.6%)	Off track, higher certainty
Cyprus	42.4% (30.7%-54.1%)	42.3% (35.3%-49.2%)	40.8% (32.1%-49.8%)	39.9% (26.8%-53.7%)	Off track, lower certainty
Czechia	24.2% (16.9%-32.1%)	23.2% (18.9%-27.9%)	23.4% (17.0%-30.3%)	23.5% (14.2%-34.2%)	Off track, lower certainty
Côte d'Ivoire^	25.0% (16.5%-35.2%)	22.3% (14.0%-31.8%)	19.7% (6.9%-37.6%)	18.4% (3.6%-43.2%)	On track, lower certainty
Democratic People's Republic of Korea*	20.7% (8.2%-38.9%)	22.9% (11.3%-38.1%)	27.0% (11.2%-47.8%)	30.1% (9.3%-58.2%)	Off track, higher certainty
Democratic Republic of the Congo*	18.7% (7.4%-35.2%)	16.4% (7.1%-29.0%)	14.9% (4.3%-31.0%)	14.3% (2.6%-35.6%)	Off track, lower certainty
Denmark	26.5% (19.2%-34.5%)	19.3% (15.5%-23.4%)	12.1% (8.2%-16.9%)	8.6% (4.4%-14.7%)	On track, higher certainty
Djibouti*	20.3% (8.1%-38.3%)	18.1% (8.0%-32.4%)	17.0% (5.2%-34.9%)	16.6% (3.1%-40.1%)	Off track, lower certainty
Dominica^	19.9% (11.1%-30.7%)	24.0% (16.3%-32.4%)	30.5% (14.4%-49.2%)	35.4% (12.0%-62.8%)	Off track, higher certainty
Dominican Republic*	27.5% (12.4%-47.0%)	31.6% (17.3%-49.2%)	37.0% (17.2%-59.9%)	40.8% (14.6%-69.6%)	Off track, higher certainty
Ecuador^	21.9% (9.5%-39.2%)	21.6% (13.7%-30.9%)	22.8% (14.1%-32.8%)	23.9% (10.8%-40.5%)	Off track, lower certainty
Egypt^	30.6% (17.9%-45.7%)	32.0% (25.1%-39.5%)	33.8% (23.6%-45.3%)	35.3% (18.8%-54.2%)	Off track, lower certainty
El Salvador^	30.3% (14.9%-48.6%)	34.1% (24.2%-44.6%)	39.2% (25.8%-53.2%)	42.8% (21.9%-65.1%)	Off track, higher certainty
Equatorial Guinea*	24.4% (10.3%-43.3%)	21.8% (10.6%-37.5%)	19.5% (6.7%-38.6%)	18.3% (4.2%-42.5%)	On track, lower certainty

	Prevalence in 2000 (95% UI)	Prevalence in 2010 (95% UI)	Prevalence in 2022 (95% UI)	Prevalence in 2030 if trends 2010-2022 continue (95% UI)	Progress towards global target during 2010-2022
Eritrea^	14.3% (6.7%-24.9%)	12.3% (7.7%-18.1%)	11.8% (4.3%-23.9%)	11.9% (2.3%-31.7%)	Off track, lower certainty
Estonia	12.3% (7.9%-17.5%)	14.2% (11.1%-17.6%)	15.9% (11.7%-20.7%)	17.3% (10.8%-25.0%)	Off track, higher certainty
Eswatini^	24.9% (11.7%-41.2%)	21.7% (14.7%-30.0%)	19.2% (10.1%-30.7%)	18.0% (5.9%-37.2%)	On track, lower certainty
Ethiopia^	14.2% (8.5%-21.5%)	11.4% (8.1%-15.6%)	9.4% (4.6%-16.2%)	8.4% (2.6%-18.2%)	On track, lower certainty
Fiji^	24.2% (12.5%-38.3%)	19.0% (12.8%-26.4%)	15.5% (6.3%-28.6%)	14.0% (2.9%-33.7%)	On track, lower certainty
Finland	27.4% (20.6%-35.3%)	17.8% (14.4%-21.6%)	9.6% (6.2%-13.8%)	6.0% (2.8%-10.6%)	On track, higher certainty
France	38.9% (32.0%-46.2%)	32.1% (27.8%-36.3%)	23.2% (16.7%-30.3%)	18.2% (10.5%-28.0%)	On track, higher certainty
Gabon*	25.6% (11.6%-44.5%)	23.0% (11.2%-38.2%)	20.8% (7.1%-40.2%)	19.7% (4.3%-44.3%)	Off track, lower certainty
Gambia^	22.4% (12.2%-35.2%)	21.9% (15.1%-29.7%)	21.2% (9.9%-35.6%)	21.0% (6.2%-43.4%)	Off track, lower certainty
Georgia	15.3% (9.7%-22.3%)	18.7% (14.7%-23.1%)	24.0% (15.3%-34.6%)	28.1% (14.3%-45.5%)	Off track, higher certainty
Germany	20.3% (14.9%-26.6%)	16.6% (14.0%-19.2%)	12.0% (9.2%-15.1%)	9.5% (5.9%-14.0%)	On track, higher certainty
Ghana^	17.0% (9.2%-27.4%)	19.0% (14.2%-24.5%)	21.2% (11.9%-32.8%)	22.9% (9.1%-42.6%)	Off track, lower certainty
Greece	28.7% (21.1%-37.1%)	32.0% (27.0%-37.5%)	35.2% (27.2%-43.7%)	37.4% (25.4%-50.1%)	Off track, higher certainty
Grenada^	27.3% (15.3%-42.6%)	30.8% (22.7%-40.0%)	35.9% (20.7%-52.5%)	39.5% (17.0%-64.6%)	Off track, higher certainty
Guatemala^	10.6% (5.8%-16.7%)	20.0% (14.9%-25.4%)	36.8% (23.9%-50.7%)	49.9% (28.8%-70.7%)	Off track, higher certainty
Guinea*	19.6% (7.6%-37.2%)	18.0% (7.9%-32.2%)	16.7% (5.0%-34.4%)	16.3% (3.0%-39.5%)	Off track, lower certainty
Guinea-Bissau*	21.2% (8.7%-39.6%)	19.3% (8.7%-33.9%)	17.6% (5.6%-36.1%)	16.9% (3.3%-41.0%)	Off track, lower certainty
Guyana^	26.4% (12.6%-43.4%)	29.8% (20.9%-39.8%)	35.1% (23.2%-47.5%)	39.0% (19.9%-59.1%)	Off track, higher certainty
Haiti*	21.8% (8.4%-41.2%)	24.4% (11.8%-40.5%)	28.3% (10.8%-49.5%)	31.2% (8.6%-59.2%)	Off track, higher certainty
Honduras*	27.8% (11.9%-47.9%)	31.6% (16.5%-48.7%)	36.8% (16.1%-59.1%)	40.5% (14.0%-68.8%)	Off track, higher certainty
Hungary	17.9% (12.2%-24.6%)	22.6% (18.5%-27.1%)	29.4% (22.2%-37.3%)	34.4% (23.1%-46.8%)	Off track, higher certainty
Iceland*	32.3% (15.6%-52.7%)	29.3% (15.4%-46.8%)	25.9% (9.8%-46.7%)	24.1% (5.9%-49.8%)	On track, lower certainty
India	22.3% (15.4%-30.3%)	33.7% (28.4%-39.3%)	49.4% (38.4%-61.0%)	59.9% (43.4%-75.9%)	Off track, higher certainty
Indonesia	15.2% (8.2%-24.4%)	16.3% (11.8%-21.3%)	19.0% (12.0%-27.1%)	21.1% (10.2%-34.7%)	Off track, higher certainty
Iran (Islamic Republic of)	29.2% (21.2%-37.6%)	36.8% (32.5%-41.1%)	46.3% (37.8%-54.4%)	52.8% (39.1%-65.7%)	Off track, higher certainty
Iraq^	40.8% (26.2%-56.8%)	46.2% (37.6%-55.2%)	52.0% (38.5%-65.5%)	55.8% (34.7%-75.4%)	Off track, higher certainty
Ireland	39.1% (31.0%-48.0%)	31.3% (26.8%-35.9%)	21.9% (16.6%-27.6%)	16.8% (10.4%-24.6%)	On track, higher certainty
Israel*	34.6% (17.0%-55.4%)	31.0% (16.3%-48.4%)	26.6% (10.4%-47.6%)	24.2% (6.3%-50.8%)	On track, lower certainty
Italy	43.6% (34.7%-52.6%)	42.5% (36.8%-48.4%)	40.1% (31.8%-48.8%)	38.5% (26.7%-51.4%)	Off track, lower certainty
Jamaica^	27.9% (17.1%-40.9%)	32.7% (23.8%-42.3%)	38.6% (20.4%-57.3%)	42.7% (16.5%-69.1%)	Off track, higher certainty
Japan^	29.8% (13.4%-50.1%)	35.8% (24.3%-48.5%)	44.7% (33.7%-55.8%)	50.8% (33.5%-67.6%)	Off track, higher certainty

	Prevalence in 2000 (95% UI)	Prevalence in 2010 (95% UI)	Prevalence in 2022 (95% UI)	Prevalence in 2030 if trends 2010-2022 continue (95% UI)	Progress towards global target during 2010-2022
Jordan	26.2% (18.8%-34.4%)	27.6% (22.5%-32.9%)	28.2% (19.3%-38.2%)	28.9% (16.1%-44.1%)	Off track, lower certainty
Kazakhstan^	17.7% (11.0%-26.2%)	21.7% (13.9%-30.8%)	28.2% (12.5%-48.7%)	33.1% (10.7%-62.5%)	Off track, higher certainty
Kenya^	13.2% (7.8%-20.3%)	10.8% (7.7%-14.6%)	8.9% (4.4%-15.3%)	8.0% (2.5%-17.2%)	On track, lower certainty
Kiribati^	42.5% (27.1%-58.5%)	37.4% (28.7%-46.1%)	31.9% (20.1%-44.2%)	28.8% (12.7%-47.6%)	On track, lower certainty
Kuwait^	58.9% (44.2%-73.4%)	62.0% (52.1%-71.1%)	63.3% (45.3%-79.7%)	63.9% (38.0%-86.2%)	Off track, higher certainty
Kyrgyzstan^	16.9% (7.7%-30.2%)	17.7% (11.7%-24.8%)	19.9% (10.3%-31.7%)	21.8% (7.5%-41.4%)	Off track, lower certainty
Lao People's Democratic Republic^	16.7% (10.6%-24.5%)	15.7% (11.5%-20.7%)	15.8% (7.7%-26.5%)	16.1% (5.2%-32.6%)	Off track, lower certainty
Latvia	21.0% (12.9%-30.8%)	17.8% (13.5%-22.6%)	14.5% (9.7%-20.1%)	12.7% (6.3%-21.1%)	On track, lower certainty
Lebanon^	34.9% (22.2%-48.5%)	46.0% (38.2%-53.6%)	58.6% (46.0%-70.8%)	66.4% (46.7%-83.3%)	Off track, higher certainty
Lesotho^	13.1% (5.6%-23.8%)	9.8% (6.0%-14.7%)	8.0% (3.2%-15.8%)	7.2% (1.5%-19.3%)	On track, lower certainty
Liberia^	32.2% (18.9%-47.8%)	23.1% (16.7%-30.3%)	14.6% (9.6%-20.7%)	10.4% (4.7%-18.9%)	On track, higher certainty
Libya^	33.9% (22.0%-46.6%)	39.4% (31.7%-47.6%)	45.6% (36.5%-54.5%)	49.8% (35.6%-63.2%)	Off track, higher certainty
Lithuania	24.2% (17.8%-31.4%)	22.3% (18.6%-26.1%)	20.2% (14.7%-26.2%)	19.0% (11.3%-28.0%)	Off track, lower certainty
Luxembourg	27.4% (20.4%-35.1%)	20.9% (17.2%-24.9%)	13.9% (9.7%-19.0%)	10.3% (5.5%-17.0%)	On track, higher certainty
Madagascar*	16.4% (6.0%-32.7%)	14.3% (6.1%-27.1%)	13.6% (4.2%-29.8%)	13.4% (2.5%-34.1%)	Off track, lower certainty
Malawi	15.7% (9.8%-23.1%)	7.4% (5.4%-9.9%)	2.7% (1.1%-5.3%)	1.3% (0.3%-3.7%)	On track, higher certainty
Malaysia	41.1% (33.5%-48.8%)	41.0% (36.4%-45.6%)	39.9% (30.7%-49.5%)	39.2% (25.8%-53.5%)	Off track, lower certainty
Maldives^	25.4% (12.4%-41.6%)	24.5% (16.5%-33.6%)	24.6% (16.9%-33.6%)	24.9% (13.4%-38.6%)	Off track, lower certainty
Mali^	36.9% (21.7%-54.3%)	33.9% (21.5%-47.8%)	29.8% (13.4%-50.8%)	27.5% (7.9%-55.5%)	On track, lower certainty
Malta	55.1% (42.5%-66.7%)	49.2% (42.0%-56.4%)	40.7% (30.5%-51.3%)	35.4% (20.9%-51.6%)	On track, lower certainty
Marshall Islands^	40.4% (25.9%-55.5%)	31.9% (19.7%-45.6%)	24.4% (9.0%-44.7%)	20.5% (4.1%-46.2%)	On track, lower certainty
Mauritania^	61.1% (49.6%-71.9%)	51.3% (39.3%-62.9%)	38.5% (18.1%-61.0%)	31.1% (8.4%-61.0%)	On track, higher certainty
Mauritius^	26.4% (17.9%-35.8%)	28.2% (22.3%-34.7%)	29.9% (21.6%-39.0%)	31.0% (18.8%-44.5%)	Off track, higher certainty
Mexico	22.3% (16.5%-28.8%)	24.8% (21.1%-28.8%)	28.0% (22.1%-34.8%)	30.3% (21.1%-41.2%)	Off track, higher certainty
Micronesia (Federated States of)	44.7% (31.1%-58.6%)	36.9% (27.7%-46.5%)	29.3% (15.8%-45.4%)	25.1% (8.7%-47.5%)	On track, lower certainty
Monaco*	33.2% (16.0%-54.1%)	29.4% (15.4%-47.2%)	25.5% (9.8%-46.6%)	23.3% (5.9%-49.5%)	On track, lower certainty
Mongolia	7.6% (4.1%-12.1%)	14.4% (11.3%-17.8%)	27.5% (19.2%-37.0%)	38.7% (23.3%-55.9%)	Off track, higher certainty
Montenegro*	19.7% (7.5%-37.9%)	20.5% (9.3%-36.3%)	22.8% (8.1%-43.5%)	24.6% (6.2%-52.2%)	Off track, lower certainty
Morocco^	21.7% (10.6%-35.6%)	22.6% (16.0%-30.2%)	24.5% (15.3%-35.2%)	26.1% (11.9%-43.2%)	Off track, lower certainty
Mozambique^	7.7% (3.9%-13.1%)	7.9% (4.4%-12.8%)	9.7% (2.7%-22.1%)	11.3% (1.7%-31.3%)	Off track, lower certainty
Myanmar	21.4% (14.9%-29.3%)	21.5% (16.8%-26.7%)	22.0% (12.8%-33.5%)	22.5% (9.8%-40.5%)	Off track, lower certainty

	Prevalence in 2000 (95% UI)	Prevalence in 2010 (95% UI)	Prevalence in 2022 (95% UI)	Prevalence in 2030 if trends 2010-2022 continue (95% UI)	Progress towards global target during 2010-2022
Namibia^	37.8% (27.8%-48.9%)	31.8% (21.9%-42.8%)	25.4% (10.0%-45.1%)	22.0% (4.9%-48.8%)	On track, lower certainty
Nauru^	45.1% (33.1%-56.9%)	39.8% (32.6%-47.3%)	35.5% (23.3%-48.7%)	32.8% (15.9%-52.8%)	On track, lower certainty
Nepal	11.5% (6.8%-17.5%)	9.6% (7.1%-12.5%)	8.2% (4.9%-12.7%)	7.5% (3.1%-14.3%)	On track, lower certainty
Netherlands	19.7% (14.5%-25.6%)	14.7% (12.0%-17.8%)	9.4% (6.9%-12.4%)	6.8% (4.0%-10.5%)	On track, higher certainty
New Zealand	21.2% (14.9%-28.1%)	20.4% (16.6%-24.6%)	18.7% (14.2%-23.6%)	17.6% (11.4%-24.7%)	Off track, lower certainty
Nicaragua*	30.9% (14.4%-52.8%)	34.4% (18.8%-52.1%)	38.7% (18.0%-62.1%)	41.8% (15.1%-71.1%)	Off track, higher certainty
Niger^	22.5% (11.4%-36.8%)	19.6% (13.1%-27.2%)	16.2% (10.4%-23.3%)	14.3% (6.9%-24.6%)	On track, lower certainty
Nigeria*	21.6% (8.7%-39.4%)	19.8% (9.4%-34.3%)	18.1% (6.0%-36.9%)	17.3% (3.5%-41.2%)	Off track, lower certainty
Niue^	18.3% (7.4%-33.0%)	11.4% (6.7%-17.4%)	7.4% (2.4%-16.2%)	5.8% (0.8%-18.3%)	On track, higher certainty
Norway	31.9% (22.4%-42.5%)	33.8% (25.6%-42.3%)	35.1% (20.0%-52.5%)	36.1% (15.5%-61.1%)	Off track, lower certainty
occupied Palestinian territory, including east Jerusalem^	41.8% (28.1%-57.1%)	36.8% (29.1%-45.2%)	29.7% (21.9%-38.8%)	25.6% (14.4%-38.9%)	On track, lower certainty
Oman^	31.7% (20.0%-44.0%)	35.8% (29.1%-43.0%)	40.9% (30.0%-52.9%)	44.2% (26.8%-62.8%)	Off track, higher certainty
Pakistan^	26.4% (18.1%-35.7%)	34.2% (27.5%-41.1%)	45.7% (31.6%-60.0%)	53.5% (32.6%-73.9%)	Off track, higher certainty
Palau^	35.8% (20.1%-52.8%)	30.5% (22.8%-38.9%)	26.0% (14.6%-39.2%)	23.4% (8.3%-44.5%)	On track, lower certainty
Panama^	35.7% (18.1%-55.3%)	45.2% (33.2%-57.2%)	57.6% (46.6%-68.0%)	65.4% (48.5%-80.1%)	Off track, higher certainty
Papua New Guinea^	19.2% (10.5%-29.9%)	14.7% (9.4%-21.2%)	12.5% (3.8%-26.2%)	11.6% (1.7%-32.1%)	On track, lower certainty
Paraguay^	23.5% (15.7%-33.1%)	28.4% (19.0%-38.7%)	36.0% (17.4%-57.3%)	41.2% (15.3%-70.8%)	Off track, higher certainty
Peru^	25.9% (11.5%-44.4%)	28.6% (19.0%-39.6%)	34.5% (23.0%-46.2%)	38.8% (20.9%-57.4%)	Off track, higher certainty
Philippines	13.8% (8.2%-21.0%)	25.7% (21.0%-30.7%)	45.5% (33.5%-57.0%)	59.4% (40.4%-76.2%)	Off track, higher certainty
Poland	22.3% (14.3%-31.9%)	28.3% (22.9%-34.1%)	37.0% (28.9%-45.4%)	43.1% (30.3%-57.2%)	Off track, higher certainty
Portugal	41.9% (33.1%-50.5%)	46.7% (40.7%-52.6%)	51.7% (42.6%-60.5%)	54.9% (41.7%-67.6%)	Off track, higher certainty
Puerto Rico*	33.4% (16.0%-54.3%)	37.1% (21.4%-54.4%)	41.6% (20.1%-64.3%)	44.6% (17.2%-72.9%)	Off track, higher certainty
Qatar^	45.1% (31.3%-60.0%)	49.0% (38.5%-59.6%)	53.5% (34.8%-71.9%)	56.2% (29.5%-81.3%)	Off track, higher certainty
Republic of Korea	27.0% (19.6%-35.3%)	39.8% (35.8%-43.8%)	58.1% (52.4%-63.7%)	69.3% (59.9%-78.0%)	Off track, higher certainty
Republic of Moldova^	15.9% (7.0%-28.4%)	13.3% (8.9%-19.0%)	10.8% (6.9%-15.7%)	9.5% (4.2%-17.5%)	On track, lower certainty
Republic of North Macedonia^	22.1% (9.3%-38.6%)	23.3% (13.5%-34.8%)	26.1% (12.9%-42.0%)	28.4% (10.3%-51.4%)	Off track, lower certainty
Romania	18.8% (8.9%-32.4%)	25.6% (18.4%-33.6%)	36.8% (28.6%-45.6%)	45.1% (30.6%-59.6%)	Off track, higher certainty
Russian Federation^	10.7% (6.4%-16.0%)	13.5% (9.0%-18.7%)	18.1% (7.3%-34.3%)	21.8% (5.9%-47.9%)	Off track, higher certainty
Rwanda^	20.7% (10.7%-33.9%)	14.0% (9.4%-19.8%)	9.0% (5.6%-13.3%)	6.6% (2.7%-12.4%)	On track, higher certainty
Saint Kitts and Nevis^	32.0% (17.8%-48.0%)	36.5% (23.6%-49.5%)	41.7% (22.1%-62.2%)	45.2% (18.6%-73.3%)	Off track, higher certainty
Saint Lucia^	24.5% (11.3%-41.5%)	22.7% (15.0%-32.0%)	20.9% (13.7%-29.1%)	20.1% (9.5%-33.7%)	Off track, lower certainty

	Prevalence in 2000 (95% UI)	Prevalence in 2010 (95% UI)	Prevalence in 2022 (95% UI)	Prevalence in 2030 if trends 2010-2022 continue (95% UI)	Progress towards global target during 2010-2022
Saint Vincent and the Grenadines^	24.7% (12.1%-40.7%)	26.9% (19.0%-35.8%)	30.5% (18.4%-43.9%)	33.1% (14.7%-54.9%)	Off track, higher certainty
Samoa^	38.5% (28.8%-48.3%)	25.1% (19.3%-31.3%)	13.5% (6.1%-23.6%)	8.5% (2.1%-20.3%)	On track, higher certainty
San Marino*	33.4% (16.3%-53.4%)	29.6% (15.4%-46.4%)	25.7% (10.0%-45.5%)	23.4% (5.8%-49.6%)	On track, lower certainty
Saudi Arabia	57.8% (47.3%-68.3%)	55.6% (50.1%-61.1%)	51.5% (40.9%-61.8%)	48.9% (32.6%-64.8%)	Off track, lower certainty
Senegal^	20.9% (9.5%-36.3%)	18.8% (12.2%-26.8%)	16.6% (8.8%-26.8%)	15.7% (5.0%-31.9%)	On track, lower certainty
Serbia^	48.9% (36.0%-62.0%)	46.8% (36.2%-58.0%)	44.9% (23.6%-65.7%)	43.7% (15.4%-72.4%)	Off track, lower certainty
Seychelles^	22.1% (14.5%-31.1%)	21.4% (16.1%-27.2%)	20.4% (10.7%-32.2%)	20.0% (7.4%-38.1%)	Off track, lower certainty
Sierra Leone^	17.7% (9.2%-28.8%)	15.3% (9.8%-21.9%)	13.0% (4.9%-25.0%)	11.9% (2.3%-29.7%)	On track, lower certainty
Singapore	25.2% (16.6%-35.6%)	23.5% (19.3%-28.2%)	23.2% (17.2%-29.9%)	23.0% (13.4%-34.7%)	Off track, lower certainty
Slovakia	27.0% (14.6%-42.4%)	24.8% (18.1%-32.7%)	23.3% (16.5%-30.5%)	22.5% (11.9%-34.8%)	Off track, lower certainty
Slovenia	19.5% (12.1%-28.6%)	18.8% (14.5%-23.9%)	19.0% (13.3%-25.4%)	19.1% (10.8%-29.3%)	Off track, lower certainty
Solomon Islands^	36.6% (24.8%-49.9%)	26.4% (20.4%-32.8%)	17.5% (9.2%-27.9%)	13.1% (4.0%-27.5%)	On track, higher certainty
Somalia*	21.3% (8.5%-39.5%)	19.1% (8.8%-33.2%)	18.1% (6.0%-36.3%)	17.8% (3.7%-43.0%)	Off track, lower certainty
South Africa	49.4% (41.0%-57.8%)	48.4% (42.6%-54.6%)	44.8% (31.8%-58.7%)	42.5% (24.0%-62.5%)	Off track, lower certainty
South Sudan*	19.5% (7.3%-37.6%)	17.2% (7.4%-31.2%)	16.3% (4.8%-34.2%)	16.0% (2.8%-40.4%)	Off track, lower certainty
Spain	40.5% (32.1%-49.0%)	32.6% (28.1%-37.1%)	21.8% (16.3%-27.7%)	16.1% (9.4%-24.2%)	On track, higher certainty
Sri Lanka	18.3% (12.4%-25.2%)	26.0% (21.4%-30.9%)	37.2% (28.5%-45.8%)	45.4% (31.9%-59.1%)	Off track, higher certainty
Sudan^	23.1% (10.9%-38.8%)	20.6% (13.5%-28.6%)	19.1% (11.1%-29.3%)	18.4% (7.3%-34.6%)	Off track, lower certainty
Suriname^	35.8% (20.4%-52.6%)	41.6% (32.3%-51.4%)	48.9% (33.8%-64.8%)	53.5% (30.5%-76.4%)	Off track, higher certainty
Sweden	29.9% (22.1%-38.3%)	18.5% (14.8%-22.6%)	8.7% (5.6%-12.7%)	4.8% (2.2%-8.9%)	On track, higher certainty
Switzerland^	28.6% (14.5%-45.6%)	23.8% (15.9%-32.8%)	19.0% (10.6%-29.9%)	16.6% (6.0%-32.9%)	On track, lower certainty
Syrian Arab Republic*	30.8% (14.6%-50.7%)	33.9% (18.7%-51.4%)	37.8% (17.5%-60.6%)	40.7% (14.5%-69.8%)	Off track, higher certainty
São Tomé and Príncipe^	17.6% (9.1%-28.5%)	17.9% (13.0%-23.7%)	18.2% (11.3%-26.3%)	18.5% (8.5%-32.1%)	Off track, lower certainty
Taiwan, China*	21.9% (9.0%-39.4%)	23.8% (11.6%-39.5%)	27.4% (11.0%-48.7%)	30.0% (8.8%-57.8%)	Off track, higher certainty
Tajikistan^	25.7% (12.0%-42.8%)	29.5% (20.5%-39.6%)	35.7% (24.0%-48.4%)	40.3% (21.7%-60.6%)	Off track, higher certainty
Thailand	15.9% (9.8%-23.5%)	21.0% (17.7%-24.8%)	28.9% (23.5%-34.7%)	34.8% (24.6%-46.3%)	Off track, higher certainty
Timor-Leste^	17.4% (7.1%-31.4%)	20.6% (12.0%-31.0%)	26.9% (13.8%-43.2%)	31.7% (12.3%-55.7%)	Off track, higher certainty
Togo^	13.5% (6.5%-22.6%)	14.0% (9.6%-19.1%)	14.8% (9.8%-20.5%)	15.4% (7.8%-25.2%)	Off track, lower certainty
Tonga	32.5% (19.7%-46.5%)	30.8% (24.4%-37.6%)	30.7% (20.2%-42.4%)	30.8% (15.0%-50.1%)	Off track, lower certainty
Trinidad and Tobago^	36.2% (21.9%-51.8%)	40.1% (31.0%-49.8%)	44.5% (27.8%-62.0%)	47.4% (22.2%-72.9%)	Off track, higher certainty
Tunisia^	22.7% (15.0%-31.6%)	27.2% (18.4%-37.3%)	33.4% (15.9%-54.6%)	37.7% (13.1%-66.8%)	Off track, higher certainty

	Prevalence in 2000 (95% UI)	Prevalence in 2010 (95% UI)	Prevalence in 2022 (95% UI)	Prevalence in 2030 if trends 2010-2022 continue (95% UI)	Progress towards global target during 2010-2022
Turkmenistan^	17.1% (6.6%-32.1%)	14.3% (8.3%-21.6%)	12.1% (6.8%-19.1%)	11.1% (3.9%-22.3%)	On track, lower certainty
Tuvalu^	38.2% (20.6%-57.1%)	30.9% (21.7%-40.5%)	24.4% (13.9%-37.2%)	21.0% (7.4%-40.4%)	On track, lower certainty
Türkiye	38.8% (29.9%-47.6%)	41.6% (35.6%-48.0%)	44.4% (32.4%-56.7%)	46.3% (28.8%-64.0%)	Off track, higher certainty
Uganda^	11.8% (4.9%-22.3%)	7.9% (4.7%-12.2%)	5.5% (3.4%-8.3%)	4.4% (1.7%-8.6%)	On track, higher certainty
Ukraine^	10.2% (5.7%-16.4%)	11.3% (7.8%-15.3%)	12.8% (7.4%-19.5%)	14.0% (6.3%-24.7%)	Off track, higher certainty
United Arab Emirates	45.9% (35.4%-56.7%)	56.9% (50.4%-63.1%)	66.1% (54.6%-76.8%)	71.6% (54.9%-85.4%)	Off track, higher certainty
United Kingdom	37.5% (29.4%-46.0%)	29.0% (24.9%-33.5%)	19.0% (14.4%-24.5%)	13.7% (8.2%-20.7%)	On track, higher certainty
United Republic of Tanzania^	13.7% (6.3%-24.0%)	8.1% (5.0%-12.0%)	4.3% (2.5%-6.8%)	2.8% (1.0%-5.8%)	On track, higher certainty
United States of America	29.3% (21.6%-37.3%)	31.9% (28.0%-35.8%)	33.7% (26.6%-41.8%)	35.0% (23.6%-48.3%)	Off track, higher certainty
Uruguay	29.3% (20.3%-39.0%)	30.8% (23.1%-39.1%)	33.8% (18.7%-51.3%)	36.0% (14.8%-61.2%)	Off track, higher certainty
Uzbekistan	8.5% (4.7%-13.4%)	16.5% (12.7%-20.6%)	31.7% (23.2%-40.7%)	44.4% (29.6%-59.3%)	Off track, higher certainty
Vanuatu^	16.1% (7.2%-28.2%)	10.6% (6.6%-15.9%)	7.5% (2.6%-16.0%)	6.3% (0.9%-18.5%)	On track, lower certainty
Venezuela (Bolivarian Republic of)^	35.7% (18.8%-54.8%)	40.8% (30.2%-51.9%)	45.7% (32.6%-59.4%)	49.1% (28.9%-69.6%)	Off track, higher certainty
Viet Nam	16.0% (10.5%-22.9%)	21.5% (17.6%-25.7%)	30.3% (23.0%-38.1%)	37.1% (24.8%-49.8%)	Off track, higher certainty
Yemen*	21.2% (8.7%-39.5%)	24.1% (11.6%-40.3%)	29.2% (11.7%-50.9%)	33.0% (10.4%-61.9%)	Off track, higher certainty
Zambia^	18.0% (11.2%-26.3%)	15.7% (11.5%-20.5%)	14.1% (7.9%-21.9%)	13.2% (5.2%-24.7%)	On track, lower certainty
Zimbabwe^	20.4% (13.1%-29.2%)	18.4% (11.3%-27.5%)	17.7% (6.0%-36.1%)	17.6% (3.5%-44.0%)	Off track, lower certainty

Appendix 15. Age-standardised prevalence of insufficient physical activity among adults aged 18 years and over in 2000, 2010 and 2022, projected prevalence in 2030 assuming trends 2010-2022 continue, and progress during 2010-2022 toward the global target of reducing the prevalence of insufficient physical activity by 15% between 2010 and 2030, by country for men.

Notes: UI: Uncertainty interval. * indicates no surveys from the country were included; ^ indicates 1 or 2 surveys from the country were included.

	Prevalence in 2000 (95% UI)	Prevalence in 2010 (95% UI)	Prevalence in 2022 (95% UI)	Prevalence in 2030 if trends 2010-2022 continue (95% UI)	Progress towards global target during 2010-2022
Afghanistan^	15.1% (4.0%-34.4%)	16.8% (8.4%-28.1%)	20.0% (9.9%-33.1%)	22.9% (7.6%-44.5%)	Off track, lower certainty
Albania*	16.0% (3.9%-37.7%)	17.6% (6.3%-36.7%)	21.4% (5.6%-48.5%)	24.6% (4.1%-60.6%)	Off track, lower certainty
Algeria^	17.1% (5.2%-36.0%)	18.1% (9.8%-28.5%)	20.1% (10.1%-33.2%)	22.1% (7.1%-43.9%)	Off track, lower certainty
Andorra^	33.5% (19.0%-50.5%)	30.5% (18.6%-44.5%)	26.2% (8.7%-50.7%)	24.1% (4.1%-57.5%)	On track, lower certainty
Angola*	18.5% (5.2%-40.3%)	16.0% (5.8%-33.3%)	13.9% (3.0%-36.0%)	13.2% (1.5%-40.9%)	On track, lower certainty
Antigua and Barbuda*	24.5% (7.4%-49.5%)	27.3% (11.6%-50.6%)	30.8% (10.2%-60.6%)	33.6% (7.2%-70.4%)	Off track, lower certainty
Argentina	32.5% (17.1%-51.0%)	33.8% (24.5%-43.6%)	36.9% (24.3%-50.3%)	39.2% (19.7%-60.6%)	Off track, higher certainty
Armenia^	20.7% (6.9%-42.2%)	23.4% (13.5%-35.4%)	28.2% (15.0%-44.3%)	32.2% (11.9%-57.4%)	Off track, higher certainty
Australia	22.0% (12.0%-35.2%)	23.0% (16.2%-31.5%)	23.7% (16.0%-33.1%)	24.4% (13.4%-38.5%)	Off track, lower certainty
Austria	29.4% (19.7%-40.1%)	25.1% (19.6%-30.7%)	19.6% (14.1%-26.2%)	16.5% (9.2%-25.6%)	On track, higher certainty
Azerbaijan^	19.2% (6.0%-39.6%)	20.9% (11.5%-32.9%)	25.1% (13.6%-39.4%)	28.7% (10.7%-52.2%)	Off track, higher certainty
Bahamas^	28.5% (12.1%-50.1%)	28.2% (18.4%-39.4%)	26.6% (15.4%-39.8%)	26.0% (9.4%-47.2%)	Off track, lower certainty
Bahrain*	29.5% (9.9%-55.3%)	32.7% (15.3%-55.2%)	35.7% (12.5%-65.1%)	38.1% (9.4%-75.0%)	Off track, lower certainty
Bangladesh	11.3% (5.4%-19.4%)	14.7% (10.2%-19.9%)	19.7% (12.1%-28.8%)	23.7% (11.5%-39.4%)	Off track, higher certainty
Barbados^	28.7% (14.9%-46.5%)	32.2% (20.4%-45.1%)	36.3% (14.8%-61.7%)	39.1% (10.4%-74.3%)	Off track, lower certainty
Belarus^	15.5% (4.5%-33.3%)	15.1% (8.5%-24.0%)	14.6% (8.5%-22.4%)	14.9% (5.4%-29.2%)	Off track, lower certainty
Belgium	41.2% (32.4%-50.1%)	32.5% (27.0%-38.3%)	22.4% (14.7%-31.5%)	16.9% (8.1%-28.5%)	On track, higher certainty
Belize*	27.8% (9.3%-54.2%)	31.2% (14.0%-54.6%)	35.2% (12.7%-64.8%)	38.1% (9.7%-75.2%)	Off track, lower certainty
Benin^	14.4% (4.6%-28.4%)	14.9% (8.6%-23.1%)	15.6% (6.5%-28.8%)	16.6% (3.9%-39.1%)	Off track, lower certainty
Bhutan^	8.5% (2.1%-20.0%)	8.3% (4.3%-14.0%)	8.9% (4.2%-15.4%)	9.8% (2.6%-22.3%)	Off track, lower certainty
Bolivia (Plurinational State of)^	18.4% (5.3%-39.4%)	17.8% (8.9%-29.4%)	18.3% (9.6%-29.4%)	19.3% (6.8%-36.9%)	Off track, lower certainty
Bosnia and Herzegovina^	10.9% (4.7%-19.6%)	13.5% (6.4%-23.1%)	18.4% (4.6%-40.6%)	22.4% (3.1%-55.4%)	Off track, higher certainty
Botswana^	22.7% (11.5%-37.2%)	20.0% (13.4%-27.4%)	17.3% (7.3%-31.1%)	16.1% (3.6%-37.3%)	On track, lower certainty
Brazil	30.9% (18.8%-44.6%)	32.4% (25.0%-40.0%)	35.6% (24.4%-47.8%)	37.8% (20.8%-57.2%)	Off track, higher certainty
Brunei Darussalam^	22.8% (7.9%-43.8%)	25.3% (14.7%-37.9%)	27.8% (13.9%-44.8%)	30.0% (10.1%-57.6%)	Off track, lower certainty
Bulgaria	17.3% (8.3%-29.0%)	23.5% (16.8%-31.1%)	32.4% (22.1%-43.9%)	39.1% (22.1%-57.6%)	Off track, higher certainty
Burkina Faso	15.9% (8.4%-25.7%)	15.4% (10.6%-21.1%)	14.3% (8.1%-22.1%)	13.8% (5.5%-25.6%)	Off track, lower certainty

	Prevalence in 2000 (95% UI)	Prevalence in 2010 (95% UI)	Prevalence in 2022 (95% UI)	Prevalence in 2030 if trends 2010-2022 continue (95% UI)	Progress towards global target during 2010-2022
Burundi*	16.9% (4.3%-38.2%)	14.8% (5.2%-32.9%)	14.0% (2.9%-35.5%)	14.0% (1.5%-41.2%)	Off track, lower certainty
Cabo Verde^	16.1% (6.7%-28.8%)	19.2% (12.3%-27.2%)	22.4% (12.6%-34.7%)	24.8% (10.3%-44.7%)	Off track, higher certainty
Cambodia^	12.7% (4.8%-24.5%)	12.4% (7.4%-19.0%)	12.6% (7.0%-19.8%)	13.1% (5.1%-24.8%)	Off track, lower certainty
Cameroon*	21.5% (6.6%-44.9%)	19.3% (7.3%-38.3%)	16.1% (3.7%-39.5%)	14.8% (1.6%-43.7%)	On track, lower certainty
Canada	23.3% (12.3%-36.9%)	29.2% (21.9%-37.3%)	35.8% (26.1%-45.9%)	40.4% (24.7%-56.9%)	Off track, higher certainty
Central African Republic*	17.3% (4.5%-38.3%)	15.2% (5.3%-32.9%)	13.8% (3.1%-36.0%)	13.4% (1.6%-41.8%)	Off track, lower certainty
Chad^	21.5% (11.7%-34.1%)	19.3% (10.4%-30.8%)	16.5% (4.1%-37.9%)	15.3% (1.8%-44.3%)	On track, lower certainty
Chile	21.6% (9.2%-39.0%)	24.8% (17.4%-33.3%)	30.4% (19.1%-43.3%)	34.6% (15.8%-57.0%)	Off track, higher certainty
China	20.4% (12.7%-29.8%)	23.2% (18.8%-28.1%)	28.0% (18.7%-38.6%)	31.4% (16.5%-49.2%)	Off track, higher certainty
Colombia*	21.6% (5.9%-46.6%)	24.5% (9.4%-47.3%)	28.1% (8.2%-57.6%)	30.9% (6.0%-68.8%)	Off track, lower certainty
Comoros^	7.5% (2.9%-14.1%)	8.2% (4.7%-12.8%)	10.5% (3.1%-24.2%)	12.7% (2.0%-36.4%)	Off track, lower certainty
Congo*	17.9% (4.6%-39.1%)	14.9% (5.0%-32.3%)	12.3% (2.4%-31.8%)	11.3% (1.0%-35.8%)	On track, lower certainty
Cook Islands	55.0% (39.1%-70.4%)	35.1% (26.8%-44.0%)	16.3% (9.3%-25.4%)	8.7% (2.8%-18.8%)	On track, higher certainty
Costa Rica^	34.3% (17.7%-54.5%)	37.7% (28.3%-47.7%)	41.6% (24.7%-60.5%)	44.3% (18.2%-73.3%)	Off track, higher certainty
Croatia	11.2% (5.1%-19.6%)	17.5% (11.9%-23.8%)	28.3% (18.7%-39.1%)	36.9% (20.8%-55.2%)	Off track, higher certainty
Cuba^	25.4% (11.1%-43.3%)	36.0% (25.9%-46.8%)	50.2% (35.7%-65.2%)	59.5% (36.5%-80.8%)	Off track, higher certainty
Cyprus	35.9% (21.3%-52.4%)	36.3% (27.6%-45.8%)	34.8% (23.8%-46.5%)	33.9% (17.5%-52.7%)	Off track, lower certainty
Czechia	22.6% (13.6%-33.4%)	22.1% (16.6%-28.2%)	22.6% (14.5%-32.3%)	23.1% (11.4%-38.6%)	Off track, lower certainty
Côte d'Ivoire^	19.5% (10.5%-31.7%)	17.9% (9.2%-28.9%)	15.8% (3.8%-36.3%)	14.9% (1.6%-43.1%)	On track, lower certainty
Democratic People's Republic of Korea*	17.6% (4.4%-40.7%)	20.2% (7.1%-40.8%)	24.4% (6.7%-52.6%)	27.7% (4.9%-64.7%)	Off track, lower certainty
Democratic Republic of the Congo*	16.4% (4.2%-37.6%)	14.0% (4.9%-30.5%)	12.1% (2.4%-31.7%)	11.5% (1.1%-35.1%)	On track, lower certainty
Denmark	25.8% (16.6%-36.3%)	19.7% (14.6%-25.6%)	13.1% (7.5%-20.1%)	9.8% (3.8%-18.9%)	On track, higher certainty
Djibouti*	16.6% (4.4%-37.3%)	14.5% (5.0%-30.8%)	13.6% (3.0%-34.9%)	13.5% (1.5%-40.6%)	Off track, lower certainty
Dominica^	13.3% (5.3%-24.9%)	16.3% (8.9%-26.1%)	21.4% (7.0%-42.7%)	25.4% (5.0%-58.8%)	Off track, higher certainty
Dominican Republic*	23.3% (7.3%-48.3%)	26.8% (11.3%-49.7%)	31.1% (9.9%-61.9%)	34.3% (7.4%-72.6%)	Off track, lower certainty
Ecuador^	17.0% (4.8%-37.2%)	16.6% (8.6%-27.2%)	17.6% (8.6%-29.1%)	18.9% (5.6%-38.3%)	Off track, lower certainty
Egypt^	25.1% (10.6%-44.2%)	27.4% (18.8%-37.3%)	30.0% (17.4%-44.4%)	32.3% (12.8%-56.3%)	Off track, lower certainty
El Salvador^	25.9% (9.0%-48.8%)	28.4% (16.9%-41.4%)	31.8% (16.6%-49.2%)	34.5% (12.4%-62.0%)	Off track, lower certainty
Equatorial Guinea*	19.6% (5.6%-41.8%)	17.3% (6.3%-36.1%)	15.3% (3.5%-37.8%)	14.6% (1.6%-43.3%)	On track, lower certainty
Eritrea^	9.3% (3.0%-19.5%)	7.5% (3.8%-12.8%)	7.0% (1.8%-17.0%)	7.1% (0.7%-22.9%)	Off track, lower certainty
Estonia	12.0% (6.4%-19.3%)	14.4% (10.1%-19.6%)	16.5% (10.6%-23.4%)	18.2% (9.5%-29.3%)	Off track, higher certainty

	Prevalence in 2000 (95% UI)	Prevalence in 2010 (95% UI)	Prevalence in 2022 (95% UI)	Prevalence in 2030 if trends 2010-2022 continue (95% UI)	Progress towards global target during 2010-2022
Eswatini^	19.1% (6.5%-37.7%)	16.7% (9.2%-26.5%)	15.4% (6.0%-28.5%)	15.2% (3.2%-36.5%)	Off track, lower certainty
Ethiopia^	10.1% (4.7%-18.0%)	8.0% (4.7%-12.3%)	6.6% (2.3%-13.4%)	6.0% (1.1%-16.3%)	On track, lower certainty
Fiji^	18.2% (6.2%-35.3%)	12.7% (6.7%-20.4%)	9.0% (2.4%-21.4%)	7.8% (0.7%-26.1%)	On track, lower certainty
Finland	26.9% (17.3%-38.0%)	18.7% (13.9%-24.3%)	11.1% (6.2%-17.5%)	7.6% (2.7%-15.5%)	On track, higher certainty
France	34.2% (25.4%-44.0%)	28.2% (22.7%-34.0%)	19.8% (12.2%-28.7%)	15.4% (6.6%-27.5%)	On track, higher certainty
Gabon*	22.4% (6.8%-46.5%)	19.7% (7.5%-39.3%)	17.1% (3.9%-40.6%)	16.1% (1.9%-45.8%)	On track, lower certainty
Gambia^	17.5% (6.9%-32.4%)	17.6% (10.0%-27.3%)	17.0% (5.6%-34.4%)	17.2% (2.9%-43.1%)	Off track, lower certainty
Georgia	14.1% (7.1%-23.0%)	17.9% (12.8%-24.1%)	24.2% (12.6%-38.7%)	29.1% (11.1%-53.0%)	Off track, higher certainty
Germany	19.1% (12.3%-27.4%)	16.4% (13.1%-20.1%)	12.4% (8.7%-16.8%)	10.2% (5.3%-17.0%)	On track, higher certainty
Ghana^	15.6% (6.4%-27.9%)	17.2% (11.3%-24.3%)	18.2% (7.9%-32.8%)	19.2% (5.0%-43.5%)	Off track, lower certainty
Greece	24.4% (15.2%-34.9%)	28.9% (22.2%-36.2%)	33.1% (22.8%-44.1%)	36.0% (20.7%-52.9%)	Off track, higher certainty
Grenada^	22.3% (8.8%-40.5%)	24.9% (15.4%-36.7%)	28.8% (12.5%-50.3%)	31.8% (8.4%-64.6%)	Off track, lower certainty
Guatemala^	10.5% (4.5%-19.2%)	17.0% (11.1%-24.1%)	28.4% (14.3%-46.2%)	37.5% (14.5%-65.3%)	Off track, higher certainty
Guinea*	16.9% (4.4%-38.7%)	15.4% (5.1%-33.3%)	13.7% (2.7%-34.9%)	13.2% (1.3%-40.3%)	Off track, lower certainty
Guinea-Bissau*	18.5% (5.2%-40.1%)	17.0% (6.2%-35.2%)	15.2% (3.6%-37.7%)	14.6% (1.8%-43.2%)	Off track, lower certainty
Guyana^	19.3% (6.3%-39.0%)	21.2% (11.9%-32.5%)	24.2% (12.1%-39.2%)	26.8% (8.6%-51.2%)	Off track, lower certainty
Haiti*	16.8% (4.1%-40.1%)	18.3% (6.6%-38.5%)	20.3% (5.4%-47.2%)	22.2% (3.5%-57.0%)	Off track, lower certainty
Honduras*	24.0% (7.0%-49.4%)	26.7% (10.9%-49.3%)	30.6% (9.7%-60.3%)	33.6% (7.0%-72.5%)	Off track, lower certainty
Hungary	14.7% (8.2%-23.0%)	20.0% (14.8%-26.1%)	28.1% (18.8%-38.7%)	34.3% (19.6%-51.7%)	Off track, higher certainty
Iceland*	28.8% (9.2%-55.3%)	26.7% (11.0%-48.6%)	23.9% (6.2%-51.1%)	22.8% (3.1%-57.6%)	Off track, lower certainty
India	19.6% (11.2%-29.7%)	28.9% (22.0%-36.6%)	42.0% (27.2%-57.8%)	51.2% (28.2%-73.8%)	Off track, higher certainty
Indonesia	14.0% (5.8%-26.7%)	16.8% (10.9%-24.0%)	21.5% (11.7%-33.7%)	25.4% (9.7%-46.5%)	Off track, higher certainty
Iran (Islamic Republic of)	22.2% (13.2%-32.3%)	28.2% (22.9%-33.8%)	35.9% (25.7%-47.5%)	41.6% (24.6%-60.7%)	Off track, higher certainty
Iraq^	38.6% (20.0%-60.5%)	40.5% (28.5%-52.5%)	41.9% (24.1%-60.5%)	43.1% (17.2%-70.1%)	Off track, lower certainty
Ireland	32.9% (22.8%-44.4%)	27.1% (21.8%-33.2%)	19.7% (13.4%-27.0%)	15.7% (8.0%-26.0%)	On track, higher certainty
Israel*	30.2% (10.6%-56.5%)	27.8% (11.9%-51.4%)	24.1% (6.3%-52.2%)	22.4% (3.1%-57.3%)	On track, lower certainty
Italy	36.9% (25.7%-49.1%)	37.7% (30.4%-45.5%)	36.4% (25.6%-48.4%)	35.6% (20.3%-52.9%)	Off track, lower certainty
Jamaica^	23.3% (11.1%-38.6%)	26.9% (16.3%-39.5%)	31.3% (11.5%-56.4%)	34.5% (7.8%-69.6%)	Off track, lower certainty
Japan^	26.2% (7.9%-54.0%)	31.8% (17.9%-49.4%)	40.0% (26.1%-54.7%)	45.7% (24.3%-67.9%)	Off track, higher certainty
Jordan	26.6% (16.4%-38.4%)	29.0% (21.9%-36.6%)	30.0% (17.9%-43.7%)	31.0% (14.1%-52.0%)	Off track, lower certainty
Kazakhstan^	18.8% (9.6%-30.6%)	22.3% (12.3%-35.1%)	28.7% (9.0%-54.8%)	33.5% (6.7%-69.7%)	Off track, higher certainty

	Prevalence in 2000 (95% UI)	Prevalence in 2010 (95% UI)	Prevalence in 2022 (95% UI)	Prevalence in 2030 if trends 2010-2022 continue (95% UI)	Progress towards global target during 2010-2022
Kenya^	11.0% (5.0%-19.5%)	9.5% (5.7%-14.6%)	8.5% (3.1%-16.8%)	8.1% (1.6%-20.8%)	Off track, lower certainty
Kiribati^	38.0% (19.3%-59.0%)	29.7% (19.3%-41.5%)	21.2% (9.7%-36.8%)	17.2% (4.0%-39.5%)	On track, higher certainty
Kuwait^	58.3% (38.2%-77.5%)	61.1% (47.8%-73.7%)	61.7% (37.3%-82.7%)	61.9% (26.7%-89.6%)	Off track, lower certainty
Kyrgyzstan^	14.2% (4.4%-29.8%)	15.0% (7.9%-24.0%)	17.3% (6.5%-32.7%)	19.7% (4.2%-44.7%)	Off track, lower certainty
Lao People's Democratic Republic^	12.3% (5.9%-21.2%)	10.4% (6.3%-15.8%)	9.1% (2.7%-19.2%)	8.7% (1.1%-24.2%)	On track, lower certainty
Latvia	17.2% (8.5%-28.9%)	16.5% (11.1%-22.7%)	15.4% (8.7%-23.2%)	15.0% (5.8%-27.6%)	Off track, lower certainty
Lebanon^	38.7% (21.8%-57.3%)	48.2% (38.0%-58.6%)	57.8% (40.8%-73.9%)	63.7% (38.4%-85.1%)	Off track, higher certainty
Lesotho^	12.0% (3.6%-25.2%)	9.8% (4.8%-16.7%)	8.6% (2.5%-19.2%)	8.4% (1.1%-25.2%)	Off track, lower certainty
Liberia^	27.3% (12.6%-47.1%)	20.1% (12.4%-29.2%)	13.6% (7.4%-21.9%)	10.4% (3.2%-21.5%)	On track, higher certainty
Libya^	29.6% (15.3%-46.5%)	33.5% (23.9%-43.9%)	37.5% (26.3%-49.9%)	40.4% (23.4%-59.0%)	Off track, higher certainty
Lithuania	23.1% (14.9%-33.1%)	22.1% (17.3%-27.7%)	20.7% (13.5%-29.4%)	20.1% (9.9%-33.6%)	Off track, lower certainty
Luxembourg	24.5% (15.8%-35.1%)	19.8% (15.0%-25.3%)	13.9% (8.3%-20.8%)	10.9% (4.4%-20.0%)	On track, higher certainty
Madagascar*	14.8% (3.6%-34.4%)	13.0% (4.3%-28.5%)	12.5% (2.7%-33.8%)	12.7% (1.5%-40.3%)	Off track, lower certainty
Malawi	11.4% (5.8%-19.0%)	5.7% (3.5%-8.5%)	2.4% (0.7%-5.4%)	1.4% (0.2%-4.6%)	On track, higher certainty
Malaysia	35.1% (25.5%-46.0%)	37.1% (31.0%-43.3%)	37.4% (25.6%-49.9%)	37.7% (20.5%-56.6%)	Off track, lower certainty
Maldives^	21.1% (7.2%-41.1%)	22.7% (13.0%-34.5%)	25.3% (14.7%-37.7%)	27.5% (11.8%-47.1%)	Off track, lower certainty
Mali^	32.6% (14.5%-55.4%)	29.9% (15.4%-47.6%)	25.9% (7.9%-50.8%)	23.9% (3.7%-57.1%)	On track, lower certainty
Malta	50.5% (34.2%-67.2%)	45.5% (35.5%-55.6%)	37.8% (24.5%-52.2%)	33.1% (15.0%-54.2%)	On track, lower certainty
Marshall Islands^	36.0% (17.9%-56.3%)	25.7% (12.3%-42.1%)	16.9% (3.2%-40.0%)	13.1% (0.9%-41.8%)	On track, higher certainty
Mauritania^	51.8% (35.8%-68.0%)	42.2% (27.4%-57.7%)	30.5% (8.8%-58.0%)	24.6% (2.8%-59.4%)	On track, higher certainty
Mauritius^	25.5% (14.5%-38.6%)	25.7% (18.2%-34.2%)	24.9% (15.0%-37.3%)	24.4% (11.4%-42.1%)	Off track, lower certainty
Mexico	21.8% (14.3%-30.4%)	23.3% (18.6%-28.6%)	25.3% (17.7%-33.9%)	26.8% (15.2%-40.2%)	Off track, higher certainty
Micronesia (Federated States of)	39.5% (22.0%-57.9%)	30.9% (20.0%-43.3%)	22.9% (8.6%-43.0%)	19.1% (3.6%-46.4%)	On track, lower certainty
Monaco*	29.8% (9.9%-56.8%)	27.0% (11.0%-50.1%)	23.3% (6.1%-51.0%)	21.4% (3.0%-55.3%)	On track, lower certainty
Mongolia	7.5% (3.1%-13.9%)	13.8% (9.8%-18.5%)	25.8% (15.7%-38.2%)	36.2% (17.7%-58.4%)	Off track, higher certainty
Montenegro*	16.3% (4.1%-38.5%)	18.2% (6.3%-38.8%)	21.6% (5.3%-48.2%)	24.4% (3.9%-59.4%)	Off track, lower certainty
Morocco^	15.5% (5.0%-30.7%)	17.3% (9.9%-26.3%)	19.8% (10.1%-32.5%)	22.2% (7.2%-43.4%)	Off track, lower certainty
Mozambique^	6.8% (2.6%-13.9%)	6.9% (3.0%-12.6%)	8.2% (1.6%-22.3%)	9.6% (0.8%-32.8%)	Off track, lower certainty
Myanmar	18.5% (10.3%-28.6%)	19.2% (13.3%-26.0%)	20.1% (9.2%-35.1%)	21.0% (6.0%-43.5%)	Off track, lower certainty
Namibia^	31.5% (19.3%-46.1%)	26.4% (15.3%-39.8%)	21.0% (5.5%-44.1%)	18.4% (2.1%-48.2%)	On track, lower certainty
Nauru^	43.2% (27.8%-59.5%)	35.1% (26.0%-44.5%)	27.8% (13.4%-45.6%)	24.0% (6.6%-49.3%)	On track, lower certainty

	Prevalence in 2000 (95% UI)	Prevalence in 2010 (95% UI)	Prevalence in 2022 (95% UI)	Prevalence in 2030 if trends 2010-2022 continue (95% UI)	Progress towards global target during 2010-2022
Nepal	9.2% (4.3%-16.0%)	9.6% (6.3%-13.7%)	10.4% (5.1%-17.7%)	11.4% (3.7%-23.7%)	Off track, lower certainty
Netherlands	19.2% (12.4%-27.2%)	13.8% (10.4%-17.7%)	8.1% (5.1%-11.9%)	5.5% (2.5%-10.1%)	On track, higher certainty
New Zealand	16.9% (9.8%-25.8%)	18.0% (13.3%-23.5%)	17.8% (12.2%-24.6%)	17.9% (9.9%-28.2%)	Off track, lower certainty
Nicaragua*	27.2% (9.0%-53.1%)	29.7% (13.1%-51.8%)	32.9% (10.9%-62.5%)	35.4% (7.9%-72.3%)	Off track, lower certainty
Niger^	19.8% (7.6%-37.3%)	16.7% (9.3%-25.9%)	13.0% (6.7%-21.6%)	11.1% (3.7%-22.8%)	On track, lower certainty
Nigeria*	18.8% (5.1%-41.4%)	17.7% (6.5%-36.8%)	15.5% (3.3%-37.4%)	14.7% (1.6%-42.4%)	On track, lower certainty
Niue^	18.2% (5.1%-37.2%)	11.0% (5.1%-19.1%)	6.3% (1.3%-16.7%)	4.9% (0.3%-19.9%)	On track, higher certainty
Norway	29.9% (17.9%-43.6%)	31.5% (21.4%-42.9%)	31.8% (14.4%-53.9%)	32.3% (9.5%-63.2%)	Off track, lower certainty
occupied Palestinian territory, including east Jerusalem^	32.9% (17.2%-52.4%)	30.6% (21.1%-41.2%)	26.1% (16.5%-37.3%)	23.8% (10.3%-40.6%)	On track, lower certainty
Oman^	28.7% (14.9%-45.6%)	31.8% (23.0%-41.3%)	35.0% (21.2%-50.8%)	37.5% (16.6%-62.0%)	Off track, higher certainty
Pakistan^	18.5% (9.8%-29.8%)	24.5% (16.7%-32.7%)	34.1% (17.9%-52.7%)	41.2% (16.5%-68.5%)	Off track, higher certainty
Palau^	32.0% (13.1%-54.6%)	23.9% (14.9%-34.3%)	16.6% (6.8%-31.9%)	13.5% (2.4%-36.5%)	On track, lower certainty
Panama^	30.9% (10.8%-56.3%)	38.2% (23.8%-54.3%)	48.3% (33.5%-63.4%)	55.1% (32.8%-77.3%)	Off track, higher certainty
Papua New Guinea^	17.3% (7.3%-31.1%)	11.5% (6.0%-19.1%)	8.2% (1.5%-22.9%)	7.1% (0.4%-28.5%)	On track, lower certainty
Paraguay^	22.3% (12.4%-35.2%)	26.0% (14.8%-39.3%)	32.1% (11.4%-58.9%)	36.4% (8.8%-72.9%)	Off track, higher certainty
Peru^	22.1% (6.8%-43.6%)	25.5% (14.2%-38.8%)	32.0% (18.0%-47.4%)	37.0% (15.9%-61.6%)	Off track, higher certainty
Philippines	11.9% (5.2%-20.9%)	20.7% (14.9%-27.2%)	35.8% (21.3%-52.5%)	47.2% (23.0%-72.8%)	Off track, higher certainty
Poland	19.2% (9.8%-30.5%)	26.1% (19.0%-33.7%)	36.0% (25.1%-47.8%)	43.1% (25.6%-61.7%)	Off track, higher certainty
Portugal	38.1% (26.6%-50.2%)	42.6% (34.4%-50.7%)	46.0% (34.1%-58.7%)	48.2% (30.8%-66.2%)	Off track, higher certainty
Puerto Rico*	30.4% (10.4%-58.2%)	33.4% (15.6%-57.7%)	36.6% (13.7%-66.0%)	39.0% (9.9%-75.2%)	Off track, lower certainty
Qatar^	41.6% (24.1%-61.2%)	46.2% (33.5%-58.9%)	50.3% (26.9%-73.1%)	52.9% (20.0%-83.5%)	Off track, higher certainty
Republic of Korea	21.5% (12.8%-32.3%)	35.2% (29.7%-40.8%)	55.9% (48.2%-63.4%)	68.8% (55.3%-80.6%)	Off track, higher certainty
Republic of Moldova^	14.1% (4.8%-29.1%)	13.3% (7.6%-20.6%)	12.1% (6.6%-19.5%)	11.9% (4.0%-24.0%)	Off track, lower certainty
Republic of North Macedonia^	16.4% (4.6%-35.6%)	17.6% (7.7%-31.6%)	20.5% (6.9%-39.7%)	23.2% (4.6%-52.1%)	Off track, lower certainty
Romania	16.2% (5.4%-31.9%)	23.9% (14.9%-34.4%)	37.0% (26.2%-49.1%)	46.8% (28.2%-67.0%)	Off track, higher certainty
Russian Federation^	10.3% (5.2%-17.5%)	13.4% (7.7%-20.6%)	18.1% (5.7%-38.3%)	22.0% (4.1%-53.9%)	Off track, higher certainty
Rwanda^	16.3% (6.0%-31.8%)	11.2% (6.3%-17.6%)	7.7% (4.0%-12.9%)	6.1% (1.8%-13.3%)	On track, higher certainty
Saint Kitts and Nevis^	26.1% (10.7%-46.5%)	29.9% (15.9%-46.7%)	34.1% (12.6%-60.3%)	37.1% (9.1%-72.3%)	Off track, lower certainty
Saint Lucia^	17.7% (5.5%-36.9%)	17.6% (9.5%-28.3%)	17.8% (9.4%-27.6%)	18.5% (6.3%-34.9%)	Off track, lower certainty
Saint Vincent and the Grenadines^	18.2% (6.3%-36.0%)	19.4% (11.4%-29.2%)	21.4% (9.4%-37.1%)	23.3% (6.1%-48.8%)	Off track, lower certainty
Samoa^	28.7% (17.3%-41.8%)	17.9% (11.5%-25.5%)	9.3% (2.8%-20.1%)	6.1% (0.7%-18.4%)	On track, higher certainty

	Prevalence in 2000 (95% UI)	Prevalence in 2010 (95% UI)	Prevalence in 2022 (95% UI)	Prevalence in 2030 if trends 2010-2022 continue (95% UI)	Progress towards global target during 2010-2022
San Marino*	29.8% (10.6%-56.8%)	26.8% (11.1%-48.6%)	22.9% (6.4%-49.8%)	20.9% (3.3%-53.9%)	On track, lower certainty
Saudi Arabia	50.7% (35.6%-65.4%)	49.4% (41.8%-56.9%)	46.7% (32.3%-61.2%)	45.1% (24.1%-67.8%)	Off track, lower certainty
Senegal^	14.6% (4.4%-30.0%)	12.8% (6.7%-21.0%)	10.9% (4.3%-21.2%)	10.3% (2.1%-26.2%)	On track, lower certainty
Serbia^	40.8% (24.7%-58.7%)	40.8% (27.7%-54.8%)	41.7% (16.4%-67.9%)	42.5% (9.5%-78.3%)	Off track, lower certainty
Seychelles^	23.0% (12.9%-35.0%)	21.9% (14.9%-30.1%)	19.7% (8.4%-36.1%)	18.8% (4.7%-42.0%)	Off track, lower certainty
Sierra Leone^	12.9% (4.9%-25.0%)	11.0% (5.6%-18.0%)	8.6% (2.1%-20.3%)	7.7% (0.7%-24.8%)	On track, lower certainty
Singapore	25.0% (13.7%-39.2%)	23.8% (18.0%-30.3%)	24.2% (15.8%-34.0%)	24.6% (11.2%-41.5%)	Off track, lower certainty
Slovakia	25.3% (10.9%-46.0%)	23.5% (15.0%-33.6%)	22.5% (13.7%-32.8%)	22.2% (9.2%-39.2%)	Off track, lower certainty
Slovenia	16.6% (8.1%-28.1%)	16.8% (11.4%-23.2%)	18.4% (11.3%-27.4%)	19.6% (8.7%-34.3%)	Off track, lower certainty
Solomon Islands^	33.6% (19.2%-50.1%)	22.1% (15.1%-30.1%)	12.7% (4.9%-24.7%)	8.8% (1.4%-24.0%)	On track, higher certainty
Somalia*	16.7% (4.5%-38.3%)	14.6% (5.0%-31.8%)	13.5% (2.8%-34.8%)	13.4% (1.3%-40.8%)	Off track, lower certainty
South Africa	45.2% (34.1%-56.2%)	45.4% (37.3%-53.8%)	41.2% (24.4%-59.7%)	38.6% (16.3%-65.3%)	Off track, lower certainty
South Sudan*	16.5% (4.0%-37.3%)	14.4% (4.7%-31.9%)	13.3% (2.7%-36.0%)	13.1% (1.4%-42.4%)	Off track, lower certainty
Spain	36.0% (25.6%-47.6%)	29.5% (23.9%-35.8%)	19.4% (12.7%-27.2%)	14.2% (6.6%-24.7%)	On track, higher certainty
Sri Lanka	14.8% (8.2%-23.1%)	20.4% (15.1%-26.8%)	28.7% (18.8%-40.2%)	35.1% (19.4%-53.3%)	Off track, higher certainty
Sudan^	19.7% (6.6%-38.8%)	17.7% (9.7%-27.9%)	17.2% (8.0%-30.0%)	17.3% (4.8%-37.7%)	Off track, lower certainty
Suriname^	31.1% (13.2%-53.6%)	36.2% (24.2%-49.4%)	42.4% (23.6%-62.9%)	46.6% (18.3%-76.1%)	Off track, higher certainty
Sweden	26.1% (16.7%-36.8%)	17.5% (12.8%-22.8%)	9.3% (5.1%-14.9%)	5.8% (2.0%-12.1%)	On track, higher certainty
Switzerland^	26.3% (9.7%-46.4%)	22.2% (12.5%-33.6%)	17.6% (7.6%-31.9%)	15.6% (3.7%-37.3%)	On track, lower certainty
Syrian Arab Republic*	27.0% (8.8%-54.0%)	29.8% (12.9%-52.6%)	33.1% (10.6%-62.5%)	35.6% (7.8%-72.7%)	Off track, lower certainty
São Tomé and Príncipe^	13.0% (4.6%-24.8%)	13.6% (8.3%-20.3%)	14.1% (6.9%-24.1%)	14.9% (4.6%-32.4%)	Off track, lower certainty
Taiwan, China*	20.1% (5.2%-42.9%)	22.9% (8.8%-43.2%)	27.2% (8.1%-56.1%)	30.5% (6.2%-67.7%)	Off track, higher certainty
Tajikistan^	20.2% (6.5%-40.6%)	22.3% (12.3%-34.2%)	26.3% (13.8%-41.6%)	29.6% (10.7%-54.4%)	Off track, higher certainty
Thailand	14.6% (7.2%-24.2%)	20.0% (15.5%-25.2%)	28.2% (21.1%-36.1%)	34.5% (20.9%-50.1%)	Off track, higher certainty
Timor-Leste^	13.5% (3.3%-30.9%)	16.0% (6.9%-28.7%)	20.9% (7.1%-40.9%)	24.9% (5.7%-55.6%)	Off track, higher certainty
Togo^	13.3% (4.7%-26.2%)	12.9% (7.4%-19.6%)	12.2% (6.7%-19.6%)	12.0% (4.5%-24.1%)	Off track, lower certainty
Tonga	27.2% (12.1%-45.4%)	24.1% (16.5%-32.6%)	22.6% (11.8%-37.0%)	22.2% (6.9%-46.4%)	Off track, lower certainty
Trinidad and Tobago^	29.3% (13.5%-49.2%)	31.7% (21.2%-43.8%)	34.6% (16.4%-56.3%)	36.8% (11.2%-69.0%)	Off track, lower certainty
Tunisia^	19.1% (10.4%-30.3%)	23.3% (13.2%-35.5%)	28.6% (9.6%-53.1%)	32.6% (6.8%-67.3%)	Off track, higher certainty
Turkmenistan^	14.3% (3.7%-32.6%)	12.3% (5.7%-21.1%)	10.8% (4.5%-19.5%)	10.6% (2.5%-24.6%)	Off track, lower certainty
Tuvalu^	33.2% (12.6%-58.0%)	24.1% (13.9%-36.4%)	16.2% (6.8%-30.1%)	12.8% (2.4%-33.9%)	On track, higher certainty

	Prevalence in 2000 (95% UI)	Prevalence in 2010 (95% UI)	Prevalence in 2022 (95% UI)	Prevalence in 2030 if trends 2010-2022 continue (95% UI)	Progress towards global target during 2010-2022
Türkiye	29.4% (19.4%-41.1%)	32.4% (24.6%-40.7%)	35.1% (20.8%-51.3%)	37.2% (16.4%-60.4%)	Off track, higher certainty
Uganda^	8.9% (2.4%-20.1%)	6.8% (3.3%-11.7%)	6.0% (3.1%-10.1%)	5.8% (1.8%-12.9%)	On track, lower certainty
Ukraine^	10.0% (4.4%-18.0%)	11.2% (6.8%-16.7%)	12.8% (6.1%-22.2%)	14.2% (4.7%-29.3%)	Off track, lower certainty
United Arab Emirates	42.4% (29.3%-56.3%)	53.1% (44.4%-61.7%)	63.1% (47.7%-76.9%)	69.1% (47.1%-86.7%)	Off track, higher certainty
United Kingdom	33.0% (22.5%-44.5%)	26.7% (21.0%-32.6%)	18.2% (12.3%-25.0%)	13.8% (6.9%-23.4%)	On track, higher certainty
United Republic of Tanzania^	12.0% (4.0%-24.8%)	7.7% (4.0%-12.8%)	4.8% (2.2%-8.5%)	3.5% (0.9%-8.6%)	On track, higher certainty
United States of America	24.6% (15.2%-34.8%)	26.8% (22.0%-31.8%)	27.6% (18.7%-37.9%)	28.3% (14.8%-45.4%)	Off track, lower certainty
Uruguay	26.4% (15.7%-38.9%)	27.8% (18.2%-38.5%)	30.8% (12.5%-52.2%)	33.0% (8.4%-63.6%)	Off track, lower certainty
Uzbekistan	8.2% (3.6%-15.0%)	14.2% (9.8%-19.7%)	26.0% (16.2%-38.1%)	36.1% (19.0%-56.4%)	Off track, higher certainty
Vanuatu^	15.9% (5.0%-31.7%)	9.6% (4.7%-16.1%)	5.8% (1.3%-15.2%)	4.6% (0.3%-18.0%)	On track, higher certainty
Venezuela (Bolivarian Republic of)^	32.7% (13.6%-56.4%)	36.2% (23.5%-50.1%)	38.5% (22.5%-55.9%)	40.3% (17.1%-66.6%)	Off track, lower certainty
Viet Nam	14.0% (7.5%-22.4%)	18.6% (13.6%-24.0%)	25.6% (16.5%-36.1%)	31.1% (16.1%-48.4%)	Off track, higher certainty
Yemen*	17.6% (4.5%-41.1%)	19.8% (7.4%-40.1%)	24.0% (6.7%-51.9%)	27.4% (5.1%-63.4%)	Off track, higher certainty
Zambia^	14.6% (7.5%-24.2%)	11.8% (7.4%-17.3%)	9.6% (4.0%-17.8%)	8.6% (2.0%-20.3%)	On track, lower certainty
Zimbabwe^	15.4% (7.7%-25.6%)	13.7% (6.9%-22.9%)	13.3% (3.1%-31.3%)	13.5% (1.5%-40.4%)	Off track, lower certainty

Appendix 16. Age-standardised prevalence of insufficient physical activity among adults aged 18 years and over in 2000, 2010 and 2022, projected prevalence in 2030 assuming trends 2010-2022 continue, and progress during 2010-2022 toward the global target of reducing the prevalence of insufficient physical activity by 15% between 2010 and 2030, by country for women.

Notes: UI: Uncertainty interval. * indicates no surveys from the country were included; ^ indicates 1 or 2 surveys from the country were included.

	Prevalence in 2000 (95% UI)	Prevalence in 2010 (95% UI)	Prevalence in 2022 (95% UI)	Prevalence in 2030 if trends 2010-2022 continue (95% UI)	Progress towards global target during 2010-2022
Afghanistan^	28.9% (8.0%-58.3%)	35.4% (20.0%-53.4%)	46.0% (28.5%-63.8%)	53.3% (26.1%-80.0%)	Off track, higher certainty
Albania*	24.4% (5.1%-56.3%)	24.7% (7.4%-51.6%)	27.1% (5.1%-61.9%)	29.4% (2.7%-75.4%)	Off track, lower certainty
Algeria^	30.7% (9.6%-57.9%)	33.5% (19.3%-49.6%)	38.2% (21.4%-57.2%)	41.7% (16.0%-71.2%)	Off track, lower certainty
Andorra^	38.4% (21.8%-57.0%)	33.1% (18.9%-49.6%)	28.3% (6.9%-60.8%)	26.2% (2.4%-70.3%)	On track, lower certainty
Angola*	25.5% (5.8%-55.8%)	22.6% (6.7%-46.3%)	20.4% (3.1%-52.4%)	20.0% (1.2%-61.3%)	Off track, lower certainty
Antigua and Barbuda*	34.4% (9.9%-68.0%)	38.9% (15.3%-66.7%)	44.8% (12.7%-79.8%)	48.5% (8.5%-89.0%)	Off track, lower certainty
Argentina	35.4% (17.1%-57.9%)	37.1% (26.5%-48.8%)	40.1% (24.9%-56.2%)	42.3% (18.7%-67.6%)	Off track, lower certainty
Armenia^	25.3% (7.2%-52.0%)	24.9% (13.7%-39.2%)	25.3% (11.5%-43.3%)	26.4% (6.7%-55.2%)	Off track, lower certainty
Australia	31.0% (17.4%-46.3%)	29.4% (20.7%-38.7%)	27.2% (17.8%-38.1%)	26.0% (13.3%-42.1%)	Off track, lower certainty
Austria	36.5% (24.7%-49.1%)	28.5% (22.2%-35.4%)	19.9% (13.9%-27.0%)	15.3% (8.0%-25.1%)	On track, higher certainty
Azerbaijan^	25.2% (7.1%-53.4%)	23.8% (12.6%-38.5%)	22.8% (10.7%-38.5%)	23.1% (5.9%-48.4%)	Off track, lower certainty
Bahamas^	45.6% (21.7%-71.6%)	46.1% (33.1%-59.6%)	46.6% (30.6%-63.3%)	47.1% (20.7%-73.2%)	Off track, lower certainty
Bahrain*	34.7% (10.1%-66.3%)	38.2% (15.7%-65.2%)	42.8% (11.5%-77.8%)	45.9% (7.9%-87.8%)	Off track, lower certainty
Bangladesh	32.8% (19.2%-48.9%)	26.9% (19.8%-35.1%)	20.9% (12.3%-31.9%)	17.9% (6.8%-33.7%)	On track, lower certainty
Barbados^	40.2% (21.4%-60.4%)	45.3% (30.6%-60.8%)	51.3% (21.0%-80.9%)	54.8% (13.8%-91.4%)	Off track, higher certainty
Belarus^	21.6% (5.4%-49.1%)	17.4% (8.9%-29.0%)	13.5% (7.0%-22.1%)	12.2% (3.0%-27.3%)	On track, lower certainty
Belgium	52.9% (42.8%-63.0%)	41.2% (34.9%-47.8%)	28.4% (18.9%-39.6%)	21.3% (10.2%-36.1%)	On track, higher certainty
Belize*	37.3% (11.4%-69.9%)	42.1% (17.5%-69.3%)	47.9% (14.7%-82.1%)	51.3% (11.3%-91.4%)	Off track, lower certainty
Benin^	18.6% (4.9%-38.4%)	19.1% (10.7%-29.7%)	20.3% (8.3%-37.8%)	21.9% (4.9%-51.7%)	Off track, lower certainty
Bhutan^	17.0% (4.4%-38.8%)	13.4% (6.9%-22.3%)	11.0% (5.0%-19.0%)	10.4% (2.0%-25.3%)	On track, lower certainty
Bolivia (Plurinational State of)^	26.3% (6.7%-56.5%)	25.8% (13.1%-42.5%)	26.6% (14.2%-41.8%)	28.0% (9.1%-52.9%)	Off track, lower certainty
Bosnia and Herzegovina^	19.3% (9.0%-32.9%)	20.5% (9.8%-35.6%)	23.7% (4.4%-57.1%)	26.3% (2.0%-72.0%)	Off track, lower certainty
Botswana^	34.0% (16.7%-54.2%)	30.6% (21.4%-40.6%)	26.8% (11.5%-47.0%)	25.2% (5.4%-56.0%)	On track, lower certainty
Brazil	32.9% (20.0%-48.1%)	37.6% (29.4%-46.7%)	45.1% (31.6%-59.2%)	50.1% (29.6%-71.3%)	Off track, higher certainty
Brunei Darussalam^	29.9% (9.6%-57.4%)	32.7% (20.2%-47.6%)	37.4% (19.3%-57.8%)	41.0% (13.1%-73.0%)	Off track, lower certainty
Bulgaria	22.8% (11.0%-37.5%)	26.7% (18.8%-35.3%)	32.1% (21.0%-44.3%)	36.2% (18.9%-55.7%)	Off track, higher certainty
Burkina Faso	16.2% (7.9%-27.5%)	17.5% (11.9%-24.2%)	19.3% (10.8%-30.0%)	20.8% (8.3%-37.8%)	Off track, lower certainty

	Prevalence in 2000 (95% UI)	Prevalence in 2010 (95% UI)	Prevalence in 2022 (95% UI)	Prevalence in 2030 if trends 2010-2022 continue (95% UI)	Progress towards global target during 2010-2022
Burundi*	18.6% (3.6%-47.6%)	16.0% (3.9%-36.6%)	14.5% (1.6%-41.3%)	14.6% (0.5%-50.0%)	Off track, lower certainty
Cabo Verde^	26.4% (11.9%-45.1%)	32.0% (21.9%-43.4%)	39.5% (24.9%-55.7%)	44.7% (22.0%-69.6%)	Off track, higher certainty
Cambodia^	14.9% (5.5%-29.6%)	13.8% (8.1%-21.7%)	13.7% (7.6%-22.0%)	14.0% (5.1%-27.7%)	Off track, lower certainty
Cameroon*	26.2% (6.3%-57.7%)	23.8% (7.5%-48.2%)	21.4% (3.2%-53.2%)	20.7% (1.2%-61.2%)	Off track, lower certainty
Canada	27.9% (15.4%-42.3%)	32.9% (24.7%-41.7%)	38.6% (27.7%-49.7%)	42.6% (25.4%-60.2%)	Off track, higher certainty
Central African Republic*	22.3% (4.6%-50.6%)	20.2% (5.6%-42.6%)	19.1% (2.6%-49.1%)	19.3% (1.1%-59.1%)	Off track, lower certainty
Chad^	25.5% (13.7%-39.5%)	22.0% (10.6%-36.9%)	19.1% (3.2%-48.0%)	18.2% (1.0%-57.4%)	On track, lower certainty
Chile	24.9% (8.9%-46.0%)	33.3% (23.4%-44.0%)	45.9% (30.3%-62.3%)	54.4% (28.1%-80.3%)	Off track, higher certainty
China	19.2% (11.4%-29.0%)	18.9% (14.9%-23.2%)	19.5% (11.6%-28.8%)	20.2% (8.4%-36.1%)	Off track, lower certainty
Colombia*	30.6% (7.8%-65.7%)	34.6% (12.5%-62.5%)	40.5% (10.0%-76.3%)	44.6% (7.2%-87.1%)	Off track, lower certainty
Comoros^	11.9% (4.9%-22.6%)	16.2% (10.0%-24.3%)	23.7% (7.7%-48.0%)	29.7% (5.6%-68.1%)	Off track, higher certainty
Congo*	25.5% (5.6%-56.2%)	22.4% (6.3%-47.5%)	19.5% (2.7%-50.7%)	18.6% (1.0%-59.0%)	On track, lower certainty
Cook Islands	60.0% (43.7%-75.0%)	47.6% (37.9%-57.2%)	33.1% (21.1%-46.6%)	25.1% (10.5%-43.8%)	On track, higher certainty
Costa Rica^	49.2% (26.0%-73.6%)	53.3% (42.1%-64.7%)	57.7% (34.9%-78.1%)	60.2% (25.1%-89.0%)	Off track, lower certainty
Croatia	13.9% (6.2%-24.3%)	19.6% (13.4%-26.7%)	28.6% (18.6%-40.5%)	35.7% (19.1%-55.7%)	Off track, higher certainty
Cuba^	40.3% (18.8%-63.5%)	55.2% (42.4%-67.6%)	72.0% (56.7%-84.5%)	80.4% (57.7%-94.6%)	Off track, higher certainty
Cyprus	48.9% (30.9%-66.6%)	48.2% (37.9%-58.5%)	47.0% (33.3%-61.1%)	46.2% (25.8%-68.0%)	Off track, lower certainty
Czechia	25.6% (15.0%-38.3%)	24.4% (18.2%-31.2%)	24.1% (15.1%-34.6%)	24.2% (11.1%-41.0%)	Off track, lower certainty
Côte d'Ivoire^	30.8% (17.2%-46.5%)	26.9% (13.6%-43.4%)	23.7% (4.3%-55.7%)	22.5% (1.4%-65.1%)	On track, lower certainty
Democratic People's Republic of Korea*	22.9% (5.5%-51.9%)	25.0% (8.1%-49.2%)	29.4% (5.8%-63.6%)	32.8% (3.6%-76.9%)	Off track, lower certainty
Democratic Republic of the Congo*	20.8% (4.1%-47.7%)	18.6% (4.8%-40.0%)	17.5% (2.3%-45.6%)	17.6% (0.9%-55.1%)	Off track, lower certainty
Denmark	27.2% (16.9%-38.8%)	18.8% (13.6%-24.9%)	11.2% (6.0%-18.5%)	7.8% (2.6%-16.5%)	On track, higher certainty
Djibouti*	23.8% (5.4%-54.4%)	21.4% (6.2%-45.3%)	20.2% (2.8%-51.3%)	20.3% (1.1%-60.3%)	Off track, lower certainty
Dominica^	26.4% (11.7%-45.5%)	31.8% (18.7%-46.3%)	39.4% (12.7%-70.7%)	44.4% (8.2%-85.5%)	Off track, higher certainty
Dominican Republic*	31.7% (8.4%-62.8%)	36.5% (13.9%-64.0%)	42.9% (11.8%-79.7%)	47.1% (8.2%-89.5%)	Off track, higher certainty
Ecuador^	26.6% (7.0%-55.5%)	26.4% (13.9%-41.9%)	27.8% (14.6%-44.4%)	29.5% (9.4%-56.8%)	Off track, lower certainty
Egypt^	35.8% (16.0%-59.9%)	36.4% (25.5%-48.1%)	37.4% (21.4%-55.3%)	38.5% (14.0%-67.4%)	Off track, lower certainty
El Salvador^	34.2% (11.3%-63.1%)	39.1% (24.2%-55.3%)	45.7% (24.9%-67.1%)	50.1% (19.1%-82.0%)	Off track, higher certainty
Equatorial Guinea*	29.3% (7.8%-61.1%)	26.6% (9.1%-52.1%)	24.3% (4.4%-58.0%)	23.7% (1.9%-67.4%)	Off track, lower certainty
Eritrea^	18.8% (6.1%-37.4%)	16.6% (8.9%-26.5%)	16.1% (3.7%-38.0%)	16.8% (1.4%-51.7%)	Off track, lower certainty
Estonia	12.5% (6.6%-20.1%)	13.9% (9.8%-18.9%)	15.4% (9.7%-22.2%)	16.6% (7.9%-27.5%)	Off track, higher certainty

	Prevalence in 2000 (95% UI)	Prevalence in 2010 (95% UI)	Prevalence in 2022 (95% UI)	Prevalence in 2030 if trends 2010-2022 continue (95% UI)	Progress towards global target during 2010-2022
Eswatini^	29.7% (9.8%-56.7%)	26.0% (14.7%-39.5%)	22.6% (8.5%-41.7%)	21.6% (3.5%-52.4%)	On track, lower certainty
Ethiopia^	18.2% (8.8%-30.5%)	14.8% (9.3%-21.7%)	12.1% (4.3%-24.0%)	11.0% (1.9%-28.1%)	On track, lower certainty
Fiji^	30.2% (11.4%-55.6%)	25.3% (14.9%-38.1%)	21.8% (6.2%-46.0%)	20.8% (2.1%-56.9%)	On track, lower certainty
Finland	27.9% (17.8%-39.8%)	16.8% (12.1%-22.2%)	8.1% (4.0%-14.1%)	4.7% (1.3%-11.1%)	On track, higher certainty
France	43.3% (32.6%-54.2%)	35.7% (29.5%-42.1%)	26.3% (16.5%-37.5%)	21.1% (9.3%-37.1%)	On track, higher certainty
Gabon*	28.8% (7.5%-59.5%)	26.4% (8.7%-52.6%)	24.6% (4.2%-58.1%)	24.3% (1.8%-68.1%)	Off track, lower certainty
Gambia^	27.0% (10.3%-48.7%)	25.9% (15.2%-38.5%)	25.1% (8.2%-50.0%)	25.4% (3.5%-62.9%)	Off track, lower certainty
Georgia	16.2% (8.1%-27.2%)	19.3% (13.7%-25.8%)	23.8% (11.9%-39.1%)	27.4% (9.3%-52.5%)	Off track, higher certainty
Germany	21.5% (13.5%-31.4%)	16.7% (13.1%-20.7%)	11.6% (7.8%-16.2%)	9.1% (4.3%-15.6%)	On track, higher certainty
Ghana^	18.4% (6.7%-35.3%)	20.7% (13.6%-29.5%)	24.0% (9.8%-43.0%)	26.9% (6.0%-58.0%)	Off track, lower certainty
Greece	33.0% (21.5%-45.7%)	35.1% (27.6%-43.4%)	37.3% (25.6%-50.2%)	39.0% (21.4%-58.4%)	Off track, higher certainty
Grenada^	32.2% (12.8%-57.1%)	36.7% (23.7%-50.7%)	42.9% (18.5%-70.4%)	47.0% (12.2%-84.4%)	Off track, lower certainty
Guatemala^	10.7% (4.2%-20.6%)	22.8% (15.0%-31.5%)	44.8% (24.5%-66.2%)	60.0% (28.9%-87.2%)	Off track, higher certainty
Guinea*	22.0% (4.6%-52.3%)	20.2% (5.6%-42.7%)	19.4% (2.7%-48.9%)	19.6% (1.1%-59.3%)	Off track, lower certainty
Guinea-Bissau*	23.7% (5.4%-54.2%)	21.4% (6.2%-44.9%)	19.8% (2.7%-51.2%)	19.7% (1.0%-59.9%)	Off track, lower certainty
Guyana^	33.2% (10.4%-62.4%)	38.0% (23.4%-54.3%)	45.1% (26.1%-65.4%)	49.8% (20.1%-79.4%)	Off track, higher certainty
Haiti*	26.5% (5.9%-59.3%)	30.2% (9.8%-58.2%)	35.8% (8.1%-70.9%)	39.8% (5.3%-83.4%)	Off track, lower certainty
Honduras*	31.6% (8.2%-65.4%)	36.5% (13.7%-64.3%)	42.9% (11.6%-78.3%)	47.1% (8.2%-88.6%)	Off track, lower certainty
Hungary	20.7% (12.1%-31.5%)	25.0% (18.7%-32.0%)	30.6% (20.2%-42.3%)	34.8% (18.7%-53.5%)	Off track, higher certainty
Iceland*	35.9% (10.0%-70.0%)	32.0% (11.4%-59.7%)	28.0% (5.2%-63.5%)	26.5% (2.0%-70.1%)	On track, lower certainty
India	25.0% (14.3%-38.6%)	38.7% (30.3%-46.9%)	57.2% (40.1%-73.7%)	68.3% (43.8%-88.2%)	Off track, higher certainty
Indonesia	16.2% (6.4%-30.8%)	15.7% (9.7%-23.0%)	16.4% (7.7%-28.3%)	17.5% (5.0%-37.8%)	Off track, lower certainty
Iran (Islamic Republic of)	36.2% (23.6%-50.2%)	45.4% (38.9%-52.2%)	56.6% (43.8%-68.8%)	63.6% (43.2%-81.0%)	Off track, higher certainty
Iraq^	42.8% (20.9%-67.1%)	51.4% (38.5%-64.2%)	61.5% (41.1%-80.8%)	67.1% (36.0%-91.6%)	Off track, higher certainty
Ireland	45.1% (32.6%-57.8%)	35.2% (28.6%-42.1%)	24.1% (16.2%-32.7%)	18.1% (8.7%-30.4%)	On track, higher certainty
Israel*	38.7% (11.6%-71.3%)	34.0% (12.7%-61.4%)	29.0% (5.8%-63.9%)	26.8% (2.2%-70.4%)	On track, lower certainty
Italy	50.0% (36.5%-63.3%)	47.0% (38.6%-55.5%)	43.8% (31.5%-56.7%)	41.7% (24.5%-60.9%)	Off track, lower certainty
Jamaica^	32.4% (15.7%-53.1%)	38.4% (24.7%-53.1%)	45.7% (16.7%-76.3%)	50.3% (10.7%-89.0%)	Off track, higher certainty
Japan^	33.2% (8.5%-66.0%)	39.7% (21.5%-59.4%)	49.5% (33.0%-65.9%)	56.0% (29.9%-79.7%)	Off track, higher certainty
Jordan	25.6% (15.2%-37.9%)	25.9% (19.1%-33.3%)	26.1% (14.1%-40.7%)	26.6% (9.7%-49.0%)	Off track, lower certainty
Kazakhstan^	16.7% (7.8%-28.7%)	21.0% (10.1%-35.5%)	27.7% (6.0%-60.4%)	32.7% (3.6%-77.6%)	Off track, lower certainty

	Prevalence in 2000 (95% UI)	Prevalence in 2010 (95% UI)	Prevalence in 2022 (95% UI)	Prevalence in 2030 if trends 2010-2022 continue (95% UI)	Progress towards global target during 2010-2022
Kenya^	15.3% (7.2%-26.8%)	12.0% (7.2%-18.0%)	9.3% (3.0%-19.5%)	8.3% (1.2%-23.4%)	On track, lower certainty
Kiribati^	46.5% (23.7%-70.3%)	44.1% (30.9%-57.6%)	41.1% (22.7%-61.1%)	39.6% (14.3%-70.0%)	Off track, lower certainty
Kuwait^	59.4% (37.4%-80.6%)	62.9% (48.7%-75.7%)	65.1% (35.9%-88.4%)	65.9% (24.9%-95.0%)	Off track, lower certainty
Kyrgyzstan^	19.1% (5.2%-40.2%)	19.9% (10.9%-31.3%)	22.0% (8.0%-41.8%)	24.4% (4.5%-57.0%)	Off track, lower certainty
Lao People's Democratic Republic^	20.8% (11.0%-33.6%)	20.8% (13.8%-29.2%)	22.4% (8.7%-41.8%)	23.9% (5.4%-53.8%)	Off track, lower certainty
Latvia	24.2% (12.2%-39.4%)	18.9% (12.8%-26.3%)	13.7% (7.4%-22.1%)	11.2% (3.6%-23.7%)	On track, higher certainty
Lebanon^	31.5% (14.5%-52.5%)	43.8% (32.8%-55.2%)	59.4% (40.6%-77.1%)	68.4% (39.7%-90.5%)	Off track, higher certainty
Lesotho^	14.0% (3.3%-31.3%)	9.8% (4.6%-17.1%)	7.4% (1.4%-19.4%)	7.0% (0.3%-26.0%)	On track, lower certainty
Liberia^	36.8% (16.9%-60.9%)	26.0% (16.3%-37.7%)	15.5% (8.5%-25.1%)	10.9% (3.2%-24.2%)	On track, higher certainty
Libya^	38.6% (19.9%-60.2%)	45.4% (33.3%-58.1%)	53.6% (39.9%-66.8%)	58.8% (37.6%-77.8%)	Off track, higher certainty
Lithuania	25.2% (15.4%-36.6%)	22.5% (17.2%-28.3%)	19.8% (12.2%-29.1%)	18.4% (8.0%-32.6%)	On track, lower certainty
Luxembourg	30.1% (19.4%-42.0%)	21.9% (16.6%-28.3%)	13.9% (8.0%-21.3%)	10.1% (3.8%-20.2%)	On track, higher certainty
Madagascar*	18.0% (3.1%-46.0%)	15.6% (3.8%-37.1%)	14.6% (1.6%-42.2%)	14.9% (0.6%-51.0%)	Off track, lower certainty
Malawi	19.6% (10.1%-31.8%)	8.9% (5.6%-13.0%)	2.9% (0.8%-7.1%)	1.4% (0.1%-5.4%)	On track, higher certainty
Malaysia	47.0% (35.9%-58.5%)	45.0% (38.3%-52.0%)	42.4% (28.8%-56.3%)	40.8% (21.7%-61.6%)	Off track, lower certainty
Maldives^	30.3% (10.3%-57.8%)	26.6% (14.6%-41.4%)	23.5% (12.8%-36.4%)	22.2% (7.5%-42.8%)	On track, lower certainty
Mali^	41.3% (17.3%-67.1%)	38.0% (18.9%-59.4%)	33.8% (8.4%-67.7%)	31.9% (3.4%-76.5%)	On track, lower certainty
Malta	59.7% (41.3%-76.4%)	52.9% (42.3%-63.5%)	44.1% (28.4%-61.2%)	38.6% (17.6%-64.9%)	On track, lower certainty
Marshall Islands^	44.9% (23.1%-68.2%)	38.1% (18.7%-60.5%)	32.0% (6.5%-67.2%)	29.2% (2.2%-74.3%)	On track, lower certainty
Mauritania^	70.2% (53.7%-83.9%)	59.7% (40.7%-77.2%)	45.8% (12.9%-81.3%)	38.3% (3.8%-85.2%)	On track, lower certainty
Mauritius^	27.3% (14.9%-42.1%)	30.5% (21.8%-40.7%)	34.7% (21.4%-49.1%)	37.7% (18.6%-58.8%)	Off track, higher certainty
Mexico	22.9% (14.6%-33.2%)	26.1% (20.7%-32.1%)	30.5% (21.4%-41.0%)	33.8% (19.8%-49.9%)	Off track, higher certainty
Micronesia (Federated States of)	49.8% (29.1%-70.0%)	42.8% (28.3%-57.8%)	35.6% (14.0%-61.3%)	31.9% (6.4%-67.4%)	On track, lower certainty
Monaco*	36.5% (10.6%-70.3%)	31.9% (11.3%-58.8%)	27.8% (4.9%-62.1%)	26.1% (1.9%-70.0%)	On track, lower certainty
Mongolia	7.6% (3.1%-14.6%)	14.8% (10.5%-19.9%)	28.8% (16.8%-43.4%)	40.7% (19.2%-65.4%)	Off track, higher certainty
Montenegro*	22.8% (4.5%-54.3%)	22.6% (6.3%-48.7%)	23.9% (3.8%-58.2%)	25.5% (1.9%-69.7%)	Off track, lower certainty
Morocco^	27.7% (9.6%-52.3%)	27.7% (16.9%-40.6%)	29.1% (14.8%-45.7%)	30.6% (9.7%-57.6%)	Off track, lower certainty
Mozambique^	8.4% (2.9%-17.2%)	8.8% (3.7%-16.6%)	11.0% (1.3%-31.1%)	13.2% (0.5%-46.3%)	Off track, lower certainty
Myanmar	24.1% (14.1%-36.6%)	23.5% (16.6%-31.6%)	23.6% (9.8%-41.9%)	24.2% (5.7%-51.9%)	Off track, lower certainty
Namibia^	43.3% (27.4%-60.2%)	36.4% (21.3%-54.2%)	29.1% (6.5%-63.6%)	25.8% (2.0%-71.5%)	On track, lower certainty
Nauru^	47.0% (29.6%-64.4%)	44.6% (34.1%-55.4%)	42.8% (22.7%-64.3%)	41.9% (14.5%-73.5%)	Off track, lower certainty

	Prevalence in 2000 (95% UI)	Prevalence in 2010 (95% UI)	Prevalence in 2022 (95% UI)	Prevalence in 2030 if trends 2010-2022 continue (95% UI)	Progress towards global target during 2010-2022
Nepal	13.6% (6.5%-23.3%)	9.5% (6.2%-13.7%)	6.3% (2.7%-12.1%)	5.1% (1.1%-12.9%)	On track, higher certainty
Netherlands	20.2% (12.7%-29.1%)	15.5% (11.6%-20.1%)	10.7% (6.7%-15.7%)	8.3% (3.8%-14.9%)	On track, higher certainty
New Zealand	25.1% (15.5%-36.6%)	22.7% (16.9%-29.3%)	19.5% (13.0%-27.5%)	17.8% (8.9%-29.2%)	On track, lower certainty
Nicaragua*	34.4% (9.6%-70.9%)	38.7% (15.5%-67.5%)	44.3% (12.1%-79.5%)	47.8% (8.6%-89.4%)	Off track, lower certainty
Niger^	24.9% (8.8%-47.4%)	22.3% (12.3%-34.9%)	19.3% (10.4%-31.6%)	18.1% (6.0%-36.6%)	On track, lower certainty
Nigeria*	24.4% (5.7%-55.1%)	21.9% (6.9%-45.2%)	20.6% (2.9%-53.7%)	20.6% (1.2%-63.9%)	Off track, lower certainty
Niue^	18.5% (4.2%-41.1%)	11.9% (5.3%-21.5%)	8.1% (1.4%-22.7%)	7.2% (0.3%-29.9%)	On track, lower certainty
Norway	34.0% (19.5%-50.3%)	36.0% (23.8%-49.2%)	38.5% (15.8%-65.3%)	40.2% (10.1%-76.7%)	Off track, lower certainty
occupied Palestinian territory, including east Jerusalem^	50.3% (28.4%-73.8%)	42.6% (30.2%-55.4%)	33.1% (21.1%-46.8%)	27.8% (11.4%-49.0%)	On track, lower certainty
Oman^	35.9% (18.2%-56.1%)	42.0% (31.7%-53.0%)	50.4% (32.5%-69.4%)	55.8% (27.8%-82.2%)	Off track, higher certainty
Pakistan^	34.9% (20.5%-50.7%)	44.5% (33.6%-55.5%)	57.0% (35.2%-77.9%)	64.4% (33.7%-89.8%)	Off track, higher certainty
Palau^	40.5% (16.4%-66.9%)	37.9% (25.5%-51.4%)	36.0% (16.1%-58.8%)	35.4% (8.4%-71.0%)	Off track, lower certainty
Panama^	40.4% (13.2%-72.4%)	52.2% (33.6%-70.4%)	66.9% (50.4%-81.0%)	74.7% (50.8%-92.1%)	Off track, higher certainty
Papua New Guinea^	21.3% (8.4%-38.3%)	18.1% (9.3%-29.5%)	17.0% (2.9%-44.0%)	17.3% (0.9%-57.3%)	Off track, lower certainty
Paraguay^	24.7% (13.3%-39.5%)	30.8% (16.9%-48.0%)	39.8% (11.4%-73.5%)	45.5% (7.7%-87.5%)	Off track, higher certainty
Peru^	29.5% (8.1%-59.4%)	31.7% (16.7%-48.9%)	36.9% (19.7%-55.8%)	40.8% (14.5%-69.3%)	Off track, higher certainty
Philippines	15.5% (7.3%-27.0%)	30.4% (23.2%-38.4%)	55.0% (36.2%-73.2%)	69.9% (42.5%-90.6%)	Off track, higher certainty
Poland	25.2% (12.9%-40.2%)	30.4% (22.4%-39.2%)	38.0% (25.9%-50.5%)	43.3% (24.4%-63.9%)	Off track, higher certainty
Portugal	45.5% (32.2%-58.8%)	50.6% (42.1%-59.1%)	56.9% (43.9%-69.8%)	60.9% (41.7%-78.6%)	Off track, higher certainty
Puerto Rico*	36.1% (10.4%-69.5%)	40.5% (17.0%-68.4%)	46.0% (13.1%-80.8%)	49.6% (9.5%-90.1%)	Off track, lower certainty
Qatar^	53.5% (31.3%-75.9%)	58.5% (43.8%-72.4%)	63.2% (33.7%-87.7%)	65.4% (23.4%-94.8%)	Off track, higher certainty
Republic of Korea	32.1% (20.3%-45.4%)	44.2% (38.2%-50.4%)	60.3% (52.0%-68.1%)	69.9% (55.7%-82.0%)	Off track, higher certainty
Republic of Moldova^	17.5% (4.9%-37.7%)	13.4% (6.8%-22.0%)	9.6% (4.7%-16.8%)	8.2% (2.0%-19.4%)	On track, lower certainty
Republic of North Macedonia^	27.8% (7.2%-57.2%)	28.9% (13.1%-48.8%)	31.6% (11.1%-59.1%)	34.1% (6.7%-71.9%)	Off track, lower certainty
Romania	21.3% (6.6%-42.8%)	27.1% (16.5%-39.4%)	36.6% (24.7%-49.7%)	43.7% (23.1%-66.3%)	Off track, higher certainty
Russian Federation^	11.1% (4.9%-19.5%)	13.6% (7.2%-21.8%)	18.2% (3.9%-42.6%)	22.1% (2.3%-61.5%)	Off track, lower certainty
Rwanda^	24.4% (9.5%-46.5%)	16.4% (9.2%-25.6%)	10.1% (5.2%-17.1%)	7.5% (2.0%-17.4%)	On track, higher certainty
Saint Kitts and Nevis^	37.7% (15.9%-63.3%)	42.8% (22.9%-63.3%)	48.7% (17.9%-80.3%)	52.2% (11.6%-90.4%)	Off track, lower certainty
Saint Lucia^	30.9% (9.5%-60.1%)	27.6% (14.8%-43.0%)	24.0% (12.8%-37.5%)	22.5% (6.8%-44.3%)	On track, lower certainty
Saint Vincent and the Grenadines^	31.4% (11.1%-59.0%)	34.8% (21.7%-50.0%)	39.9% (19.9%-63.2%)	43.6% (12.8%-78.0%)	Off track, lower certainty
Samoa^	49.0% (33.2%-64.6%)	32.6% (23.0%-43.3%)	17.6% (5.5%-35.4%)	11.5% (1.3%-32.9%)	On track, higher certainty

	Prevalence in 2000 (95% UI)	Prevalence in 2010 (95% UI)	Prevalence in 2022 (95% UI)	Prevalence in 2030 if trends 2010-2022 continue (95% UI)	Progress towards global target during 2010-2022
San Marino*	36.7% (11.4%-68.9%)	32.3% (11.4%-57.6%)	28.3% (5.5%-61.5%)	26.7% (2.2%-69.6%)	On track, lower certainty
Saudi Arabia	68.8% (53.7%-82.3%)	64.4% (56.4%-71.6%)	57.8% (41.9%-72.2%)	53.4% (28.4%-76.3%)	On track, lower certainty
Senegal^	26.6% (8.1%-52.5%)	24.0% (13.4%-37.5%)	21.6% (8.9%-39.0%)	21.0% (4.3%-49.4%)	Off track, lower certainty
Serbia^	56.5% (36.3%-76.2%)	52.5% (36.2%-68.9%)	47.9% (16.6%-80.1%)	45.5% (6.8%-87.4%)	Off track, lower certainty
Seychelles^	21.3% (10.5%-35.1%)	20.9% (13.6%-29.2%)	21.2% (7.6%-40.9%)	22.0% (4.0%-52.9%)	Off track, lower certainty
Sierra Leone^	22.1% (8.5%-41.7%)	19.3% (10.5%-30.1%)	17.1% (3.9%-39.5%)	16.7% (1.4%-50.1%)	Off track, lower certainty
Singapore	25.3% (12.7%-40.3%)	23.1% (17.0%-29.9%)	22.0% (13.7%-31.8%)	21.7% (8.9%-38.4%)	Off track, lower certainty
Slovakia	28.6% (10.8%-53.4%)	26.0% (16.0%-37.9%)	24.1% (14.6%-35.1%)	23.4% (8.9%-42.6%)	Off track, lower certainty
Slovenia	22.1% (11.3%-37.5%)	20.8% (14.5%-28.7%)	19.5% (11.5%-29.7%)	19.1% (7.9%-35.1%)	Off track, lower certainty
Solomon Islands^	39.8% (21.7%-59.6%)	30.7% (21.4%-40.5%)	22.3% (8.8%-39.8%)	18.4% (3.0%-44.2%)	On track, lower certainty
Somalia*	25.7% (6.1%-56.3%)	23.5% (7.5%-47.7%)	22.4% (3.6%-54.5%)	22.6% (1.6%-65.8%)	Off track, lower certainty
South Africa	52.9% (40.5%-64.9%)	51.1% (42.6%-59.9%)	48.1% (29.0%-68.2%)	46.2% (19.6%-74.6%)	Off track, lower certainty
South Sudan*	22.0% (4.3%-50.9%)	19.8% (5.5%-42.6%)	18.9% (2.6%-49.9%)	19.3% (1.0%-60.7%)	Off track, lower certainty
Spain	44.8% (32.4%-57.8%)	35.6% (29.2%-42.5%)	24.2% (16.2%-33.6%)	18.2% (8.3%-31.1%)	On track, higher certainty
Sri Lanka	21.7% (12.3%-33.4%)	31.0% (23.9%-39.0%)	44.8% (31.4%-58.3%)	54.3% (33.7%-74.0%)	Off track, higher certainty
Sudan^	26.4% (7.6%-53.8%)	23.4% (12.6%-36.7%)	20.9% (9.3%-37.0%)	20.2% (4.7%-46.1%)	Off track, lower certainty
Suriname^	40.4% (16.3%-68.0%)	47.1% (32.4%-61.9%)	55.1% (30.9%-78.2%)	59.7% (23.3%-90.0%)	Off track, higher certainty
Sweden	33.7% (22.2%-46.8%)	19.5% (13.9%-25.9%)	8.1% (4.0%-14.1%)	4.1% (1.1%-9.8%)	On track, higher certainty
Switzerland^	30.7% (9.8%-57.9%)	25.3% (13.7%-39.1%)	20.4% (8.3%-38.0%)	18.5% (3.5%-45.7%)	On track, lower certainty
Syrian Arab Republic*	34.6% (10.3%-67.3%)	37.9% (15.3%-64.5%)	42.4% (11.4%-77.7%)	45.4% (7.6%-87.1%)	Off track, lower certainty
São Tomé and Príncipe^	22.0% (8.6%-40.1%)	21.9% (14.1%-31.1%)	22.0% (11.4%-35.9%)	22.6% (7.3%-45.3%)	Off track, lower certainty
Taiwan, China*	23.7% (5.2%-54.2%)	24.6% (7.5%-49.6%)	27.4% (4.9%-61.1%)	29.8% (2.8%-72.1%)	Off track, lower certainty
Tajikistan^	30.7% (9.8%-59.8%)	36.6% (22.2%-52.8%)	45.0% (25.9%-64.9%)	50.7% (20.9%-80.0%)	Off track, higher certainty
Thailand	17.1% (8.2%-29.2%)	21.8% (16.7%-27.5%)	29.4% (21.6%-38.1%)	35.3% (20.3%-52.1%)	Off track, higher certainty
Timor-Leste^	21.5% (5.8%-46.4%)	25.3% (11.5%-43.5%)	33.0% (12.2%-59.9%)	38.7% (9.8%-76.5%)	Off track, higher certainty
Togo^	13.7% (4.2%-28.7%)	15.0% (8.5%-23.5%)	17.3% (9.5%-27.1%)	19.4% (7.3%-36.9%)	Off track, lower certainty
Tonga	37.7% (18.4%-59.3%)	37.3% (27.4%-47.8%)	38.1% (21.2%-56.3%)	39.0% (13.8%-67.9%)	Off track, lower certainty
Trinidad and Tobago^	42.8% (20.1%-69.1%)	48.1% (33.6%-62.6%)	54.2% (28.0%-80.0%)	57.5% (19.6%-91.2%)	Off track, higher certainty
Tunisia^	26.1% (14.2%-40.5%)	30.9% (17.1%-47.6%)	37.8% (10.8%-72.6%)	42.3% (6.8%-86.3%)	Off track, higher certainty
Turkmenistan^	19.5% (4.2%-43.6%)	16.0% (7.1%-27.5%)	13.1% (5.5%-24.4%)	12.3% (2.4%-30.5%)	On track, lower certainty
Tuvalu^	42.8% (16.8%-72.0%)	37.8% (23.1%-53.6%)	32.8% (15.1%-53.9%)	30.6% (7.2%-63.7%)	On track, lower certainty

	Prevalence in 2000 (95% UI)	Prevalence in 2010 (95% UI)	Prevalence in 2022 (95% UI)	Prevalence in 2030 if trends 2010-2022 continue (95% UI)	Progress towards global target during 2010-2022
Türkiye	47.8% (34.5%-61.7%)	50.5% (41.4%-60.1%)	53.4% (34.9%-71.3%)	55.2% (28.3%-80.4%)	Off track, higher certainty
Uganda^	14.3% (3.8%-32.2%)	8.9% (4.1%-15.7%)	5.2% (2.4%-9.3%)	3.8% (0.9%-9.5%)	On track, higher certainty
Ukraine^	10.5% (4.2%-20.1%)	11.3% (6.5%-17.4%)	12.8% (5.4%-22.8%)	14.2% (3.7%-30.3%)	Off track, lower certainty
United Arab Emirates	54.6% (39.1%-69.5%)	64.2% (55.3%-72.4%)	73.6% (58.8%-86.0%)	78.5% (57.0%-93.2%)	Off track, higher certainty
United Kingdom	41.7% (29.5%-54.5%)	31.3% (25.1%-38.2%)	19.7% (12.8%-28.0%)	14.0% (6.2%-24.8%)	On track, higher certainty
United Republic of Tanzania^	15.2% (4.4%-33.6%)	8.4% (4.0%-14.8%)	3.9% (1.7%-7.2%)	2.4% (0.5%-6.6%)	On track, higher certainty
United States of America	33.9% (22.3%-46.8%)	36.8% (31.0%-42.8%)	39.8% (28.3%-52.9%)	41.9% (23.6%-62.7%)	Off track, higher certainty
Uruguay	32.0% (18.8%-47.5%)	33.6% (22.1%-46.7%)	36.8% (13.4%-64.8%)	39.2% (8.5%-77.2%)	Off track, lower certainty
Uzbekistan	8.6% (3.6%-16.4%)	18.4% (12.7%-25.1%)	37.1% (23.8%-51.4%)	51.7% (29.4%-73.0%)	Off track, higher certainty
Vanuatu^	16.2% (4.1%-35.1%)	11.6% (5.7%-19.9%)	9.2% (1.5%-24.5%)	8.9% (0.3%-33.7%)	On track, lower certainty
Venezuela (Bolivarian Republic of)^	38.7% (13.2%-68.8%)	45.3% (29.2%-62.5%)	52.8% (32.3%-72.0%)	57.3% (26.2%-84.8%)	Off track, higher certainty
Viet Nam	17.6% (9.6%-27.9%)	23.9% (18.0%-30.5%)	34.5% (23.4%-46.6%)	42.5% (24.4%-61.9%)	Off track, higher certainty
Yemen*	24.5% (5.7%-54.9%)	28.1% (9.2%-54.4%)	34.1% (7.4%-69.7%)	38.4% (5.1%-82.6%)	Off track, lower certainty
Zambia^	20.8% (10.4%-34.2%)	19.1% (12.4%-27.2%)	17.9% (8.0%-31.9%)	17.6% (4.8%-38.4%)	Off track, lower certainty
Zimbabwe^	24.7% (13.1%-38.8%)	22.1% (10.8%-37.0%)	21.1% (3.6%-52.2%)	21.3% (1.3%-65.2%)	Off track, lower certainty

Appendix 17. Country progress during 2010-2022 toward the global target of a 15% relative reduction in insufficient physical activity prevalence among adults aged 18 years and over between 2010 and 2030, by sex

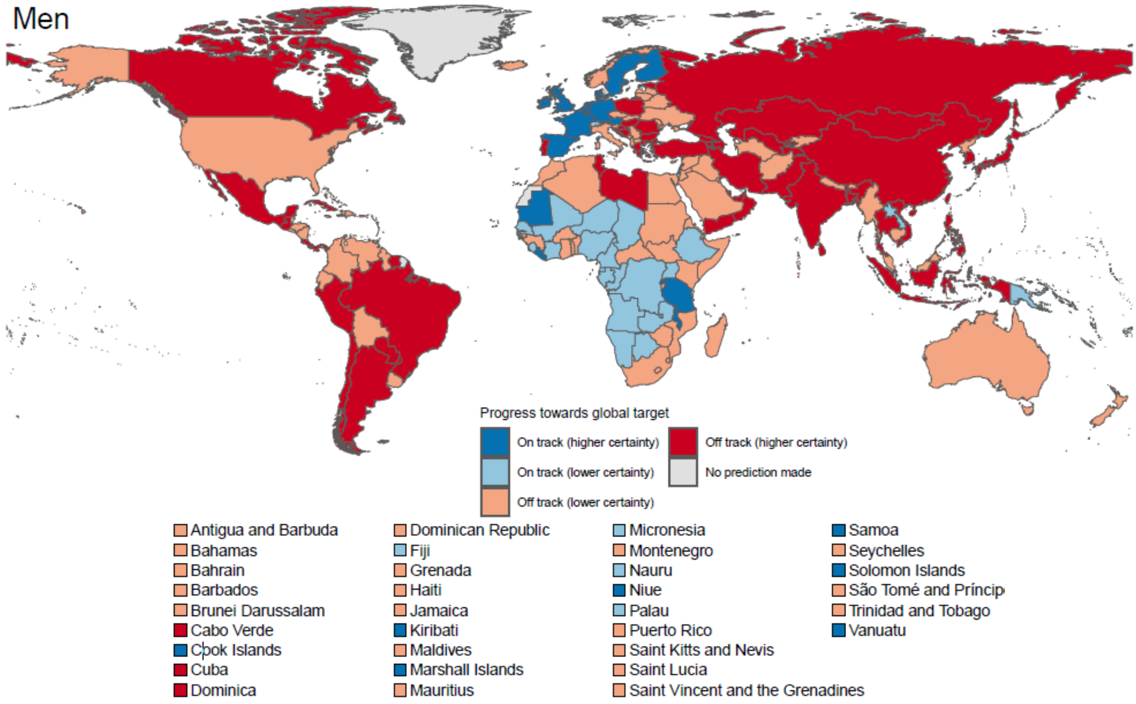

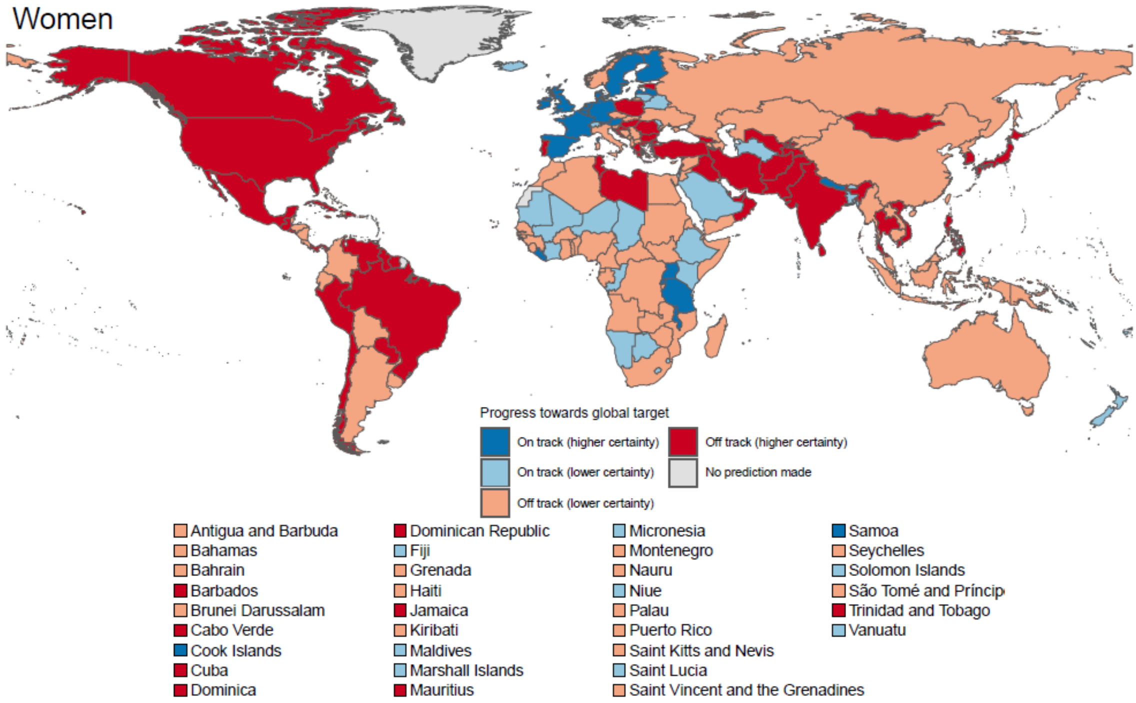

Appendix 18. Model input data and estimated levels of insufficient physical activity, by country, sex, and age.

Notes: The following plots show the model input data (colours indicate the questionnaire, and the shape of symbols denotes survey coverage). The vertical error bar indicates the 95% confidence interval for the input data. These were computed using the prevalence estimate, effective sample size and the normal approximation of the binomial proportion confidence interval. As indicated in the methods, adjustments for urban-rural coverage and definition of insufficient physical activity were made prior to fitting the Bayesian model. Adjustment for questionnaire (relative to GPAQ) was made within the Bayesian model. Both the unadjusted and adjusted values are shown on the plots. The black line and grey shaded area show the model estimated prevalence of insufficient physical activity and the 95% uncertainty interval.

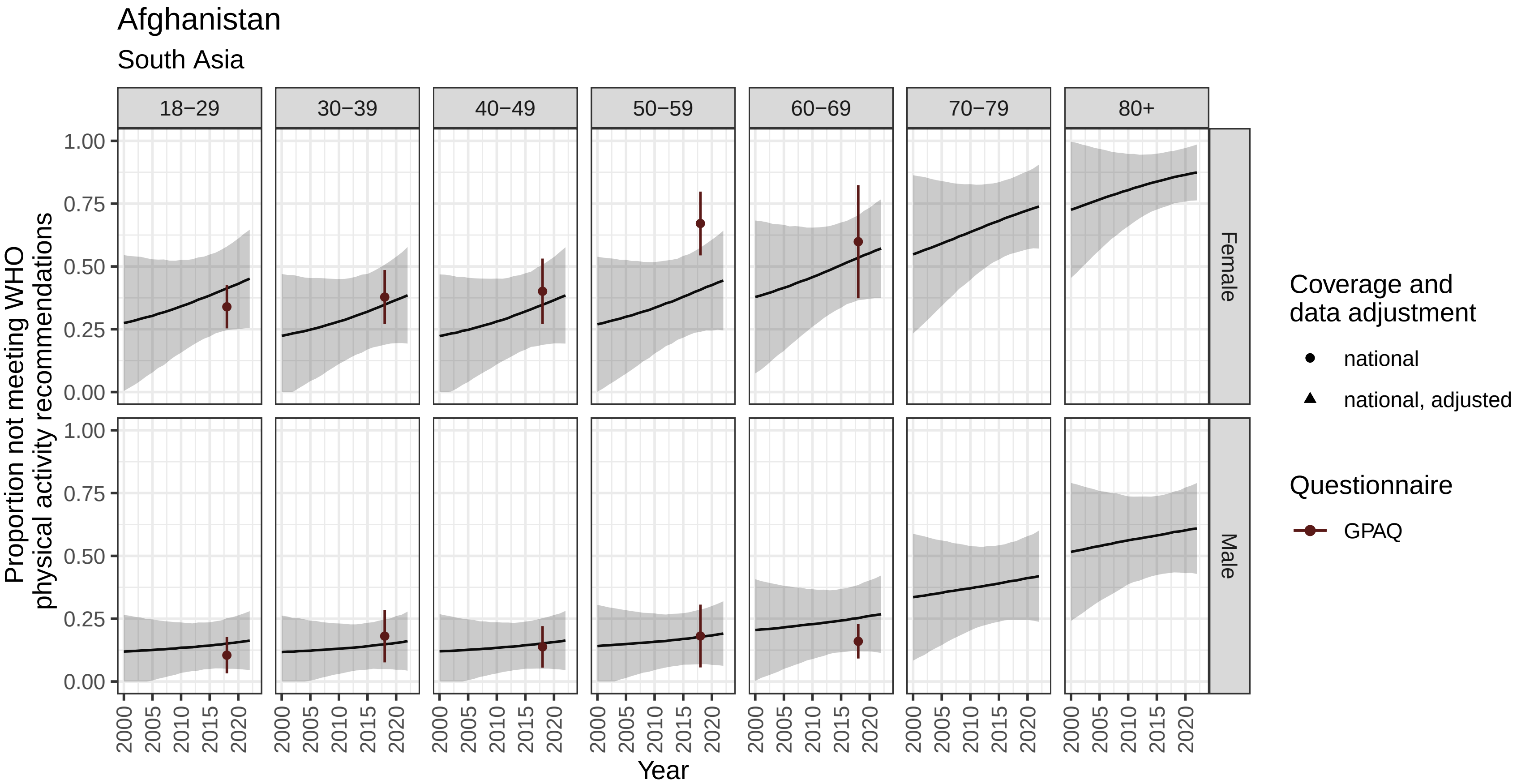

Notes: vertical lines show input data 95% confidence interval; black line shows estimate; shaded area shows 95% uncertainty interval of estimate

Albania

Central and Eastern Europe

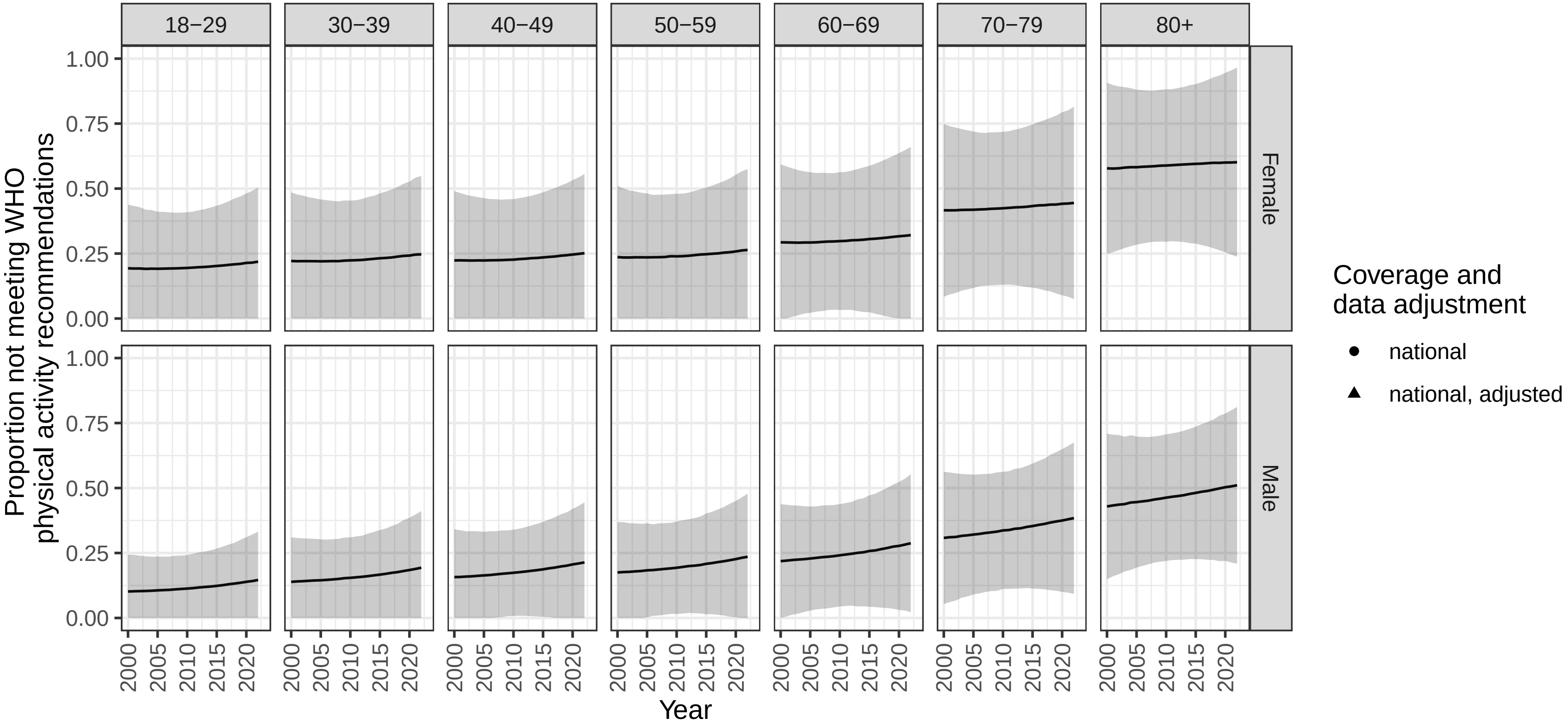

Notes: vertical lines show input data 95% confidence interval; black line shows estimate; shaded area shows 95% uncertainty interval of estimate

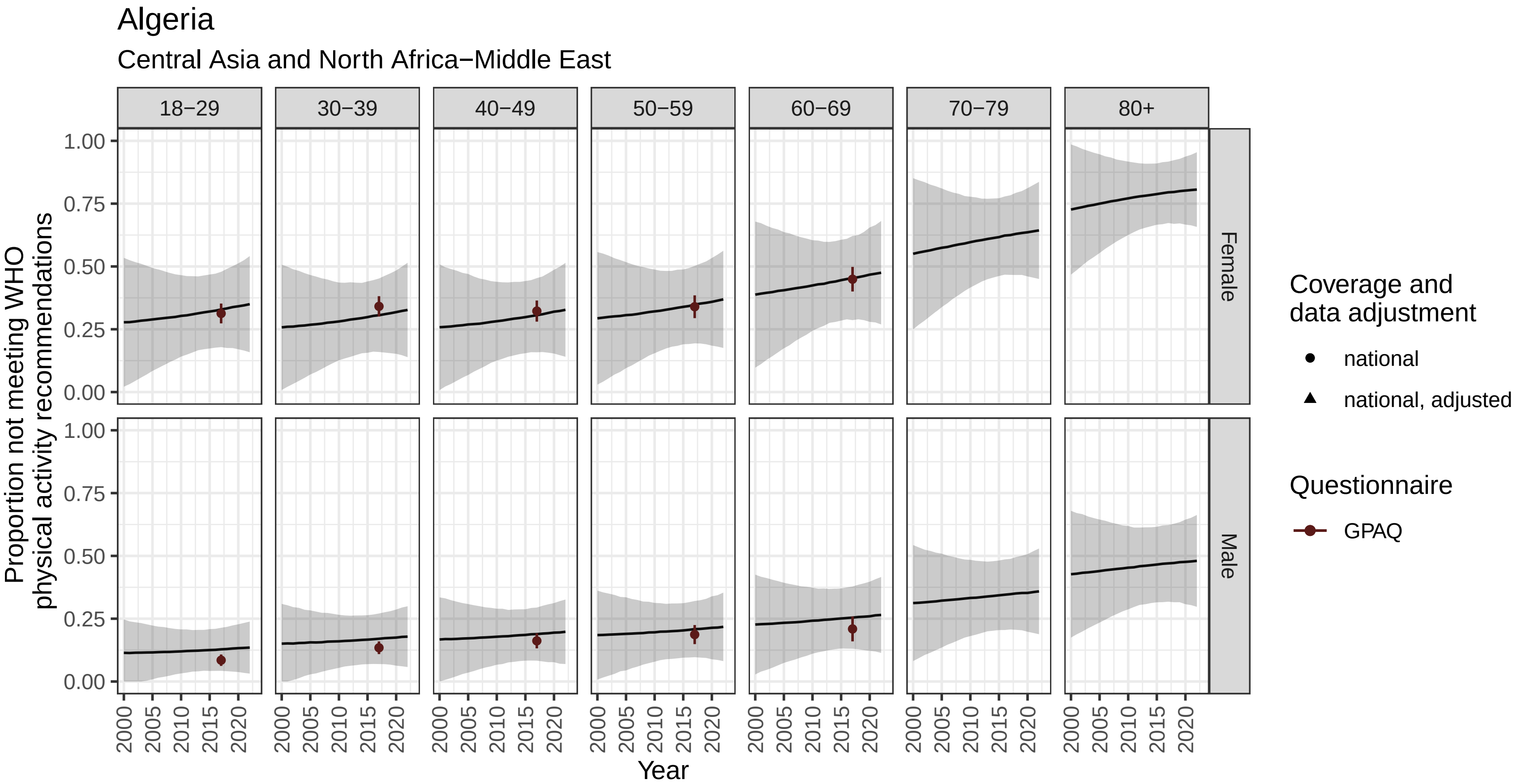

Notes: vertical lines show input data 95% confidence interval; black line shows estimate; shaded area shows 95% uncertainty interval of estimate

Andorra

High−income Western countries

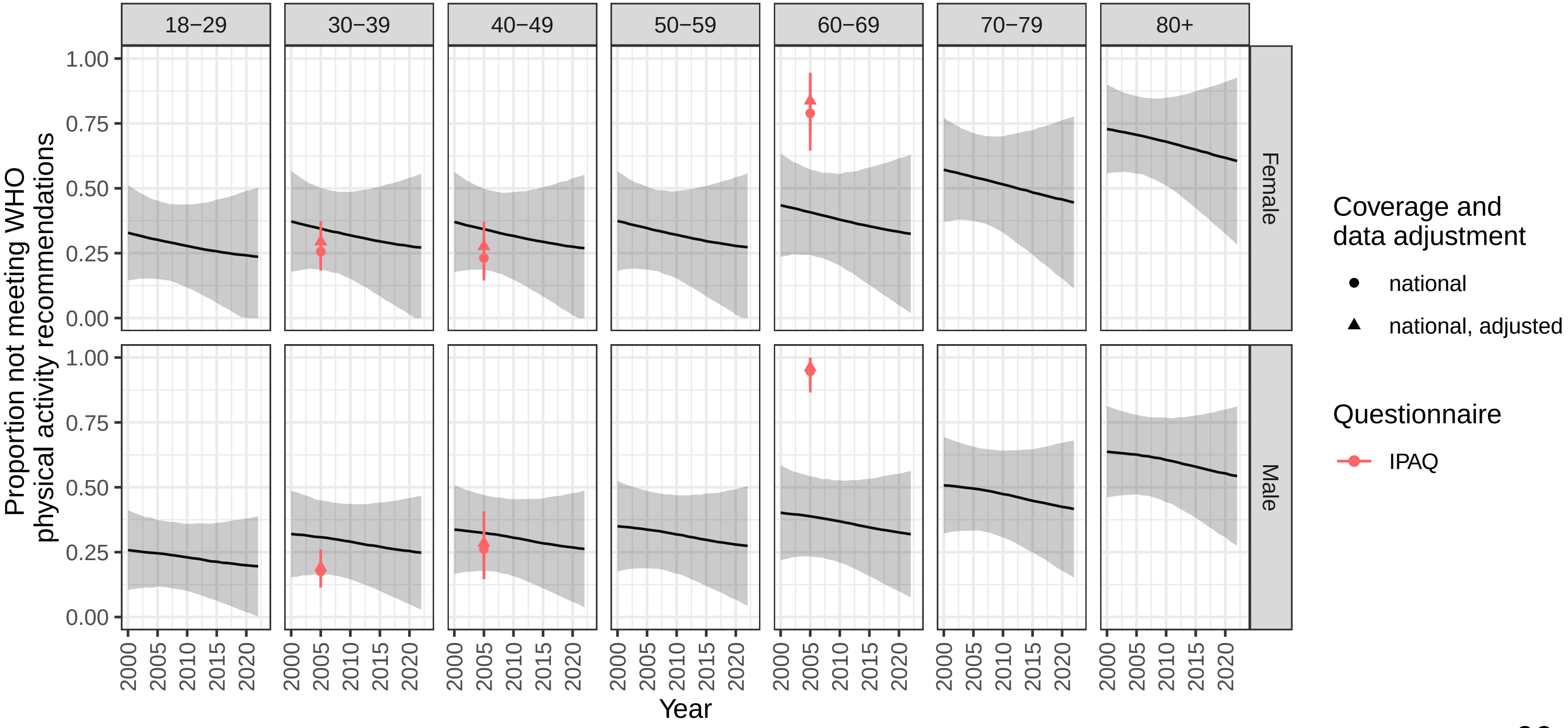

Notes: vertical lines show input data 95% confidence interval; black line shows estimate; shaded area shows 95% uncertainty interval of estimate

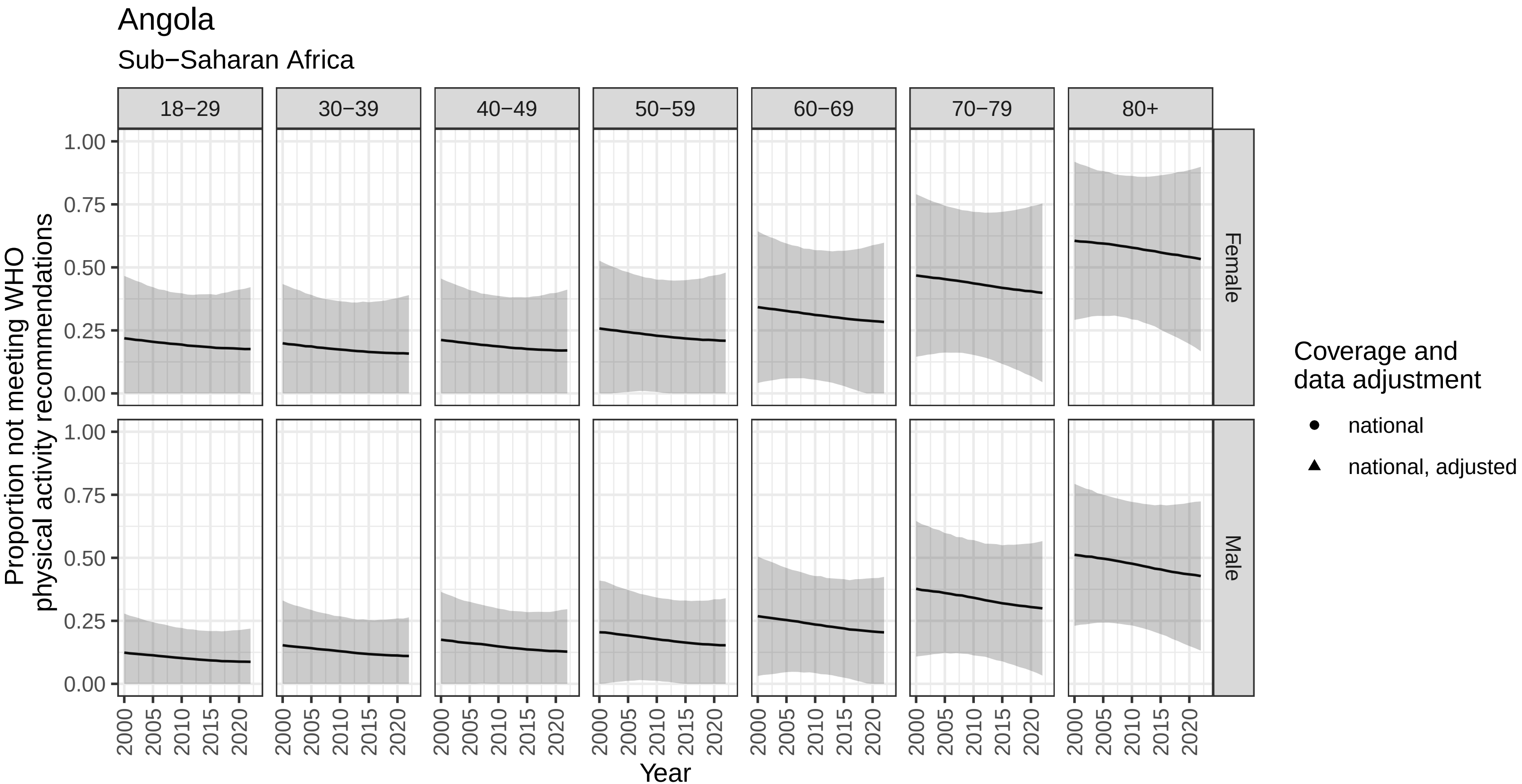

Notes: vertical lines show input data 95% confidence interval; black line shows estimate; shaded area shows 95% uncertainty interval of estimate

Antigua and Barbuda

Latin America and Caribbean

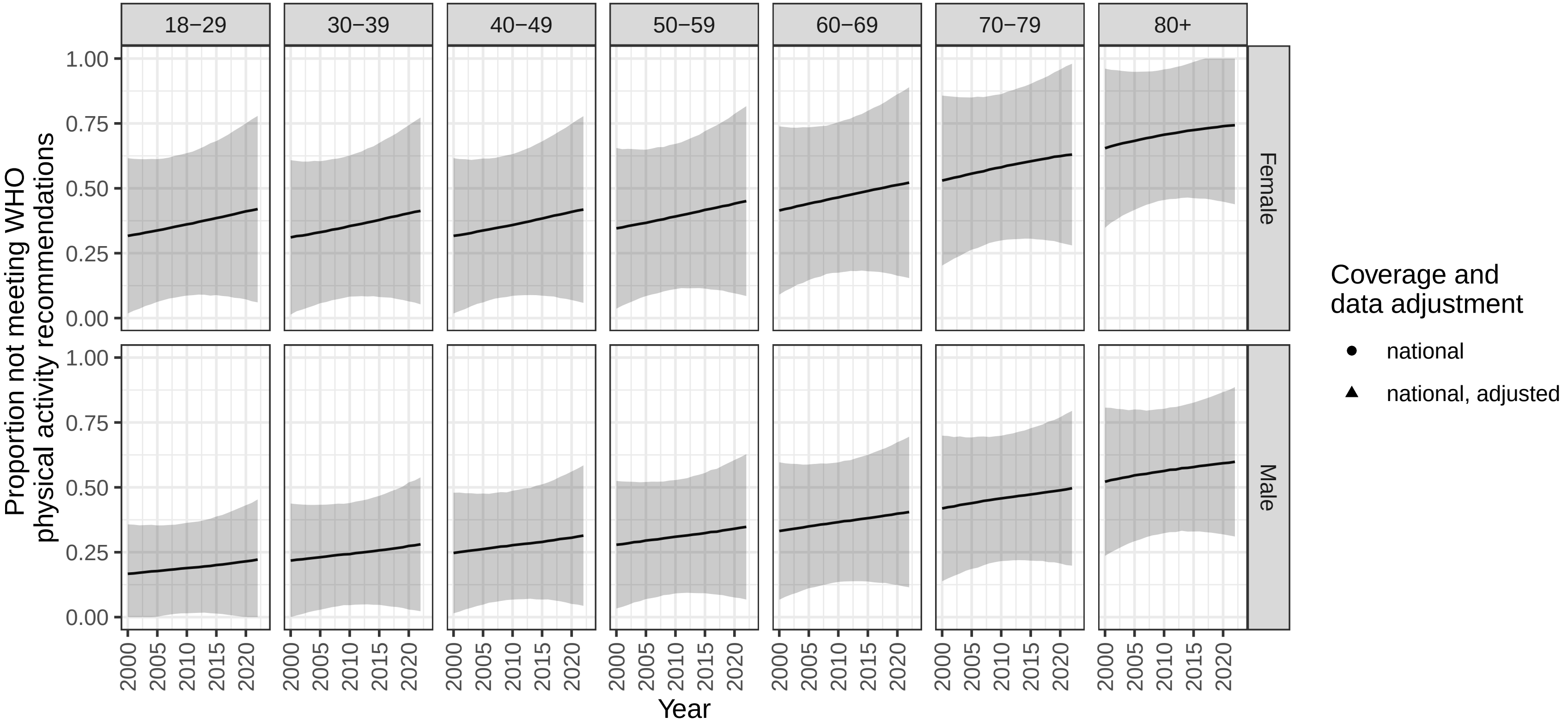

Notes: vertical lines show input data 95% confidence interval; black line shows estimate; shaded area shows 95% uncertainty interval of estimate

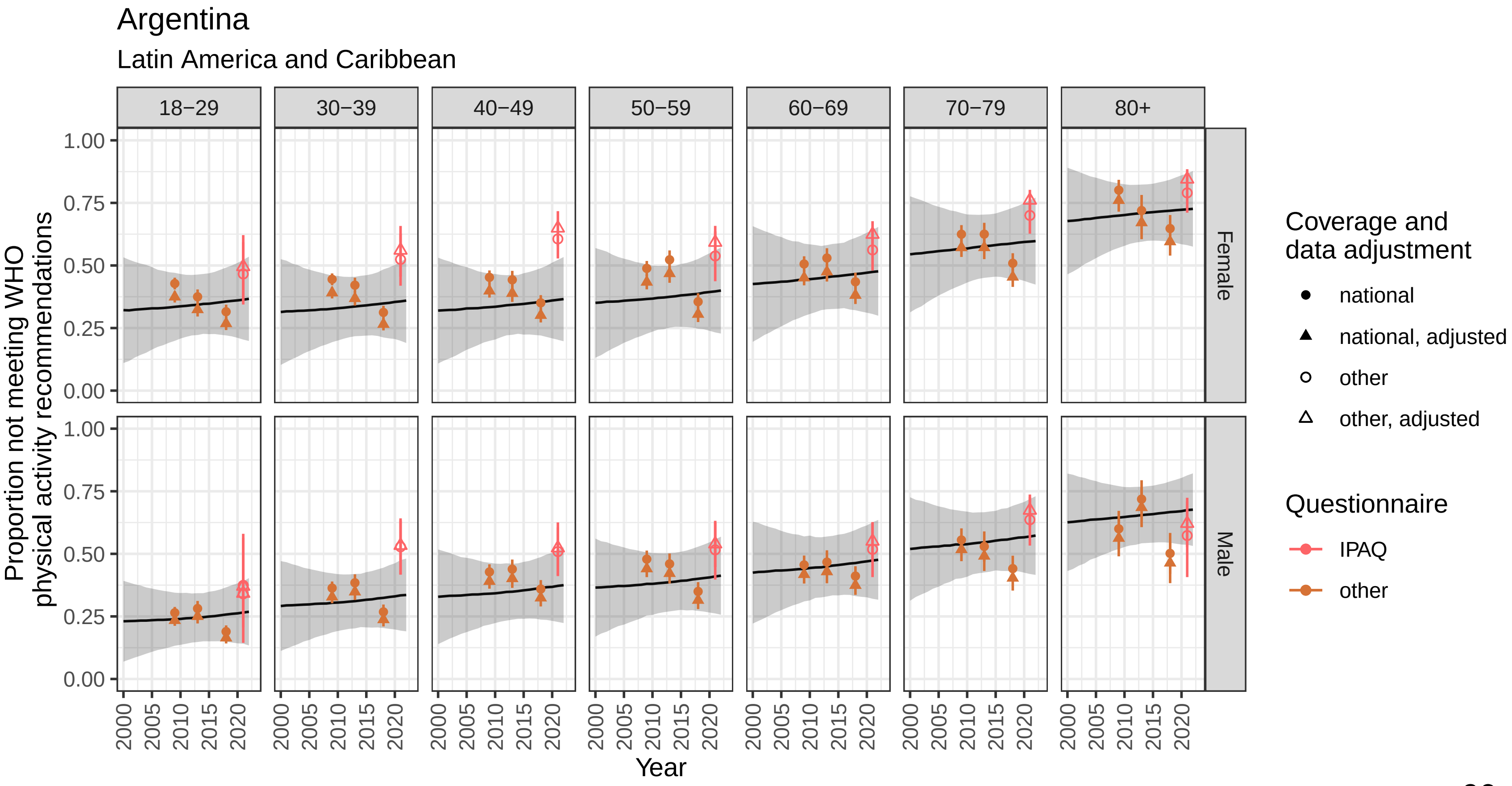

Notes: vertical lines show input data 95% confidence interval; black line shows estimate; shaded area shows 95% uncertainty interval of estimate

Armenia

Central Asia and North Africa−Middle East

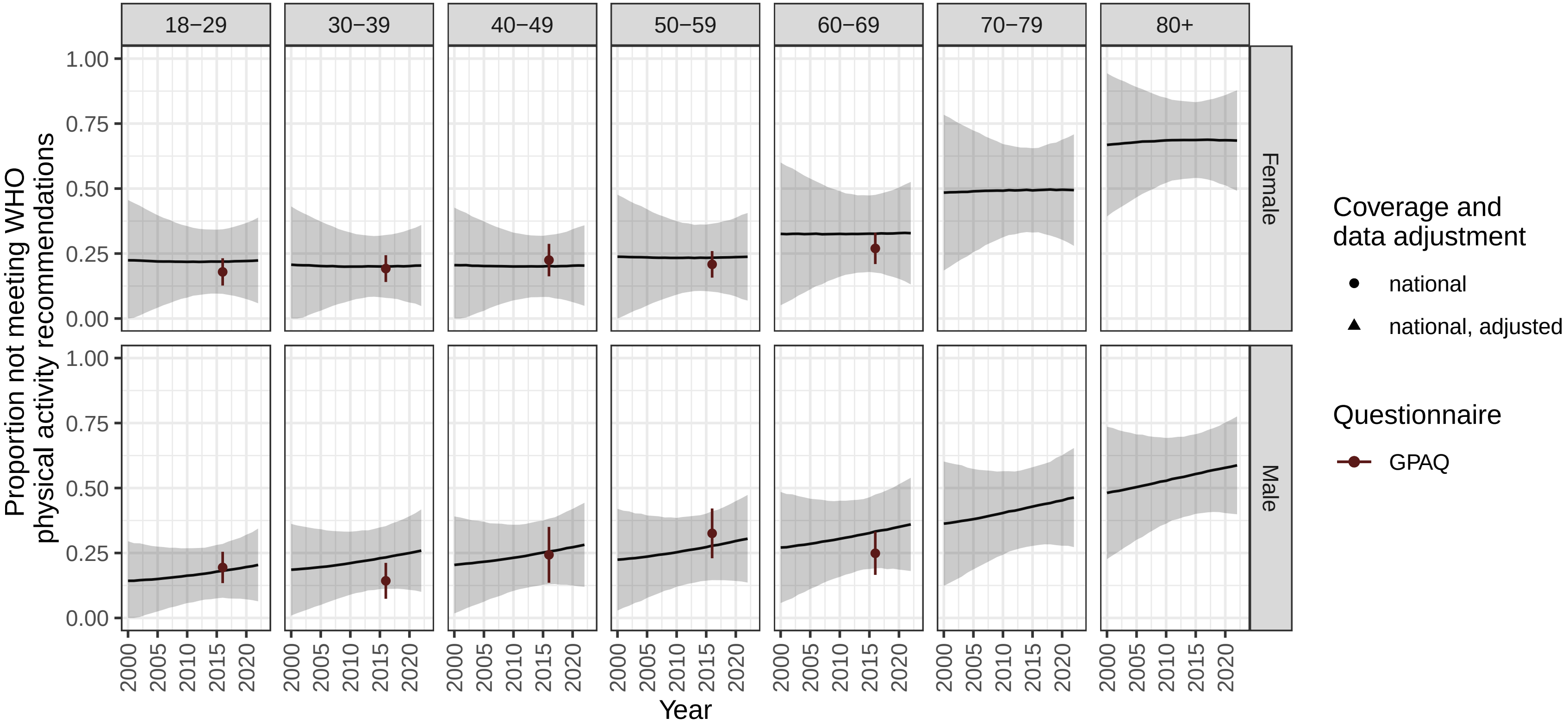

Notes: vertical lines show input data 95% confidence interval; black line shows estimate; shaded area shows 95% uncertainty interval of estimate

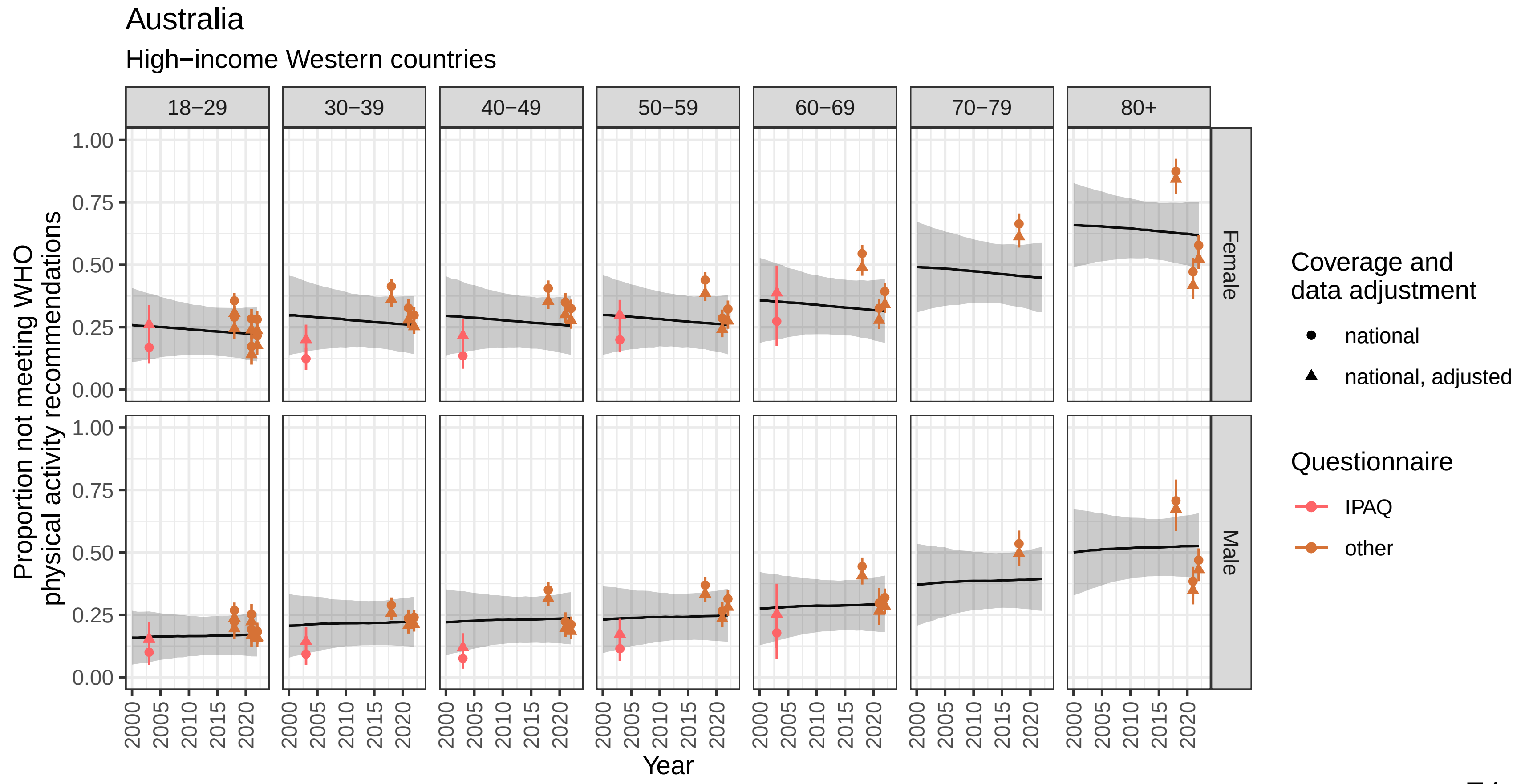

Notes: vertical lines show input data 95% confidence interval; black line shows estimate; shaded area shows 95% uncertainty interval of estimate

Austria

High−income Western countries

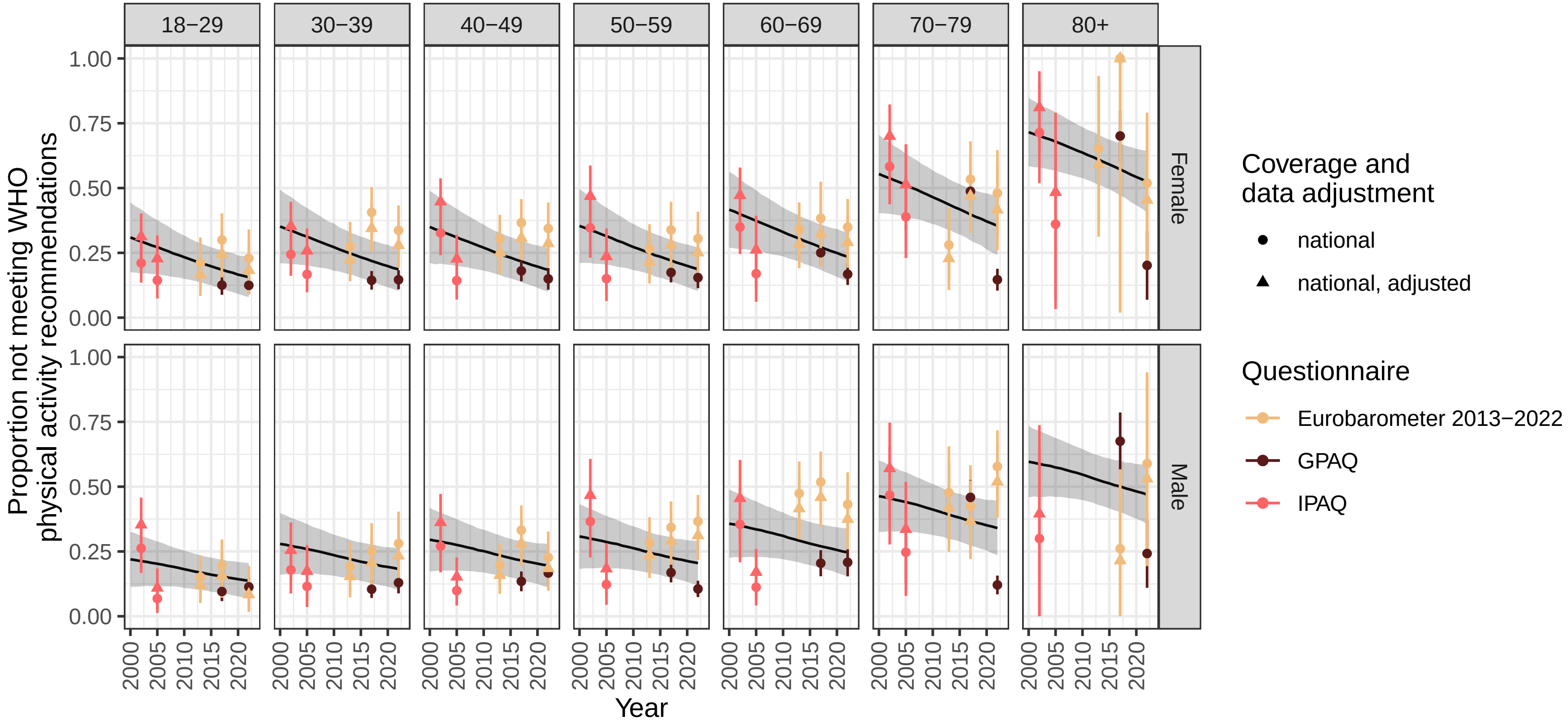

Notes: vertical lines show input data 95% confidence interval; black line shows estimate; shaded area shows 95% uncertainty interval of estimate

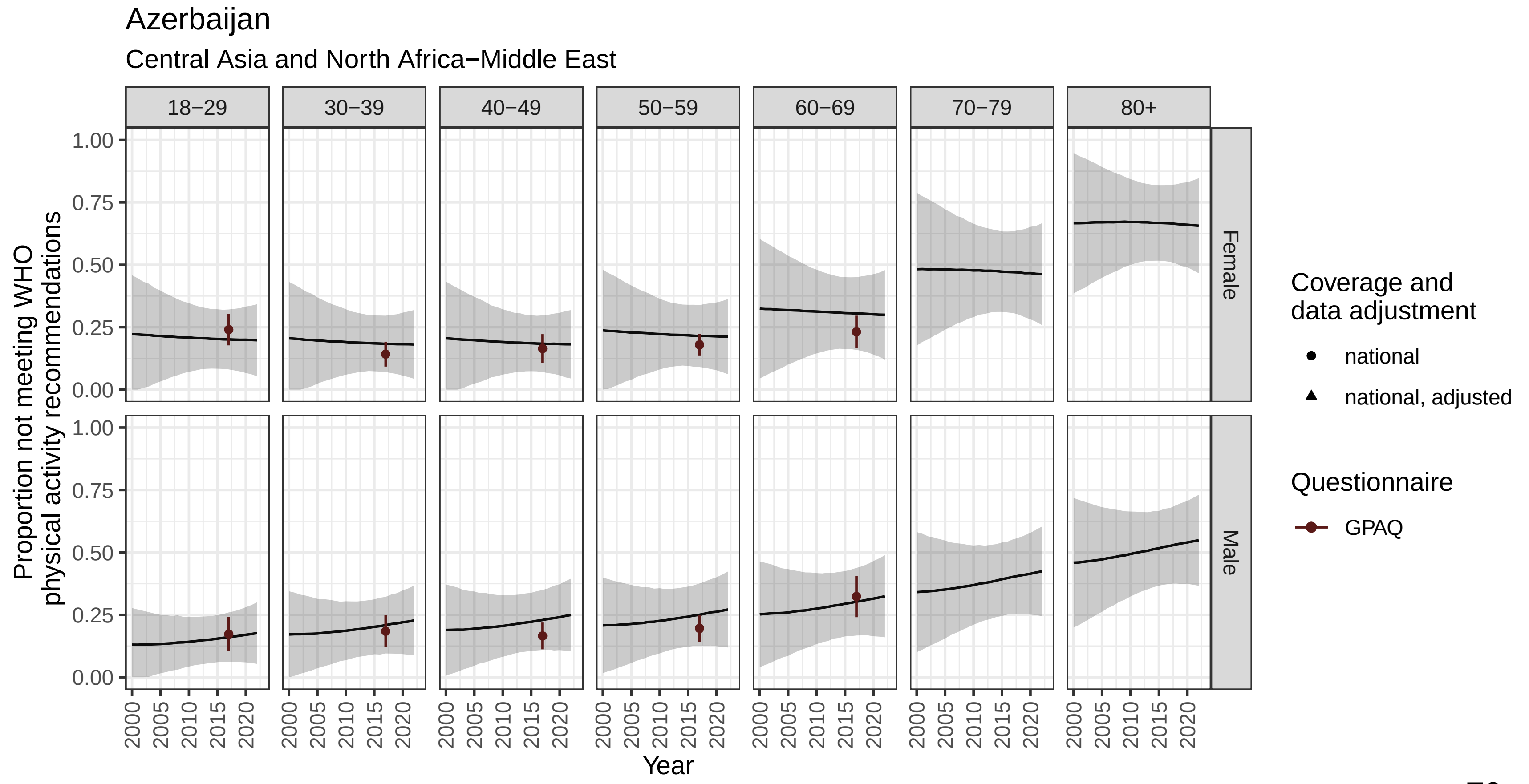

Notes: vertical lines show input data 95% confidence interval; black line shows estimate; shaded area shows 95% uncertainty interval of estimate

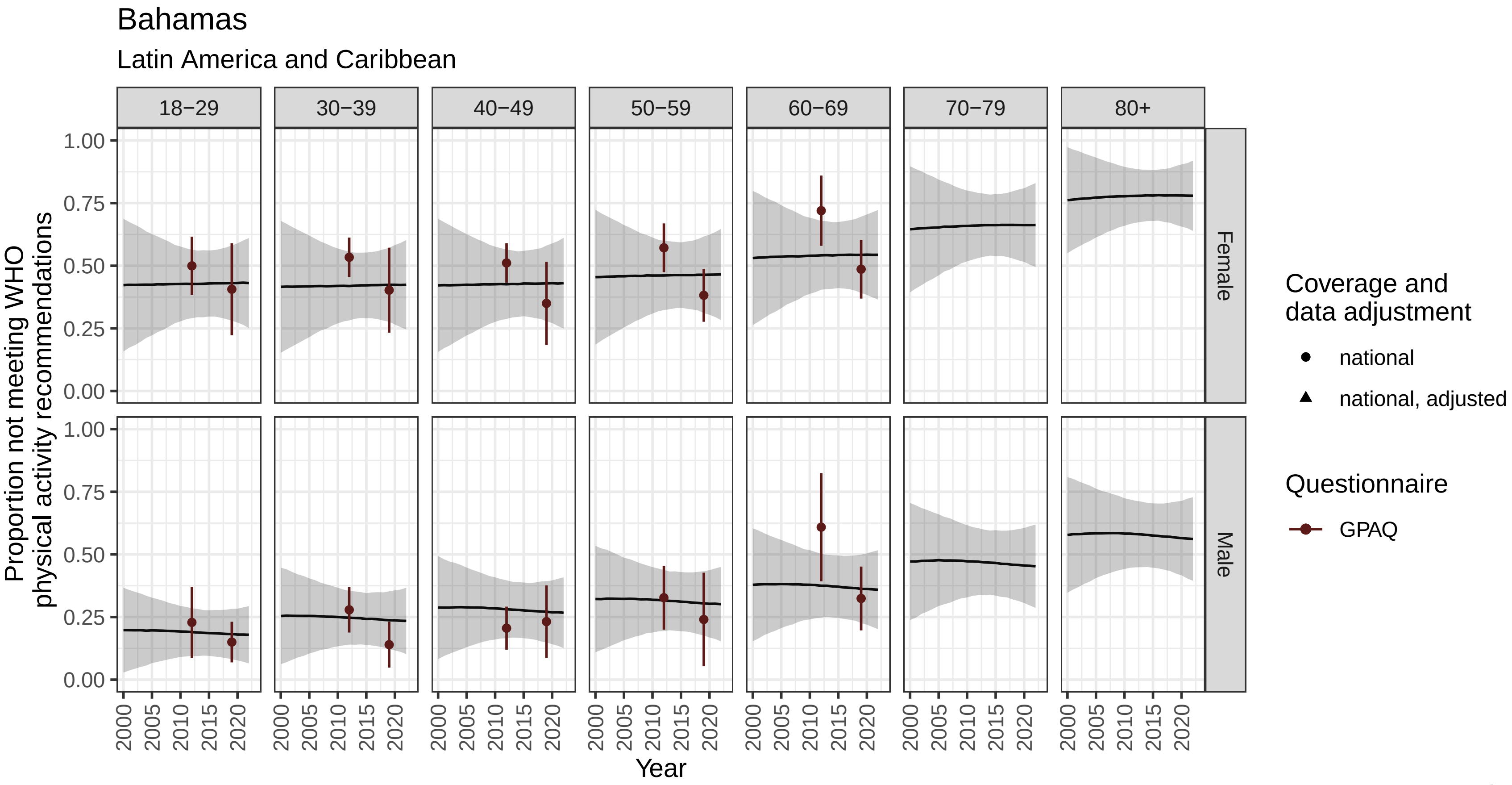

Notes: vertical lines show input data 95% confidence interval; black line shows estimate; shaded area shows 95% uncertainty interval of estimate

Bahrain

Central Asia and North Africa−Middle East

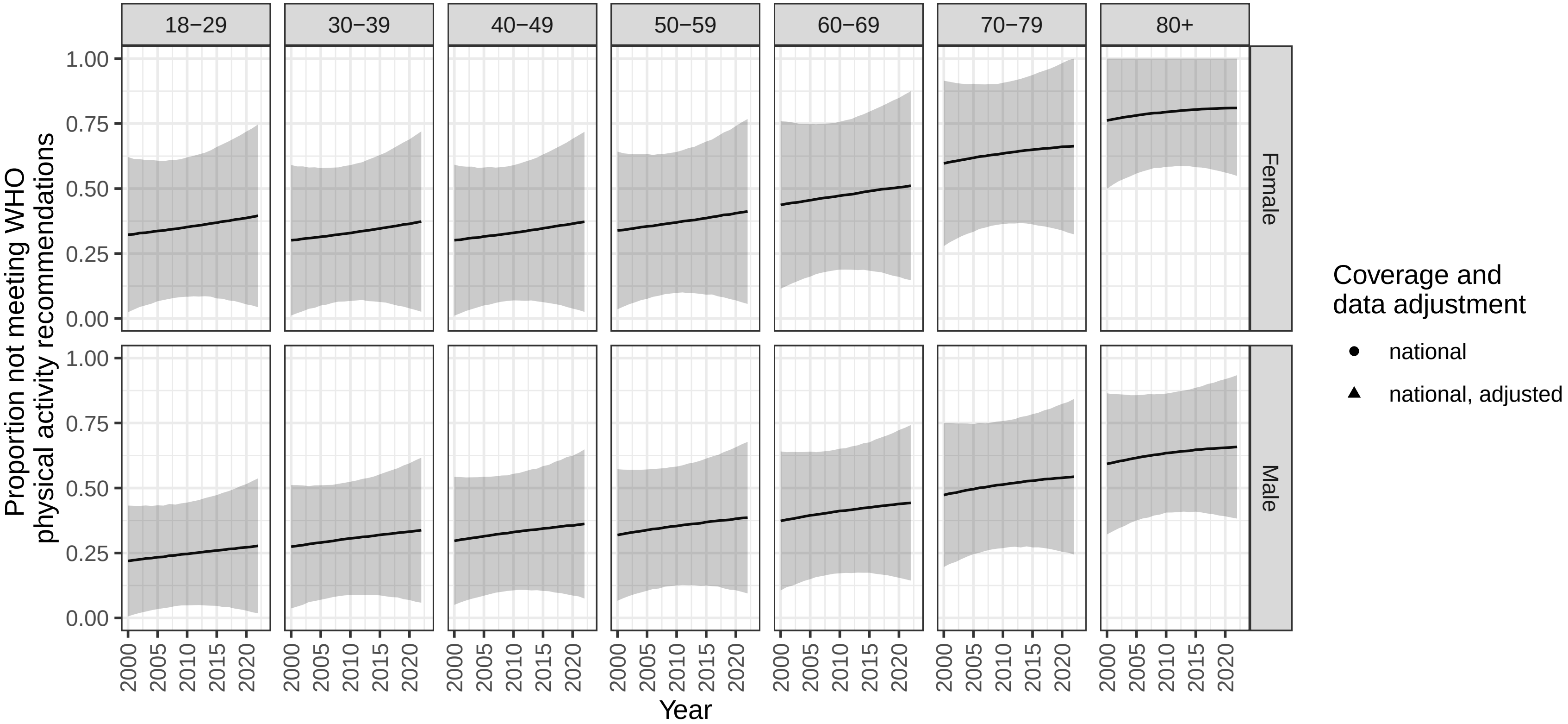

Notes: vertical lines show input data 95% confidence interval; black line shows estimate; shaded area shows 95% uncertainty interval of estimate

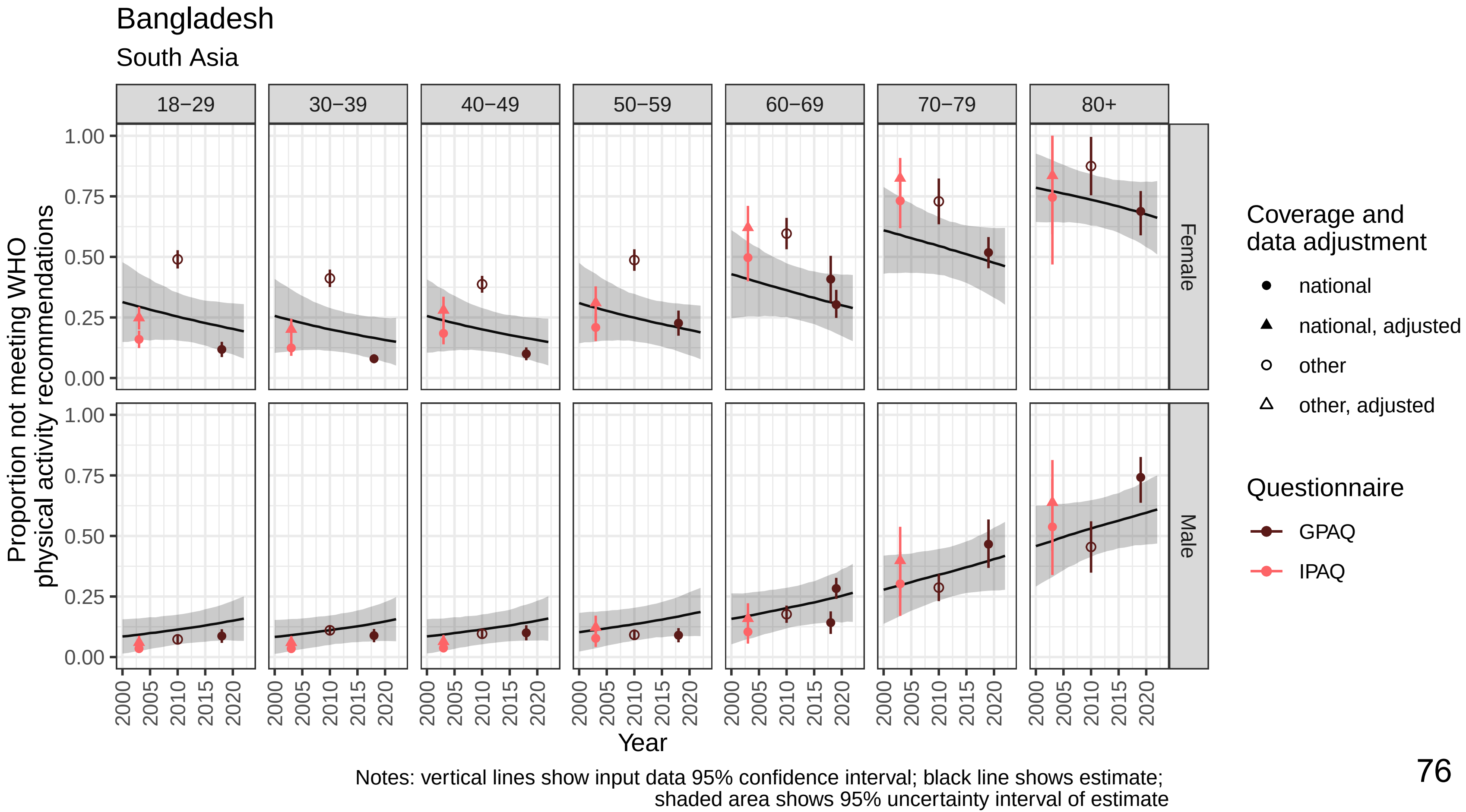
Bangladesh
South Asia
18−29
30−39
40−49
50−59
60−69
70−79
80+
Female
Male
Proportion not meeting WHO physical activity recommendations
1.00
0.75
0.50
0.25
0.00
2000
2005
2010
2015
2020
Year
Coverage and data adjustment
national
national, adjusted
other
other, adjusted
Questionnaire
GPAQ
IPAQ
Notes: vertical lines show input data 95% confidence interval; black line shows estimate;
shaded area shows 95% uncertainty interval of estimate

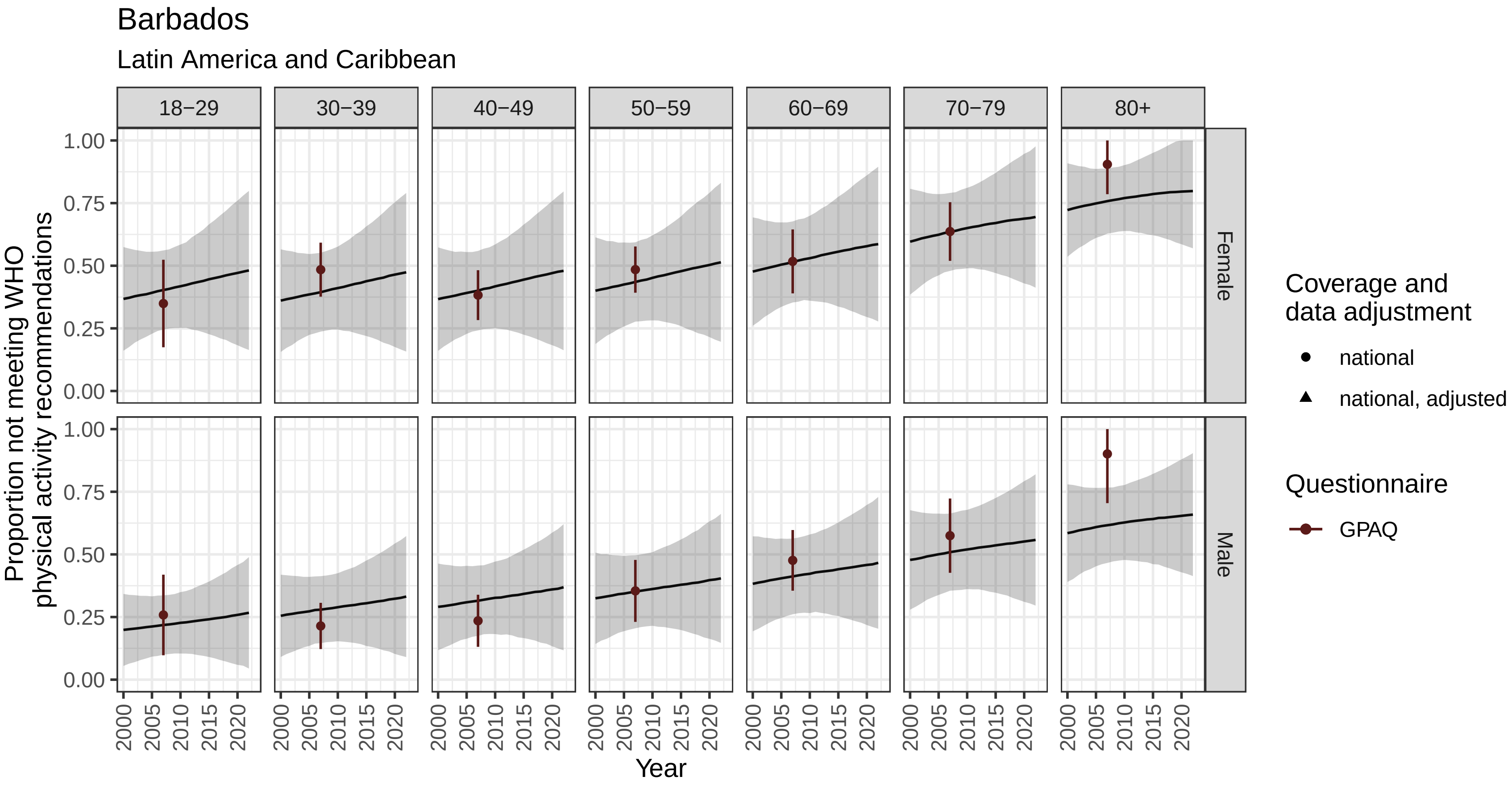

Notes: vertical lines show input data 95% confidence interval; black line shows estimate; shaded area shows 95% uncertainty interval of estimate

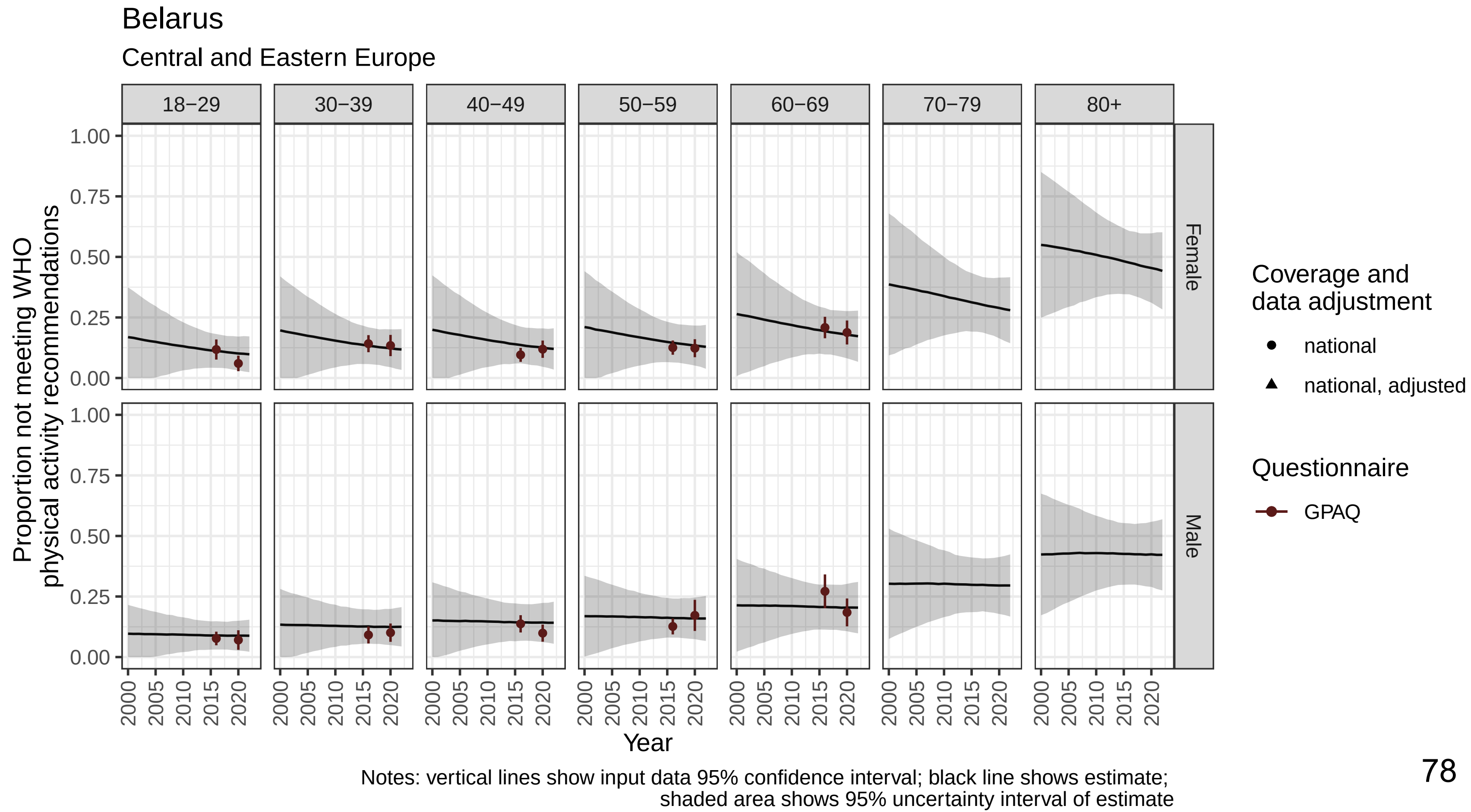
Belarus
Central and Eastern Europe
18−29
30−39
40−49
50−59
60−69
70−79
80+
Female
Male
Proportion not meeting WHO physical activity recommendations
1.00
0.75
0.50
0.25
0.00
2000
2005
2010
2015
2020
Year
Coverage and data adjustment
national
national, adjusted
Questionnaire
GPAQ
Notes: vertical lines show input data 95% confidence interval; black line shows estimate; shaded area shows 95% uncertainty interval of estimate

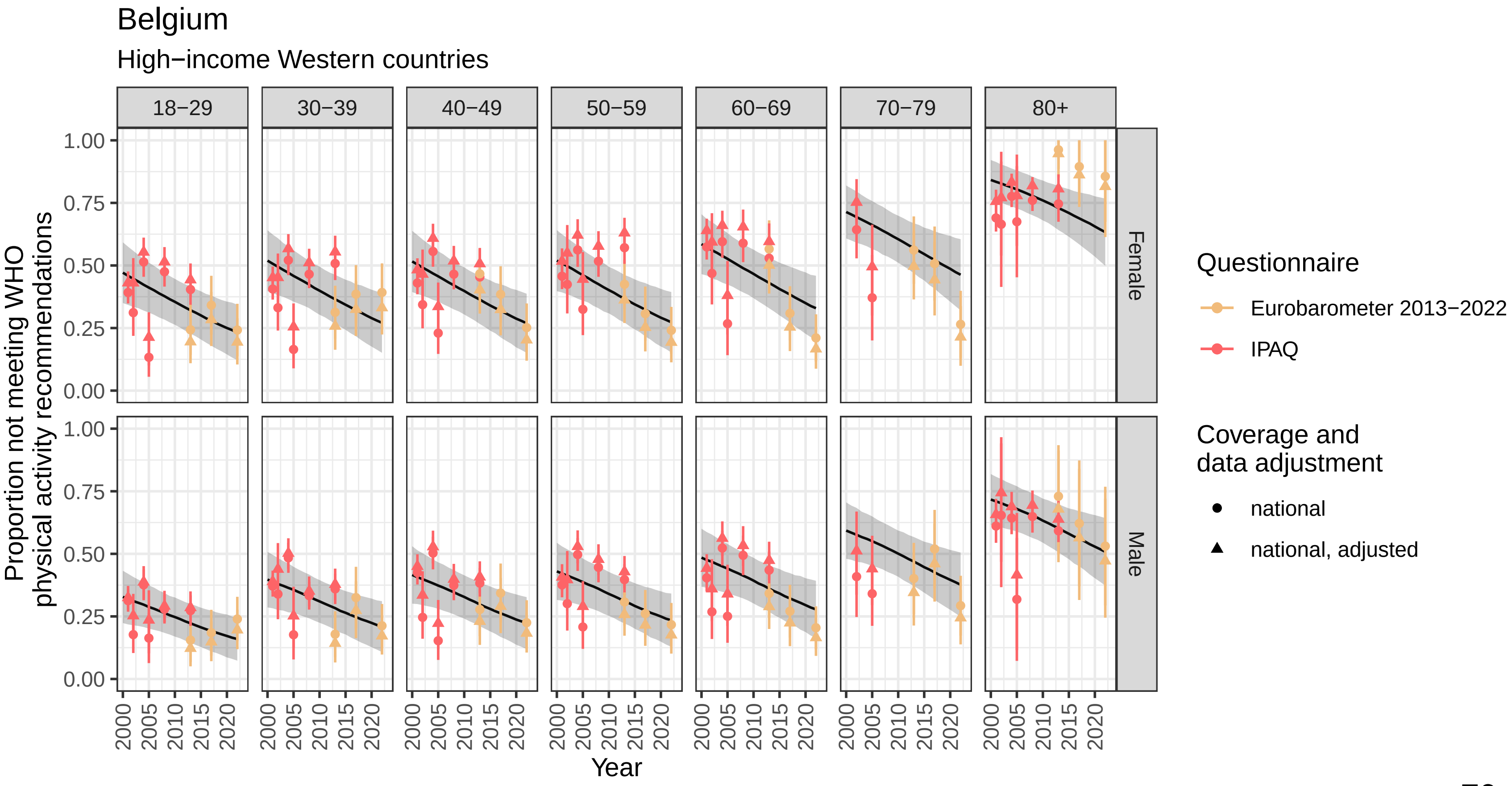

Notes: vertical lines show input data 95% confidence interval; black line shows estimate; shaded area shows 95% uncertainty interval of estimate

Belize

Latin America and Caribbean

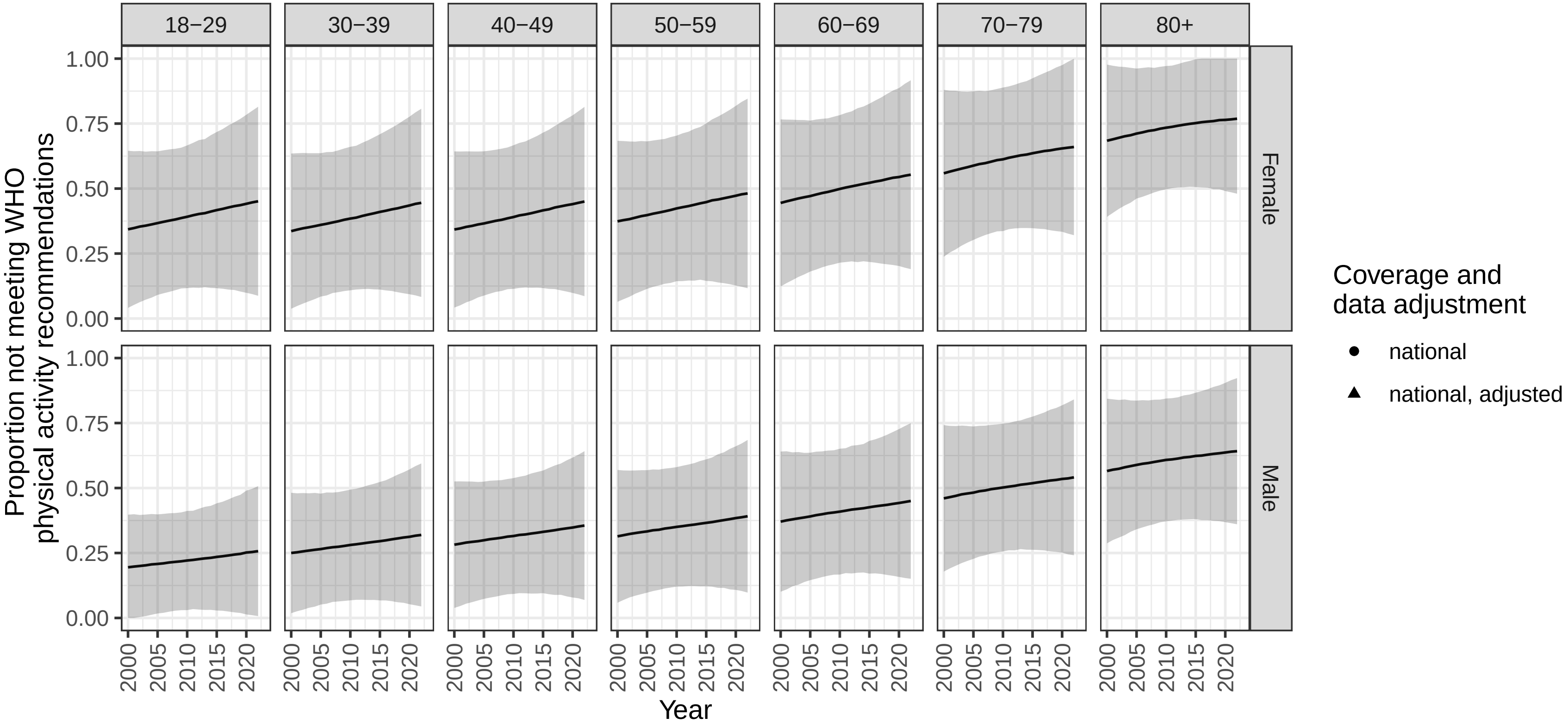

Notes: vertical lines show input data 95% confidence interval; black line shows estimate; shaded area shows 95% uncertainty interval of estimate

Benin

Sub−Saharan Africa

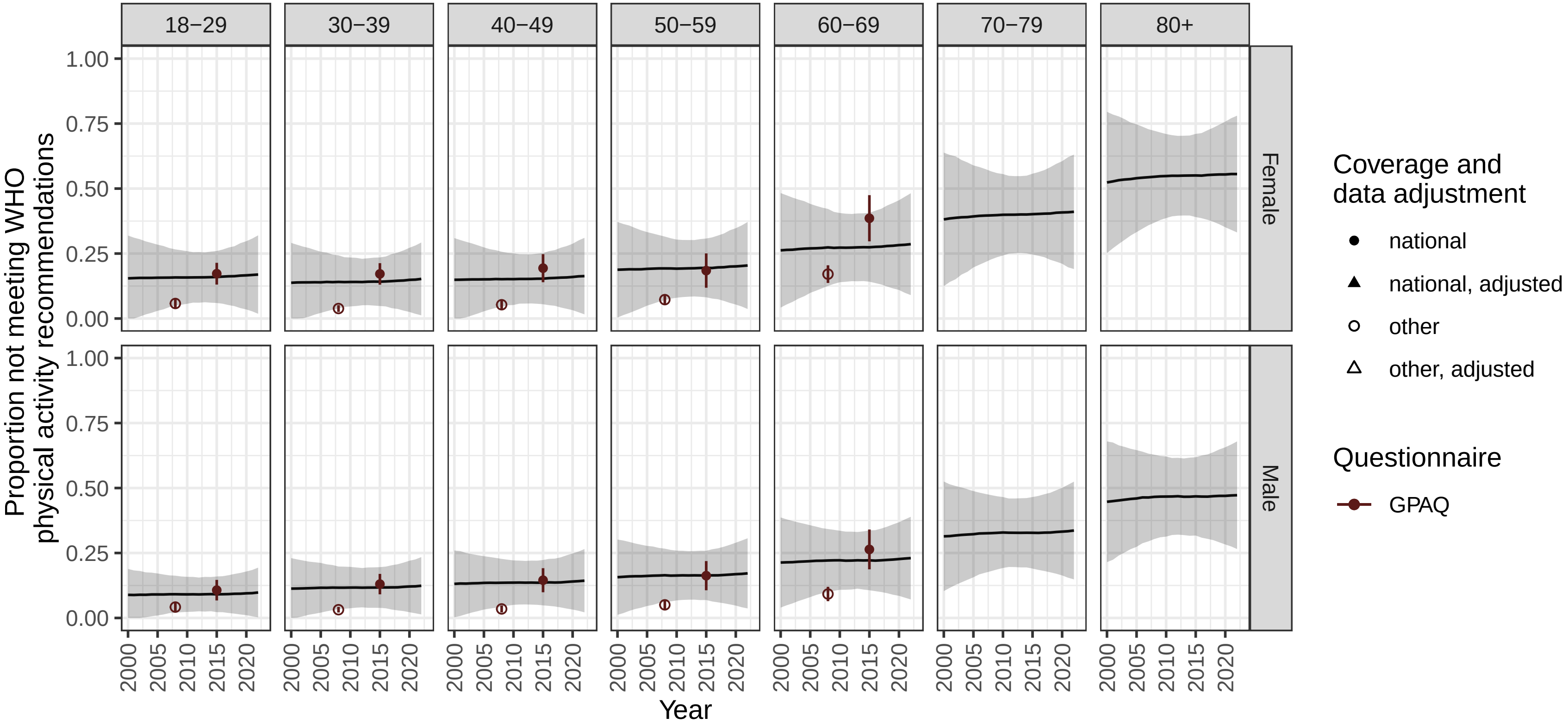

Notes: vertical lines show input data 95% confidence interval; black line shows estimate; shaded area shows 95% uncertainty interval of estimate

Bhutan

South Asia

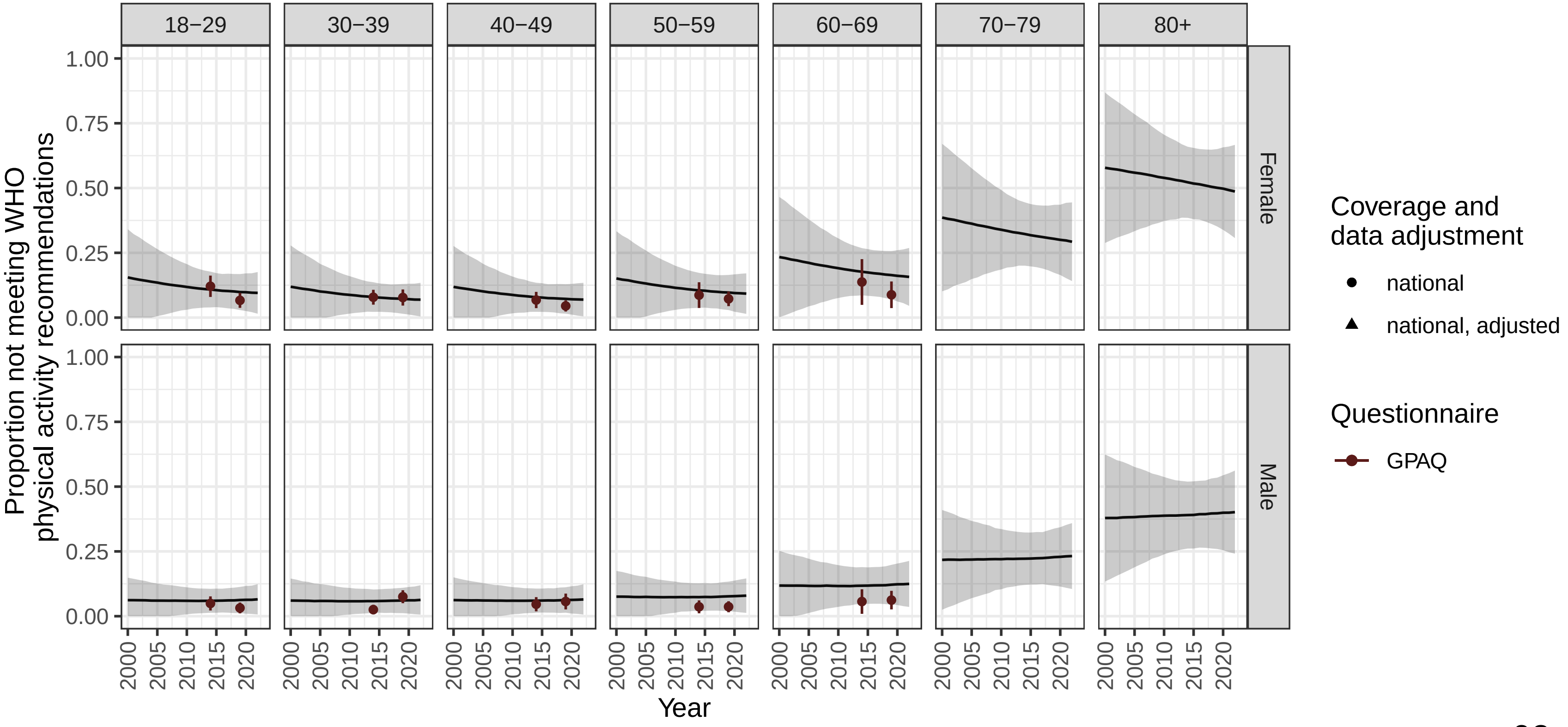

Notes: vertical lines show input data 95% confidence interval; black line shows estimate; shaded area shows 95% uncertainty interval of estimate

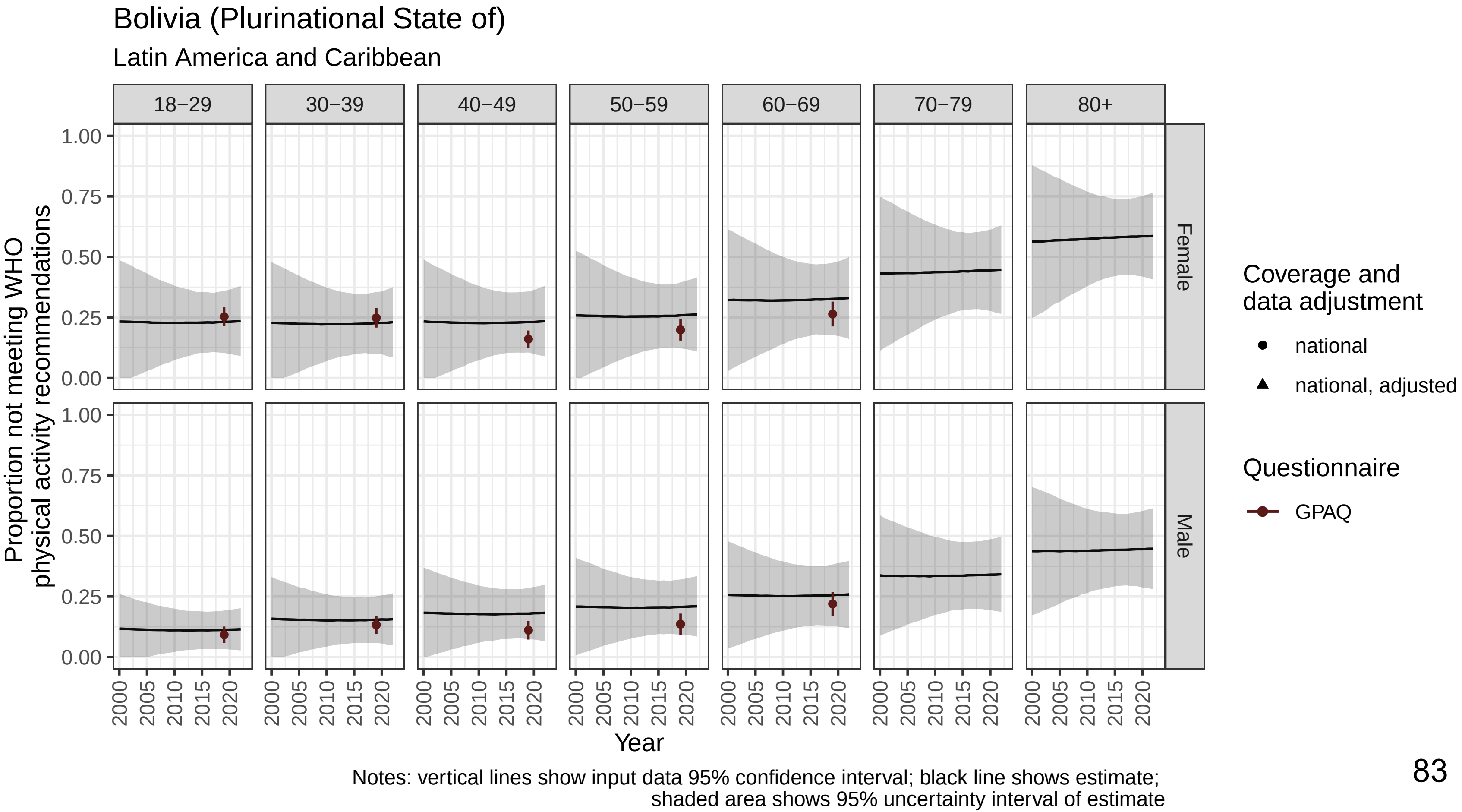

Bolivia (Plurinational State of)
Latin America and Caribbean
18−29
30−39
40−49
50−59
60−69
70−79
80+
Female
Male
Proportion not meeting WHO physical activity recommendations
0.00
0.25
0.50
0.75
1.00
2000
2005
2010
2015
2020
Year
Coverage and data adjustment
national
national, adjusted
Questionnaire
GPAQ
Notes: vertical lines show input data 95% confidence interval; black line shows estimate; shaded area shows 95% uncertainty interval of estimate

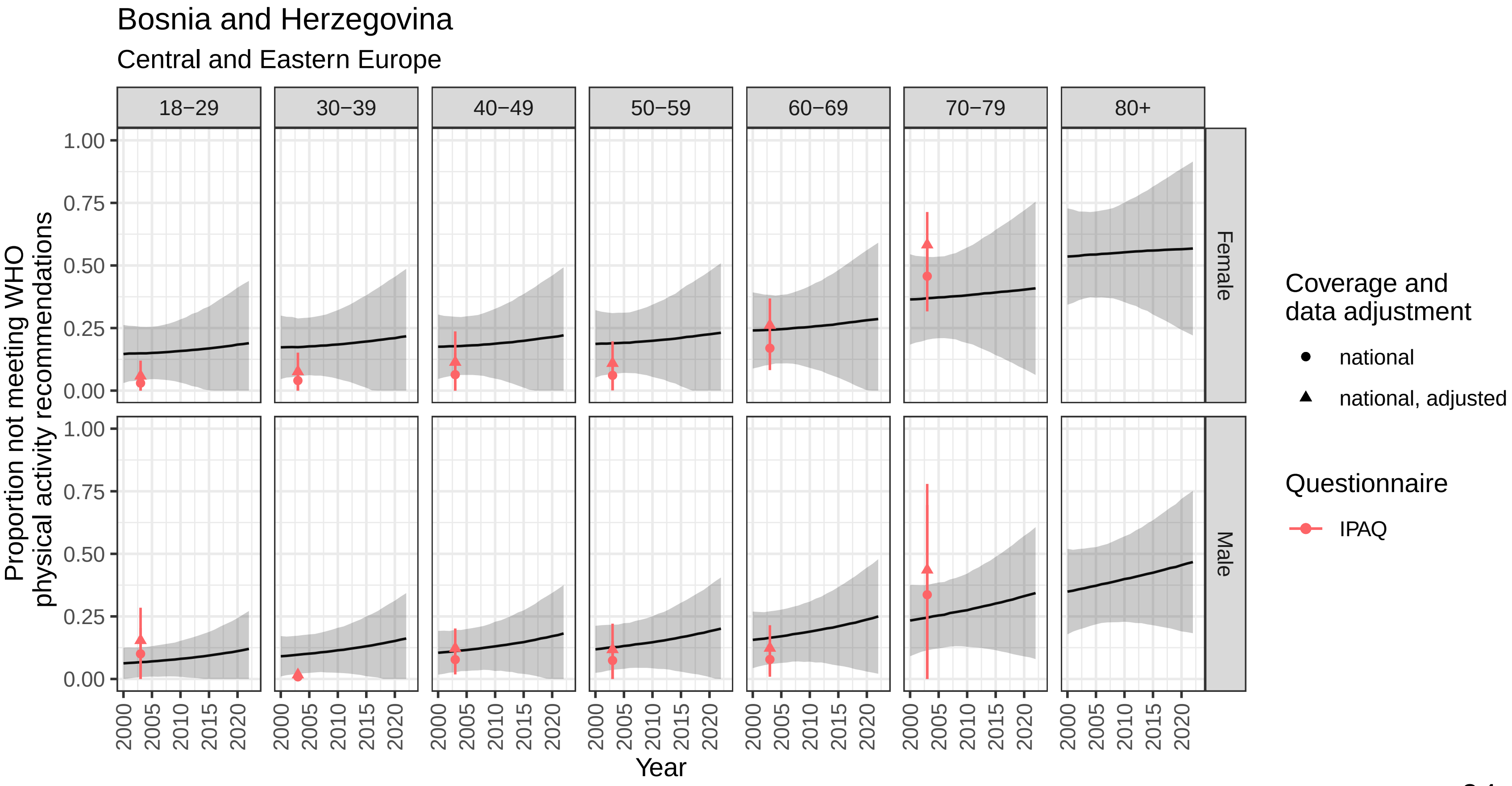

Notes: vertical lines show input data 95% confidence interval; black line shows estimate; shaded area shows 95% uncertainty interval of estimate

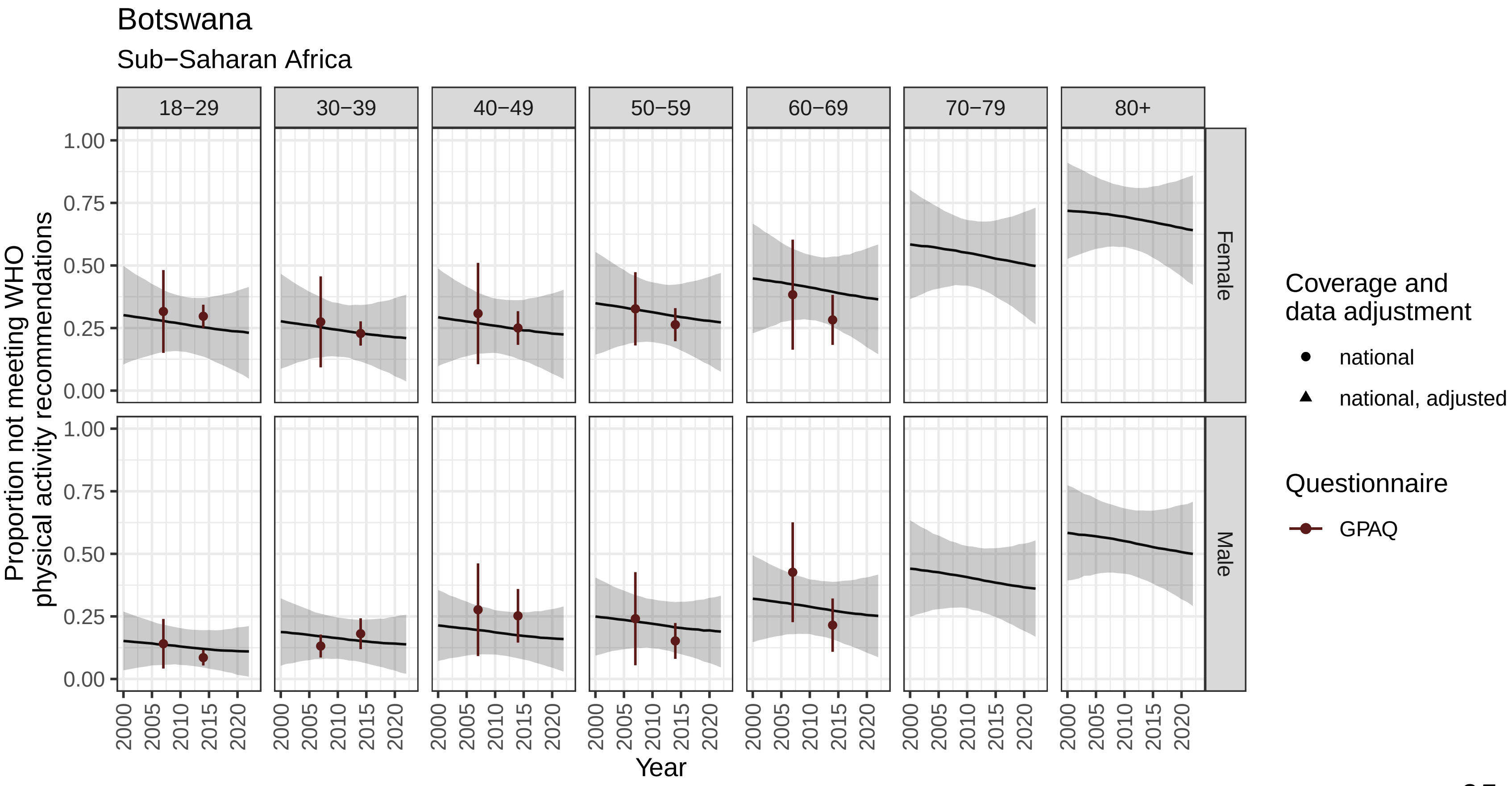

Notes: vertical lines show input data 95% confidence interval; black line shows estimate; shaded area shows 95% uncertainty interval of estimate

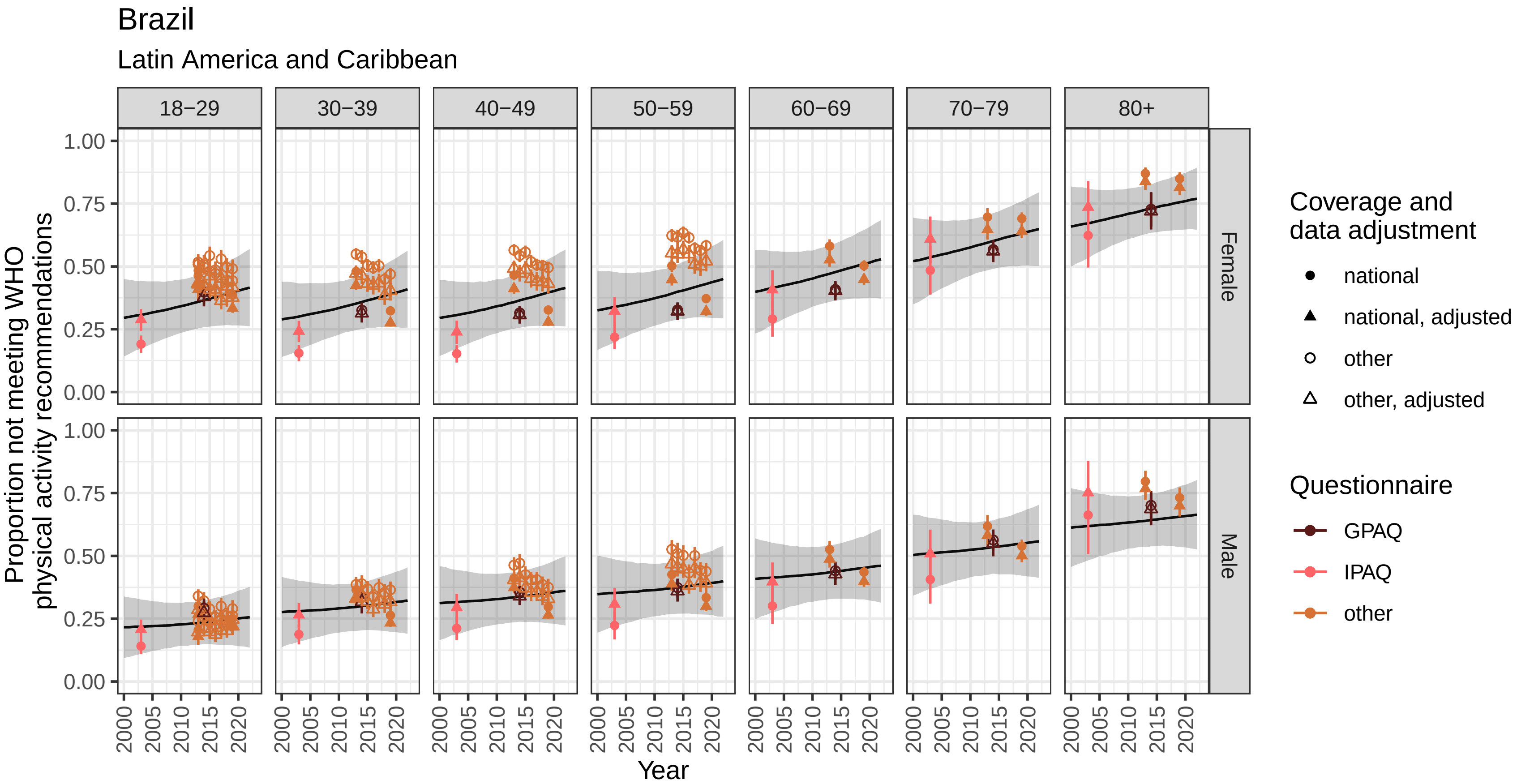

Notes: vertical lines show input data 95% confidence interval; black line shows estimate; shaded area shows 95% uncertainty interval of estimate

Brunei Darussalam

East and South East Asia

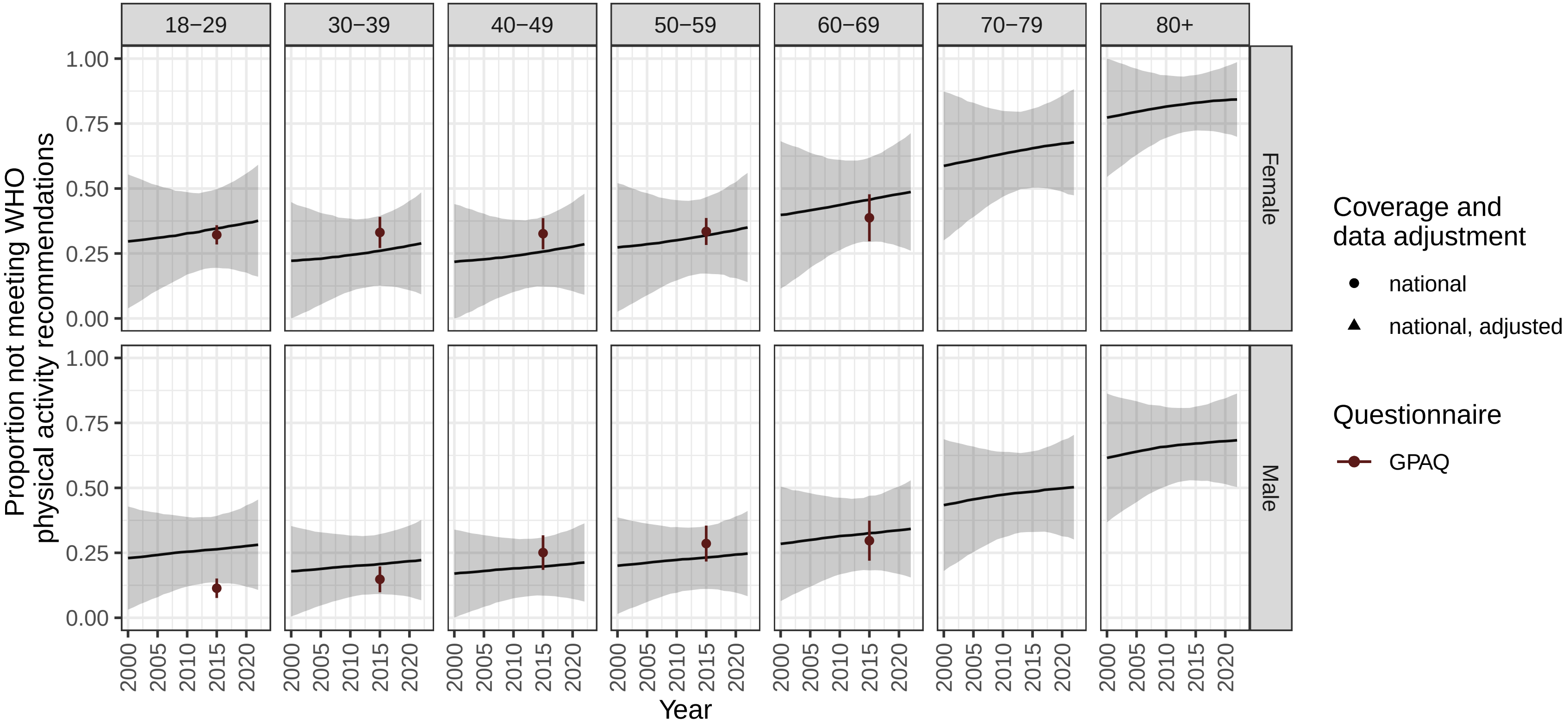

Notes: vertical lines show input data 95% confidence interval; black line shows estimate; shaded area shows 95% uncertainty interval of estimate

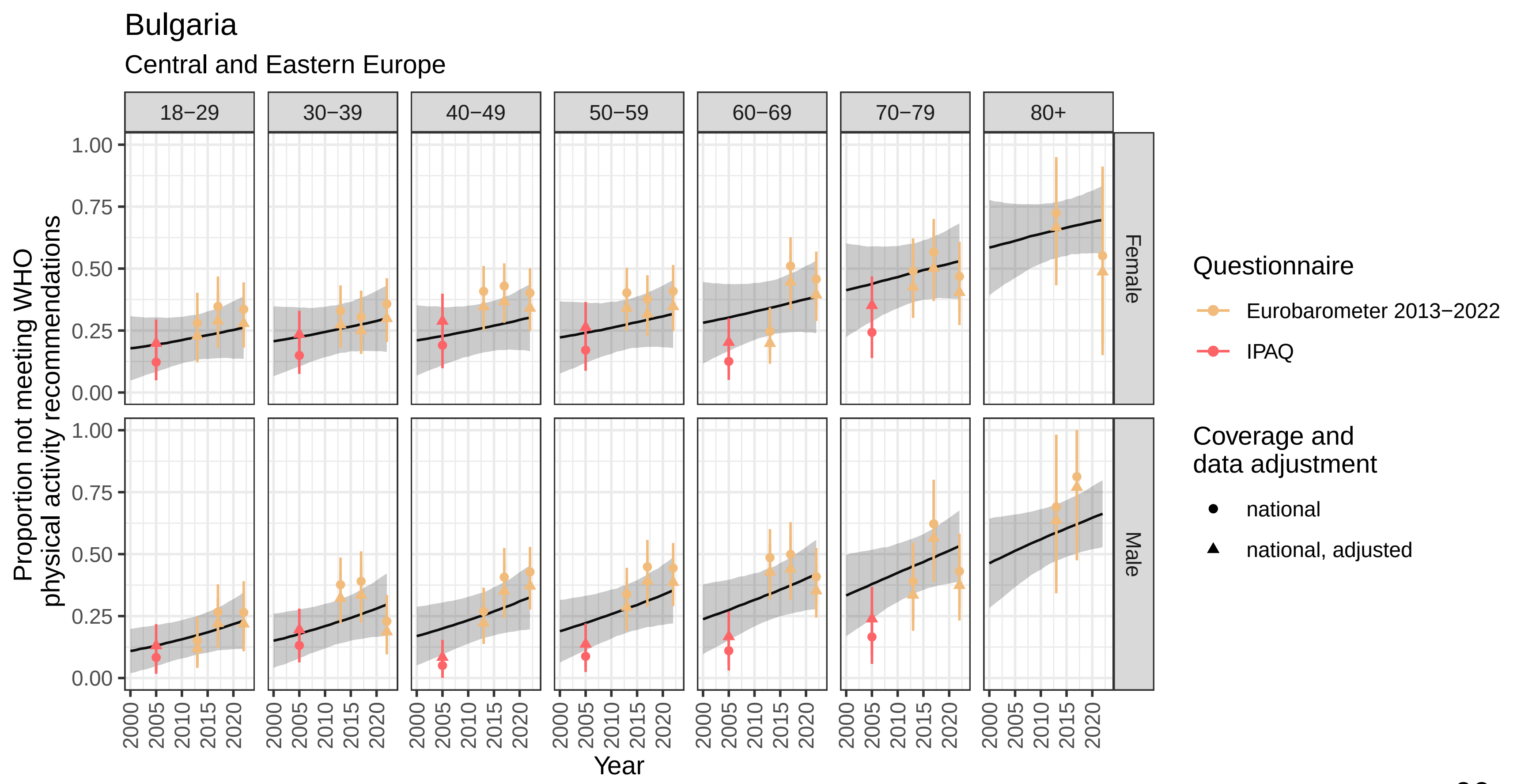

Notes: vertical lines show input data 95% confidence interval; black line shows estimate; shaded area shows 95% uncertainty interval of estimate

Burkina Faso

Sub−Saharan Africa

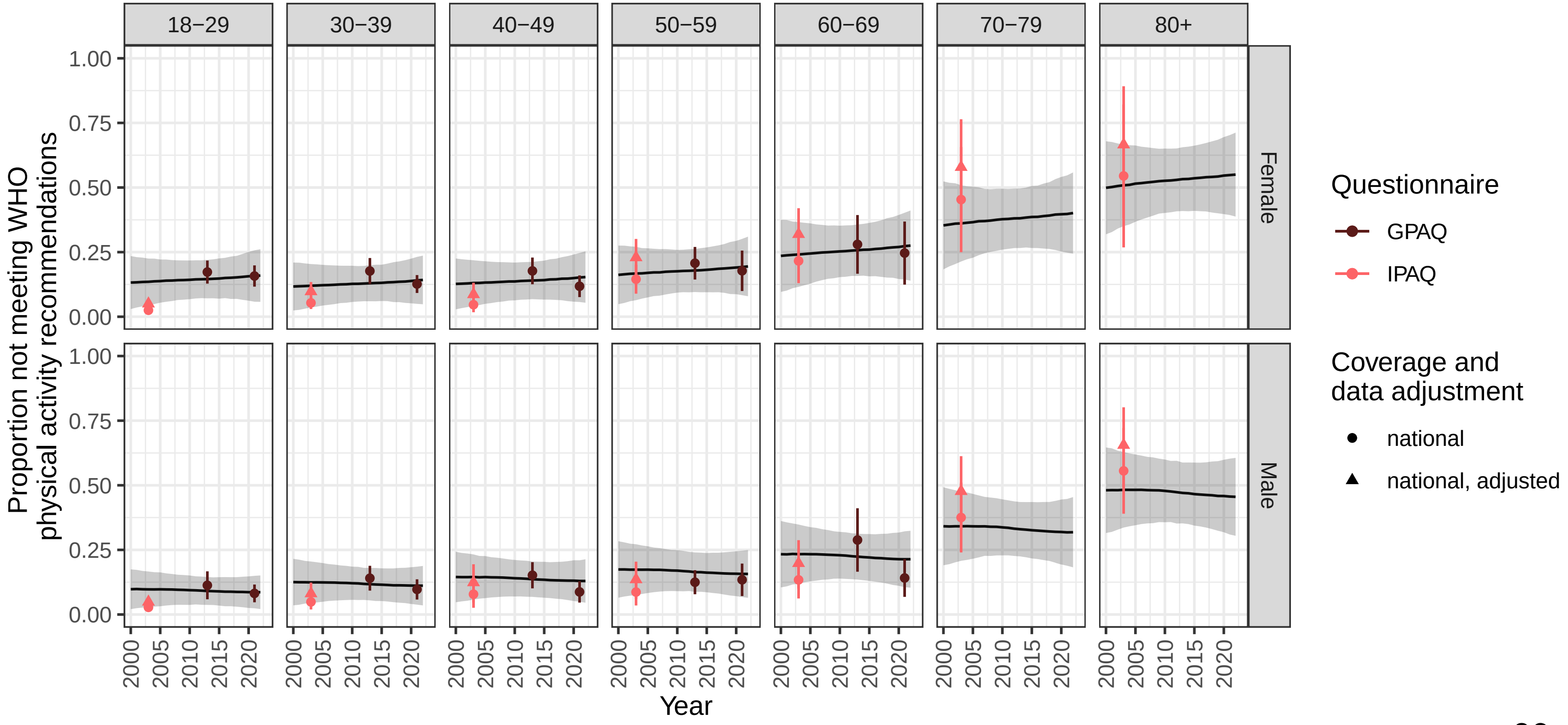

Notes: vertical lines show input data 95% confidence interval; black line shows estimate; shaded area shows 95% uncertainty interval of estimate

Burundi

Sub−Saharan Africa

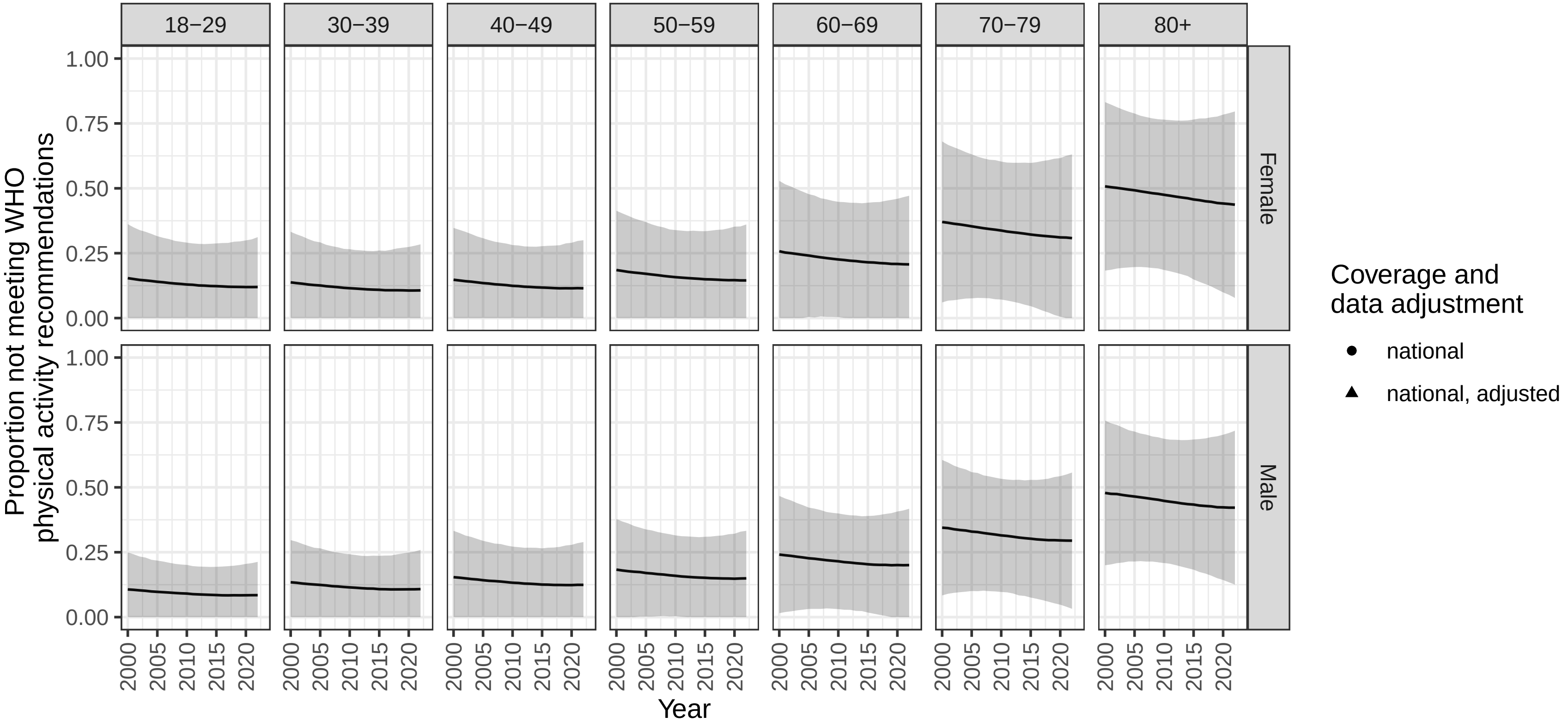

Notes: vertical lines show input data 95% confidence interval; black line shows estimate; shaded area shows 95% uncertainty interval of estimate

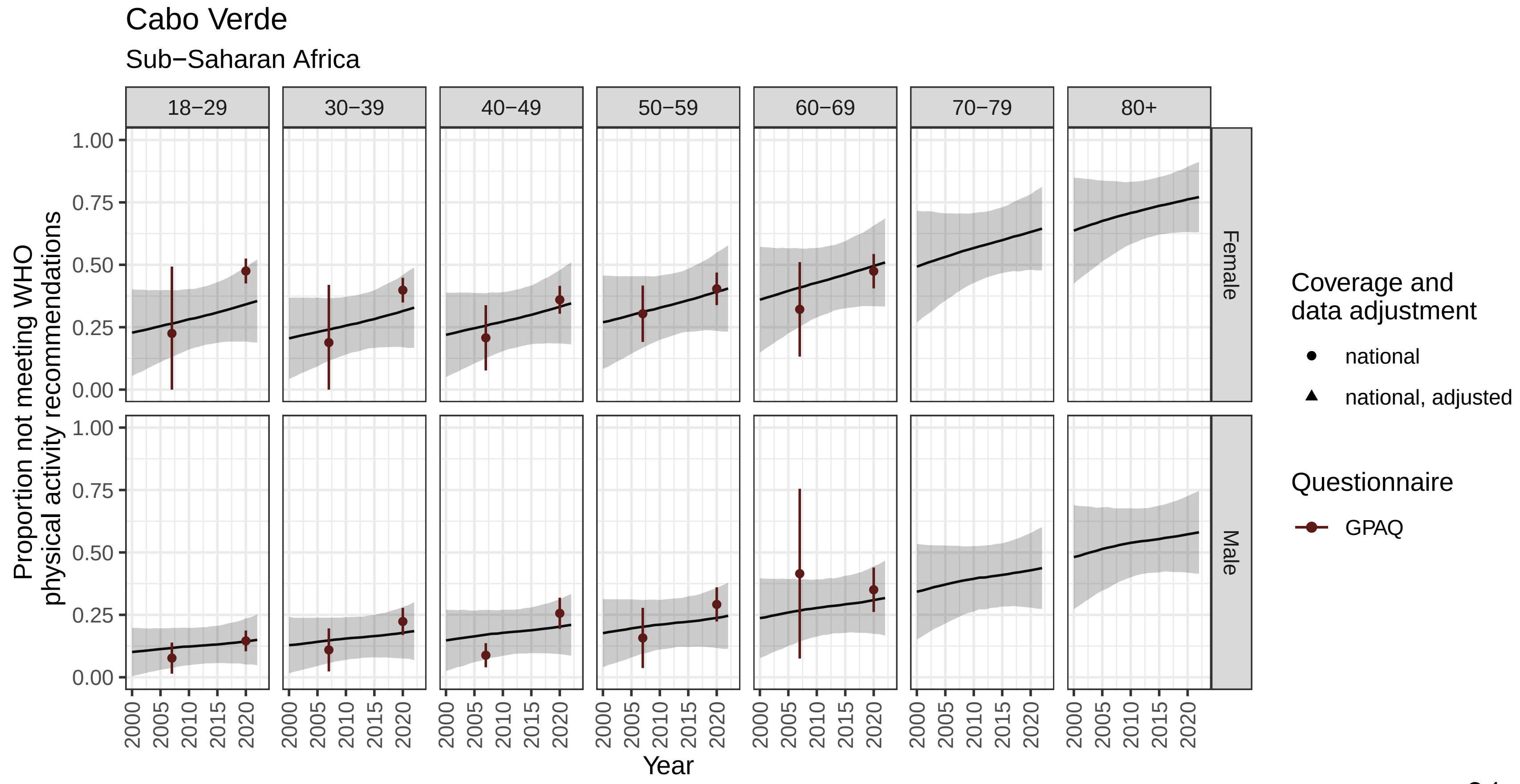

Notes: vertical lines show input data 95% confidence interval; black line shows estimate; shaded area shows 95% uncertainty interval of estimate

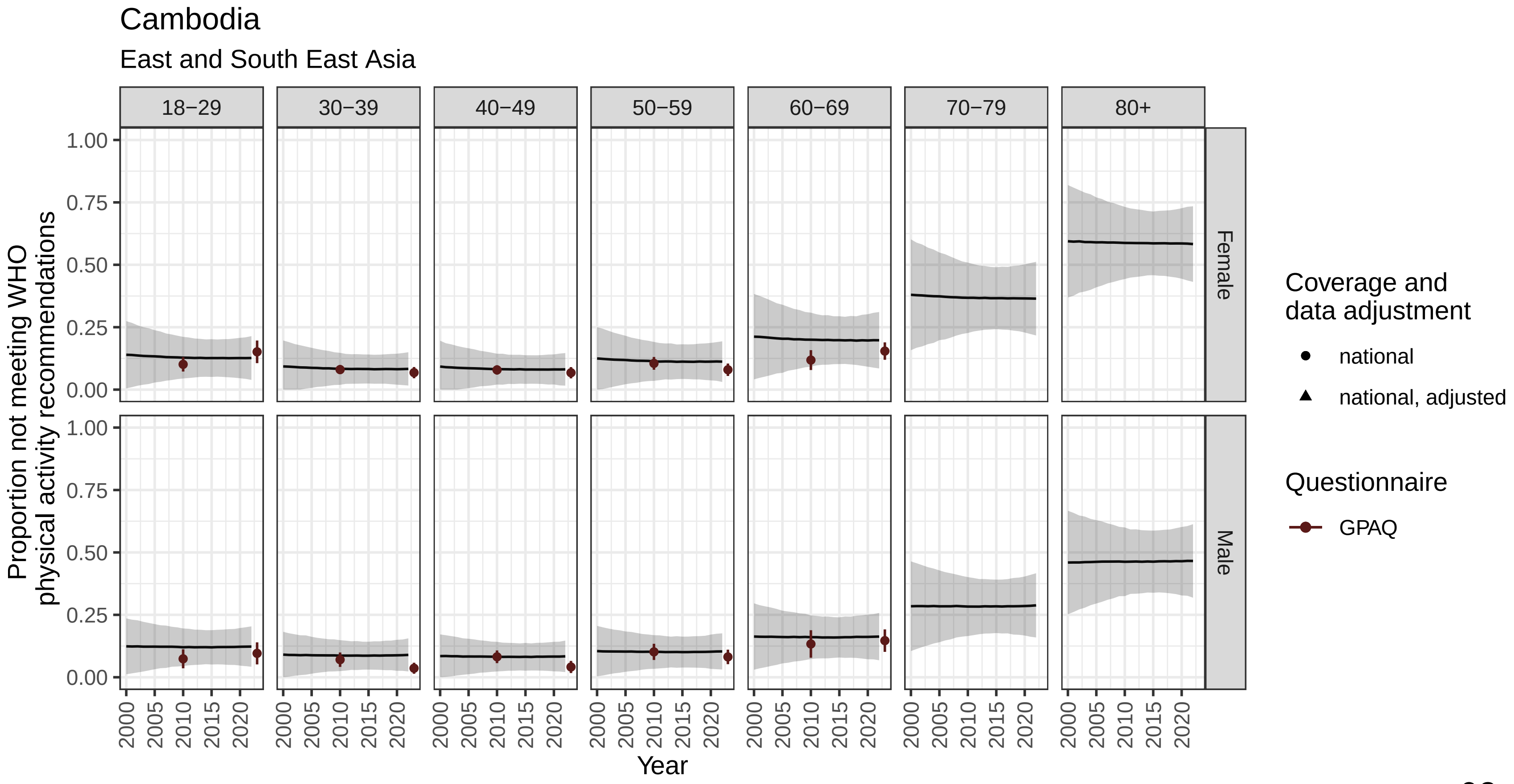

Notes: vertical lines show input data 95% confidence interval; black line shows estimate; shaded area shows 95% uncertainty interval of estimate

Cameroon

Sub−Saharan Africa

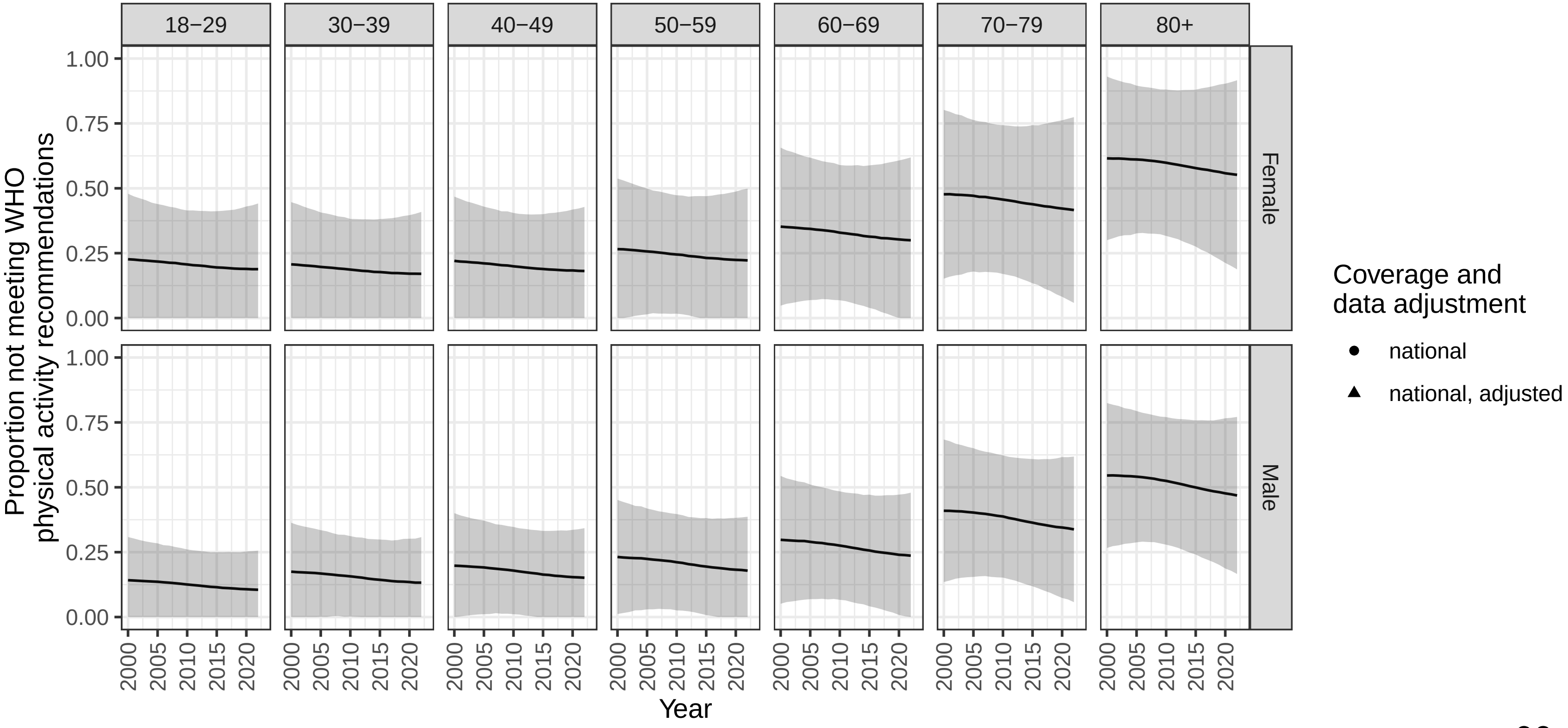

Notes: vertical lines show input data 95% confidence interval; black line shows estimate; shaded area shows 95% uncertainty interval of estimate

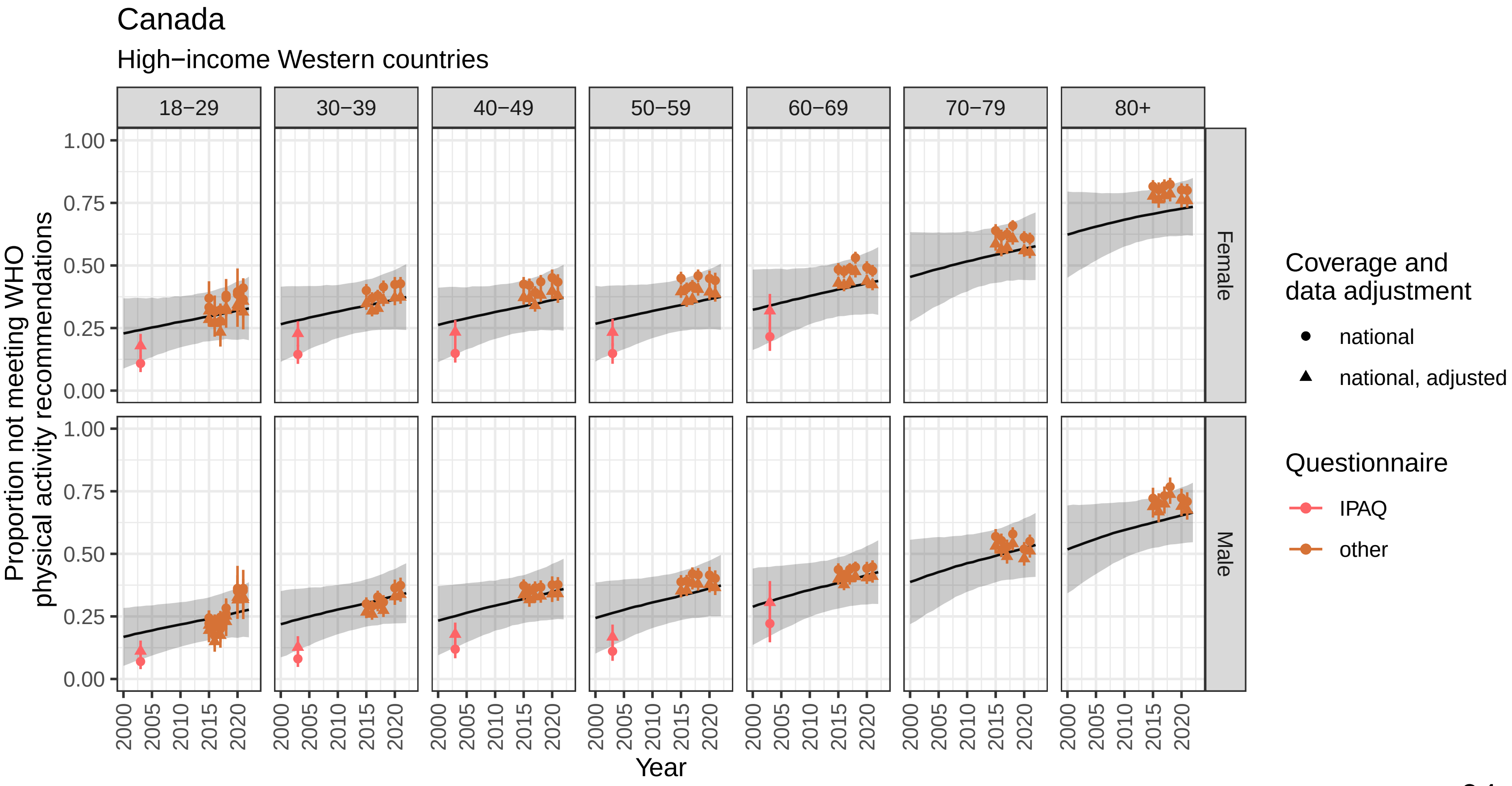

Notes: vertical lines show input data 95% confidence interval; black line shows estimate; shaded area shows 95% uncertainty interval of estimate

Central African Republic

Sub−Saharan Africa

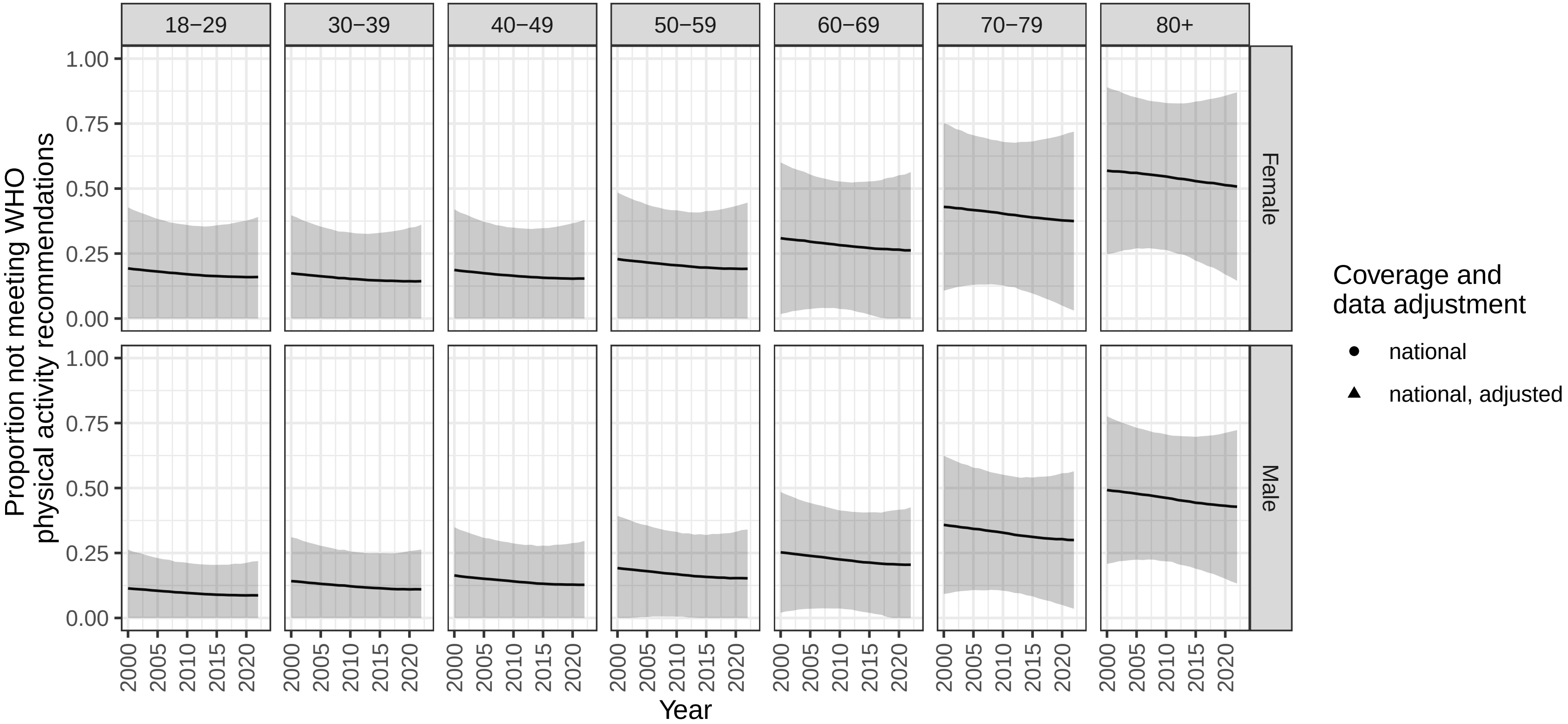

Notes: vertical lines show input data 95% confidence interval; black line shows estimate; shaded area shows 95% uncertainty interval of estimate

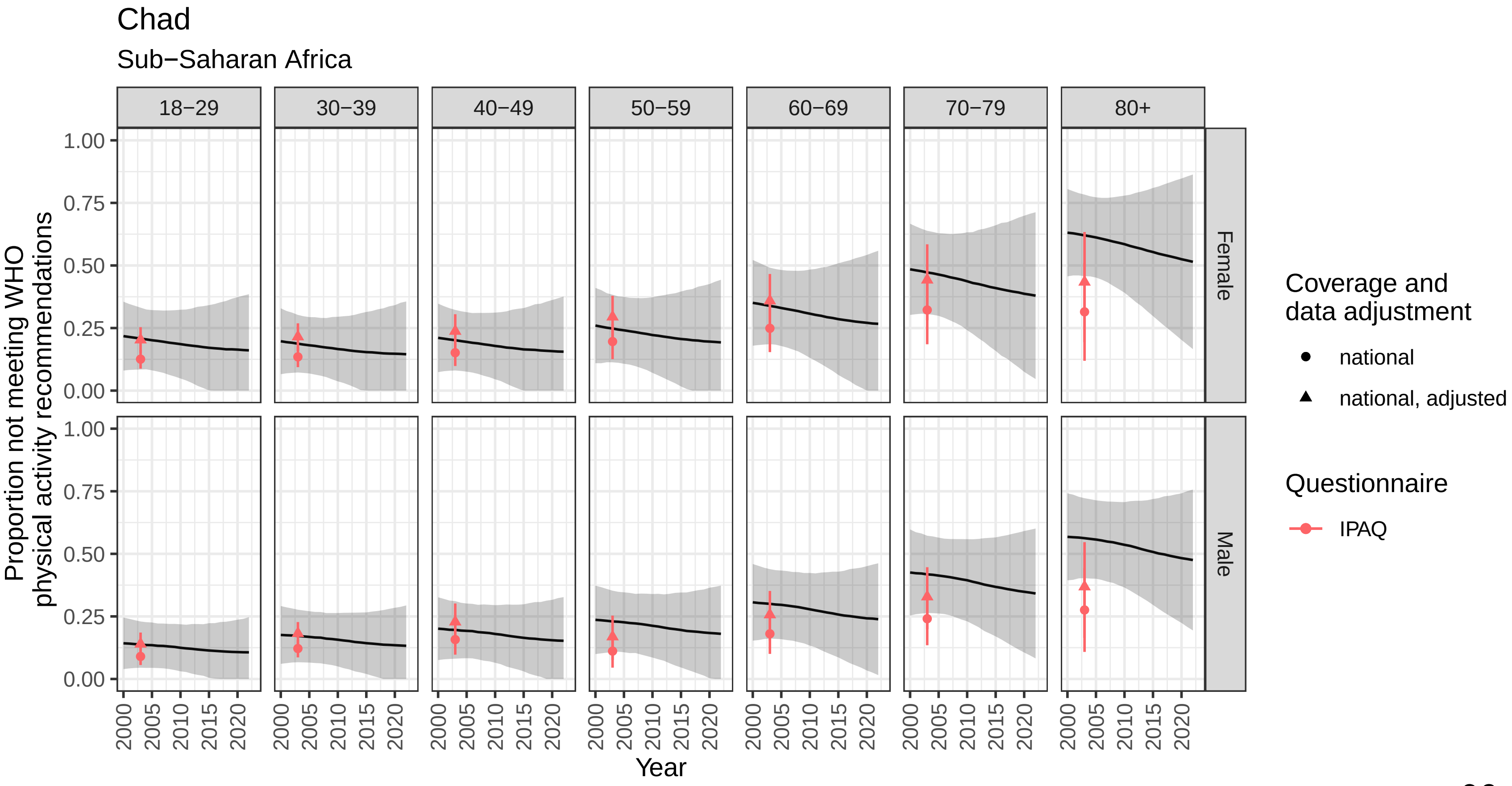

Notes: vertical lines show input data 95% confidence interval; black line shows estimate; shaded area shows 95% uncertainty interval of estimate

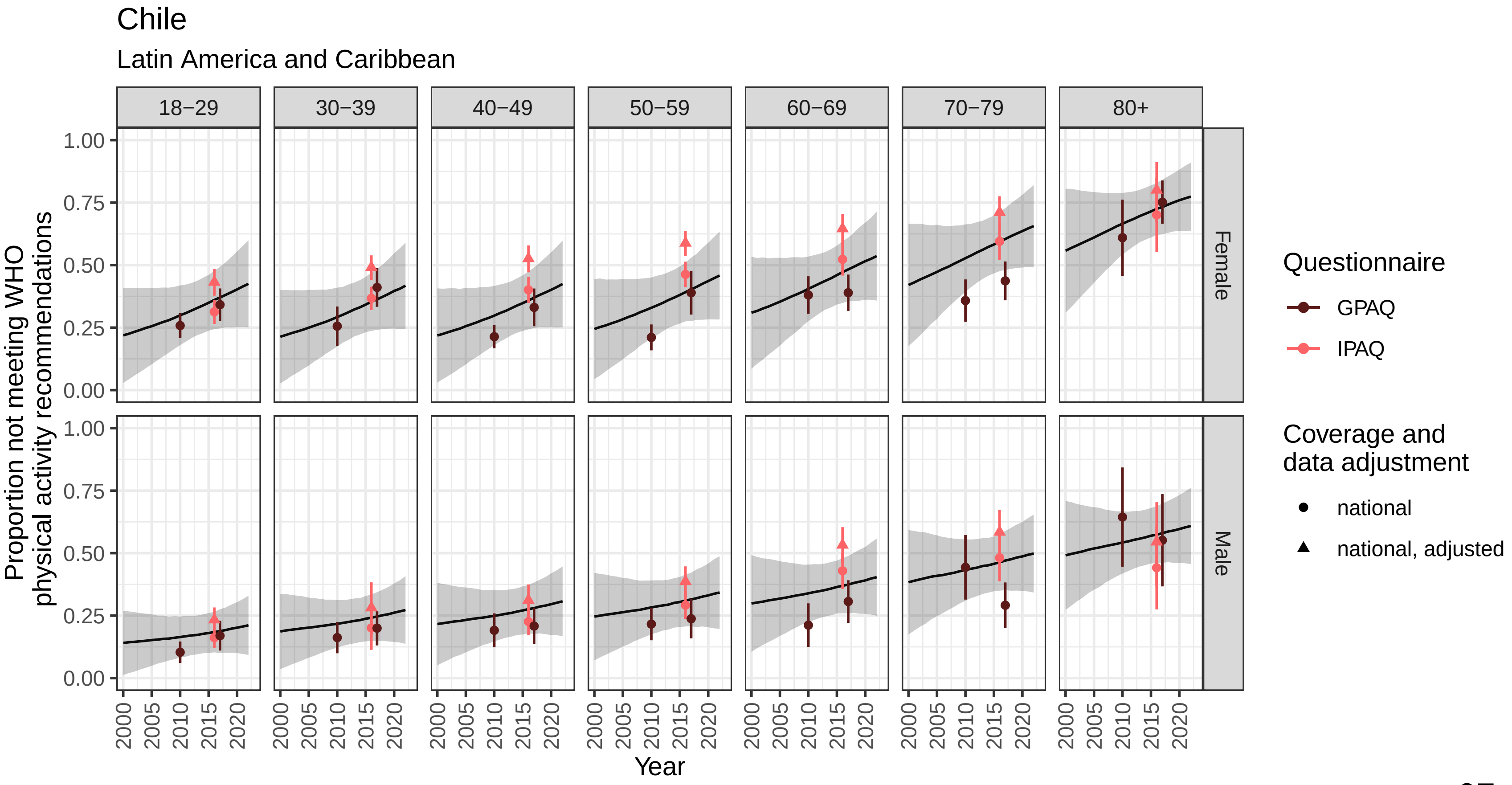

Notes: vertical lines show input data 95% confidence interval; black line shows estimate; shaded area shows 95% uncertainty interval of estimate

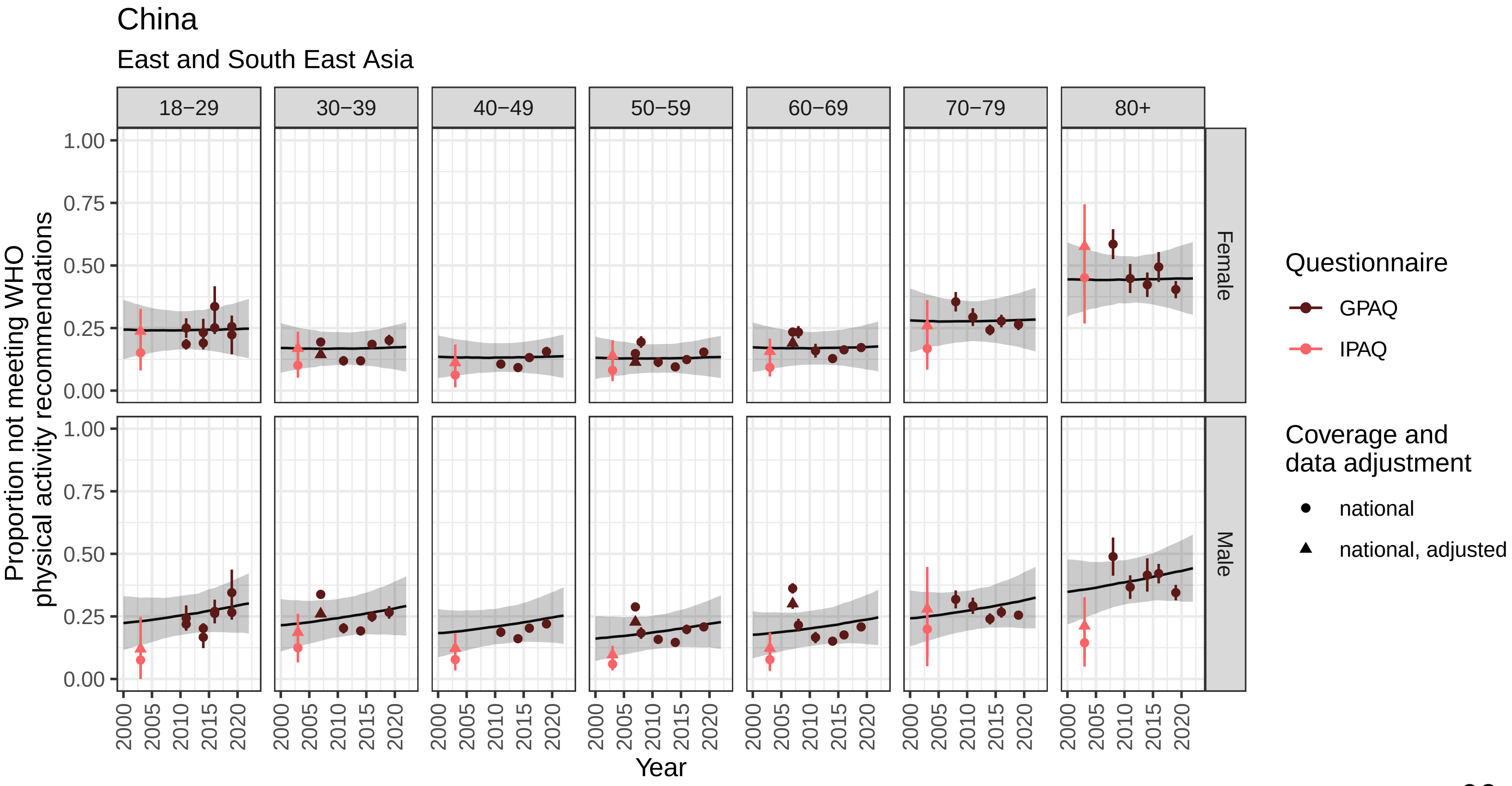

Notes: vertical lines show input data 95% confidence interval; black line shows estimate; shaded area shows 95% uncertainty interval of estimate

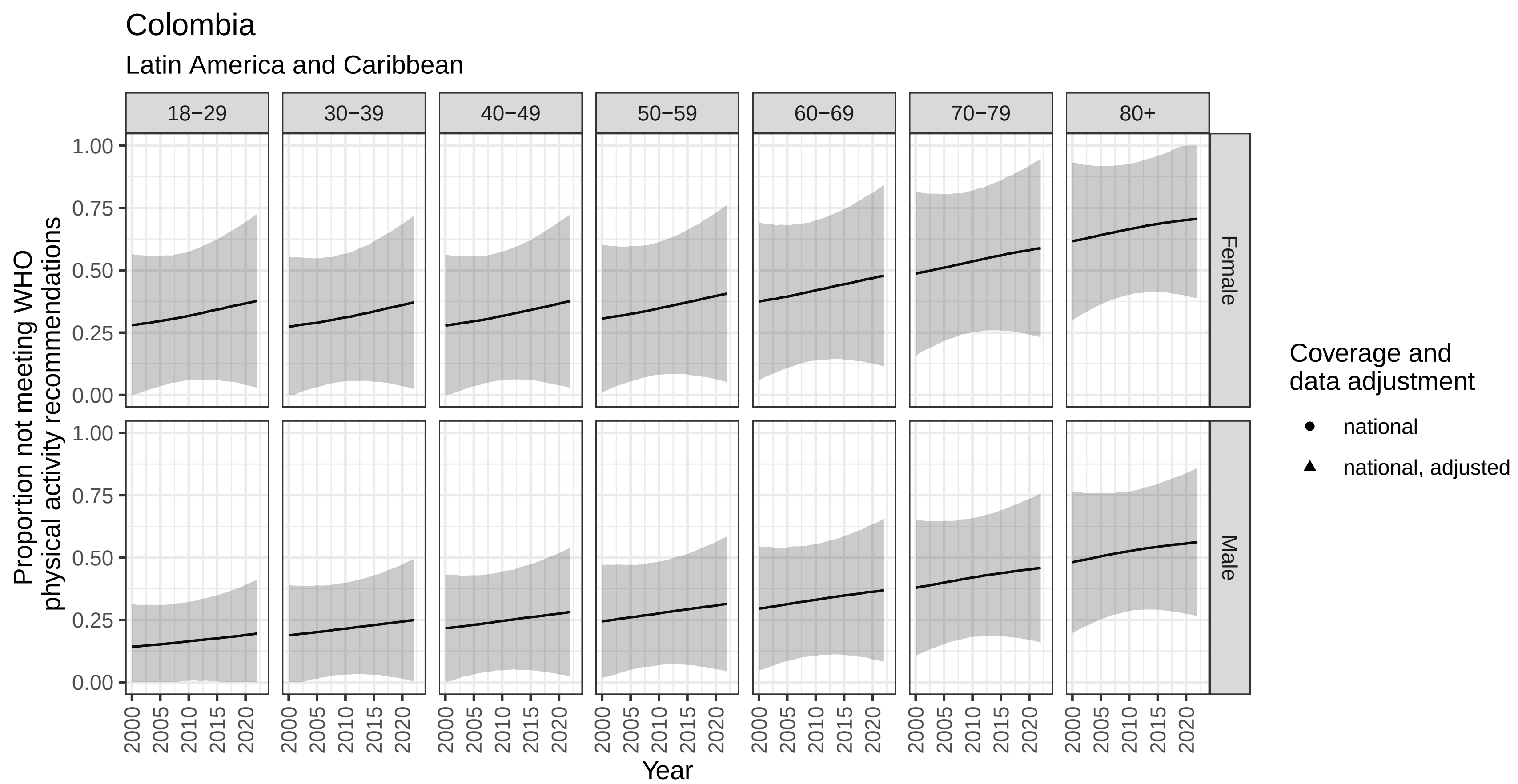

Notes: vertical lines show input data 95% confidence interval; black line shows estimate; shaded area shows 95% uncertainty interval of estimate

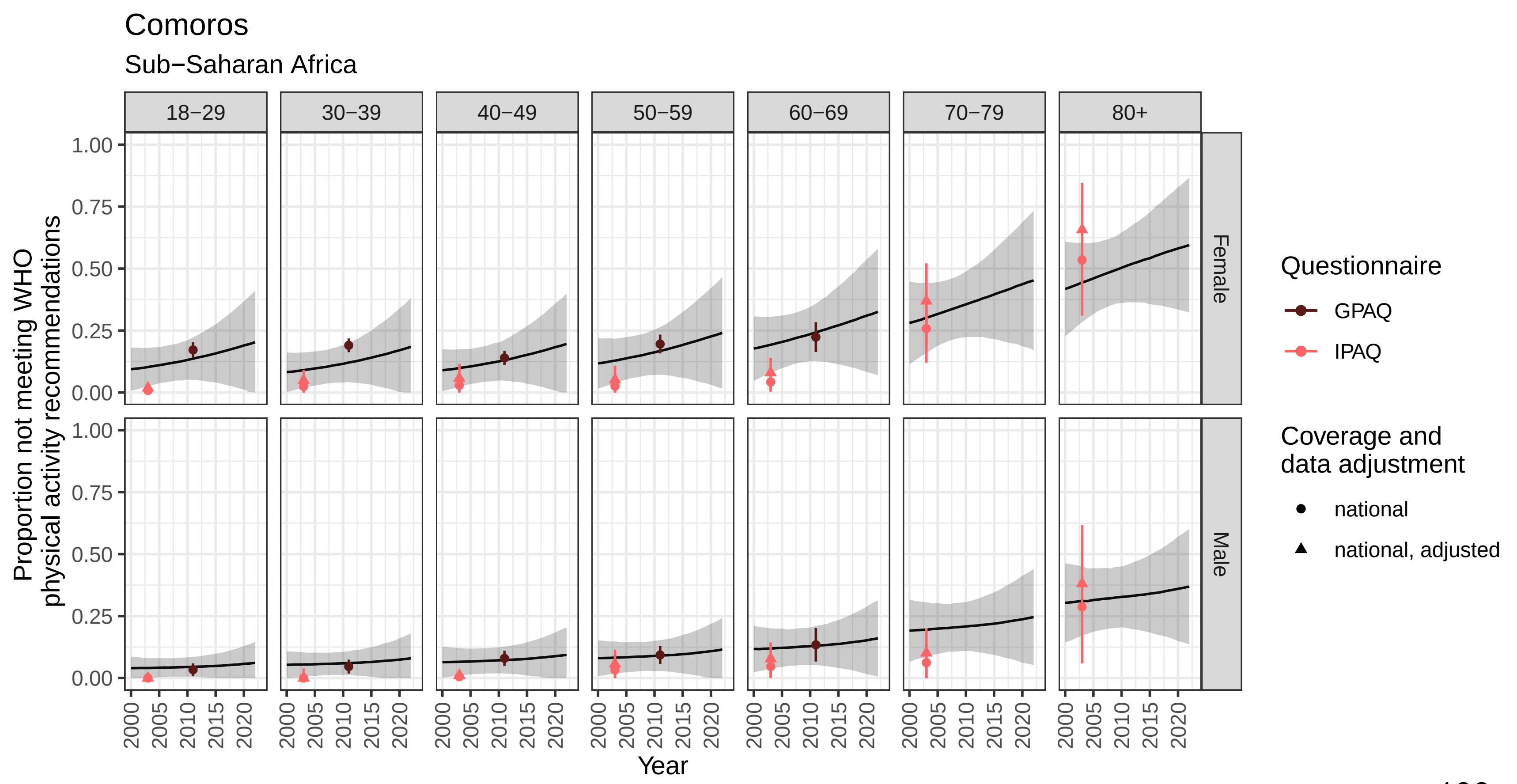

Notes: vertical lines show input data 95% confidence interval; black line shows estimate; shaded area shows 95% uncertainty interval of estimate

Congo

Sub−Saharan Africa

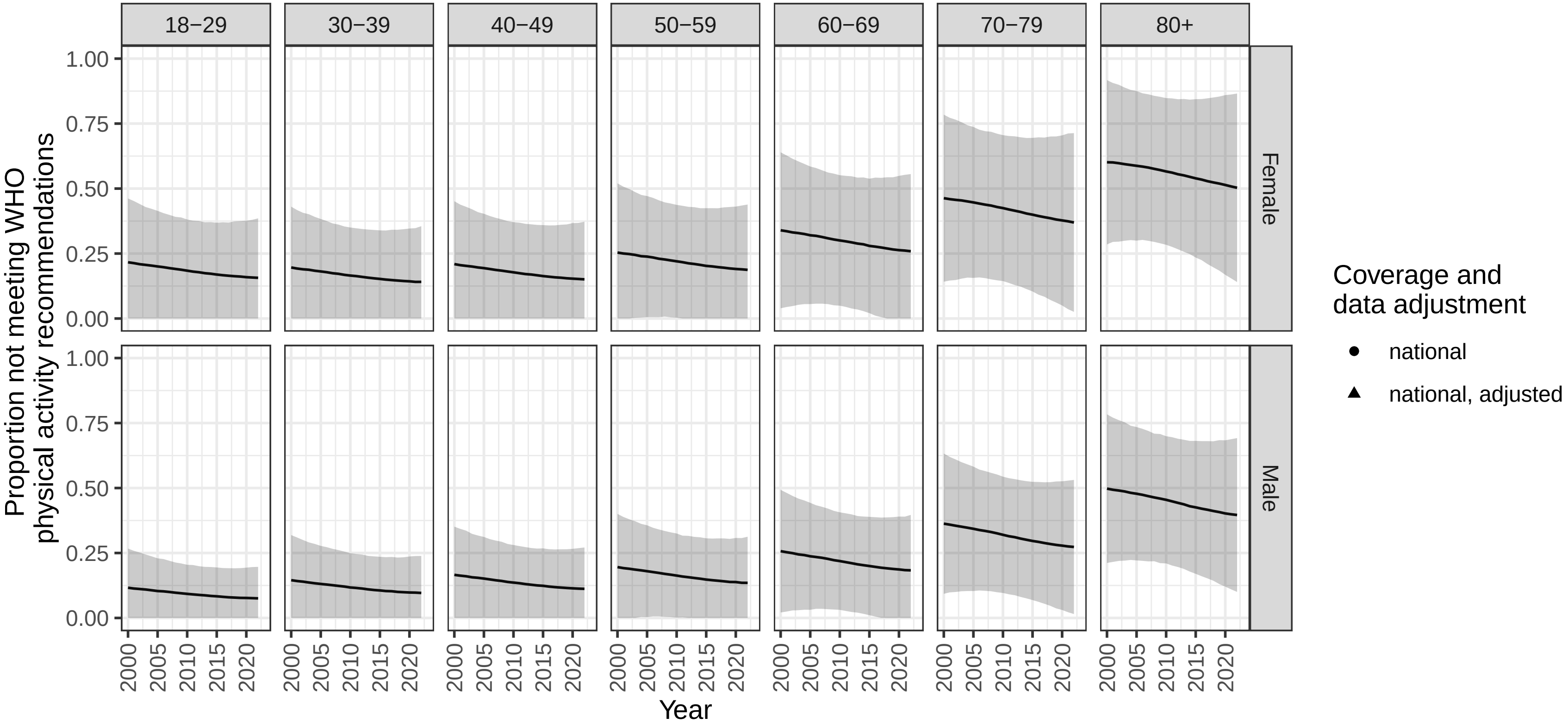

Notes: vertical lines show input data 95% confidence interval; black line shows estimate; shaded area shows 95% uncertainty interval of estimate

Cook Islands

Oceania

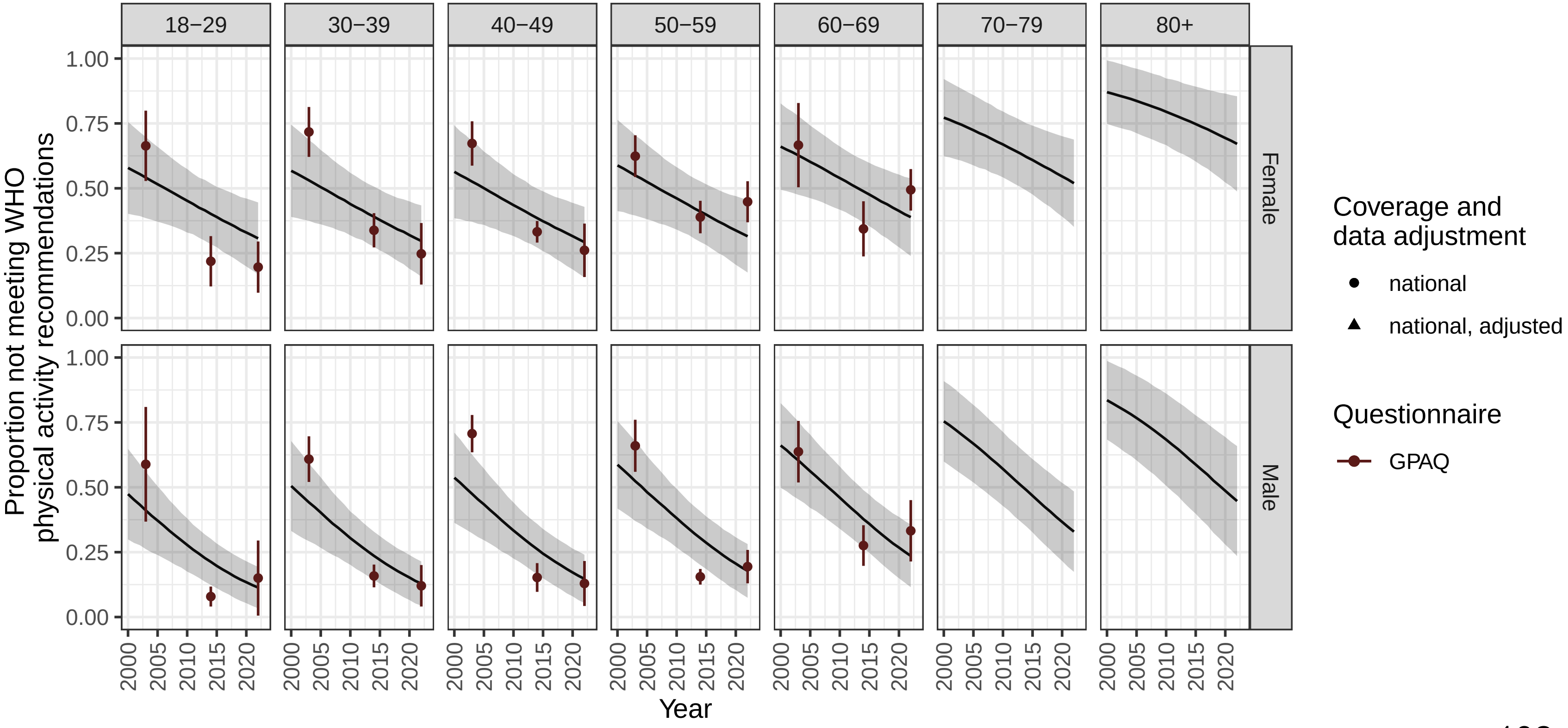

Notes: vertical lines show input data 95% confidence interval; black line shows estimate; shaded area shows 95% uncertainty interval of estimate

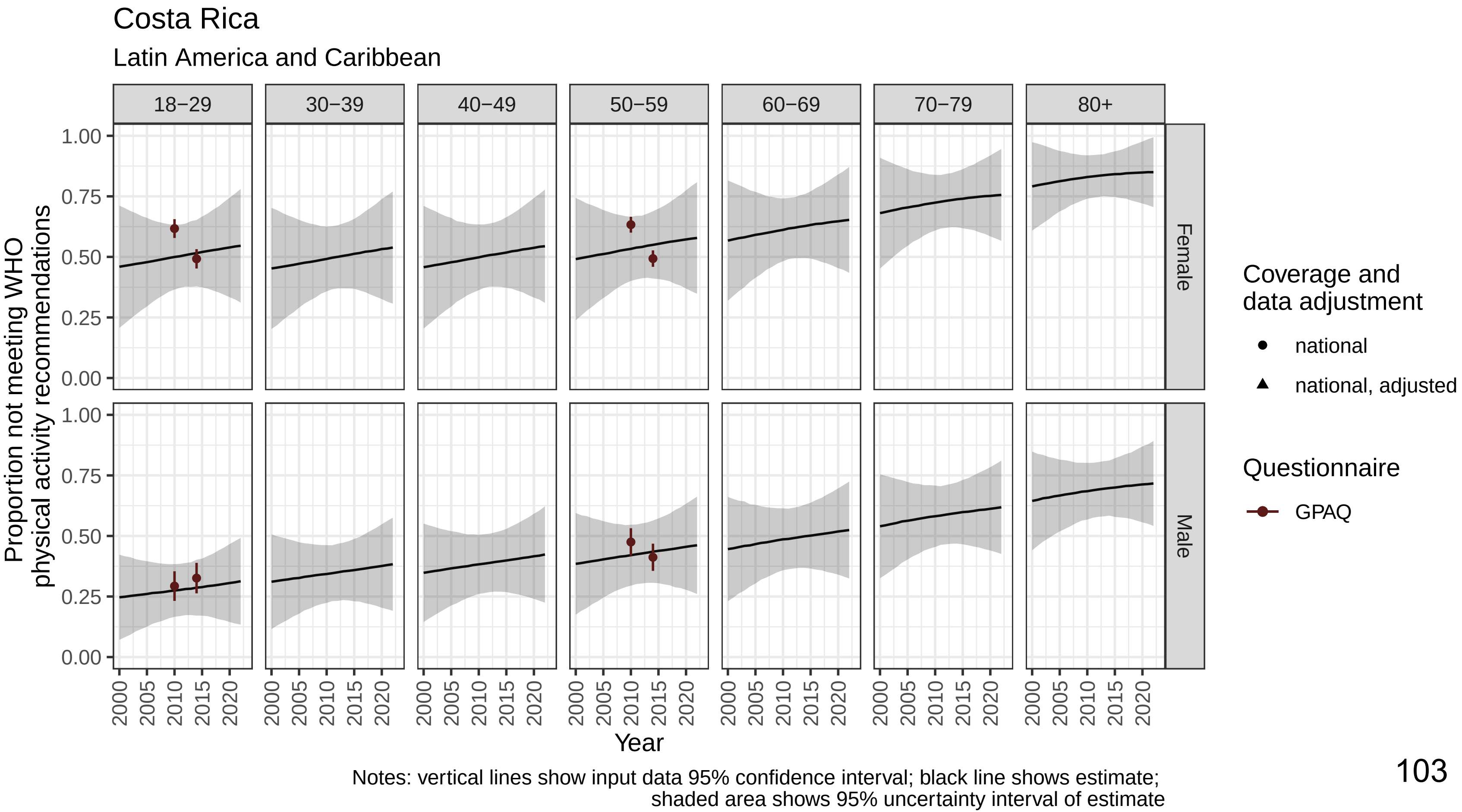
Costa Rica
Latin America and Caribbean
18−29
30−39
40−49
50−59
60−69
70−79
80+
Female
Male
Proportion not meeting WHO physical activity recommendations
1.00
0.75
0.50
0.25
0.00
2000
2005
2010
2015
2020
Year
Coverage and data adjustment
national
national, adjusted
Questionnaire
GPAQ
Notes: vertical lines show input data 95% confidence interval; black line shows estimate;
shaded area shows 95% uncertainty interval of estimate

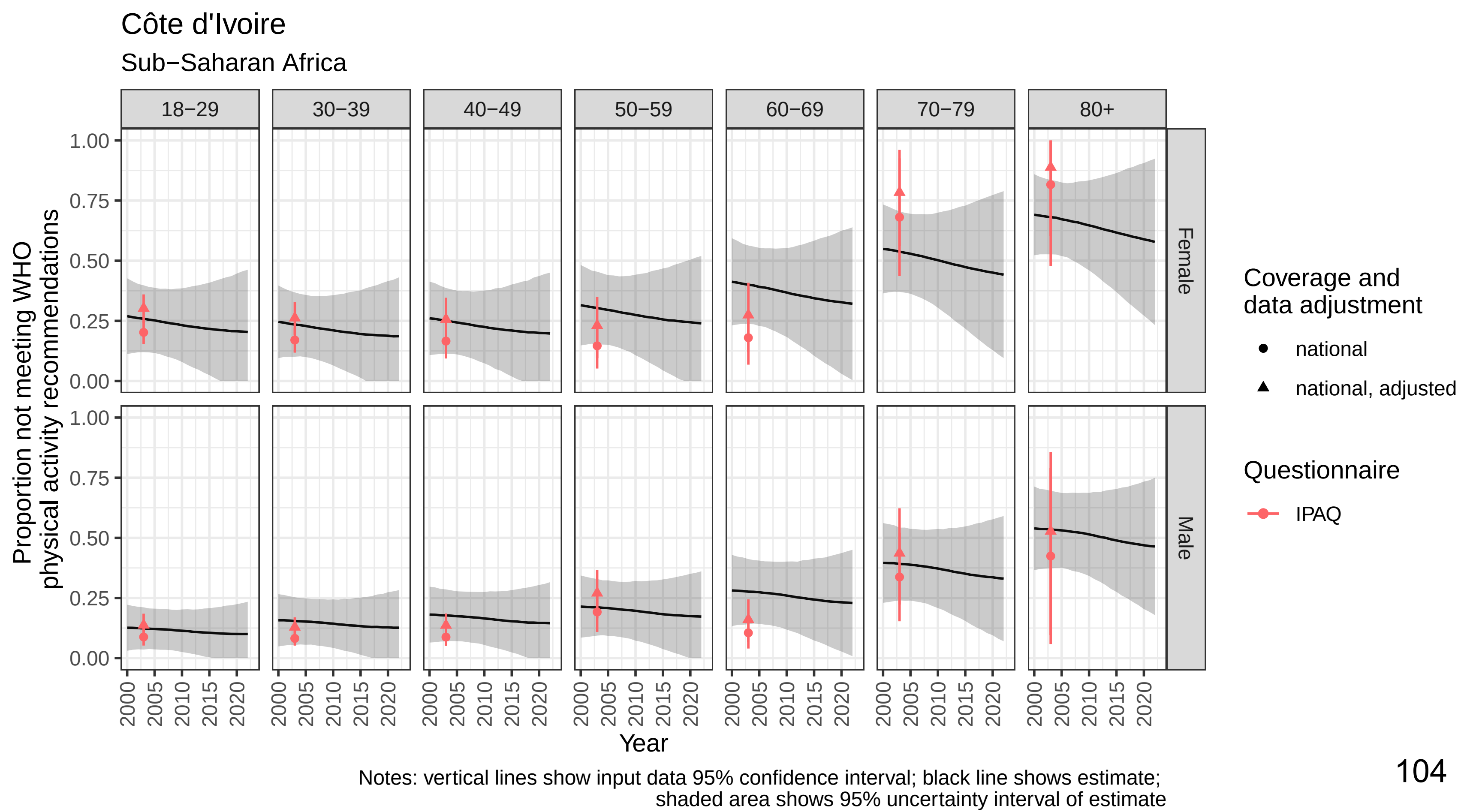
Côte d'Ivoire
Sub−Saharan Africa
18−29
30−39
40−49
50−59
60−69
70−79
80+
Female
Male
Proportion not meeting WHO physical activity recommendations
Year
Coverage and data adjustment
national
national, adjusted
Questionnaire
IPAQ
Notes: vertical lines show input data 95% confidence interval; black line shows estimate; shaded area shows 95% uncertainty interval of estimate

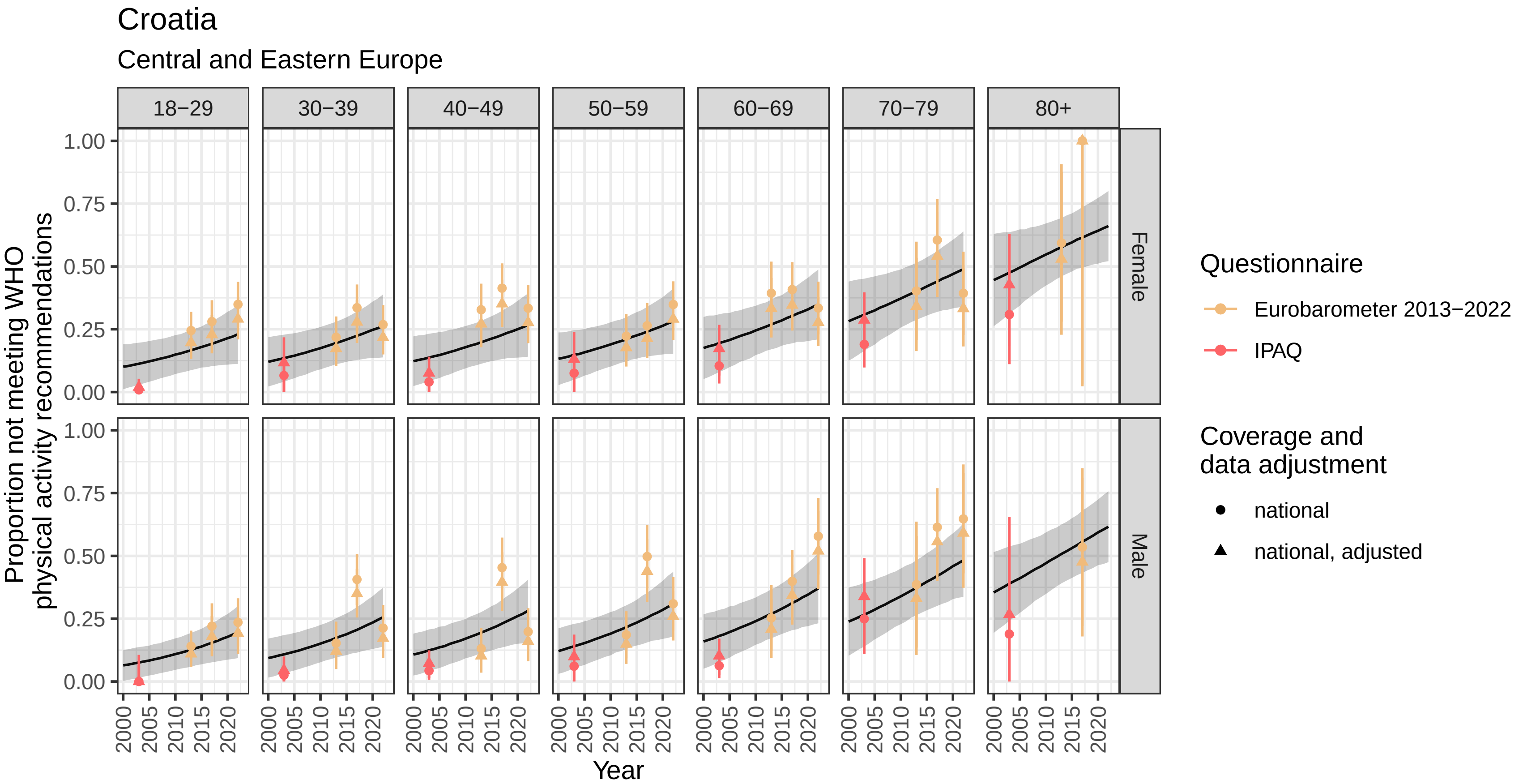

Notes: vertical lines show input data 95% confidence interval; black line shows estimate; shaded area shows 95% uncertainty interval of estimate

Cuba

Latin America and Caribbean

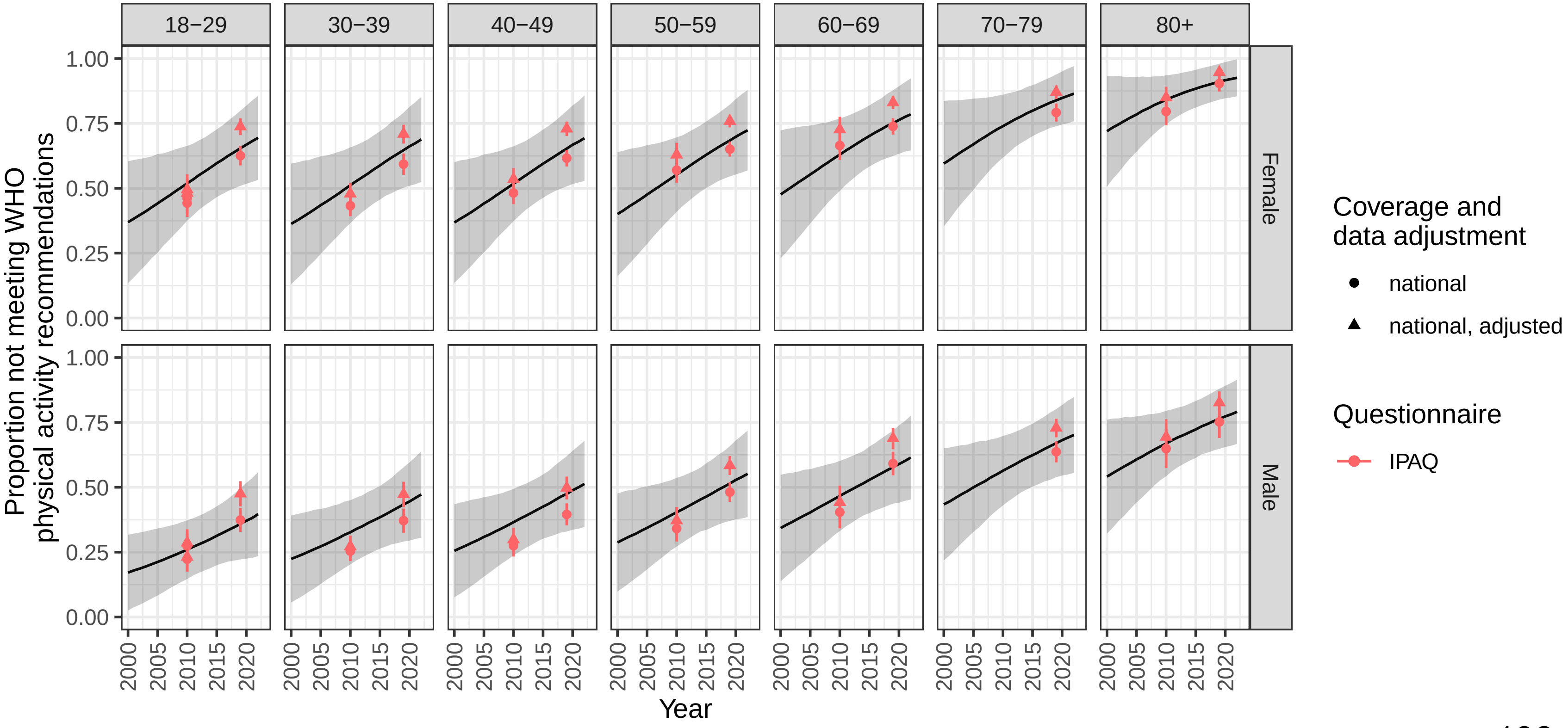

Notes: vertical lines show input data 95% confidence interval; black line shows estimate; shaded area shows 95% uncertainty interval of estimate

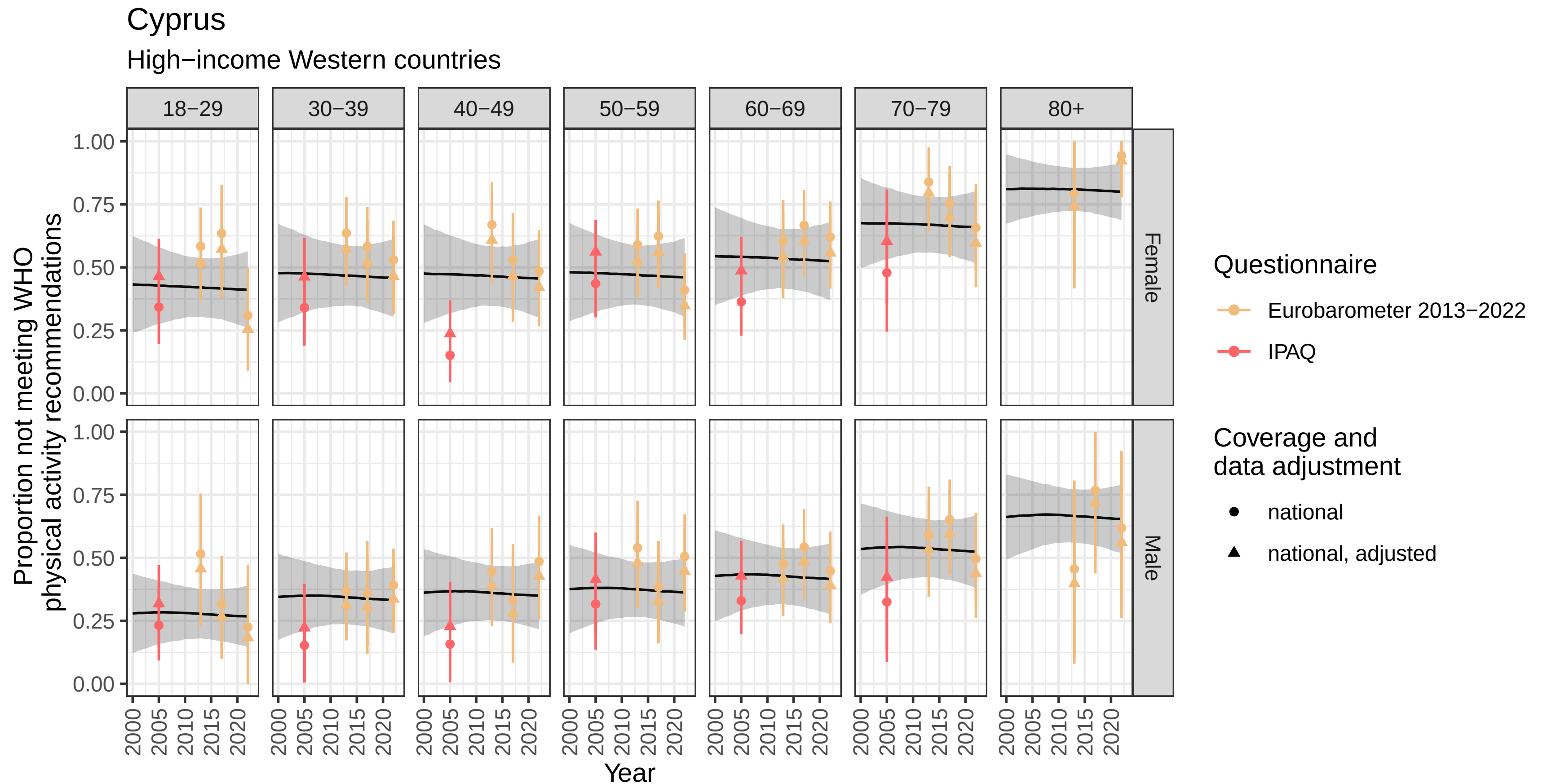

Notes: vertical lines show input data 95% confidence interval; black line shows estimate; shaded area shows 95% uncertainty interval of estimate

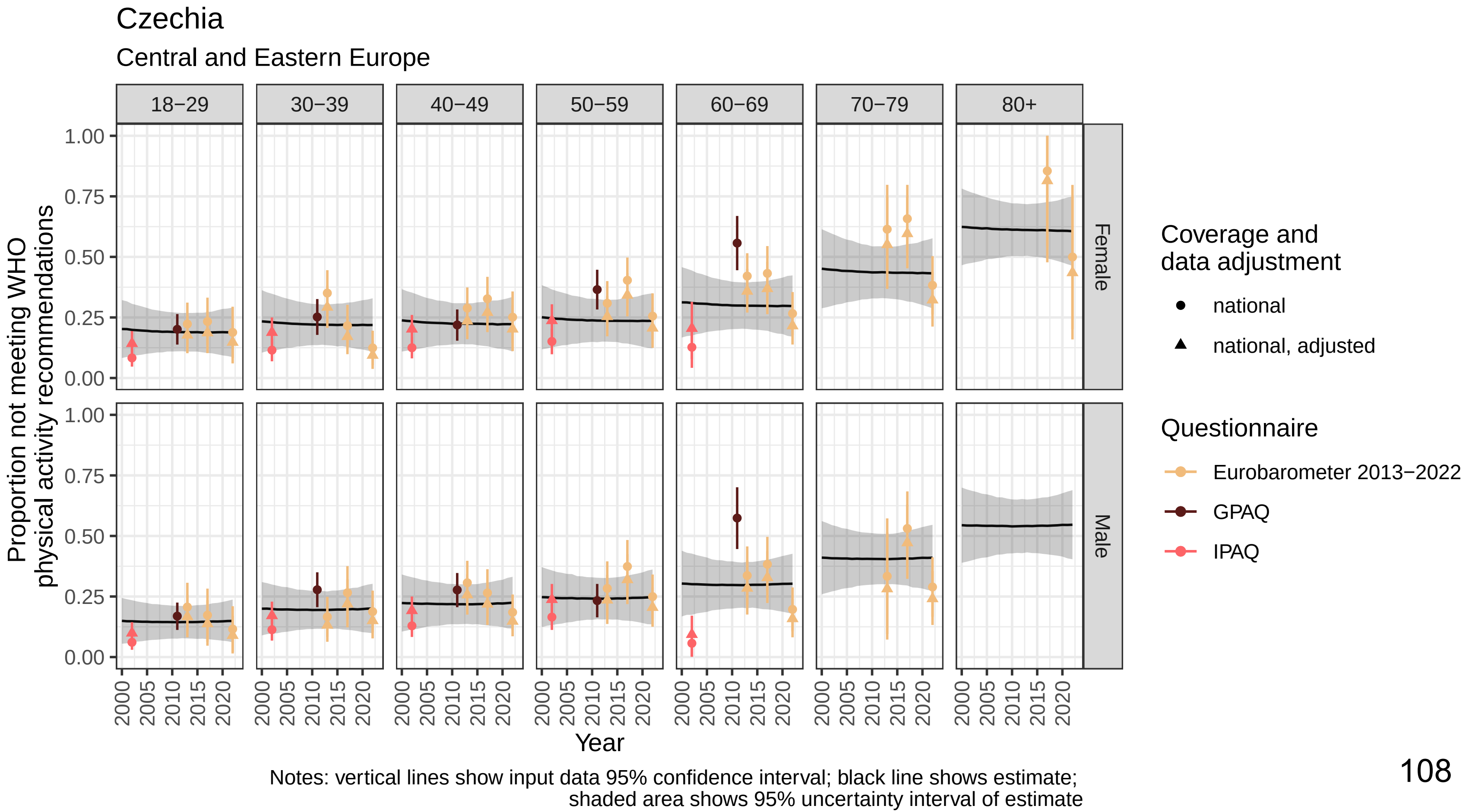

Czechia
Central and Eastern Europe
18−29
30−39
40−49
50−59
60−69
70−79
80+
Female
Male
1.00
0.75
0.50
0.25
0.00
Proportion not meeting WHO physical activity recommendations
2000
2005
2010
2015
2020
Year
Coverage and data adjustment
national
national, adjusted
Questionnaire
Eurobarometer 2013−2022
GPAQ
IPAQ
Notes: vertical lines show input data 95% confidence interval; black line shows estimate; shaded area shows 95% uncertainty interval of estimate

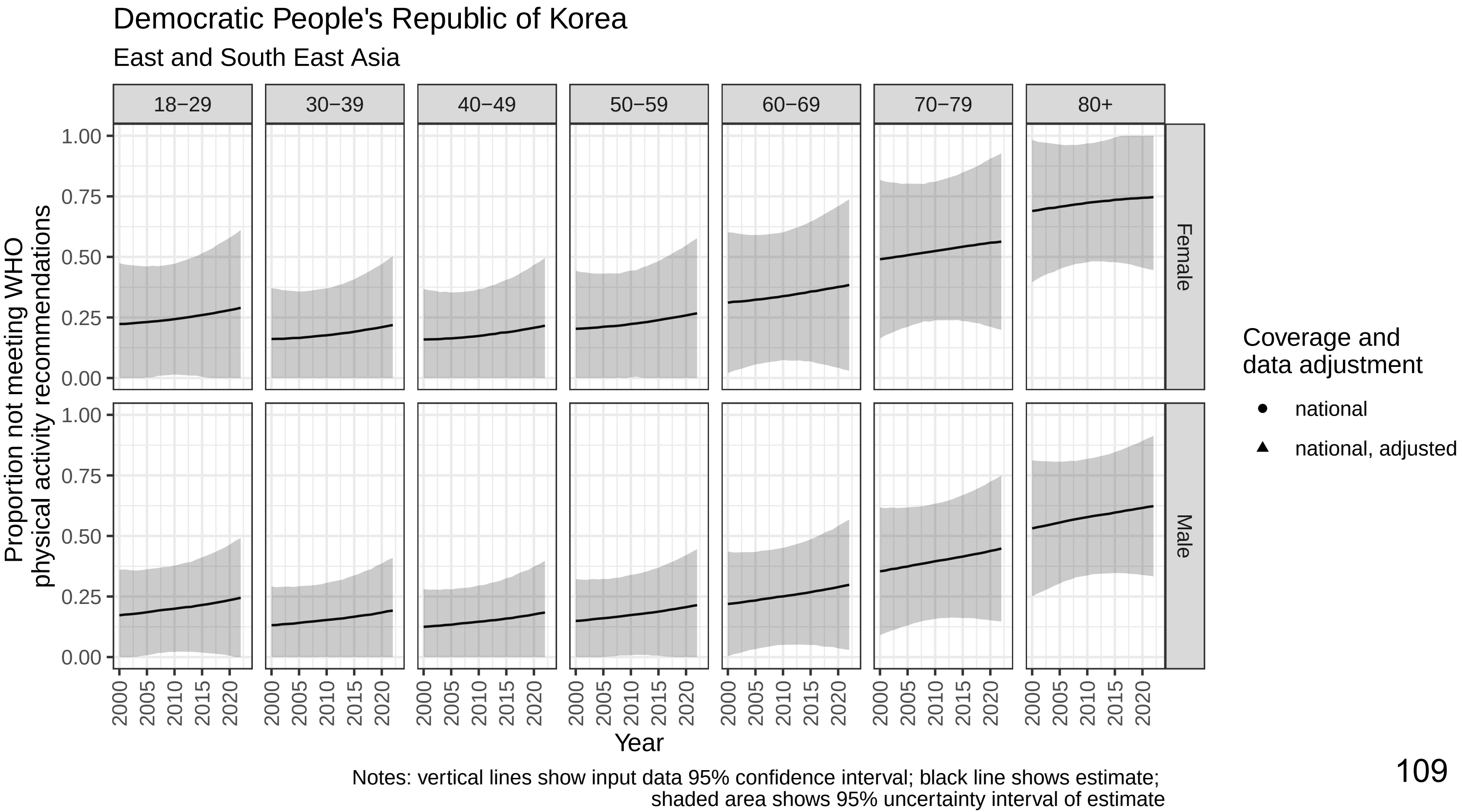
Democratic People's Republic of Korea
East and South East Asia
18−29
30−39
40−49
50−59
60−69
70−79
80+
Female
Male
Proportion not meeting WHO physical activity recommendations
Year
Coverage and data adjustment
national
national, adjusted
Notes: vertical lines show input data 95% confidence interval; black line shows estimate; shaded area shows 95% uncertainty interval of estimate

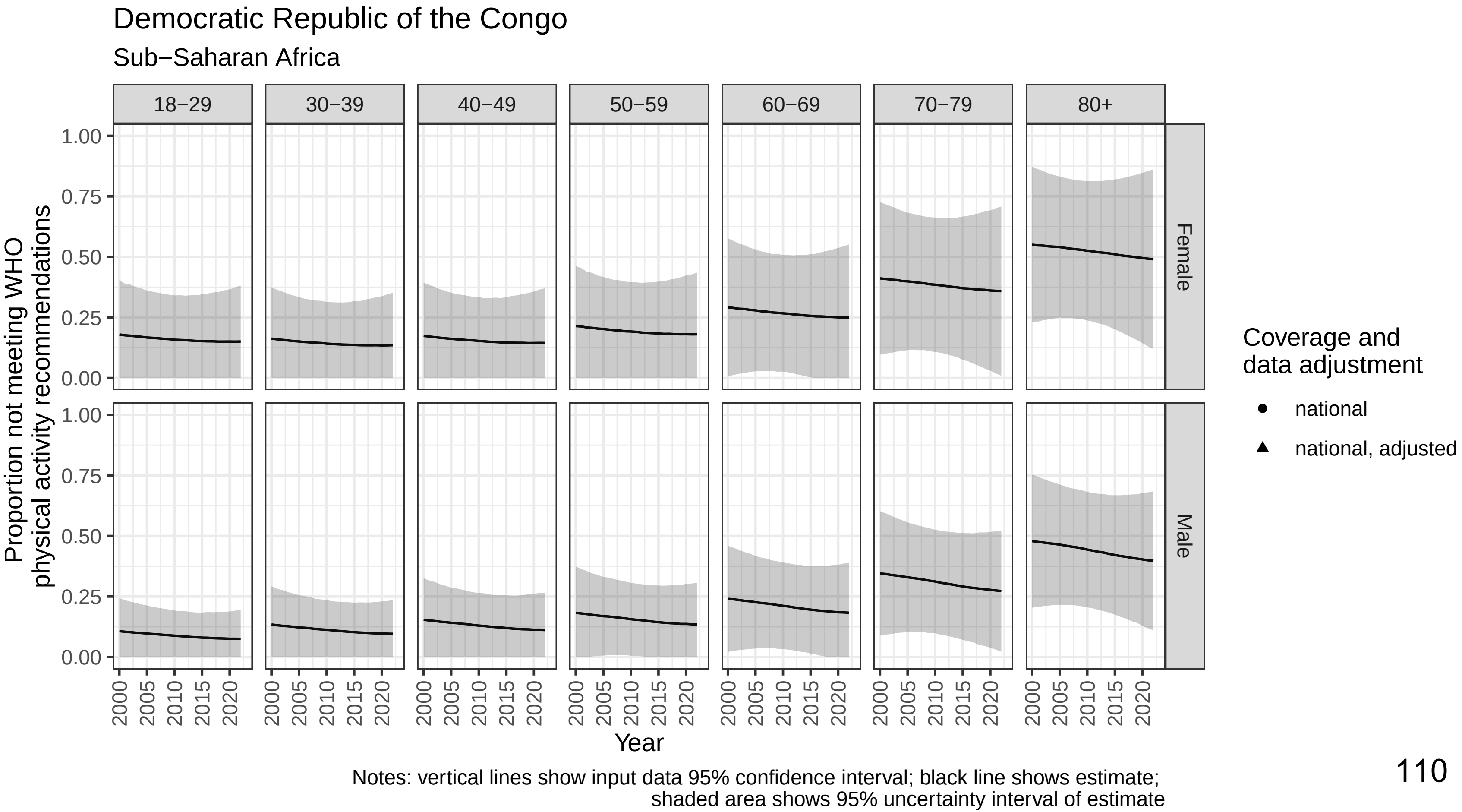
Democratic Republic of the Congo
Sub−Saharan Africa
18−29
30−39
40−49
50−59
60−69
70−79
80+
Female
Male
1.00
0.75
0.50
0.25
0.00
2000
2005
2010
2015
2020
Proportion not meeting WHO
physical activity recommendations
Year
Coverage and
data adjustment
national
national, adjusted
Notes: vertical lines show input data 95% confidence interval; black line shows estimate;
shaded area shows 95% uncertainty interval of estimate

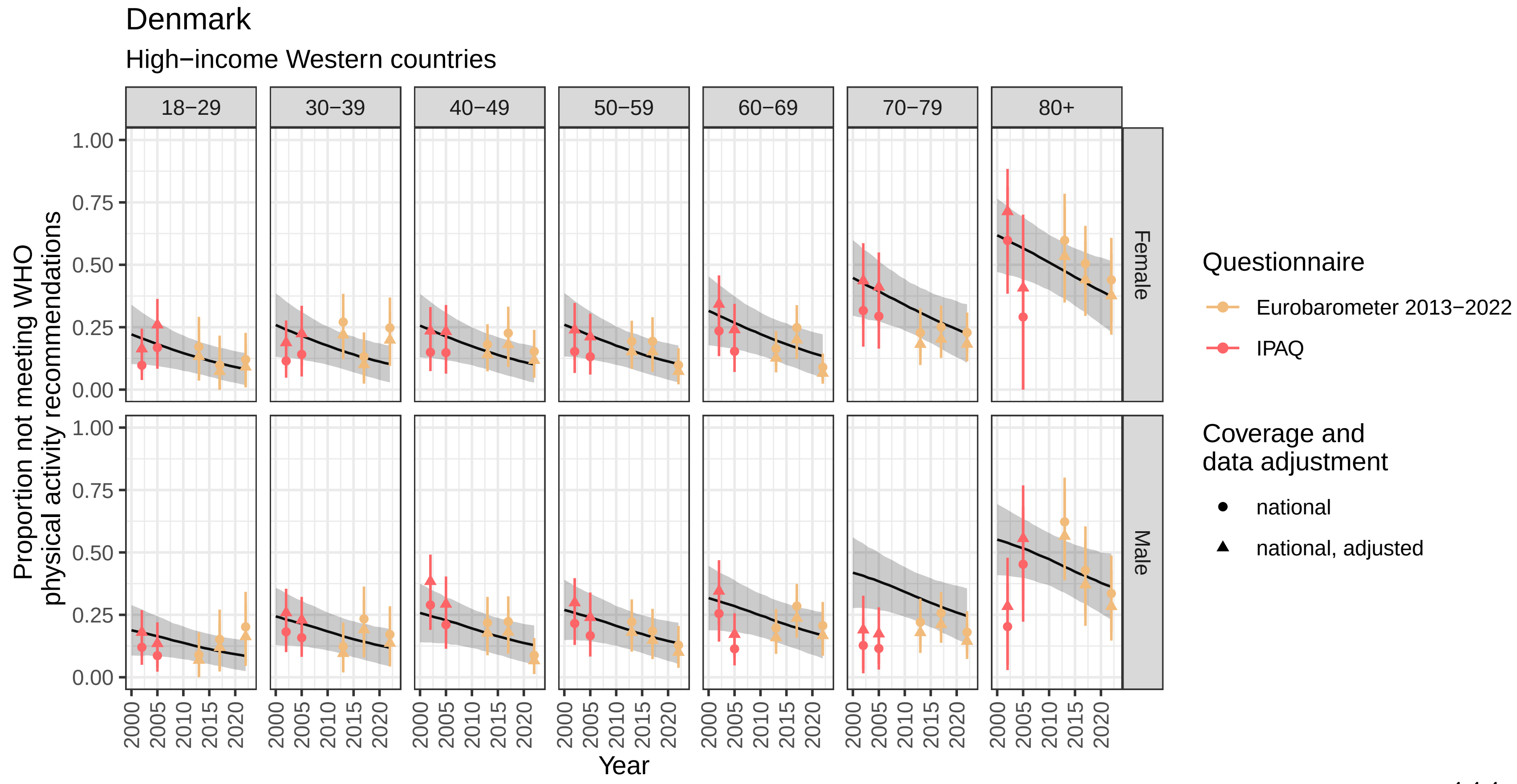

Notes: vertical lines show input data 95% confidence interval; black line shows estimate; shaded area shows 95% uncertainty interval of estimate

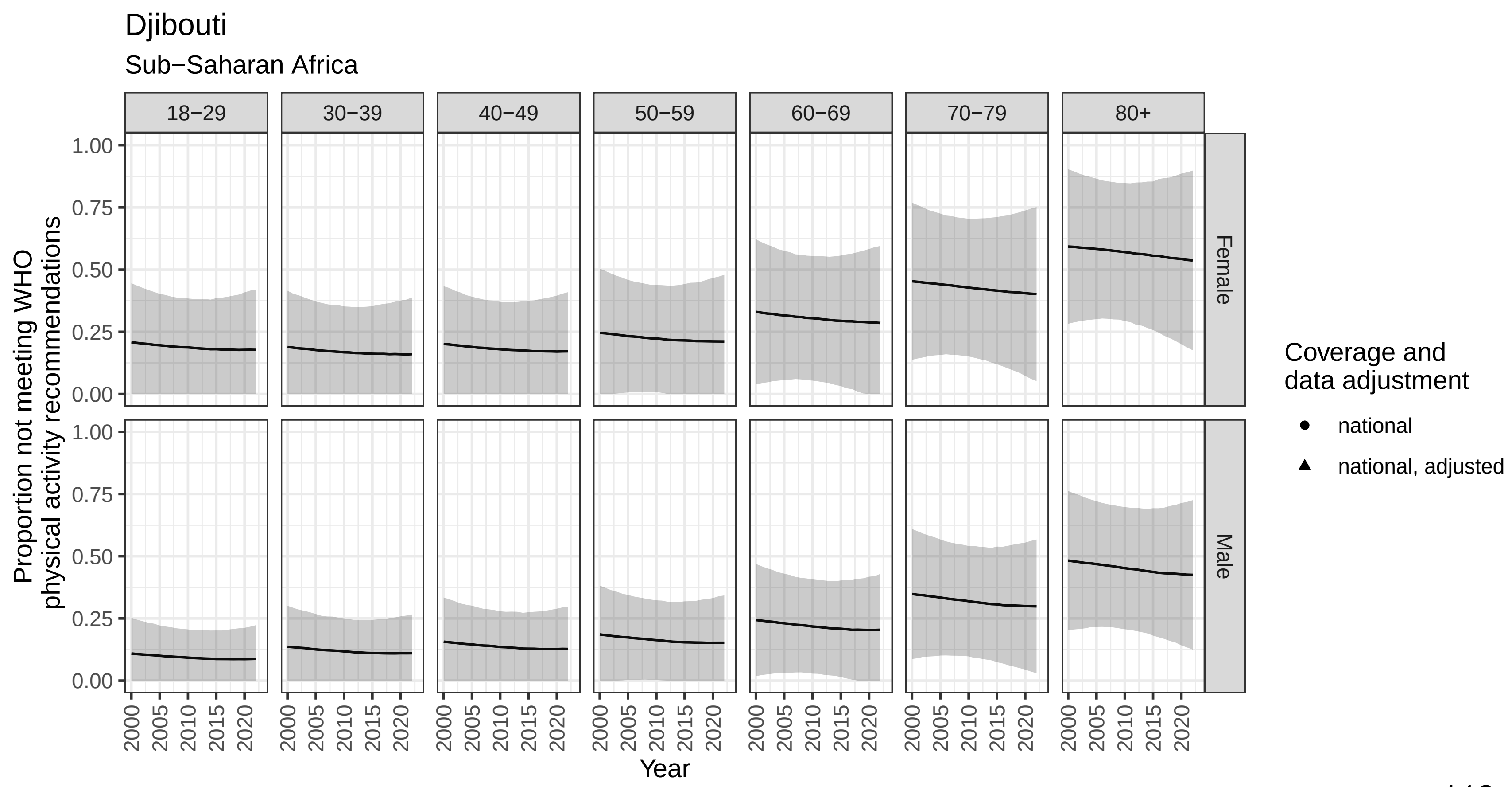

Notes: vertical lines show input data 95% confidence interval; black line shows estimate; shaded area shows 95% uncertainty interval of estimate

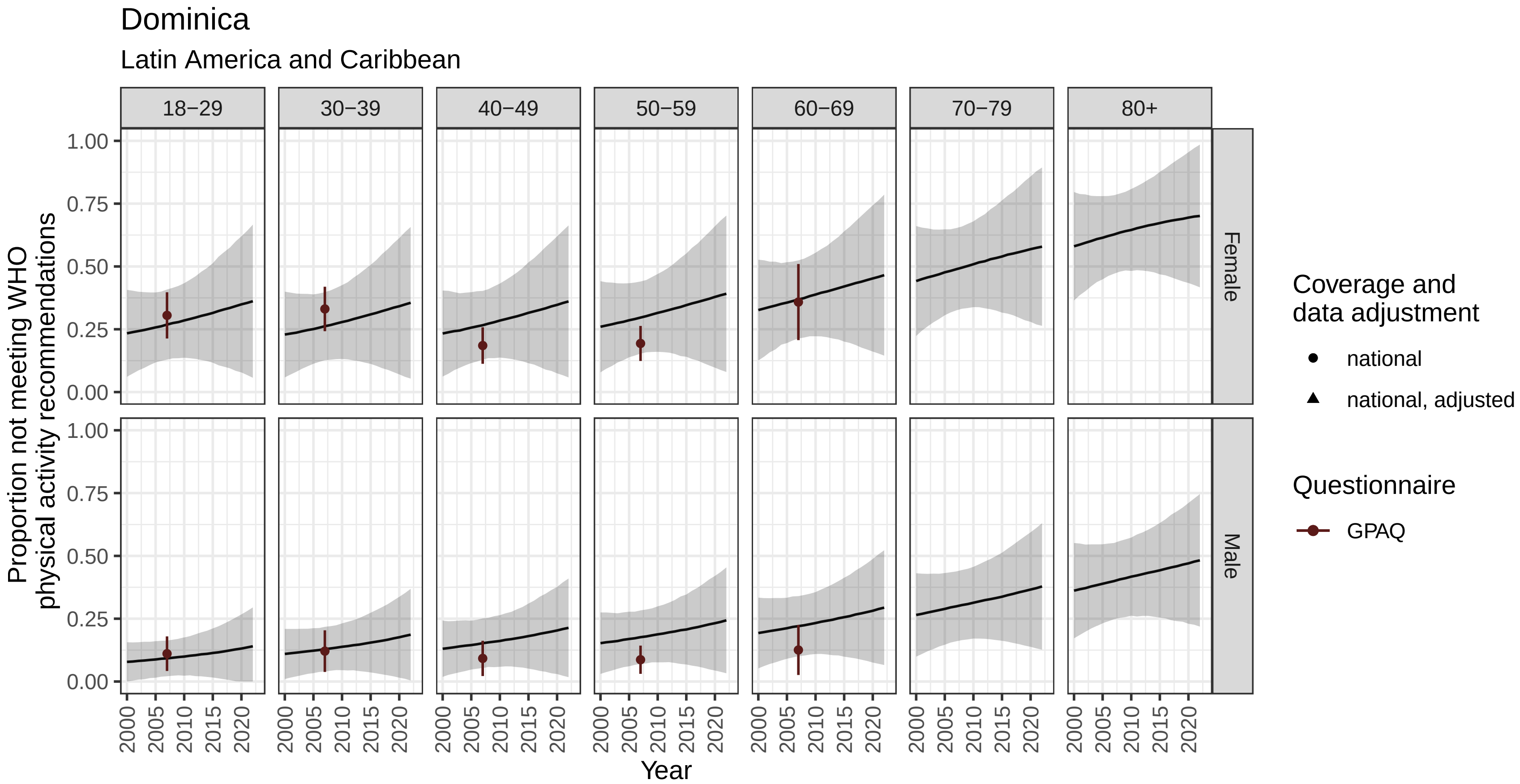

Notes: vertical lines show input data 95% confidence interval; black line shows estimate; shaded area shows 95% uncertainty interval of estimate

Dominican Republic

Latin America and Caribbean

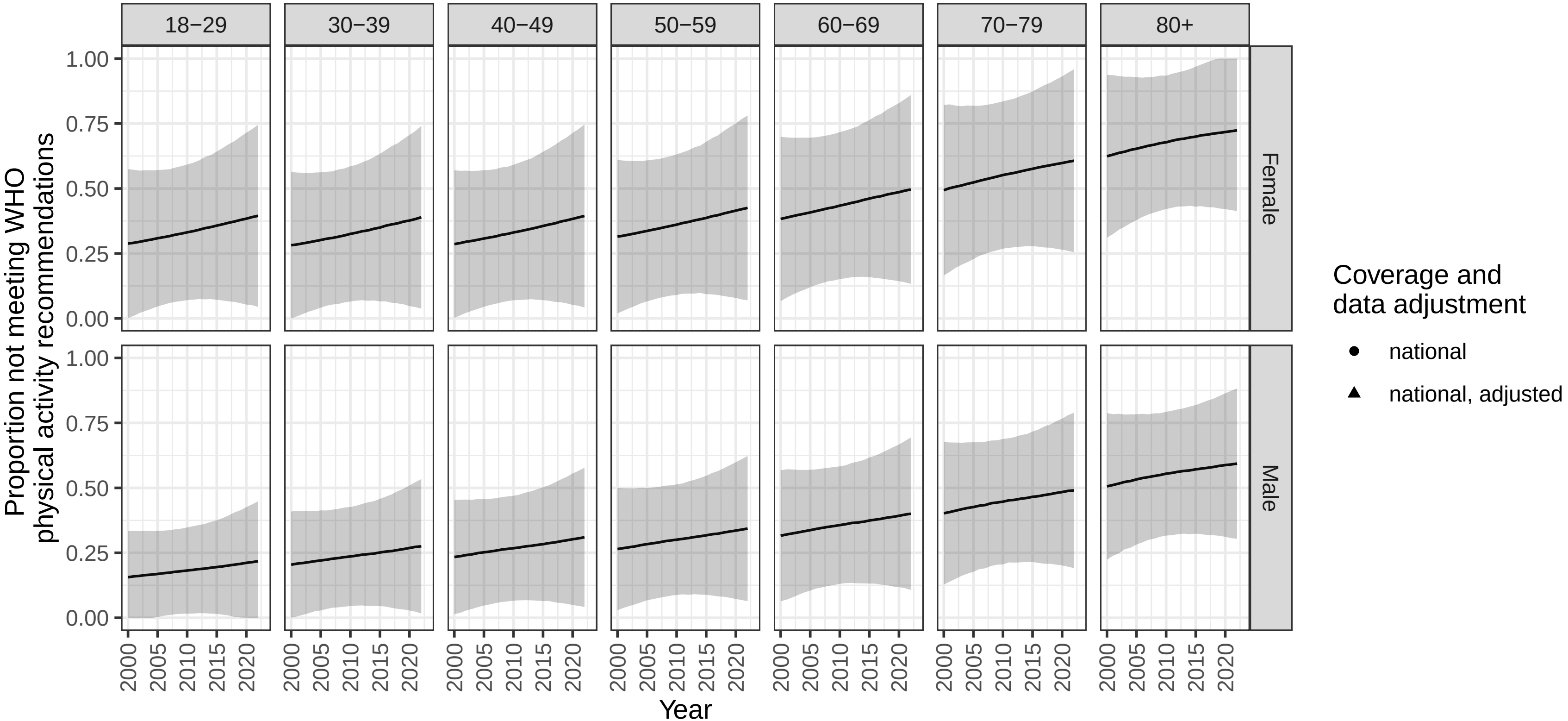

Notes: vertical lines show input data 95% confidence interval; black line shows estimate; shaded area shows 95% uncertainty interval of estimate

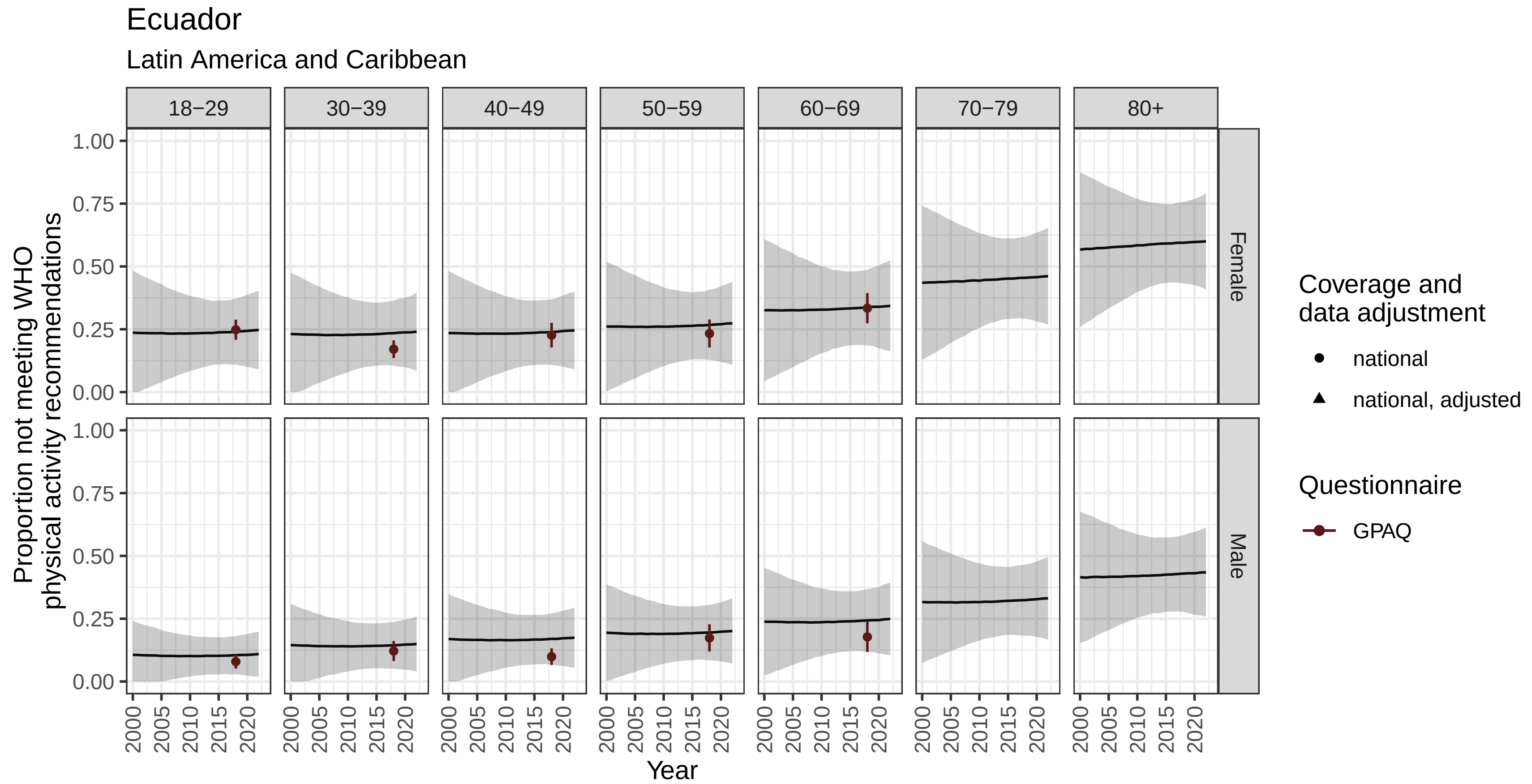

Notes: vertical lines show input data 95% confidence interval; black line shows estimate; shaded area shows 95% uncertainty interval of estimate

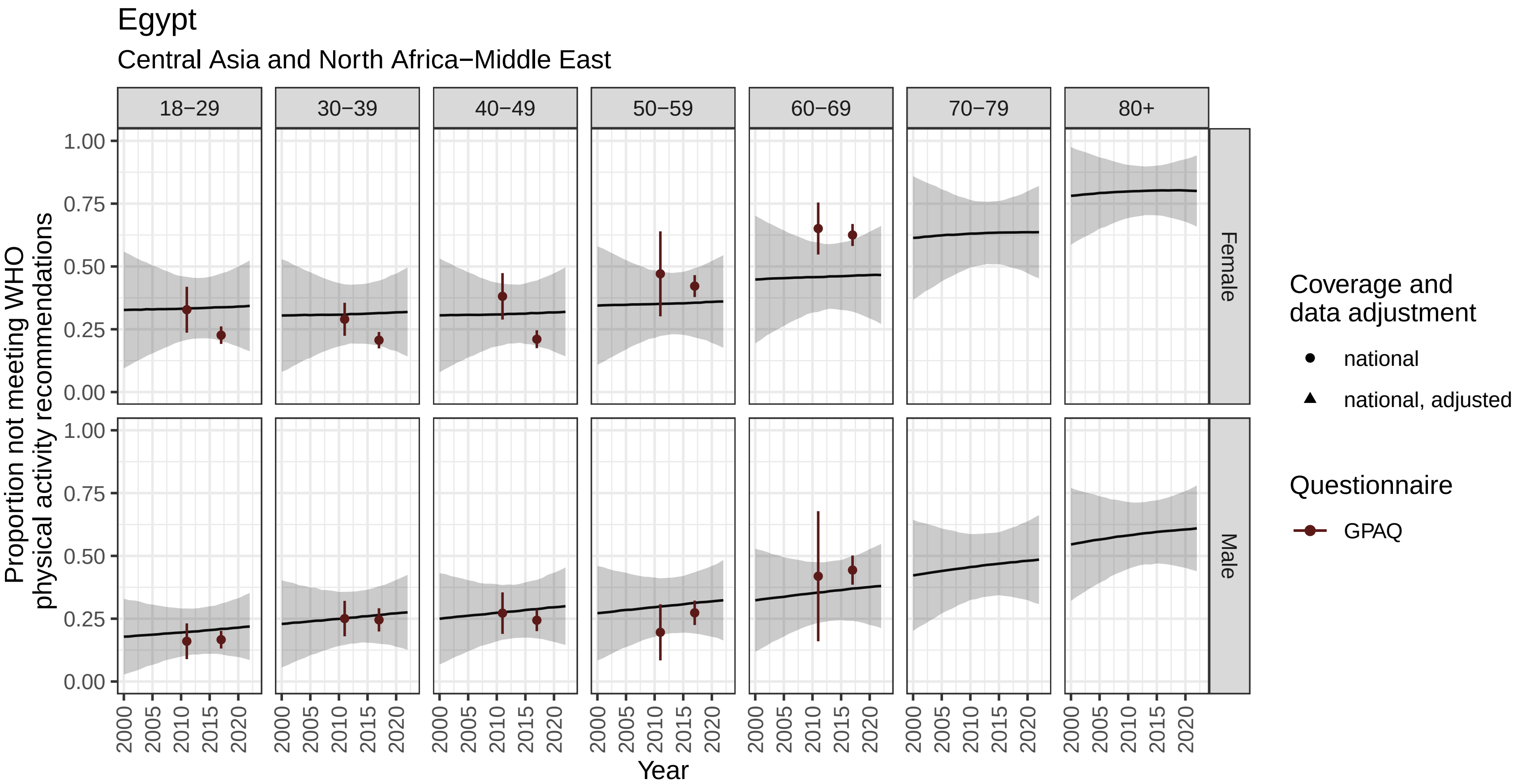

Notes: vertical lines show input data 95% confidence interval; black line shows estimate; shaded area shows 95% uncertainty interval of estimate

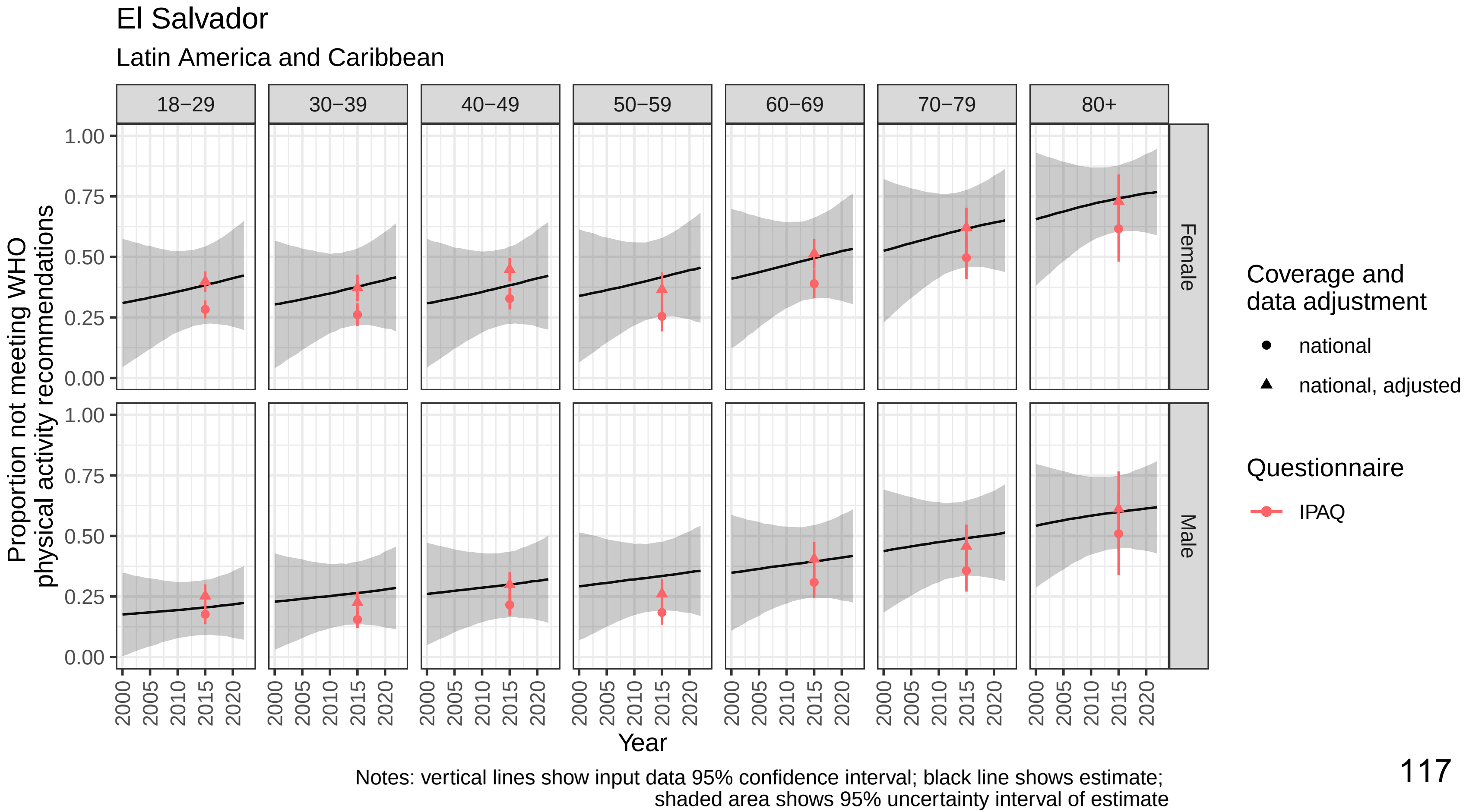
El Salvador
Latin America and Caribbean
18−29
30−39
40−49
50−59
60−69
70−79
80+
Female
Male
Proportion not meeting WHO physical activity recommendations
1.00
0.75
0.50
0.25
0.00
2000
2005
2010
2015
2020
Year
Coverage and data adjustment
national
national, adjusted
Questionnaire
IPAQ
Notes: vertical lines show input data 95% confidence interval; black line shows estimate; shaded area shows 95% uncertainty interval of estimate

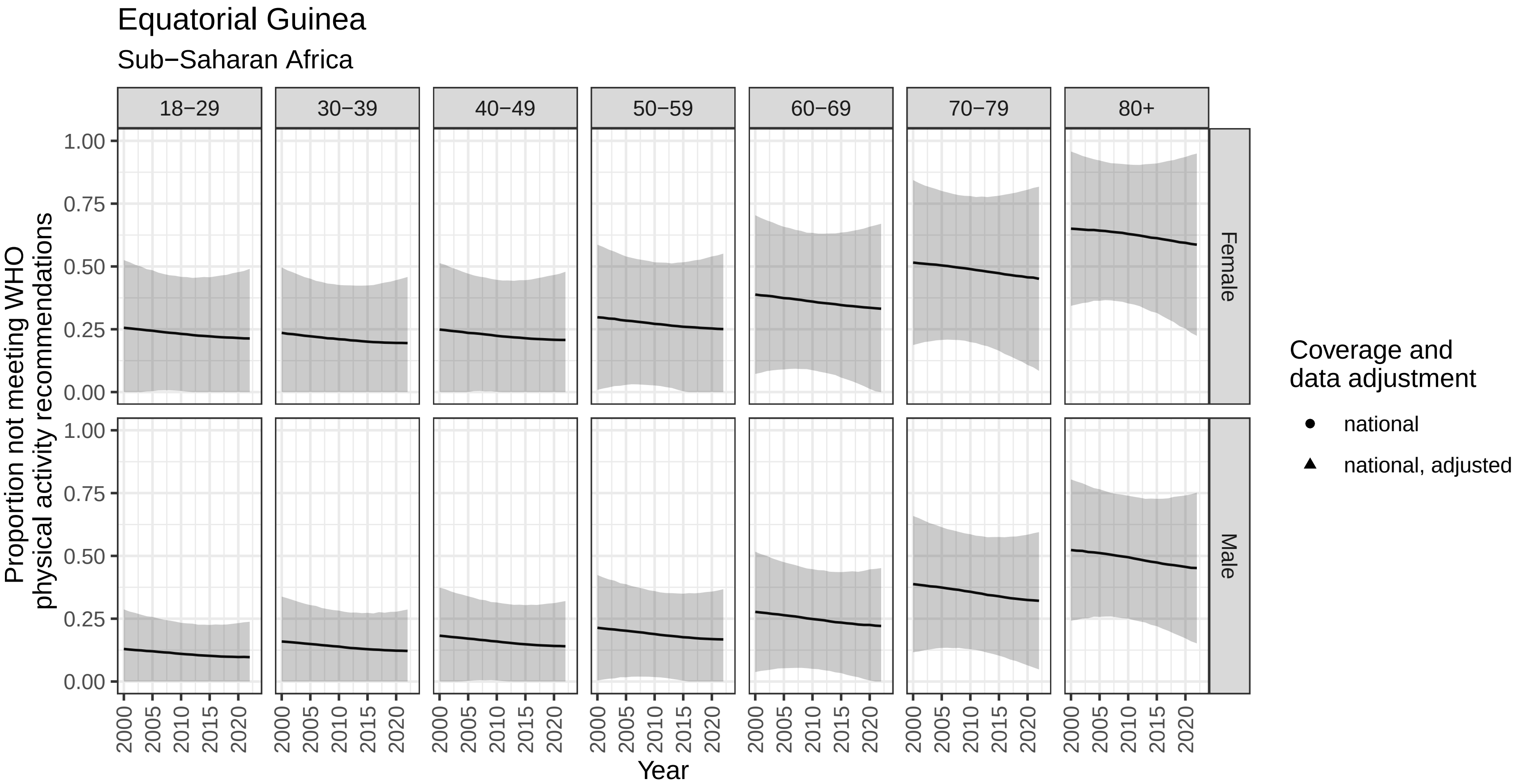

Notes: vertical lines show input data 95% confidence interval; black line shows estimate; shaded area shows 95% uncertainty interval of estimate

Eritrea

Sub−Saharan Africa

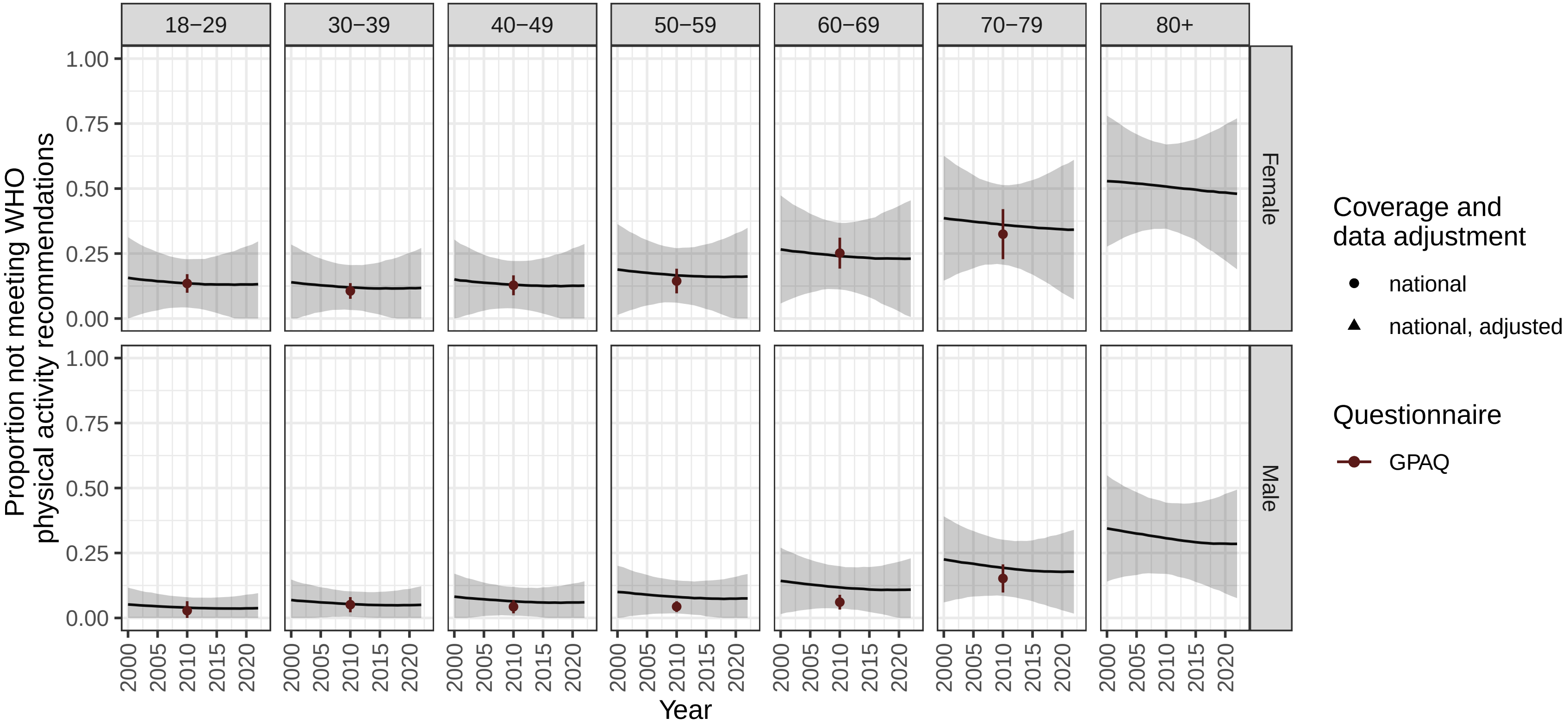

Notes: vertical lines show input data 95% confidence interval; black line shows estimate; shaded area shows 95% uncertainty interval of estimate

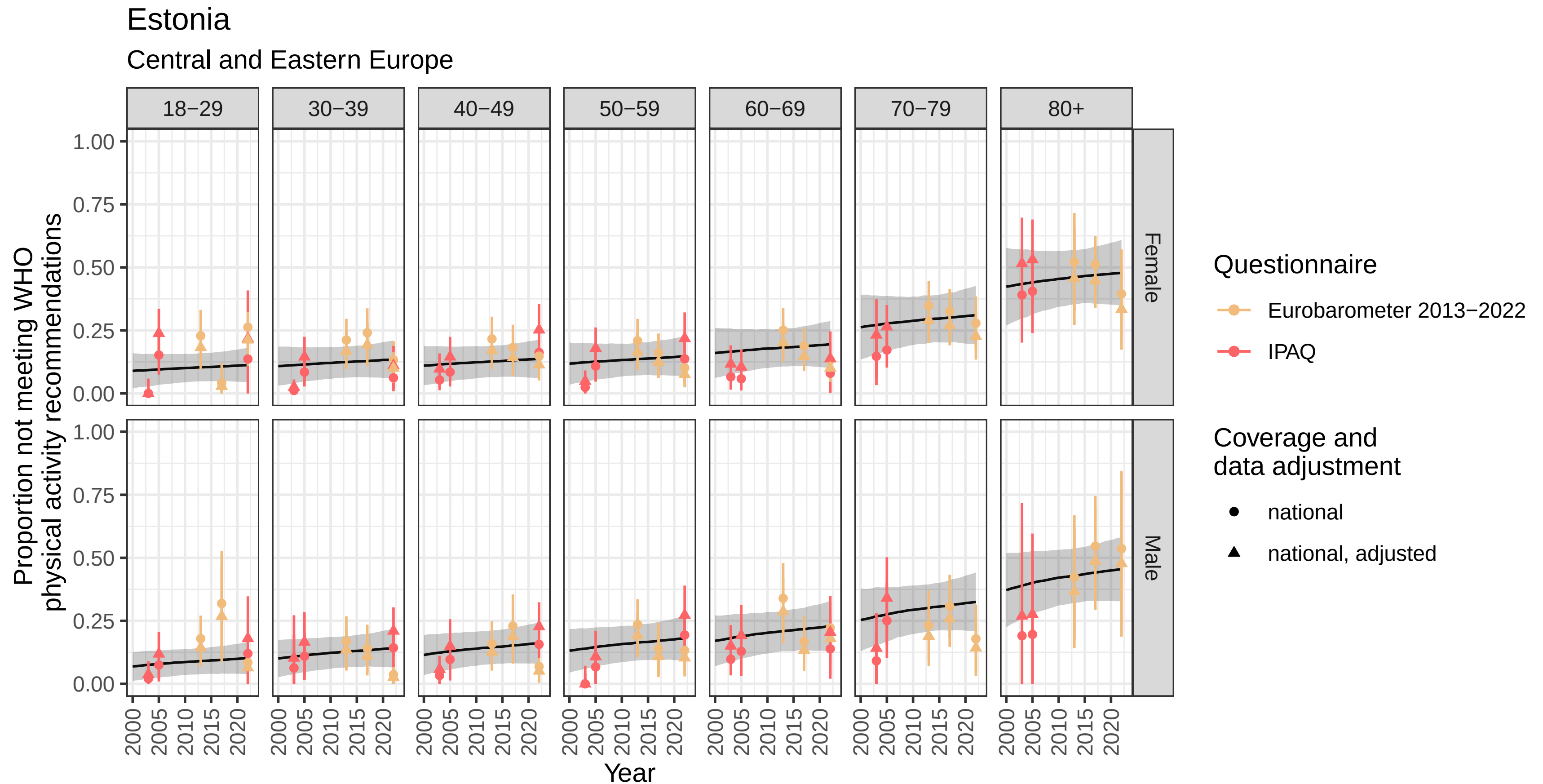

Notes: vertical lines show input data 95% confidence interval; black line shows estimate; shaded area shows 95% uncertainty interval of estimate

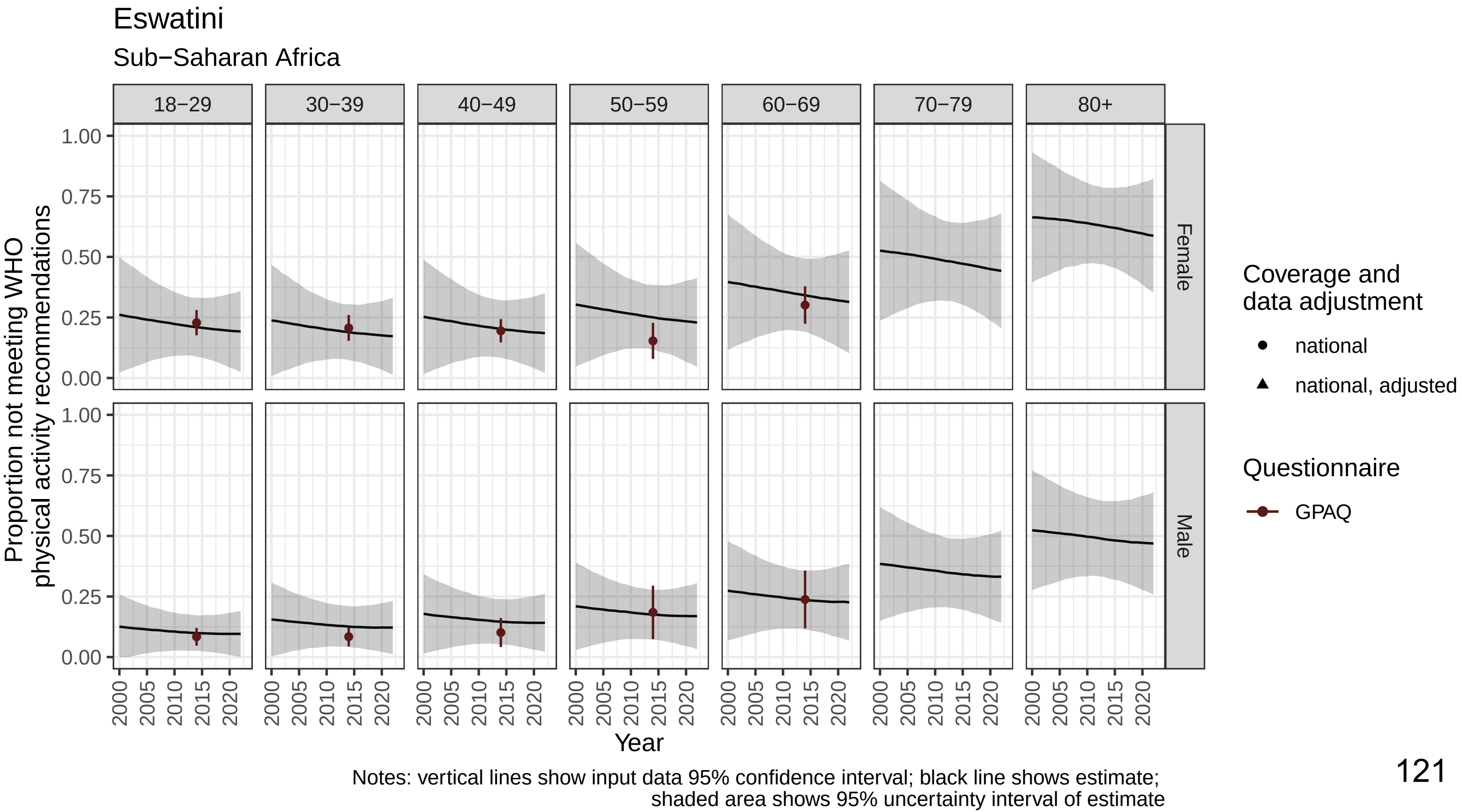

Eswatini
Sub−Saharan Africa
18−29
30−39
40−49
50−59
60−69
70−79
80+
Female
Male
Proportion not meeting WHO physical activity recommendations
1.00
0.75
0.50
0.25
0.00
2000
2005
2010
2015
2020
Year
Coverage and data adjustment
national
national, adjusted
Questionnaire
GPAQ
Notes: vertical lines show input data 95% confidence interval; black line shows estimate; shaded area shows 95% uncertainty interval of estimate

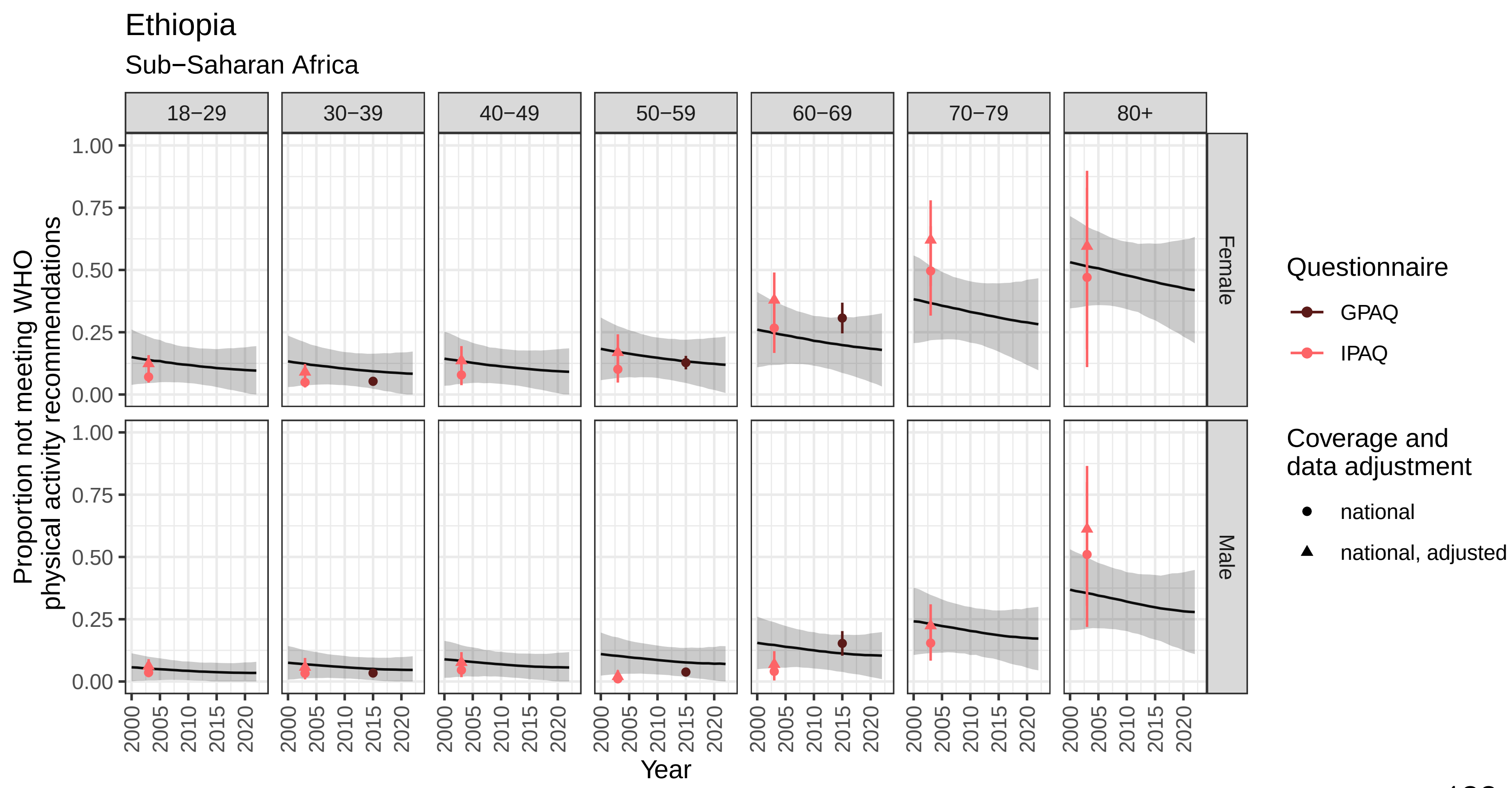

Notes: vertical lines show input data 95% confidence interval; black line shows estimate; shaded area shows 95% uncertainty interval of estimate

Fiji

Oceania

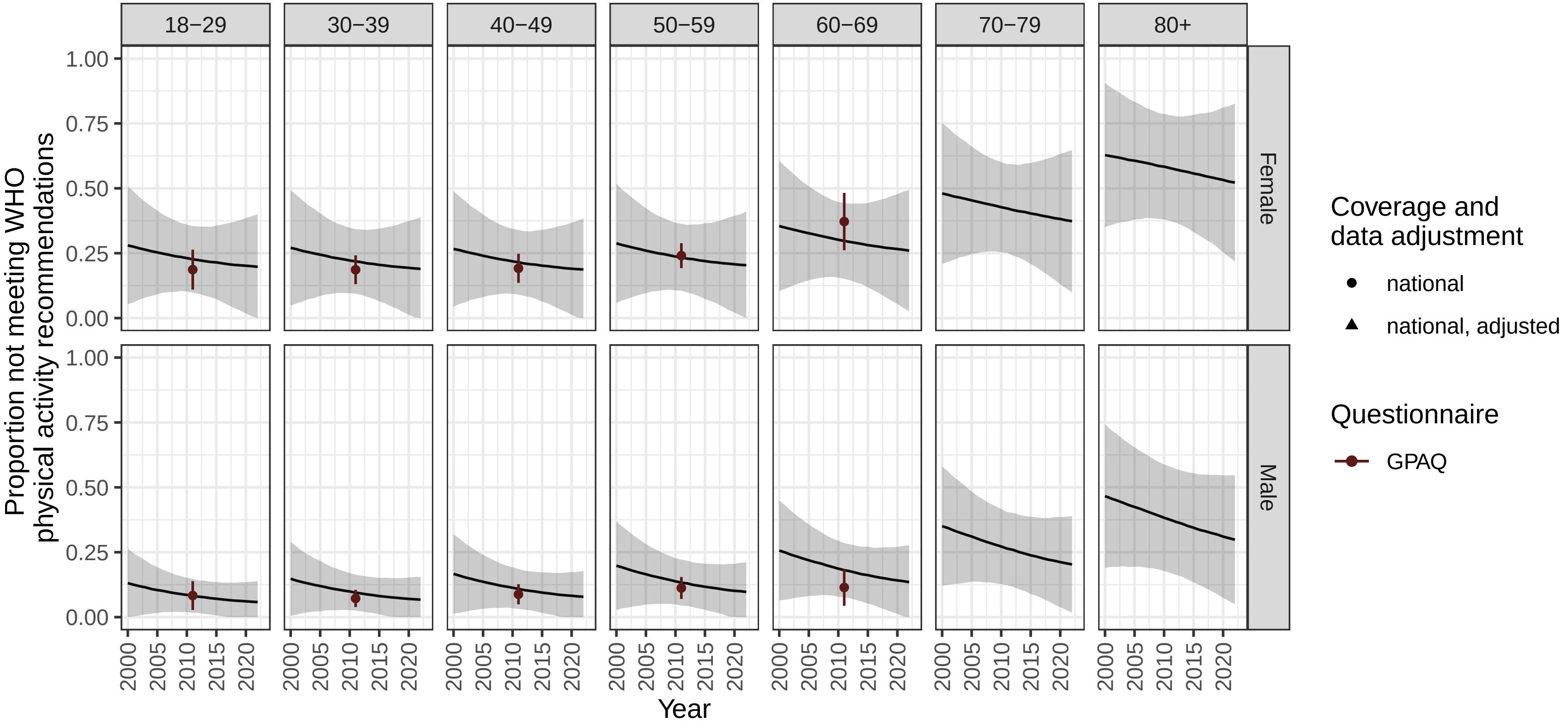

Notes: vertical lines show input data 95% confidence interval; black line shows estimate; shaded area shows 95% uncertainty interval of estimate

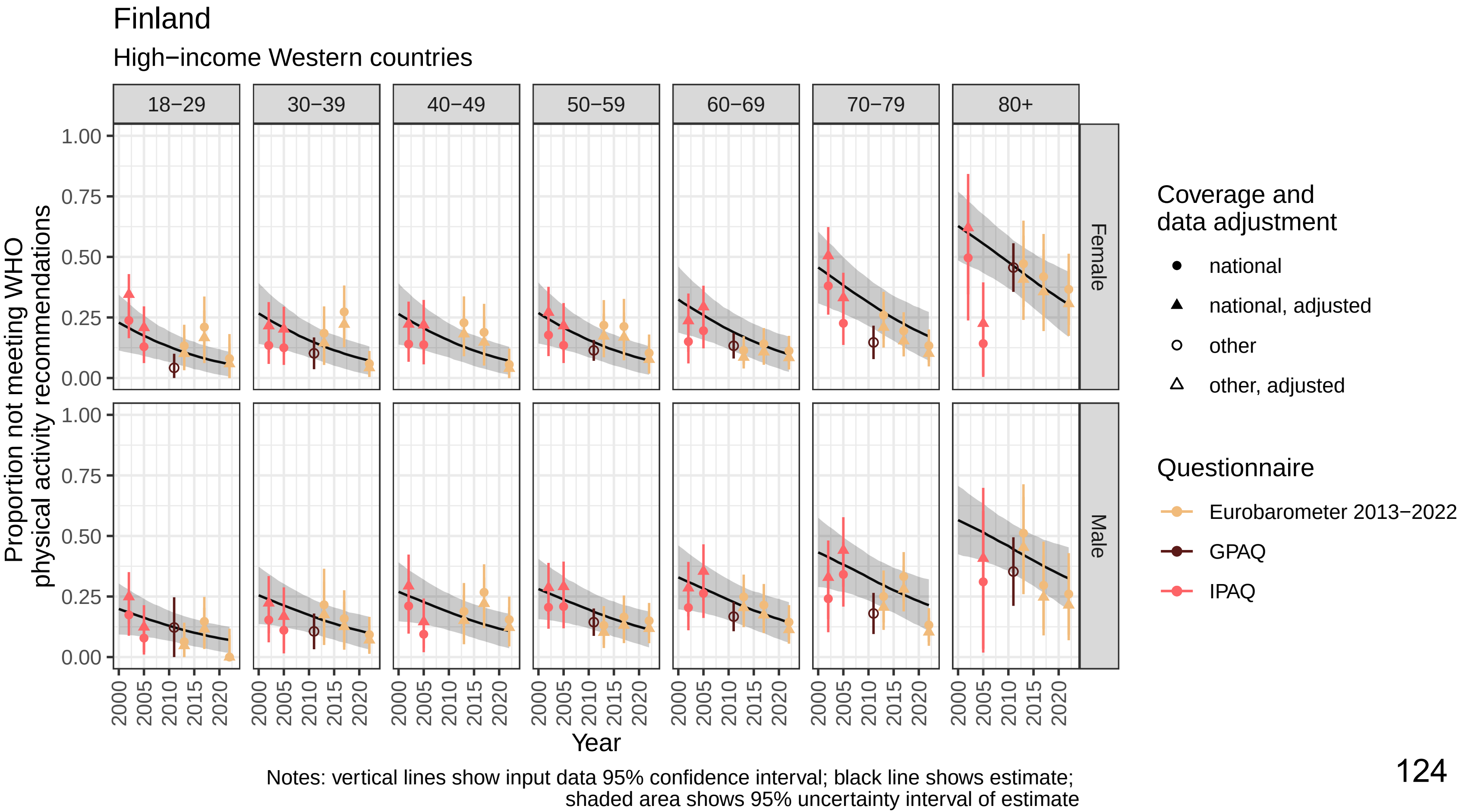

Finland
High−income Western countries
18−29
30−39
40−49
50−59
60−69
70−79
80+
Female
Male
Proportion not meeting WHO physical activity recommendations
Year
Coverage and data adjustment
national
national, adjusted
other
other, adjusted
Questionnaire
Eurobarometer 2013−2022
GPAQ
IPAQ
Notes: vertical lines show input data 95% confidence interval; black line shows estimate; shaded area shows 95% uncertainty interval of estimate

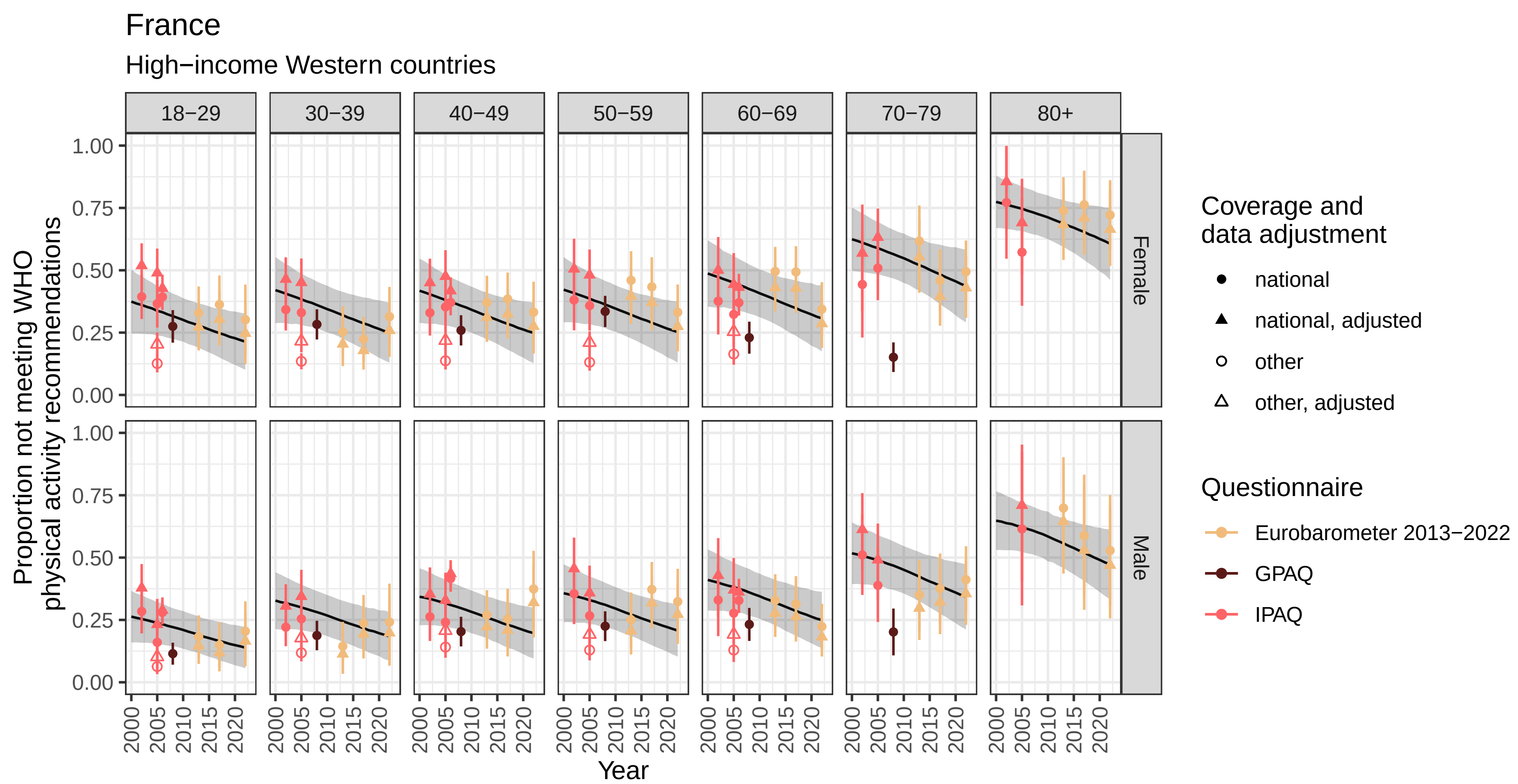

Notes: vertical lines show input data 95% confidence interval; black line shows estimate; shaded area shows 95% uncertainty interval of estimate

Gabon

Sub−Saharan Africa

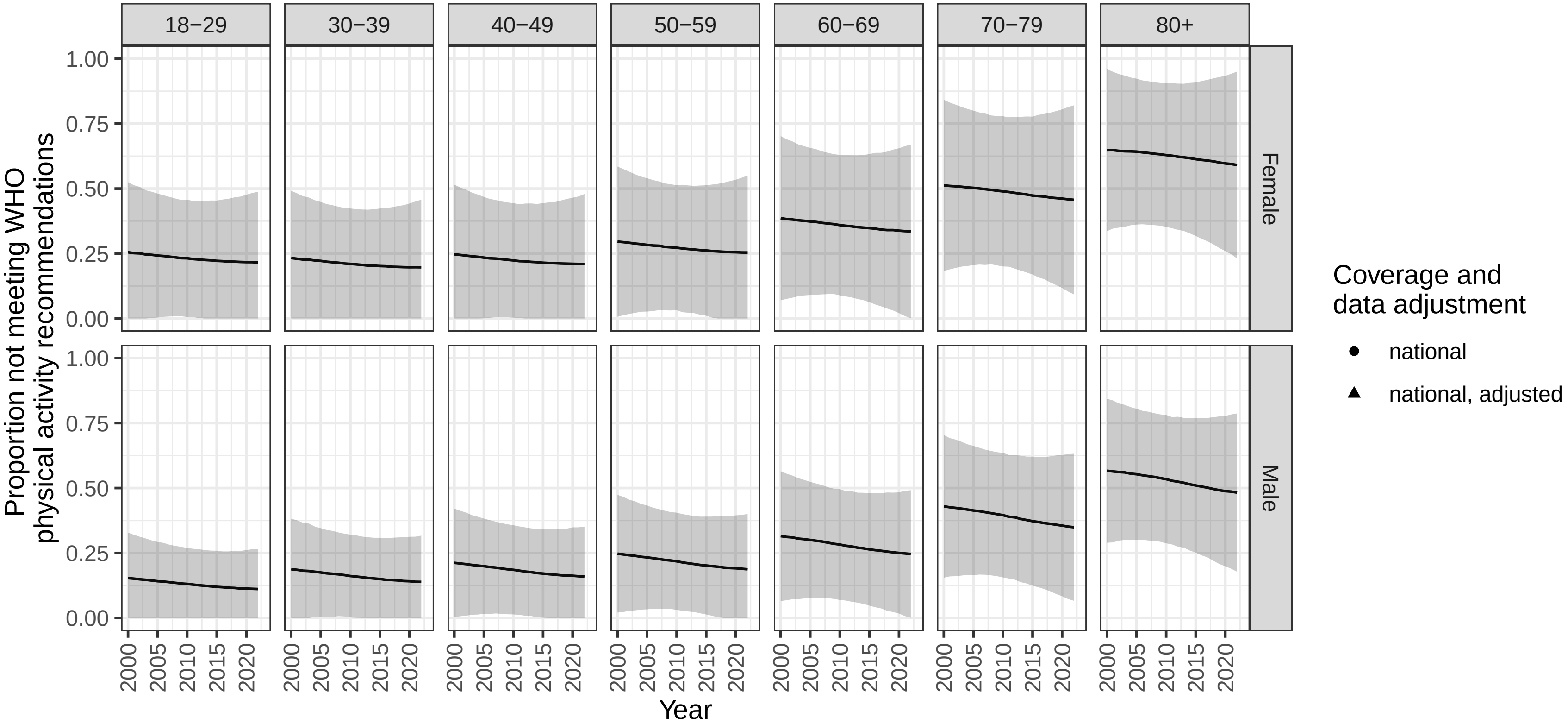

Notes: vertical lines show input data 95% confidence interval; black line shows estimate; shaded area shows 95% uncertainty interval of estimate

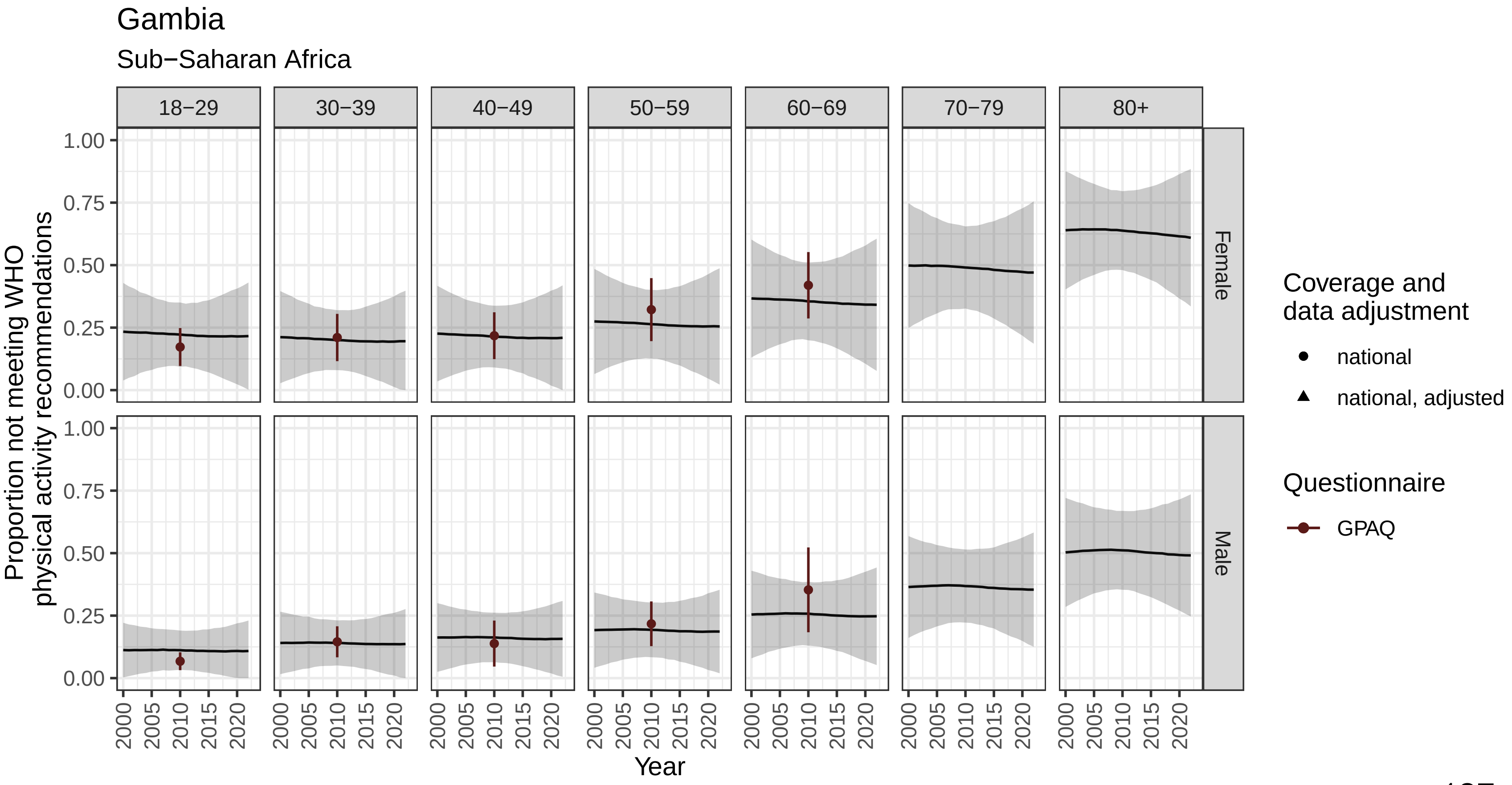

Notes: vertical lines show input data 95% confidence interval; black line shows estimate; shaded area shows 95% uncertainty interval of estimate

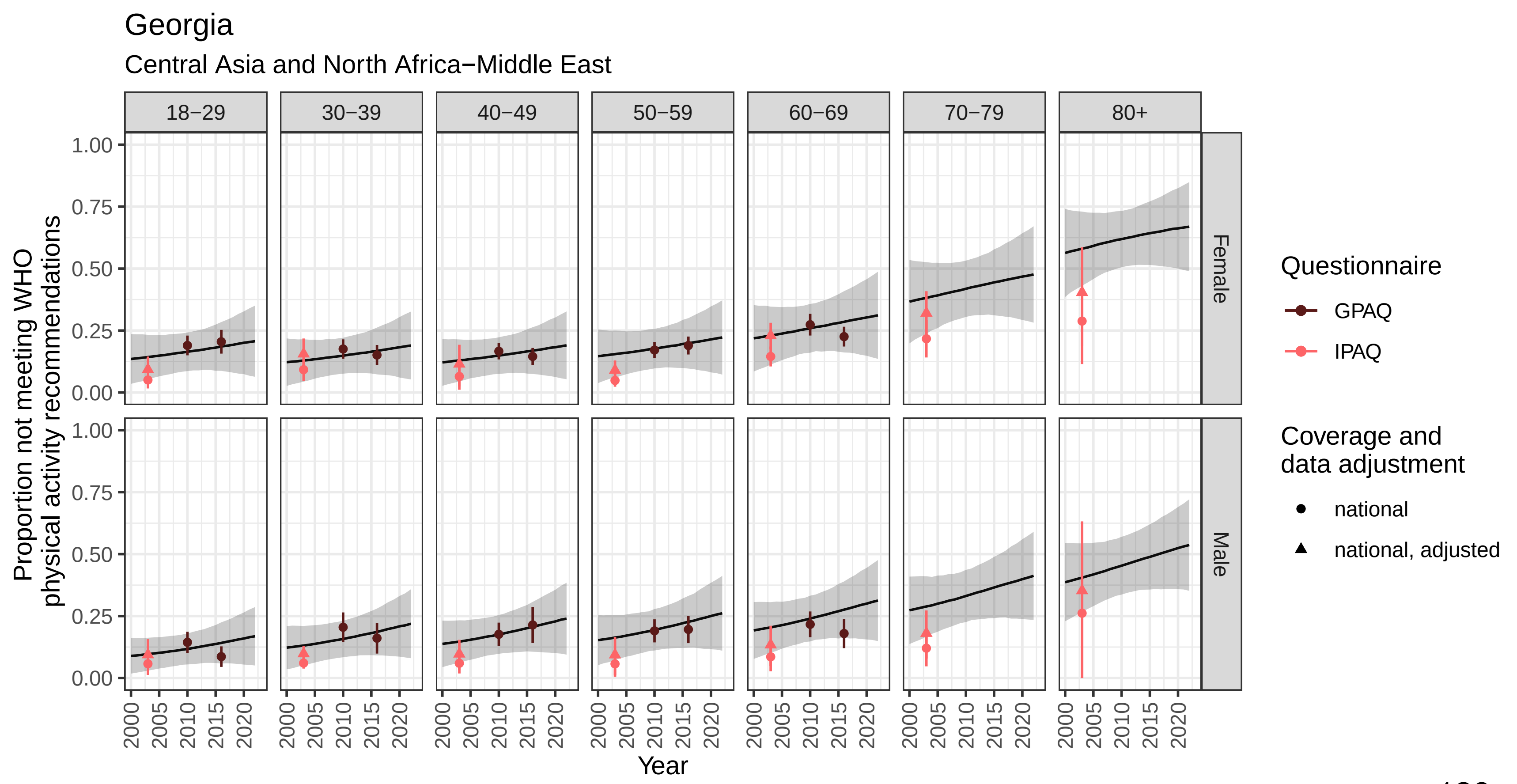

Notes: vertical lines show input data 95% confidence interval; black line shows estimate; shaded area shows 95% uncertainty interval of estimate

Germany

High−income Western countries

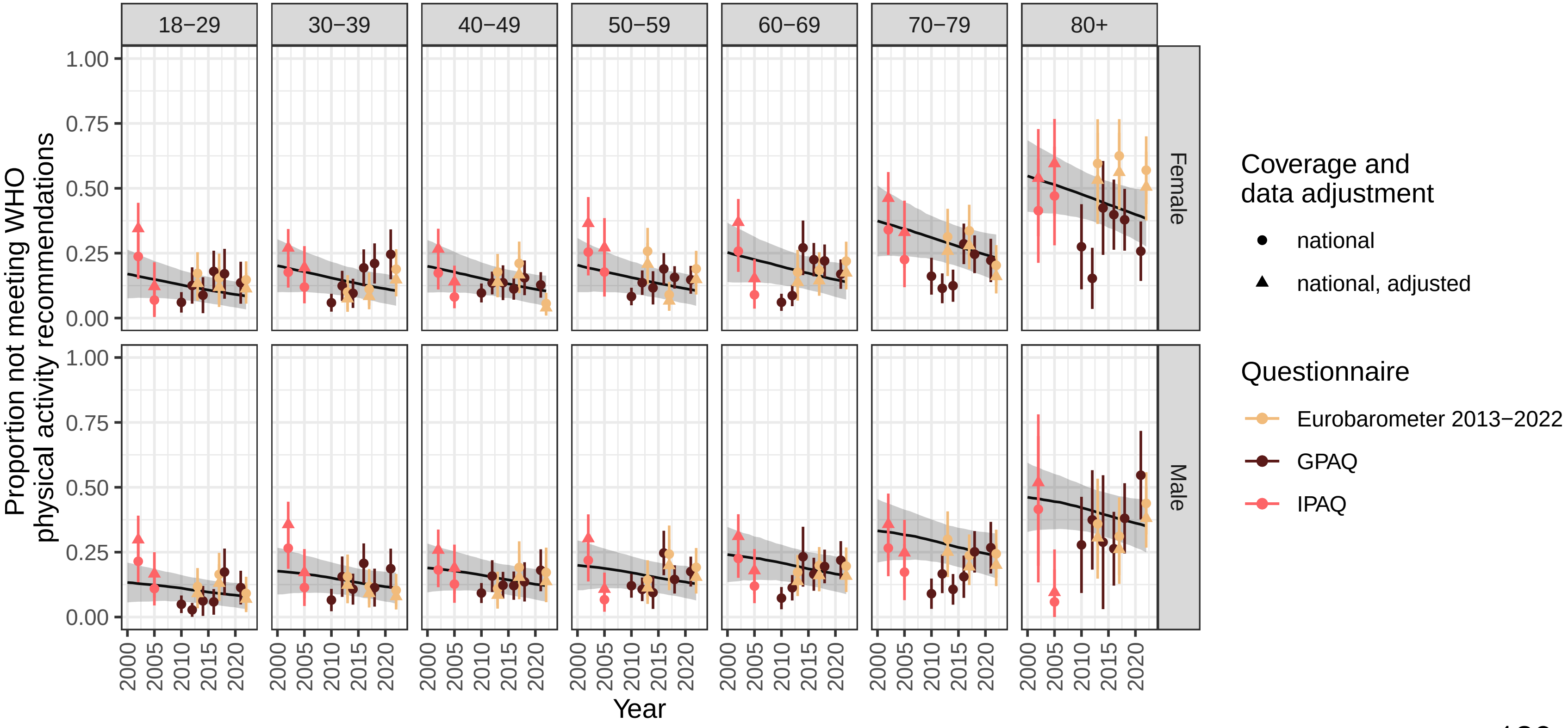

Notes: vertical lines show input data 95% confidence interval; black line shows estimate; shaded area shows 95% uncertainty interval of estimate

Ghana

Sub−Saharan Africa

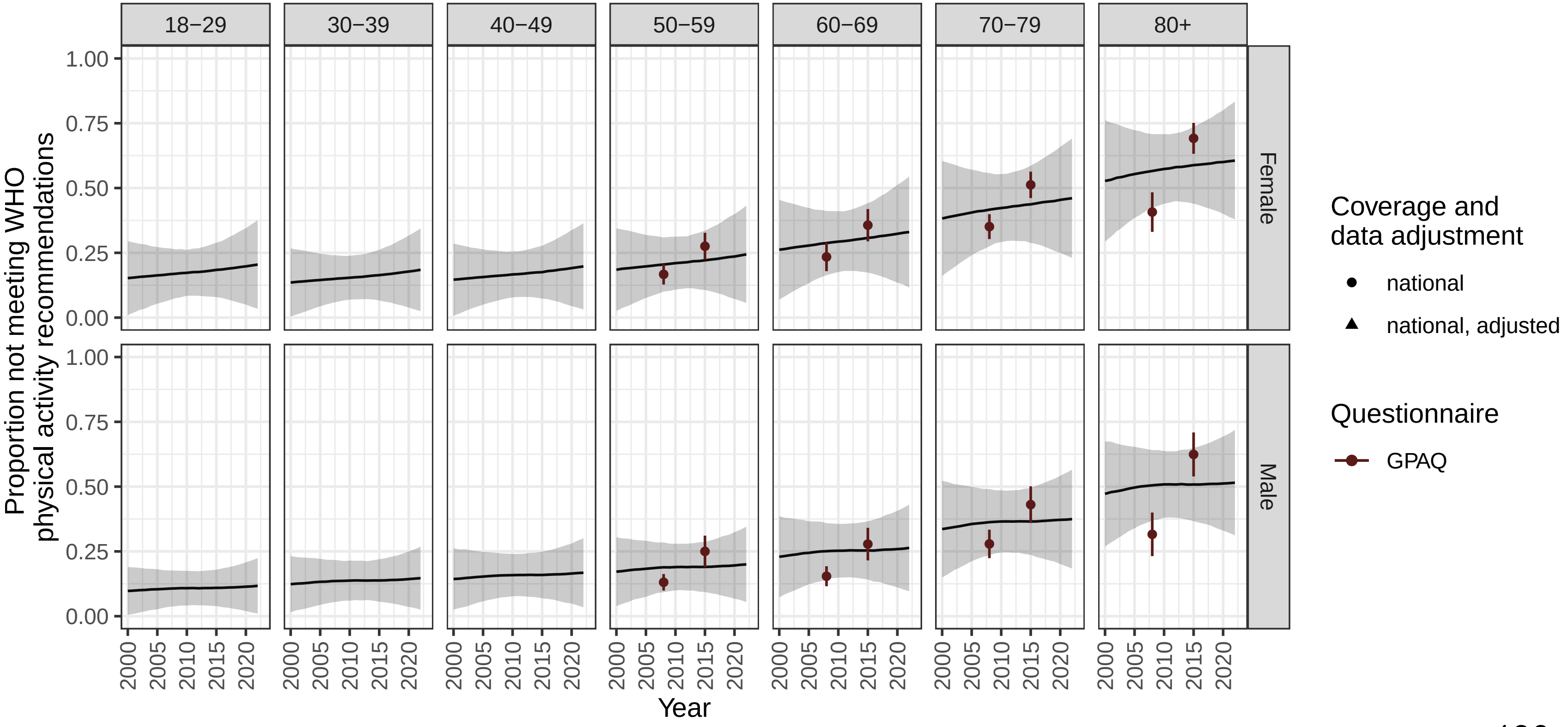

Notes: vertical lines show input data 95% confidence interval; black line shows estimate; shaded area shows 95% uncertainty interval of estimate

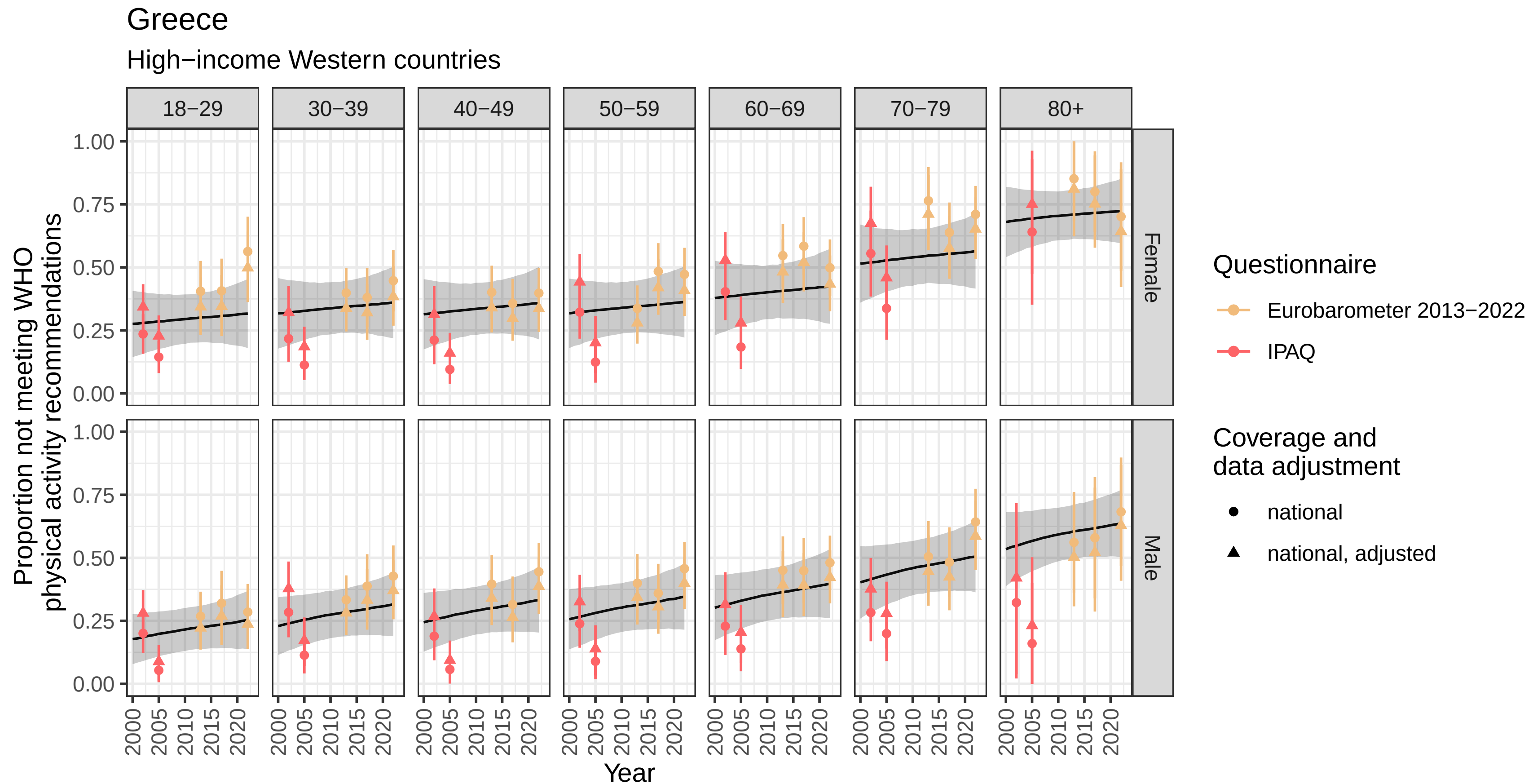

Notes: vertical lines show input data 95% confidence interval; black line shows estimate; shaded area shows 95% uncertainty interval of estimate

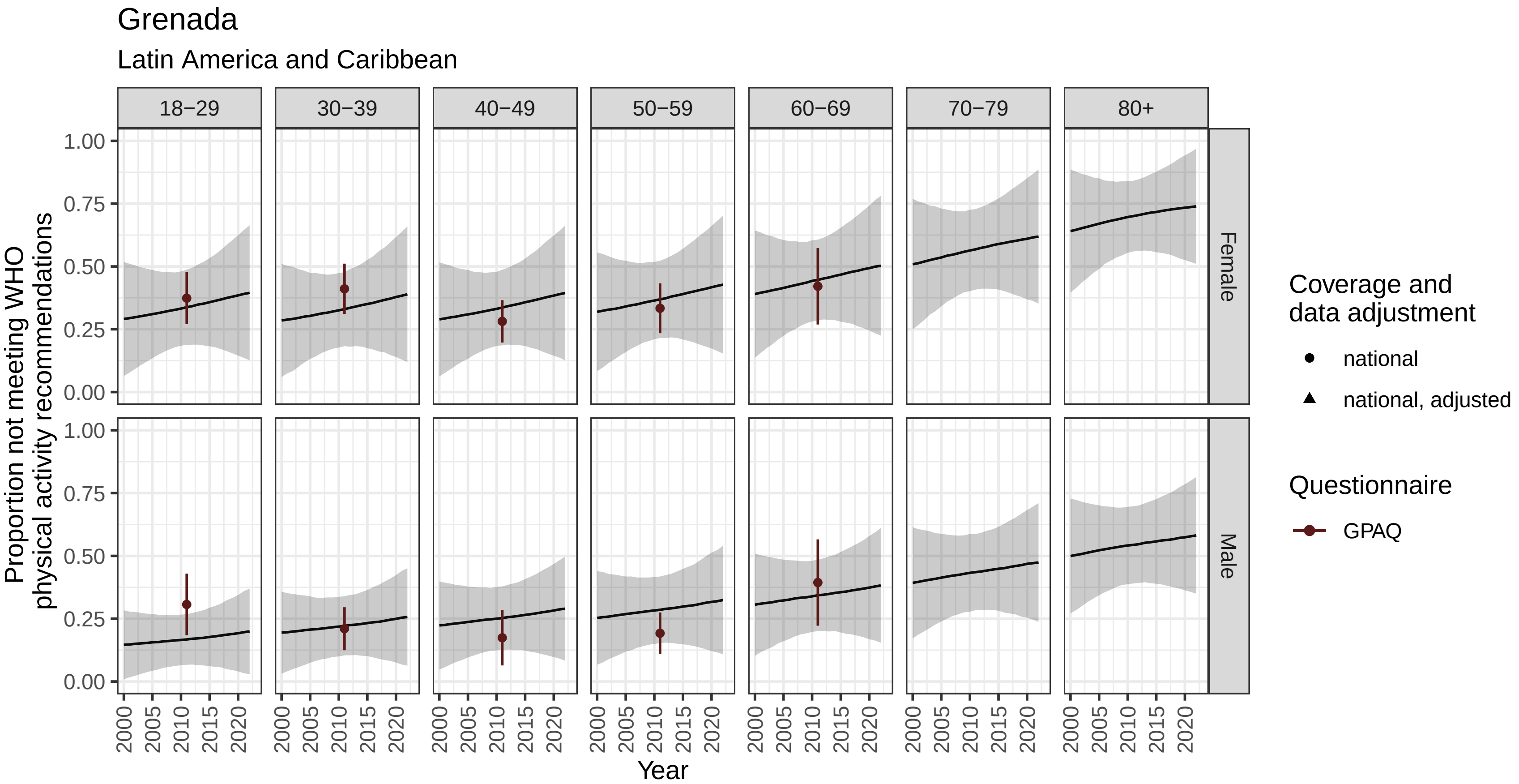

Notes: vertical lines show input data 95% confidence interval; black line shows estimate; shaded area shows 95% uncertainty interval of estimate

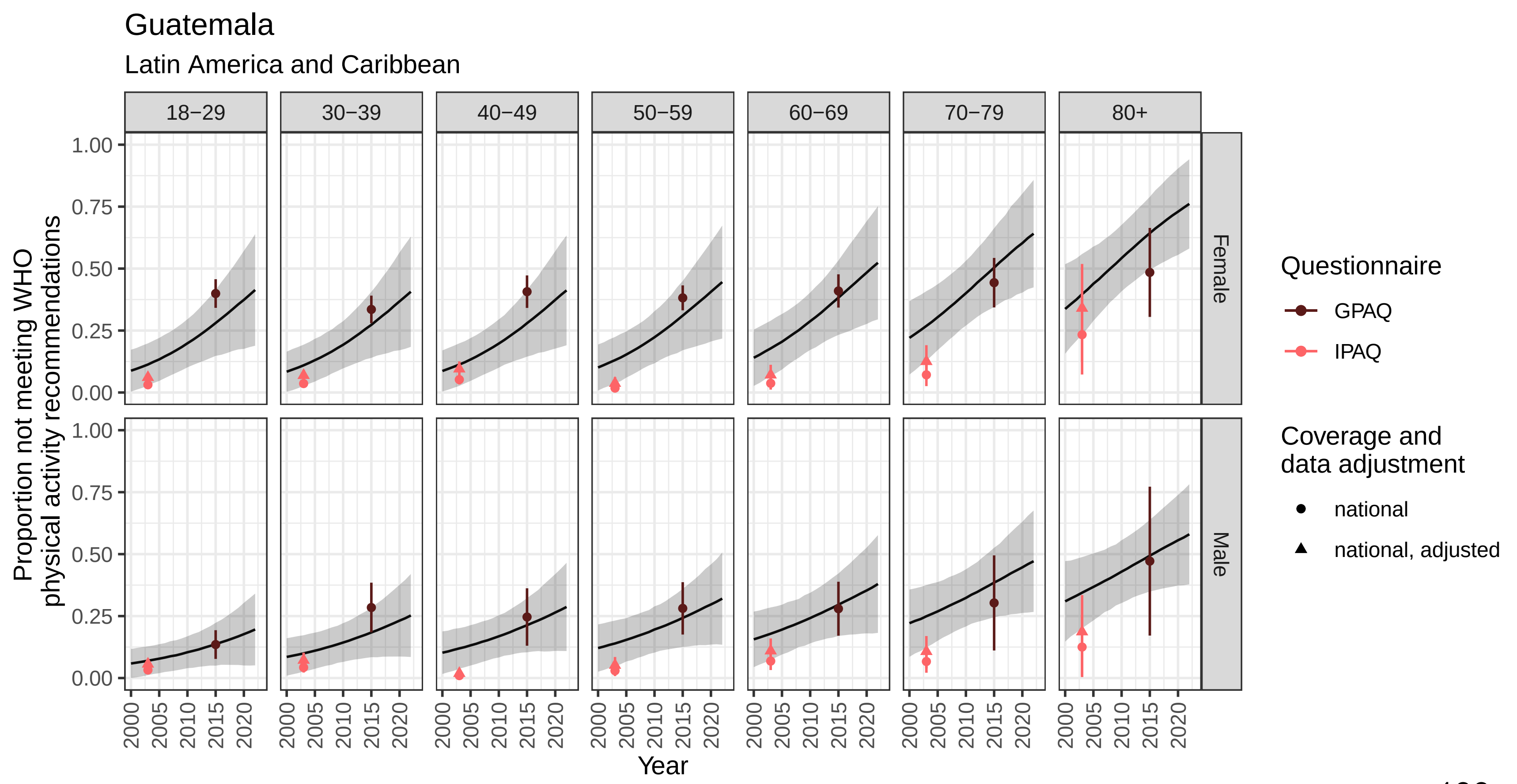

Notes: vertical lines show input data 95% confidence interval; black line shows estimate; shaded area shows 95% uncertainty interval of estimate

Guinea

Sub−Saharan Africa

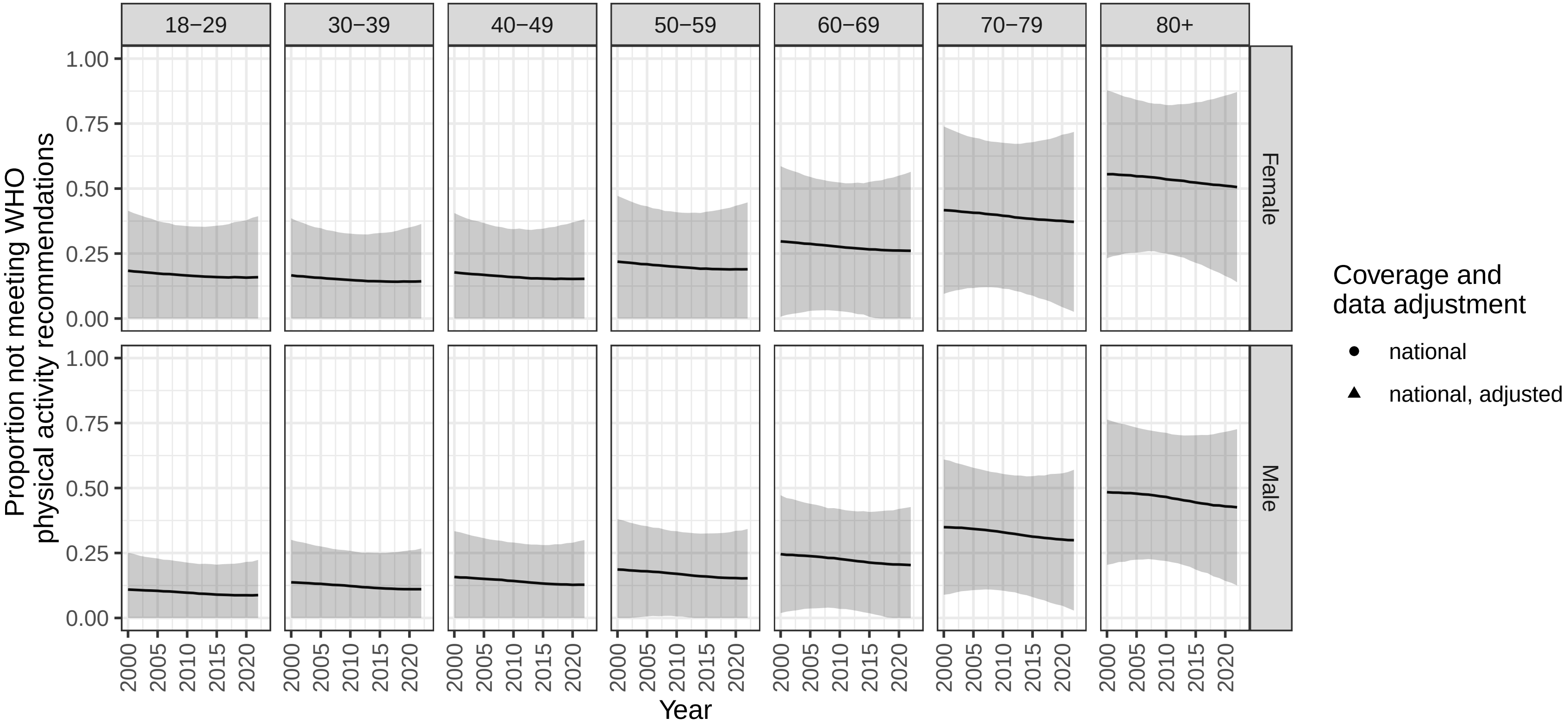

Notes: vertical lines show input data 95% confidence interval; black line shows estimate; shaded area shows 95% uncertainty interval of estimate

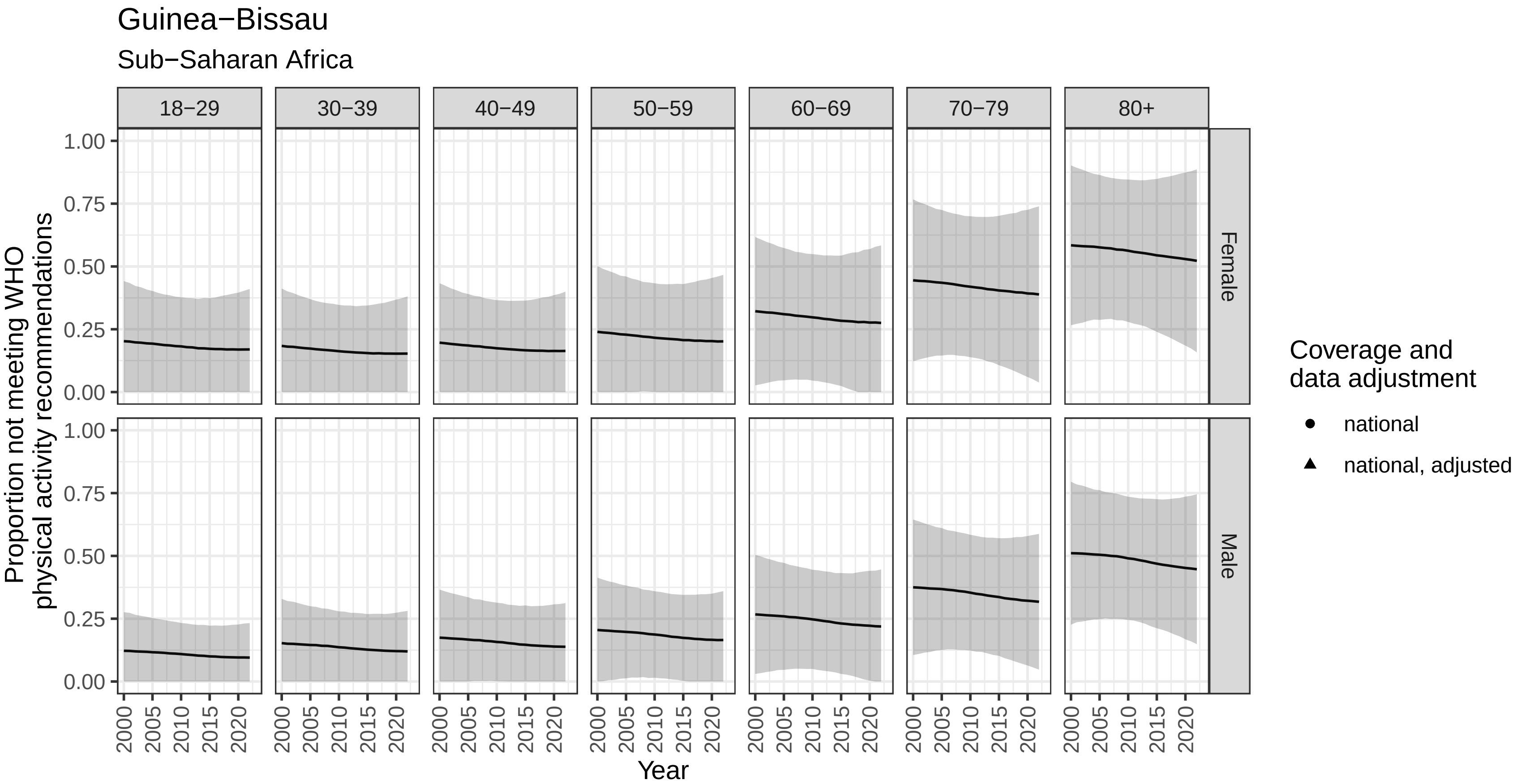

Notes: vertical lines show input data 95% confidence interval; black line shows estimate; shaded area shows 95% uncertainty interval of estimate

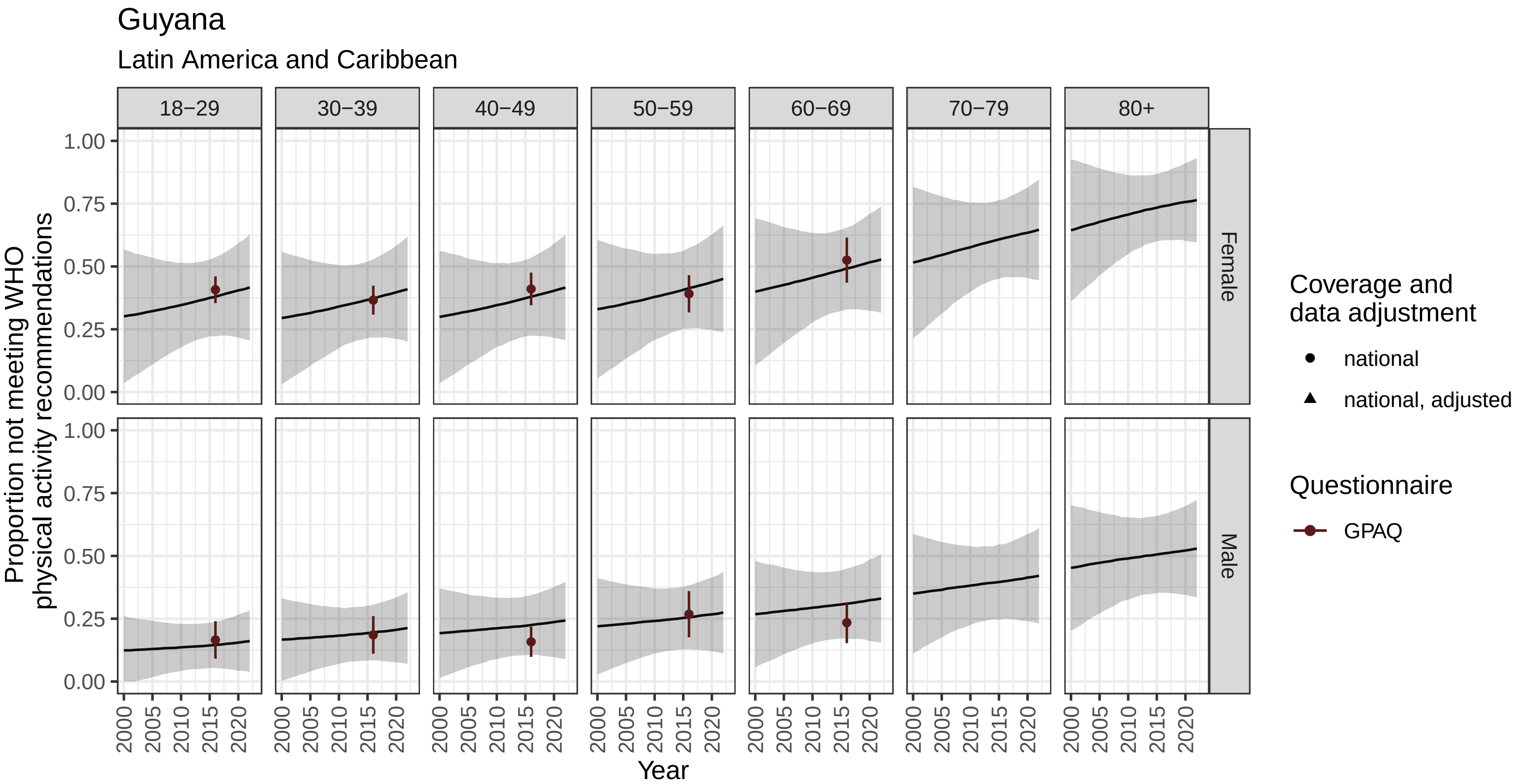

Notes: vertical lines show input data 95% confidence interval; black line shows estimate; shaded area shows 95% uncertainty interval of estimate

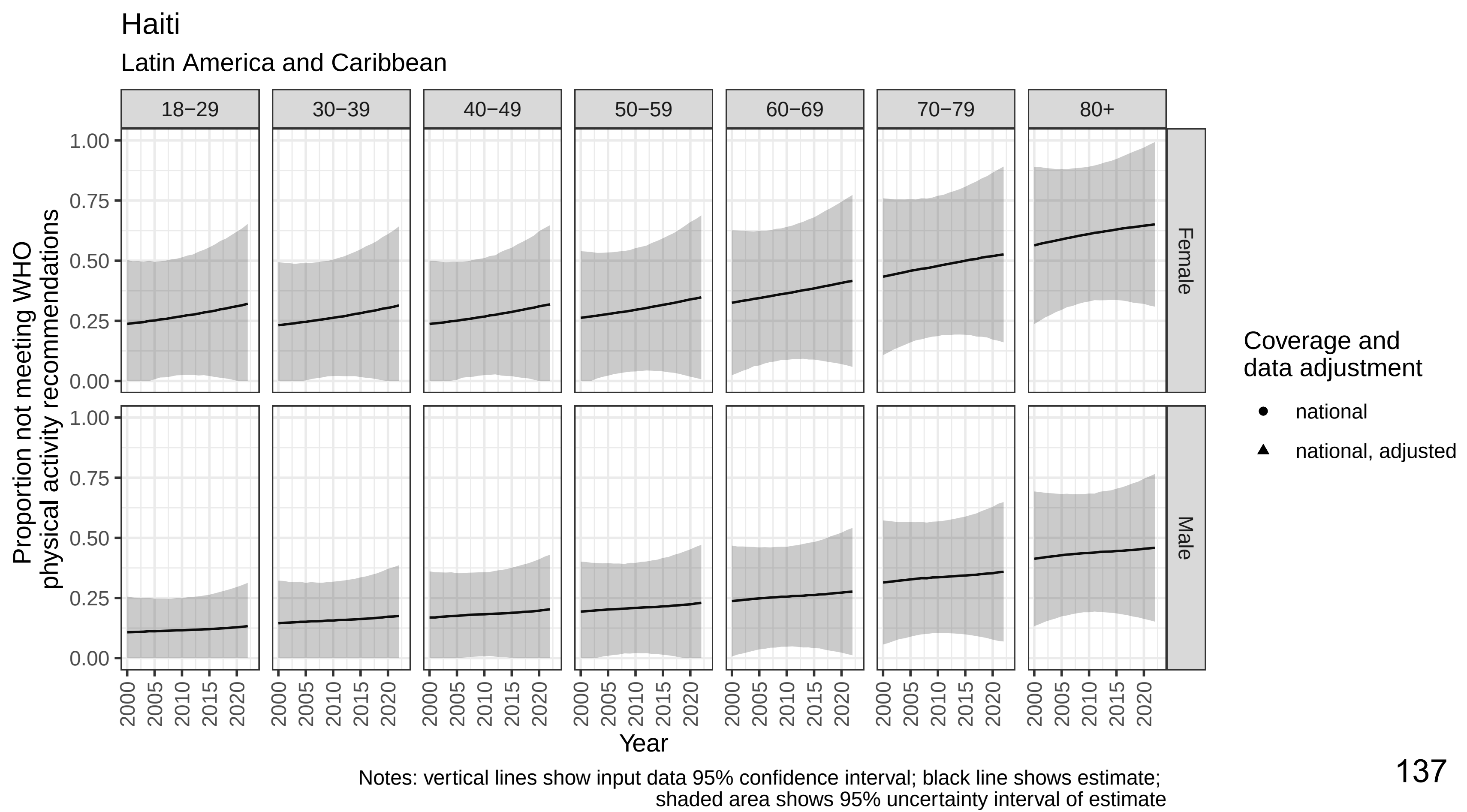

Haiti
Latin America and Caribbean
18−29
30−39
40−49
50−59
60−69
70−79
80+
Female
Male
Proportion not meeting WHO physical activity recommendations
0.00
0.25
0.50
0.75
1.00
2000
2005
2010
2015
2020
Year
Coverage and data adjustment
national
national, adjusted
Notes: vertical lines show input data 95% confidence interval; black line shows estimate;
shaded area shows 95% uncertainty interval of estimate

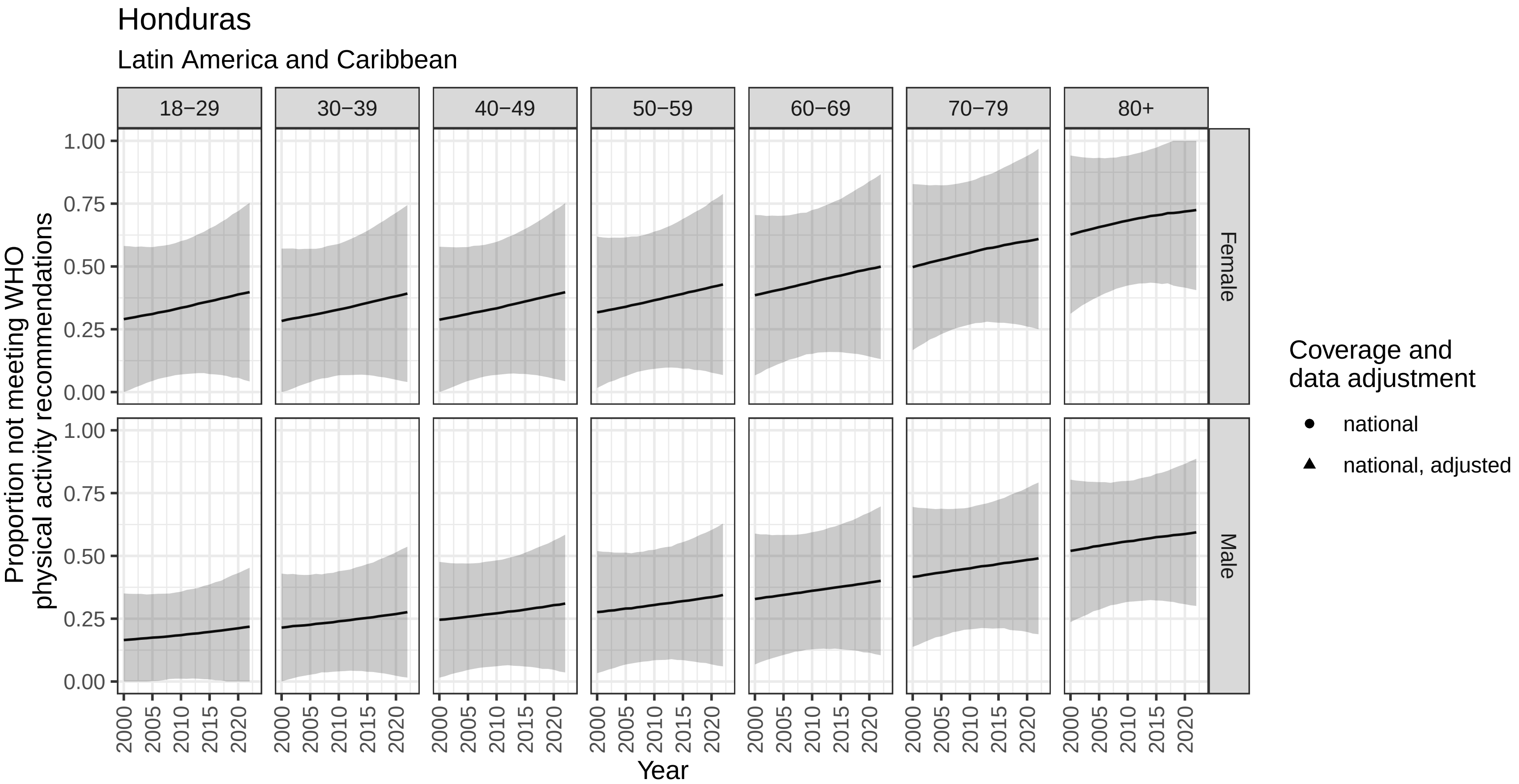

Notes: vertical lines show input data 95% confidence interval; black line shows estimate; shaded area shows 95% uncertainty interval of estimate

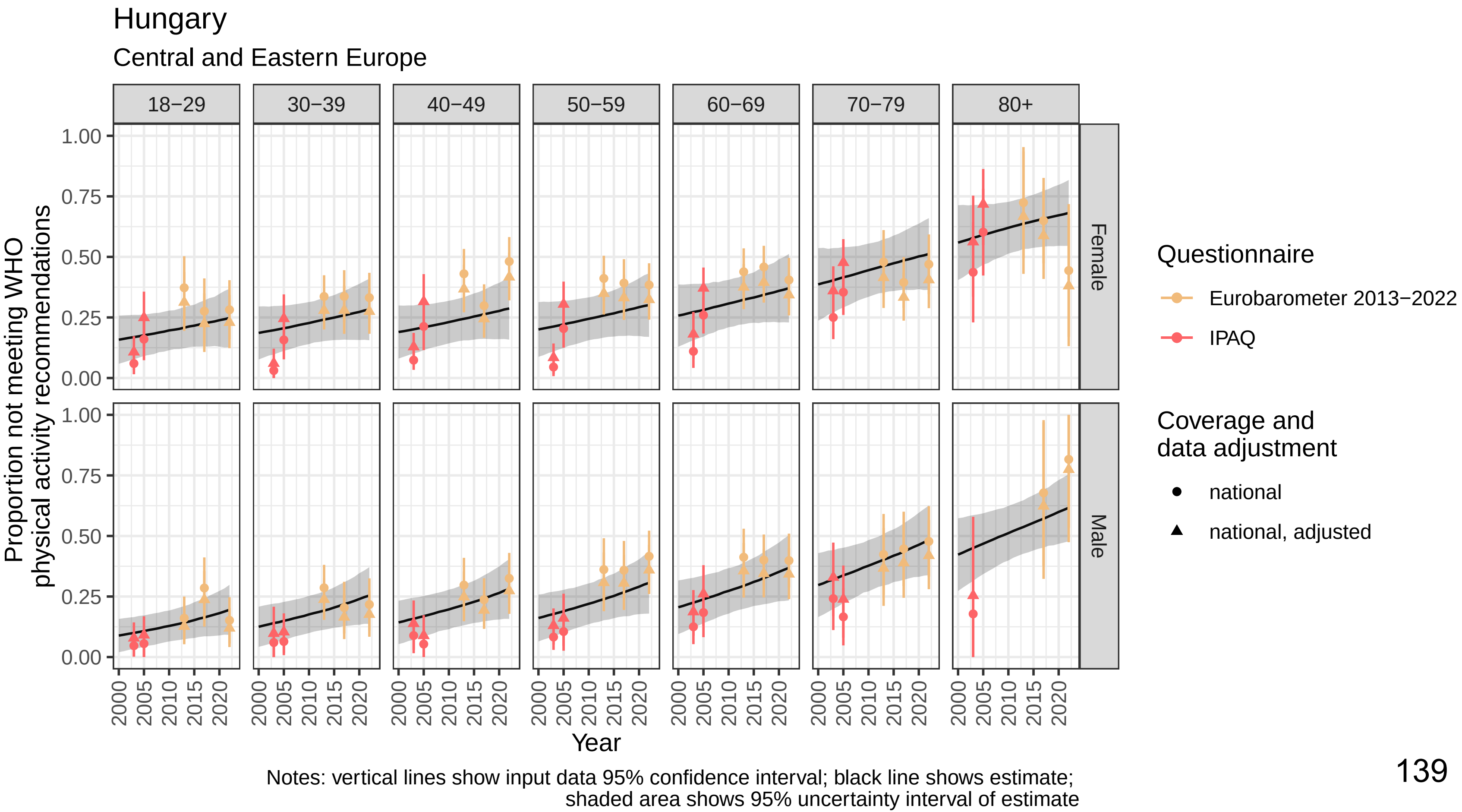
Hungary
Central and Eastern Europe
18−29
30−39
40−49
50−59
60−69
70−79
80+
Female
Male
Proportion not meeting WHO physical activity recommendations
Year
Questionnaire
Eurobarometer 2013−2022
IPAQ
Coverage and data adjustment
national
national, adjusted
Notes: vertical lines show input data 95% confidence interval; black line shows estimate;
shaded area shows 95% uncertainty interval of estimate

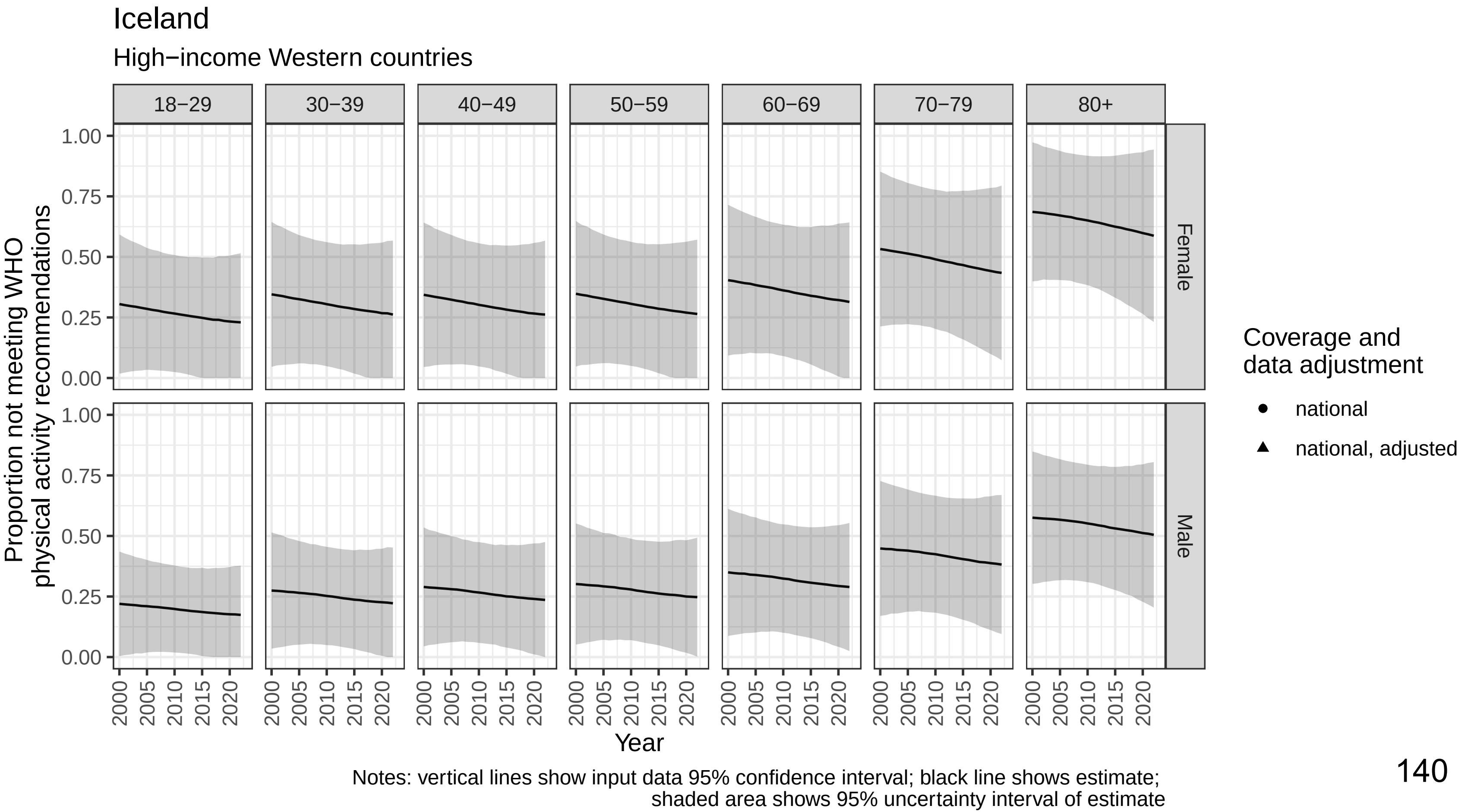
Iceland
High−income Western countries
18−29
30−39
40−49
50−59
60−69
70−79
80+
Female
Male
Proportion not meeting WHO physical activity recommendations
1.00
0.75
0.50
0.25
0.00
2000
2005
2010
2015
2020
Year
Coverage and data adjustment
national
national, adjusted
Notes: vertical lines show input data 95% confidence interval; black line shows estimate; shaded area shows 95% uncertainty interval of estimate

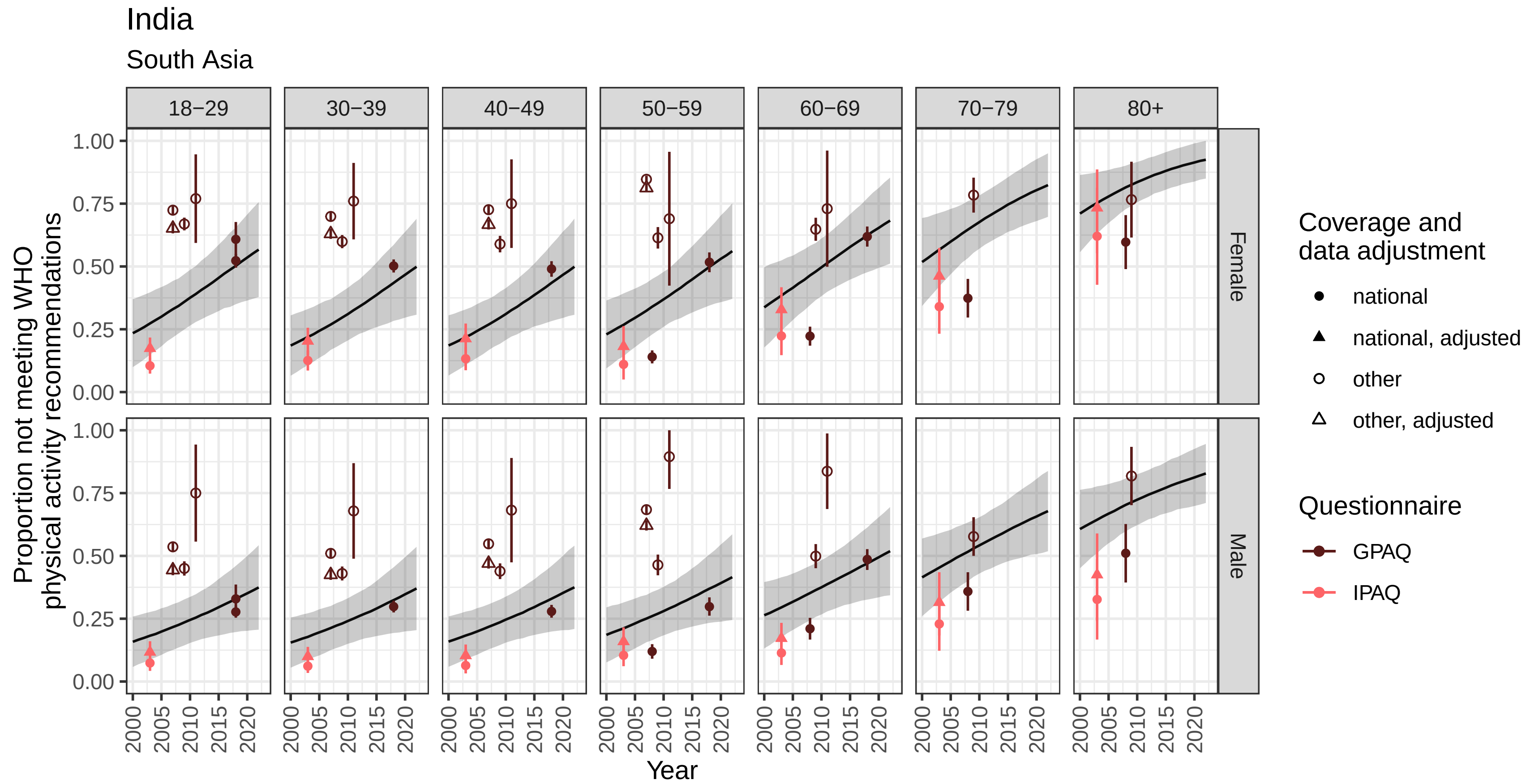

Notes: vertical lines show input data 95% confidence interval; black line shows estimate; shaded area shows 95% uncertainty interval of estimate

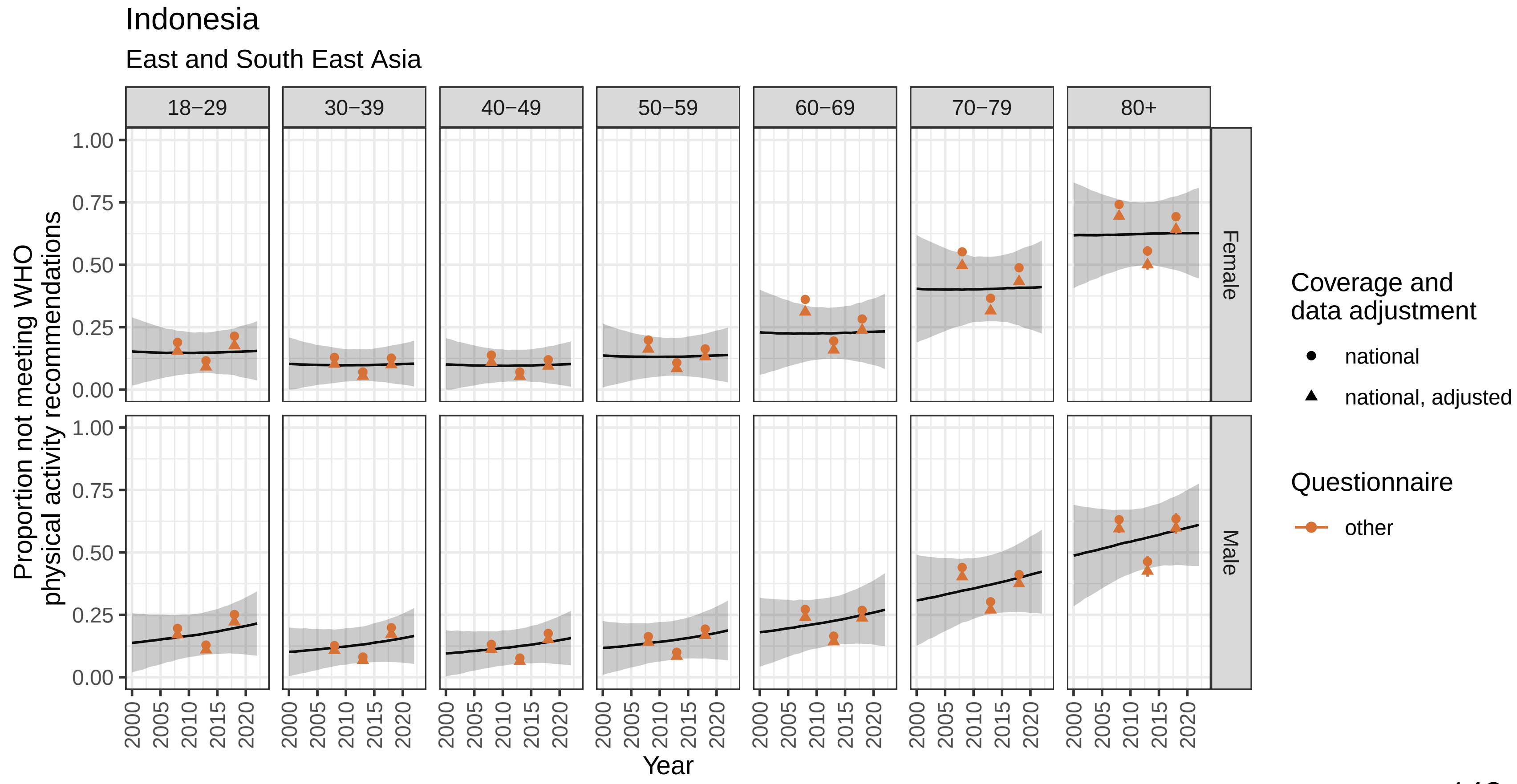

Notes: vertical lines show input data 95% confidence interval; black line shows estimate; shaded area shows 95% uncertainty interval of estimate

Iran (Islamic Republic of)

Central Asia and North Africa−Middle East

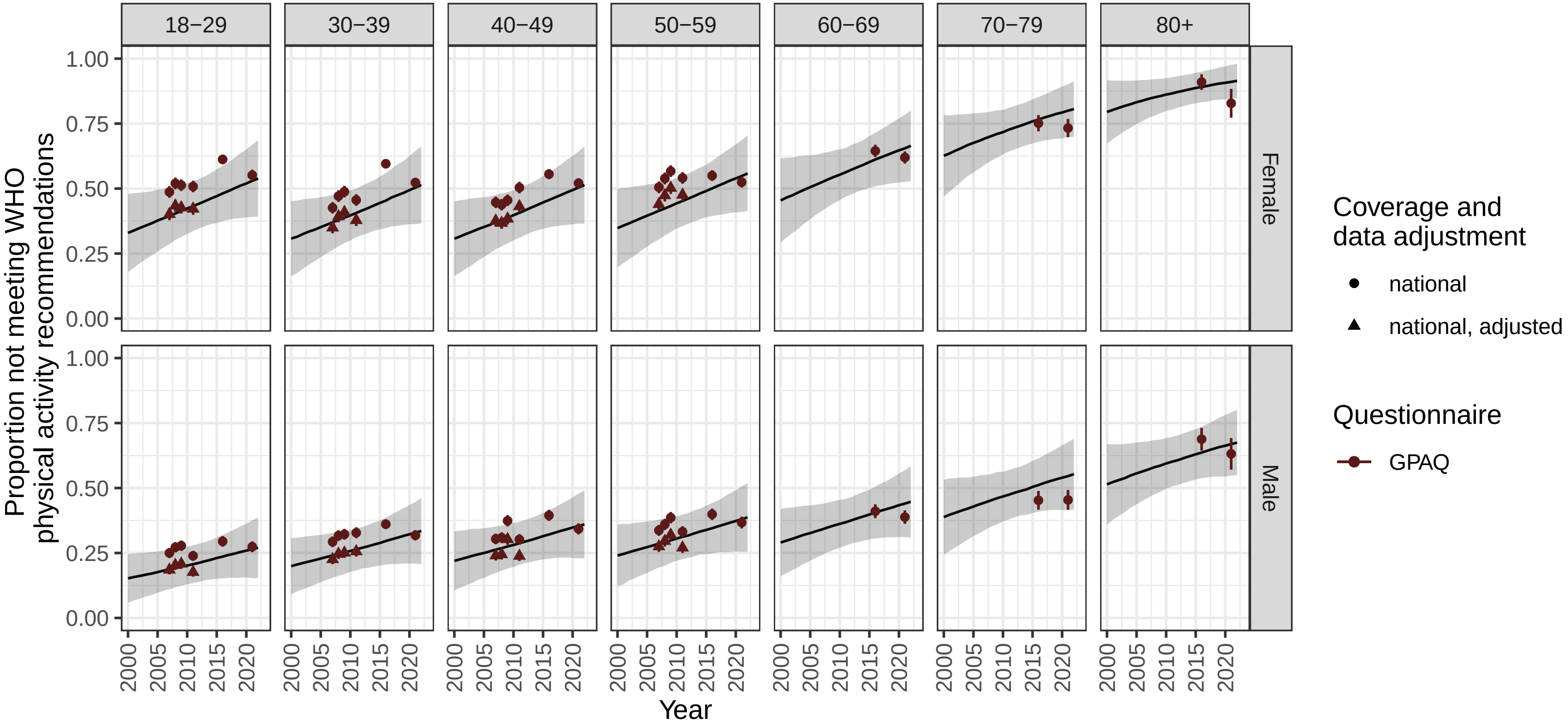

Notes: vertical lines show input data 95% confidence interval; black line shows estimate; shaded area shows 95% uncertainty interval of estimate

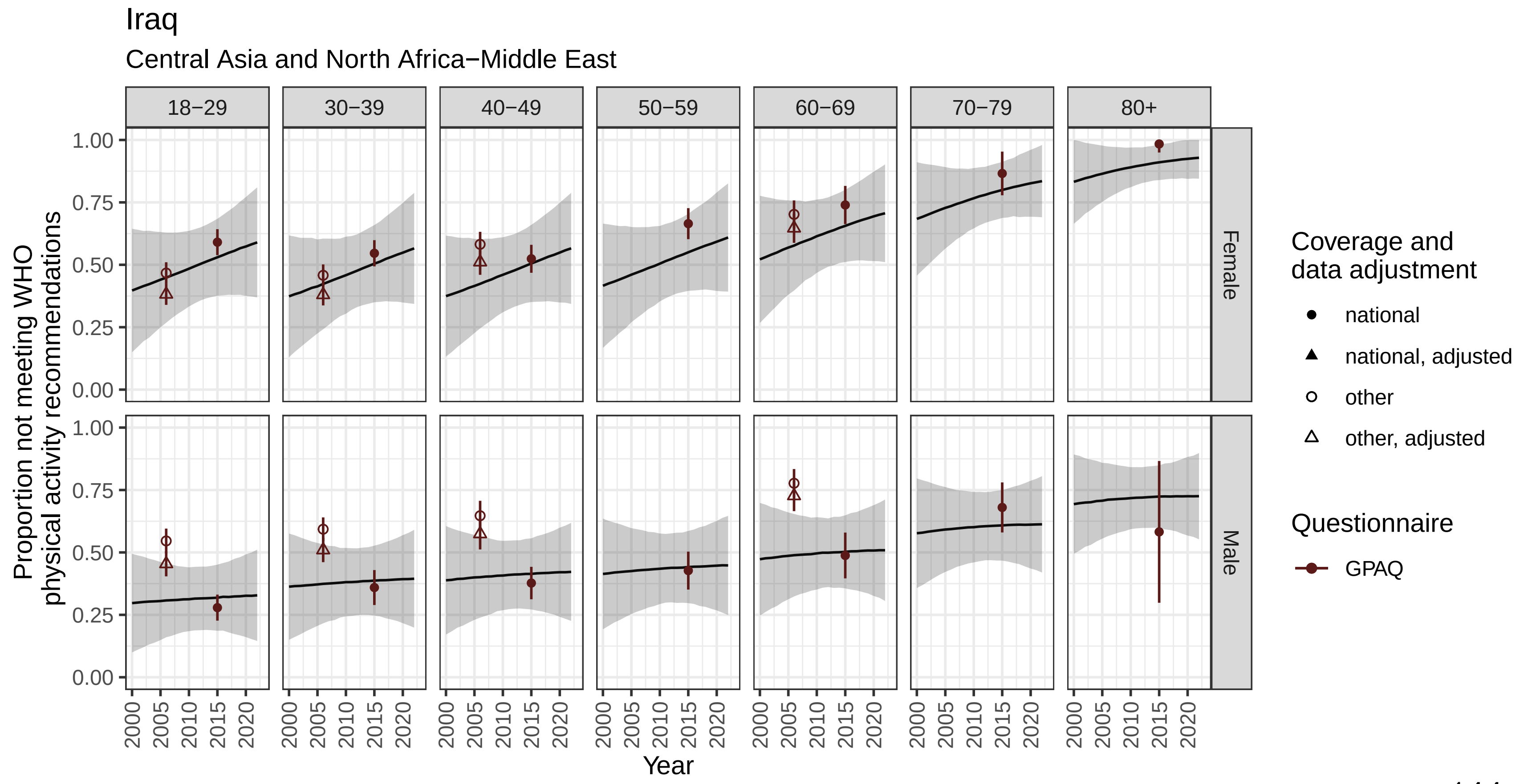

Notes: vertical lines show input data 95% confidence interval; black line shows estimate; shaded area shows 95% uncertainty interval of estimate

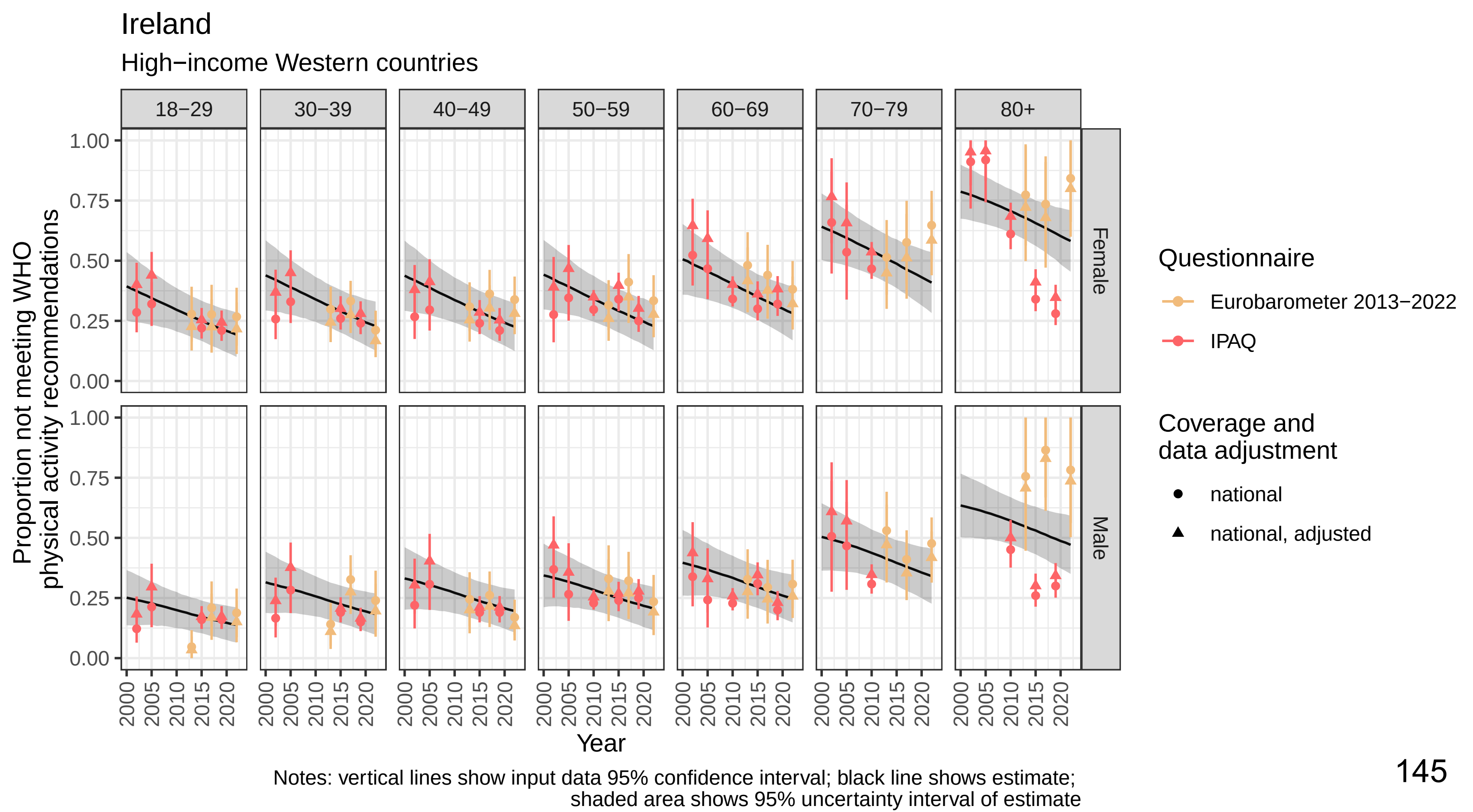
Ireland
High−income Western countries
18−29
30−39
40−49
50−59
60−69
70−79
80+
Female
Male
Proportion not meeting WHO physical activity recommendations
1.00
0.75
0.50
0.25
0.00
2000
2005
2010
2015
2020
Year
Questionnaire
Eurobarometer 2013−2022
IPAQ
Coverage and data adjustment
national
national, adjusted
Notes: vertical lines show input data 95% confidence interval; black line shows estimate;
shaded area shows 95% uncertainty interval of estimate

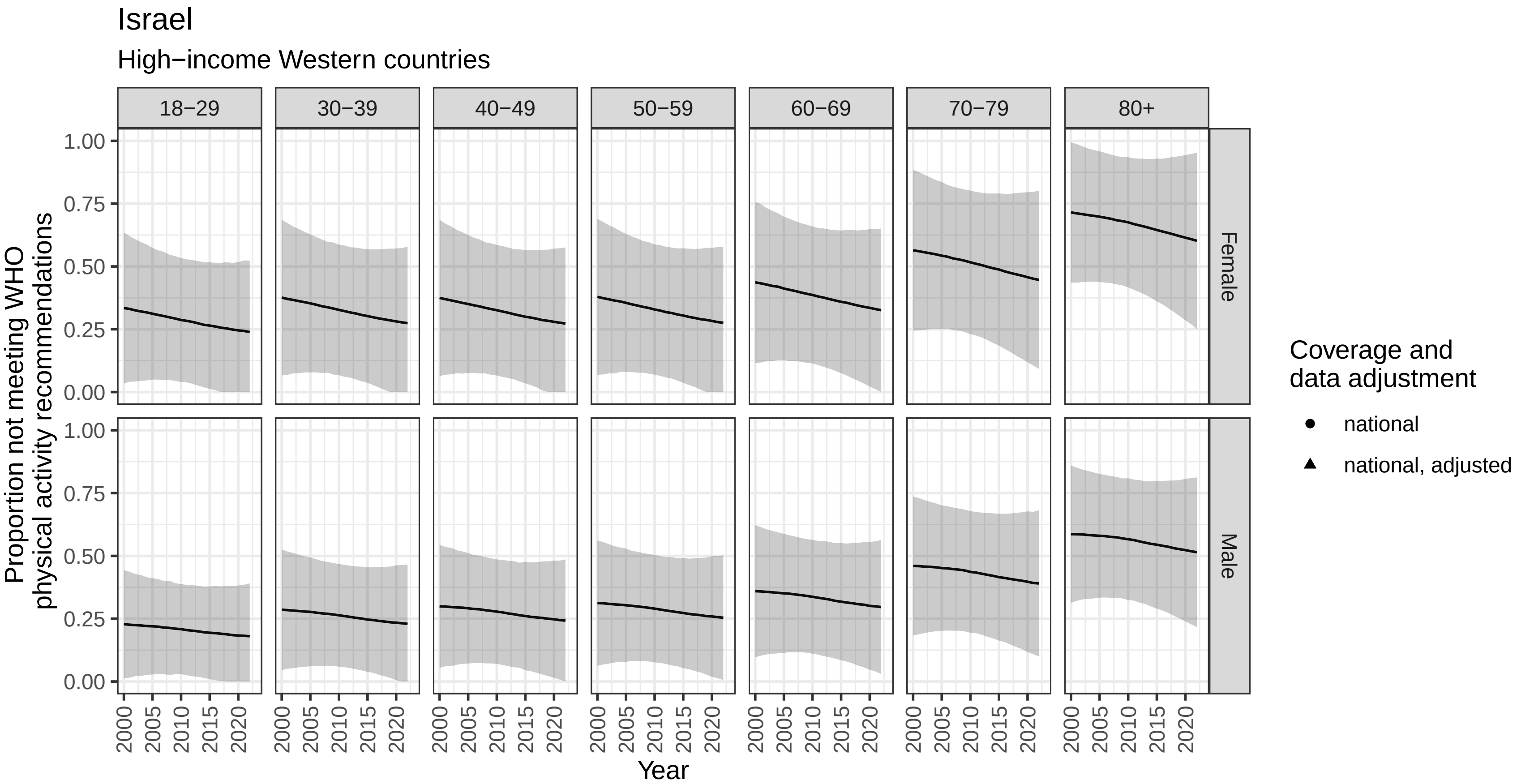

Notes: vertical lines show input data 95% confidence interval; black line shows estimate; shaded area shows 95% uncertainty interval of estimate

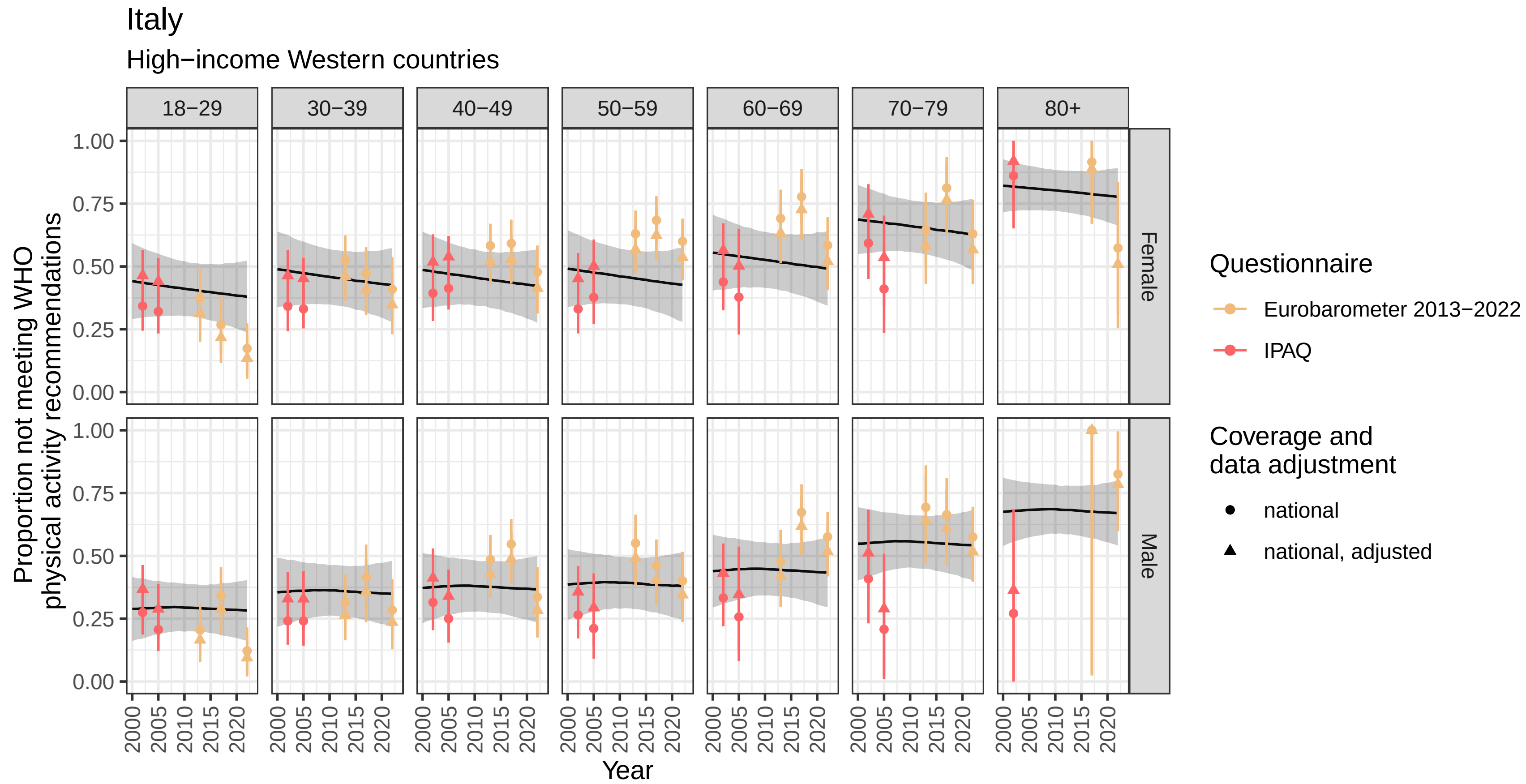

Notes: vertical lines show input data 95% confidence interval; black line shows estimate; shaded area shows 95% uncertainty interval of estimate

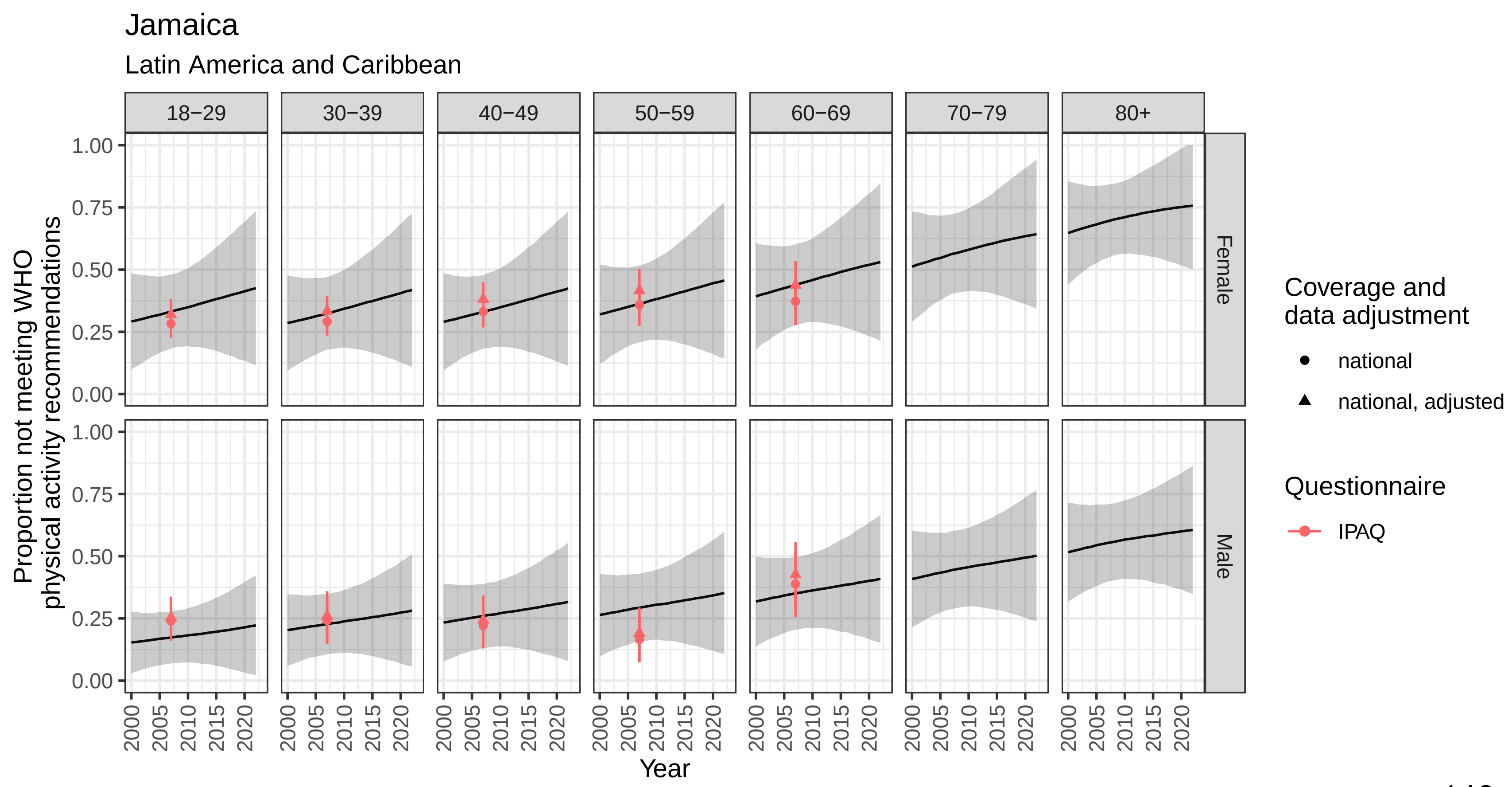

Notes: vertical lines show input data 95% confidence interval; black line shows estimate; shaded area shows 95% uncertainty interval of estimate

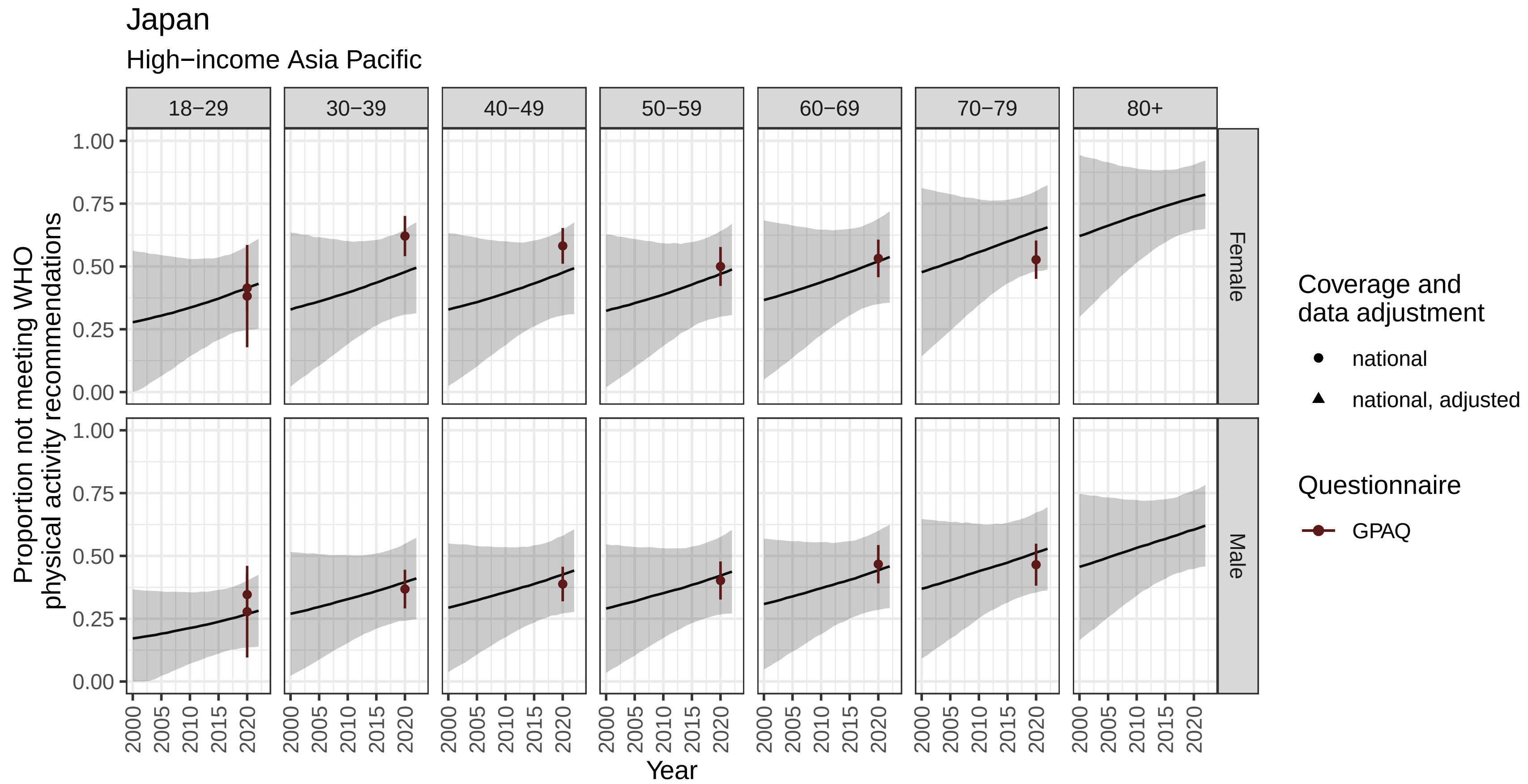

Notes: vertical lines show input data 95% confidence interval; black line shows estimate; shaded area shows 95% uncertainty interval of estimate

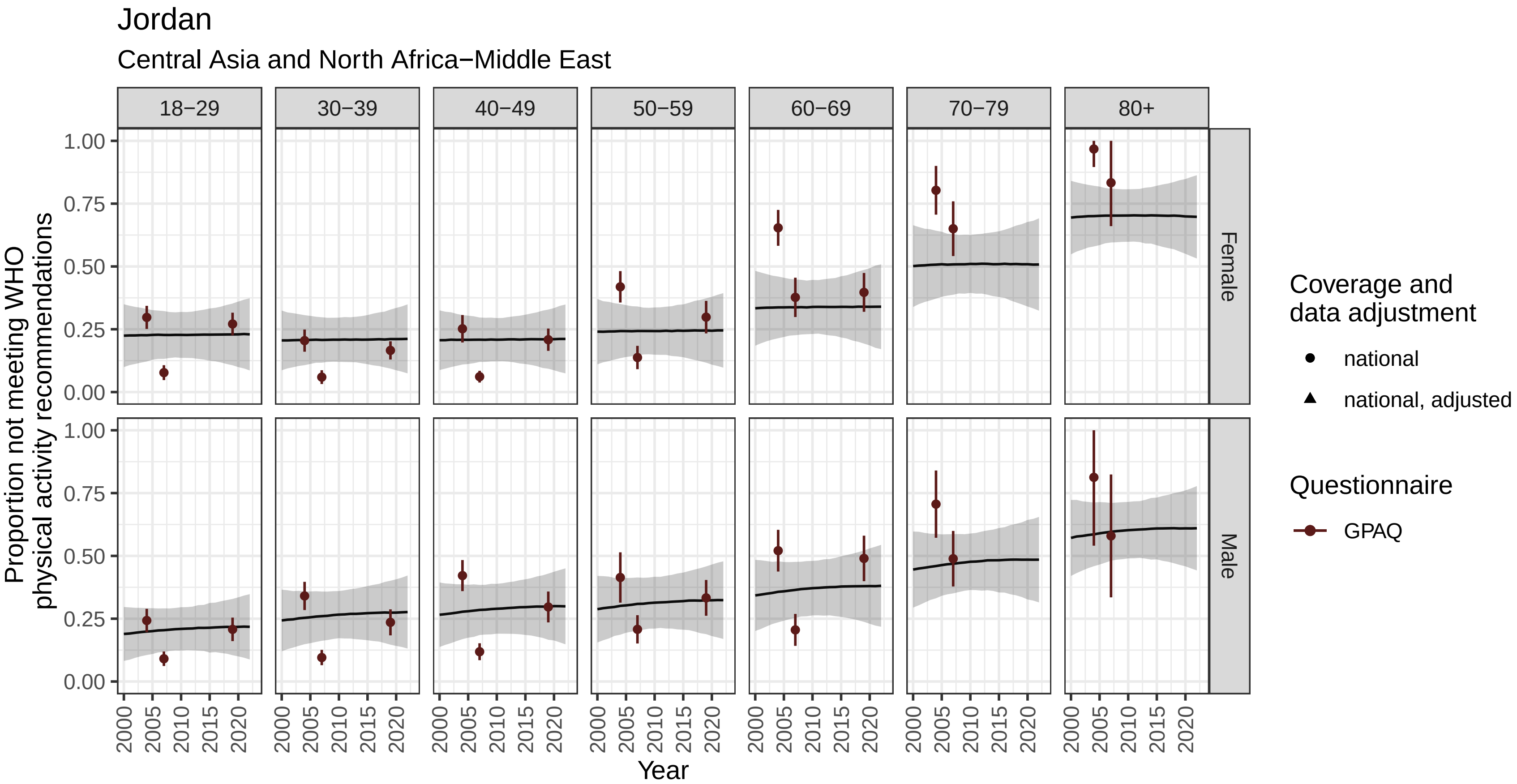

Notes: vertical lines show input data 95% confidence interval; black line shows estimate; shaded area shows 95% uncertainty interval of estimate

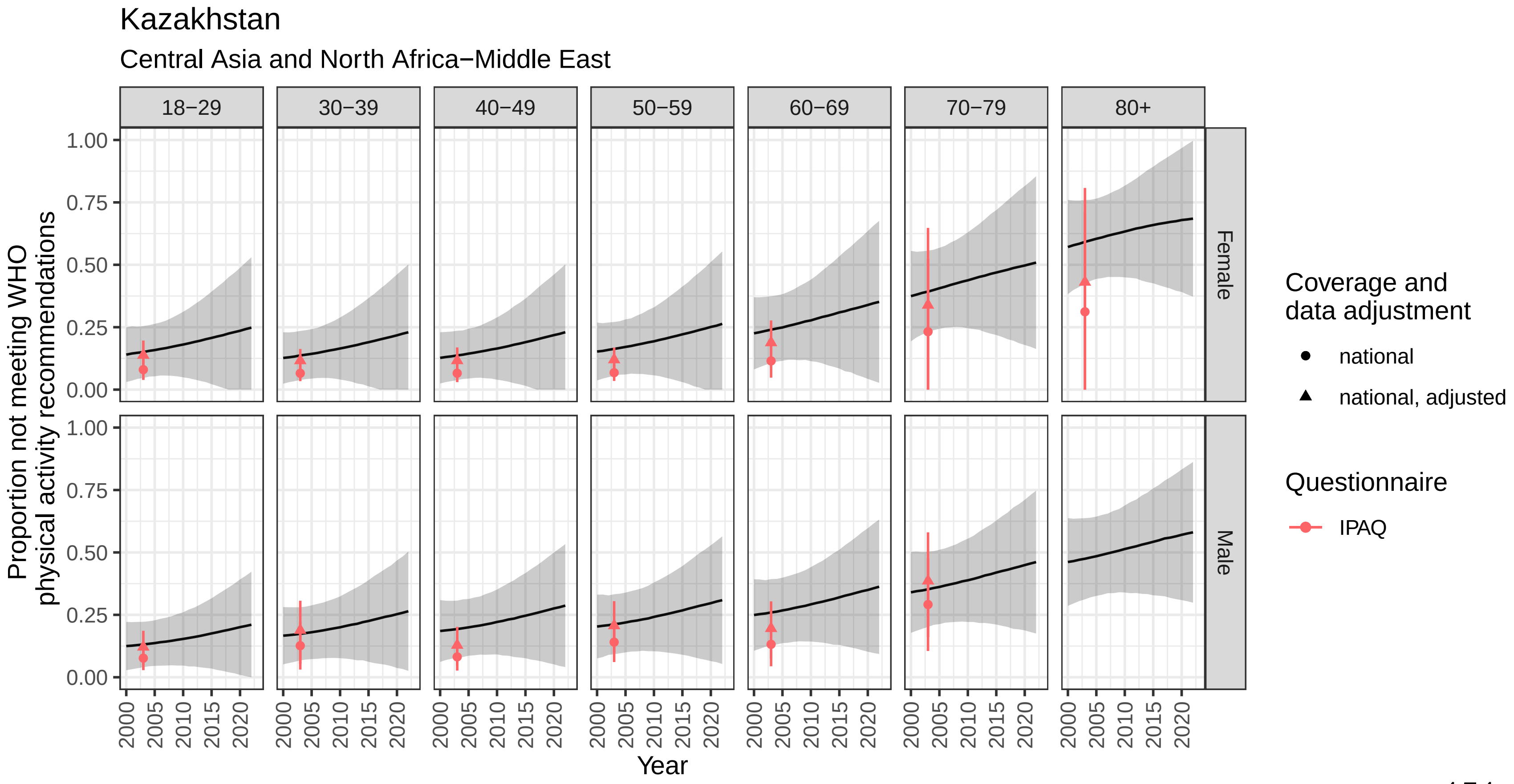

Notes: vertical lines show input data 95% confidence interval; black line shows estimate; shaded area shows 95% uncertainty interval of estimate

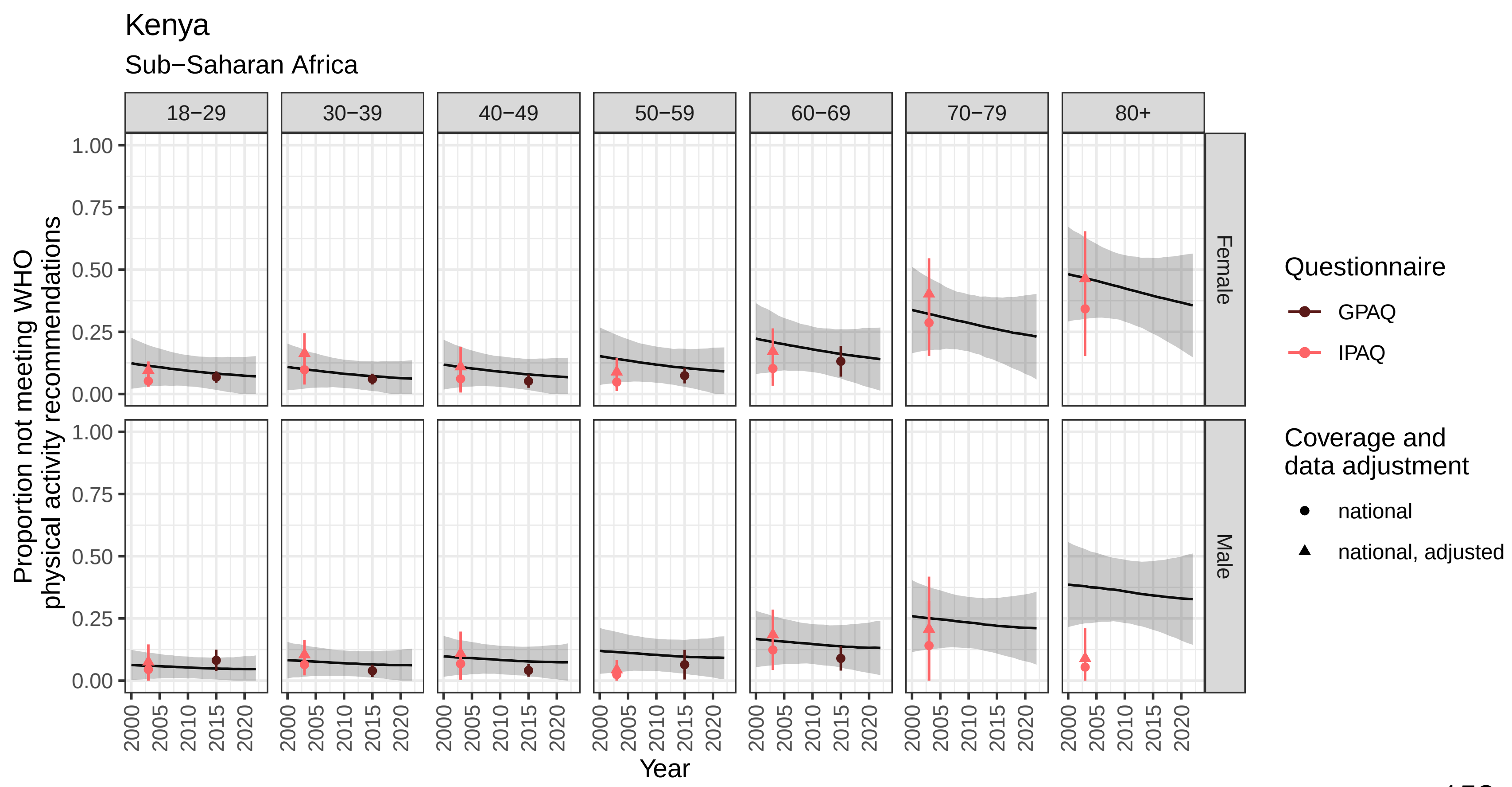

Notes: vertical lines show input data 95% confidence interval; black line shows estimate; shaded area shows 95% uncertainty interval of estimate

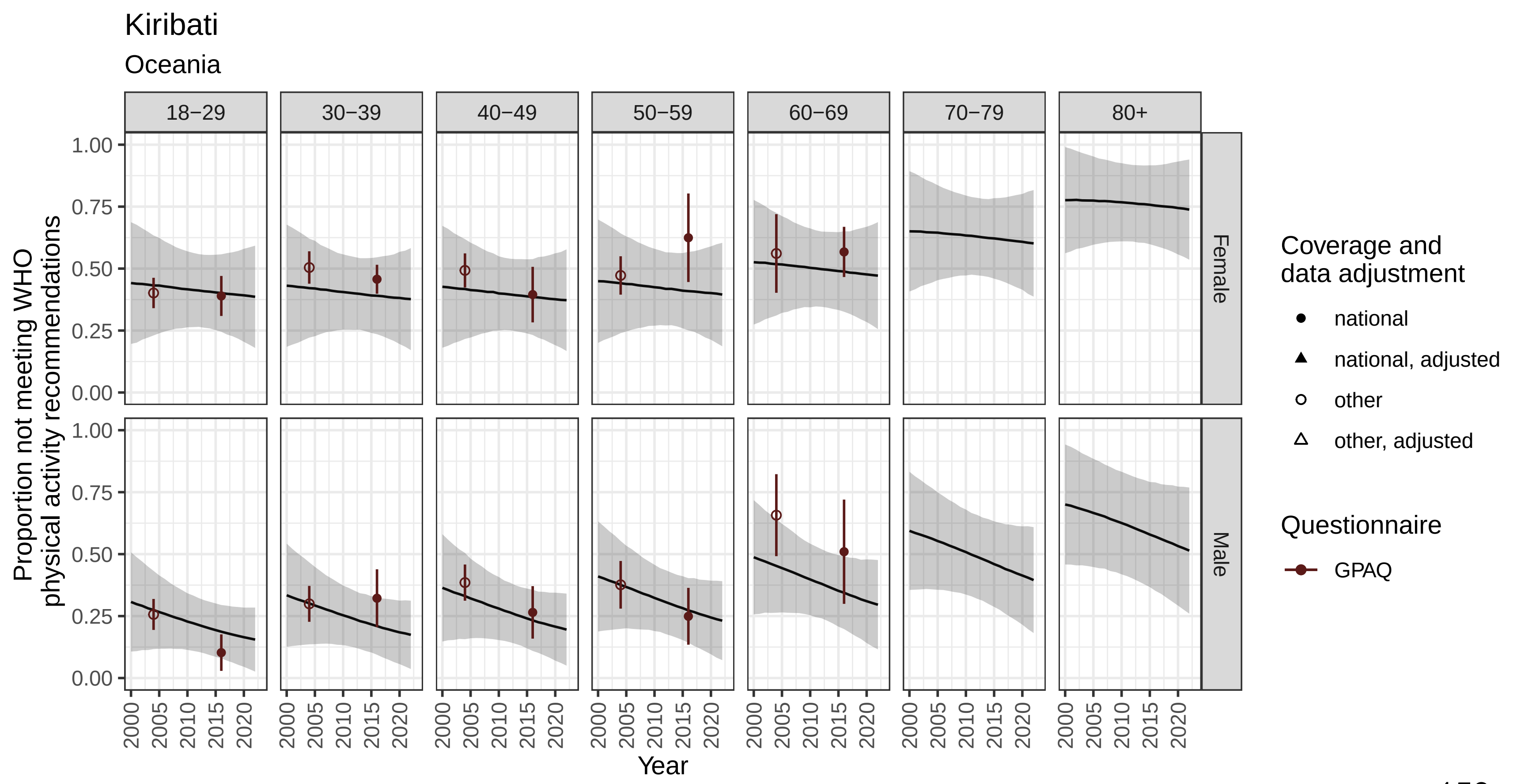

Notes: vertical lines show input data 95% confidence interval; black line shows estimate; shaded area shows 95% uncertainty interval of estimate

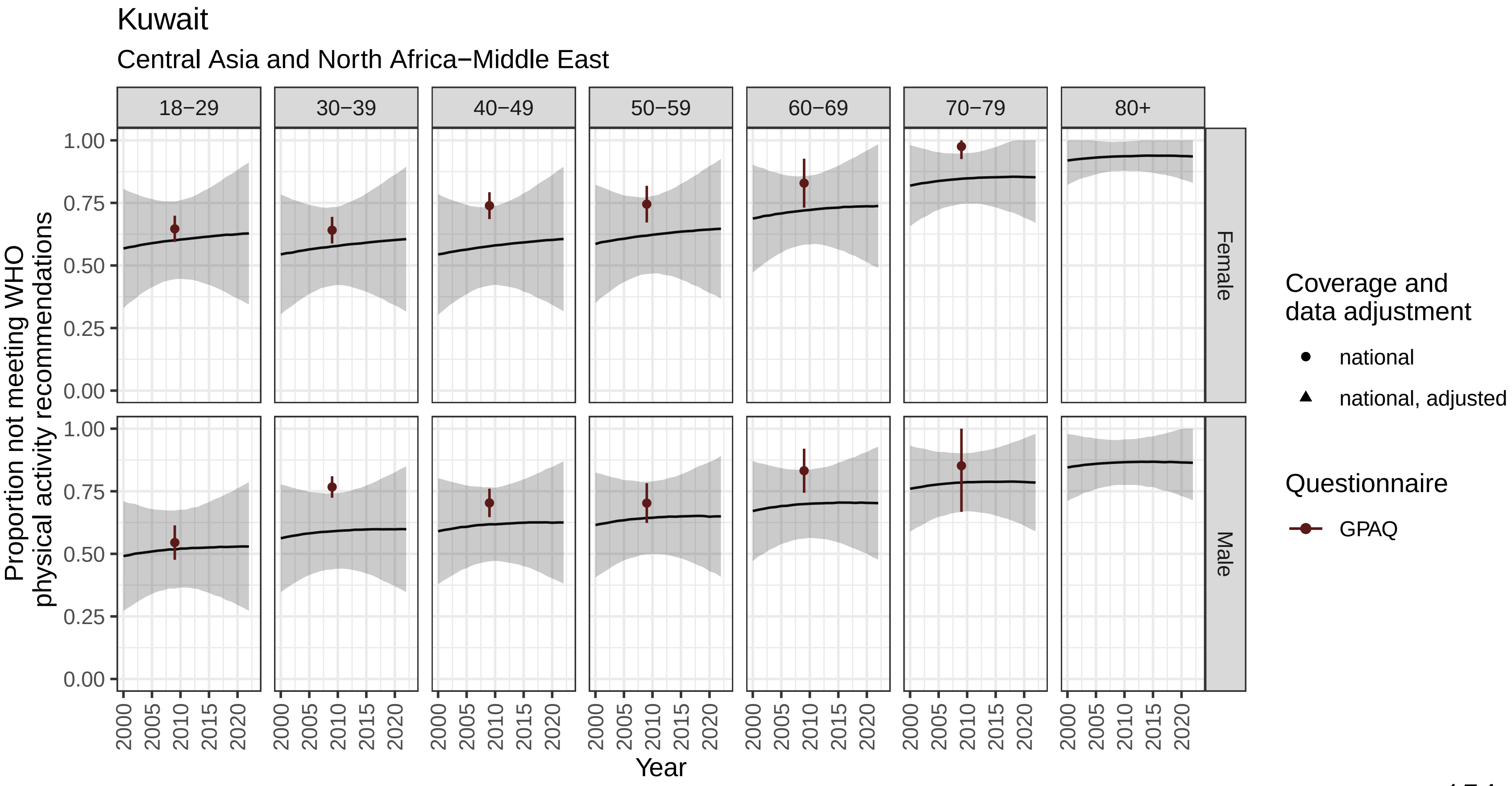

Notes: vertical lines show input data 95% confidence interval; black line shows estimate; shaded area shows 95% uncertainty interval of estimate

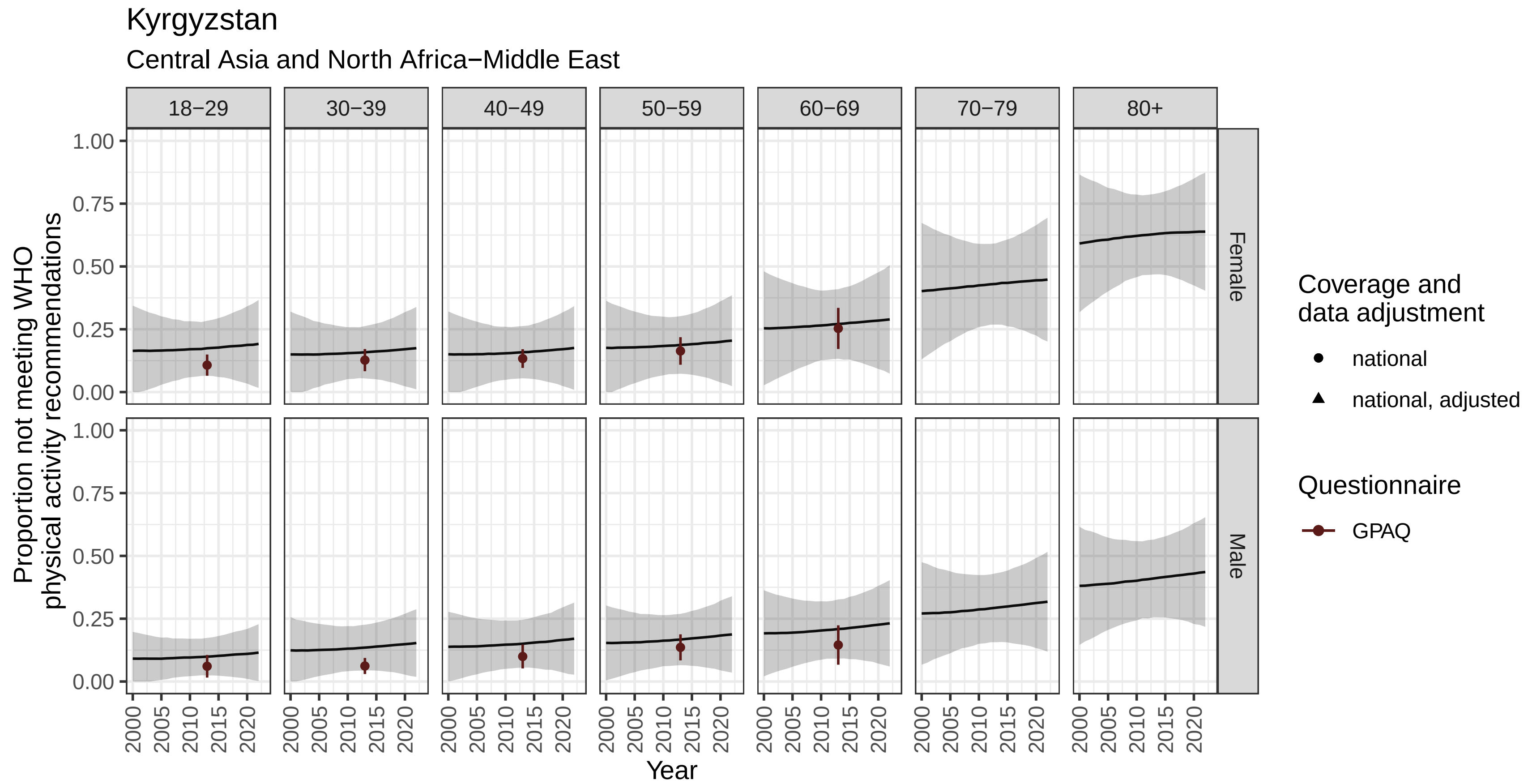

Notes: vertical lines show input data 95% confidence interval; black line shows estimate; shaded area shows 95% uncertainty interval of estimate

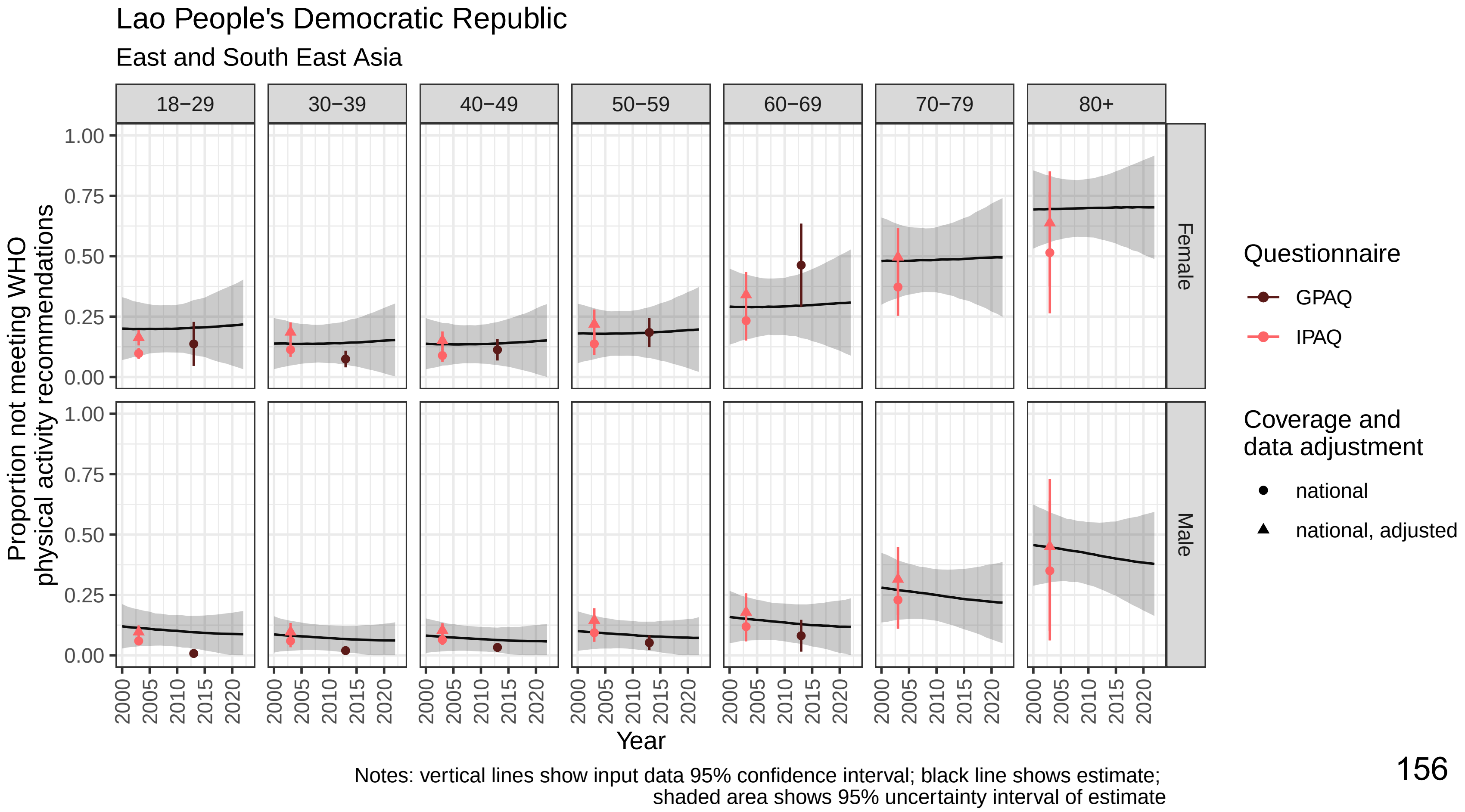

Lao People's Democratic Republic
East and South East Asia
18−29
30−39
40−49
50−59
60−69
70−79
80+
Female
Male
1.00
0.75
0.50
0.25
0.00
Proportion not meeting WHO physical activity recommendations
2000
2005
2010
2015
2020
Year
Questionnaire
GPAQ
IPAQ
Coverage and data adjustment
national
national, adjusted
Notes: vertical lines show input data 95% confidence interval; black line shows estimate; shaded area shows 95% uncertainty interval of estimate

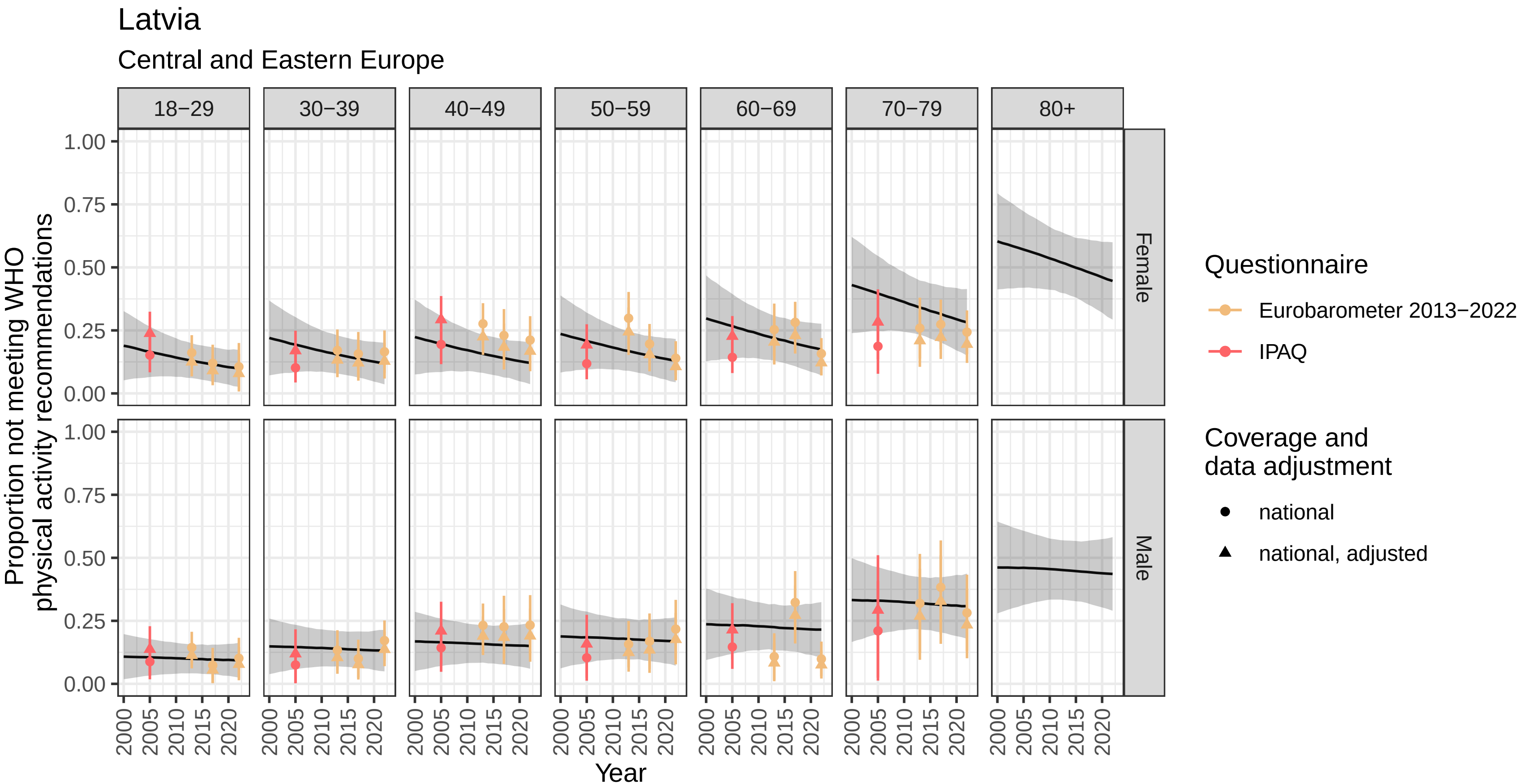

Notes: vertical lines show input data 95% confidence interval; black line shows estimate; shaded area shows 95% uncertainty interval of estimate

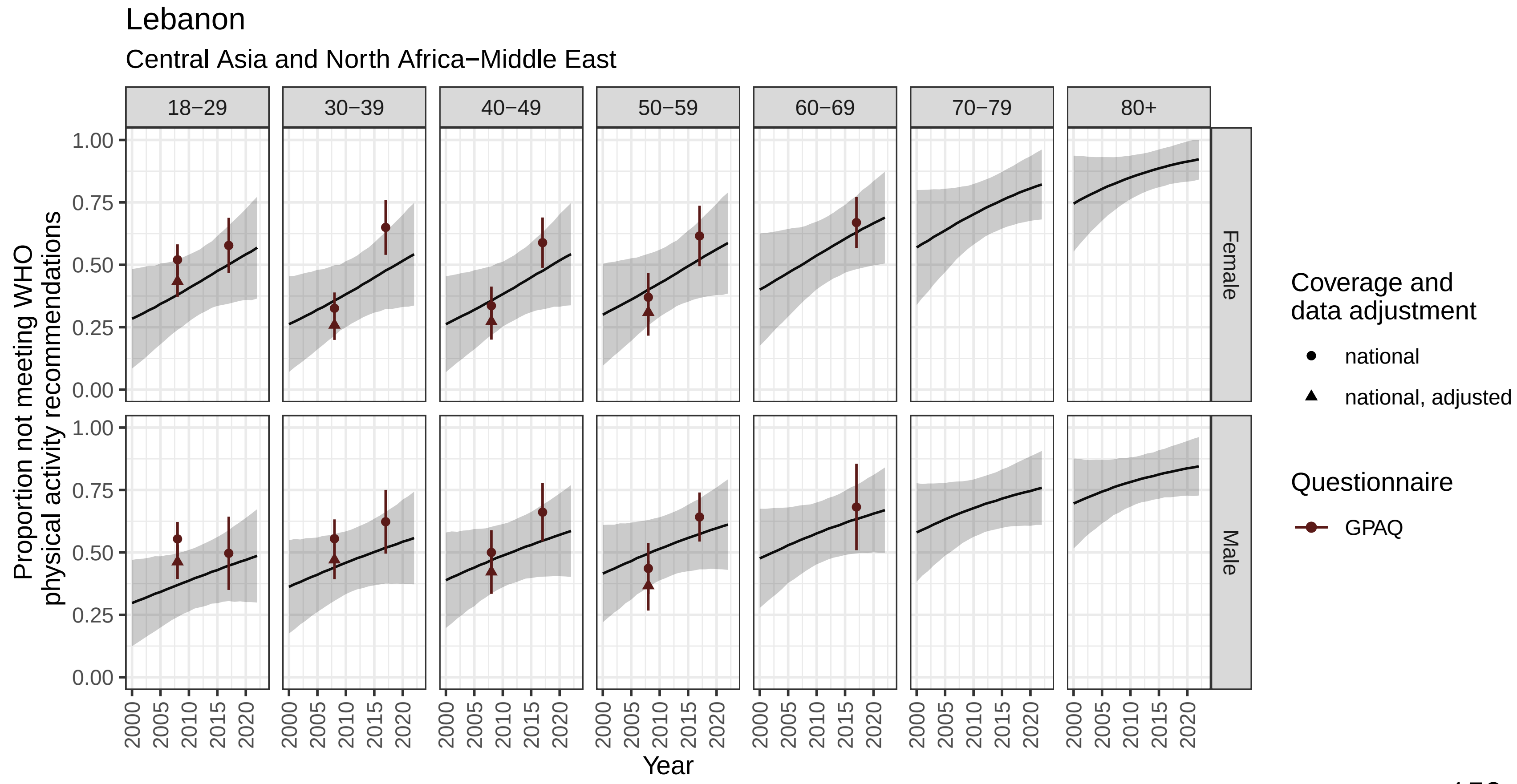

Notes: vertical lines show input data 95% confidence interval; black line shows estimate; shaded area shows 95% uncertainty interval of estimate

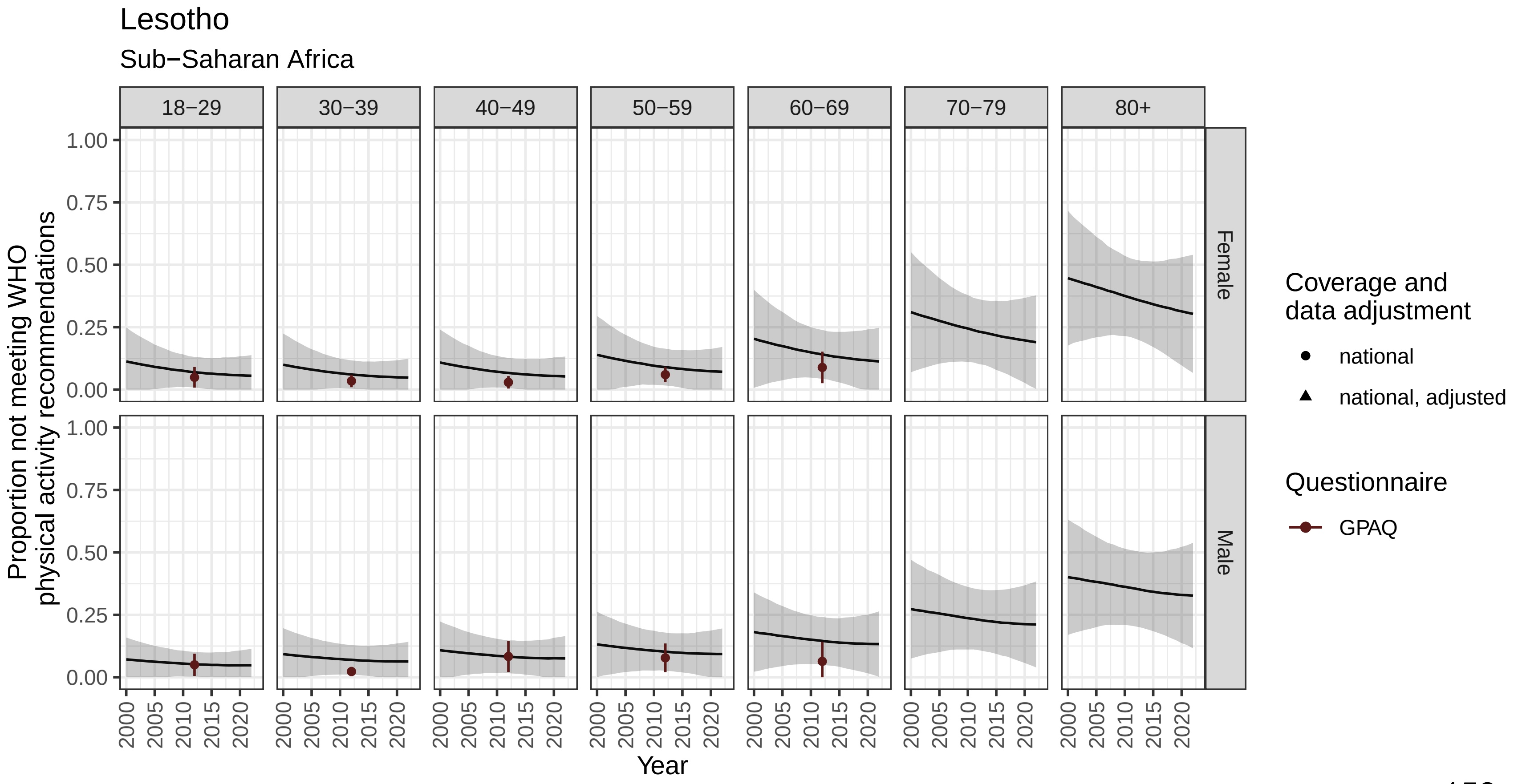

Notes: vertical lines show input data 95% confidence interval; black line shows estimate; shaded area shows 95% uncertainty interval of estimate

Liberia

Sub−Saharan Africa

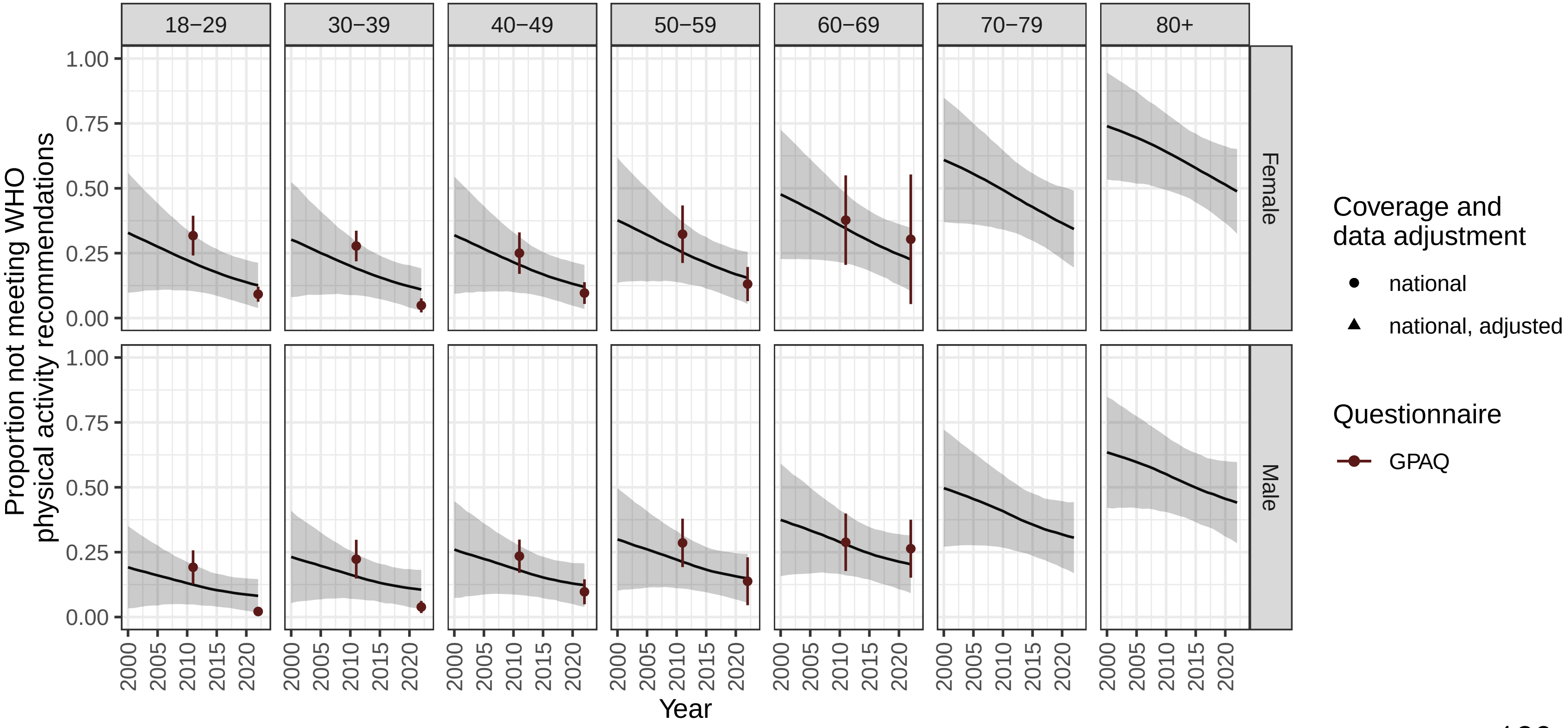

Notes: vertical lines show input data 95% confidence interval; black line shows estimate; shaded area shows 95% uncertainty interval of estimate

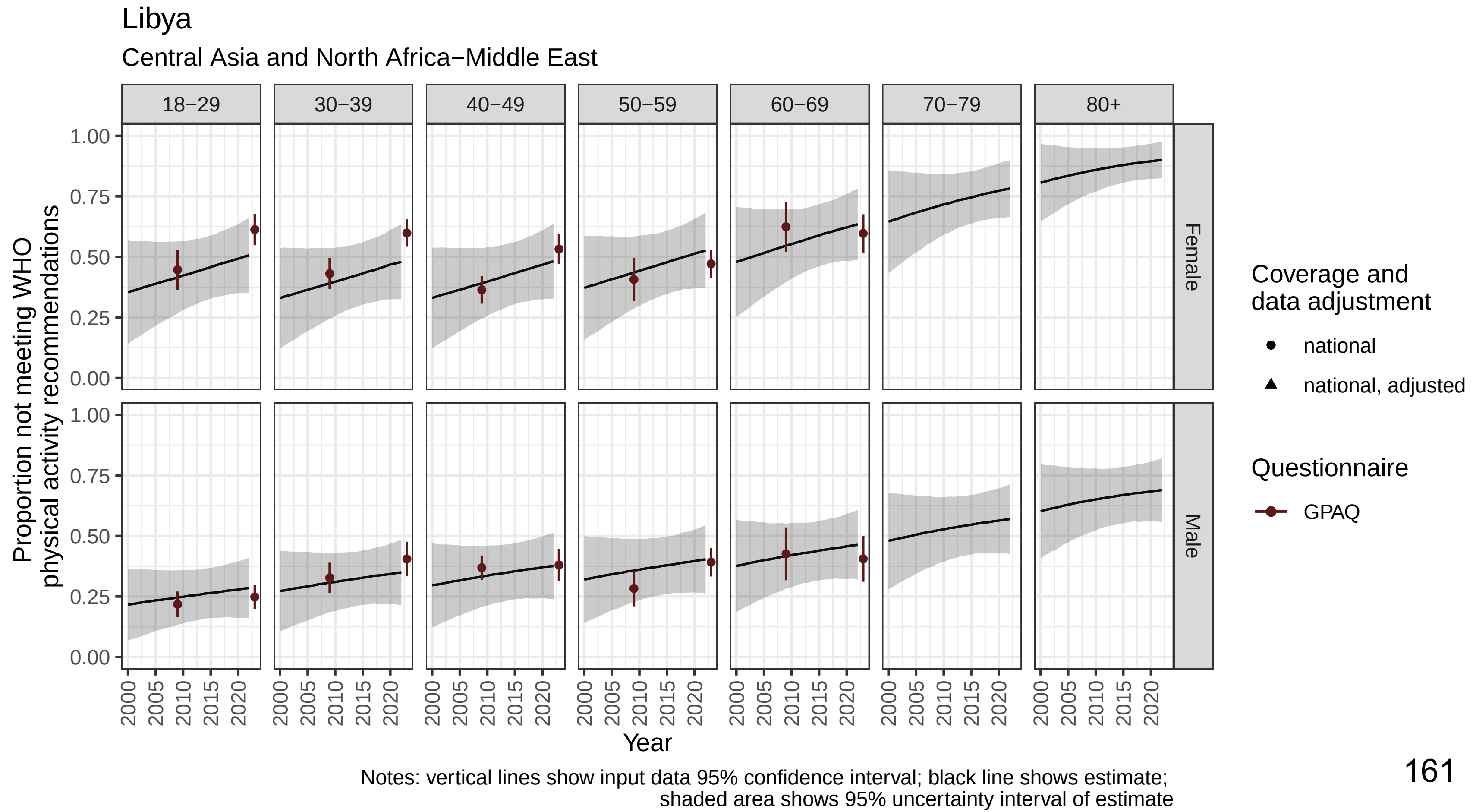
Libya
Central Asia and North Africa−Middle East
18−29
30−39
40−49
50−59
60−69
70−79
80+
Female
Male
Proportion not meeting WHO physical activity recommendations
0.00
0.25
0.50
0.75
1.00
2000
2005
2010
2015
2020
Year
Coverage and data adjustment
national
national, adjusted
Questionnaire
GPAQ
Notes: vertical lines show input data 95% confidence interval; black line shows estimate; shaded area shows 95% uncertainty interval of estimate

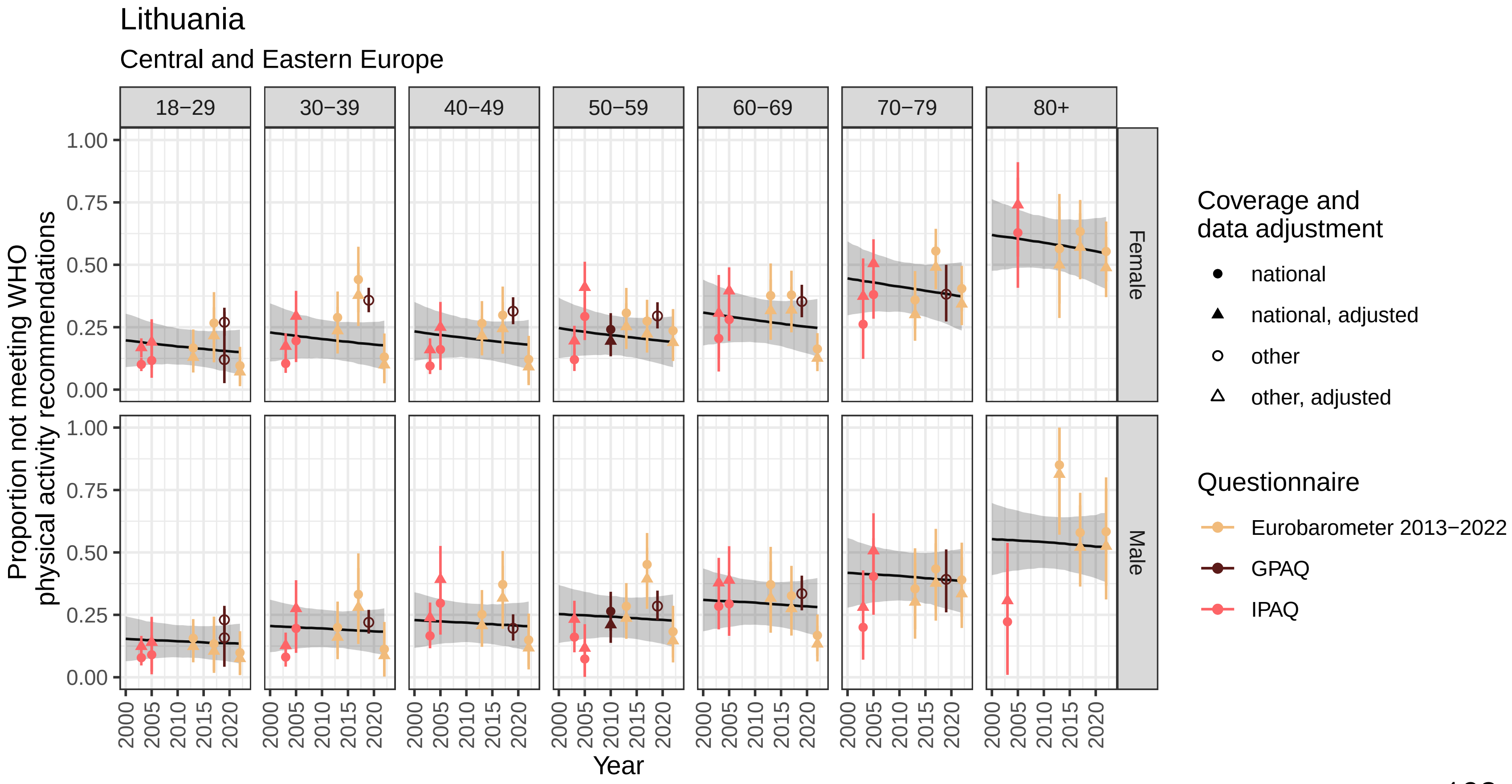

Notes: vertical lines show input data 95% confidence interval; black line shows estimate; shaded area shows 95% uncertainty interval of estimate

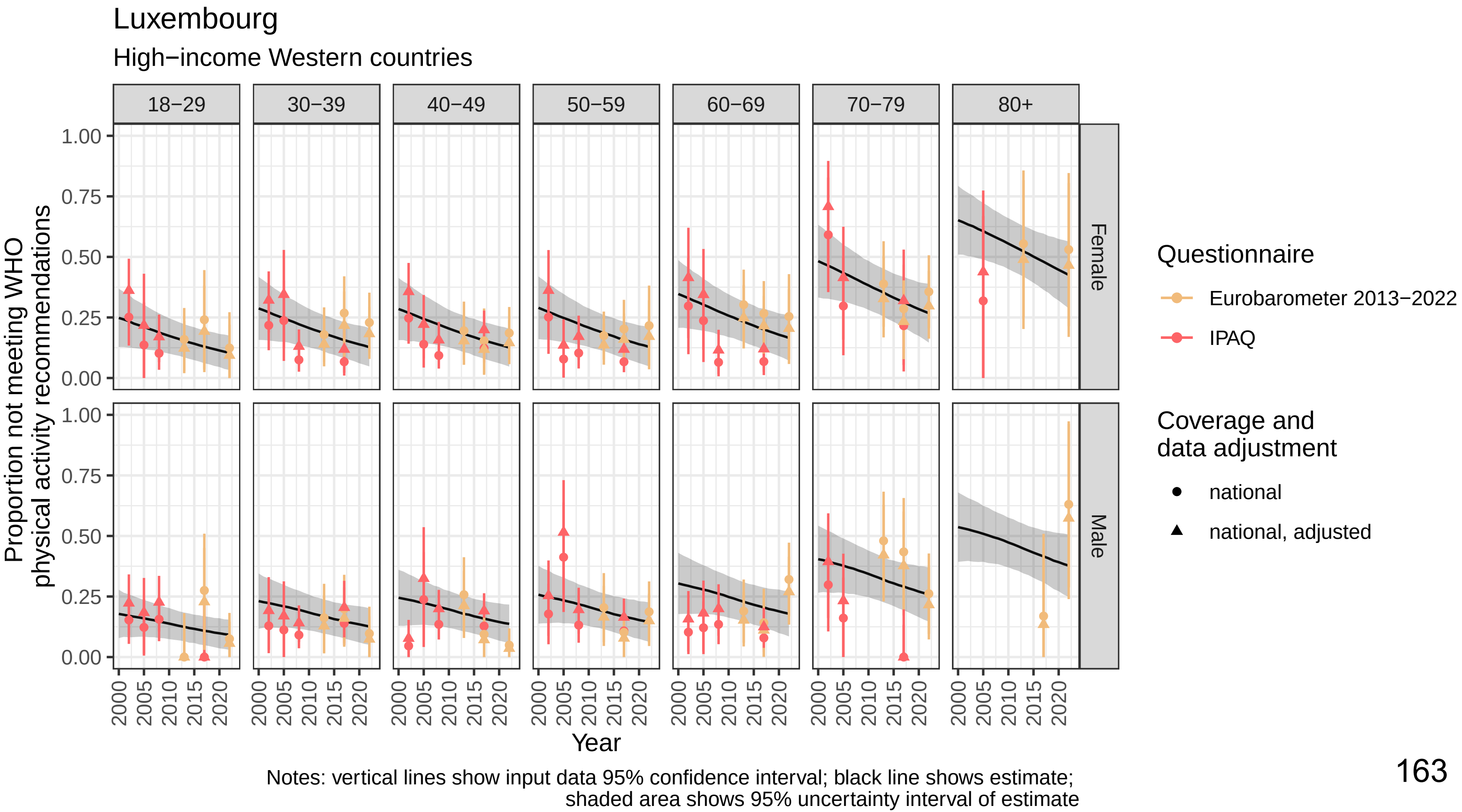

Luxembourg
High−income Western countries
18−29
30−39
40−49
50−59
60−69
70−79
80+
Female
Male
Proportion not meeting WHO physical activity recommendations
1.00
0.75
0.50
0.25
0.00
2000
2005
2010
2015
2020
Year
Questionnaire
Eurobarometer 2013−2022
IPAQ
Coverage and data adjustment
national
national, adjusted
Notes: vertical lines show input data 95% confidence interval; black line shows estimate;
shaded area shows 95% uncertainty interval of estimate

Madagascar

Sub−Saharan Africa

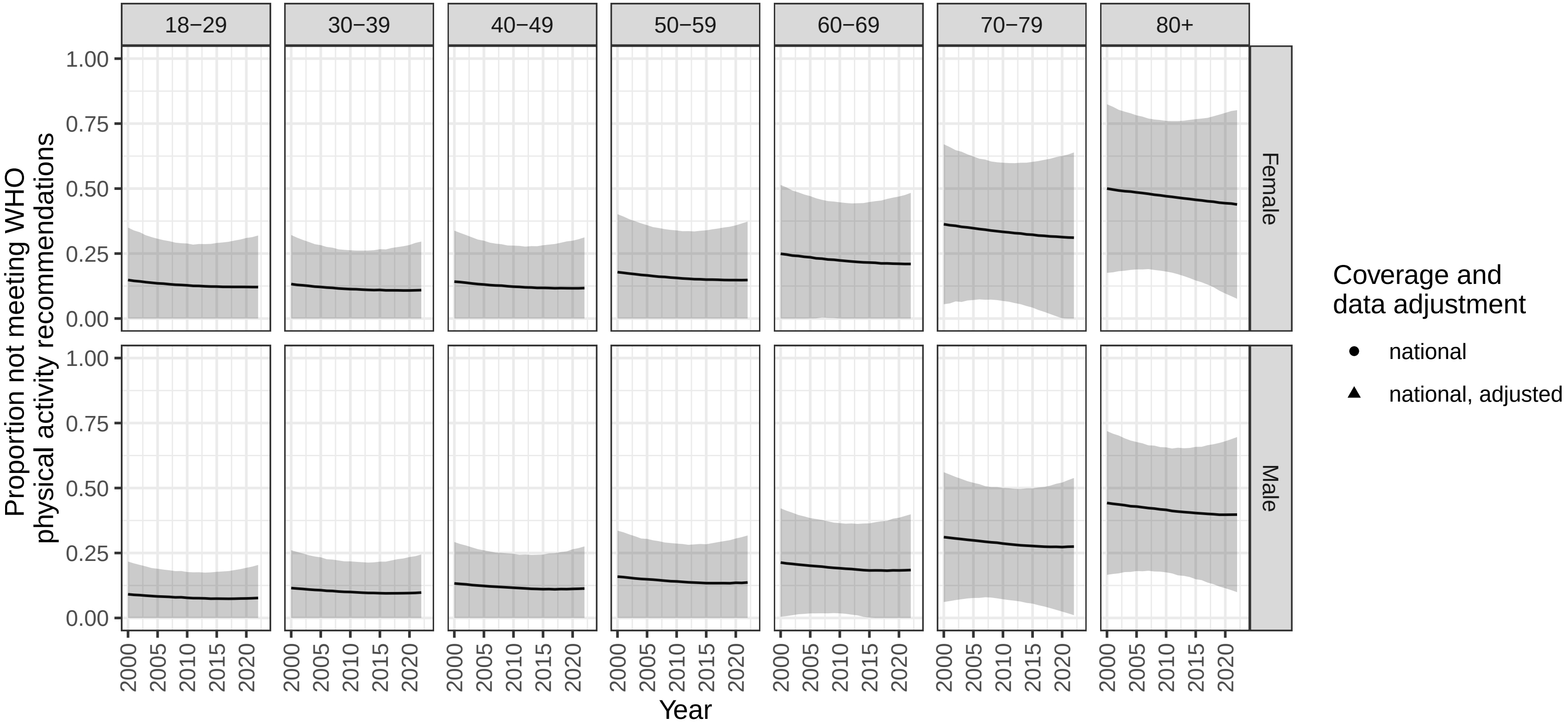

Notes: vertical lines show input data 95% confidence interval; black line shows estimate; shaded area shows 95% uncertainty interval of estimate

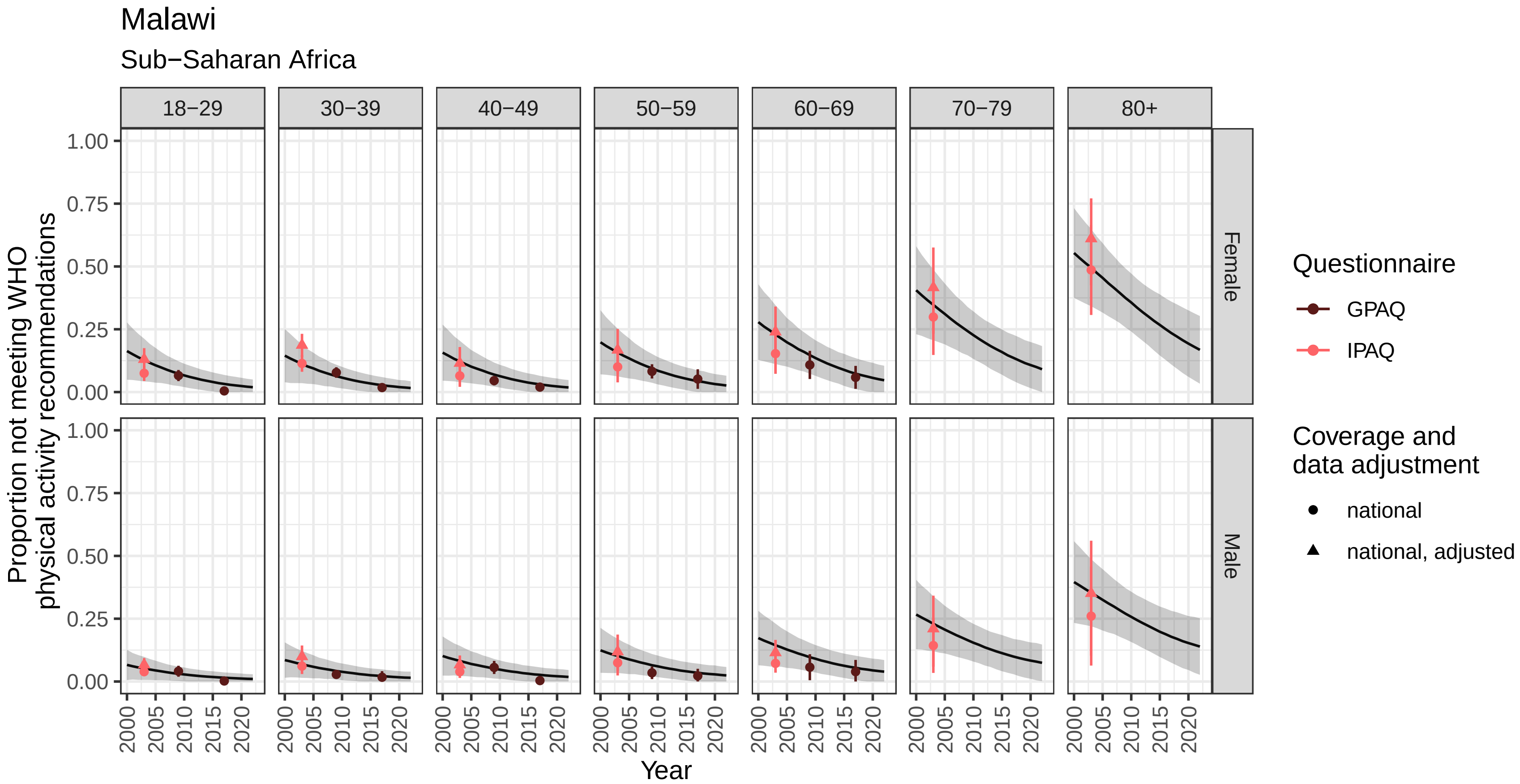

Notes: vertical lines show input data 95% confidence interval; black line shows estimate; shaded area shows 95% uncertainty interval of estimate

Malaysia

East and South East Asia

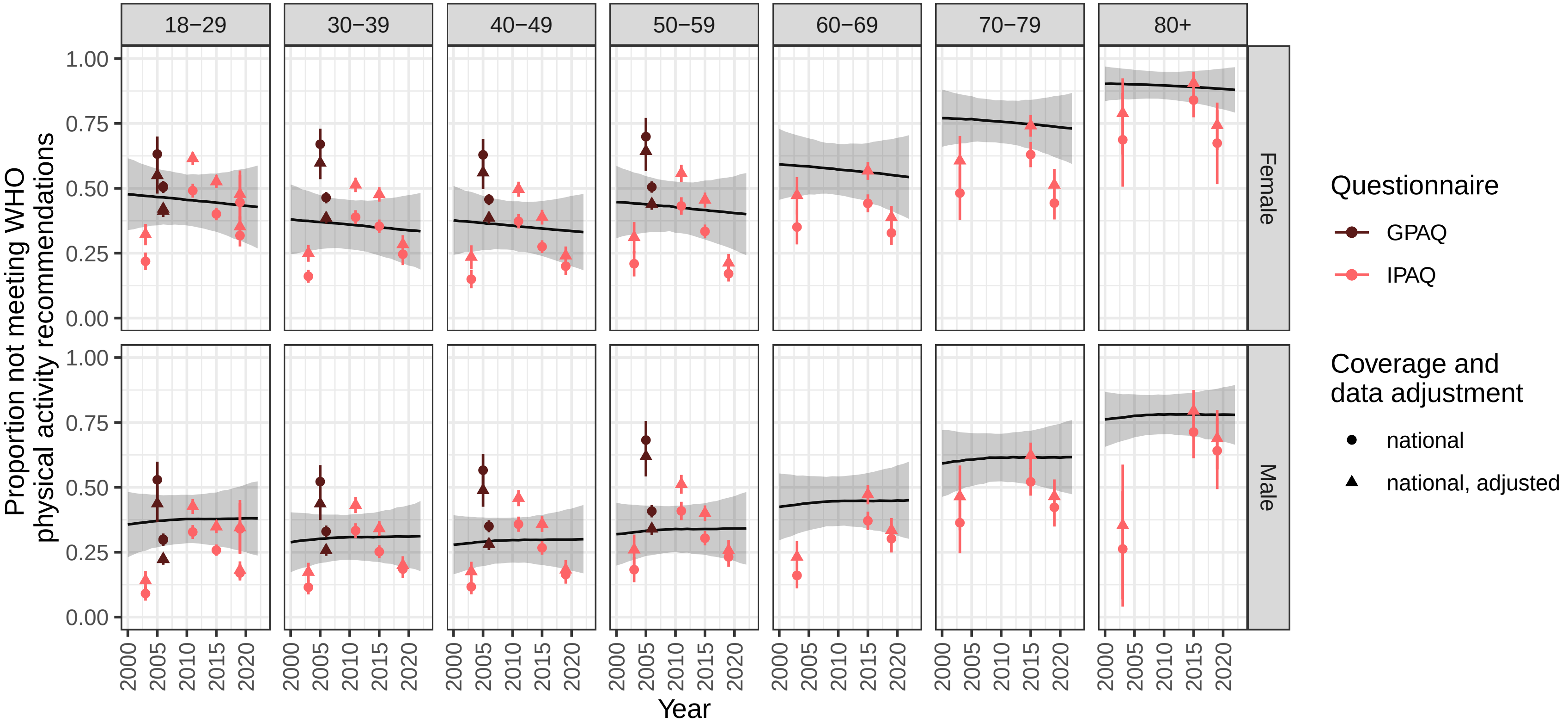

Notes: vertical lines show input data 95% confidence interval; black line shows estimate; shaded area shows 95% uncertainty interval of estimate

Maldives

East and South East Asia

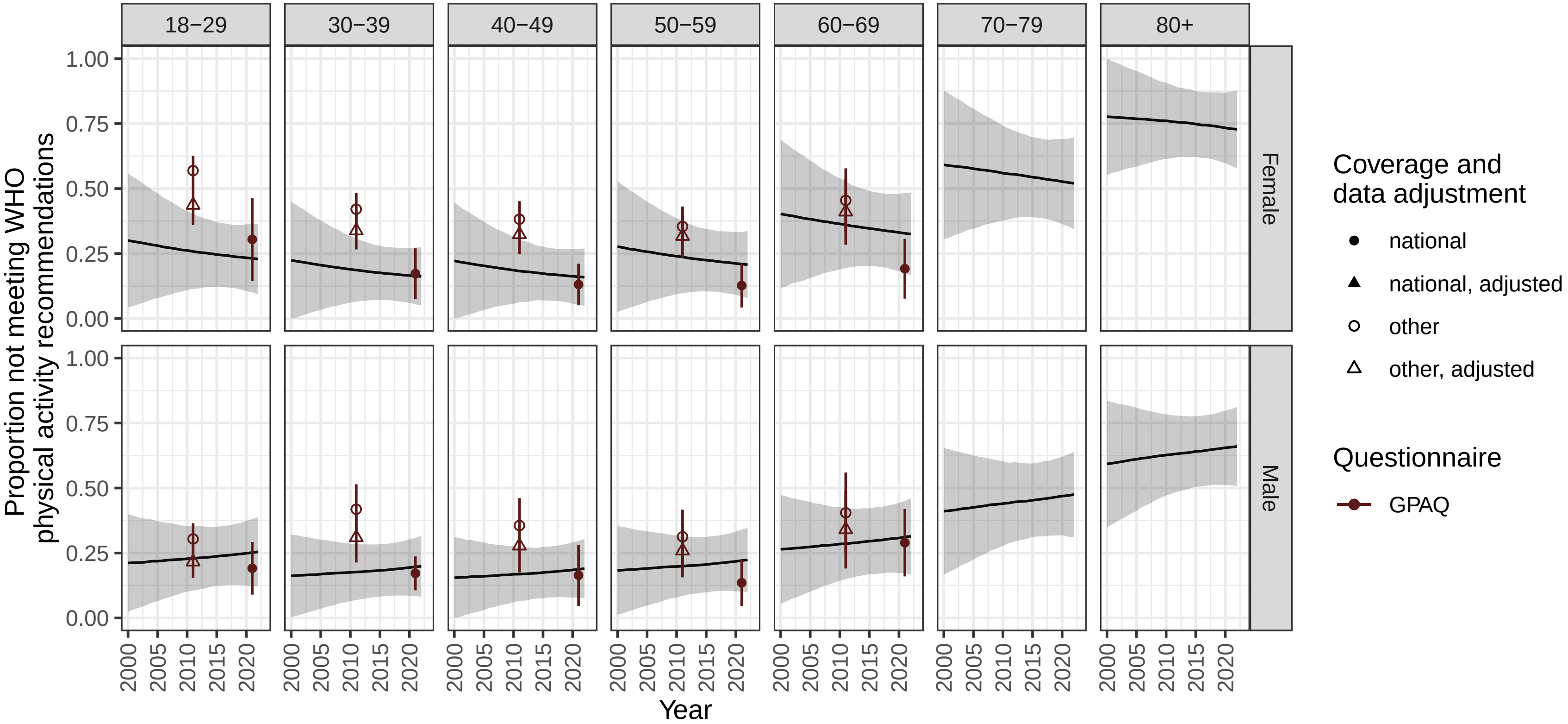

Notes: vertical lines show input data 95% confidence interval; black line shows estimate; shaded area shows 95% uncertainty interval of estimate

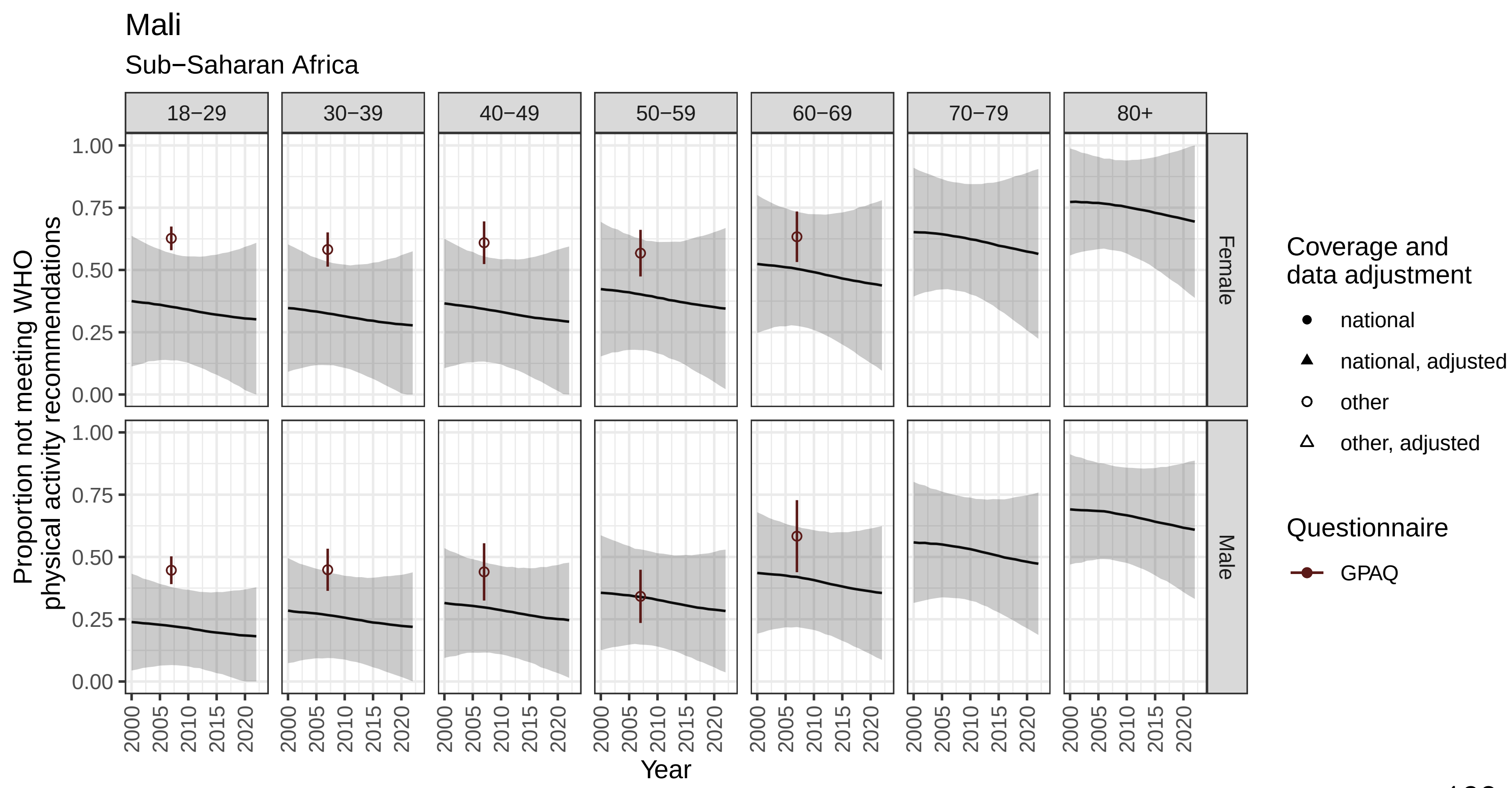

Notes: vertical lines show input data 95% confidence interval; black line shows estimate; shaded area shows 95% uncertainty interval of estimate

Malta

High−income Western countries

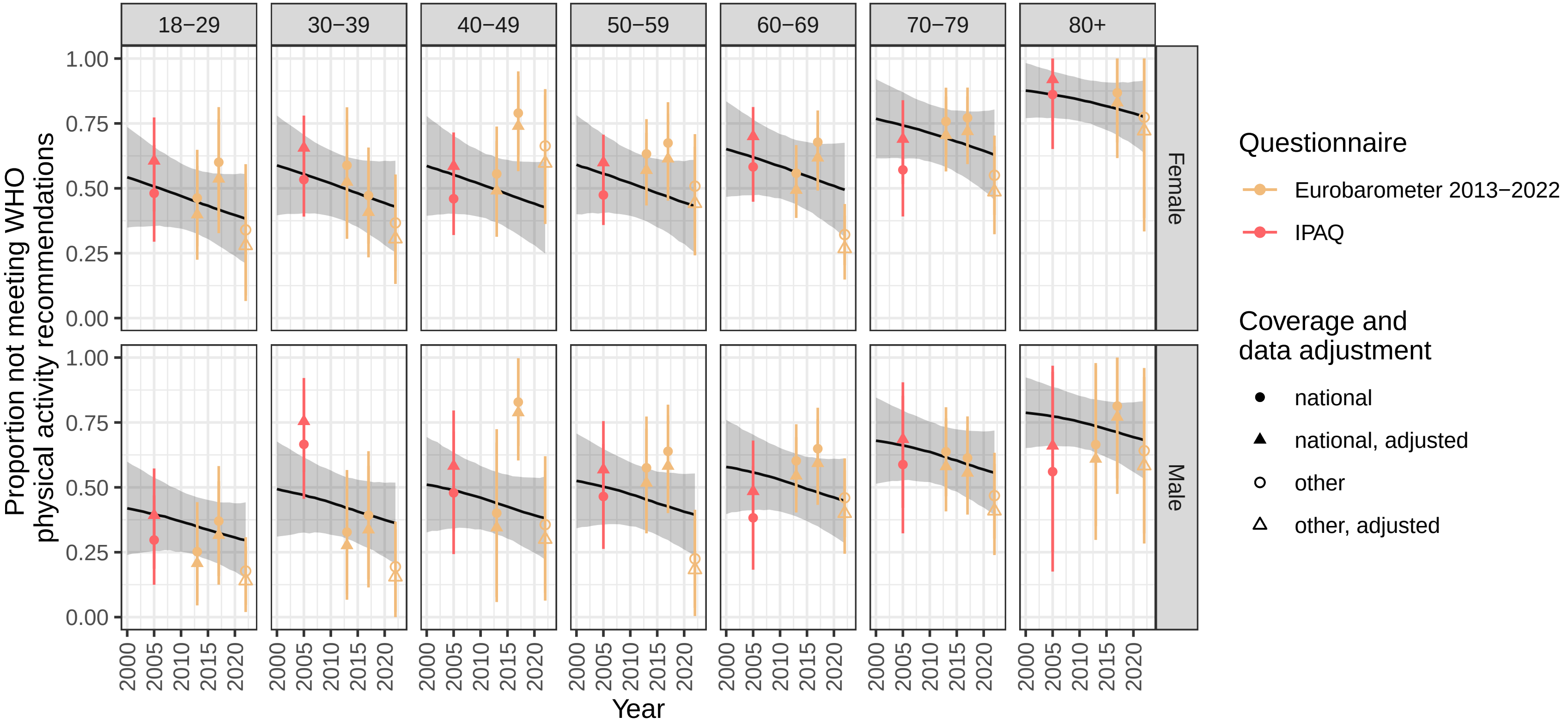

Notes: vertical lines show input data 95% confidence interval; black line shows estimate; shaded area shows 95% uncertainty interval of estimate

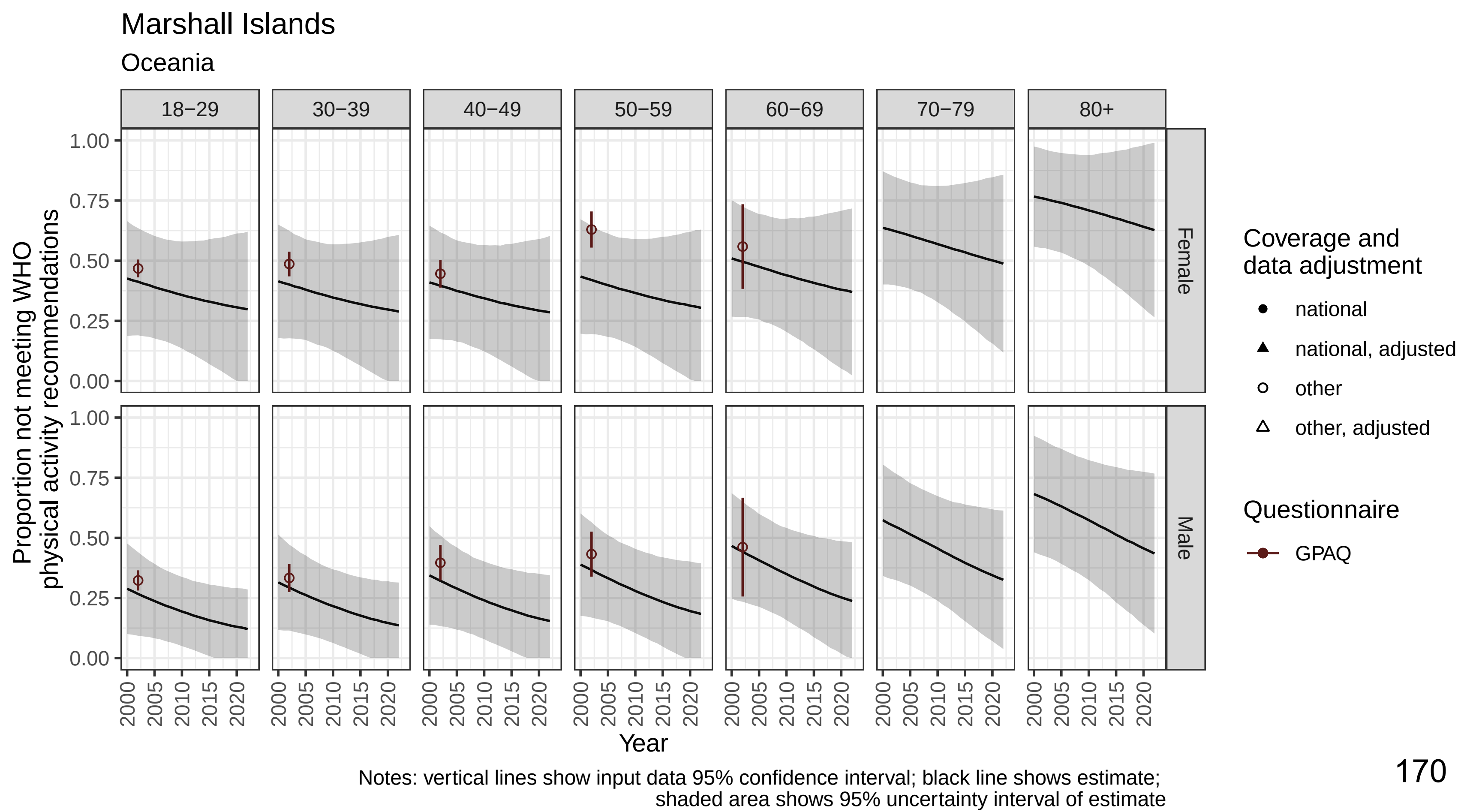

Marshall Islands
Oceania
18−29
30−39
40−49
50−59
60−69
70−79
80+
Female
Male
Proportion not meeting WHO physical activity recommendations
1.00
0.75
0.50
0.25
0.00
2000
2005
2010
2015
2020
Year
Coverage and data adjustment
national
national, adjusted
other
other, adjusted
Questionnaire
GPAQ
Notes: vertical lines show input data 95% confidence interval; black line shows estimate; shaded area shows 95% uncertainty interval of estimate

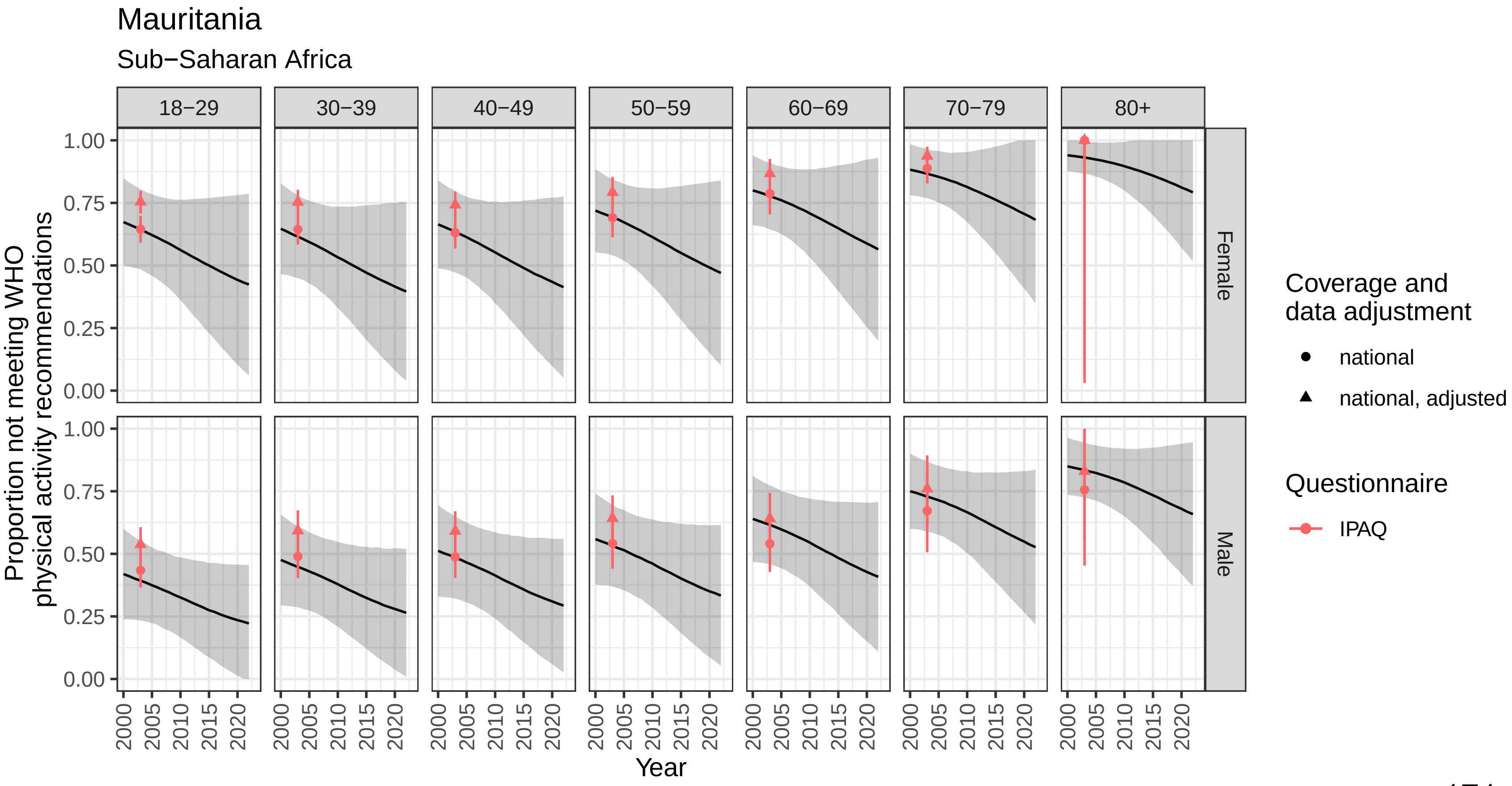

Notes: vertical lines show input data 95% confidence interval; black line shows estimate;
shaded area shows 95% uncertainty interval of estimate

Mauritius

Sub−Saharan Africa

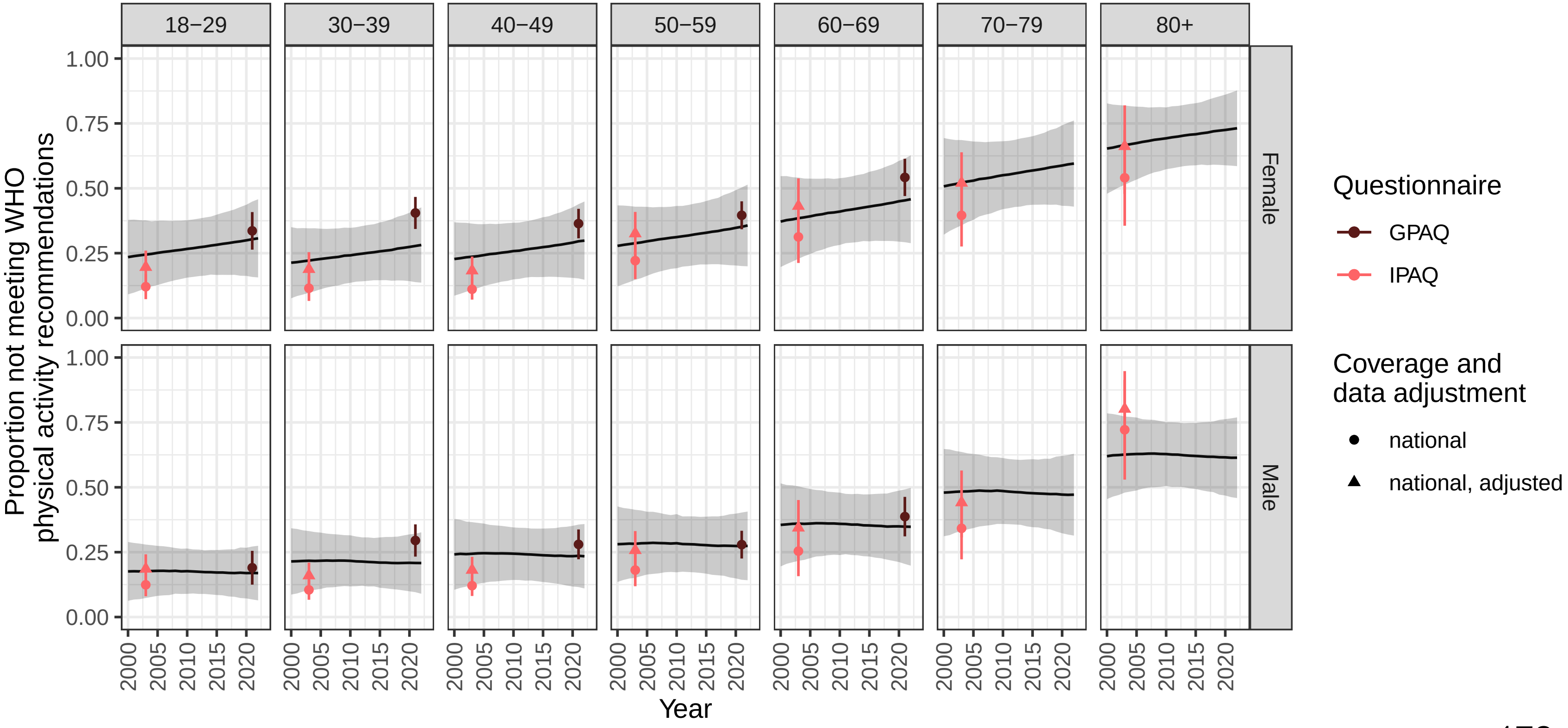

Notes: vertical lines show input data 95% confidence interval; black line shows estimate; shaded area shows 95% uncertainty interval of estimate

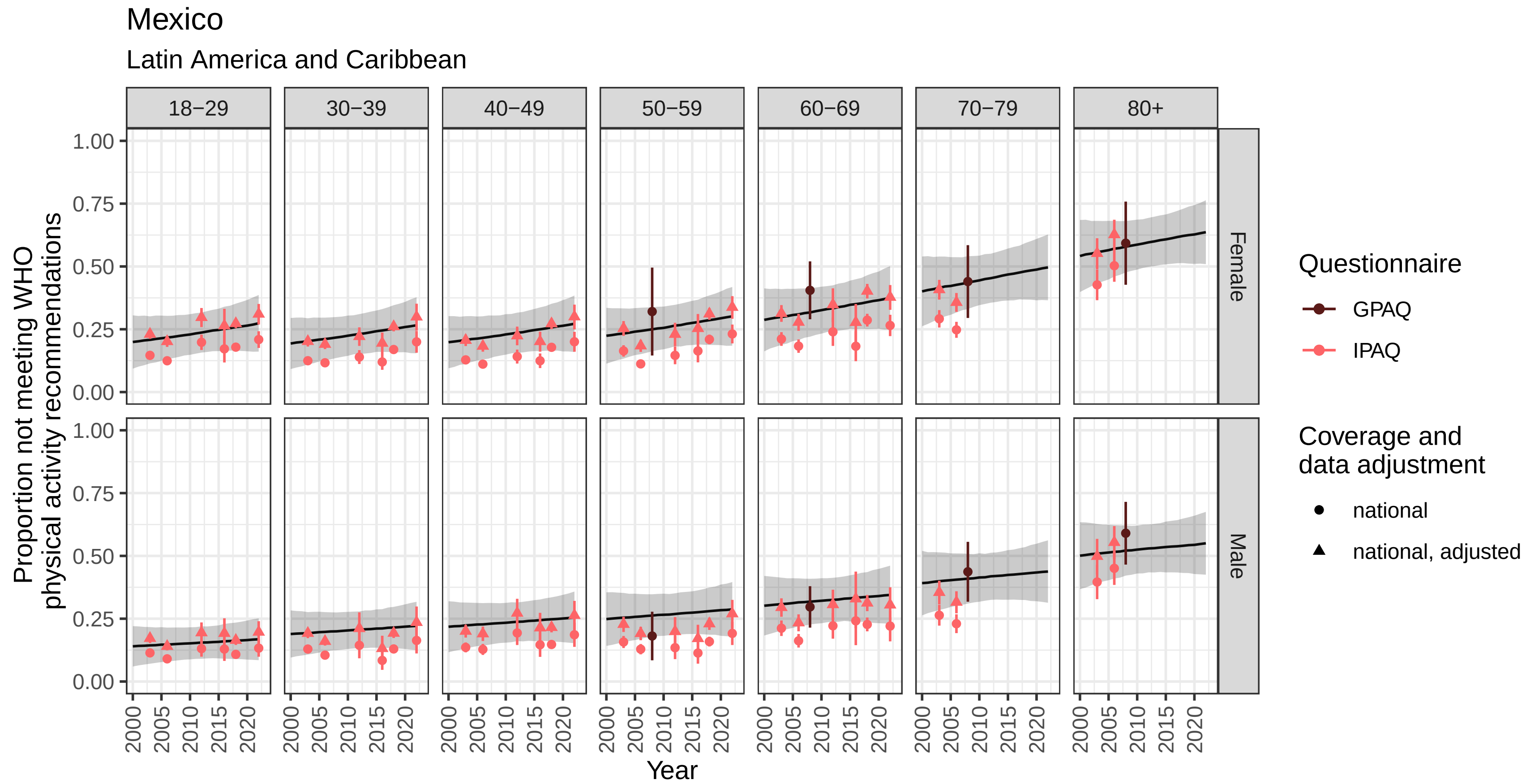

Notes: vertical lines show input data 95% confidence interval; black line shows estimate; shaded area shows 95% uncertainty interval of estimate

Micronesia (Federated States of)

Oceania

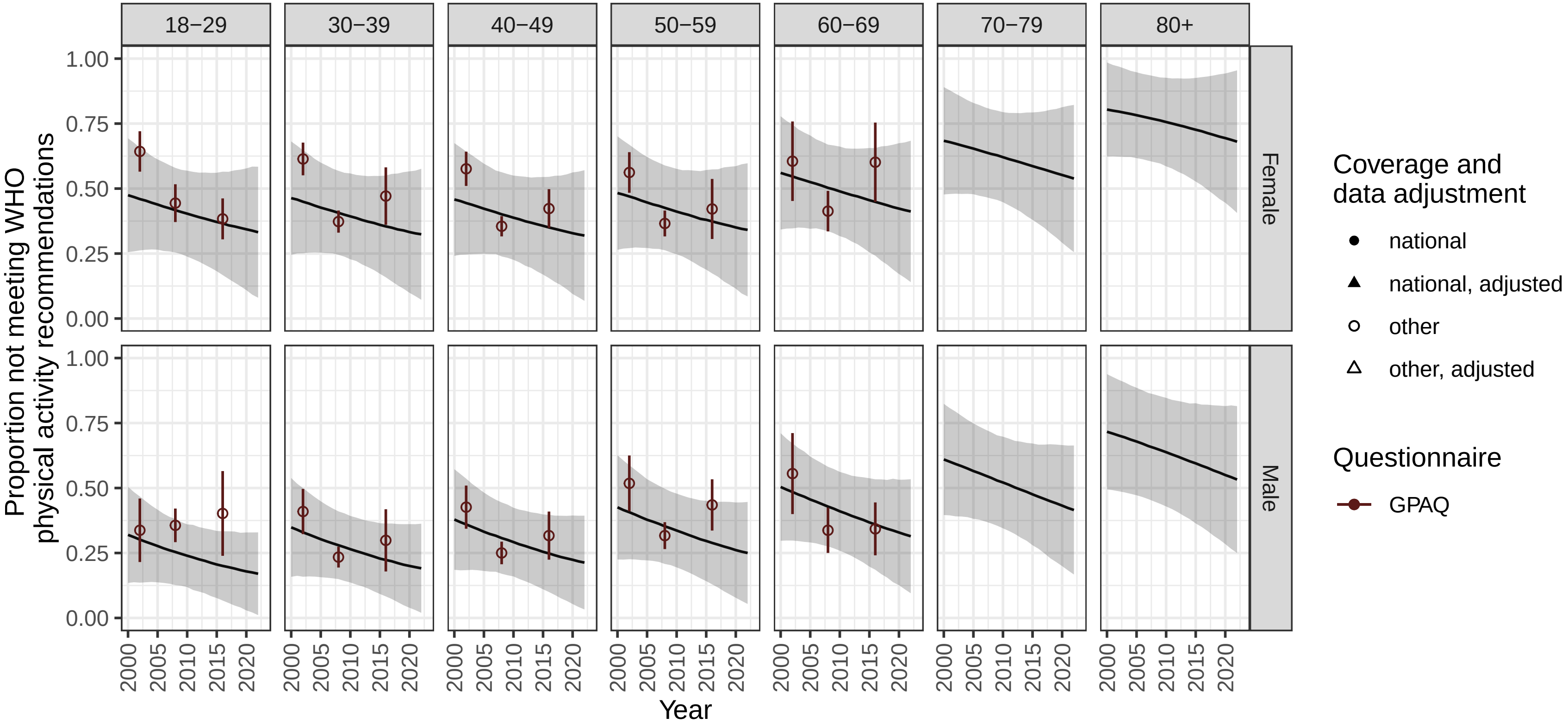

Notes: vertical lines show input data 95% confidence interval; black line shows estimate; shaded area shows 95% uncertainty interval of estimate

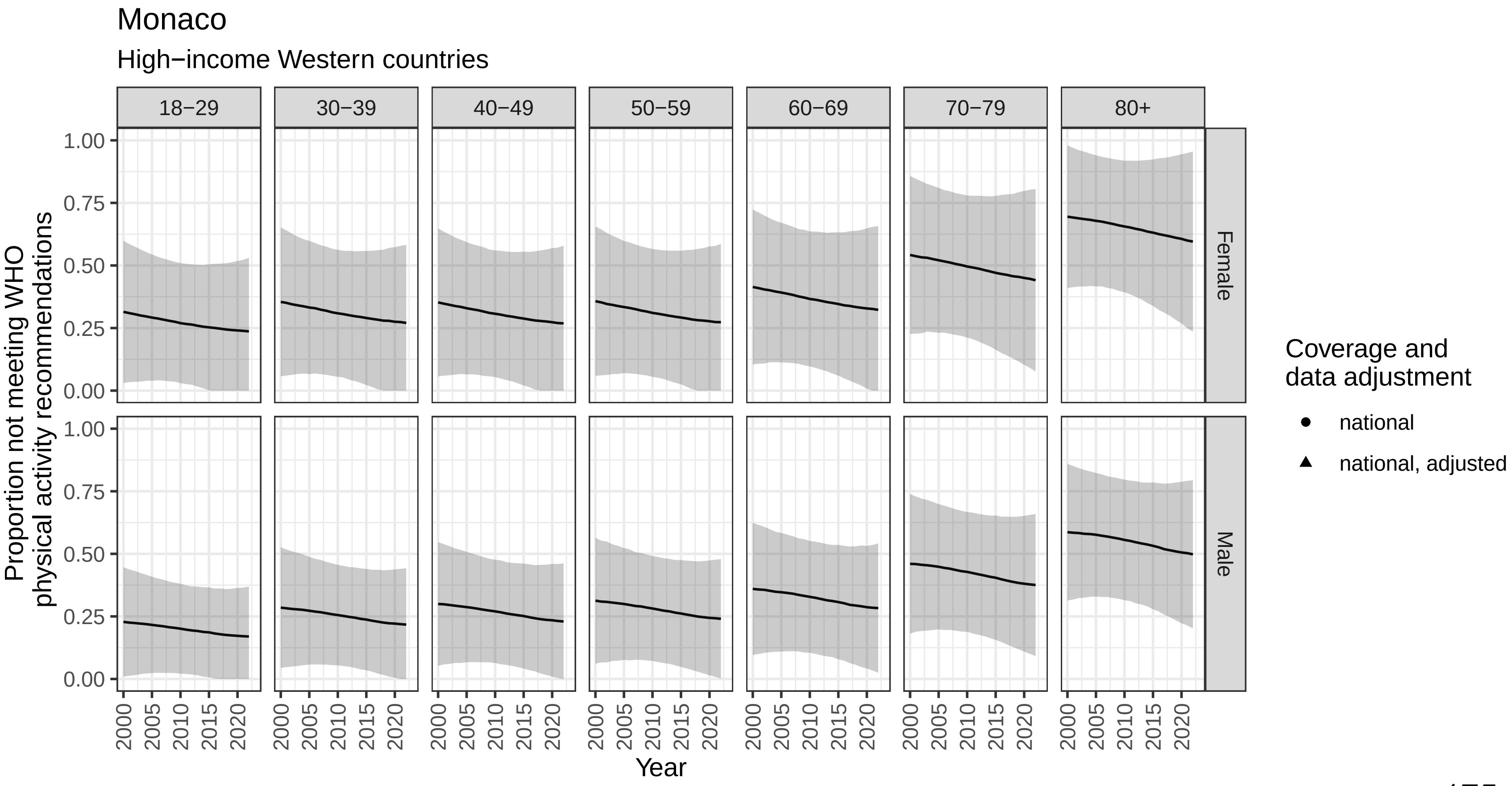

Notes: vertical lines show input data 95% confidence interval; black line shows estimate; shaded area shows 95% uncertainty interval of estimate

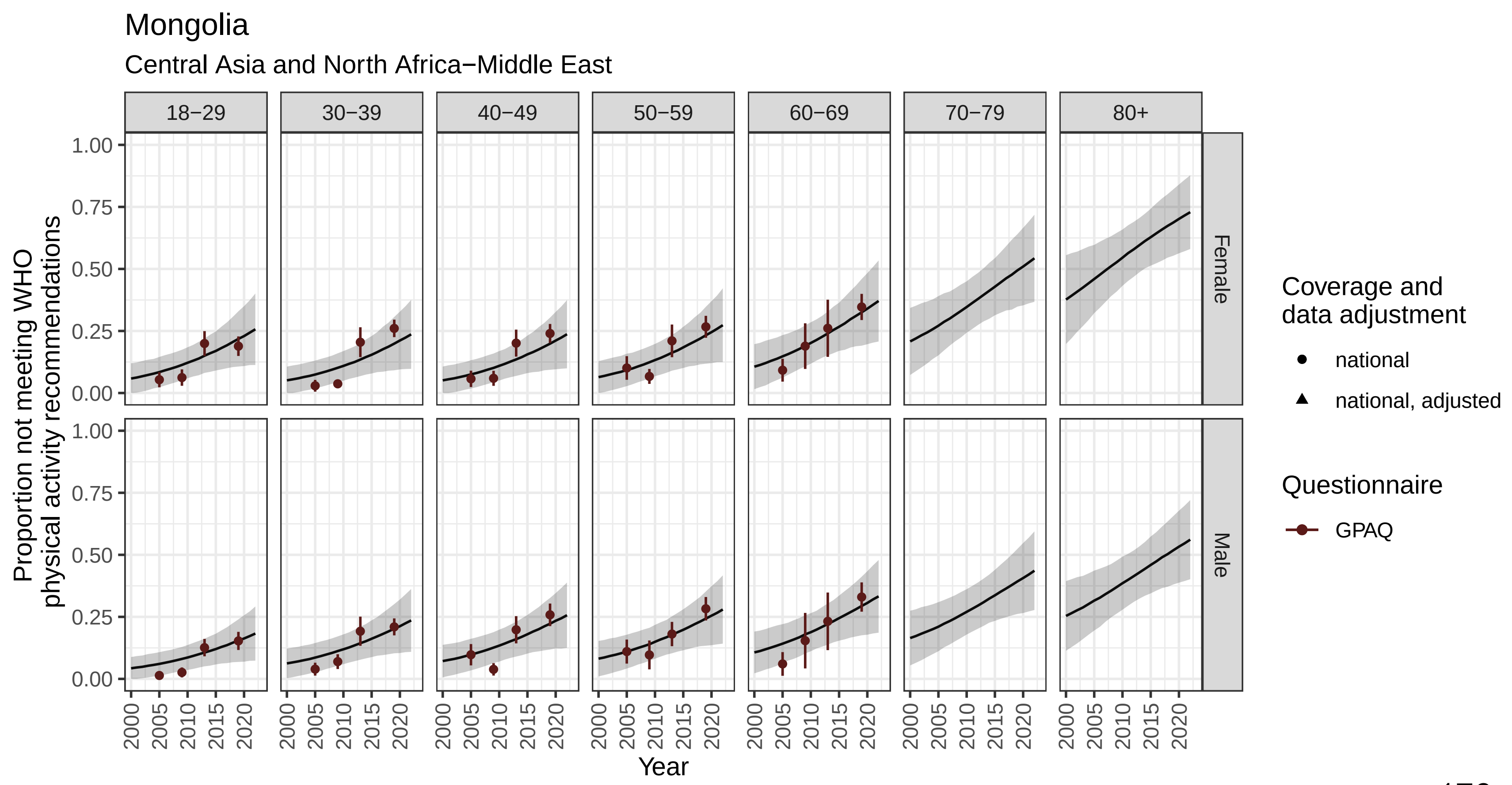

Notes: vertical lines show input data 95% confidence interval; black line shows estimate; shaded area shows 95% uncertainty interval of estimate

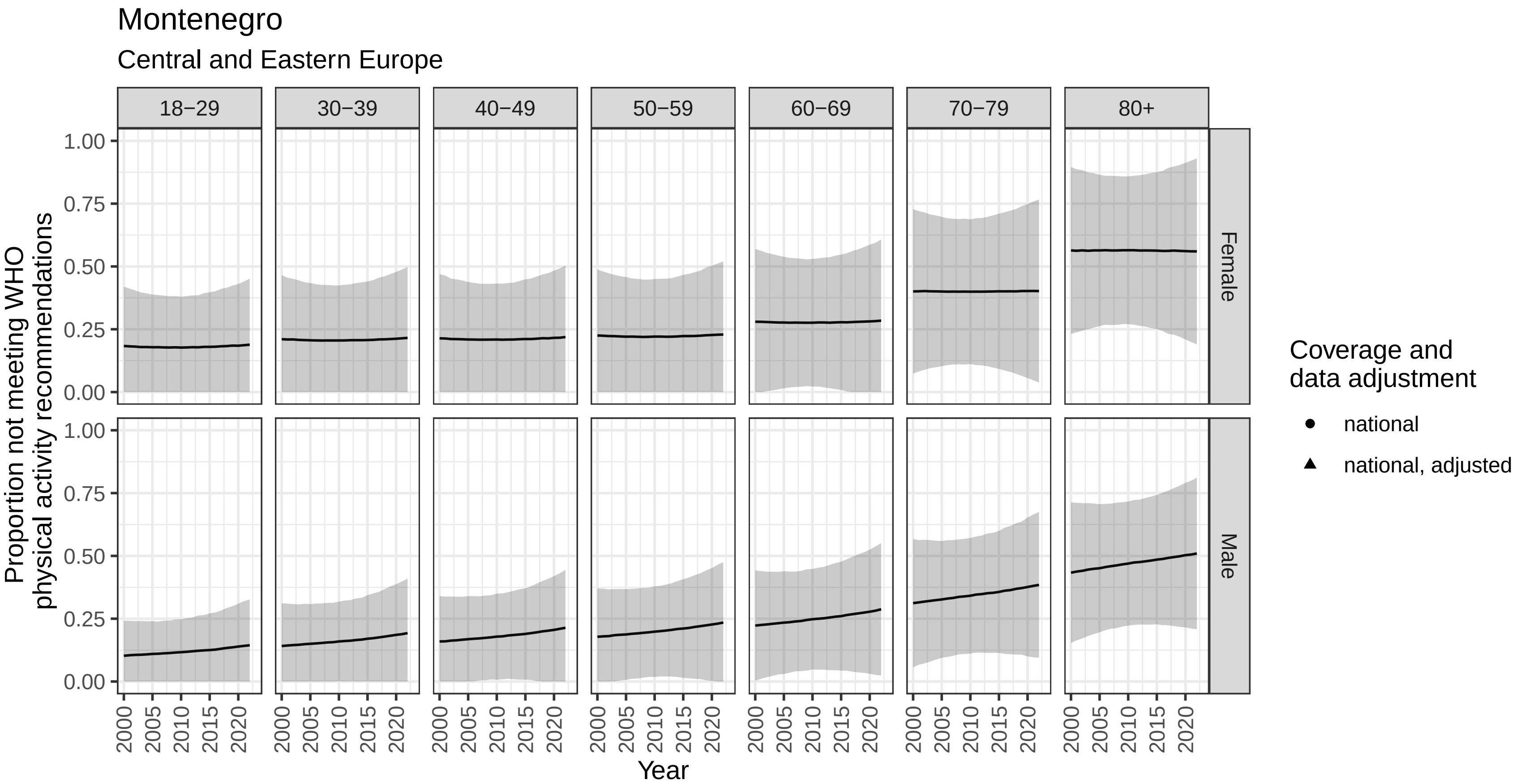

Notes: vertical lines show input data 95% confidence interval; black line shows estimate; shaded area shows 95% uncertainty interval of estimate

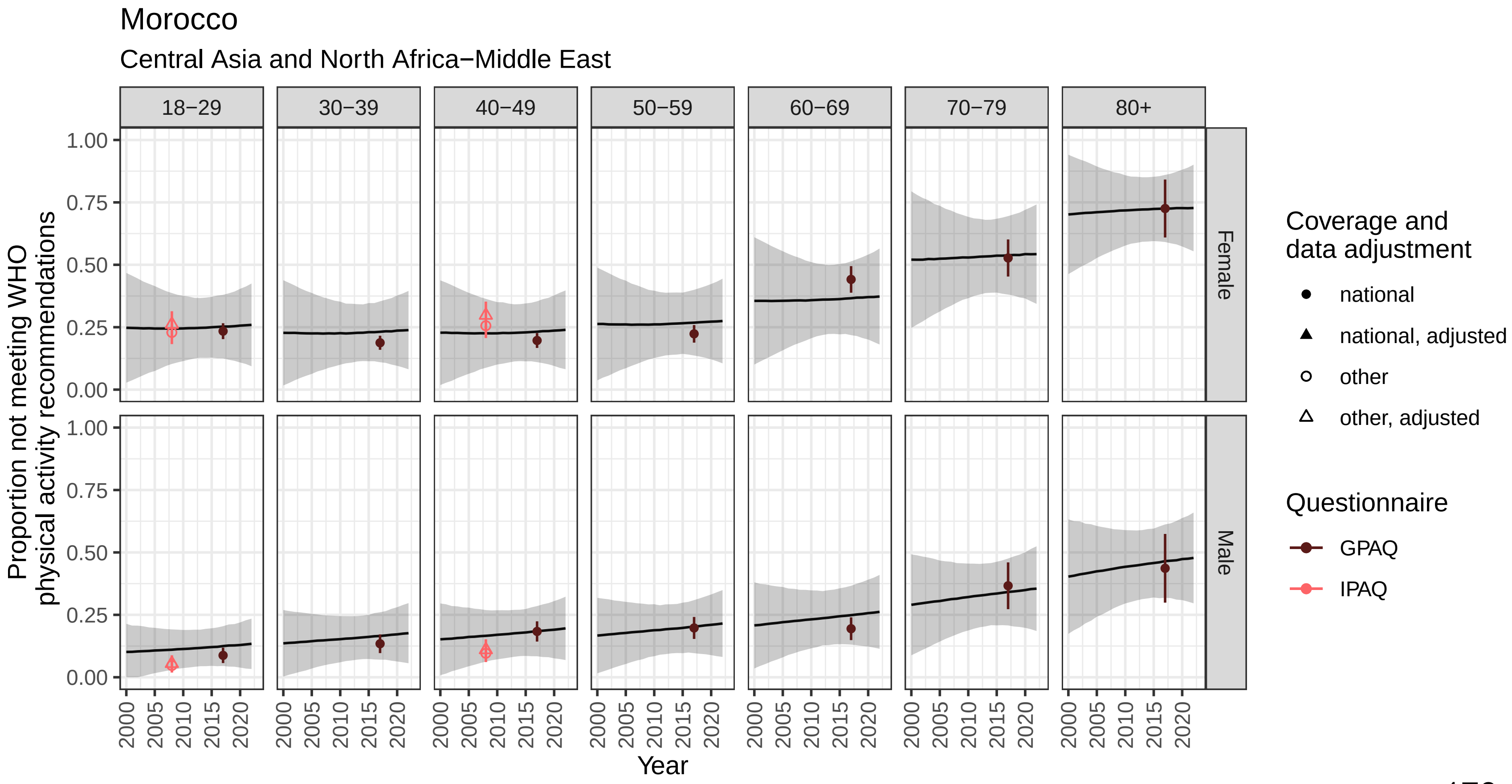

Notes: vertical lines show input data 95% confidence interval; black line shows estimate; shaded area shows 95% uncertainty interval of estimate

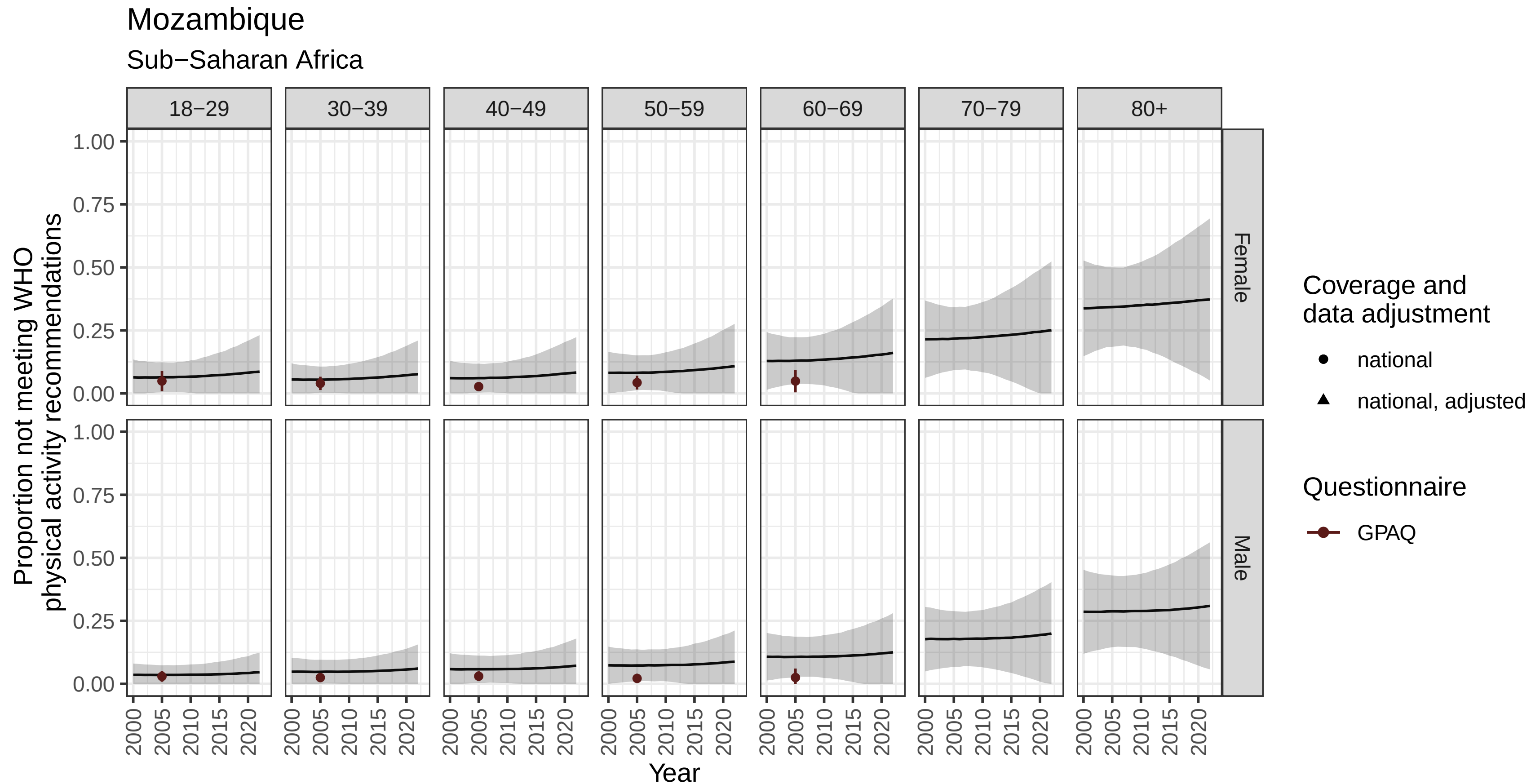

Notes: vertical lines show input data 95% confidence interval; black line shows estimate; shaded area shows 95% uncertainty interval of estimate

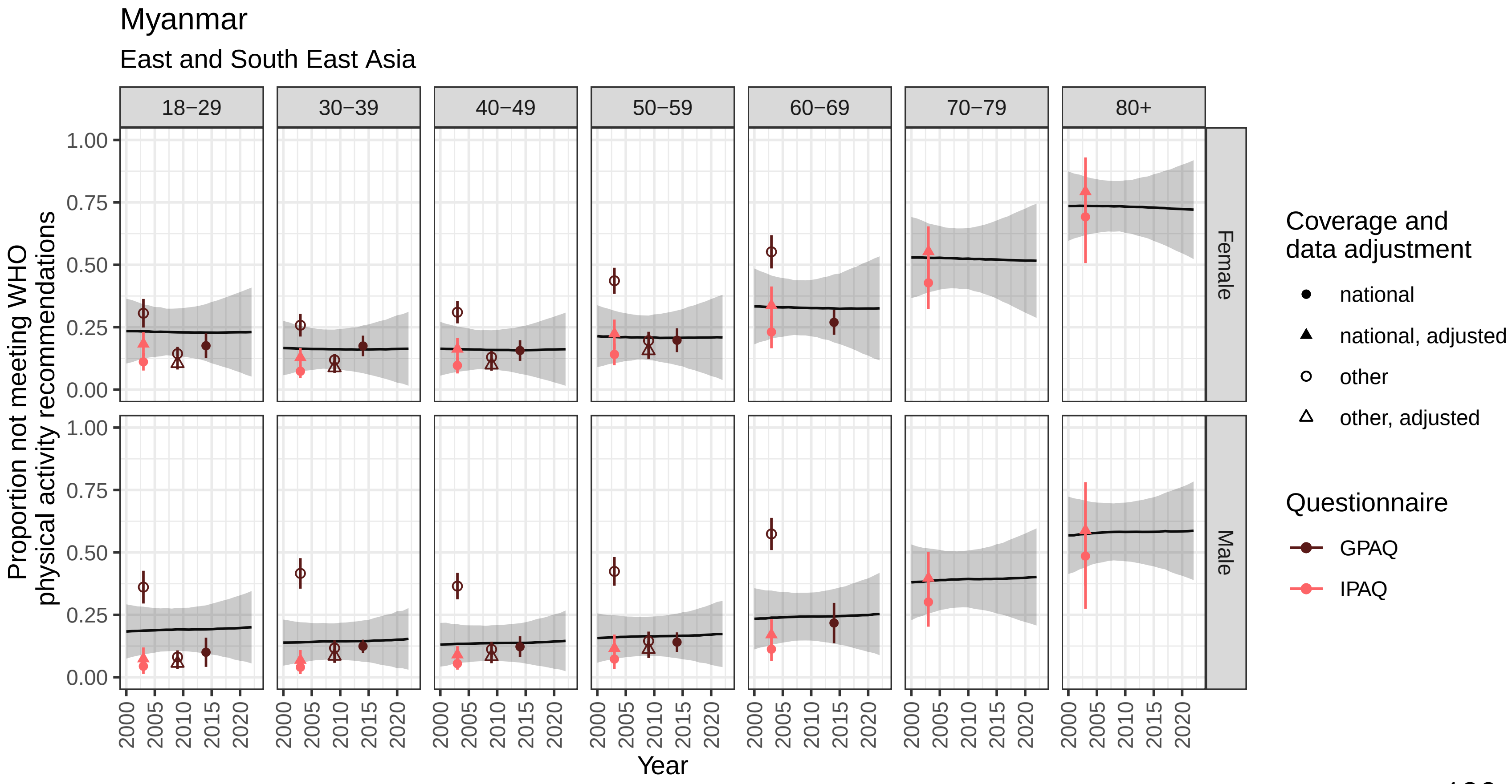

Notes: vertical lines show input data 95% confidence interval; black line shows estimate; shaded area shows 95% uncertainty interval of estimate

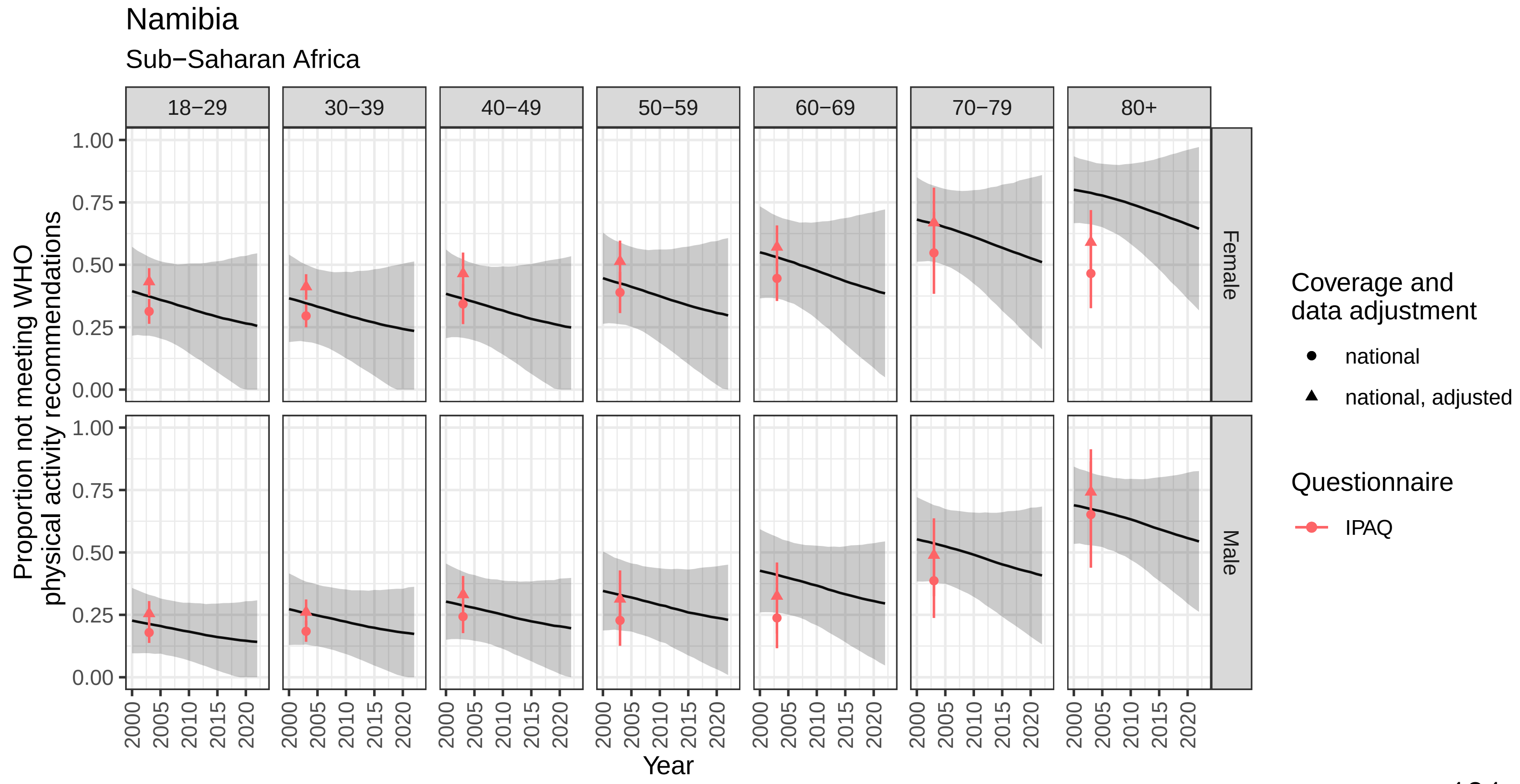

Notes: vertical lines show input data 95% confidence interval; black line shows estimate; shaded area shows 95% uncertainty interval of estimate

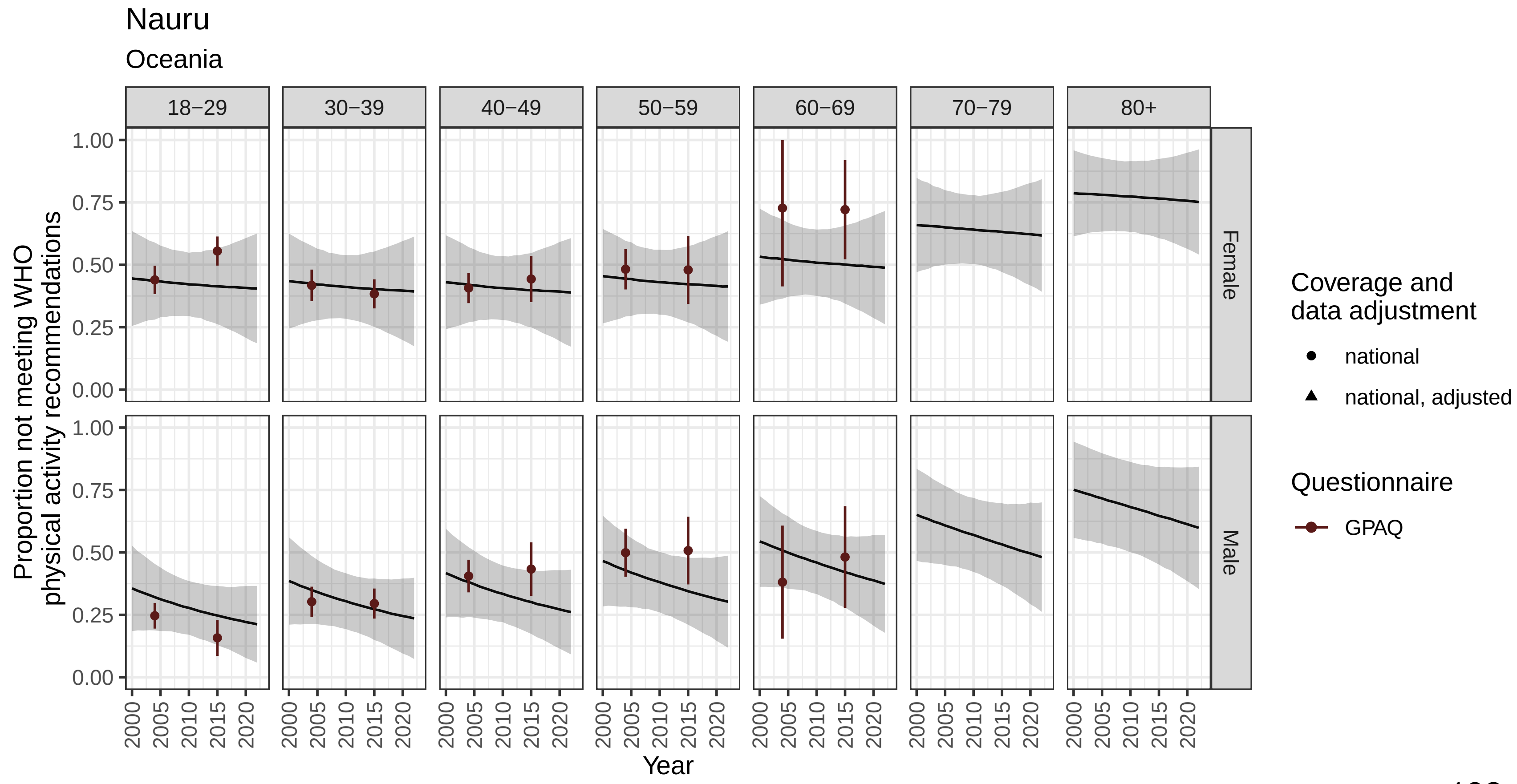

Notes: vertical lines show input data 95% confidence interval; black line shows estimate; shaded area shows 95% uncertainty interval of estimate

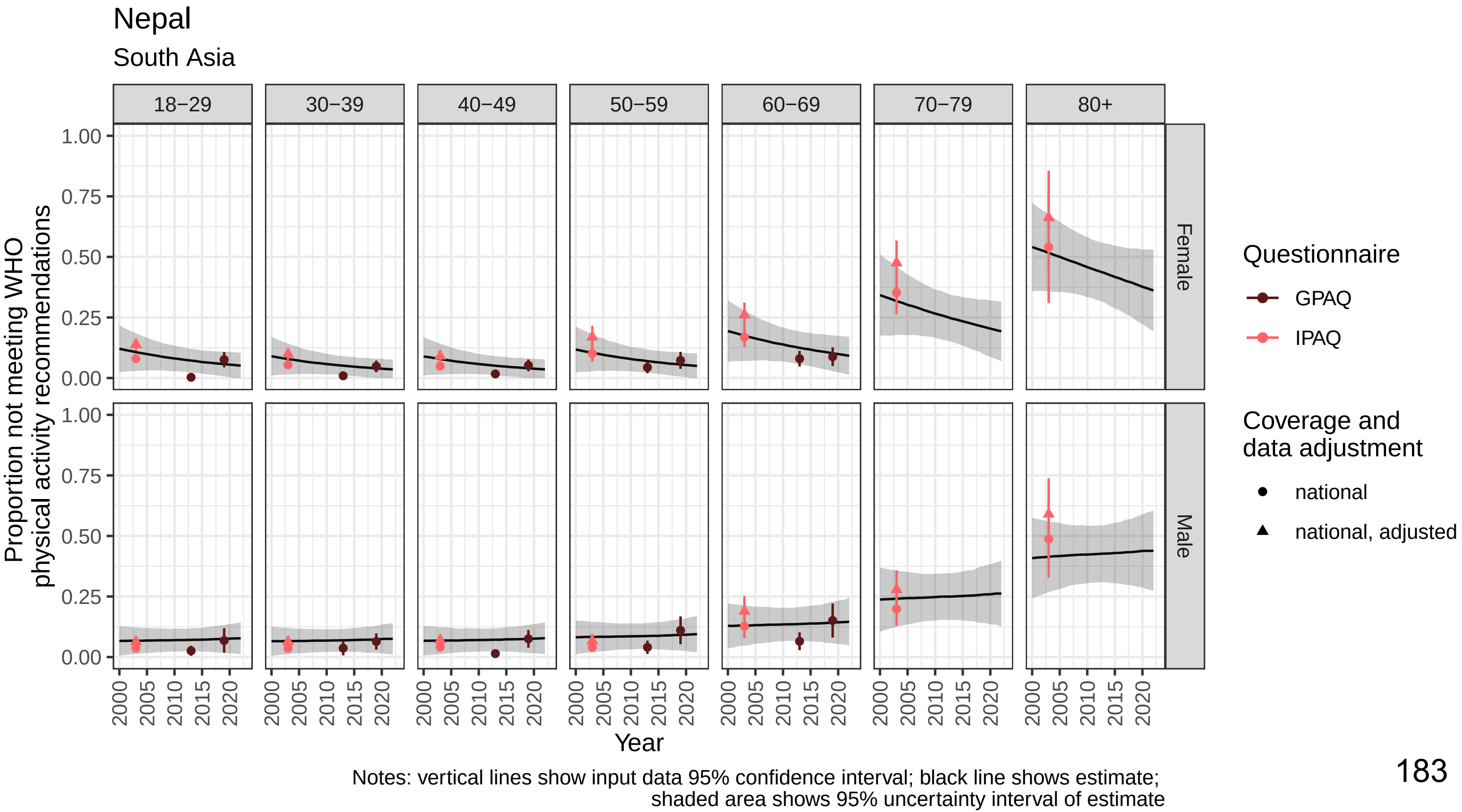

Nepal
South Asia
18−29
30−39
40−49
50−59
60−69
70−79
80+
Female
Male
1.00
0.75
0.50
0.25
0.00
2000
2005
2010
2015
2020
Proportion not meeting WHO physical activity recommendations
Year
Questionnaire
GPAQ
IPAQ
Coverage and data adjustment
national
national, adjusted
Notes: vertical lines show input data 95% confidence interval; black line shows estimate; shaded area shows 95% uncertainty interval of estimate

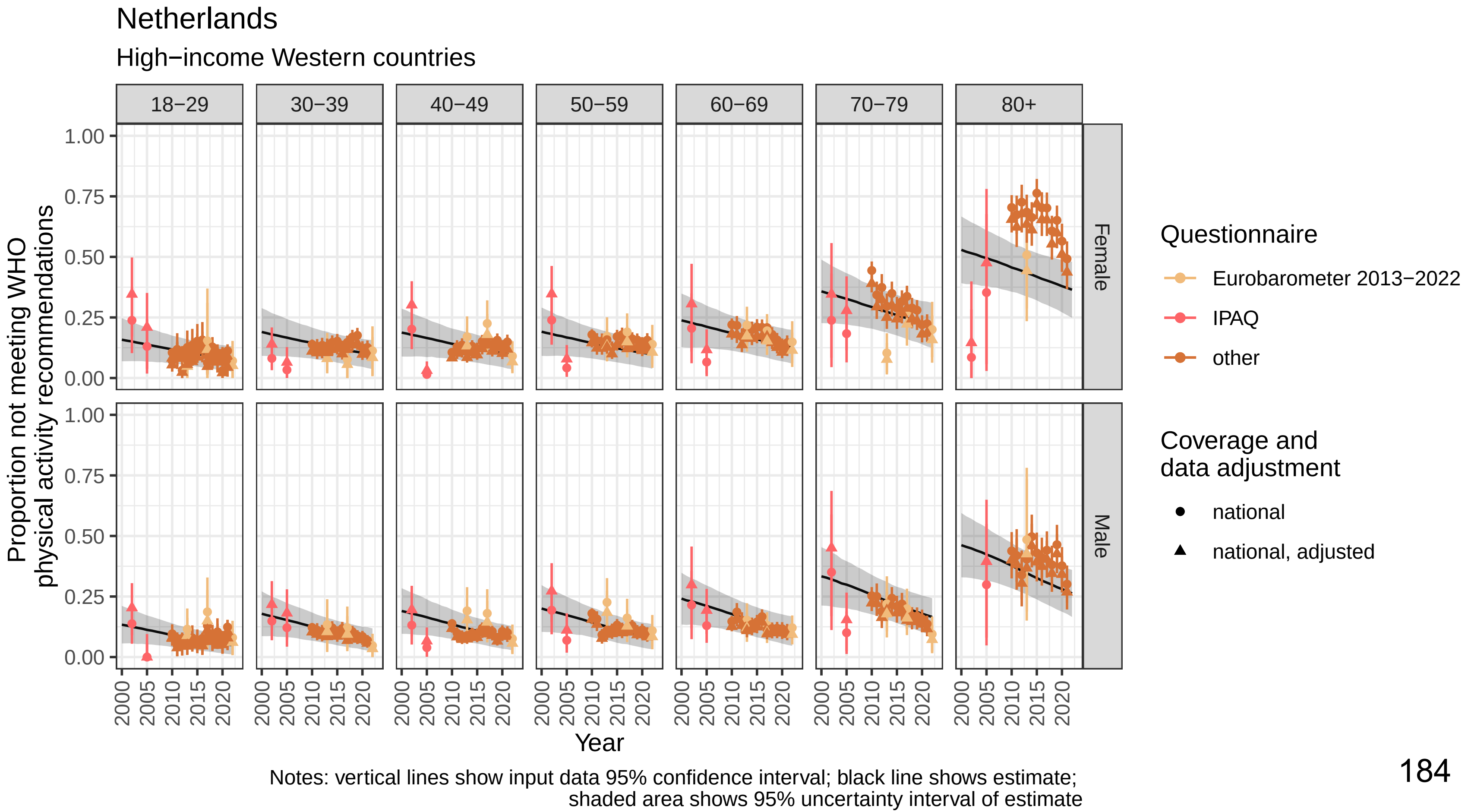

Netherlands
High−income Western countries
18−29
30−39
40−49
50−59
60−69
70−79
80+
Female
Male
Proportion not meeting WHO physical activity recommendations
1.00
0.75
0.50
0.25
0.00
2000
2005
2010
2015
2020
Year
Questionnaire
Eurobarometer 2013−2022
IPAQ
other
Coverage and data adjustment
national
national, adjusted
Notes: vertical lines show input data 95% confidence interval; black line shows estimate;
shaded area shows 95% uncertainty interval of estimate

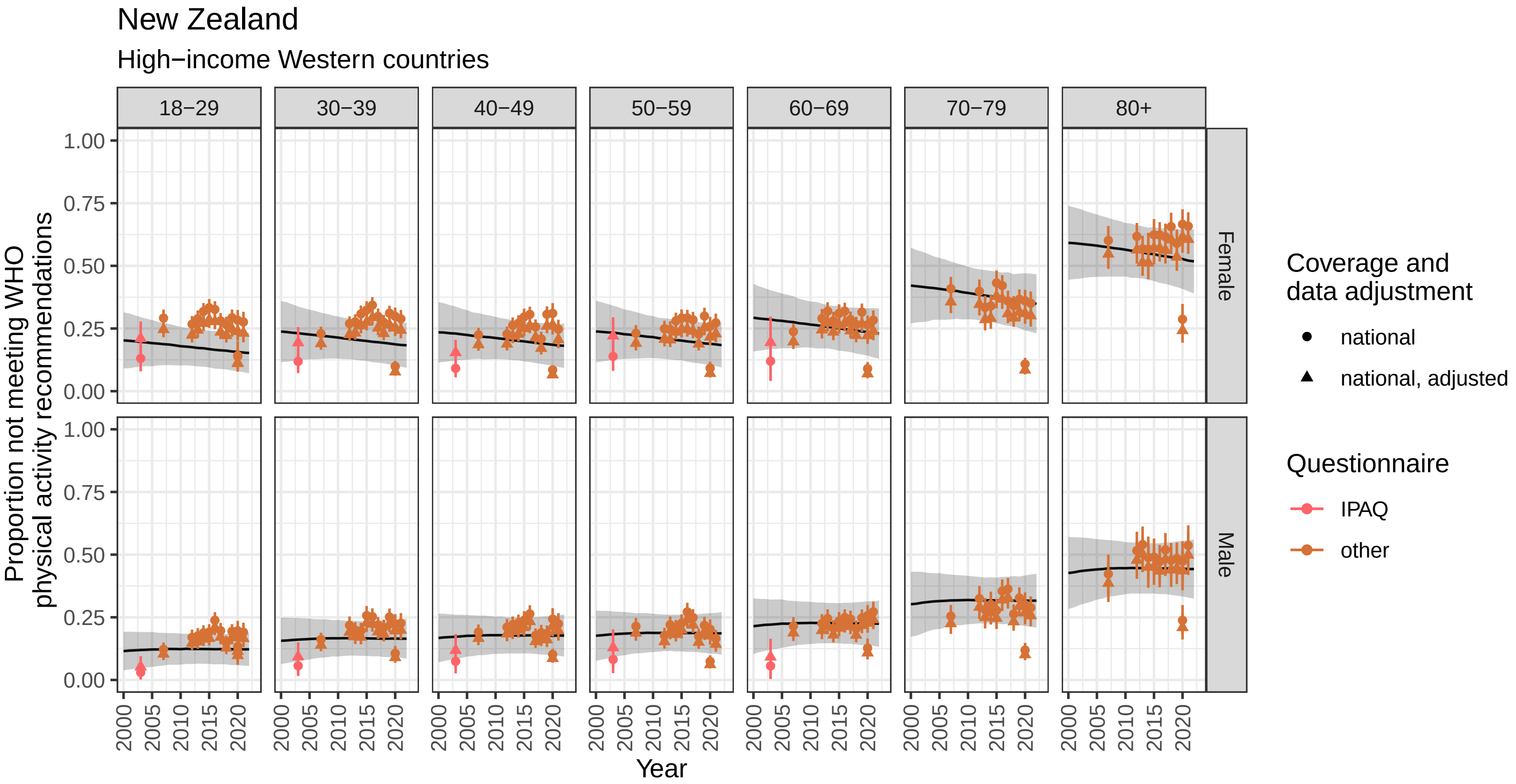

Notes: vertical lines show input data 95% confidence interval; black line shows estimate;
shaded area shows 95% uncertainty interval of estimate

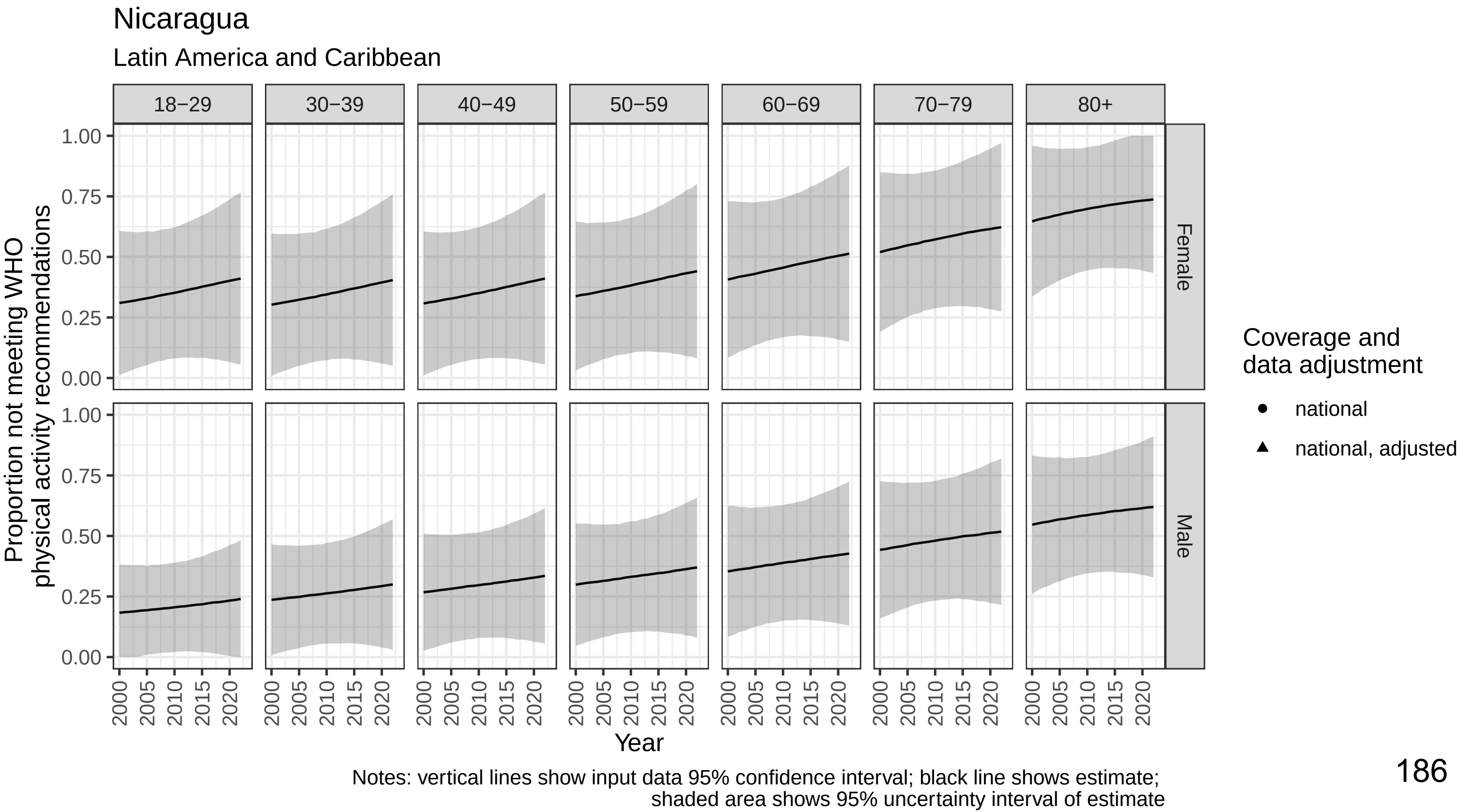

Nicaragua
Latin America and Caribbean
18−29
30−39
40−49
50−59
60−69
70−79
80+
Female
Male
Proportion not meeting WHO physical activity recommendations
1.00
0.75
0.50
0.25
0.00
2000
2005
2010
2015
2020
Year
Coverage and data adjustment
national
national, adjusted
Notes: vertical lines show input data 95% confidence interval; black line shows estimate; shaded area shows 95% uncertainty interval of estimate

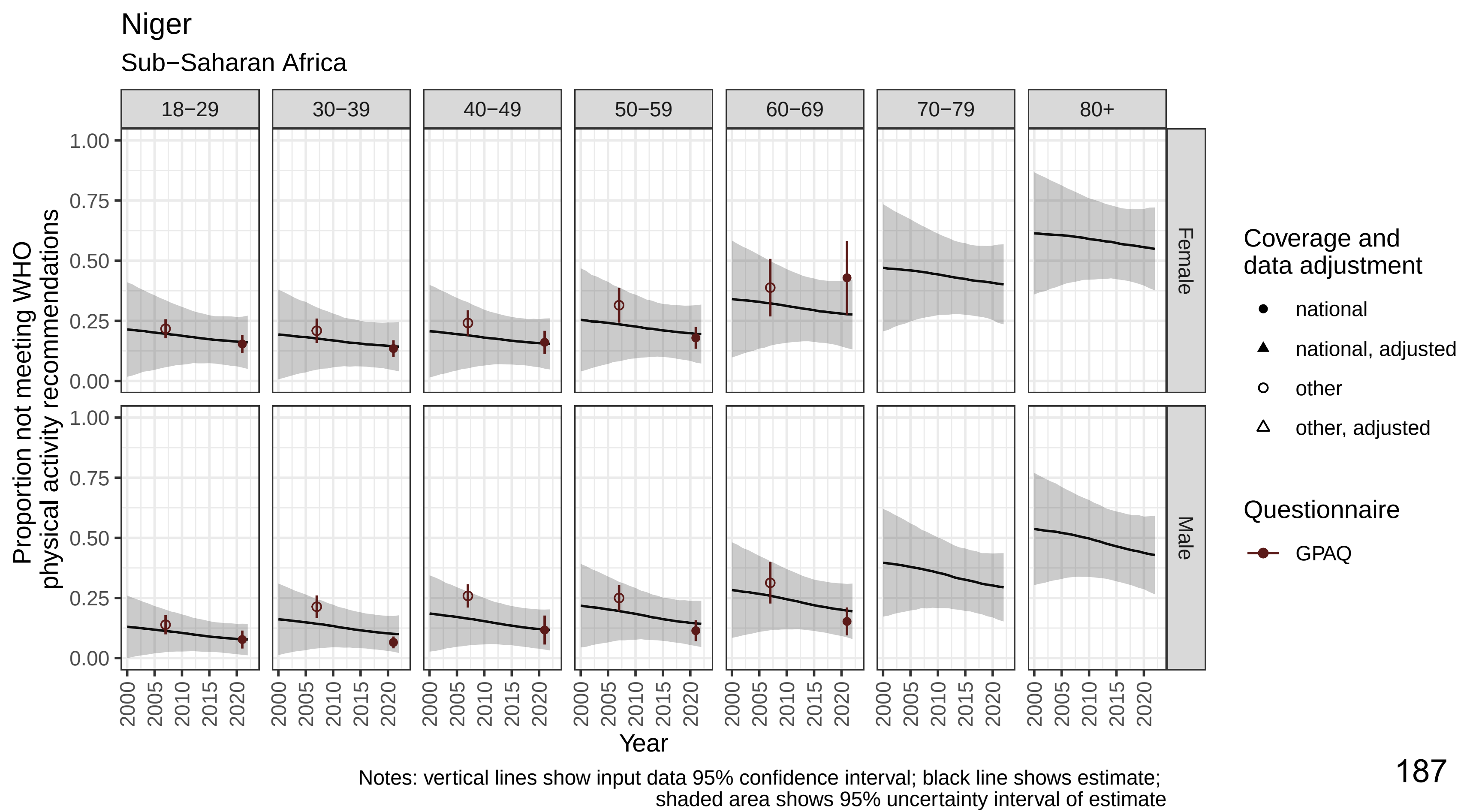

Niger
Sub−Saharan Africa
18−29
30−39
40−49
50−59
60−69
70−79
80+
Female
Male
Proportion not meeting WHO physical activity recommendations
1.00
0.75
0.50
0.25
0.00
2000
2005
2010
2015
2020
Year
Coverage and data adjustment
national
national, adjusted
other
other, adjusted
Questionnaire
GPAQ
Notes: vertical lines show input data 95% confidence interval; black line shows estimate; shaded area shows 95% uncertainty interval of estimate

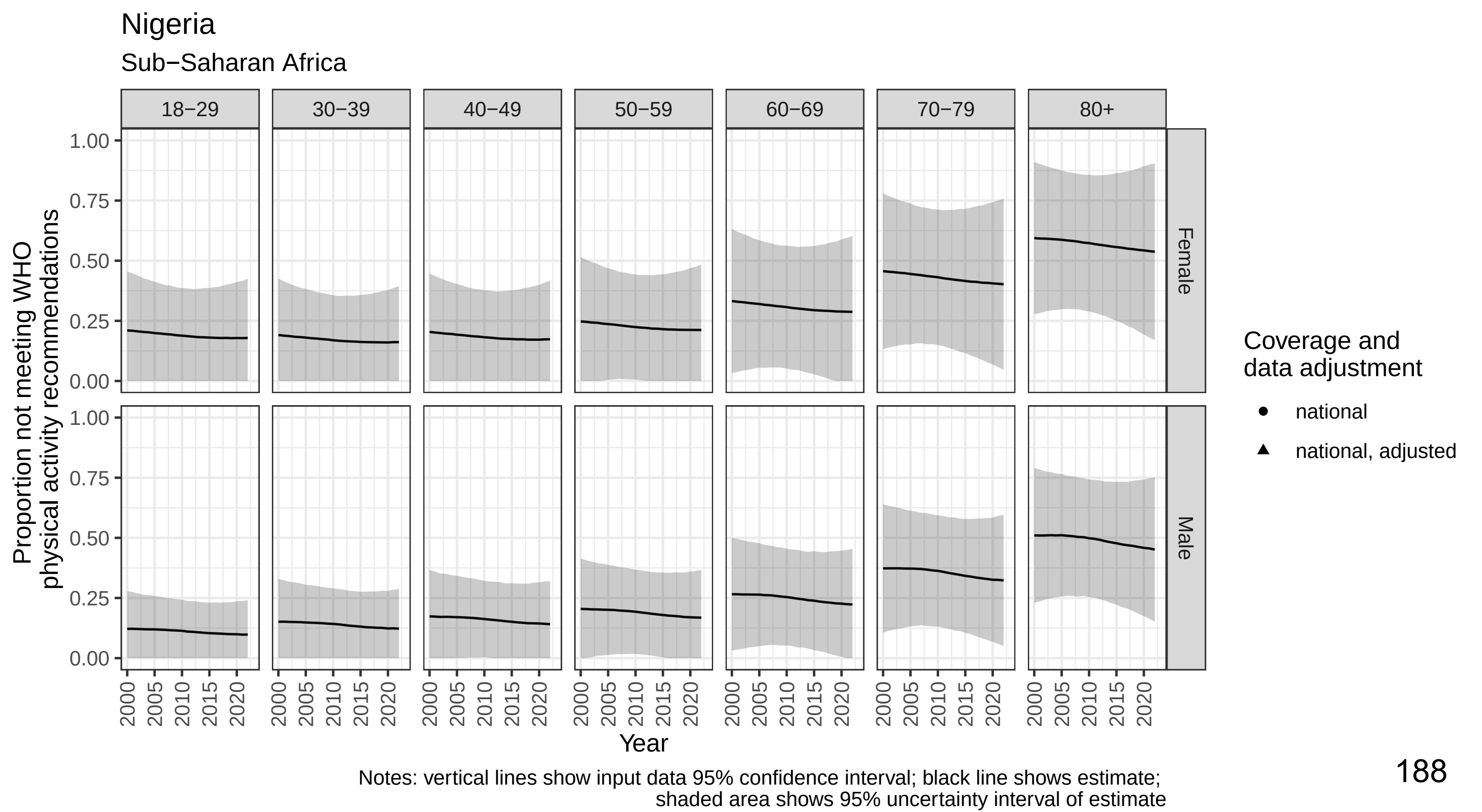

Nigeria
Sub−Saharan Africa
18−29
30−39
40−49
50−59
60−69
70−79
80+
Female
Male
Proportion not meeting WHO physical activity recommendations
1.00
0.75
0.50
0.25
0.00
2000
2005
2010
2015
2020
Year
Coverage and data adjustment
national
national, adjusted
Notes: vertical lines show input data 95% confidence interval; black line shows estimate; shaded area shows 95% uncertainty interval of estimate

Niue

Oceania

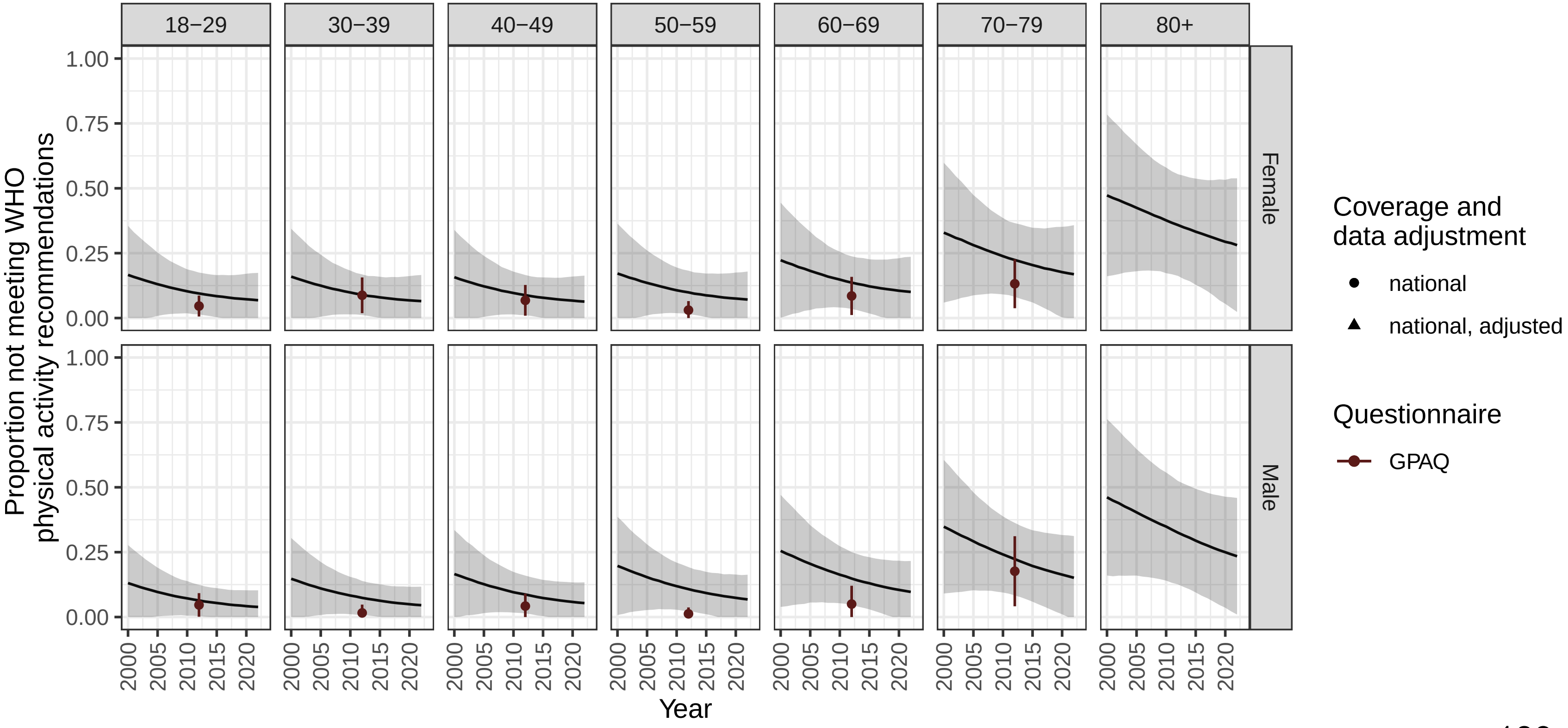

Notes: vertical lines show input data 95% confidence interval; black line shows estimate; shaded area shows 95% uncertainty interval of estimate

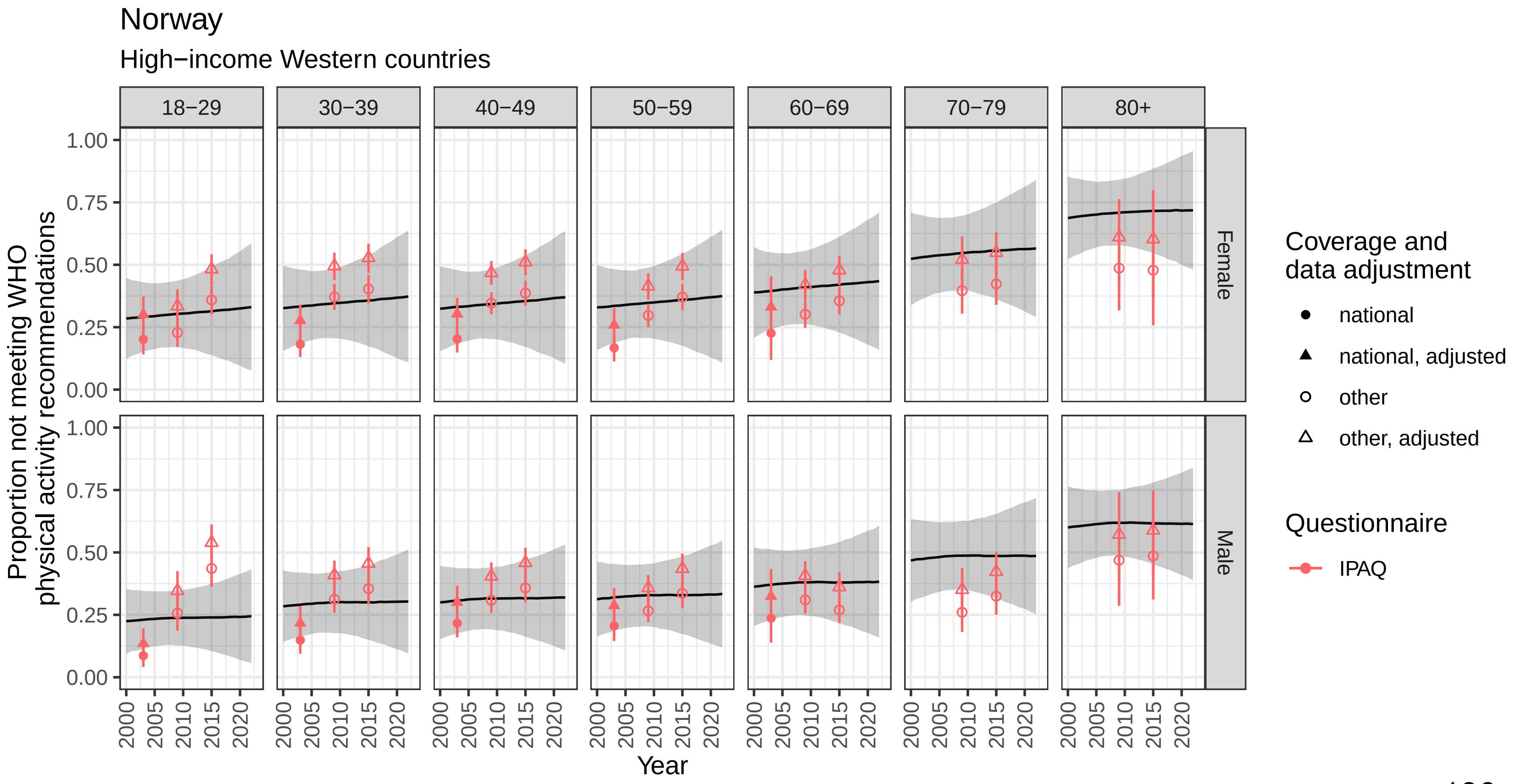

Notes: vertical lines show input data 95% confidence interval; black line shows estimate; shaded area shows 95% uncertainty interval of estimate

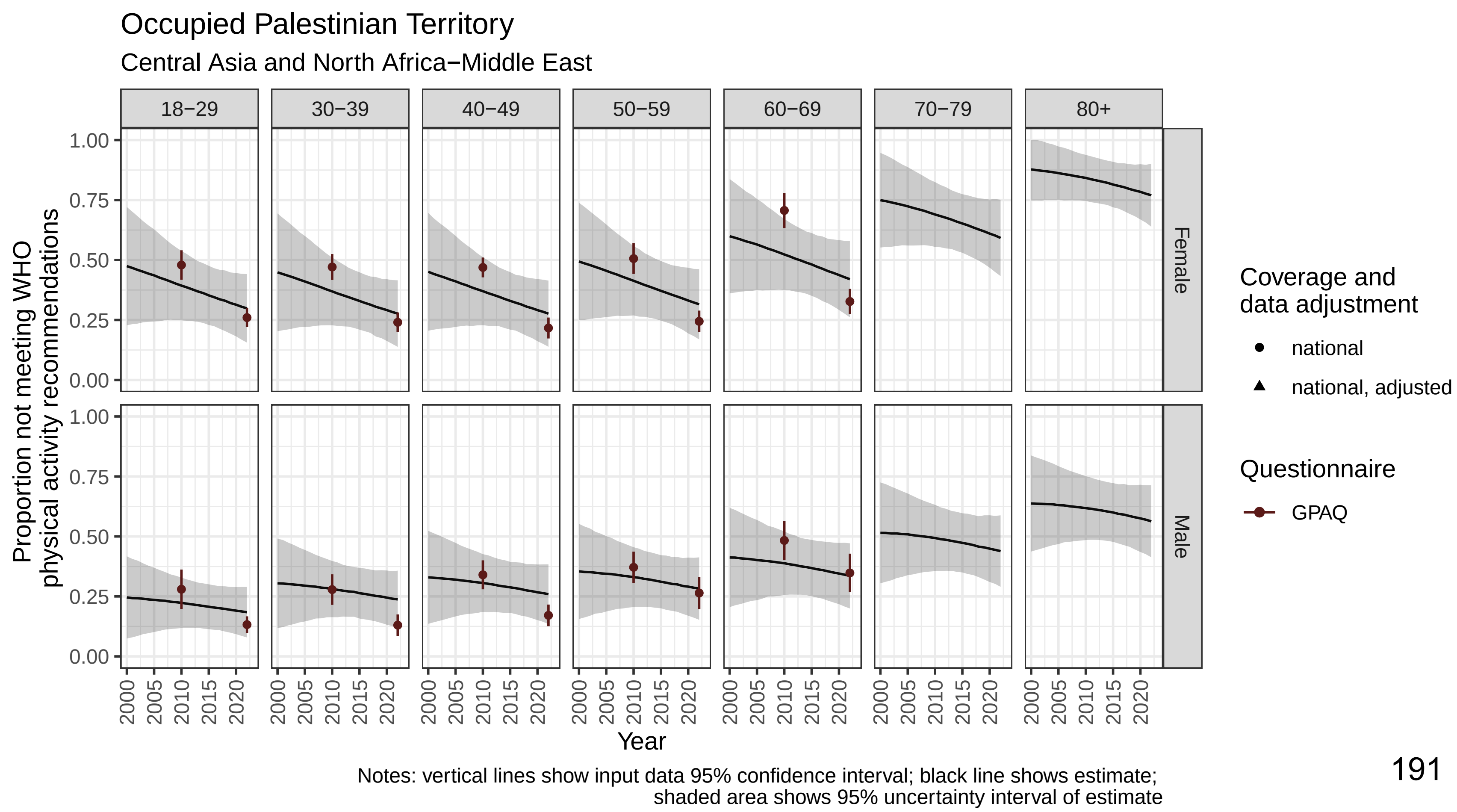
Occupied Palestinian Territory
Central Asia and North Africa−Middle East
18−29
30−39
40−49
50−59
60−69
70−79
80+
Female
Male
Proportion not meeting WHO physical activity recommendations
1.00
0.75
0.50
0.25
0.00
2000
2005
2010
2015
2020
Year
Coverage and data adjustment
national
national, adjusted
Questionnaire
GPAQ
Notes: vertical lines show input data 95% confidence interval; black line shows estimate; shaded area shows 95% uncertainty interval of estimate

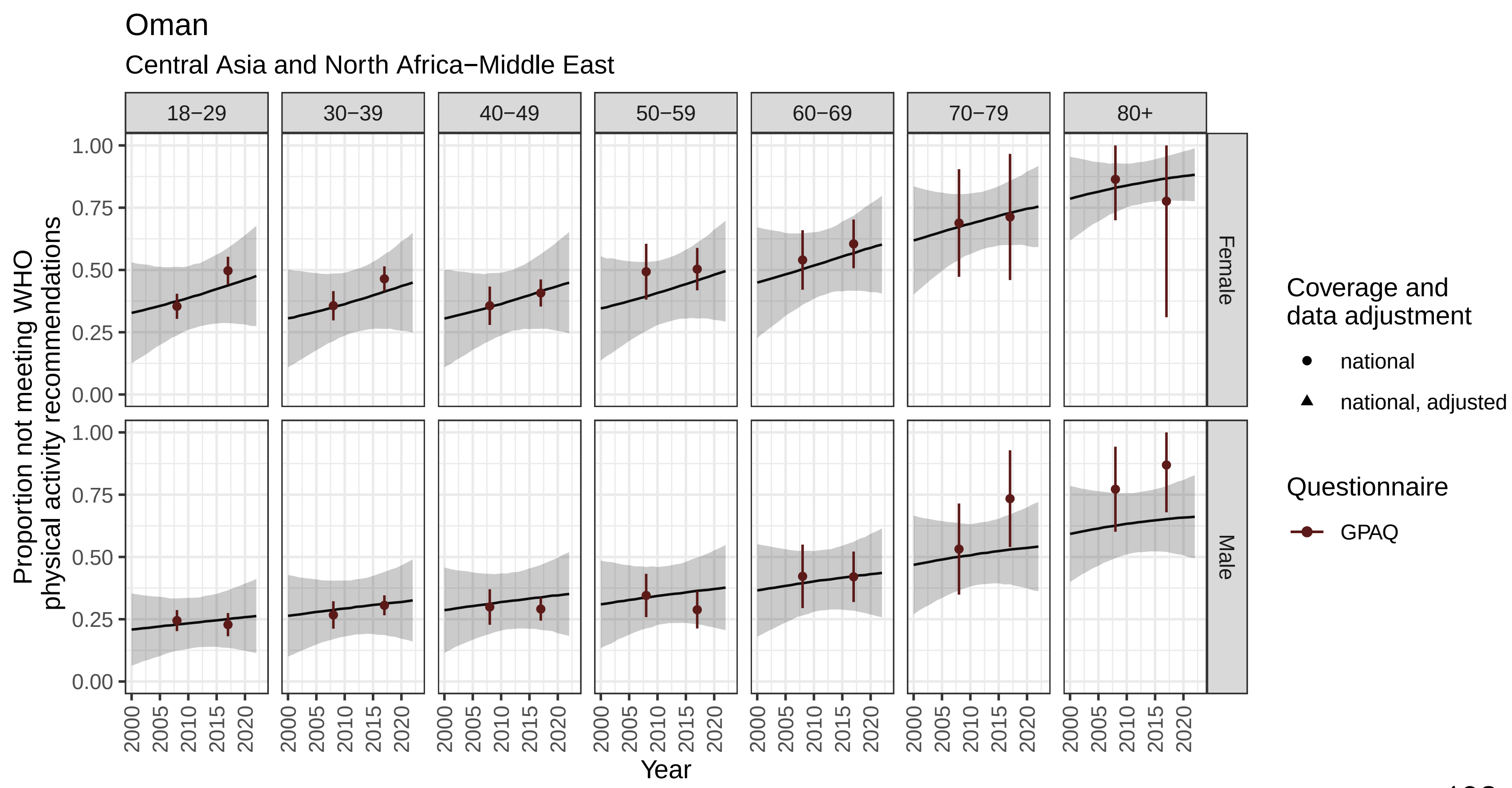

Notes: vertical lines show input data 95% confidence interval; black line shows estimate; shaded area shows 95% uncertainty interval of estimate

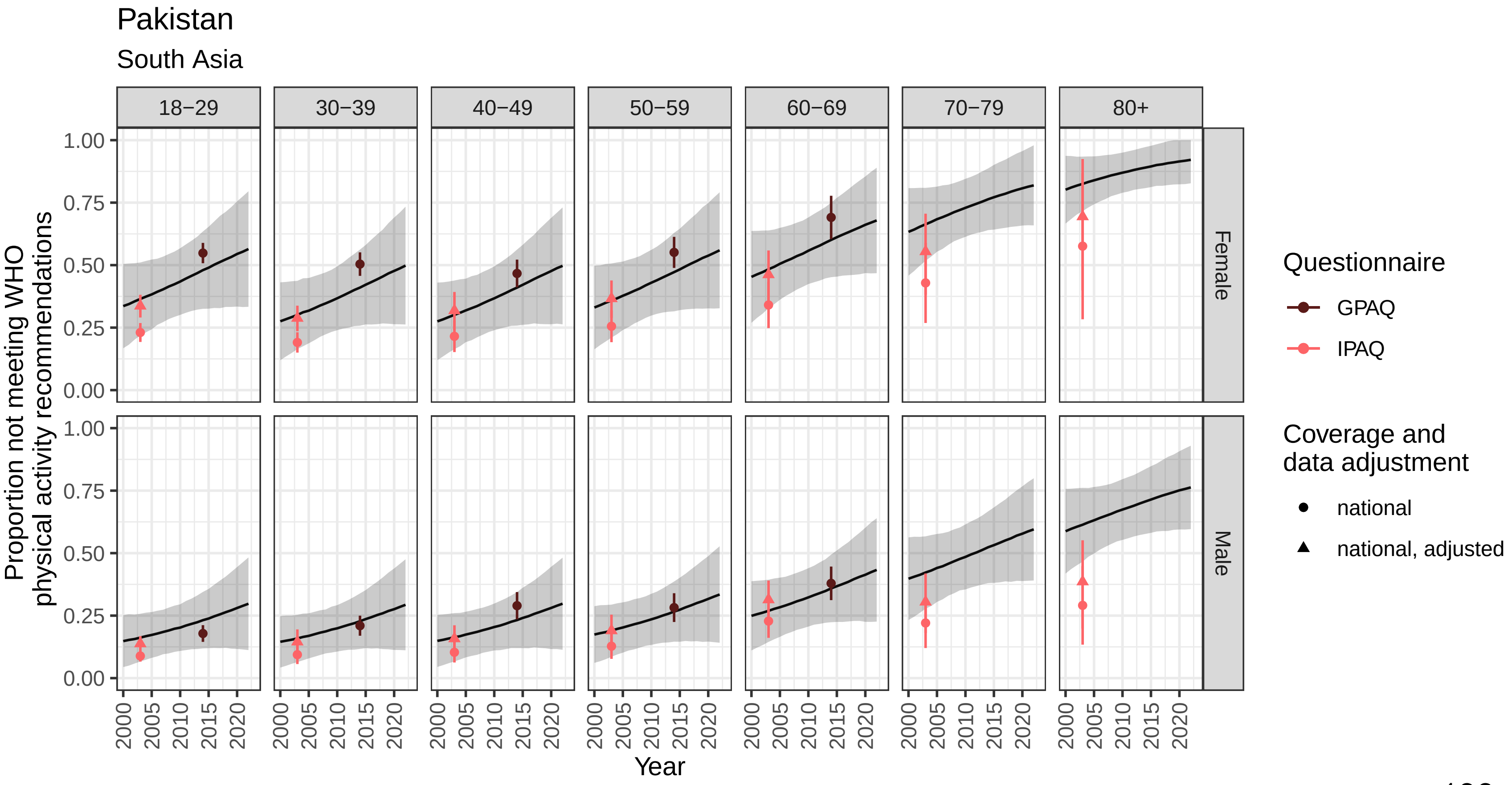

Notes: vertical lines show input data 95% confidence interval; black line shows estimate; shaded area shows 95% uncertainty interval of estimate

Palau

Oceania

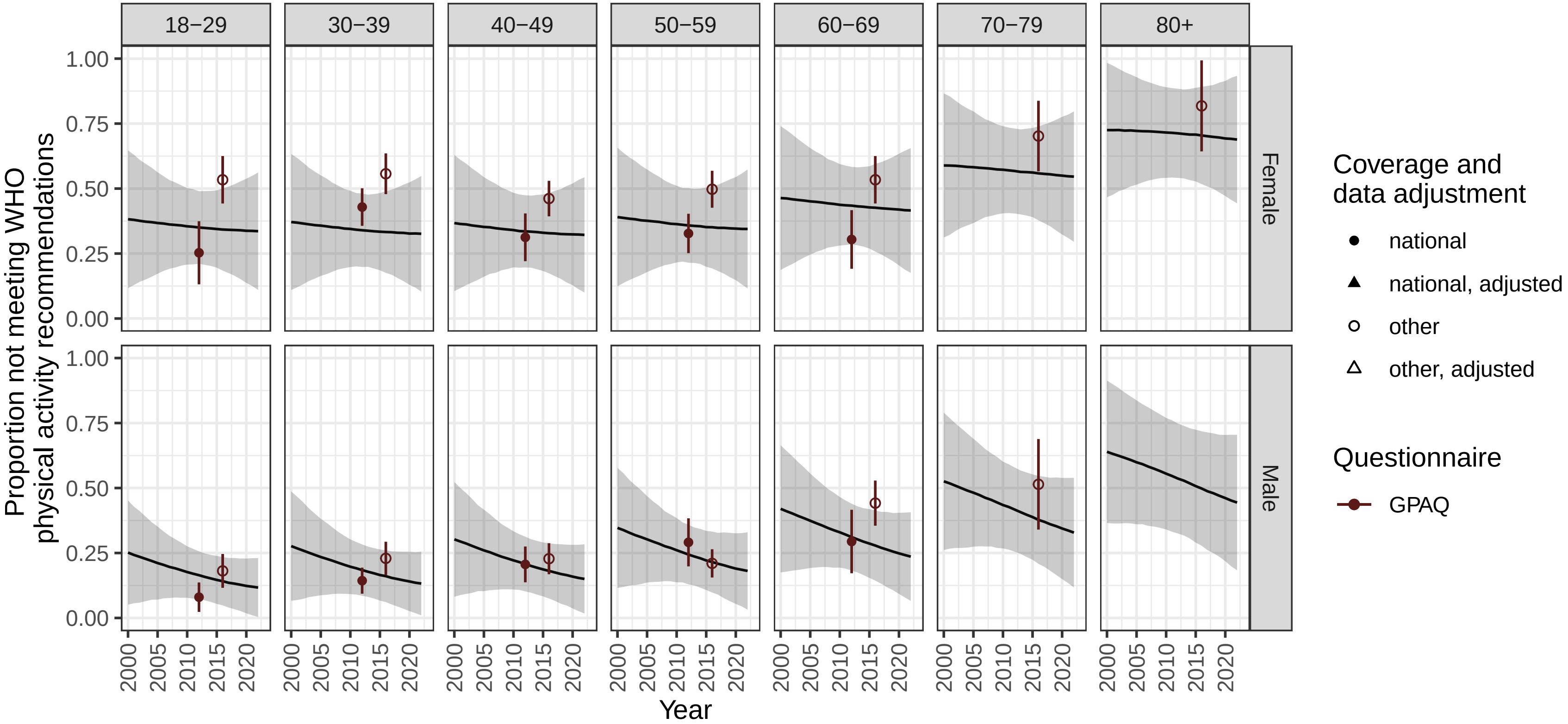

Notes: vertical lines show input data 95% confidence interval; black line shows estimate; shaded area shows 95% uncertainty interval of estimate

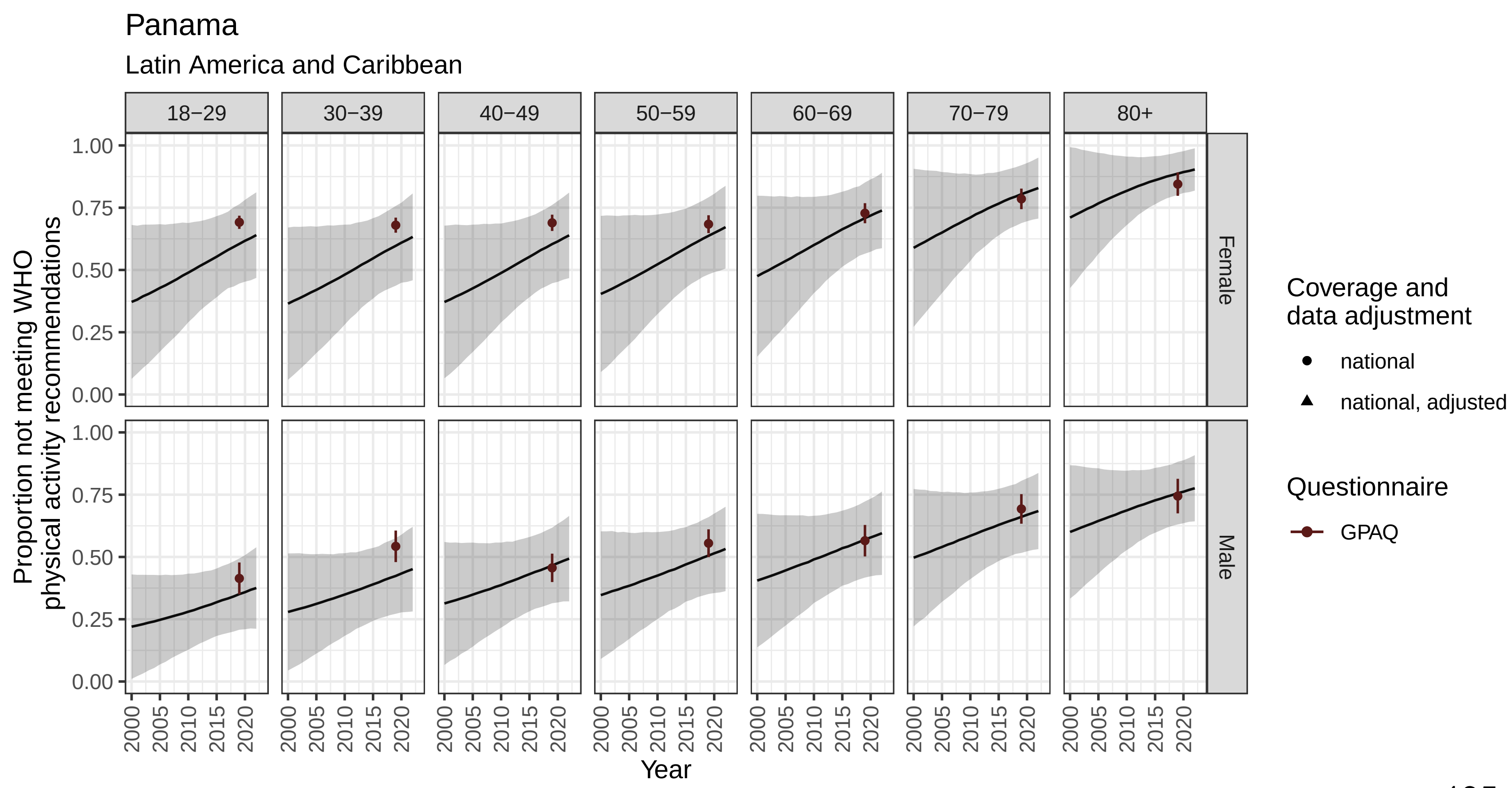

Notes: vertical lines show input data 95% confidence interval; black line shows estimate; shaded area shows 95% uncertainty interval of estimate

Papua New Guinea

Oceania

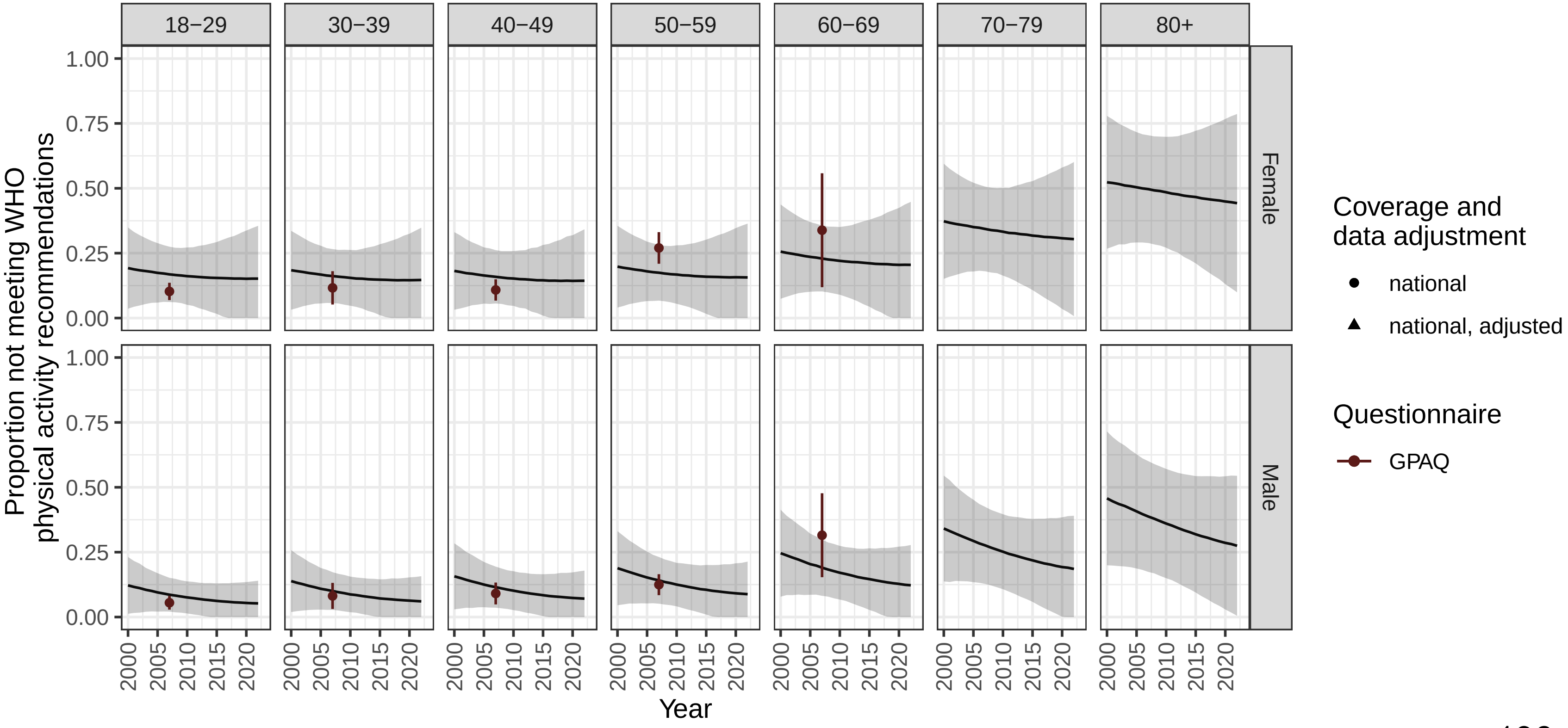

Notes: vertical lines show input data 95% confidence interval; black line shows estimate; shaded area shows 95% uncertainty interval of estimate

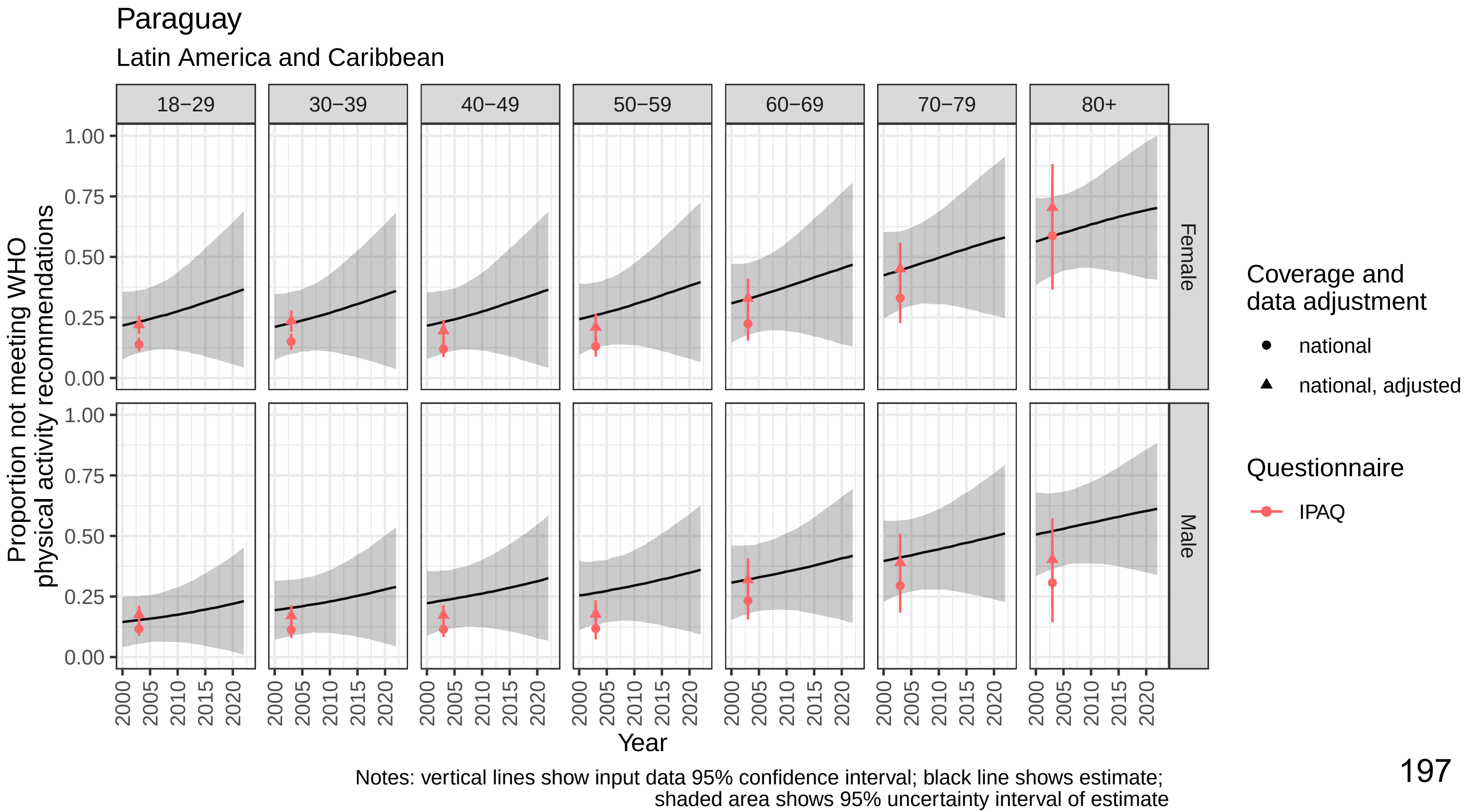

Paraguay
Latin America and Caribbean
18−29
30−39
40−49
50−59
60−69
70−79
80+
Female
Male
Proportion not meeting WHO physical activity recommendations
1.00
0.75
0.50
0.25
0.00
2000
2005
2010
2015
2020
Year
Coverage and data adjustment
national
national, adjusted
Questionnaire
IPAQ
Notes: vertical lines show input data 95% confidence interval; black line shows estimate; shaded area shows 95% uncertainty interval of estimate

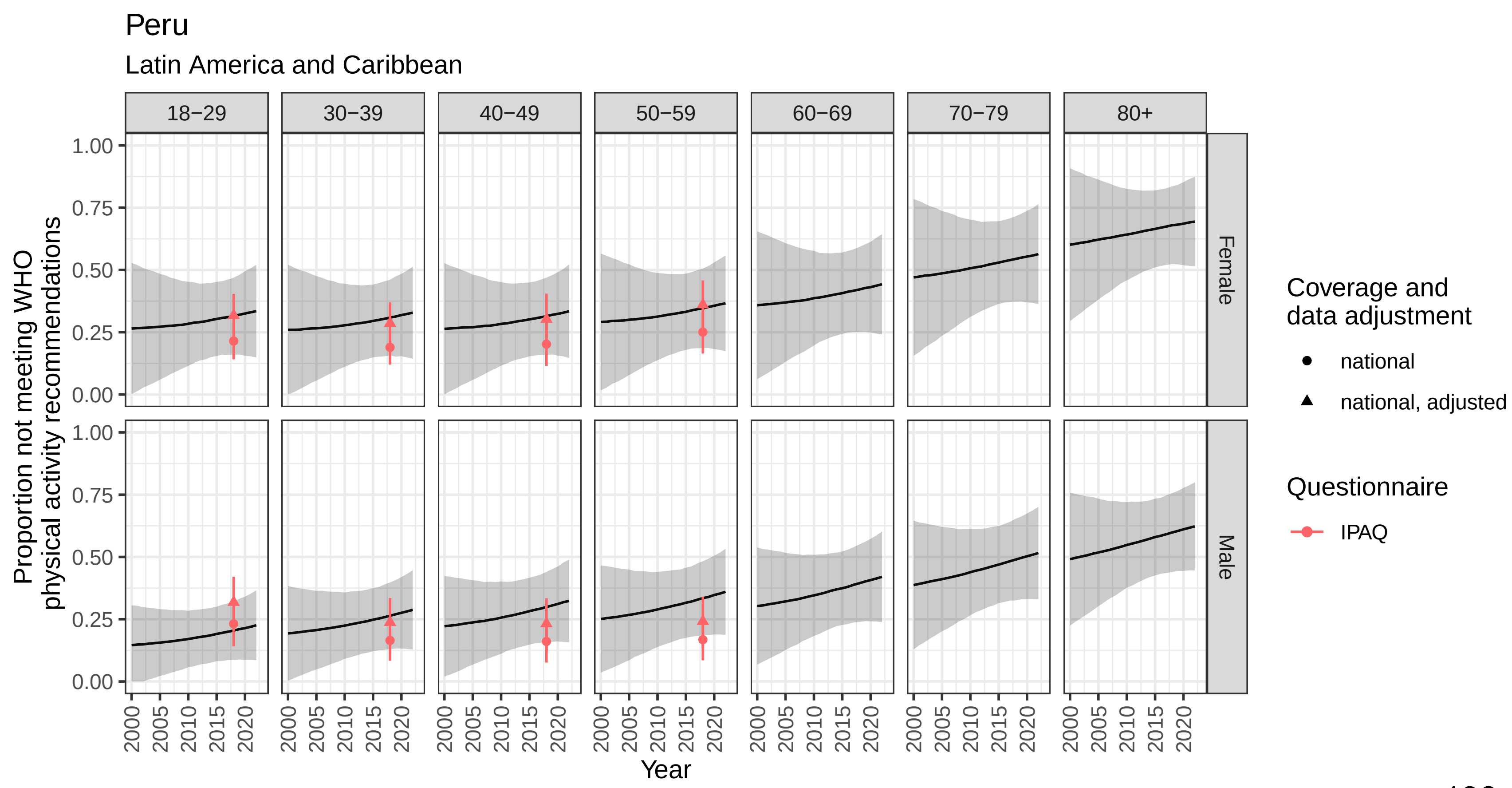

Notes: vertical lines show input data 95% confidence interval; black line shows estimate; shaded area shows 95% uncertainty interval of estimate

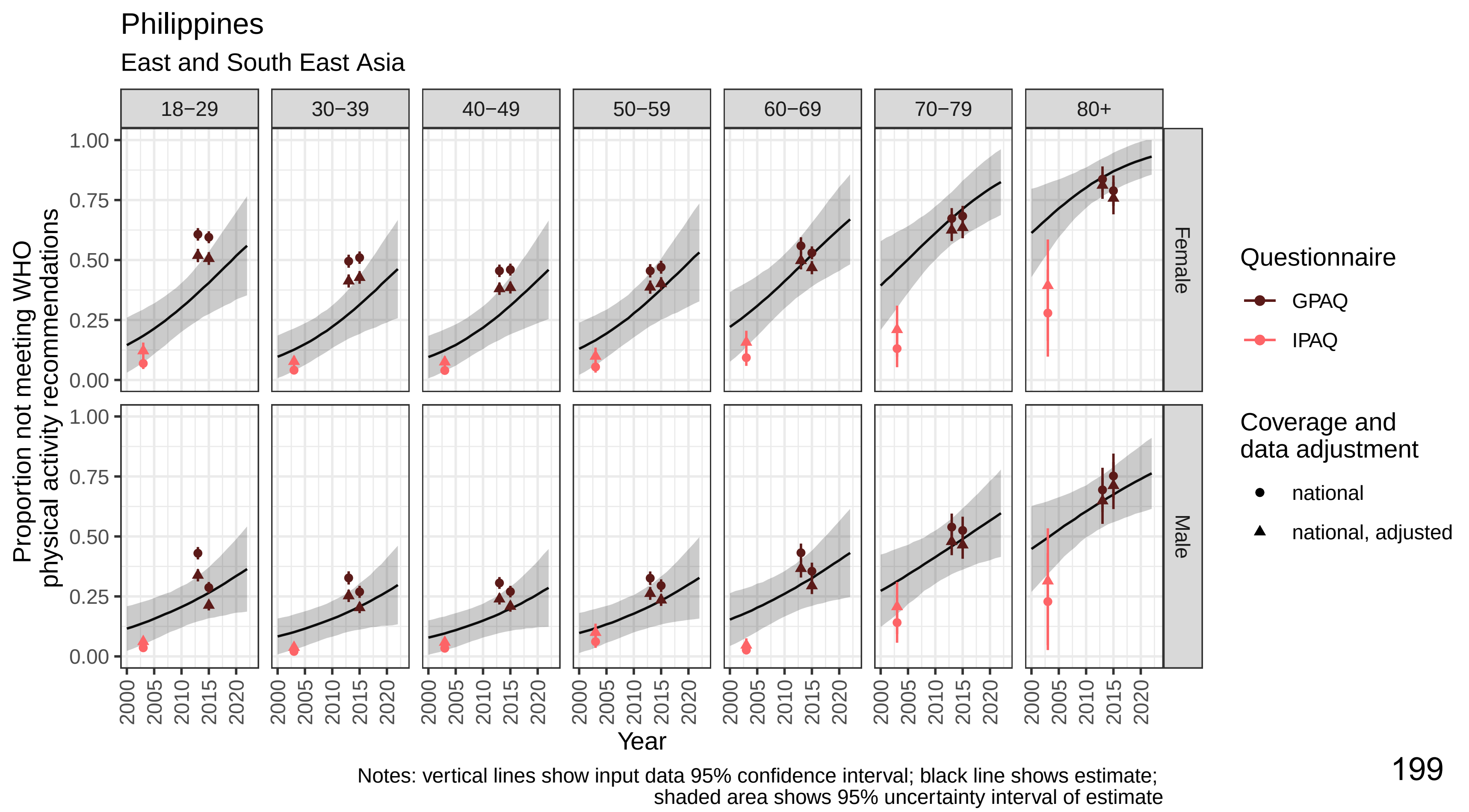

Philippines
East and South East Asia
18−29
30−39
40−49
50−59
60−69
70−79
80+
Female
Male
Proportion not meeting WHO physical activity recommendations
Year
Questionnaire
GPAQ
IPAQ
Coverage and data adjustment
national
national, adjusted
Notes: vertical lines show input data 95% confidence interval; black line shows estimate; shaded area shows 95% uncertainty interval of estimate

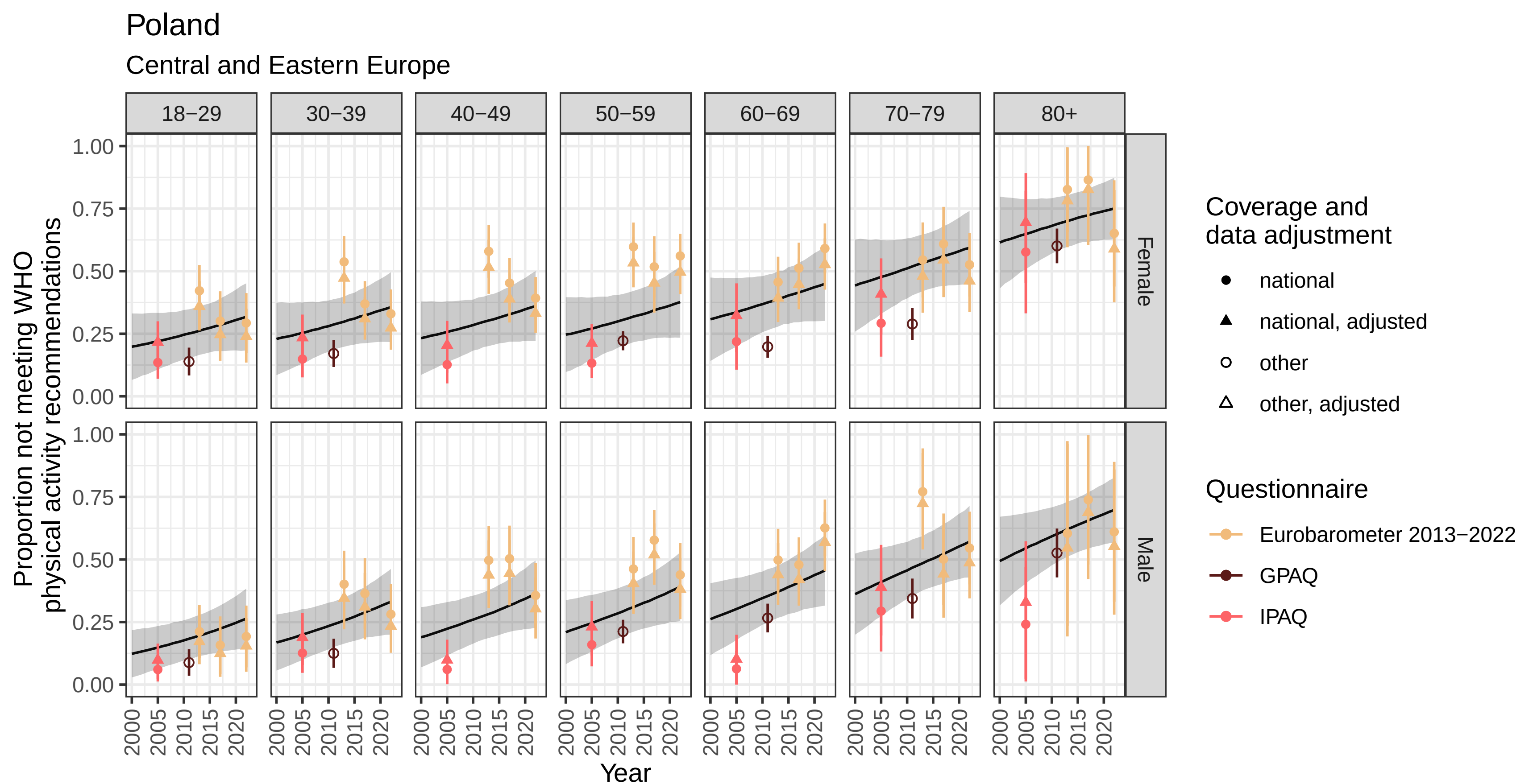

Notes: vertical lines show input data 95% confidence interval; black line shows estimate; shaded area shows 95% uncertainty interval of estimate

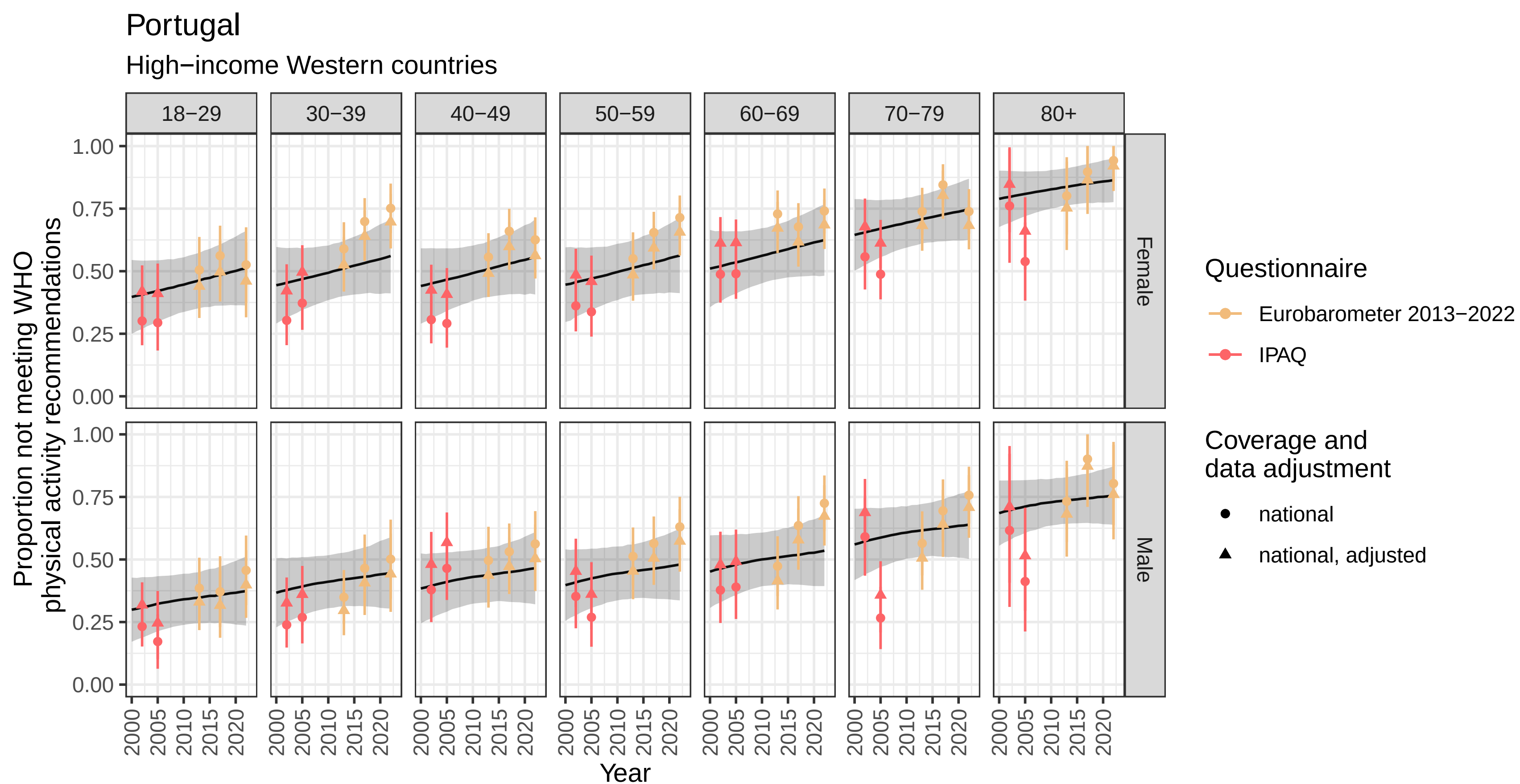

Notes: vertical lines show input data 95% confidence interval; black line shows estimate; shaded area shows 95% uncertainty interval of estimate

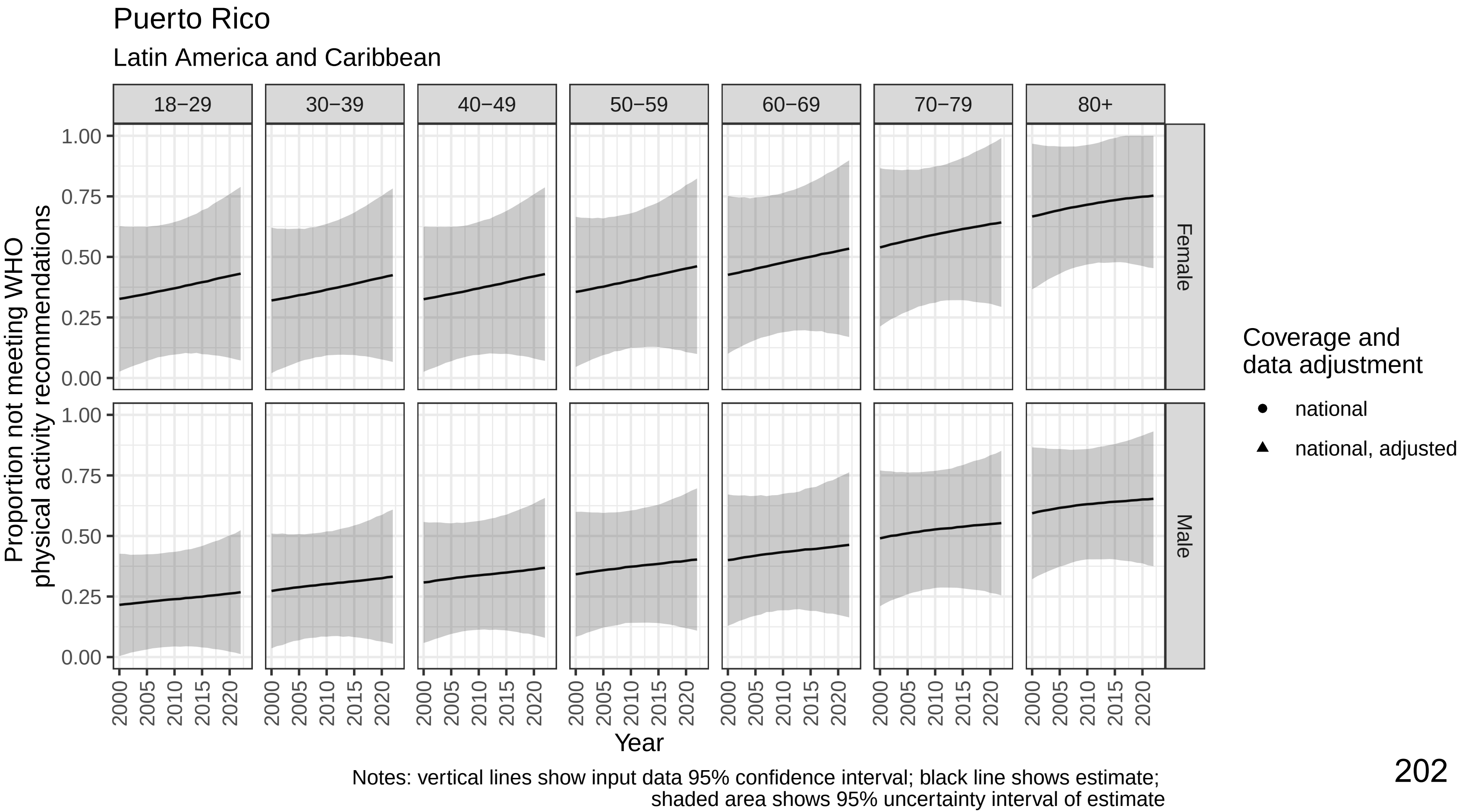
Puerto Rico
Latin America and Caribbean
18−29
30−39
40−49
50−59
60−69
70−79
80+
Female
Male
Proportion not meeting WHO physical activity recommendations
Year
Coverage and data adjustment
national
national, adjusted
Notes: vertical lines show input data 95% confidence interval; black line shows estimate; shaded area shows 95% uncertainty interval of estimate

Qatar

Central Asia and North Africa−Middle East

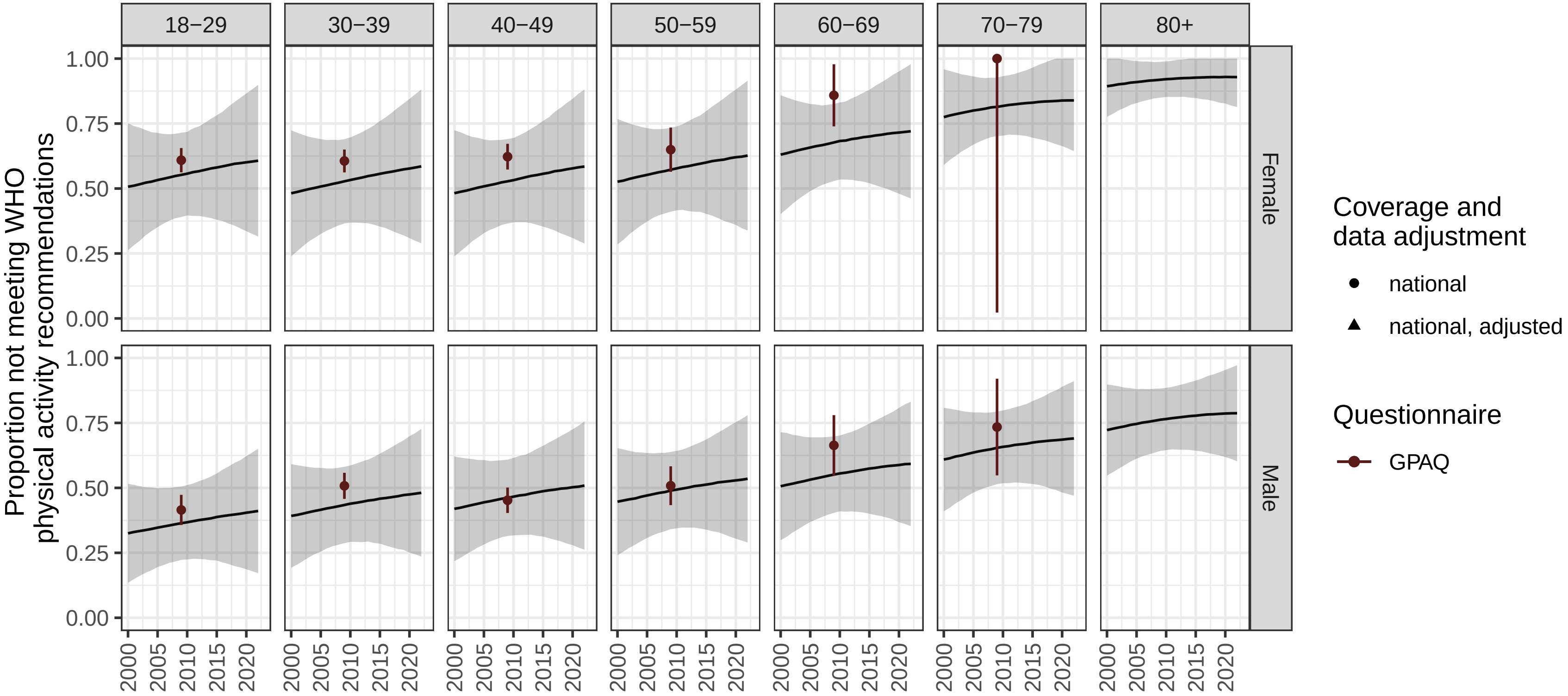

Notes: vertical lines show input data 95% confidence interval; black line shows estimate; shaded area shows 95% uncertainty interval of estimate

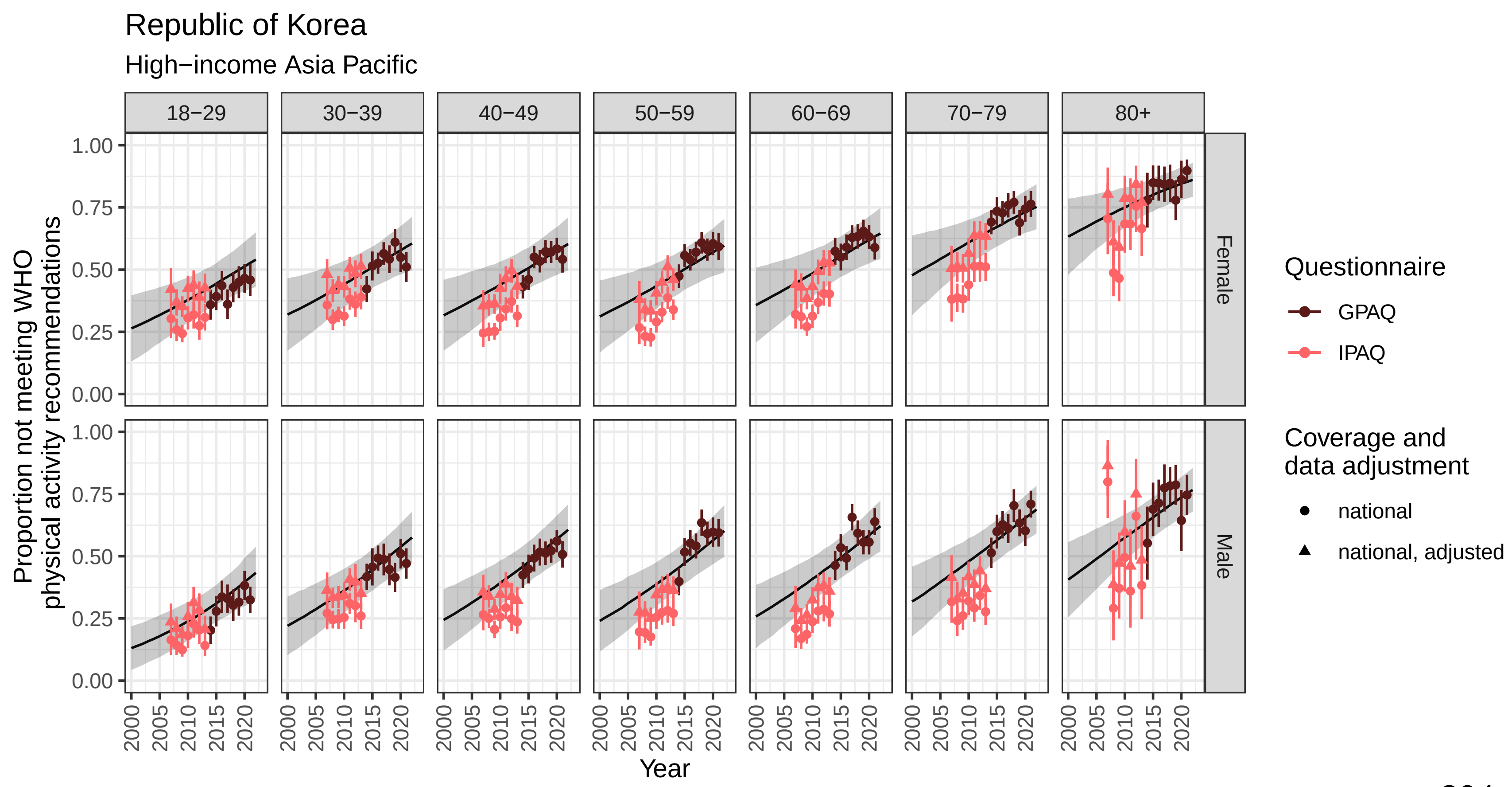

Notes: vertical lines show input data 95% confidence interval; black line shows estimate; shaded area shows 95% uncertainty interval of estimate

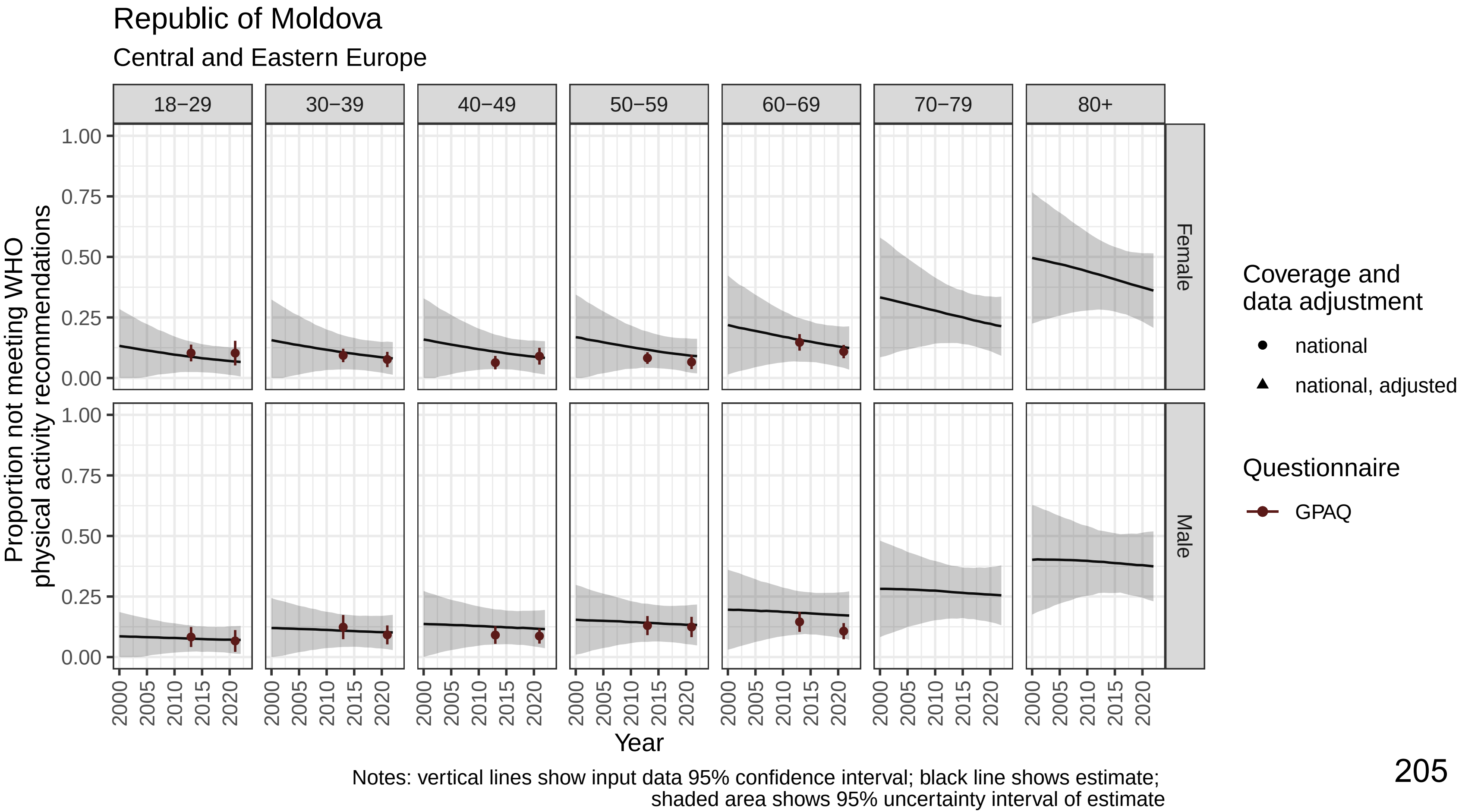

Republic of Moldova
Central and Eastern Europe
18−29
30−39
40−49
50−59
60−69
70−79
80+
Female
Male
Proportion not meeting WHO physical activity recommendations
1.00
0.75
0.50
0.25
0.00
2000
2005
2010
2015
2020
Year
Coverage and data adjustment
national
national, adjusted
Questionnaire
GPAQ
Notes: vertical lines show input data 95% confidence interval; black line shows estimate; shaded area shows 95% uncertainty interval of estimate

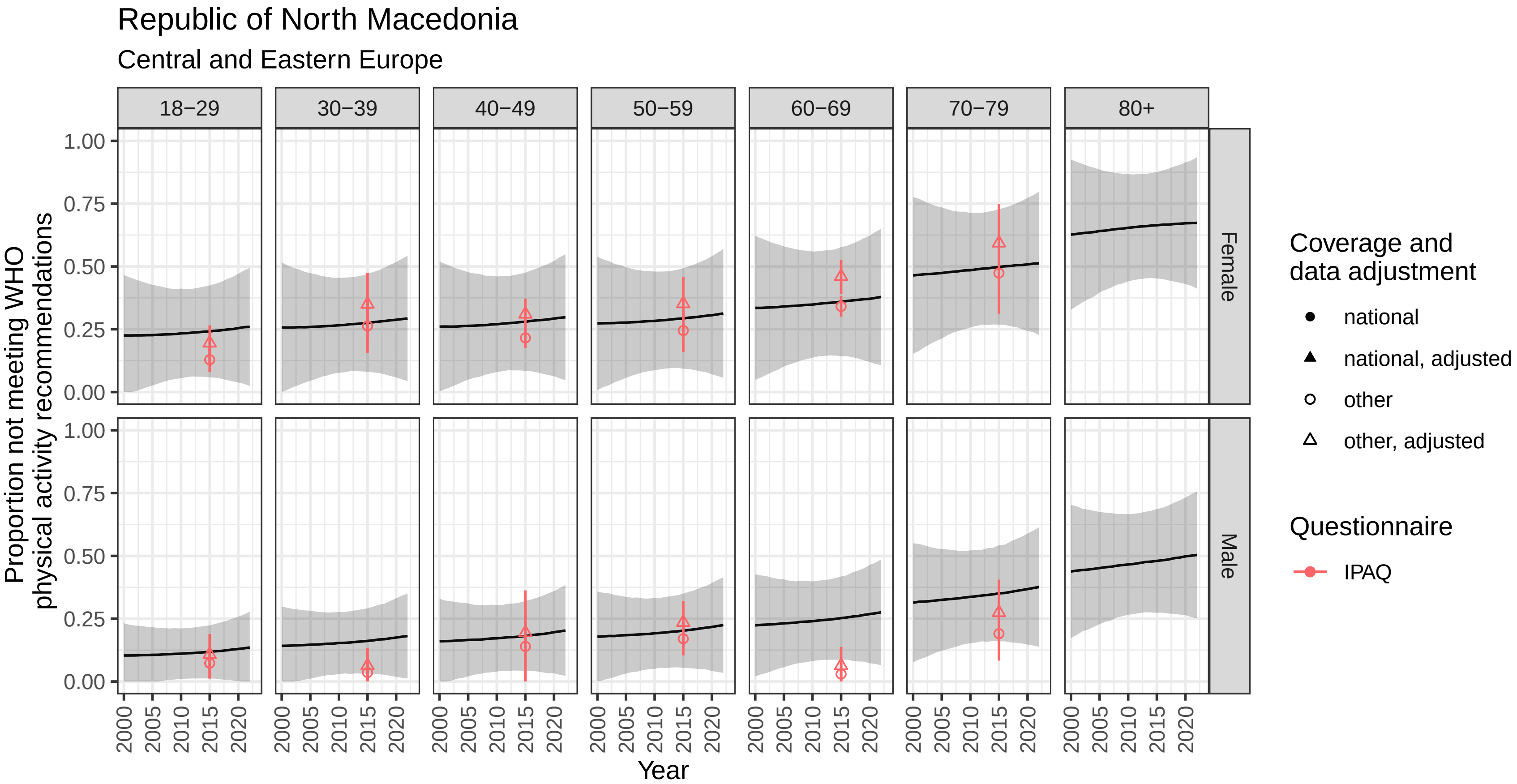

Notes: vertical lines show input data 95% confidence interval; black line shows estimate; shaded area shows 95% uncertainty interval of estimate

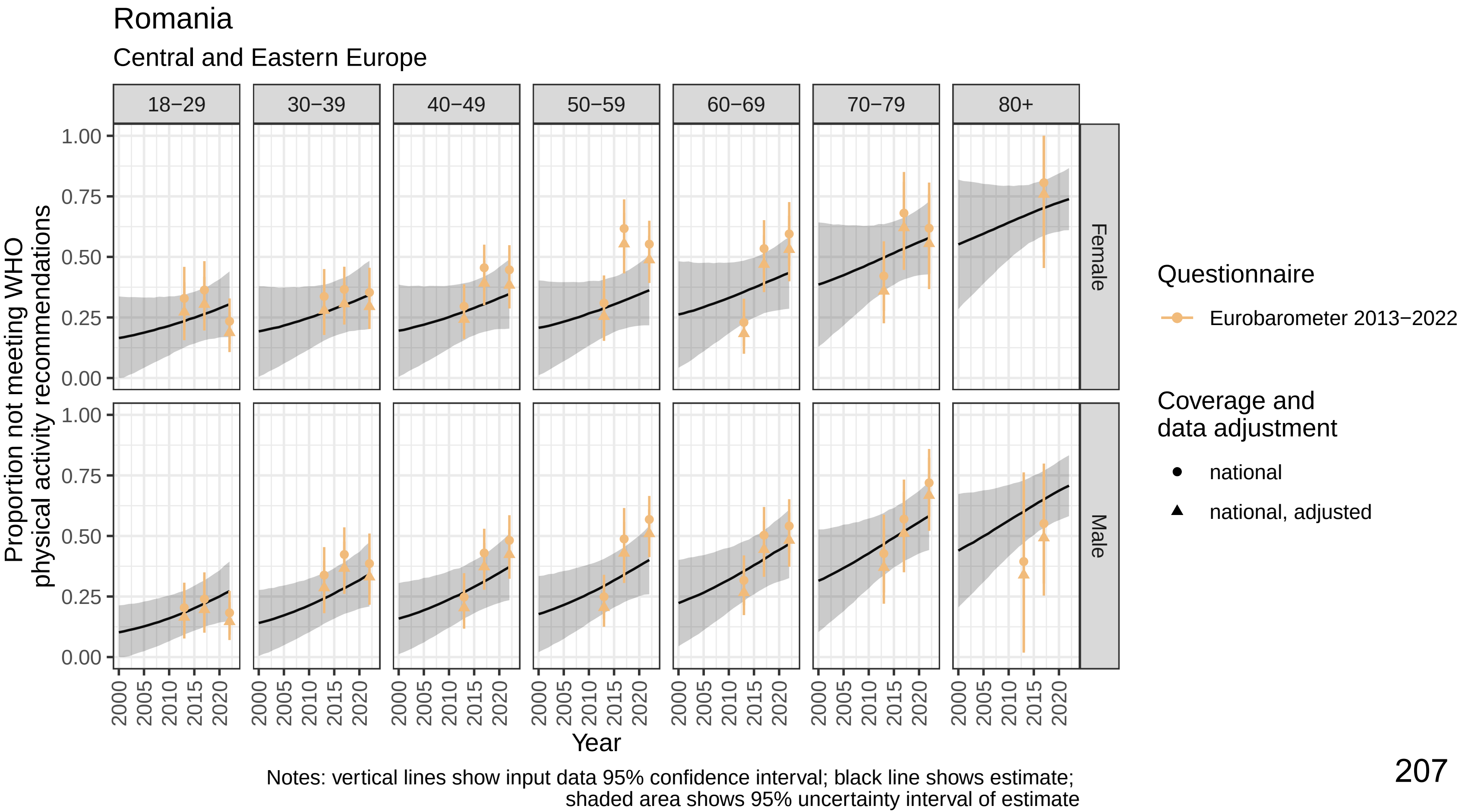
Romania
Central and Eastern Europe
18−29
30−39
40−49
50−59
60−69
70−79
80+
Female
Male
Proportion not meeting WHO physical activity recommendations
1.00
0.75
0.50
0.25
0.00
2000
2005
2010
2015
2020
Year
Questionnaire
Eurobarometer 2013−2022
Coverage and data adjustment
national
national, adjusted
Notes: vertical lines show input data 95% confidence interval; black line shows estimate;
shaded area shows 95% uncertainty interval of estimate

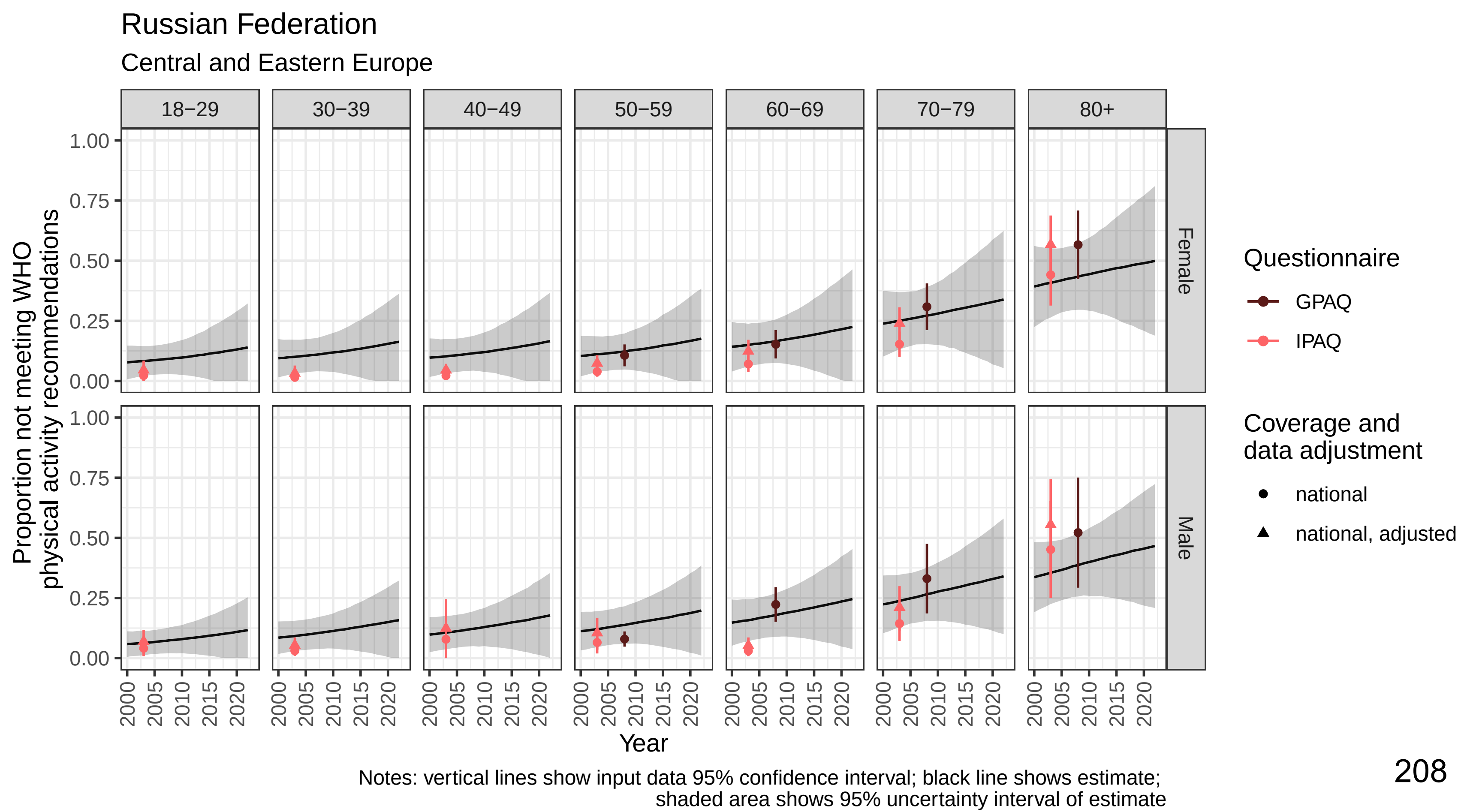

Russian Federation
Central and Eastern Europe
18−29
30−39
40−49
50−59
60−69
70−79
80+
Female
Male
Proportion not meeting WHO physical activity recommendations
0.00
0.25
0.50
0.75
1.00
2000
2005
2010
2015
2020
Year
Questionnaire
GPAQ
IPAQ
Coverage and data adjustment
national
national, adjusted
Notes: vertical lines show input data 95% confidence interval; black line shows estimate; shaded area shows 95% uncertainty interval of estimate

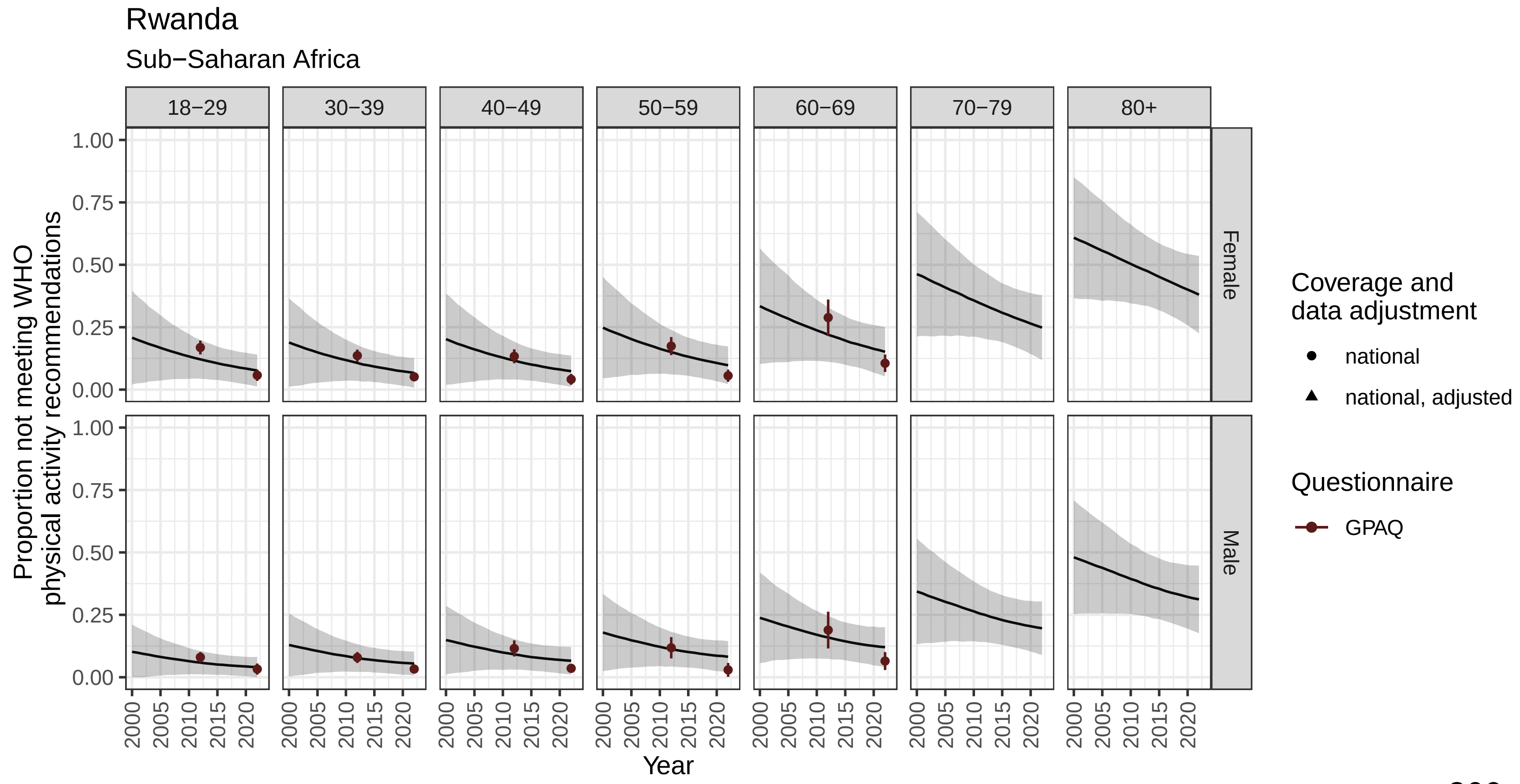

Notes: vertical lines show input data 95% confidence interval; black line shows estimate; shaded area shows 95% uncertainty interval of estimate

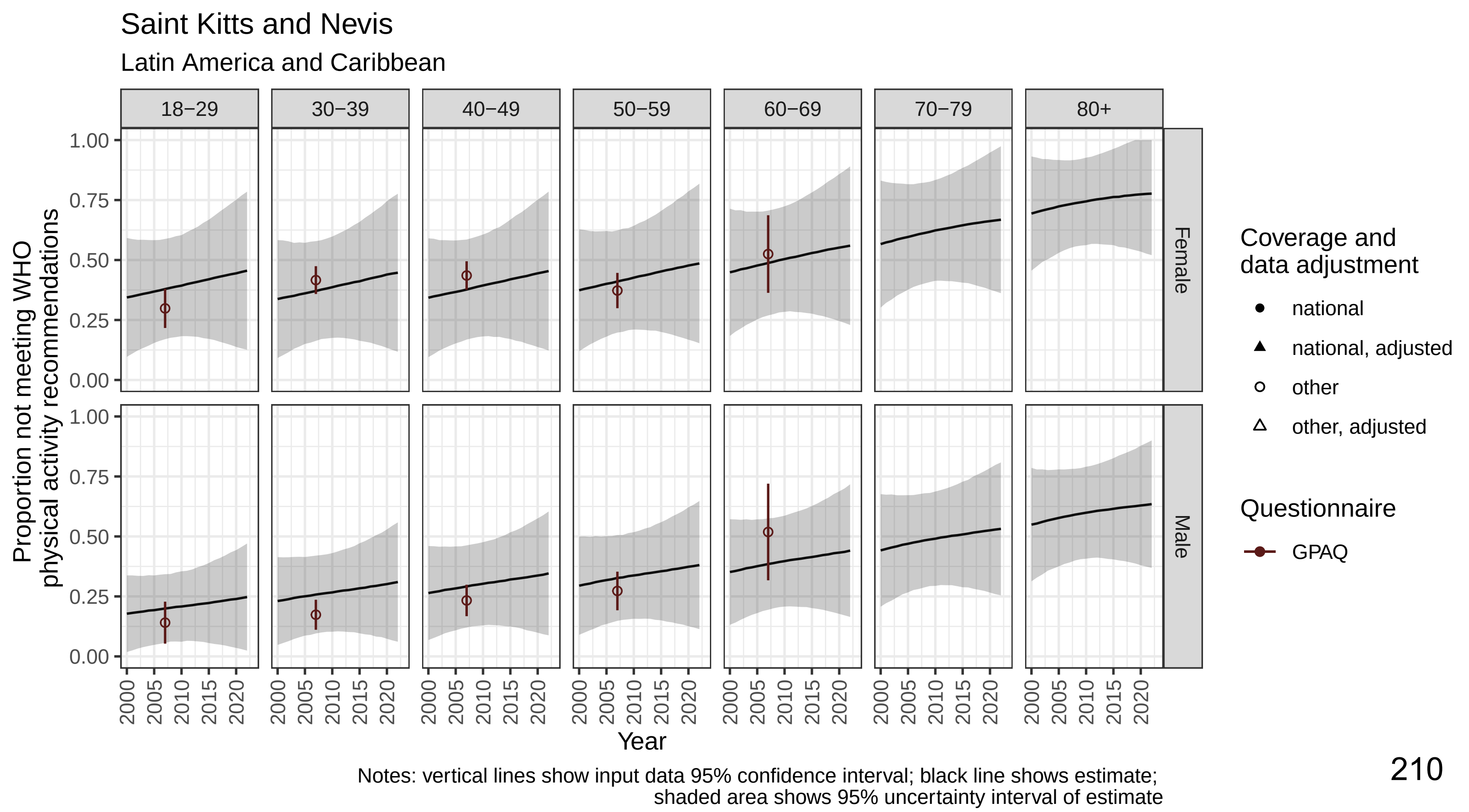
Saint Kitts and Nevis
Latin America and Caribbean
18−29
30−39
40−49
50−59
60−69
70−79
80+
Female
Male
Proportion not meeting WHO physical activity recommendations
1.00
0.75
0.50
0.25
0.00
2000
2005
2010
2015
2020
Year
Coverage and data adjustment
national
national, adjusted
other
other, adjusted
Questionnaire
GPAQ
Notes: vertical lines show input data 95% confidence interval; black line shows estimate;
shaded area shows 95% uncertainty interval of estimate

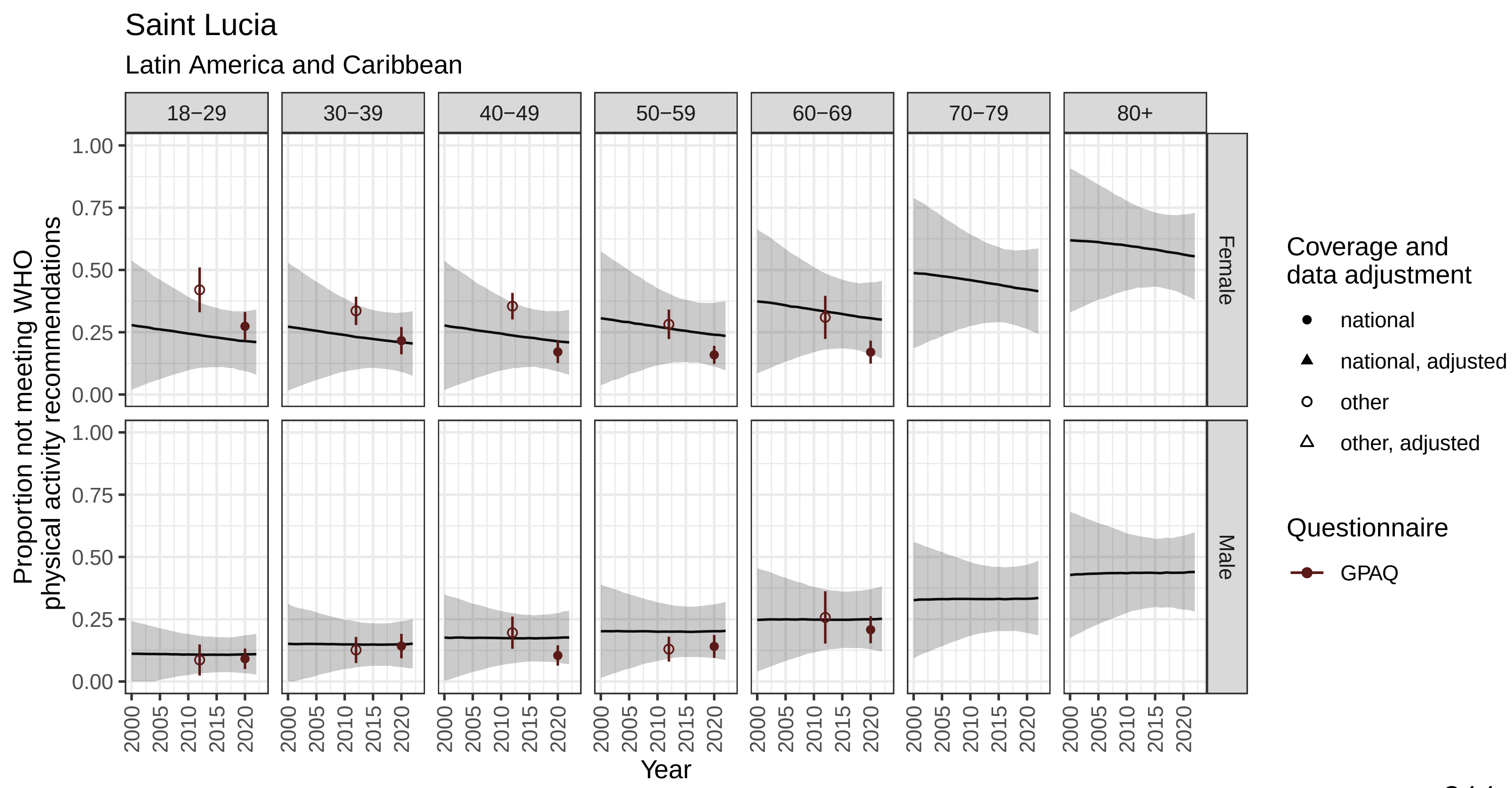

Notes: vertical lines show input data 95% confidence interval; black line shows estimate; shaded area shows 95% uncertainty interval of estimate

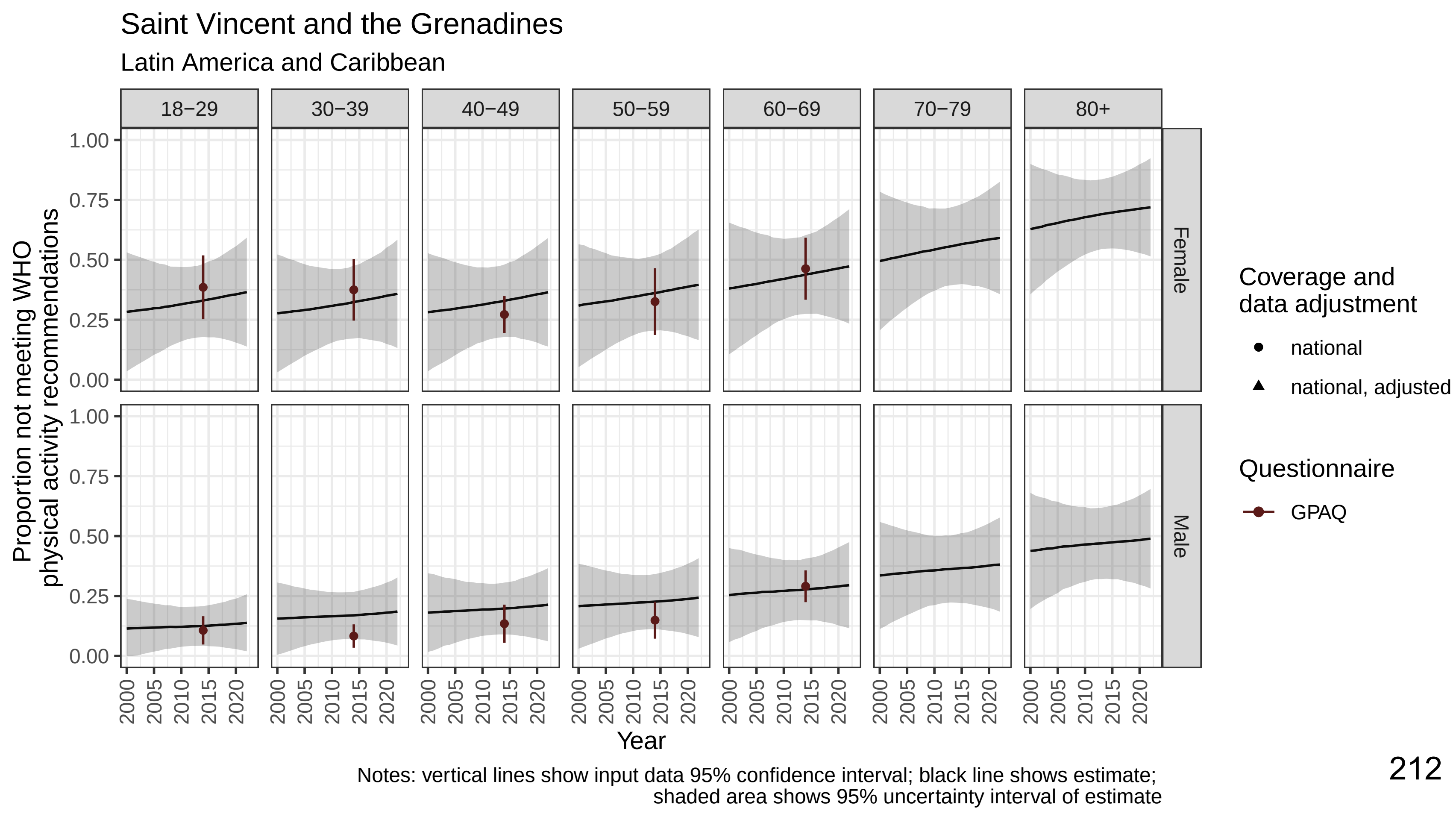
Saint Vincent and the Grenadines
Latin America and Caribbean
18−29
30−39
40−49
50−59
60−69
70−79
80+
Female
Male
Proportion not meeting WHO physical activity recommendations
Year
Coverage and data adjustment
national
national, adjusted
Questionnaire
GPAQ
Notes: vertical lines show input data 95% confidence interval; black line shows estimate; shaded area shows 95% uncertainty interval of estimate

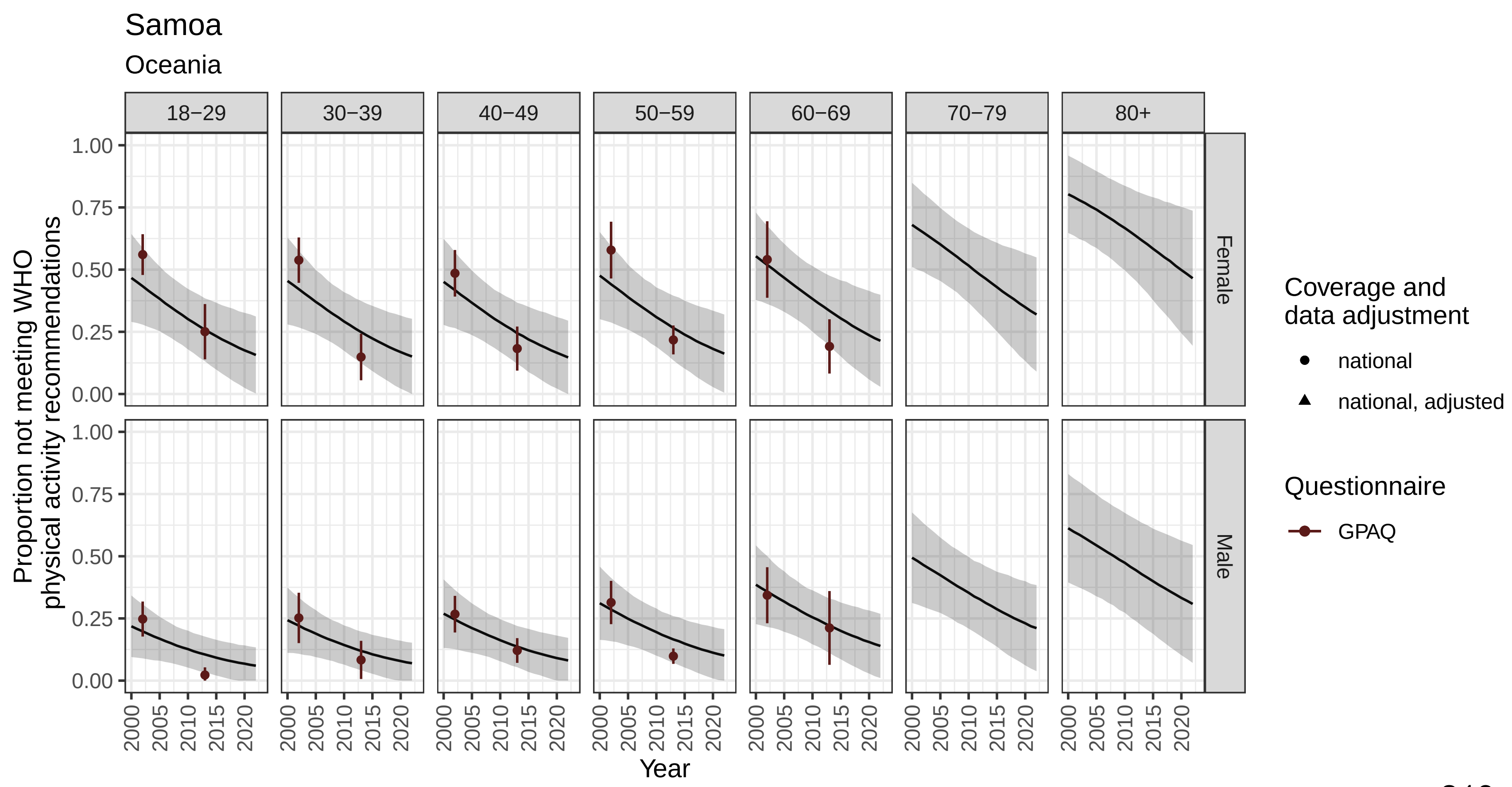

Notes: vertical lines show input data 95% confidence interval; black line shows estimate; shaded area shows 95% uncertainty interval of estimate

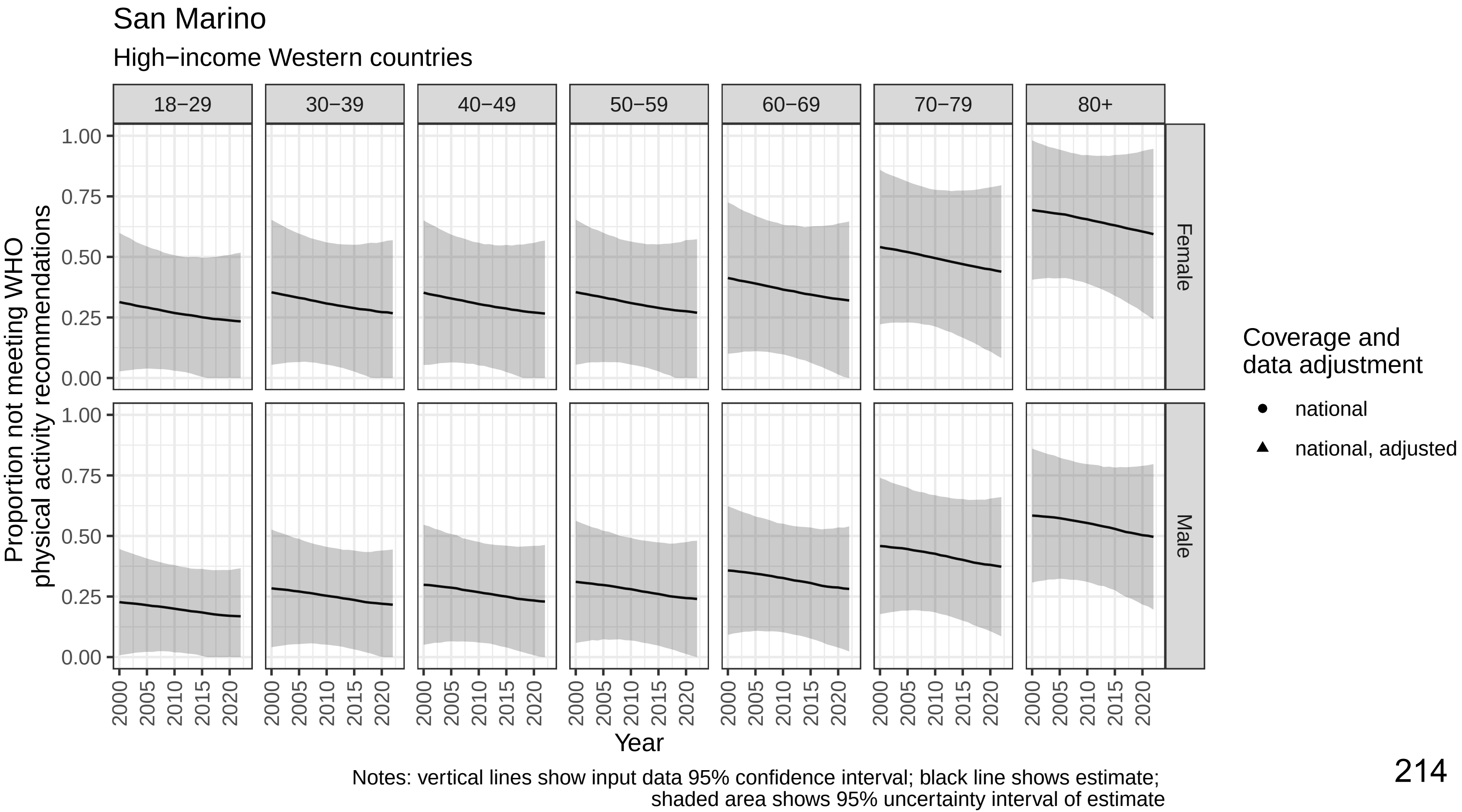
San Marino
High−income Western countries
18−29
30−39
40−49
50−59
60−69
70−79
80+
Female
Male
Proportion not meeting WHO physical activity recommendations
1.00
0.75
0.50
0.25
0.00
2000
2005
2010
2015
2020
Year
Coverage and data adjustment
national
national, adjusted
Notes: vertical lines show input data 95% confidence interval; black line shows estimate;
shaded area shows 95% uncertainty interval of estimate

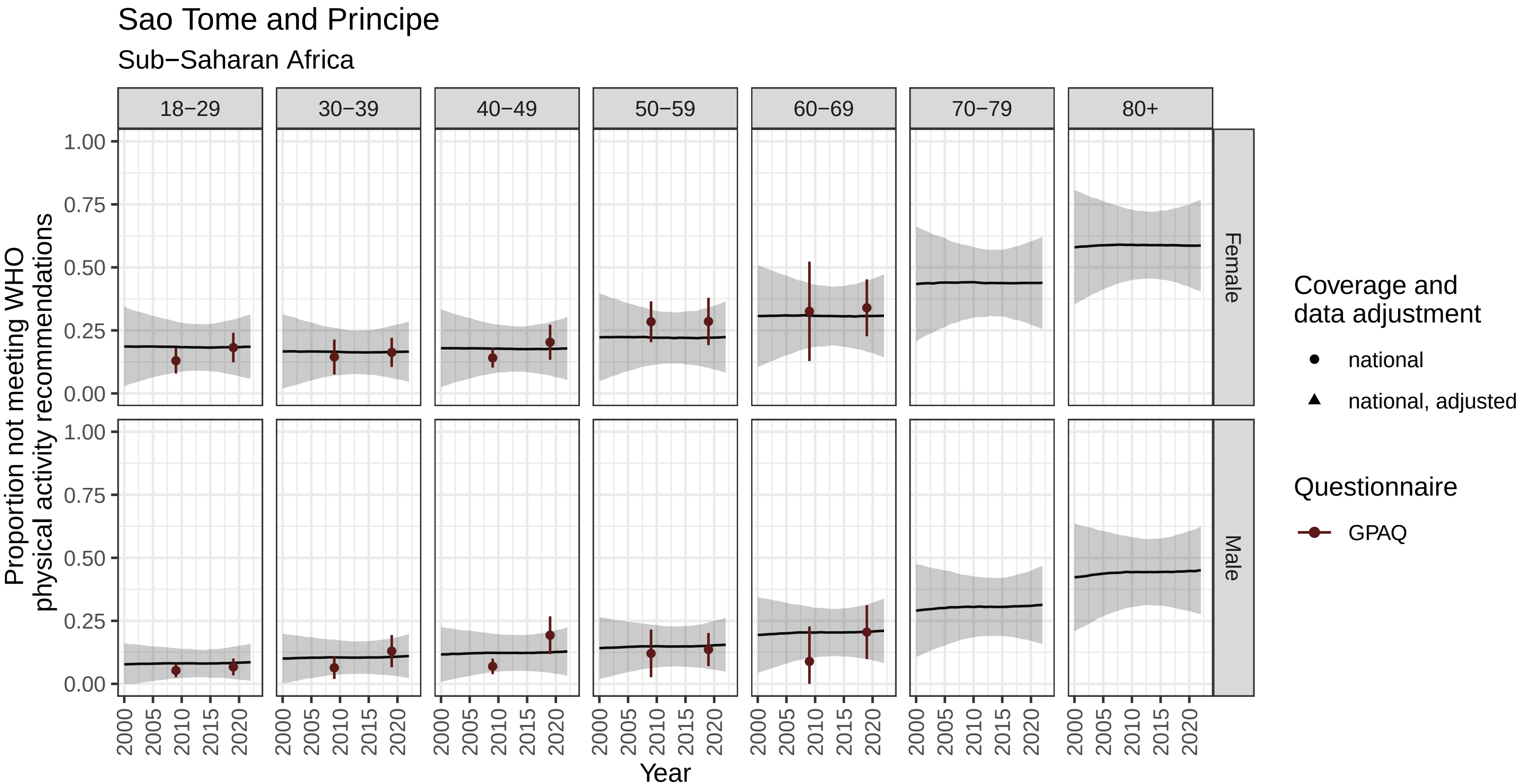

Notes: vertical lines show input data 95% confidence interval; black line shows estimate; shaded area shows 95% uncertainty interval of estimate

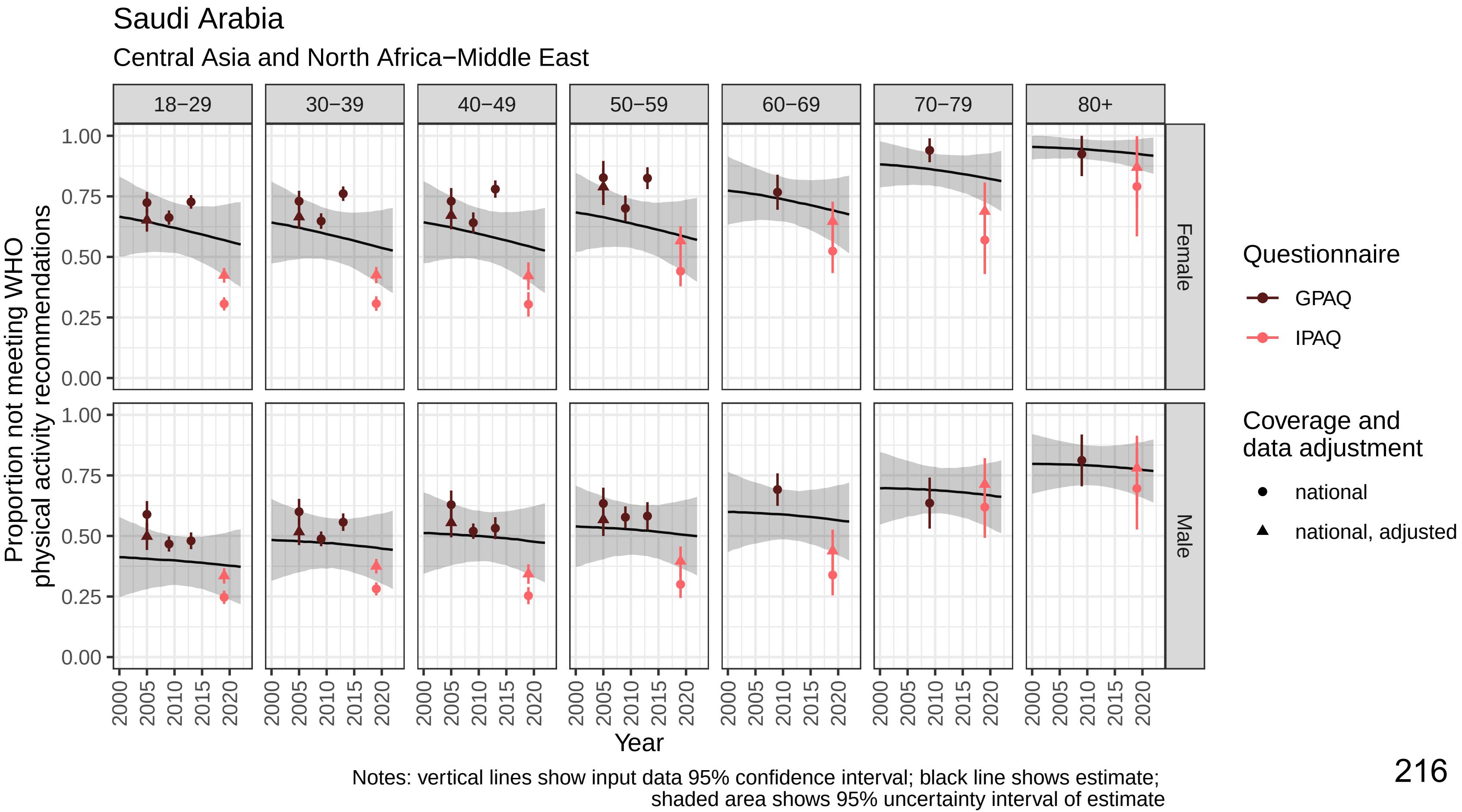

Saudi Arabia
Central Asia and North Africa−Middle East
18−29
30−39
40−49
50−59
60−69
70−79
80+
Female
Male
Proportion not meeting WHO physical activity recommendations
1.00
0.75
0.50
0.25
0.00
2000
2005
2010
2015
2020
Year
Questionnaire
GPAQ
IPAQ
Coverage and data adjustment
national
national, adjusted
Notes: vertical lines show input data 95% confidence interval; black line shows estimate;
shaded area shows 95% uncertainty interval of estimate

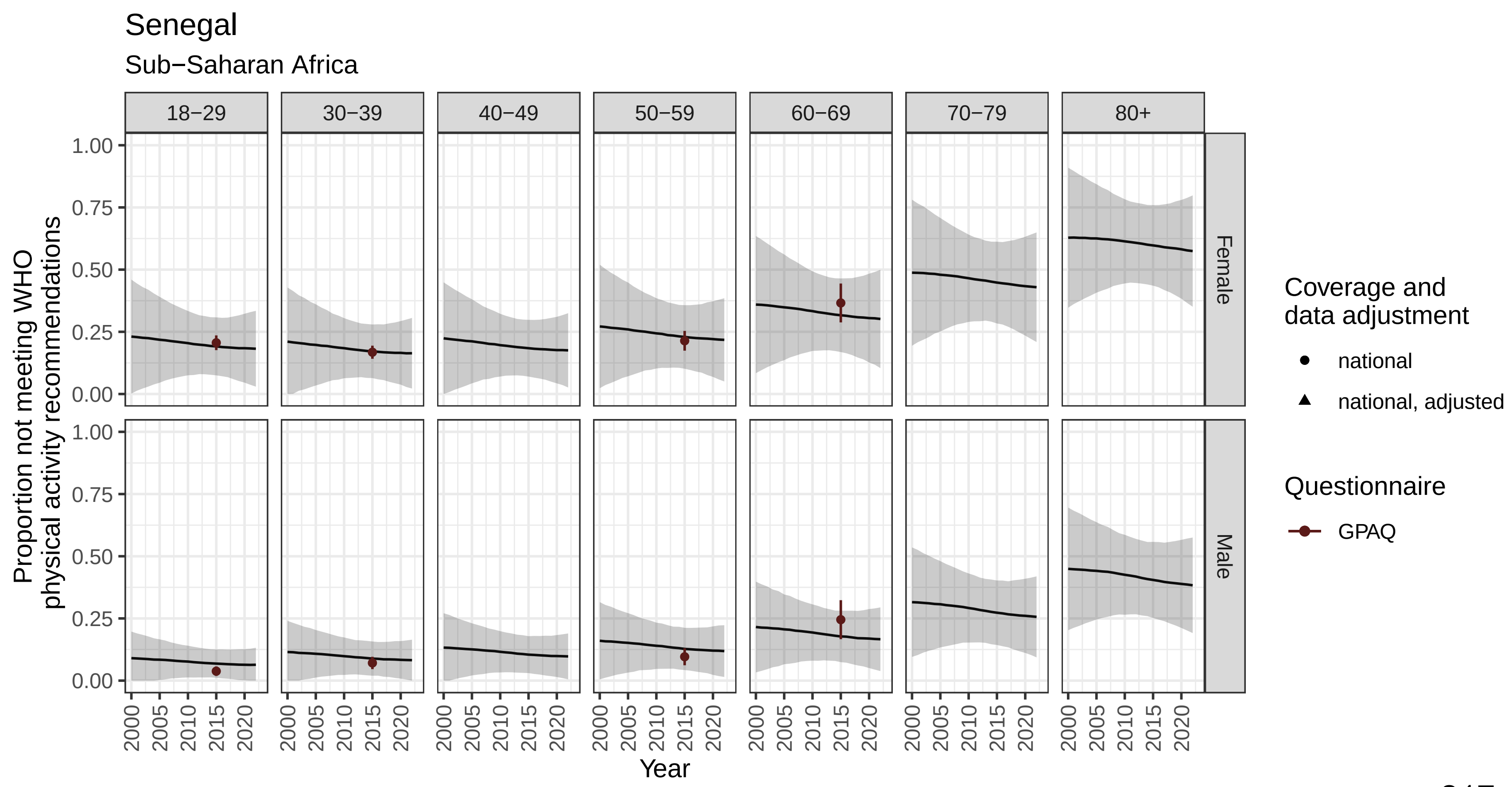

Notes: vertical lines show input data 95% confidence interval; black line shows estimate; shaded area shows 95% uncertainty interval of estimate

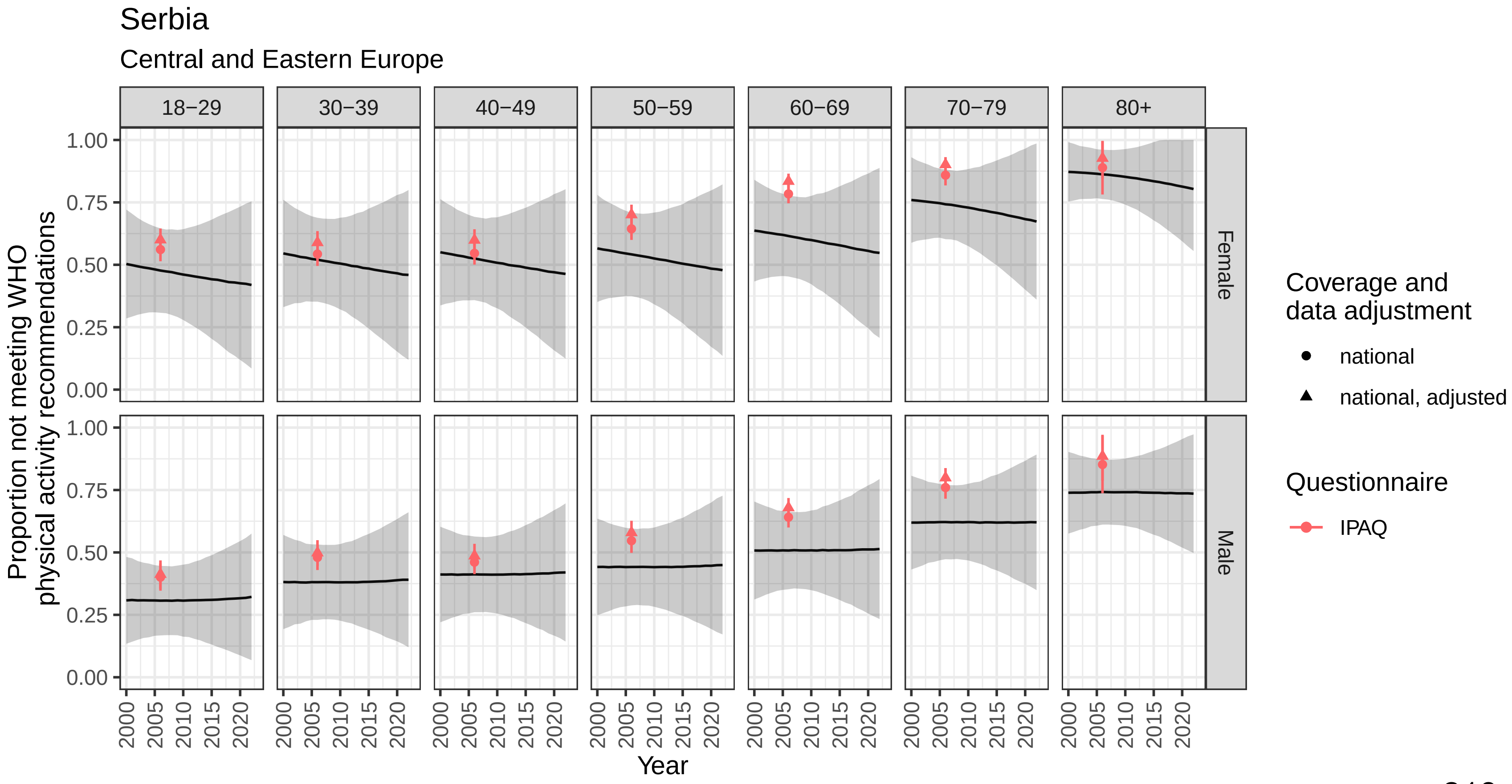

Notes: vertical lines show input data 95% confidence interval; black line shows estimate; shaded area shows 95% uncertainty interval of estimate

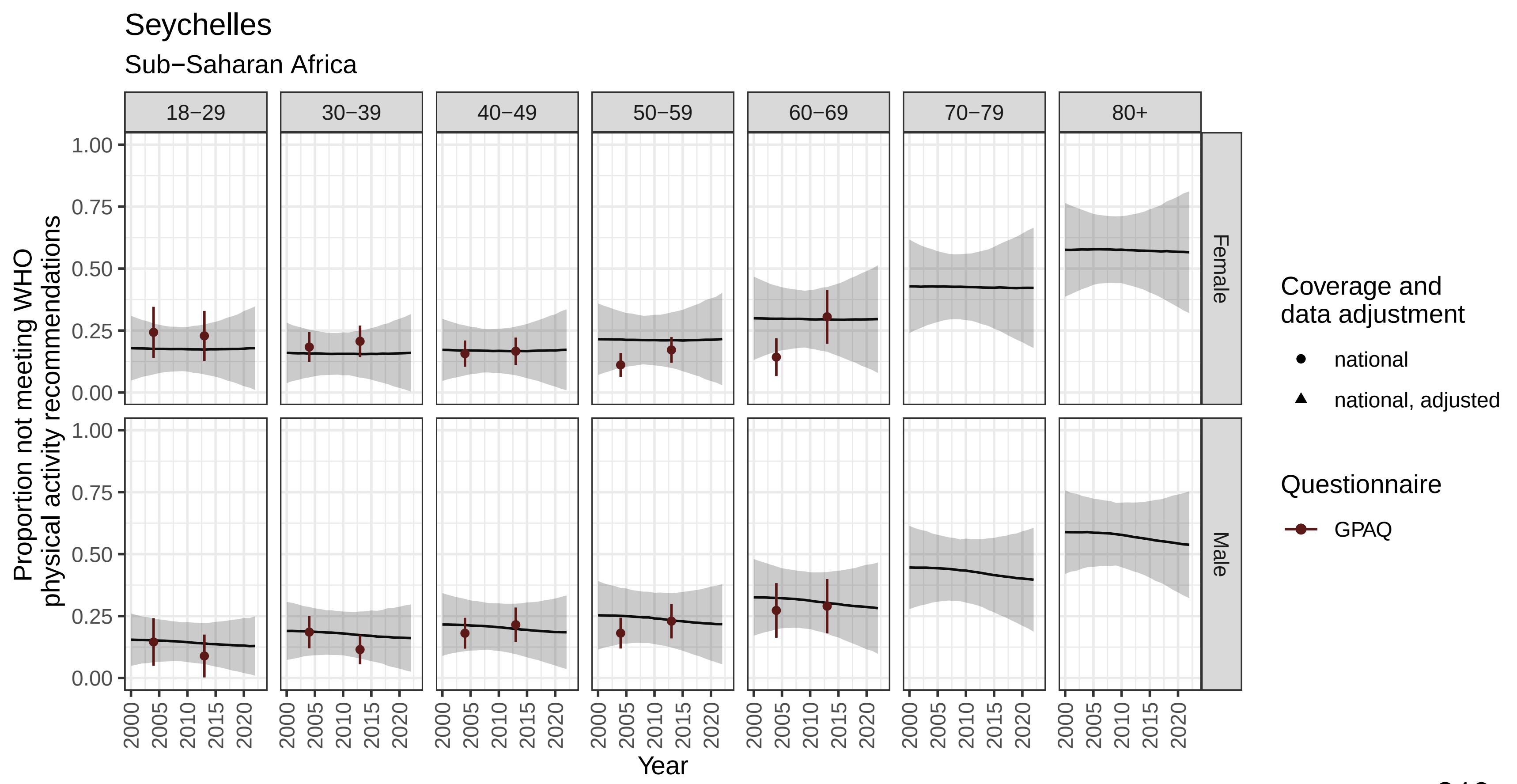

Notes: vertical lines show input data 95% confidence interval; black line shows estimate; shaded area shows 95% uncertainty interval of estimate

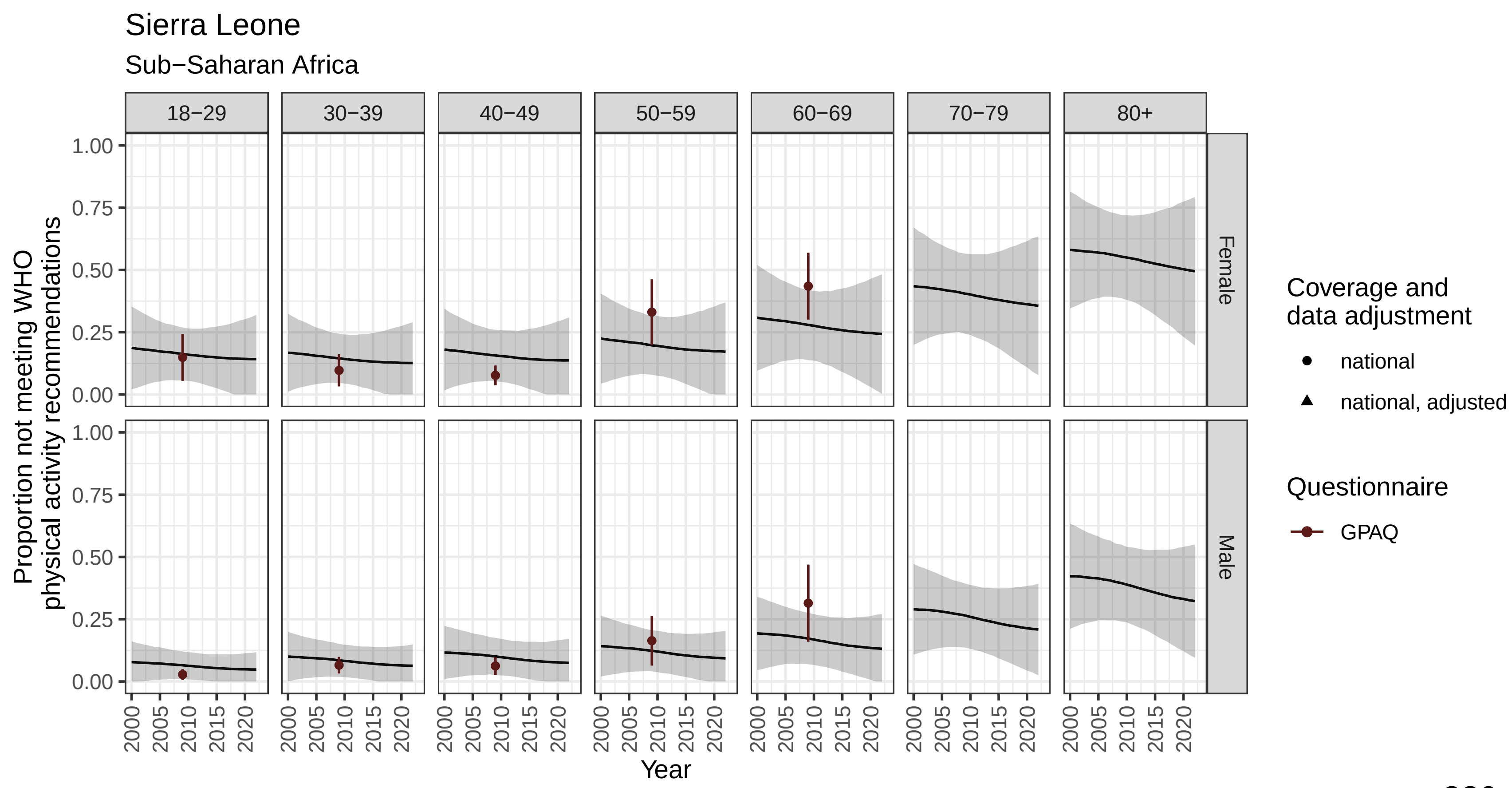

Notes: vertical lines show input data 95% confidence interval; black line shows estimate; shaded area shows 95% uncertainty interval of estimate

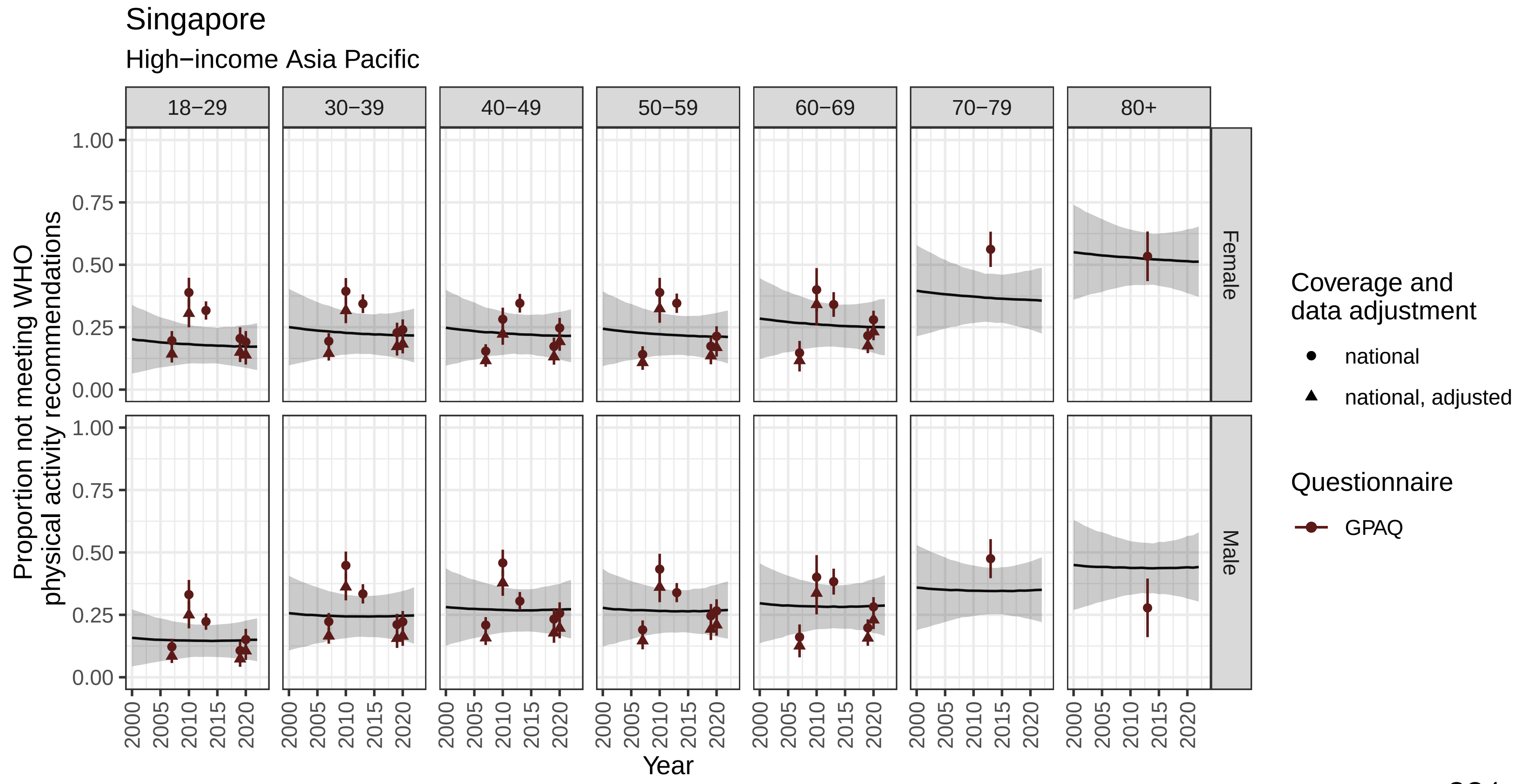

Notes: vertical lines show input data 95% confidence interval; black line shows estimate; shaded area shows 95% uncertainty interval of estimate

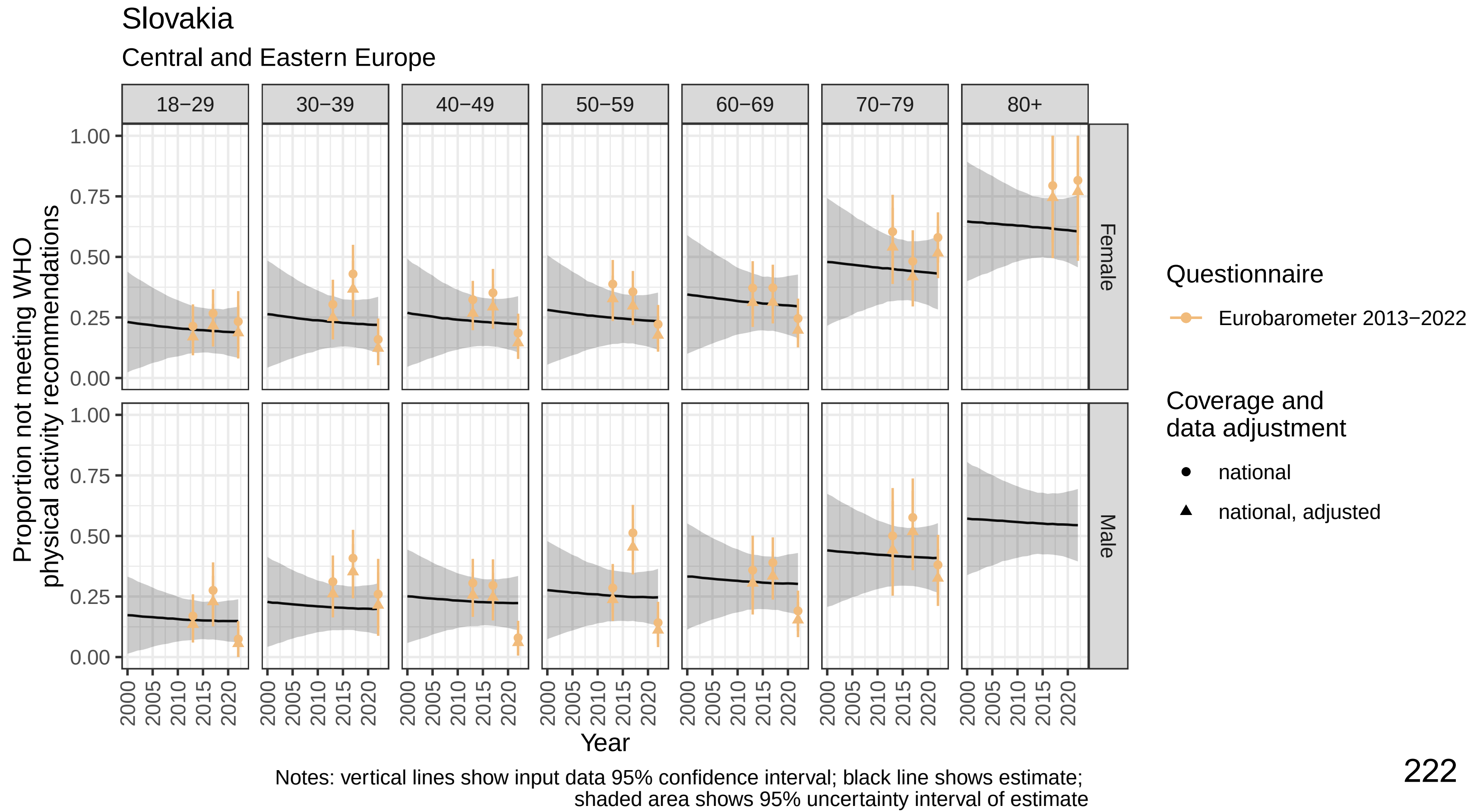
Slovakia
Central and Eastern Europe
18−29
30−39
40−49
50−59
60−69
70−79
80+
Female
Male
Proportion not meeting WHO physical activity recommendations
1.00
0.75
0.50
0.25
0.00
2000
2005
2010
2015
2020
Year
Questionnaire
Eurobarometer 2013−2022
Coverage and data adjustment
national
national, adjusted
Notes: vertical lines show input data 95% confidence interval; black line shows estimate; shaded area shows 95% uncertainty interval of estimate

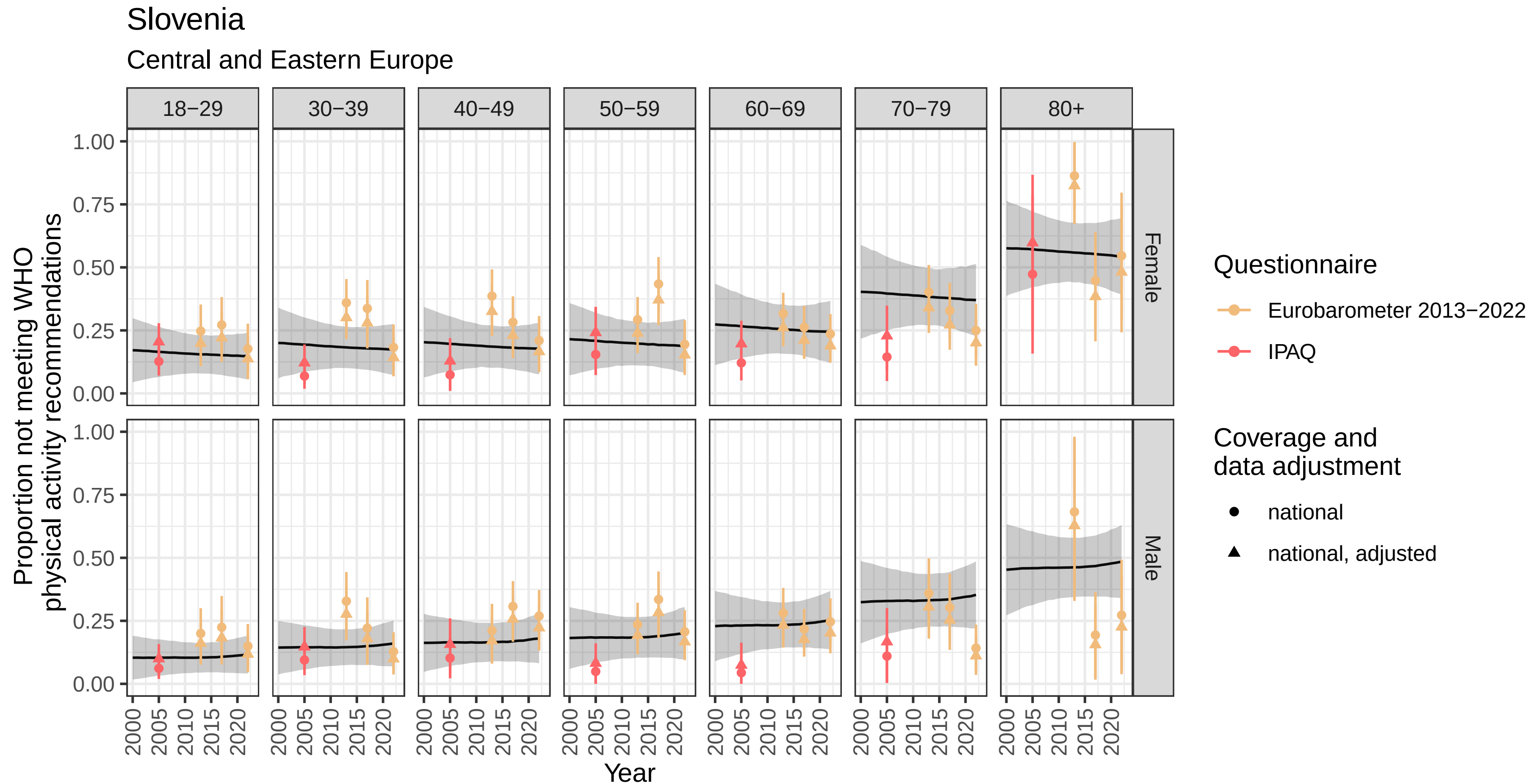

Notes: vertical lines show input data 95% confidence interval; black line shows estimate; shaded area shows 95% uncertainty interval of estimate

Solomon Islands

Oceania

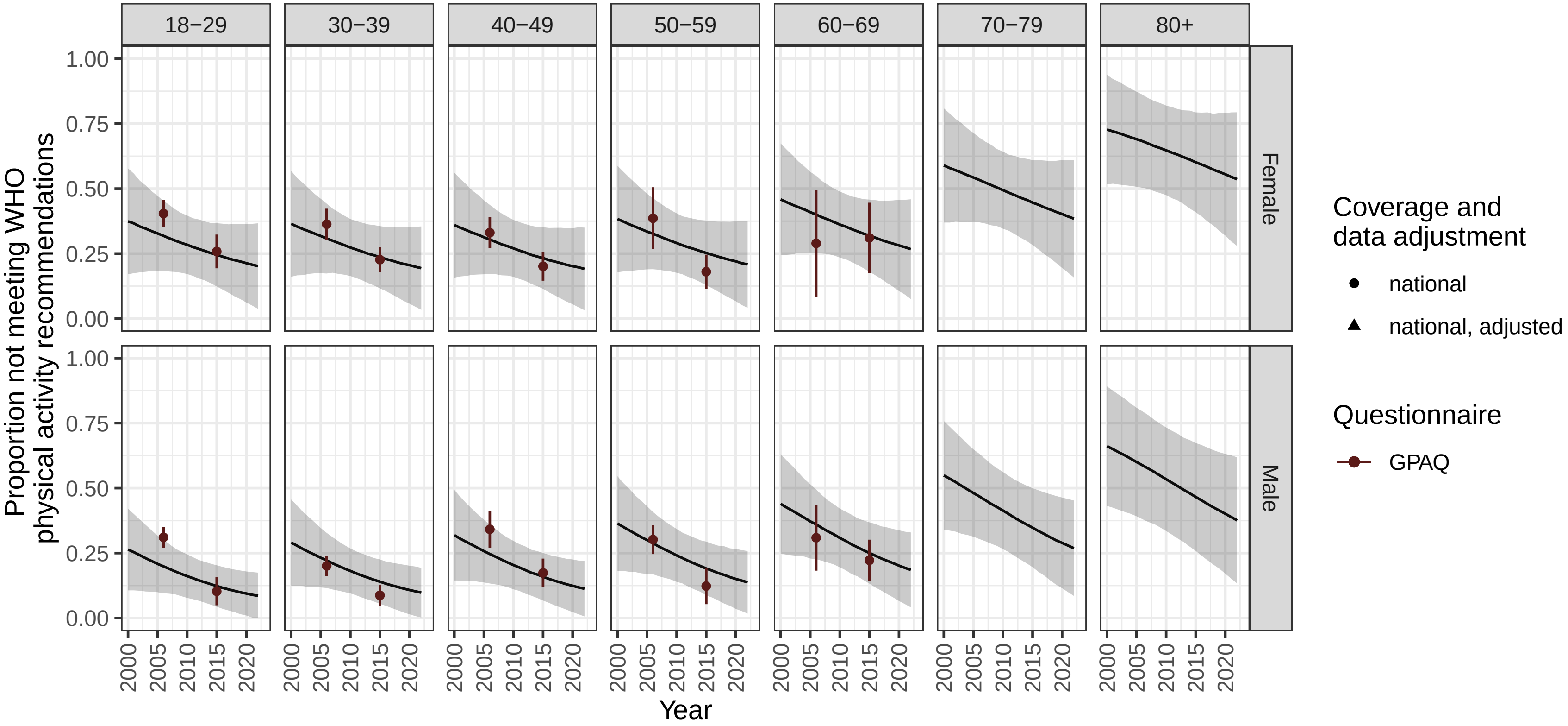

Notes: vertical lines show input data 95% confidence interval; black line shows estimate; shaded area shows 95% uncertainty interval of estimate

Somalia

Sub−Saharan Africa

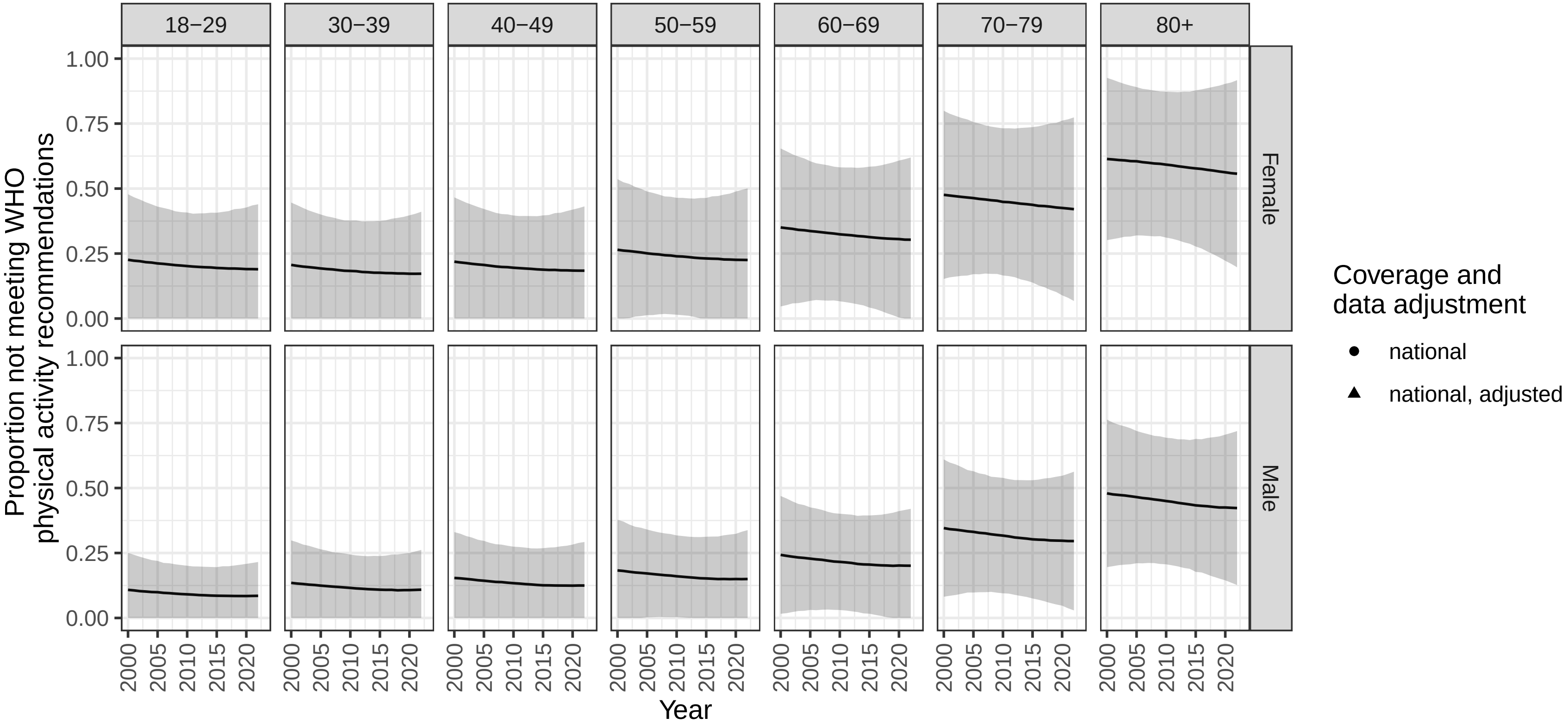

Notes: vertical lines show input data 95% confidence interval; black line shows estimate; shaded area shows 95% uncertainty interval of estimate

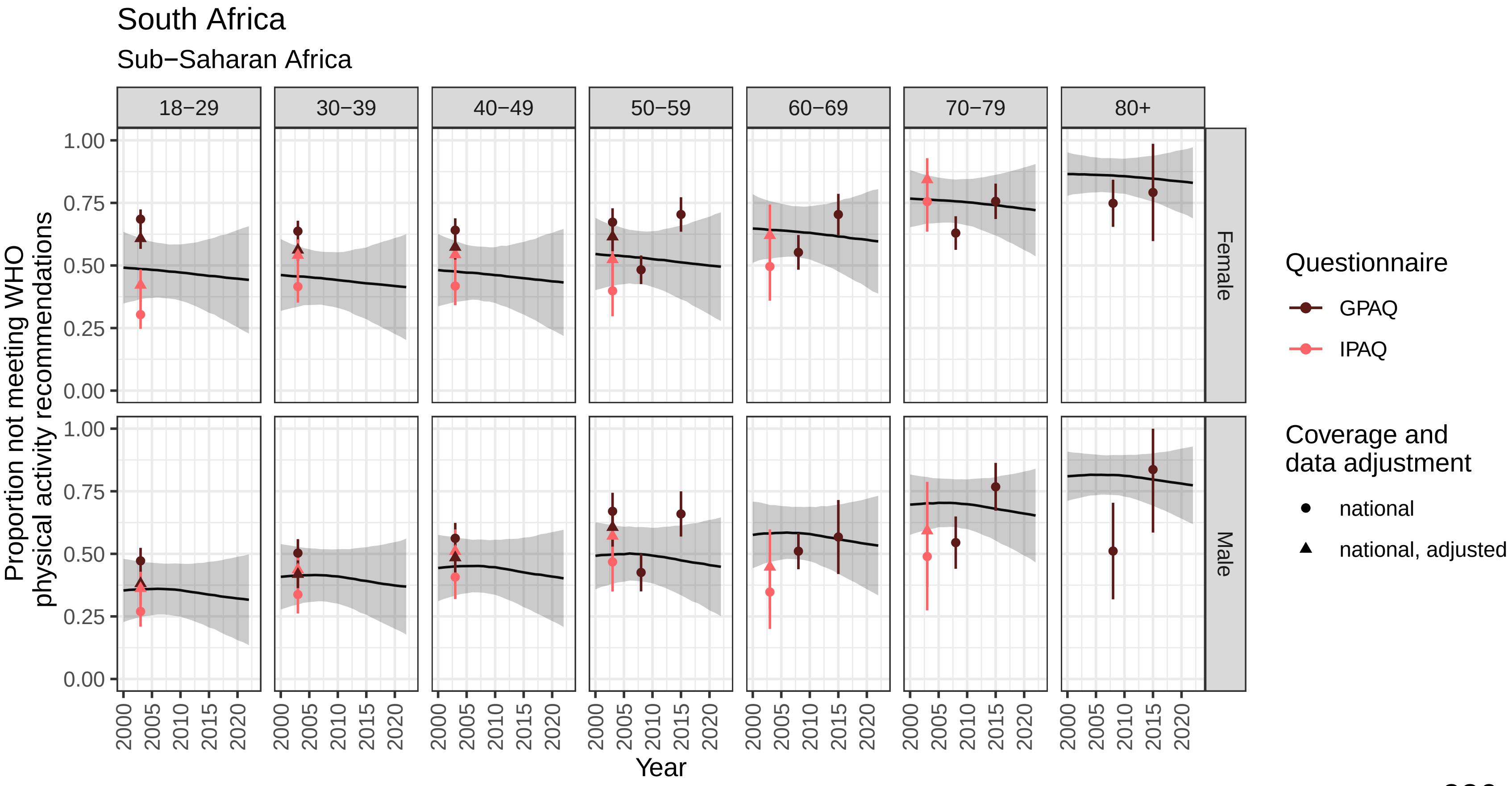

Notes: vertical lines show input data 95% confidence interval; black line shows estimate; shaded area shows 95% uncertainty interval of estimate

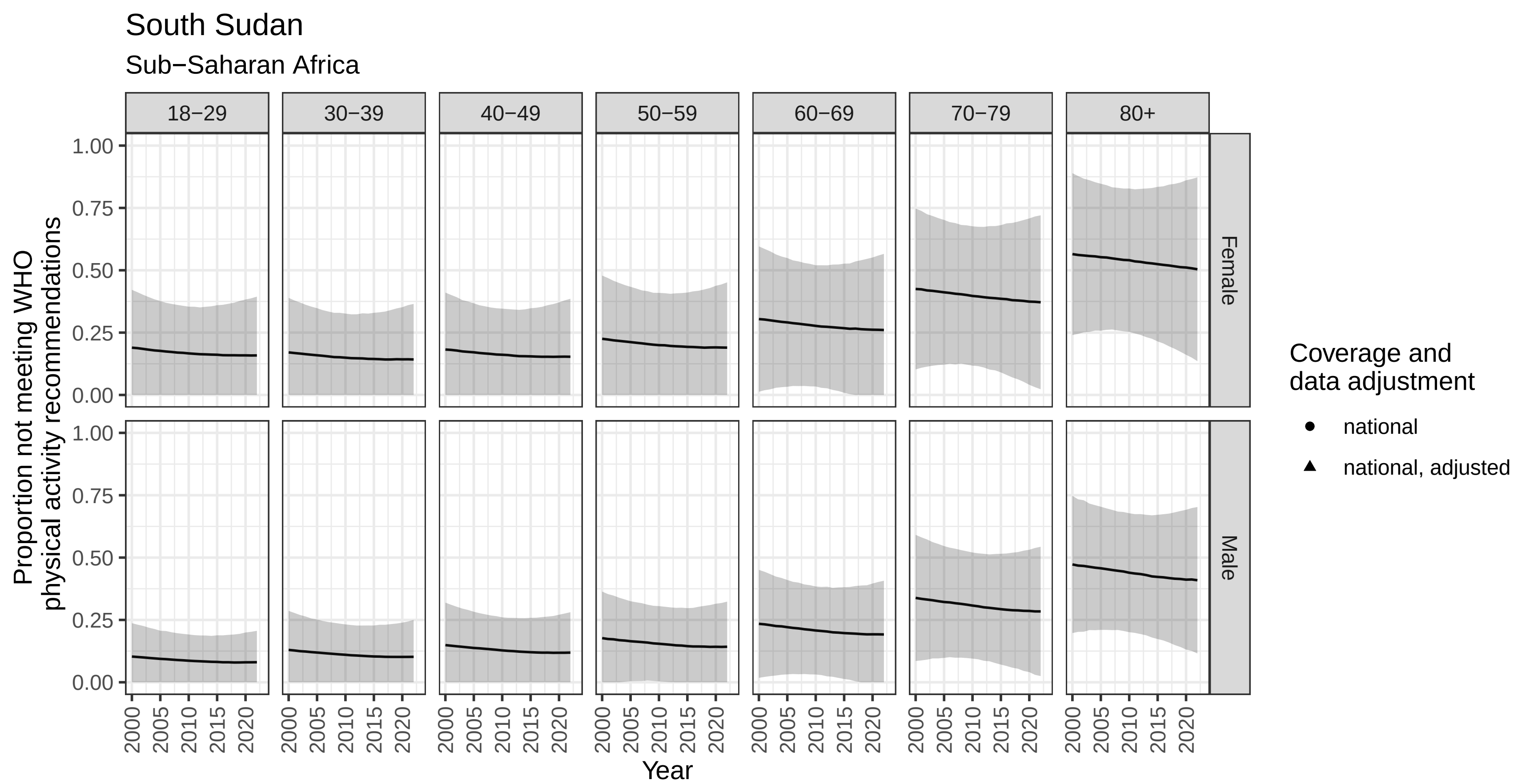

Notes: vertical lines show input data 95% confidence interval; black line shows estimate; shaded area shows 95% uncertainty interval of estimate

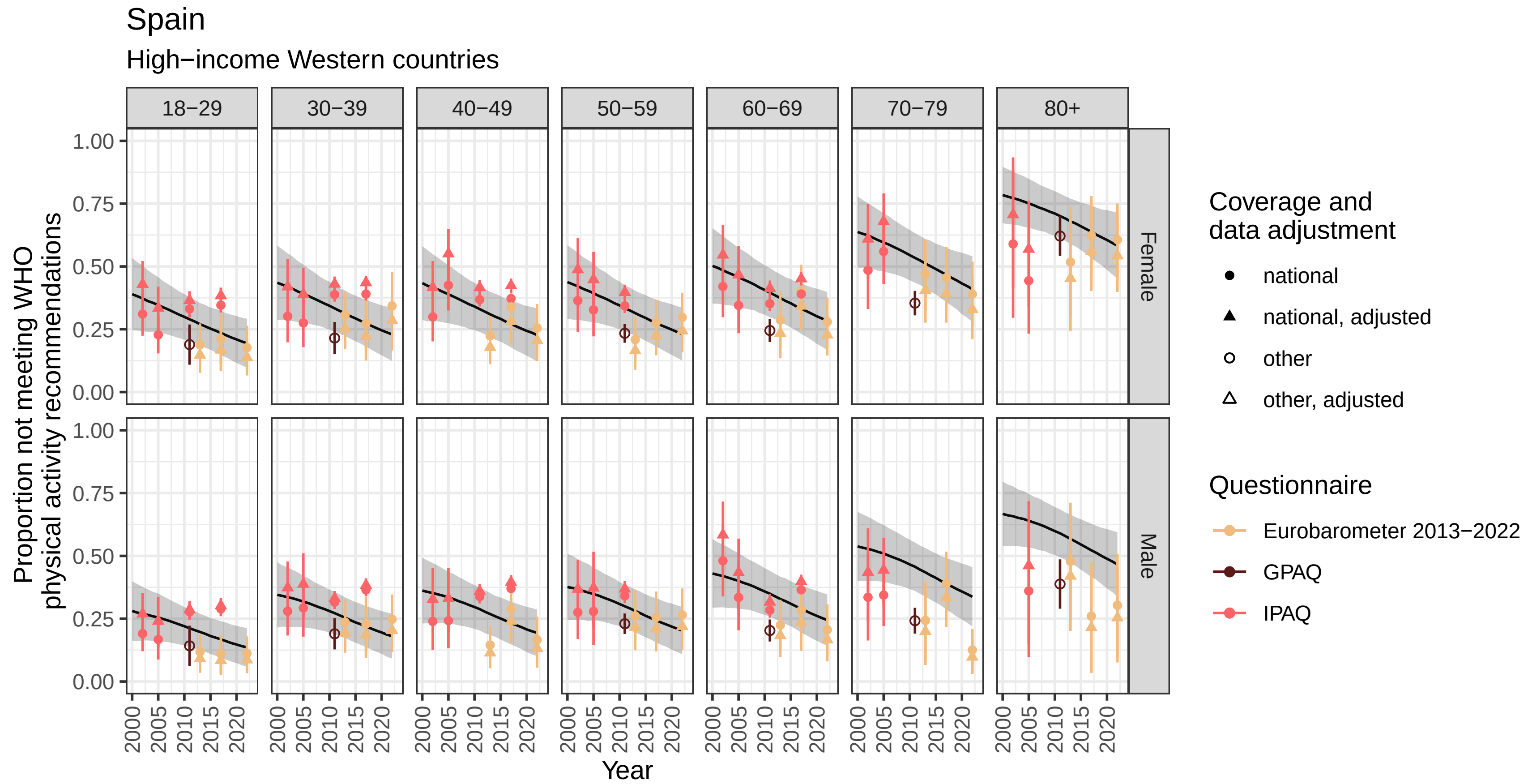

Notes: vertical lines show input data 95% confidence interval; black line shows estimate; shaded area shows 95% uncertainty interval of estimate

Sri Lanka

South Asia

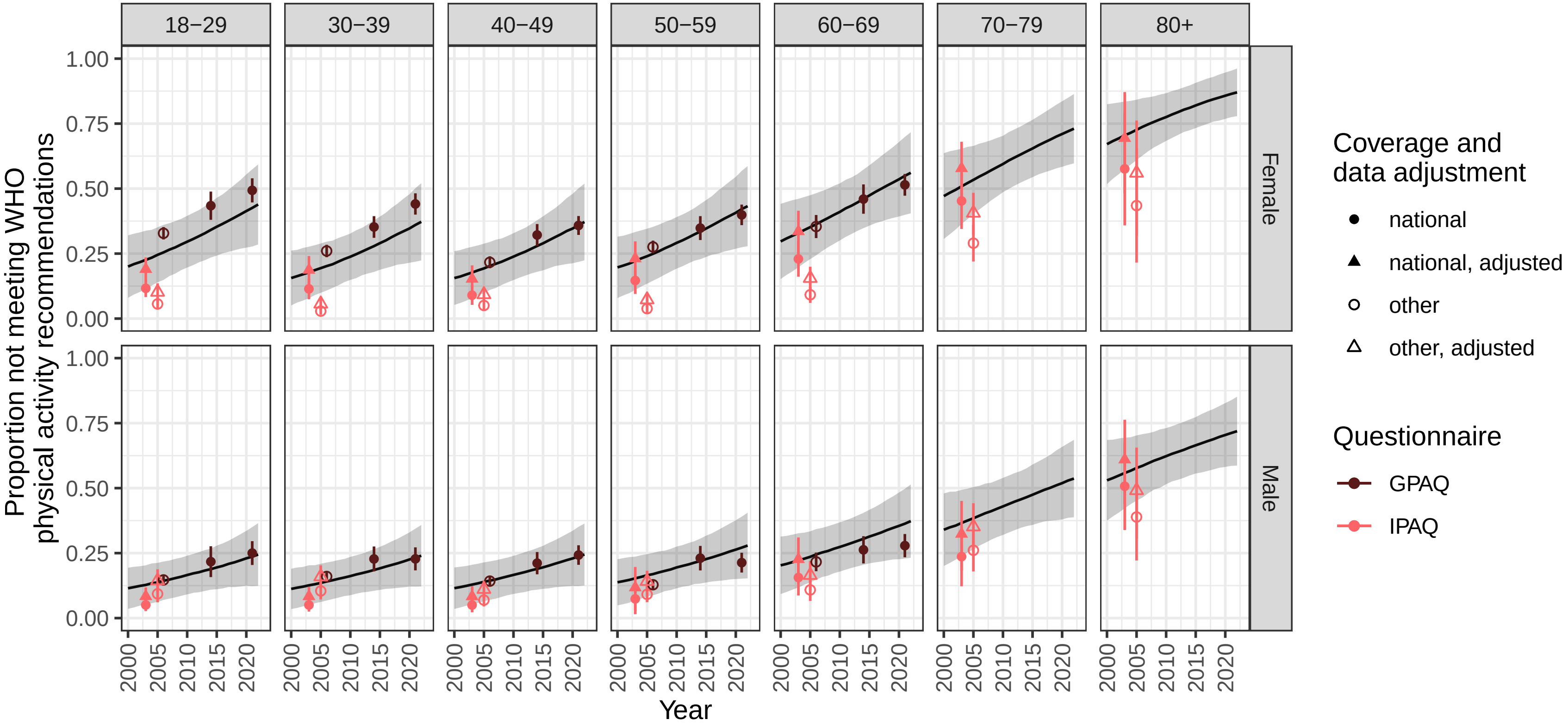

Notes: vertical lines show input data 95% confidence interval; black line shows estimate; shaded area shows 95% uncertainty interval of estimate

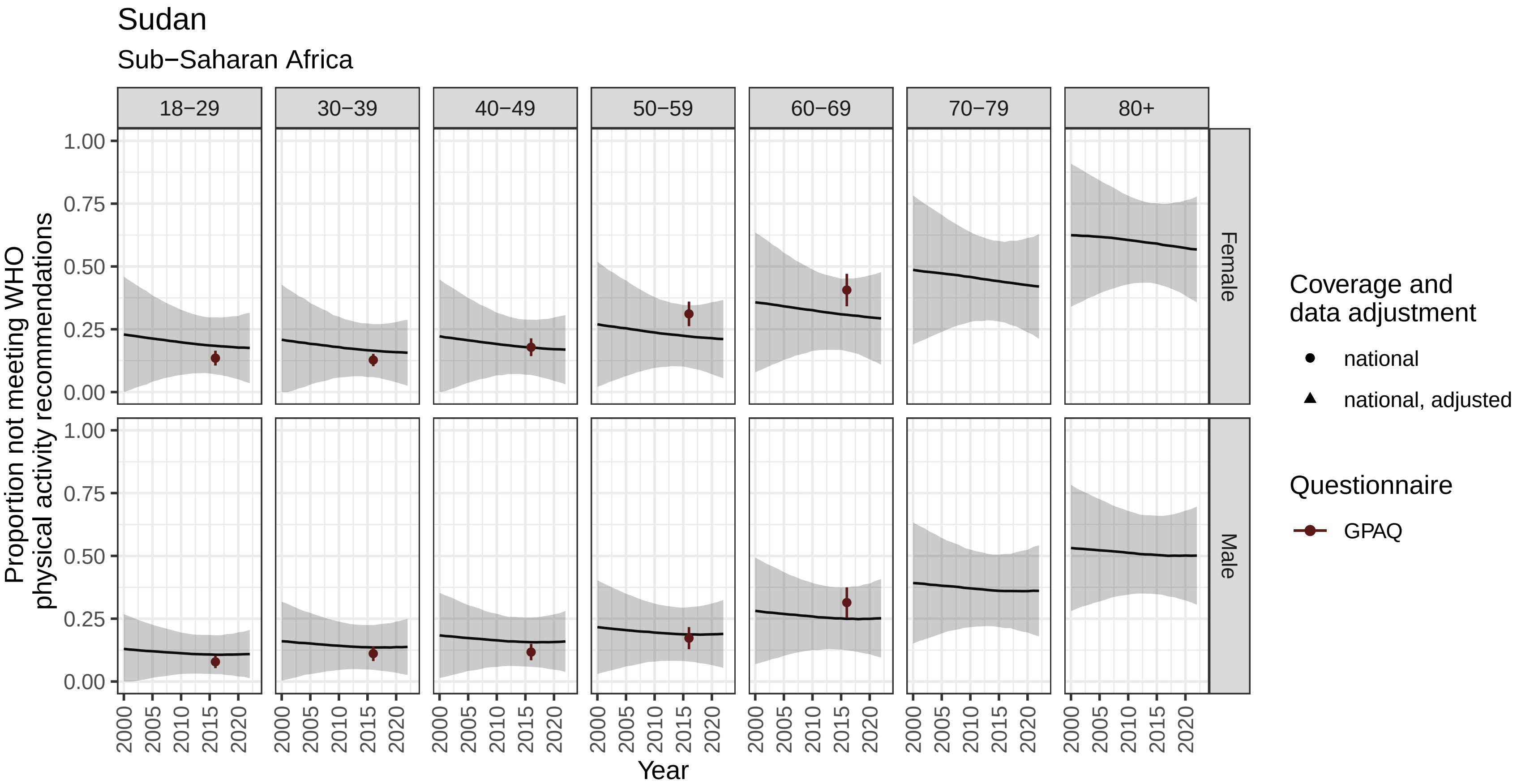

Notes: vertical lines show input data 95% confidence interval; black line shows estimate; shaded area shows 95% uncertainty interval of estimate

Suriname

Latin America and Caribbean

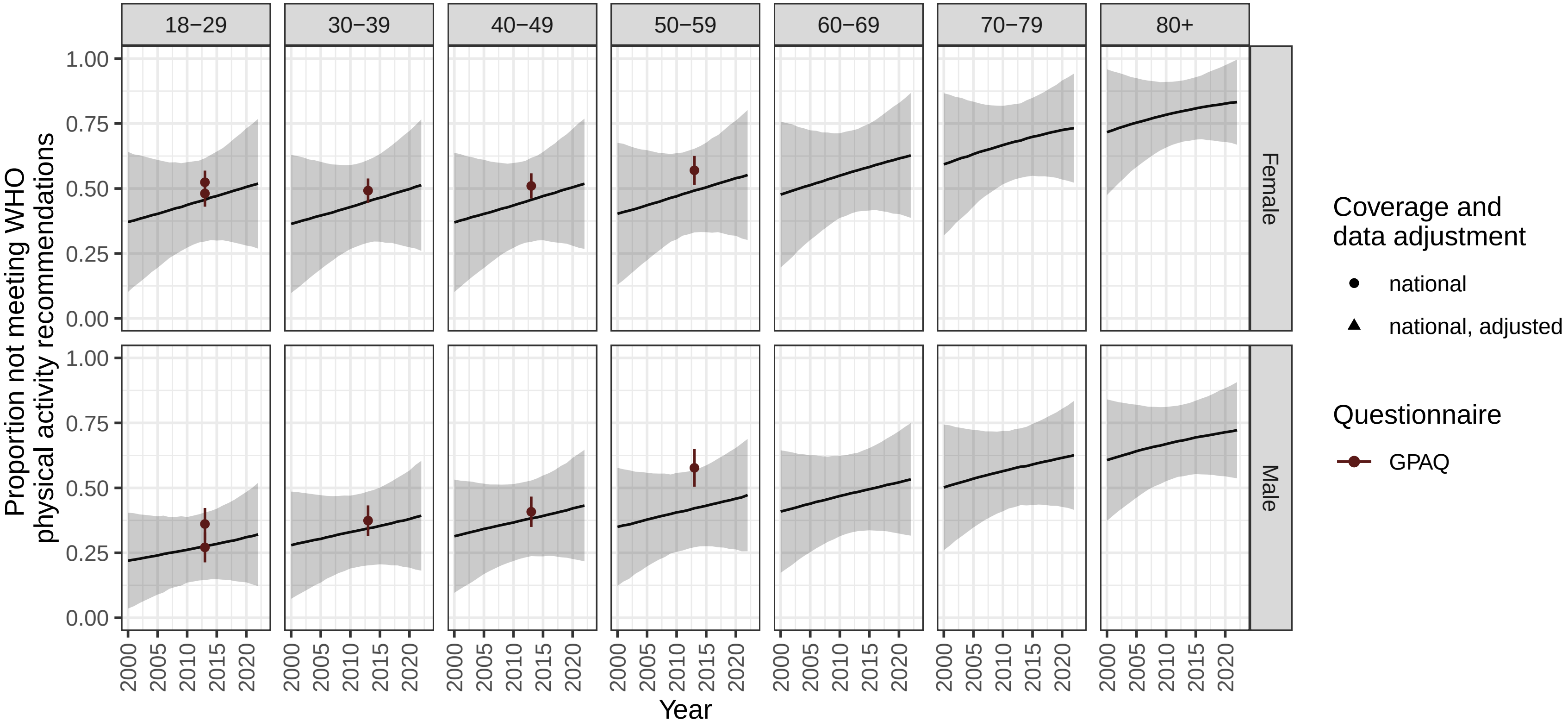

Notes: vertical lines show input data 95% confidence interval; black line shows estimate; shaded area shows 95% uncertainty interval of estimate

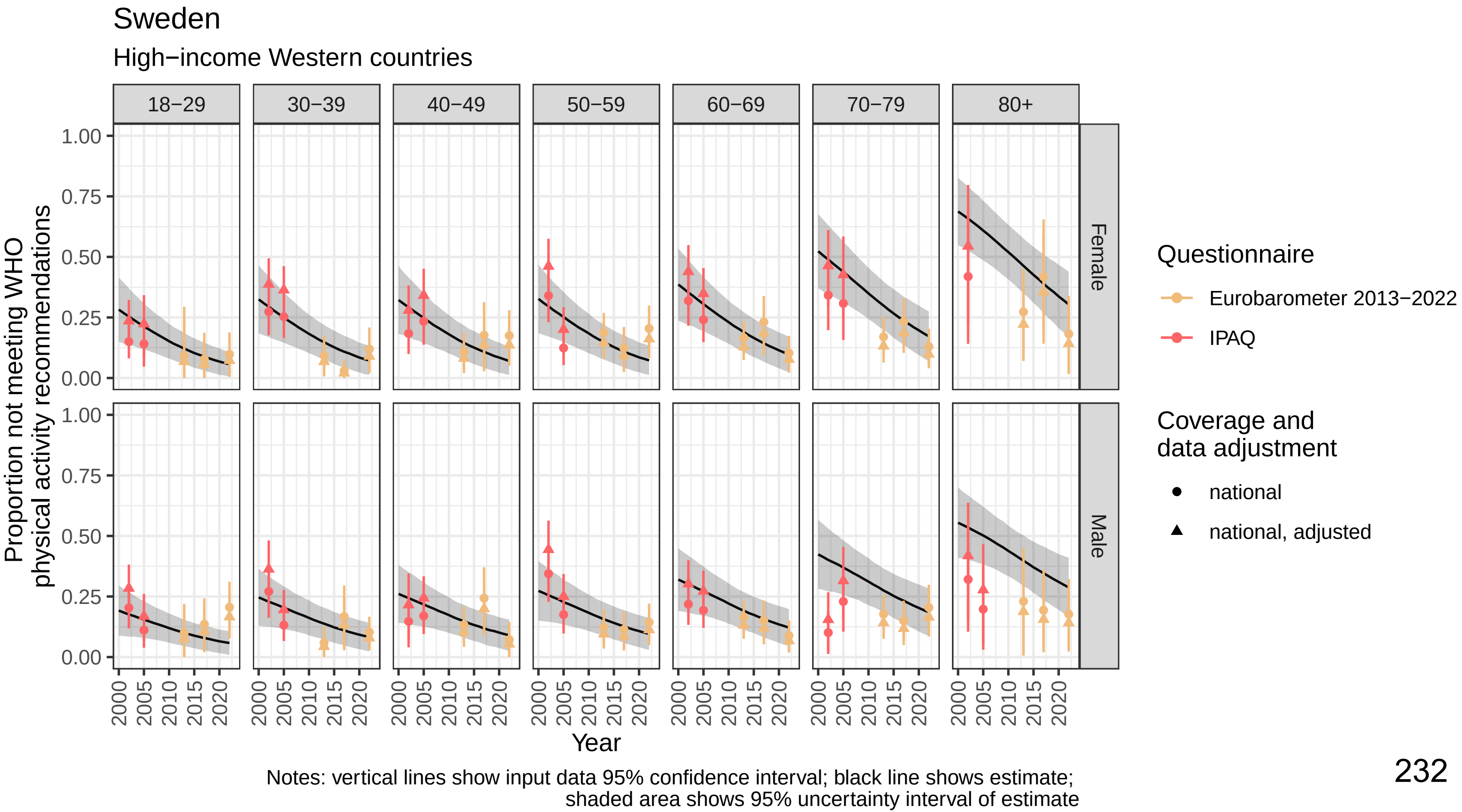
Sweden
High−income Western countries
18−29
30−39
40−49
50−59
60−69
70−79
80+
Female
Male
Proportion not meeting WHO physical activity recommendations
1.00
0.75
0.50
0.25
0.00
2000
2005
2010
2015
2020
Year
Questionnaire
Eurobarometer 2013−2022
IPAQ
Coverage and data adjustment
national
national, adjusted
Notes: vertical lines show input data 95% confidence interval; black line shows estimate;
shaded area shows 95% uncertainty interval of estimate

Switzerland

High−income Western countries

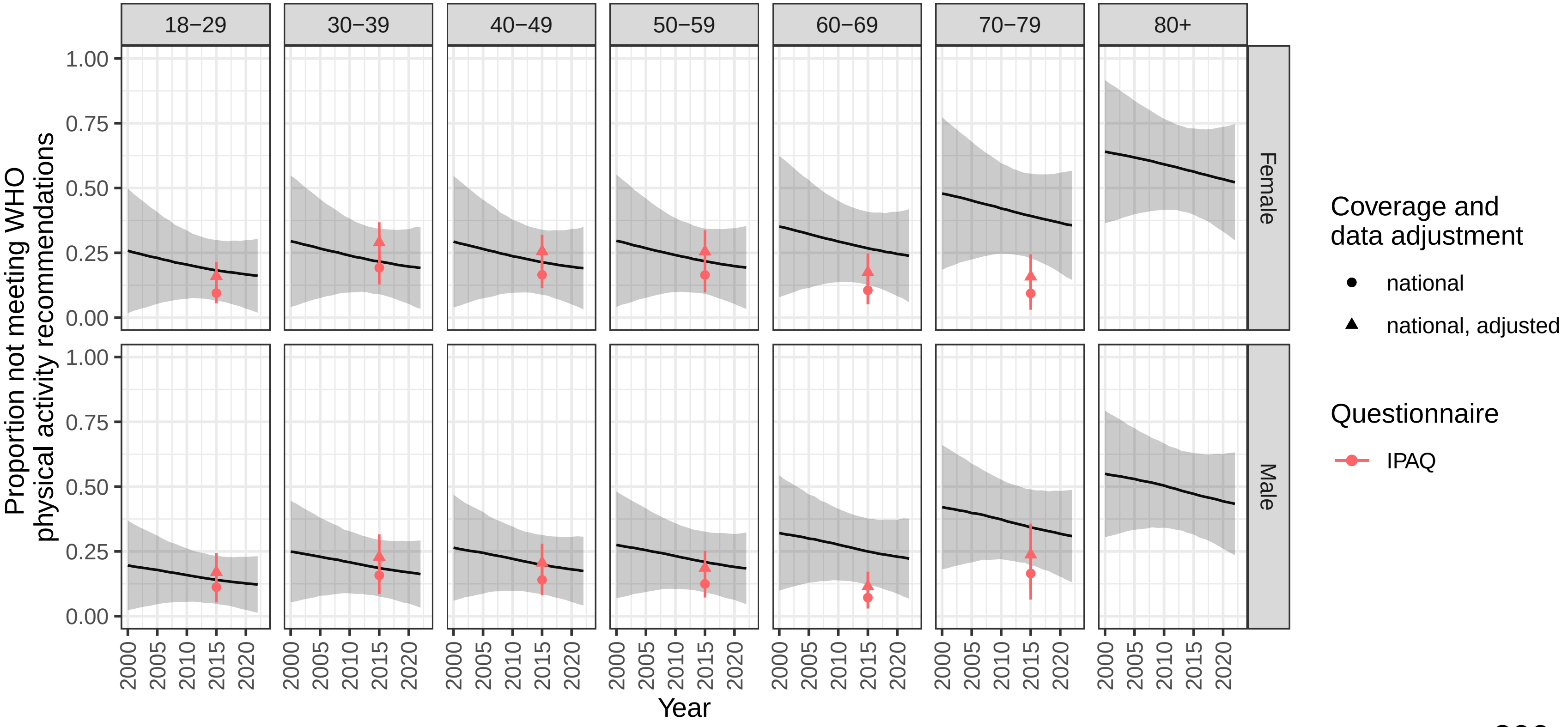

Notes: vertical lines show input data 95% confidence interval; black line shows estimate; shaded area shows 95% uncertainty interval of estimate

Syrian Arab Republic

Central Asia and North Africa−Middle East

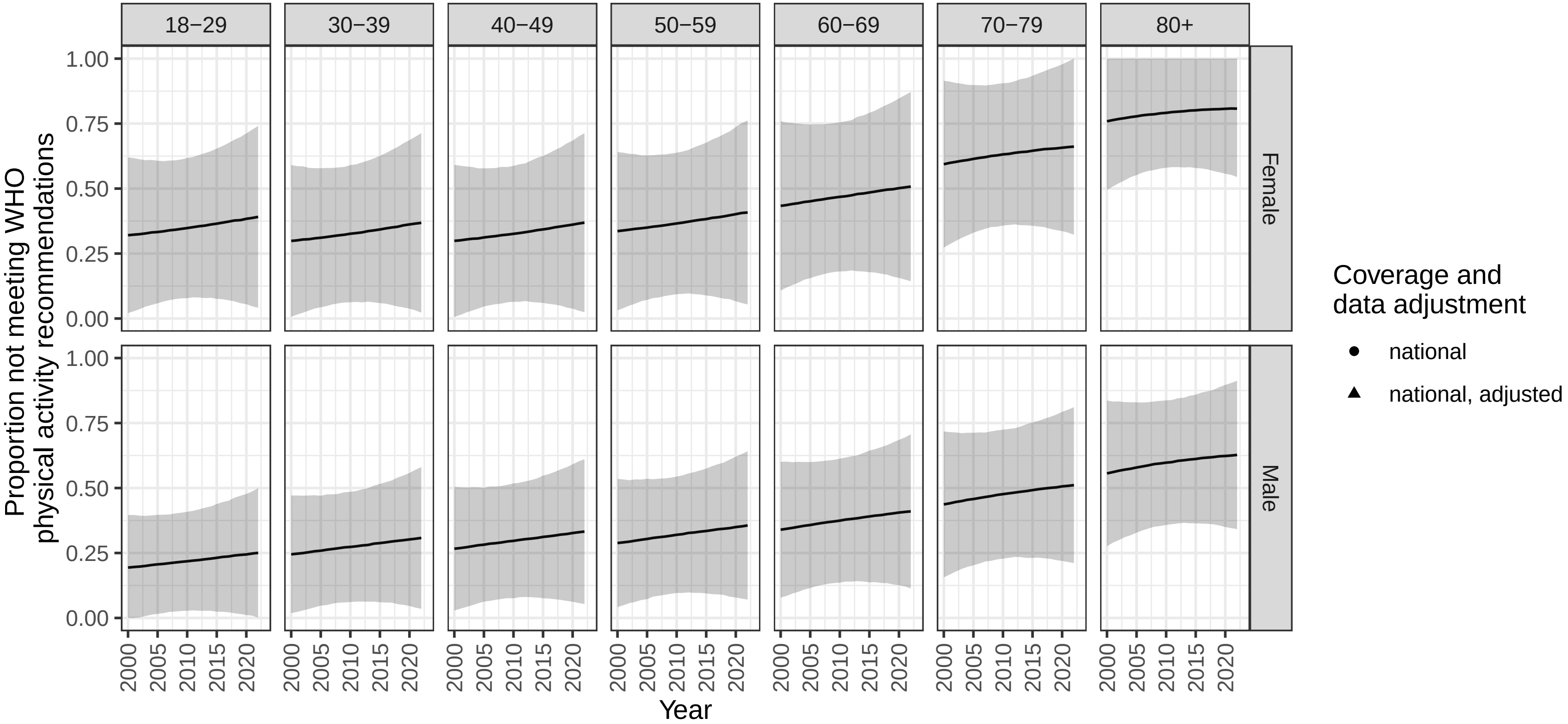

Notes: vertical lines show input data 95% confidence interval; black line shows estimate; shaded area shows 95% uncertainty interval of estimate

Taiwan, China

East and South East Asia

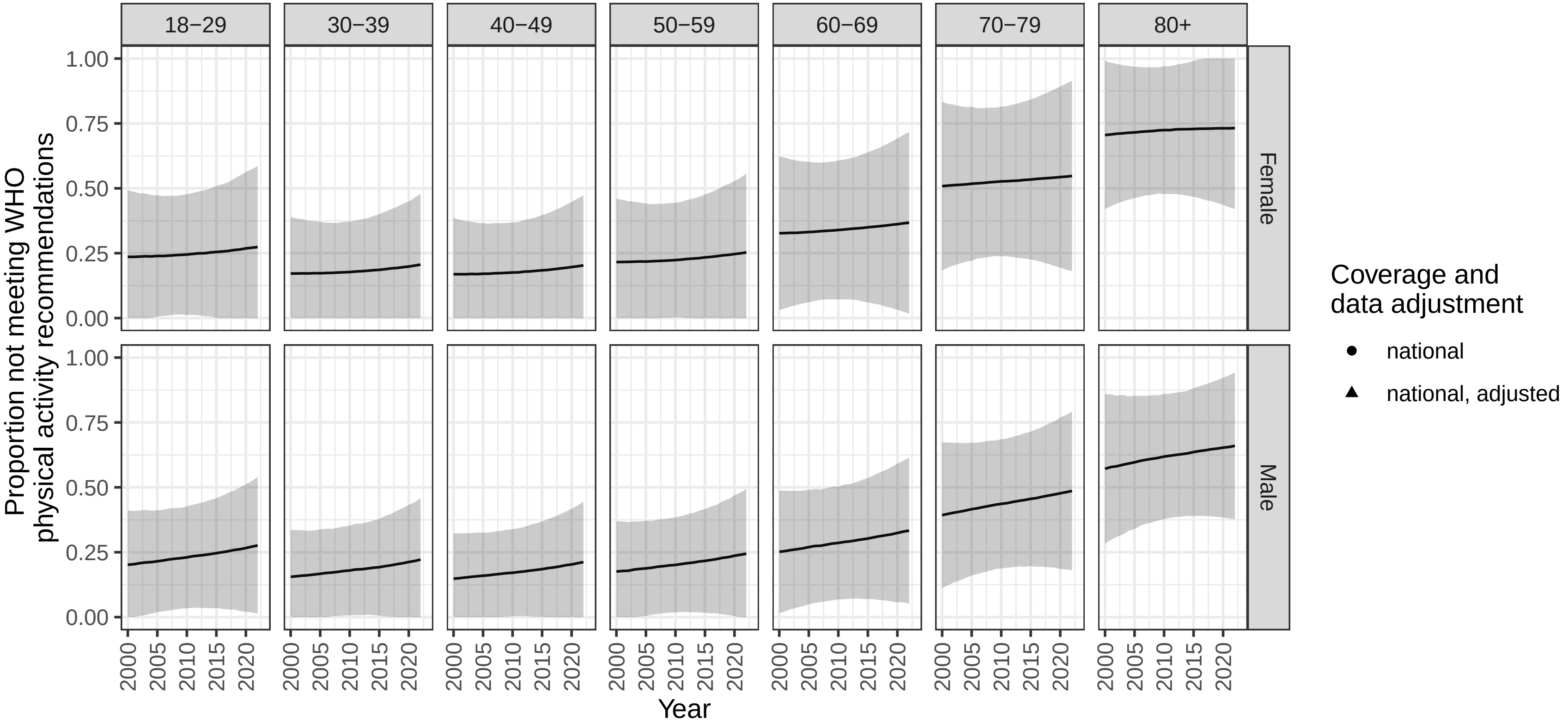

Notes: vertical lines show input data 95% confidence interval; black line shows estimate; shaded area shows 95% uncertainty interval of estimate

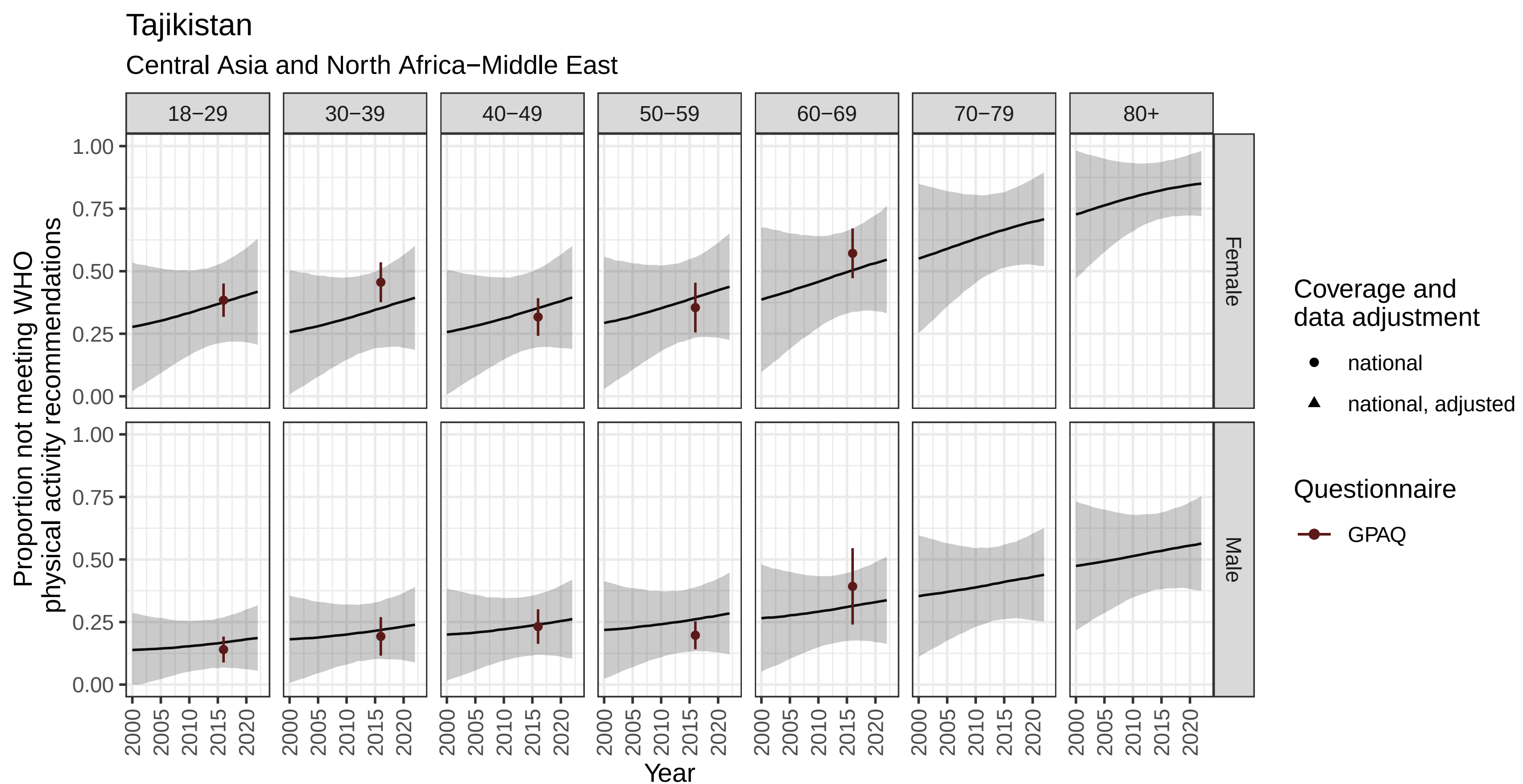

Notes: vertical lines show input data 95% confidence interval; black line shows estimate; shaded area shows 95% uncertainty interval of estimate

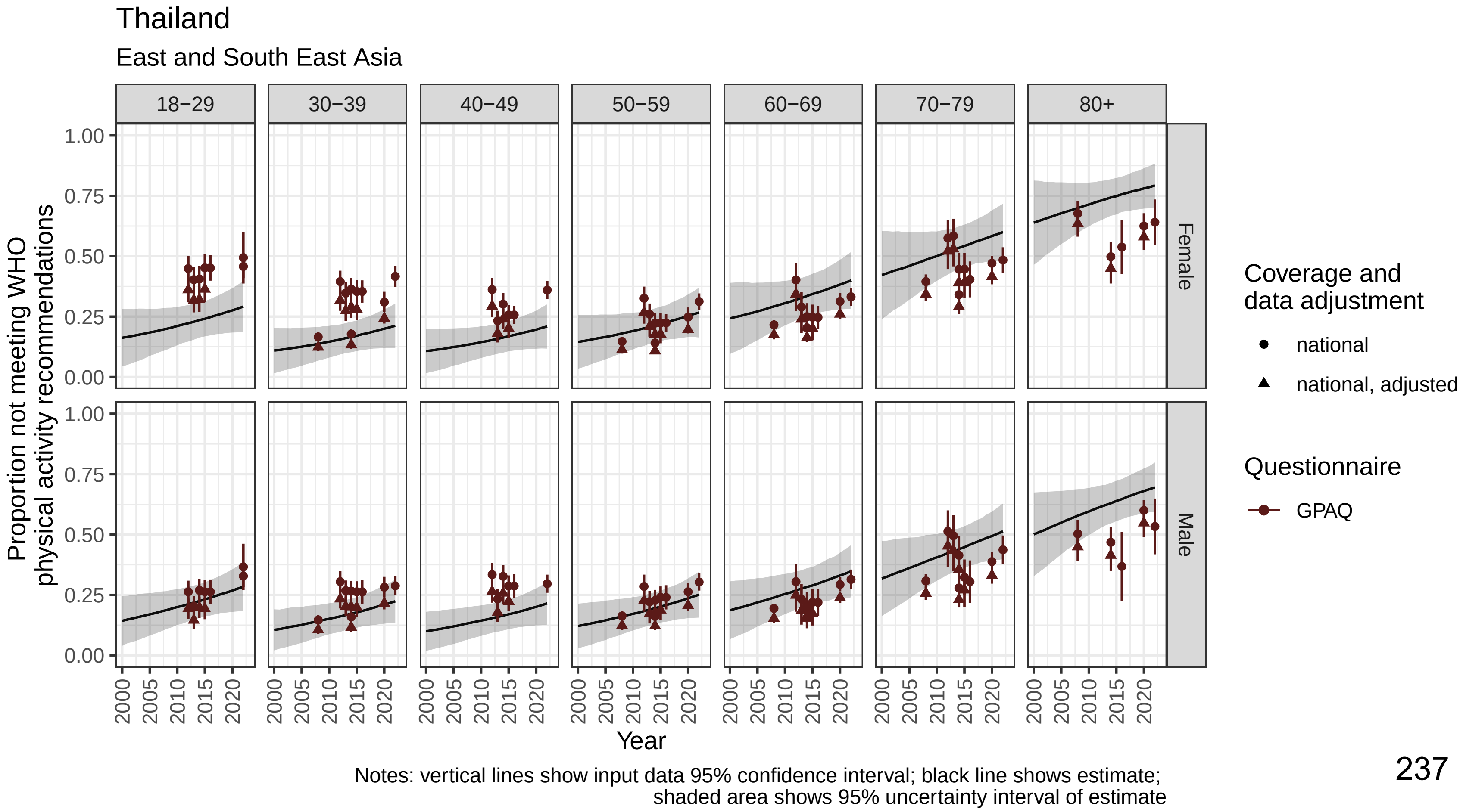
Thailand
East and South East Asia
18−29
30−39
40−49
50−59
60−69
70−79
80+
Female
Male
Proportion not meeting WHO physical activity recommendations
Year
Coverage and data adjustment
national
national, adjusted
Questionnaire
GPAQ
Notes: vertical lines show input data 95% confidence interval; black line shows estimate; shaded area shows 95% uncertainty interval of estimate

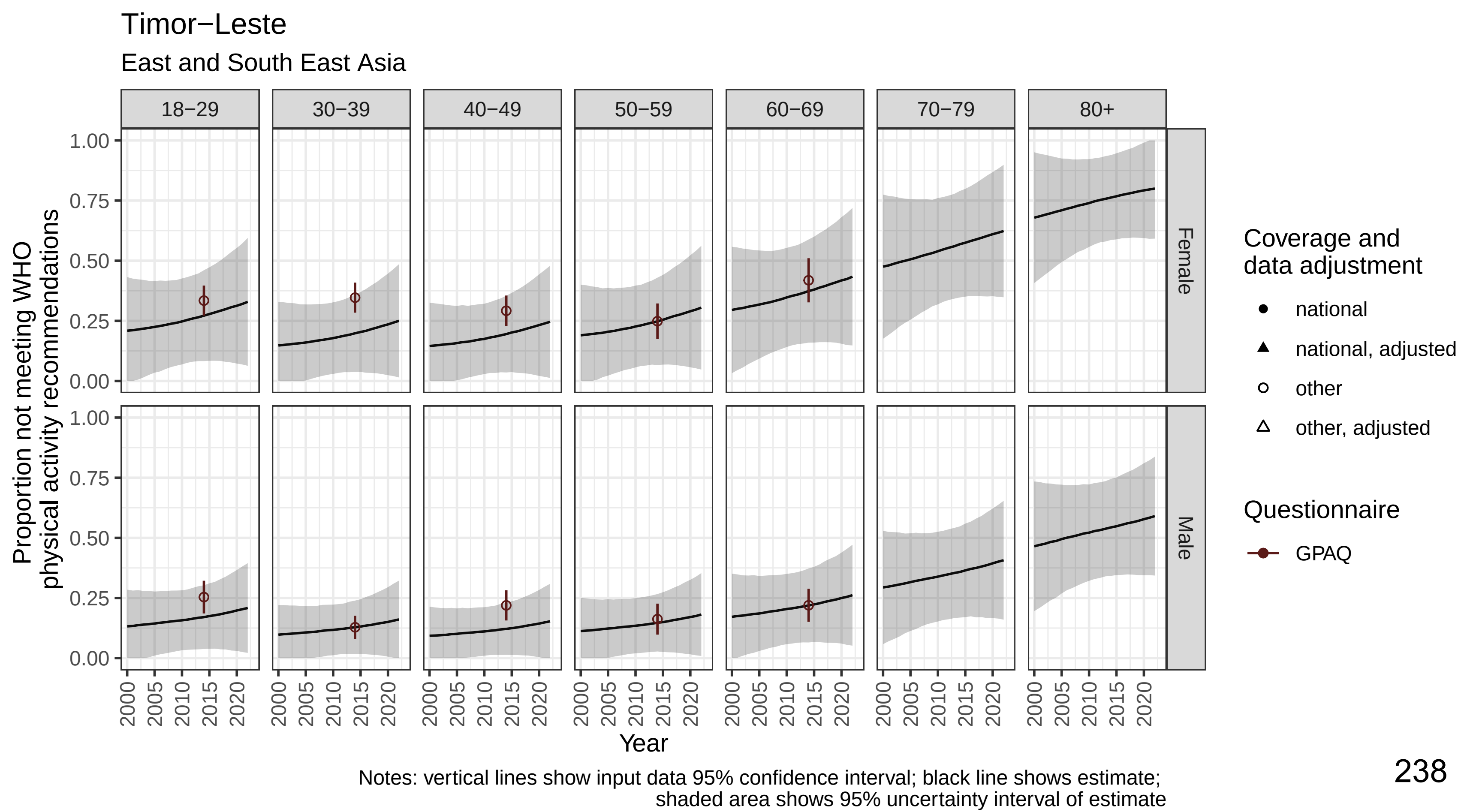
Timor−Leste
East and South East Asia
18−29
30−39
40−49
50−59
60−69
70−79
80+
Female
Male
Proportion not meeting WHO physical activity recommendations
1.00
0.75
0.50
0.25
0.00
2000
2005
2010
2015
2020
Year
Coverage and data adjustment
national
national, adjusted
other
other, adjusted
Questionnaire
GPAQ
Notes: vertical lines show input data 95% confidence interval; black line shows estimate; shaded area shows 95% uncertainty interval of estimate

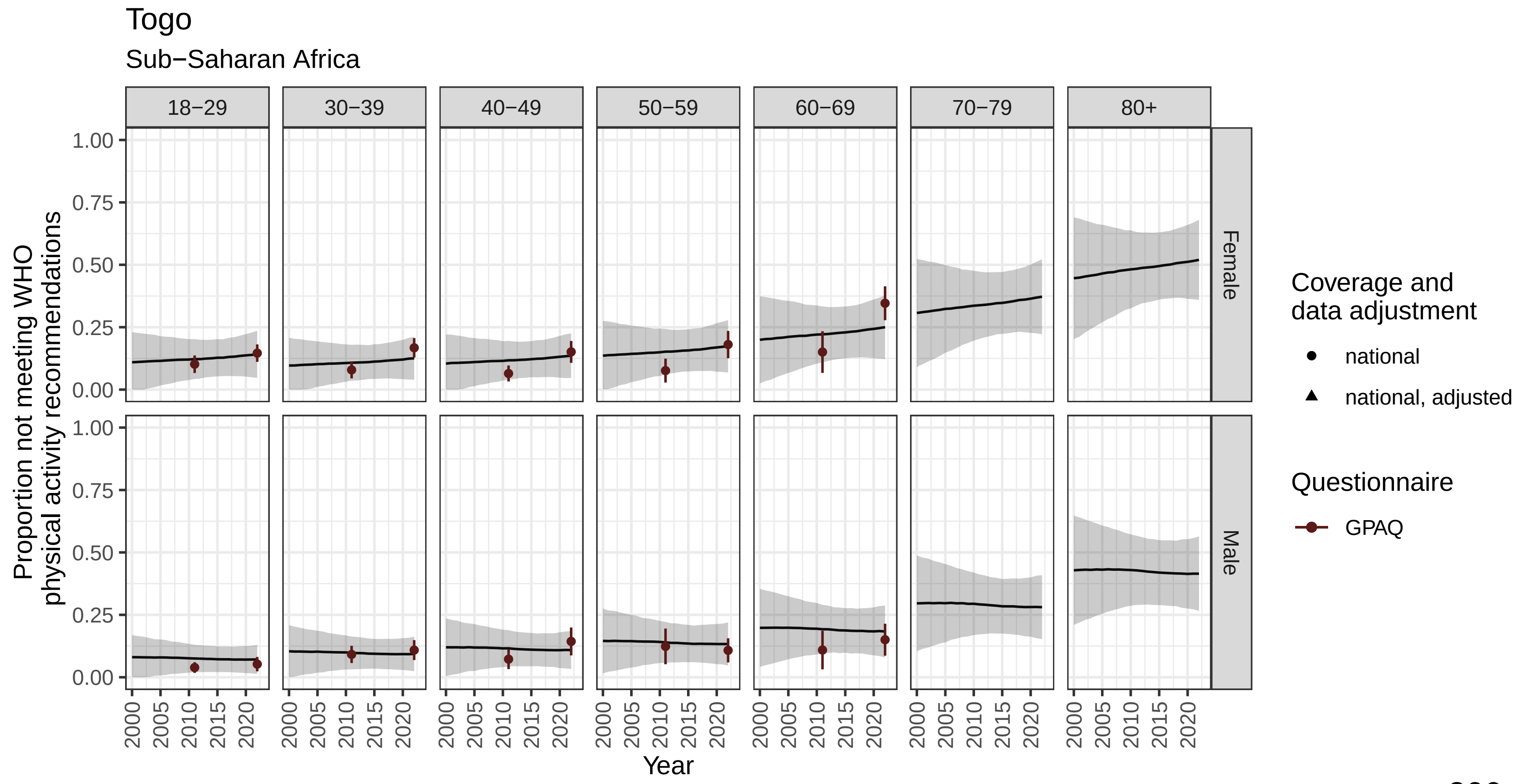

Notes: vertical lines show input data 95% confidence interval; black line shows estimate; shaded area shows 95% uncertainty interval of estimate

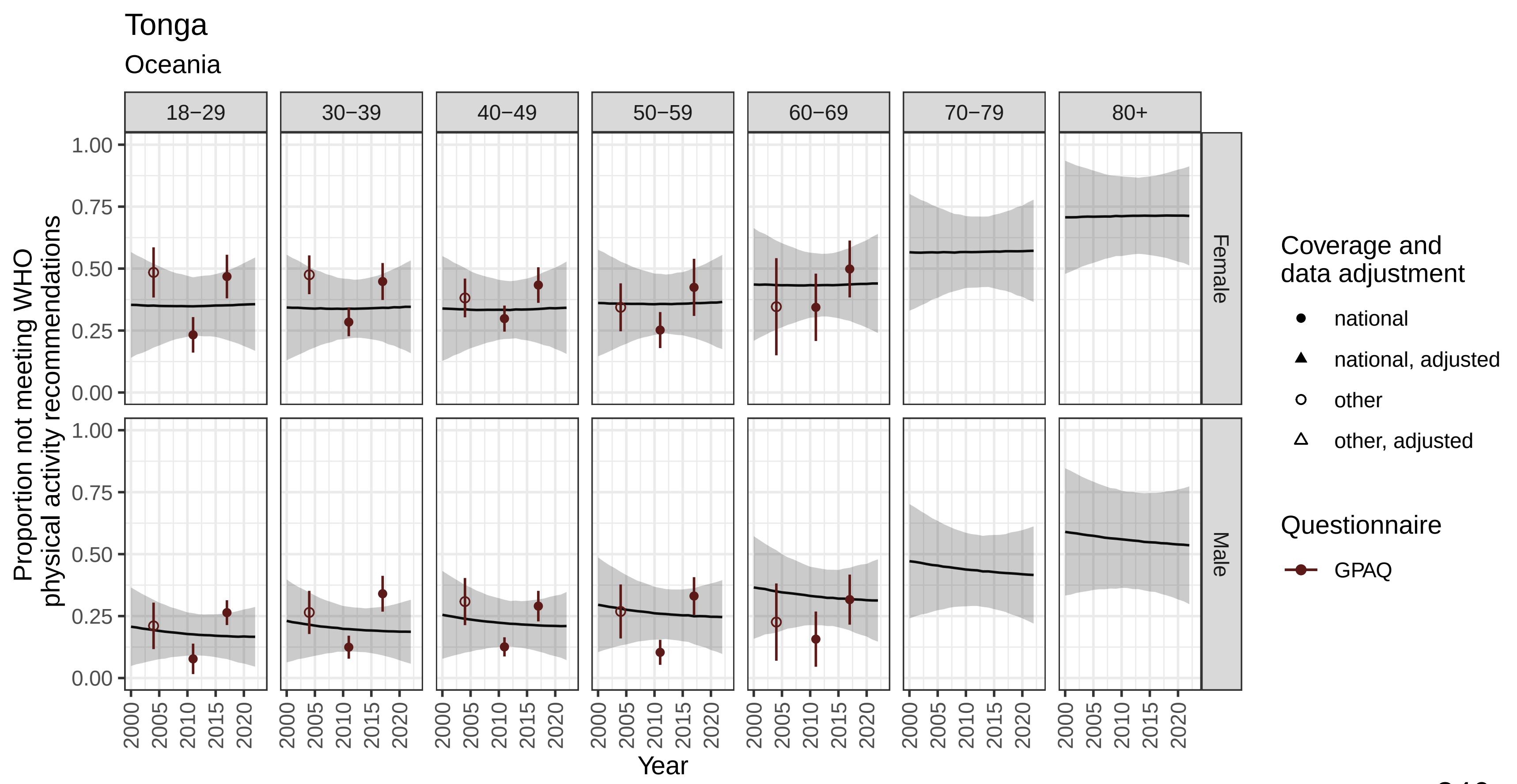

Notes: vertical lines show input data 95% confidence interval; black line shows estimate; shaded area shows 95% uncertainty interval of estimate

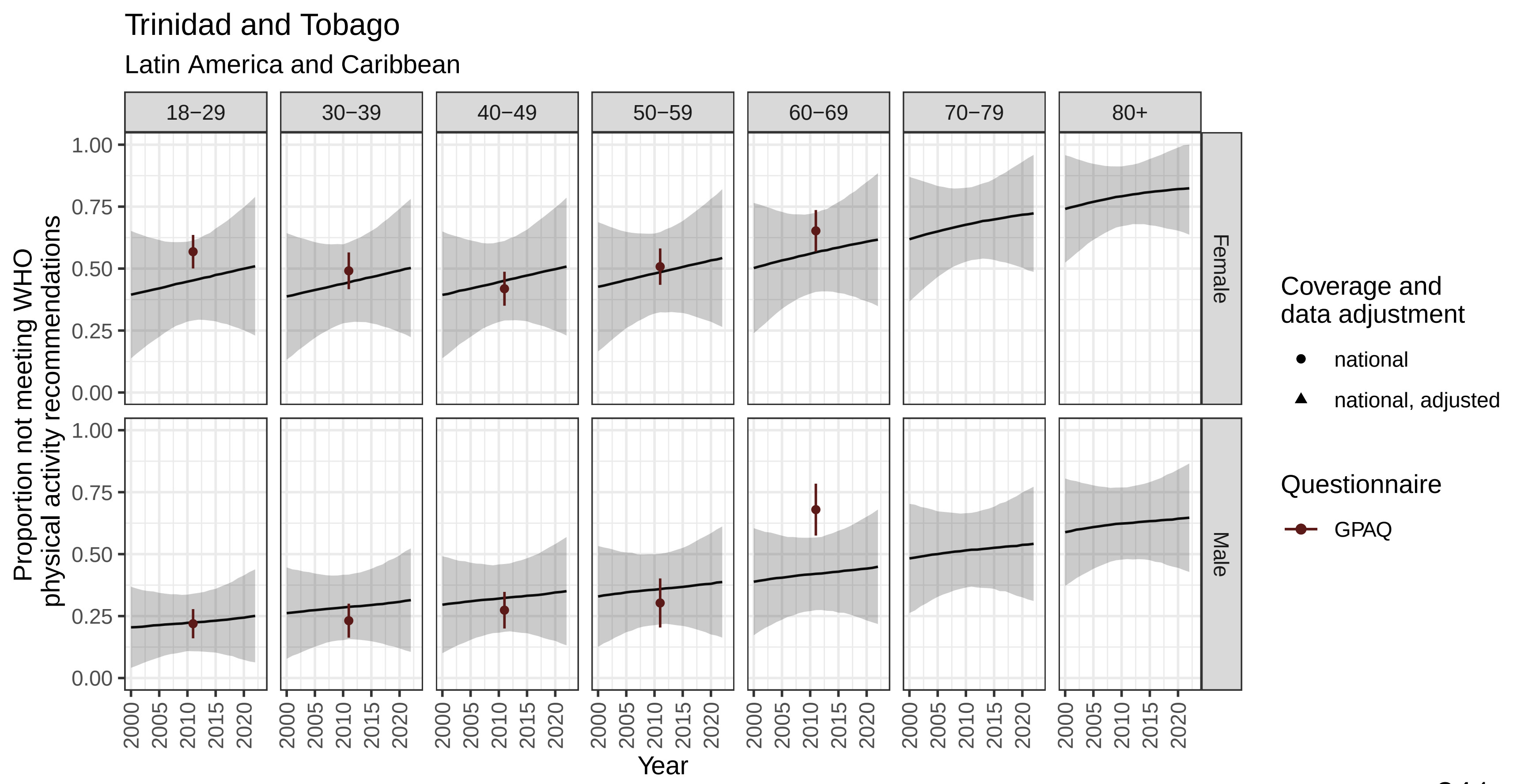

Notes: vertical lines show input data 95% confidence interval; black line shows estimate; shaded area shows 95% uncertainty interval of estimate

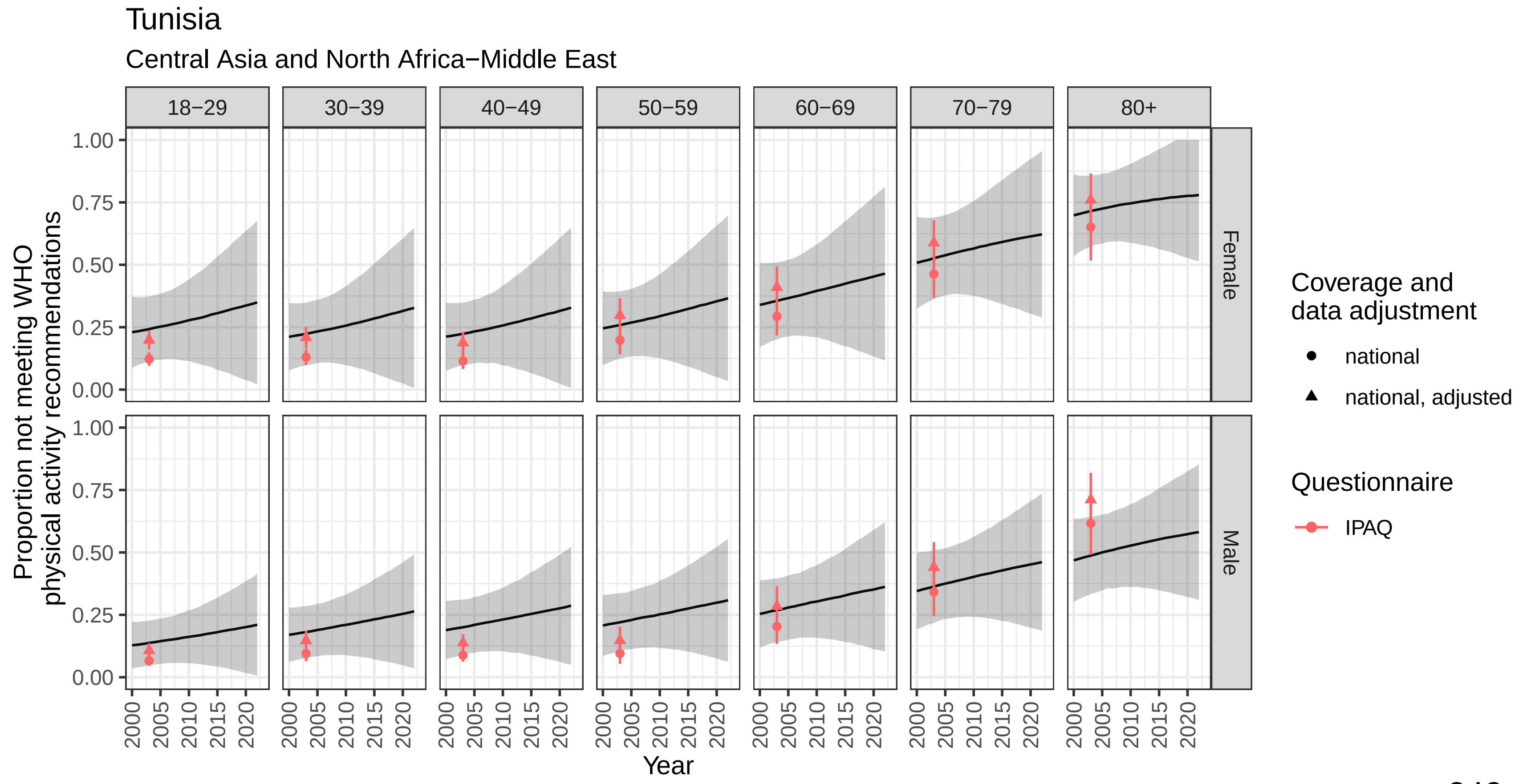

Notes: vertical lines show input data 95% confidence interval; black line shows estimate; shaded area shows 95% uncertainty interval of estimate

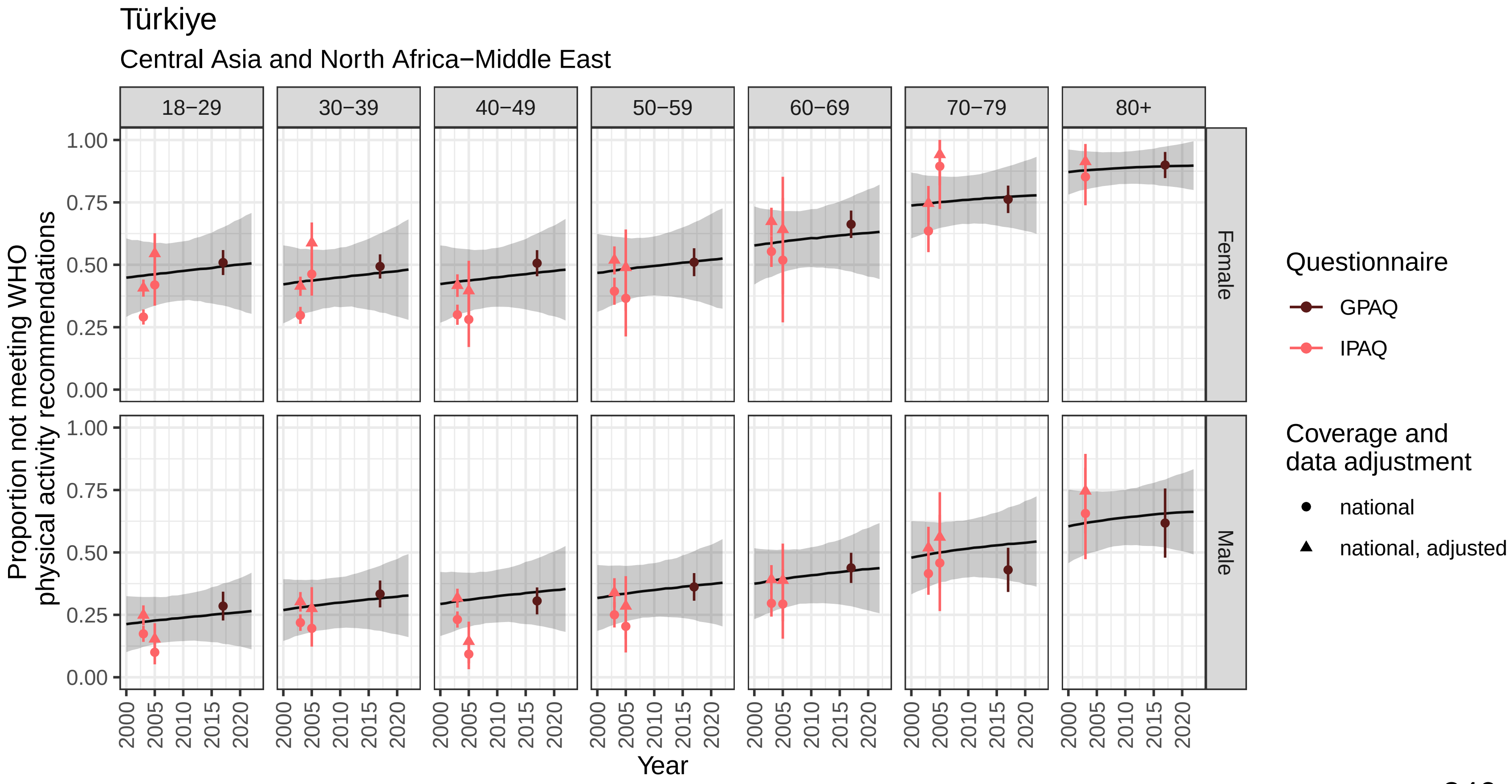

Notes: vertical lines show input data 95% confidence interval; black line shows estimate; shaded area shows 95% uncertainty interval of estimate

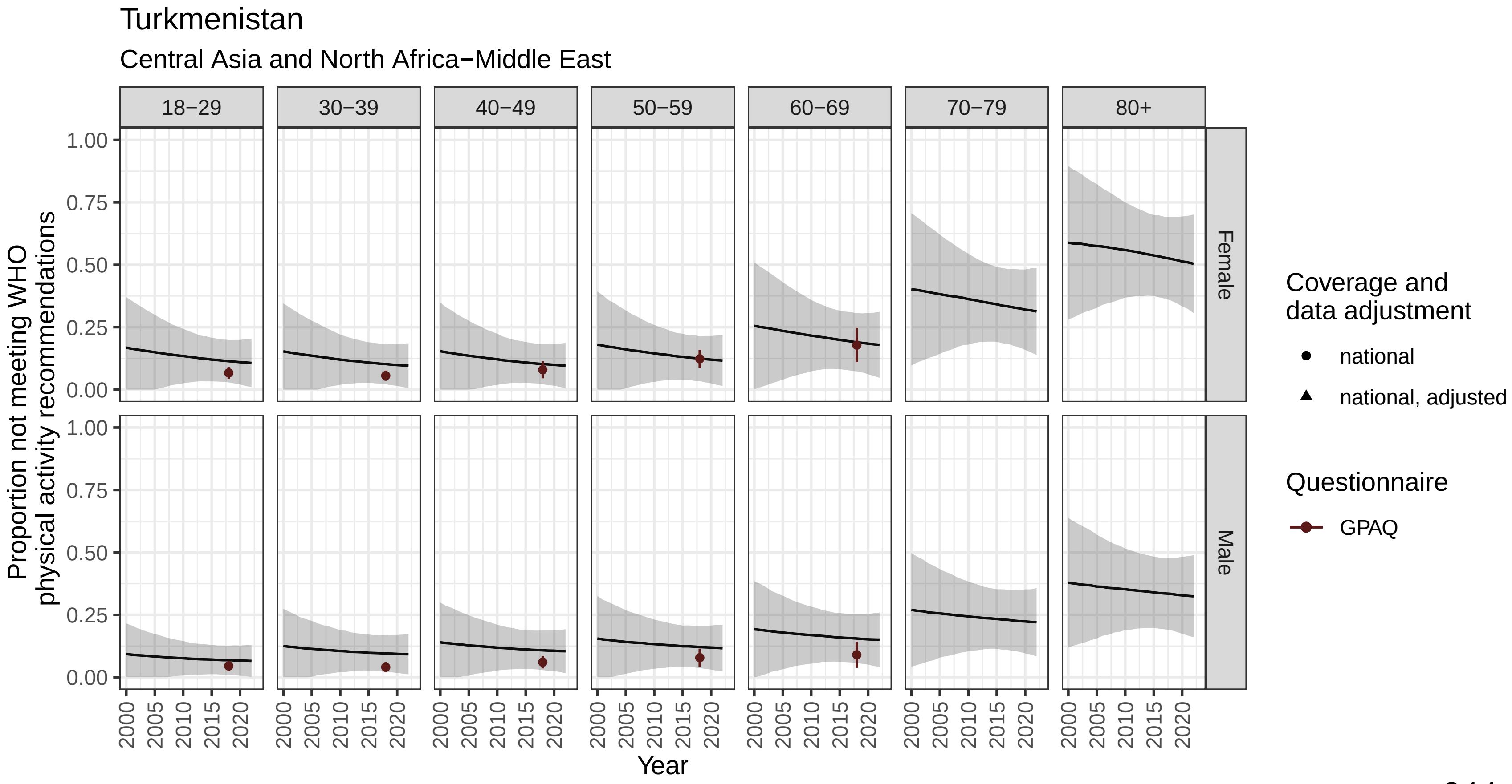

Notes: vertical lines show input data 95% confidence interval; black line shows estimate; shaded area shows 95% uncertainty interval of estimate

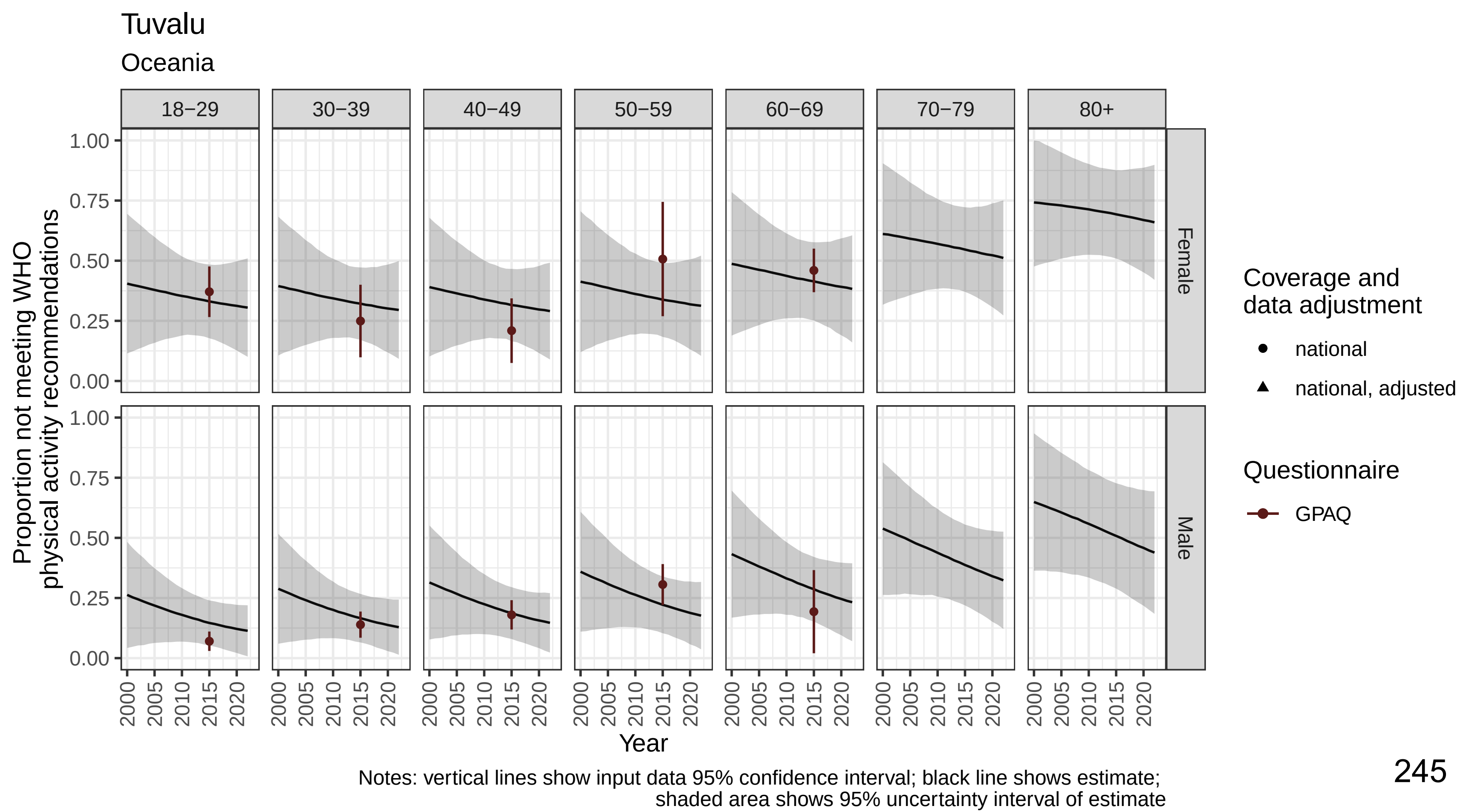

Tuvalu
Oceania
18−29
30−39
40−49
50−59
60−69
70−79
80+
Female
Male
Proportion not meeting WHO physical activity recommendations
1.00
0.75
0.50
0.25
0.00
2000
2005
2010
2015
2020
Year
Coverage and data adjustment
national
national, adjusted
Questionnaire
GPAQ
Notes: vertical lines show input data 95% confidence interval; black line shows estimate; shaded area shows 95% uncertainty interval of estimate

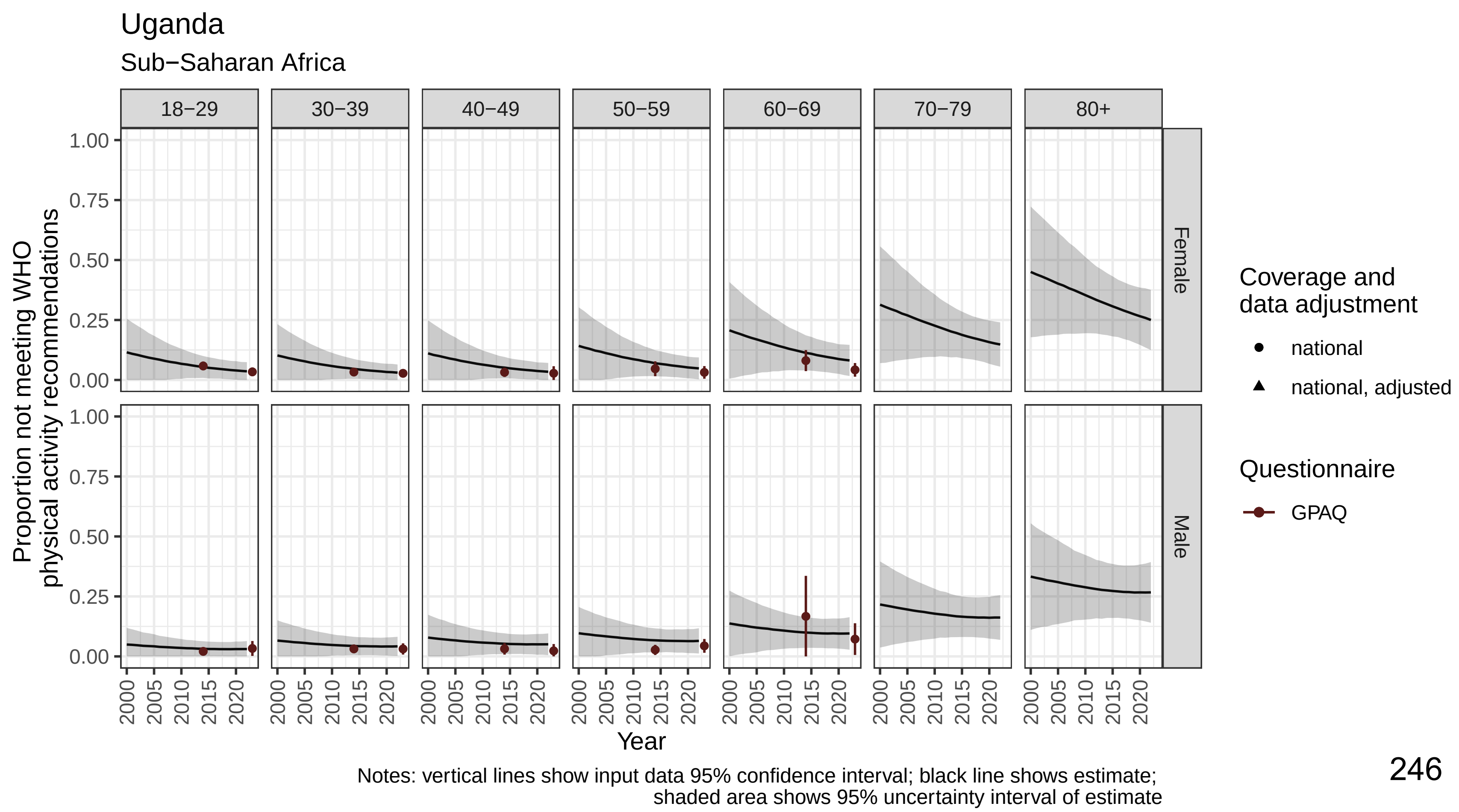

Uganda
Sub−Saharan Africa
18−29
30−39
40−49
50−59
60−69
70−79
80+
Female
Male
Proportion not meeting WHO physical activity recommendations
1.00
0.75
0.50
0.25
0.00
2000
2005
2010
2015
2020
Year
Coverage and data adjustment
national
national, adjusted
Questionnaire
GPAQ
Notes: vertical lines show input data 95% confidence interval; black line shows estimate;
shaded area shows 95% uncertainty interval of estimate

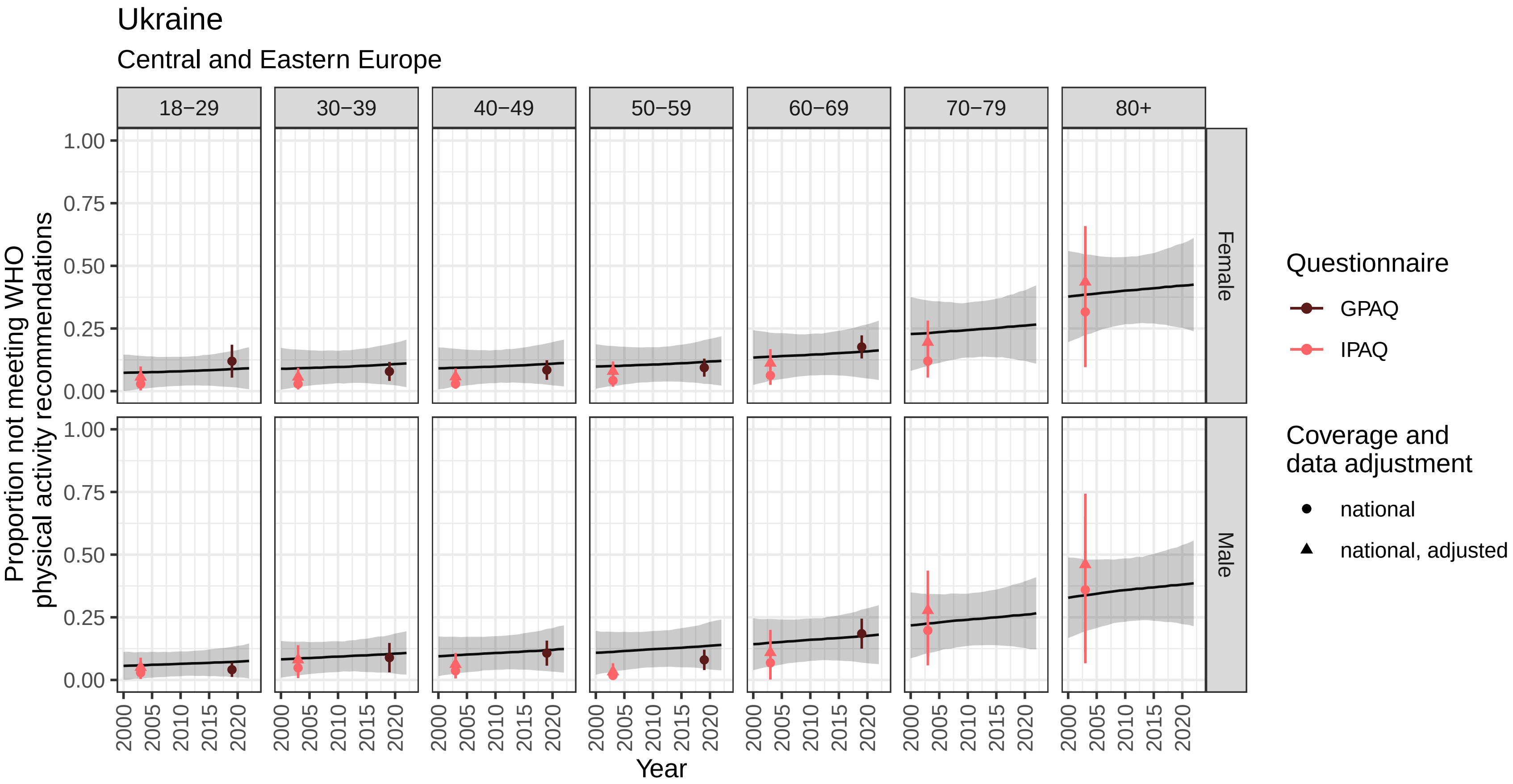

Notes: vertical lines show input data 95% confidence interval; black line shows estimate; shaded area shows 95% uncertainty interval of estimate

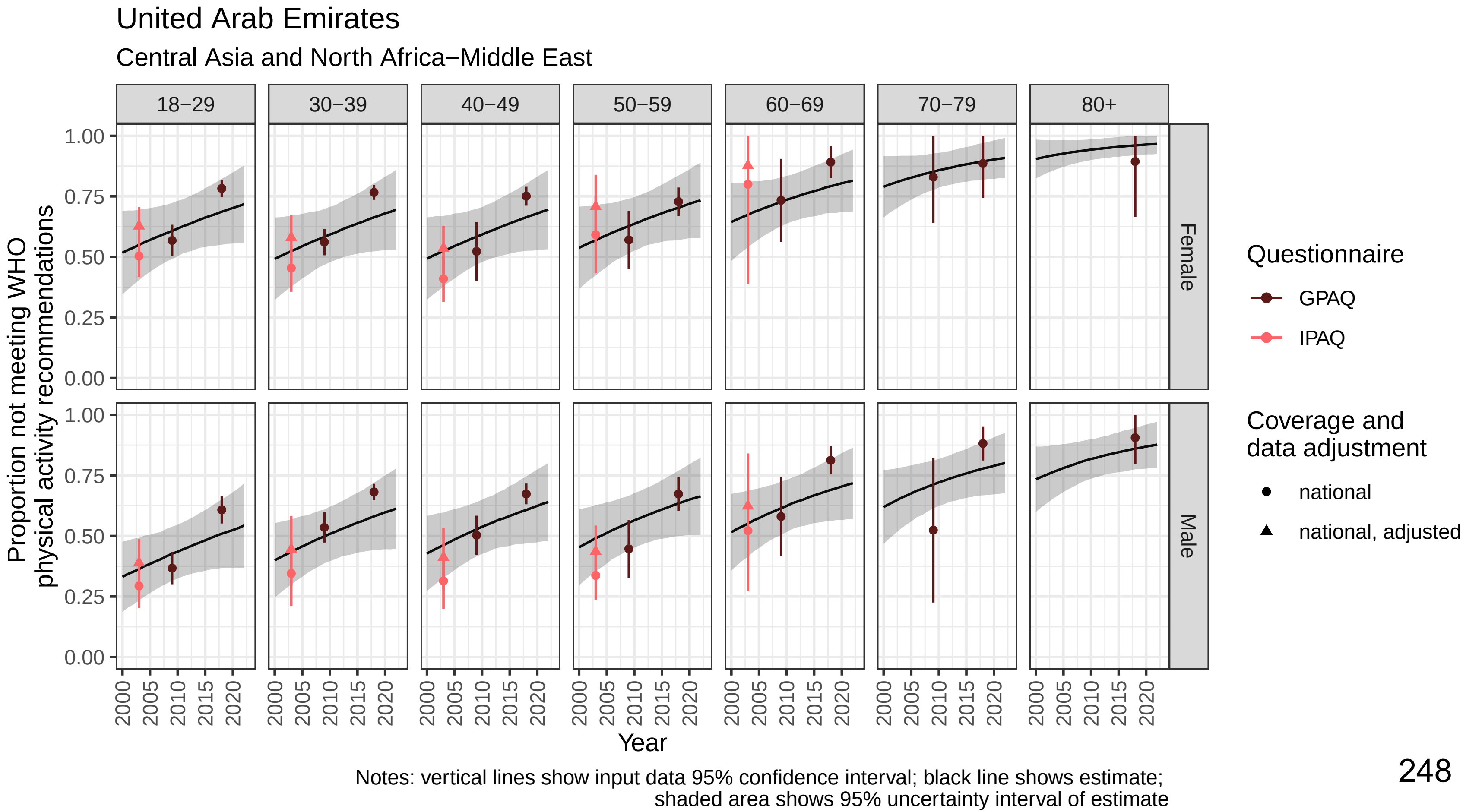

United Arab Emirates
Central Asia and North Africa−Middle East
18−29
30−39
40−49
50−59
60−69
70−79
80+
Female
Male
Proportion not meeting WHO physical activity recommendations
1.00
0.75
0.50
0.25
0.00
2000
2005
2010
2015
2020
Year
Questionnaire
GPAQ
IPAQ
Coverage and data adjustment
national
national, adjusted
Notes: vertical lines show input data 95% confidence interval; black line shows estimate;
shaded area shows 95% uncertainty interval of estimate

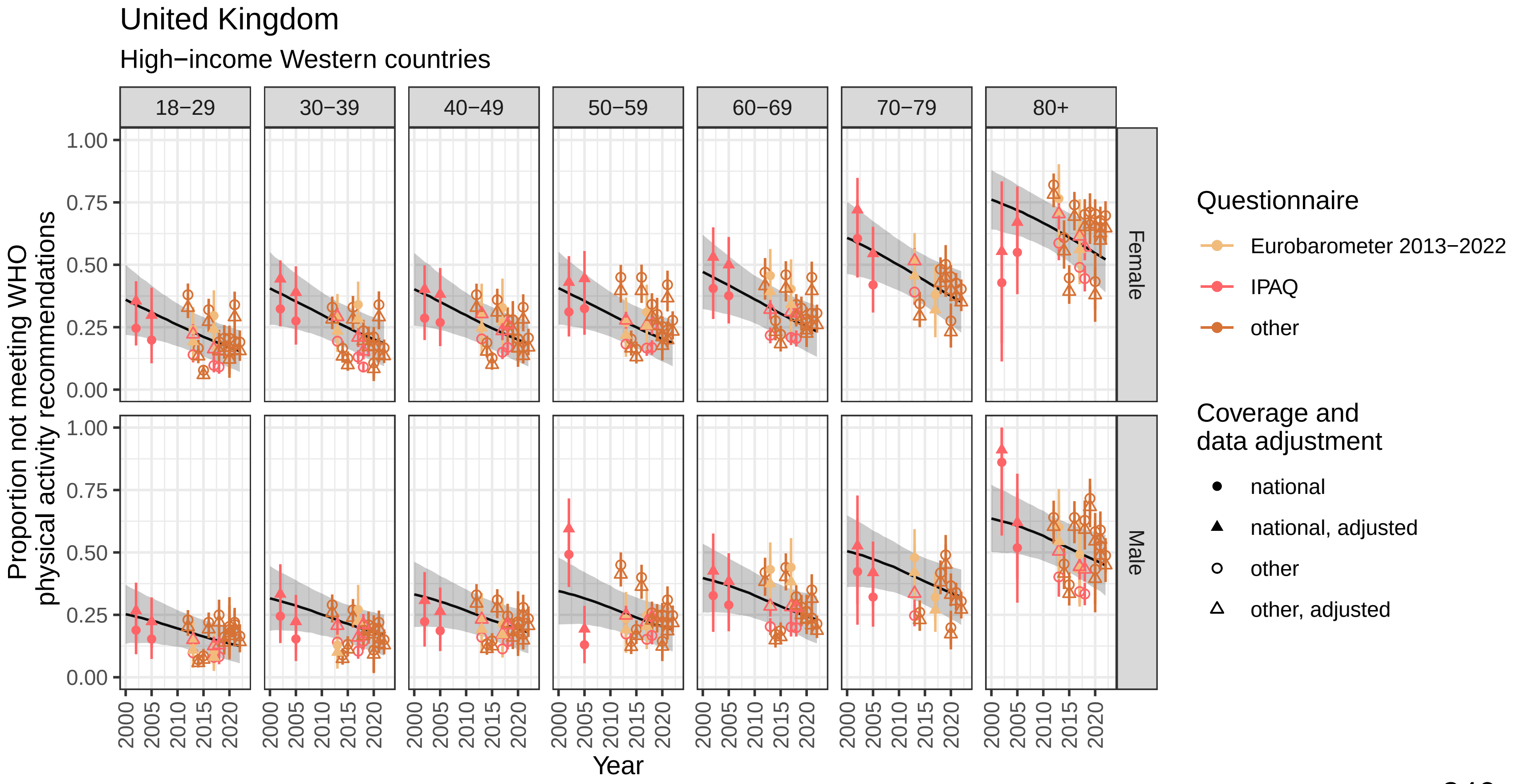

Notes: vertical lines show input data 95% confidence interval; black line shows estimate; shaded area shows 95% uncertainty interval of estimate

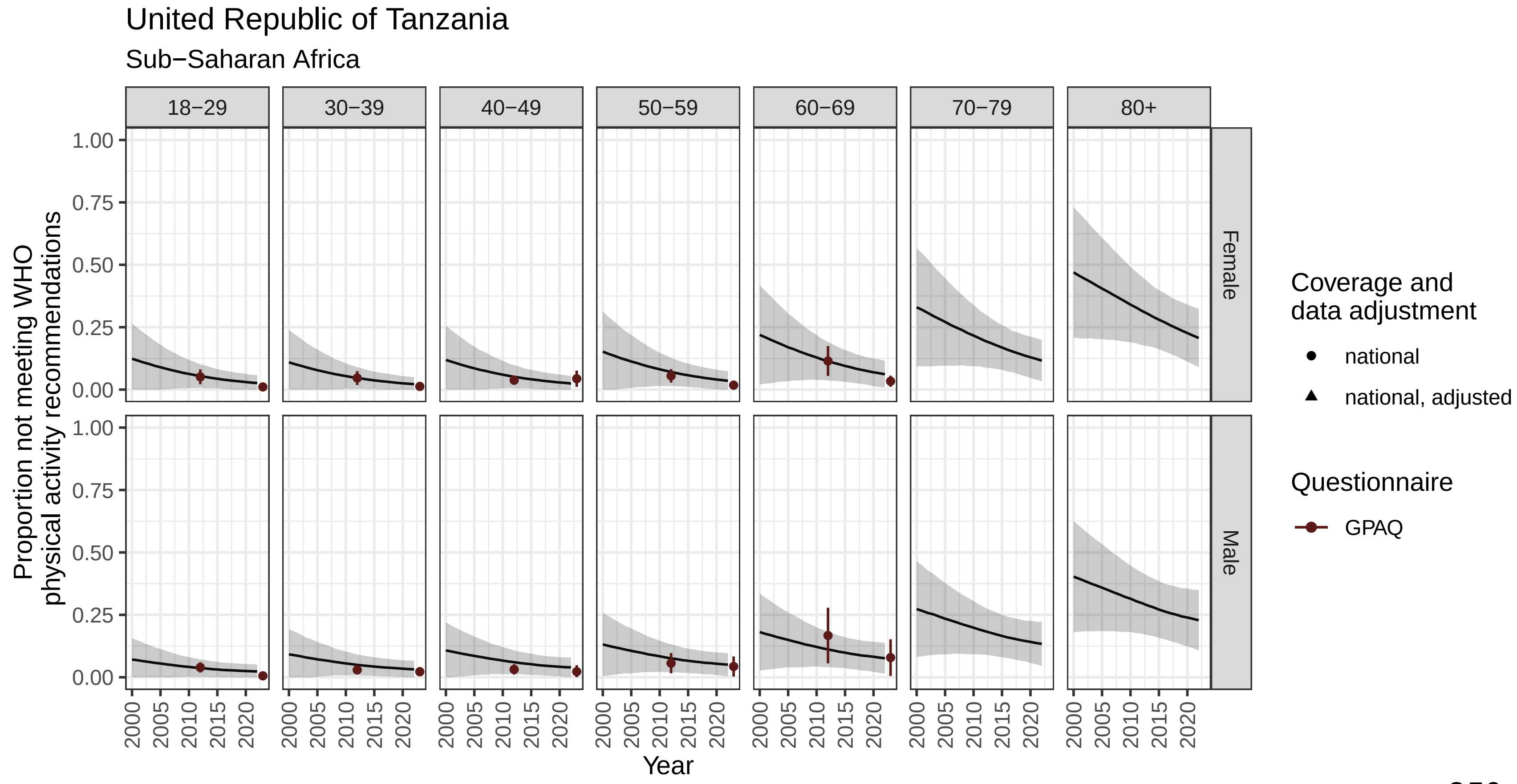

Notes: vertical lines show input data 95% confidence interval; black line shows estimate; shaded area shows 95% uncertainty interval of estimate

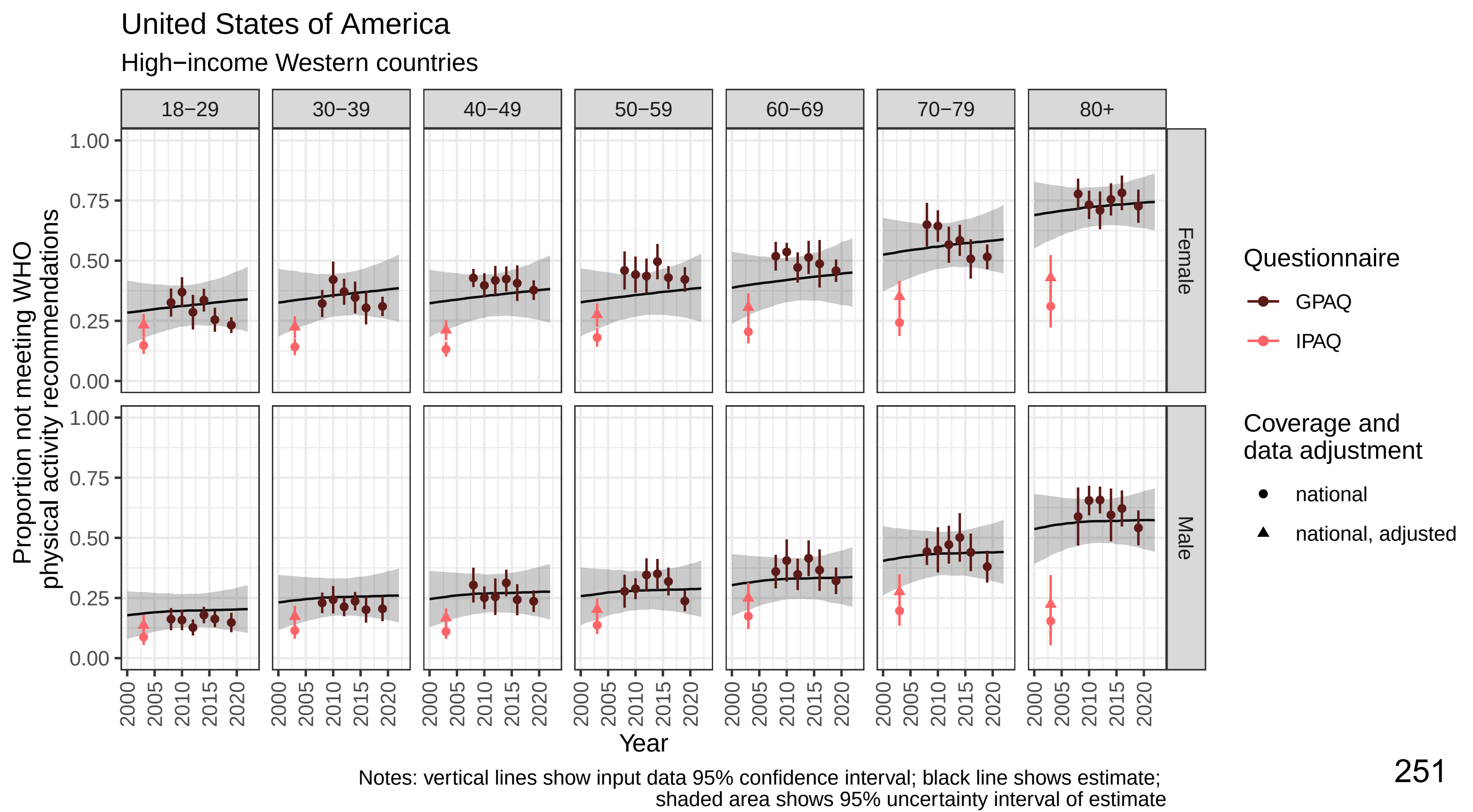

United States of America
High−income Western countries
18−29
30−39
40−49
50−59
60−69
70−79
80+
Female
Male
Proportion not meeting WHO physical activity recommendations
1.00
0.75
0.50
0.25
0.00
2000
2005
2010
2015
2020
Year
Questionnaire
GPAQ
IPAQ
Coverage and data adjustment
national
national, adjusted
Notes: vertical lines show input data 95% confidence interval; black line shows estimate; shaded area shows 95% uncertainty interval of estimate

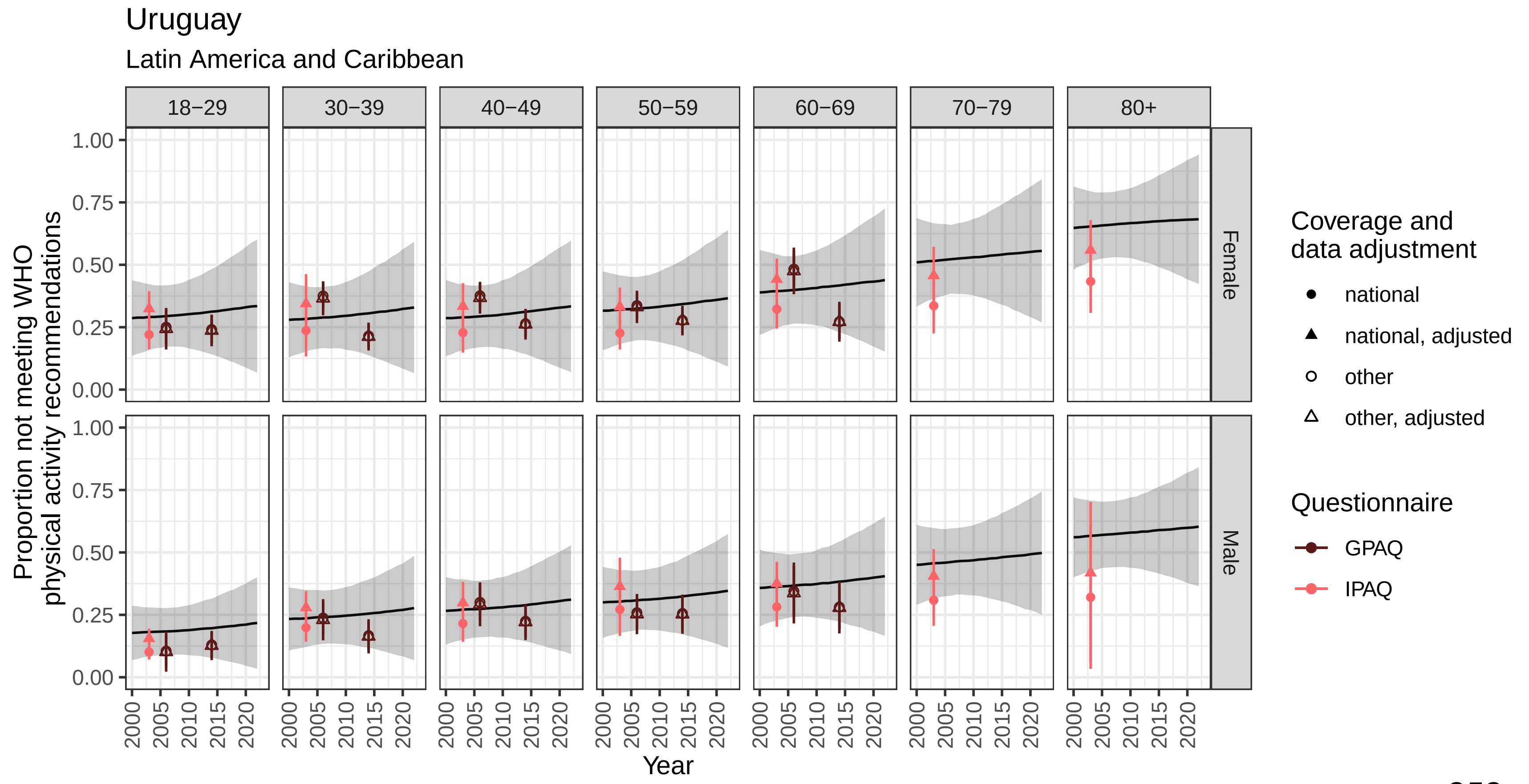

Notes: vertical lines show input data 95% confidence interval; black line shows estimate; shaded area shows 95% uncertainty interval of estimate

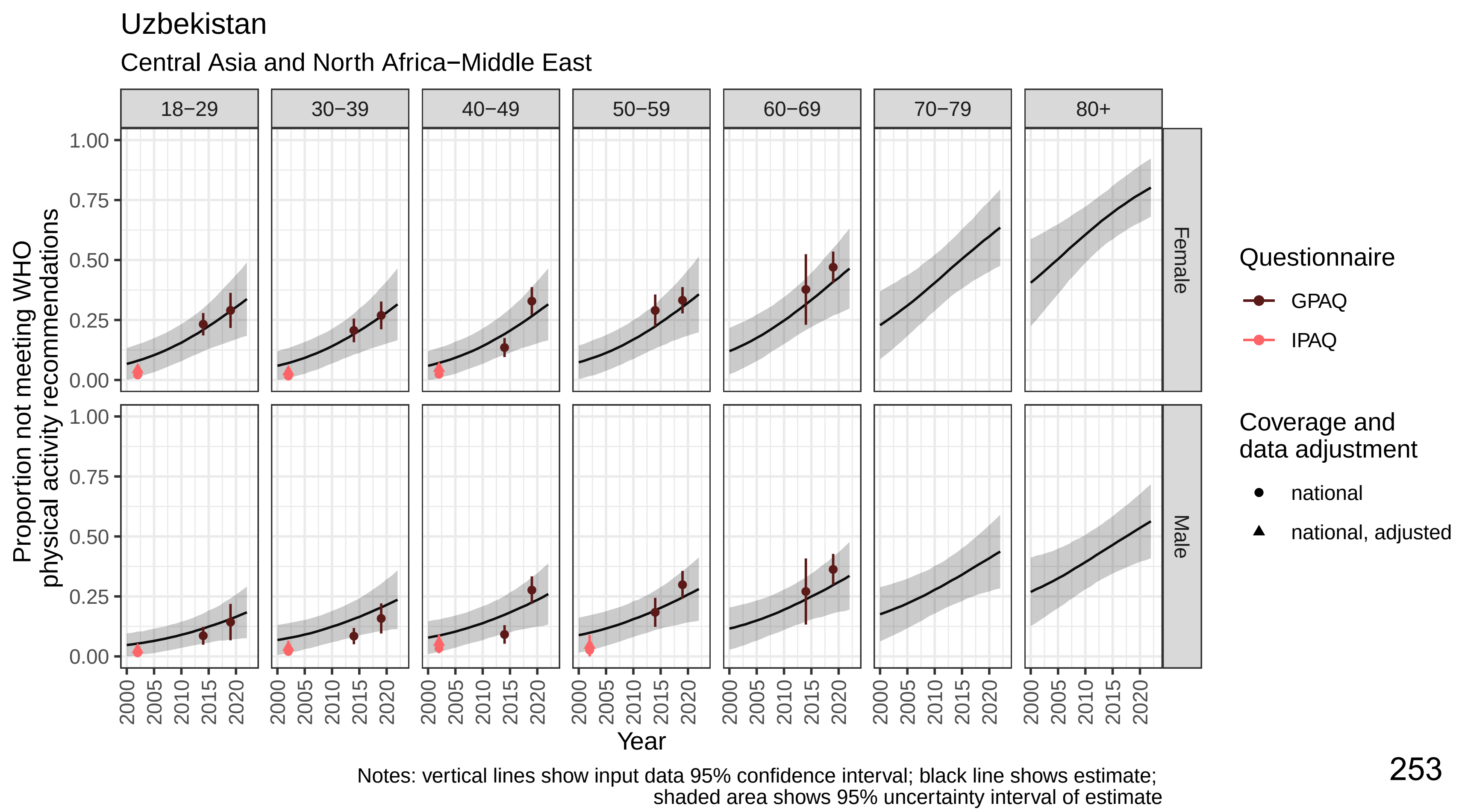
Uzbekistan
Central Asia and North Africa−Middle East
18−29
30−39
40−49
50−59
60−69
70−79
80+
Female
Male
Proportion not meeting WHO physical activity recommendations
Year
Questionnaire
GPAQ
IPAQ
Coverage and data adjustment
national
national, adjusted
Notes: vertical lines show input data 95% confidence interval; black line shows estimate; shaded area shows 95% uncertainty interval of estimate

Vanuatu

Oceania

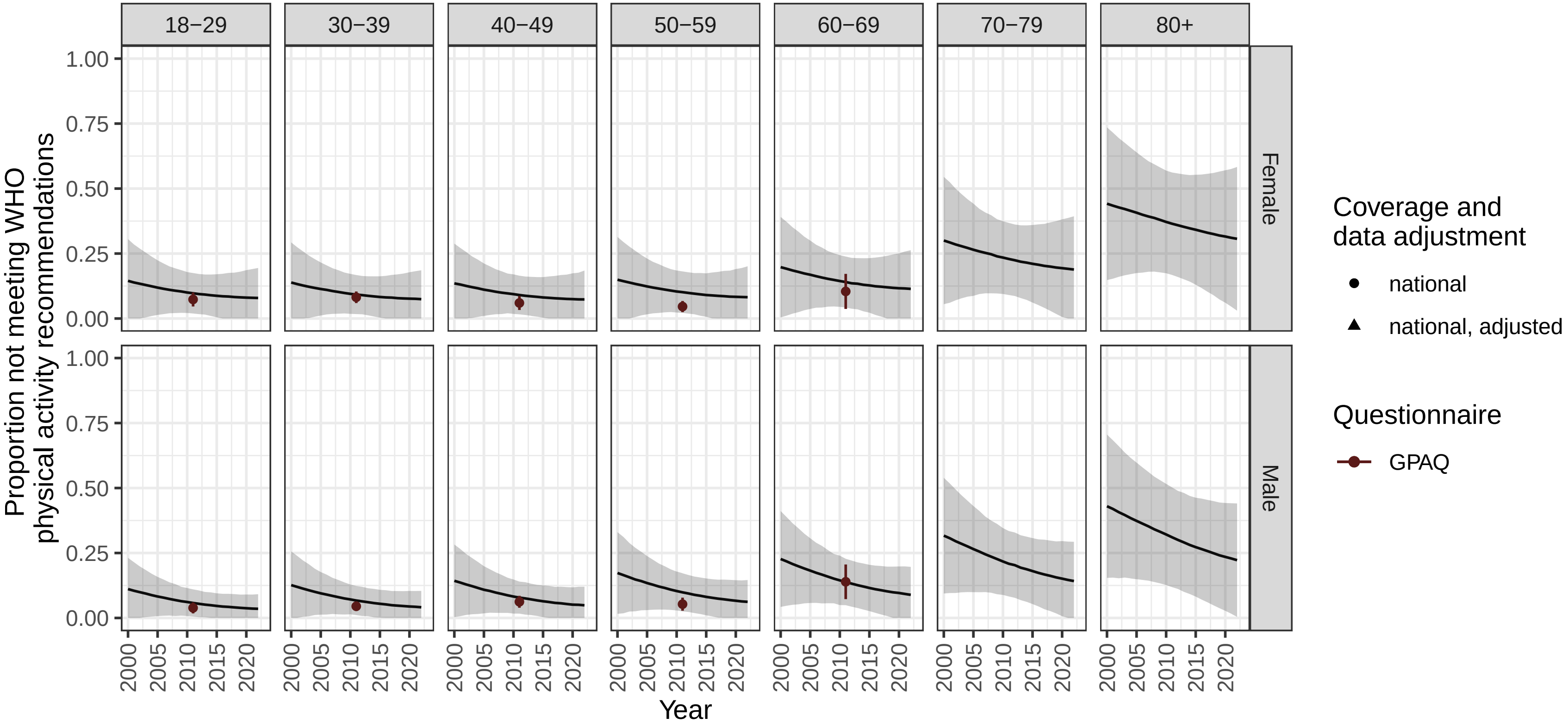

Notes: vertical lines show input data 95% confidence interval; black line shows estimate; shaded area shows 95% uncertainty interval of estimate

Venezuela (Bolivarian Republic of)

Latin America and Caribbean

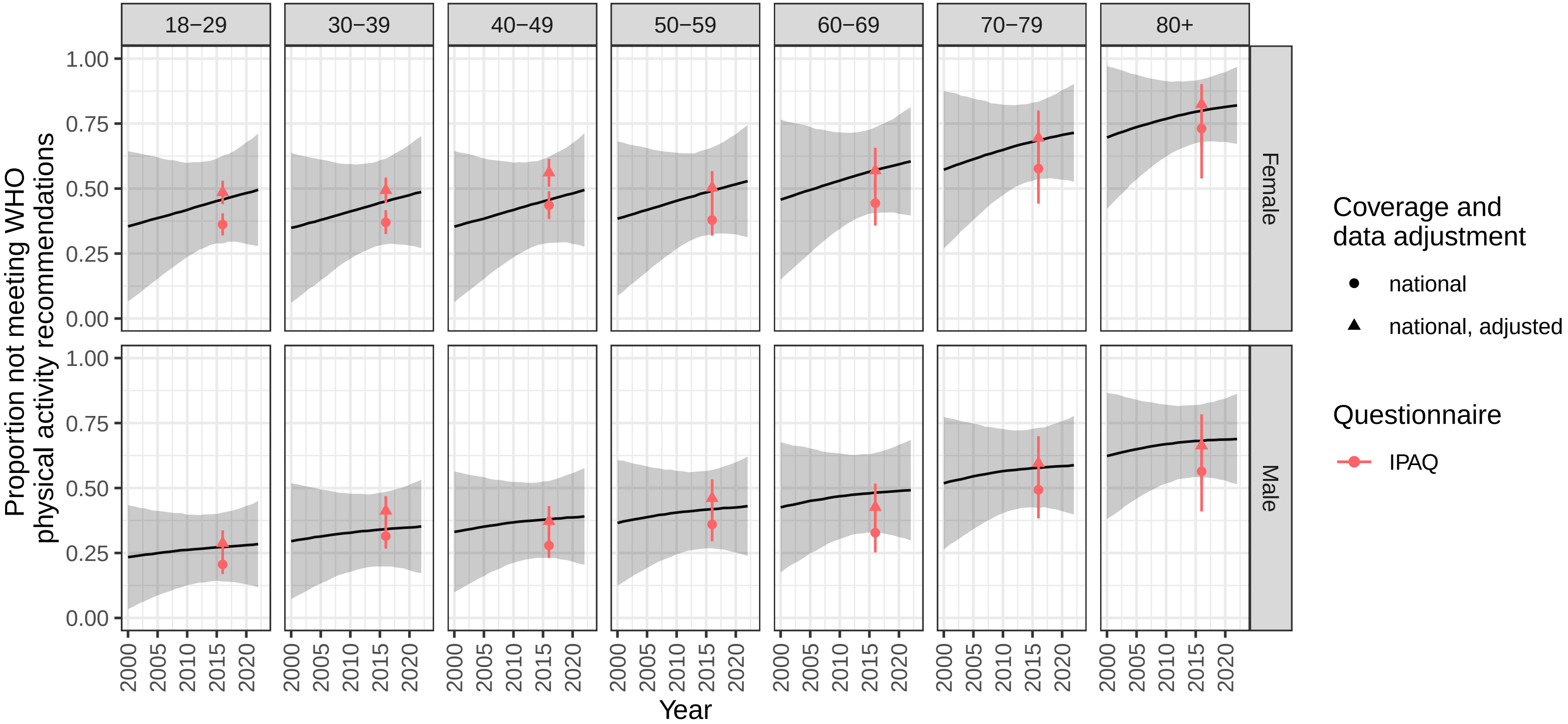

Notes: vertical lines show input data 95% confidence interval; black line shows estimate; shaded area shows 95% uncertainty interval of estimate

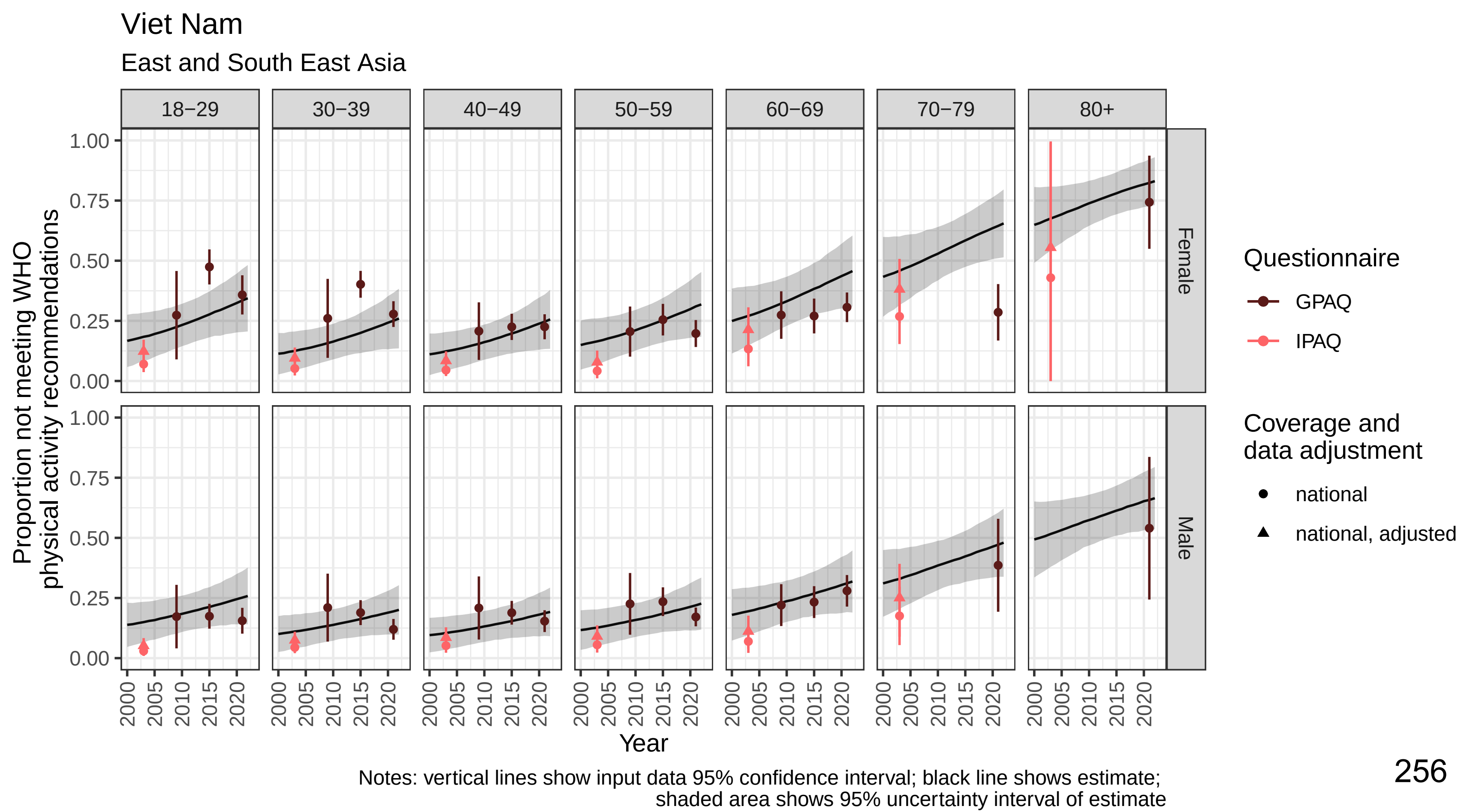

Viet Nam
East and South East Asia
18−29
30−39
40−49
50−59
60−69
70−79
80+
Female
Male
1.00
0.75
0.50
0.25
0.00
Proportion not meeting WHO physical activity recommendations
2000
2005
2010
2015
2020
Year
Questionnaire
GPAQ
IPAQ
Coverage and data adjustment
national
national, adjusted
Notes: vertical lines show input data 95% confidence interval; black line shows estimate;
shaded area shows 95% uncertainty interval of estimate

Yemen

Central Asia and North Africa−Middle East

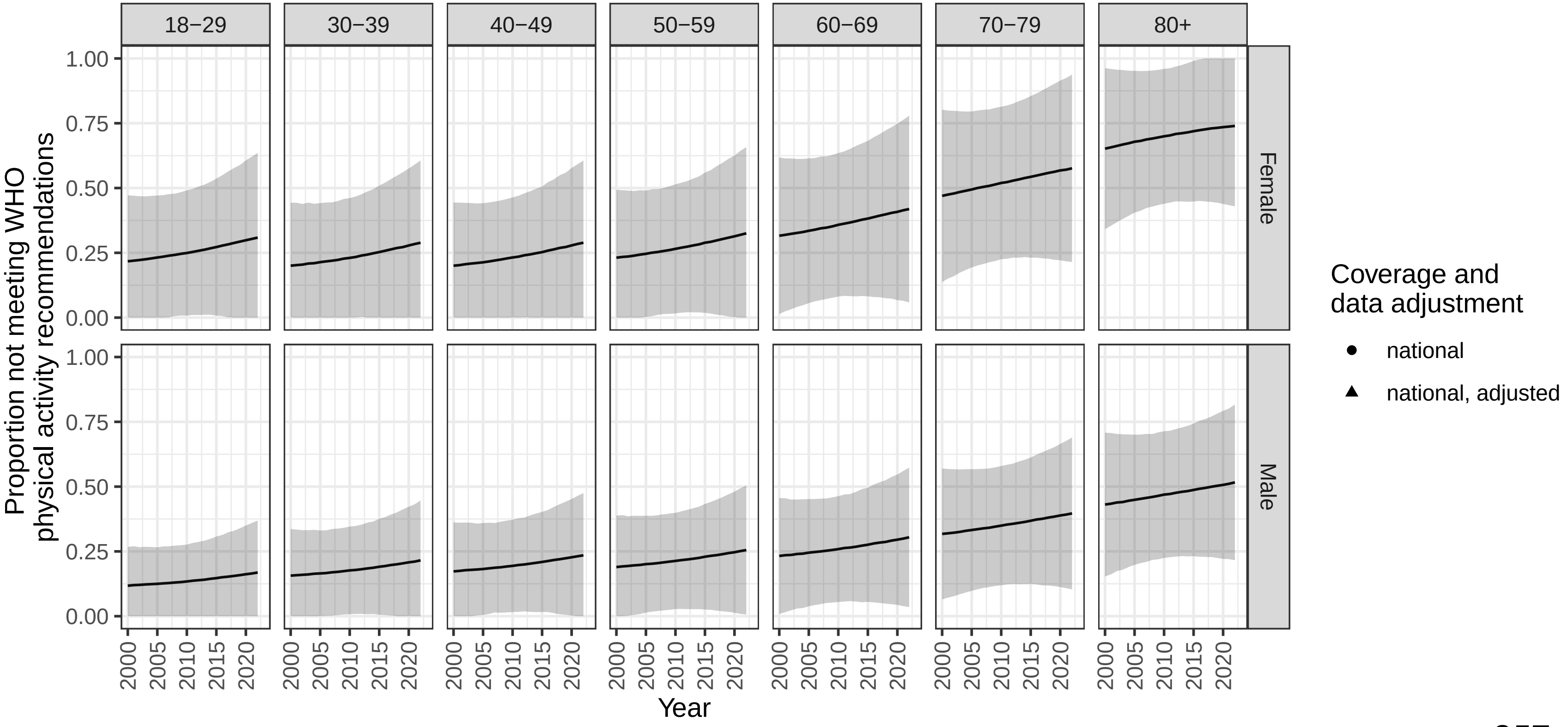

Notes: vertical lines show input data 95% confidence interval; black line shows estimate; shaded area shows 95% uncertainty interval of estimate

Zambia

Sub−Saharan Africa

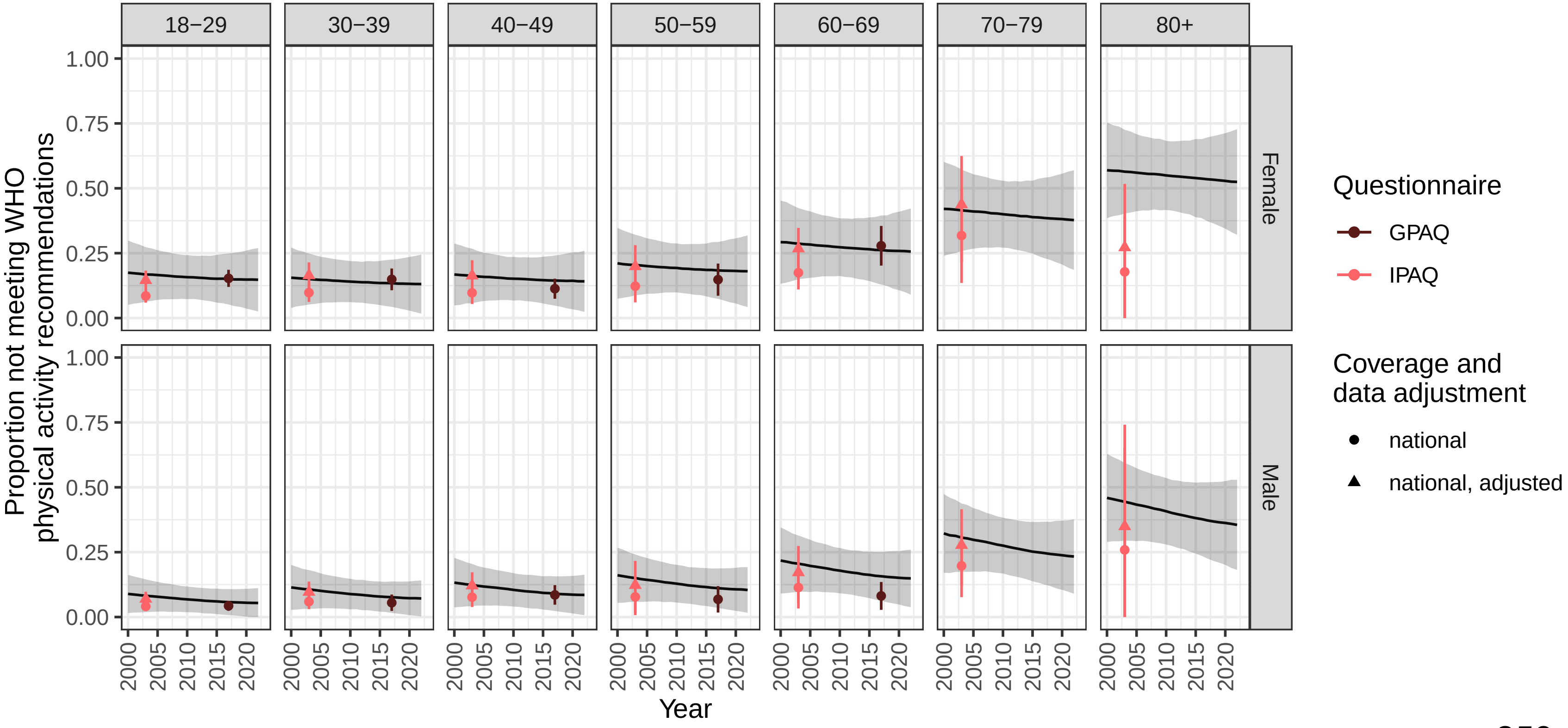

Notes: vertical lines show input data 95% confidence interval; black line shows estimate; shaded area shows 95% uncertainty interval of estimate

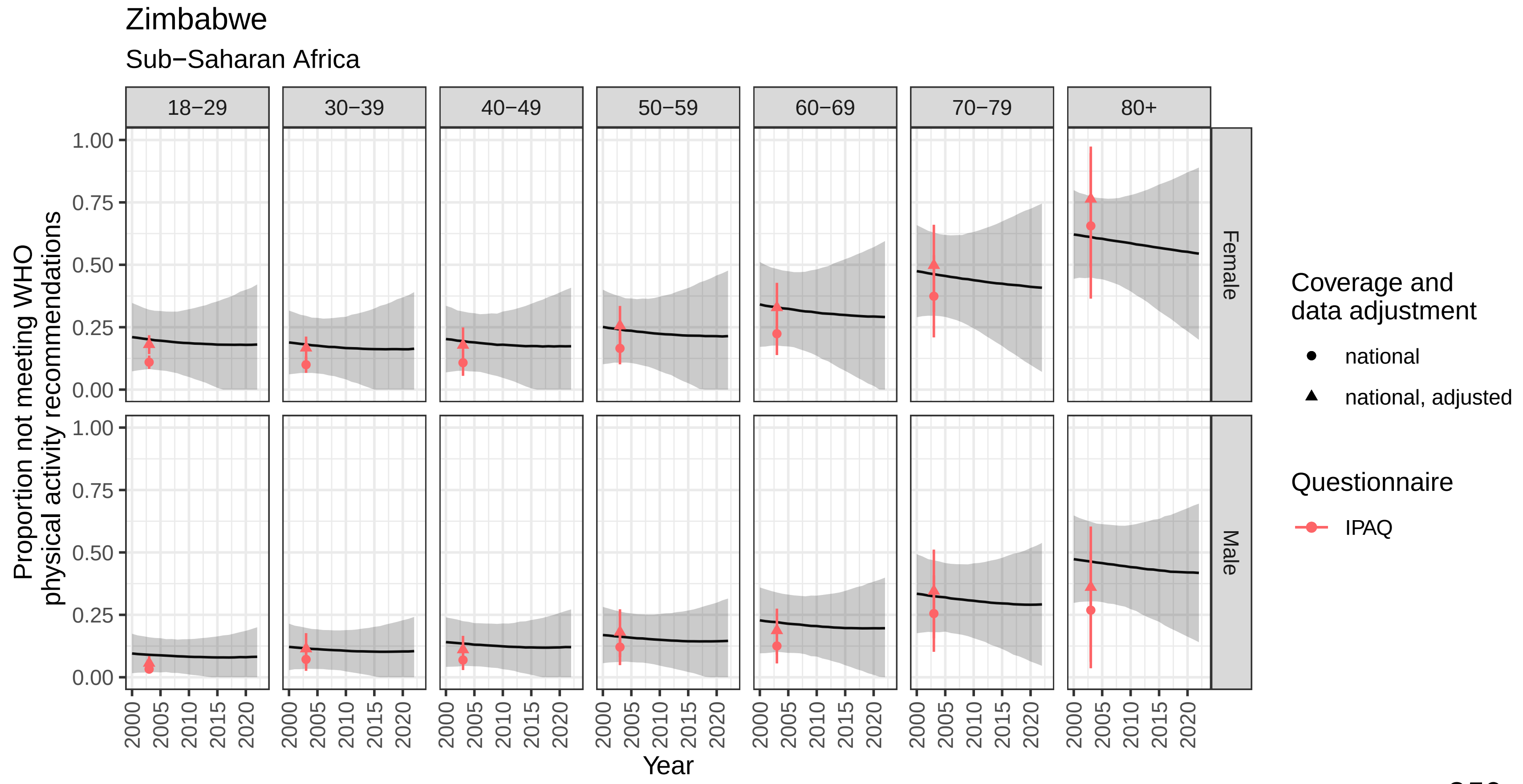

Notes: vertical lines show input data 95% confidence interval; black line shows estimate; shaded area shows 95% uncertainty interval of estimate

References

1. Guthold R, Stevens GA, Riley LM, Bull FC. Worldwide trends in insufficient physical activity from 2001 to 2016: a pooled analysis of 358 population-based surveys with 1·9 million participants. Lancet Glob Health. 2018 Oct;6(10):e1077–86.

2. World Health Organization. STEPwise approach to NCD risk factor surveillance (STEPS) [Internet]. Available from: https://www.who.int/teams/noncommunicable-diseases/surveillance/systems-tools/steps

3. World Health Organization. Assessing national capacity for the prevention and control of noncommunicable diseases: report of the 2021 global survey [Internet]. Geneva, Switzerland: World Health Organization; 2023. Available from: https://apps.who.int/iris/rest/bitstreams/1515822/retrieve

4. United Nations Department of Economic and Social Affairs. World Population Prospects 2022. 2022; Available from: https://population.un.org/wpp/

5. Surveillance and Population-Based Prevention, World Health Organization. Global Physical Activity Questionnaire (GPAQ) Analysis Guide [Internet]. Geneva, Switzerland: World Health Organization; Available from: https://cdn.who.int/media/docs/default-source/ncds/ncd-surveillance/gpaq-analysis-guide.pdf

6. Craig CL, Marshall AL, Sjöström M, Bauman AE, Booth ML, Ainsworth BE, et al. International Physical Activity Questionnaire: 12-Country Reliability and Validity: Med Sci Sports Exerc. 2003 Aug;35(8):1381–95.

7. International Physical Activity Questionnaire (IPAQ). Guidelines for Data Processing and Analysis of the International Physical Activity Questionnaire (IPAQ) – Short and Long Forms [Internet]. [cited 2023 Jul 30]. Available from: https://sites.google.com/view/ipaq/score

8. Phelps NH, Singleton RK, Zhou B, Heap RA, Mishra A, Bennett JE, et al. Worldwide trends in underweight and obesity from 1990 to 2022: a pooled analysis of 3663 population-representative studies with 222 million children, adolescents, and adults. The Lancet [Internet]. 2024 Feb 29 [cited 2024 Mar 3];0(0). Available from: https://www.thelancet.com/journals/lancet/article/PIIS0140-6736(23)02750-2/fulltext

9. Ainsworth BE, Macera CA, Jones DA, Reis JP, Addy CL, Bowles HR, et al. Comparison of the 2001 BRFSS and the IPAQ Physical Activity Questionnaires. Med Sci Sports Exerc. 2006 Sep;38(9):1584–92.

10. Scholes S, Bridges S, Fat LN, Mindell JS. Comparison of the Physical Activity and Sedentary Behaviour Assessment Questionnaire and the Short-Form International Physical Activity Questionnaire: An Analysis of Health Survey for England Data. PLOS ONE. 2016 Mar 18;11(3):e0151647.

11. United Nations, Department of Economic and Social Affairs, Population Division. World Urbanization Prospects: The 2018 Revision, Online Edition [Internet]. 2018. Available from: https://population.un.org/wup/

12. Bentham J, Di Cesare M, Bilano V, Bixby H, Zhou B, Stevens GA, et al. Worldwide trends in body-mass index, underweight, overweight, and obesity from 1975 to 2016: a pooled analysis of 2416 population-based measurement studies in 128·9 million children, adolescents, and adults. The Lancet. 2017 Dec;390(10113):2627–42.

13. Bürkner PC. Advanced {Bayesian} Multilevel Modeling with the {R} Package {brms}. R J. 2018;10(1):395–411.

14. Bürkner PC, Gabry J, Weber S, Johnson A, Modrak M, Badr HS, et al. brms: Bayesian Regression Models using "Stan" [Internet]. 2023 [cited 2023 Aug 3]. Available from: https://cran.r-project.org/web/packages/brms/index.html

15. Ahmad OB, Boschi-Pinto C, Lopez AD, Murray CJL, Lozano R, Inoue M. Age standardization of rates: A new WHO standard [Internet]. 2001 [cited 2023 Oct 31]. Available from:

https://cdn.who.int/media/docs/default-source/gho-documents/global-health-estimates/gpe_discussion_paper_series_paper31_2001_age_standardization_rates.pdf

16. The World Bank Group. World Bank Analytical Classifications [Internet]. 2023. Available from: http://databank.worldbank.org/data/download/site-content/OGHIST.xlsx

17. World Health Organization. Discussion Paper on the Development of an Implementation Roadmap 2023-2030 for the Global Action Plan for the Prevention and Control of NCDs 2013-2020 [Internet]. Geneva, Switzerland: World Health Organization; 2021. Available from: https://cdn.who.int/media/docs/default-source/documents/health-topics/non-communicable-diseases/eb150---who-discussion-paper-on-ncd-roadmap-development-(20-aug-2021)---for-web.pdf
